# Global mortality associated with 33 bacterial pathogens in 2019: a systematic analysis for the Global Burden of Disease Study 2019

**DOI:** 10.1016/S0140-6736(22)02185-7

**Published:** 2022-12-17

**Authors:** GBD 2019 Antimicrobial Resistance Collaborators, Kevin S Ikuta, Lucien R Swetschinski, Gisela Robles Aguilar, Fablina Sharara, Tomislav Mestrovic, Authia P Gray, Nicole Davis Weaver, Eve E Wool, Chieh Han, Anna Gershberg Hayoon, Amirali Aali, Semagn Mekonnen Abate, Mohsen Abbasi-Kangevari, Zeinab Abbasi-Kangevari, Sherief Abd-Elsalam, Getachew Abebe, Aidin Abedi, Amir Parsa Abhari, Hassan Abidi, Richard Gyan Aboagye, Abdorrahim Absalan, Hiwa Abubaker Ali, Juan Manuel Acuna, Tigist Demssew Adane, Isaac Yeboah Addo, Oyelola A Adegboye, Mohammad Adnan, Qorinah Estiningtyas Sakilah Adnani, Muhammad Sohail Afzal, Saira Afzal, Zahra Babaei Aghdam, Bright Opoku Ahinkorah, Aqeel Ahmad, Araz Ramazan Ahmad, Rizwan Ahmad, Sajjad Ahmad, Sohail Ahmad, Sepideh Ahmadi, Ali Ahmed, Haroon Ahmed, Jivan Qasim Ahmed, Tarik Ahmed Rashid, Marjan Ajami, Budi Aji, Mostafa Akbarzadeh-Khiavi, Chisom Joyqueenet Akunna, Hanadi Al Hamad, Fares Alahdab, Ziyad Al-Aly, Mamoon A Aldeyab, Alicia V Aleman, Fadwa Alhalaiqa Naji Alhalaiqa, Robert Kaba Alhassan, Beriwan Abdulqadir Ali, Liaqat Ali, Syed Shujait Ali, Yousef Alimohamadi, Vahid Alipour, Atiyeh Alizadeh, Syed Mohamed Aljunid, Kasim Allel, Sami Almustanyir, Edward Kwabena Ameyaw, Arianna Maever L Amit, Nivedita Anandavelane, Robert Ancuceanu, Catalina Liliana Andrei, Tudorel Andrei, Dewi Anggraini, Adnan Ansar, Anayochukwu Edward Anyasodor, Jalal Arabloo, Aleksandr Y Aravkin, Demelash Areda, Timur Aripov, Anton A Artamonov, Judie Arulappan, Raphael Taiwo Aruleba, Muhammad Asaduzzaman, Tahira Ashraf, Seyyed Shamsadin Athari, Daniel Atlaw, Sameh Attia, Marcel Ausloos, Tewachew Awoke, Beatriz Paulina Ayala Quintanilla, Tegegn Mulatu Ayana, Sina Azadnajafabad, Amirhossein Azari Jafari, Darshan B B, Muhammad Badar, Ashish D Badiye, Nayereh Baghcheghi, Sara Bagherieh, Atif Amin Baig, Indrajit Banerjee, Aleksandra Barac, Mainak Bardhan, Francesco Barone-Adesi, Hiba Jawdat Barqawi, Amadou Barrow, Pritish Baskaran, Saurav Basu, Abdul-Monim Mohammad Batiha, Neeraj Bedi, Melaku Ashagrie Belete, Uzma Iqbal Belgaumi, Rose G Bender, Bharti Bhandari, Dinesh Bhandari, Pankaj Bhardwaj, Sonu Bhaskar, Krittika Bhattacharyya, Suraj Bhattarai, Saeid Bitaraf, Danilo Buonsenso, Zahid A Butt, Florentino Luciano Caetano dos Santos, Jiao Cai, Daniela Calina, Paulo Camargos, Luis Alberto Cámera, Rosario Cárdenas, Muge Cevik, Joshua Chadwick, Jaykaran Charan, Akhilanand Chaurasia, Patrick R Ching, Sonali Gajanan Choudhari, Enayet Karim Chowdhury, Fazle Rabbi Chowdhury, Dinh-Toi Chu, Isaac Sunday Chukwu, Omid Dadras, Fentaw Teshome Dagnaw, Xiaochen Dai, Saswati Das, Anna Dastiridou, Sisay Abebe Debela, Fitsum Wolde Demisse, Solomon Demissie, Diriba Dereje, Msganaw Derese, Hardik Dineshbhai Desai, Fikadu Nugusu Dessalegn, Samuel Abebe A Dessalegni, Belay Desye, Kartik Dhaduk, Meghnath Dhimal, Sameer Dhingra, Nancy Diao, Daniel Diaz, Shirin Djalalinia, Milad Dodangeh, Deepa Dongarwar, Bezabih Terefe Dora, Fariba Dorostkar, Haneil Larson Dsouza, Eleonora Dubljanin, Susanna J Dunachie, Oyewole Christopher Durojaiye, Hisham Atan Edinur, Habtamu Bekele Ejigu, Michael Ekholuenetale, Temitope Cyrus Ekundayo, Hassan El-Abid, Muhammed Elhadi, Mohamed A Elmonem, Amir Emami, Luchuo Engelbert Bain, Daniel Berhanie Enyew, Ryenchindorj Erkhembayar, Babak Eshrati, Farshid Etaee, Adeniyi Francis Fagbamigbe, Shahab Falahi, Aida Fallahzadeh, Emerito Jose A Faraon, Ali Fatehizadeh, Ginenus Fekadu, João C Fernandes, Allegra Ferrari, Getahun Fetensa, Irina Filip, Florian Fischer, Masoud Foroutan, Peter Andras Gaal, Muktar A Gadanya, Abhay Motiramji Gaidhane, Balasankar Ganesan, Mesfin Gebrehiwot, Reza Ghanbari, Mohammad Ghasemi Nour, Ahmad Ghashghaee, Ali Gholamrezanezhad, Abdolmajid Gholizadeh, Mahaveer Golechha, Pouya Goleij, Davide Golinelli, Amador Goodridge, Damitha Asanga Gunawardane, Yuming Guo, Rajat Das Gupta, Sapna Gupta, Veer Bala Gupta, Vivek Kumar Gupta, Alemu Guta, Parham Habibzadeh, Atlas Haddadi Avval, Rabih Halwani, Asif Hanif, Md. Abdul Hannan, Harapan Harapan, Shoaib Hassan, Hadi Hassankhani, Khezar Hayat, Behzad Heibati, Golnaz Heidari, Mohammad Heidari, Reza Heidari-Soureshjani, Claudiu Herteliu, Demisu Zenbaba Heyi, Kamal Hezam, Praveen Hoogar, Nobuyuki Horita, Md Mahbub Hossain, Mehdi Hosseinzadeh, Mihaela Hostiuc, Sorin Hostiuc, Soodabeh Hoveidamanesh, Junjie Huang, Salman Hussain, Nawfal R Hussein, Segun Emmanuel Ibitoye, Olayinka Stephen Ilesanmi, Irena M Ilic, Milena D Ilic, Mohammad Tarique Imam, Mustapha Immurana, Leeberk Raja Inbaraj, Arnaud Iradukunda, Nahlah Elkudssiah Ismail, Chidozie C D Iwu, Chinwe Juliana Iwu, Linda Merin J, Mihajlo Jakovljevic, Elham Jamshidi, Tahereh Javaheri, Fatemeh Javanmardi, Javad Javidnia, Sathish Kumar Jayapal, Umesh Jayarajah, Rime Jebai, Ravi Prakash Jha, Tamas Joo, Nitin Joseph, Farahnaz Joukar, Jacek Jerzy Jozwiak, Salah Eddine Oussama Kacimi, Vidya Kadashetti, Laleh R Kalankesh, Rohollah Kalhor, Vineet Kumar Kamal, Himal Kandel, Neeti Kapoor, Samad Karkhah, Bekalu Getnet Kassa, Nicholas J Kassebaum, Patrick DMC Katoto, Mohammad Keykhaei, Himanshu Khajuria, Abbas Khan, Imteyaz A Khan, Maseer Khan, Md Nuruzzaman Khan, Moien AB Khan, Moawiah Mohammad Khatatbeh, Mona M Khater, Hamid Reza Khayat Kashani, Jagdish Khubchandani, Hanna Kim, Min Seo Kim, Ruth W Kimokoti, Niranjan Kissoon, Sonali Kochhar, Farzad Kompani, Soewarta Kosen, Parvaiz A Koul, Sindhura Lakshmi Koulmane Laxminarayana, Fiorella Krapp Lopez, Kewal Krishan, Vijay Krishnamoorthy, Vishnutheertha Kulkarni, Naveen Kumar, Om P Kurmi, Ambily Kuttikkattu, Hmwe Hmwe Kyu, Dharmesh Kumar Lal, Judit Lám, Iván Landires, Savita Lasrado, Sang-woong Lee, Jacopo Lenzi, Sonia Lewycka, Shanshan Li, Stephen S Lim, Wei Liu, Rakesh Lodha, Michael J Loftus, Ayush Lohiya, László Lorenzovici, Mojgan Lotfi, Ata Mahmoodpoor, Mansour Adam Mahmoud, Razzagh Mahmoudi, Azeem Majeed, Jamal Majidpoor, Alaa Makki, Galana Ayana Mamo, Yosef Manla, Miquel Martorell, Clara N Matei, Barney McManigal, Entezar Mehrabi Nasab, Ravi Mehrotra, Addisu Melese, Oliver Mendoza-Cano, Ritesh G Menezes, Alexios-Fotios A Mentis, Georgia Micha, Irmina Maria Michalek, Ana Carolina Micheletti Gomide Nogueira de Sá, Neda Milevska Kostova, Shabir Ahmad Mir, Mojgan Mirghafourvand, Seyyedmohammadsadeq Mirmoeeni, Erkin M Mirrakhimov, Mohammad Mirza-Aghazadeh-Attari, Abay Sisay Misganaw, Awoke Misganaw, Sanjeev Misra, Esmaeil Mohammadi, Mokhtar Mohammadi, Abdollah Mohammadian-Hafshejani, Shafiu Mohammed, Syam Mohan, Mohammad Mohseni, Ali H Mokdad, Sara Momtazmanesh, Lorenzo Monasta, Catrin E Moore, Maryam Moradi, Mostafa Moradi Sarabi, Shane Douglas Morrison, Majid Motaghinejad, Haleh Mousavi Isfahani, Amin Mousavi Khaneghah, Seyed Ali Mousavi-Aghdas, Sumaira Mubarik, Francesk Mulita, Getaneh Baye B Mulu, Sandra B Munro, Saravanan Muthupandian, Tapas Sadasivan Nair, Atta Abbas Naqvi, Himanshi Narang, Zuhair S Natto, Muhammad Naveed, Biswa Prakash Nayak, Shumaila Naz, Ionut Negoi, Seyed Aria Nejadghaderi, Sandhya Neupane Kandel, Che Henry Ngwa, Robina Khan Niazi, Antonio Tolentino Nogueira de Sá, Nafise Noroozi, Hasti Nouraei, Ali Nowroozi, Virginia Nuñez-Samudio, Jerry John Nutor, Chimezie Igwegbe Nzoputam, Ogochukwu Janet Nzoputam, Bogdan Oancea, Rahman Md Obaidur, Vivek Anand Ojha, Akinkunmi Paul Okekunle, Osaretin Christabel Okonji, Andrew T Olagunju, Bolajoko Olubukunola Olusanya, Ahmed Omar Bali, Emad Omer, Nikita Otstavnov, Bilcha Oumer, Mahesh P A, Jagadish Rao Padubidri, Keyvan Pakshir, Tamás Palicz, Adrian Pana, Shahina Pardhan, Jose L Paredes, Utsav Parekh, Eun-Cheol Park, Seoyeon Park, Ashish Pathak, Rajan Paudel, Uttam Paudel, Shrikant Pawar, Hamidreza Pazoki Toroudi, Minjin Peng, Umberto Pensato, Veincent Christian Filipino Pepito, Marcos Pereira, Mario F P Peres, Norberto Perico, Ionela-Roxana Petcu, Zahra Zahid Piracha, Indrashis Podder, Nayanum Pokhrel, Ramesh Poluru, Maarten J Postma, Naeimeh Pourtaheri, Akila Prashant, Ibrahim Qattea, Mohammad Rabiee, Navid Rabiee, Amir Radfar, Saber Raeghi, Sima Rafiei, Pankaja Raghav Raghav, Leila Rahbarnia, Vafa Rahimi-Movaghar, Mosiur Rahman, Muhammad Aziz Rahman, Amir Masoud Rahmani, Vahid Rahmanian, Pradhum Ram, Muhammad Modassar Ali Nawaz Ranjha, Sowmya J Rao, Mohammad-Mahdi Rashidi, Azad Rasul, Zubair Ahmed Ratan, Salman Rawaf, Reza Rawassizadeh, Mohammad Sadegh Razeghinia, Elrashdy Moustafa Mohamed Redwan, Misganu Teshoma Regasa, Giuseppe Remuzzi, Melese Abate Reta, Nazila Rezaei, Aziz Rezapour, Abanoub Riad, Rezaul Karim Ripon, Kristina E Rudd, Basema Saddik, Saeid Sadeghian, Umar Saeed, Mohsen Safaei, Azam Safary, Sher Zaman Safi, Maryam Sahebazzamani, Amirhossein Sahebkar, Harihar Sahoo, Saina Salahi, Sarvenaz Salahi, Hedayat Salari, Sana Salehi, Hossein Samadi Kafil, Abdallah M Samy, Nima Sanadgol, Senthilkumar Sankararaman, Francesco Sanmarchi, Brijesh Sathian, Monika Sawhney, Ganesh Kumar Saya, Subramanian Senthilkumaran, Allen Seylani, Pritik A Shah, Masood Ali Shaikh, Elaheh Shaker, Murad Ziyaudinovich Shakhmardanov, Mequannent Melaku Sharew, Athena Sharifi-Razavi, Purva Sharma, Rahim Ali Sheikhi, Ali Sheikhy, Pavanchand H Shetty, Mika Shigematsu, Jae Il Shin, Hesamaddin Shirzad-Aski, K M Shivakumar, Parnian Shobeiri, Seyed Afshin Shorofi, Sunil Shrestha, Migbar Mekonnen Sibhat, Negussie Boti Sidemo, Mustafa Kamal Sikder, Luís Manuel Lopes Rodrigues Silva, Jasvinder A Singh, Paramdeep Singh, Surjit Singh, Md Shahjahan Siraj, Samarjeet Singh Siwal, Valentin Yurievich Skryabin, Anna Aleksandrovna Skryabina, Bogdan Socea, Damtew Damtew Solomon, Yimeng Song, Chandrashekhar T Sreeramareddy, Muhammad Suleman, Rizwan Suliankatchi Abdulkader, Saima Sultana, Miklós Szócska, Seyed-Amir Tabatabaeizadeh, Mohammad Tabish, Majid Taheri, Elahe Taki, Ker-Kan Tan, Sarmila Tandukar, Nathan Y Tat, Vivian Y Tat, Belay Negash Tefera, Yibekal Manaye Tefera, Gebremaryam Temesgen, Mohamad-Hani Temsah, Samar Tharwat, Arulmani Thiyagarajan, Imad I Tleyjeh, Christopher E Troeger, Krishna Kishore Umapathi, Era Upadhyay, Sahel Valadan Tahbaz, Pascual R Valdez, Jef Van den Eynde, H. Rogier van Doorn, Siavash Vaziri, Georgios-Ioannis Verras, Harimadhav Viswanathan, Bay Vo, Abdul Waris, Gizachew Tadesse Wassie, Nuwan Darshana Wickramasinghe, Sajad Yaghoubi, Gahin Abdulraheem Tayib Yahya Yahya, Seyed Hossein Yahyazadeh Jabbari, Arzu Yigit, Vahit Yiğit, Dong Keon Yon, Naohiro Yonemoto, Mazyar Zahir, Burhan Abdullah Zaman, Sojib Bin Zaman, Moein Zangiabadian, Iman Zare, Mikhail Sergeevich Zastrozhin, Zhi-Jiang Zhang, Peng Zheng, Chenwen Zhong, Mohammad Zoladl, Alimuddin Zumla, Simon I Hay, Christiane Dolecek, Benn Sartorius, Christopher J L Murray, Mohsen Naghavi

## Abstract

**Background:**

Reducing the burden of death due to infection is an urgent global public health priority. Previous studies have estimated the number of deaths associated with drug-resistant infections and sepsis and found that infections remain a leading cause of death globally. Understanding the global burden of common bacterial pathogens (both susceptible and resistant to antimicrobials) is essential to identify the greatest threats to public health. To our knowledge, this is the first study to present global comprehensive estimates of deaths associated with 33 bacterial pathogens across 11 major infectious syndromes.

**Methods:**

We estimated deaths associated with 33 bacterial genera or species across 11 infectious syndromes in 2019 using methods from the Global Burden of Diseases, Injuries, and Risk Factors Study (GBD) 2019, in addition to a subset of the input data described in the Global Burden of Antimicrobial Resistance 2019 study. This study included 343 million individual records or isolates covering 11 361 study-location-years. We used three modelling steps to estimate the number of deaths associated with each pathogen: deaths in which infection had a role, the fraction of deaths due to infection that are attributable to a given infectious syndrome, and the fraction of deaths due to an infectious syndrome that are attributable to a given pathogen. Estimates were produced for all ages and for males and females across 204 countries and territories in 2019. 95% uncertainty intervals (UIs) were calculated for final estimates of deaths and infections associated with the 33 bacterial pathogens following standard GBD methods by taking the 2·5th and 97·5th percentiles across 1000 posterior draws for each quantity of interest.

**Findings:**

From an estimated 13·7 million (95% UI 10·9–17·1) infection-related deaths in 2019, there were 7·7 million deaths (5·7–10·2) associated with the 33 bacterial pathogens (both resistant and susceptible to antimicrobials) across the 11 infectious syndromes estimated in this study. We estimated deaths associated with the 33 bacterial pathogens to comprise 13·6% (10·2–18·1) of all global deaths and 56·2% (52·1–60·1) of all sepsis-related deaths in 2019. Five leading pathogens—*Staphylococcus aureus, Escherichia coli, Streptococcus pneumoniae, Klebsiella pneumoniae*, and *Pseudomonas aeruginosa*—were responsible for 54·9% (52·9–56·9) of deaths among the investigated bacteria. The deadliest infectious syndromes and pathogens varied by location and age. The age-standardised mortality rate associated with these bacterial pathogens was highest in the sub-Saharan Africa super-region, with 230 deaths (185–285) per 100 000 population, and lowest in the high-income super-region, with 52·2 deaths (37·4–71·5) per 100 000 population. *S aureus* was the leading bacterial cause of death in 135 countries and was also associated with the most deaths in individuals older than 15 years, globally. Among children younger than 5 years, *S pneumoniae* was the pathogen associated with the most deaths. In 2019, more than 6 million deaths occurred as a result of three bacterial infectious syndromes, with lower respiratory infections and bloodstream infections each causing more than 2 million deaths and peritoneal and intra-abdominal infections causing more than 1 million deaths.

**Interpretation:**

The 33 bacterial pathogens that we investigated in this study are a substantial source of health loss globally, with considerable variation in their distribution across infectious syndromes and locations. Compared with GBD Level 3 underlying causes of death, deaths associated with these bacteria would rank as the second leading cause of death globally in 2019; hence, they should be considered an urgent priority for intervention within the global health community. Strategies to address the burden of bacterial infections include infection prevention, optimised use of antibiotics, improved capacity for microbiological analysis, vaccine development, and improved and more pervasive use of available vaccines. These estimates can be used to help set priorities for vaccine need, demand, and development.

**Funding:**

Bill & Melinda Gates Foundation, Wellcome Trust, and Department of Health and Social Care, using UK aid funding managed by the Fleming Fund.

**Research in context**

**Evidence before this study**

Communicable diseases have long been recognised as a cause of substantial health loss globally, but few studies to date have concentrated on pathogen-specific mortality caused by common bacterial pathogens. Many estimates exist for pathogens like *Mycobacterium tuberculosis*, *Plasmodium* spp, and HIV but estimates of the burden of bacterial infections have been restricted to either a small number of locations, specific populations (such as invasive pneumococcal disease in children younger than 5 years), or a small number of bacteria in the context of the scope of infectious syndromes (eg, *Streptococcus pneumoniae* and *Neisseria meningitidis* as a cause of meningitis). The US Centers for Disease Control and Prevention (CDC) Active Bacterial Core surveillance and Emerging Infections Program, and the European CDC's European Antimicrobial Resistance Surveillance Network have provided crucial estimates of selected invasive bacterial infections in high-income countries. These estimates are important first steps in building our understanding of the burden of specific bacterial infections but they provide an incomplete picture: within the locations with the greatest infectious burden, the mortality associated with these pathogens remains unknown, making it difficult to set global public health priorities.

**Added value of this study**

To our knowledge, this is the first study to produce global estimates of mortality associated with 33 clinically significant bacterial pathogens (including those susceptible to antibacterial compounds) across 11 infectious syndromes, and to provide these estimates for all ages and for males and females across 204 countries and territories in 2019. This analysis is intended to provide an audit of the mortality associated with common bacterial pathogens. We estimated the number of deaths associated with each of these bacterial pathogens using three modelling steps: deaths where infection had a role, the fraction of deaths due to infection attributable to a given infectious syndrome, and the fraction of deaths due to infectious syndromes attributable to a given pathogen. Deaths in which infection had a role were estimated using the number of deaths for which either the underlying cause of death was infectious or the pathway of death was through sepsis. The fraction of deaths due to infection attributable to a given infectious syndrome was estimated using data to determine the infectious syndrome responsible for sepsis by underlying cause of death, age, sex, and geographical location. The fraction of deaths due to an infectious syndrome attributable to a given pathogen was estimated by integrating estimates of pathogen-specific and syndrome-specific case-fatality ratios with modelled pathogen distributions for each infectious syndrome that varied by age and geographical location.

**Implications of all the available evidence**

Our findings show that more than half of all global bacterial deaths in 2019 were due to five bacterial pathogens: *Staphylococcus aureus, Escherichia coli*, *Streptococcus pneumoniae*, *Klebsiella pneumoniae,* and *Pseudomonas aeruginosa.* The substantial burden of health loss associated with these five pathogens requires increased attention from the global health community and collaborative intervention approaches. Understanding the leading infectious syndromes and pathogens for each region is of the utmost importance so that targeted prevention efforts can be implemented. This study can be used to guide strategies for reducing the burden of bacterial infectious diseases, including infection prevention and control measures, vaccine development and implementation, and the availability of basic acute care services.

## Introduction

Communicable diseases have long been highlighted as a global public health priority and are recognised as a leading cause of health loss globally.^1^, ^2^, ^3^ A recent study estimated that there were more than 10 million sepsis-related deaths in 2017, indicating that infections were involved in more than 20% of deaths globally for that year.^4^ Reducing the number of deaths due to infections is a foundational principle in moving towards health equity^5^ because there is a disproportionate infectious burden in low-income and middle-income countries (LMICs).^4^, ^6^ Preventing and effectively treating infections is also essential to achieving Sustainable Development Goal (SDG) 3: ensure healthy lives and promote wellbeing for all at all ages.^7^ Although the contribution of non-bacterial causes (eg, fungal infections, malaria, and HIV) to the overall infection burden must be acknowledged, reducing the number of cases and health impact of bacterial infectious diseases is a priority area that necessitates a multipronged approach with infection prevention and control measures;^8^ vaccine development, deployment, and uptake;^9^, ^10^ and early and effective case management.^11^, ^12^ Detailed estimates of the number of deaths related to bacterial infections and their causes are an important step in tracking progress towards global health goals and are essential to inform priorities for vaccine and drug development.

To date, no global burden estimates exist for many common bacterial pathogens, making establishment of public health priorities difficult. The few estimates that do exist are often constrained to specific pathogens,^13^ infectious syndromes,^14^ or high-income countries.^15^ For example, global estimates of the burden of *Streptococcus pneumoniae* are available; however, these estimates are mostly restricted to children younger than 5 years^13^ or as a cause of pneumonia or meningitis^3^ and do not reflect the total burden across all populations and all infectious syndromes.^16^ Estimates of selected invasive bacterial infections exist in high-income countries that use passive surveillance systems, such as the US Centers for Disease Control (CDC) Active Bacterial Core surveillance and Emerging Infections Program^17^ and the European CDC's European Antimicrobial Resistance Surveillance Network.^18^ Although such estimates offer important insights, no comprehensive estimates exist covering all locations for a broad range of bacteria across major infectious syndromes. Notably absent are country-level estimates for LMICs, which have the greatest burden of infectious diseases,^4^ as also emphasised by the recent Global Burden of Antimicrobial Resistance 2019 study.^19^ For this reason, there has been profound neglect of these pathogens, and relevant infectious syndromes, in global advocacy campaigns aiming to maximise life-saving interventions.

In this study, we present, to our knowledge, the first global estimates of deaths associated with 33 clinically significant bacterial pathogens (both susceptible and resistant to antimicrobials), across 11 infectious syndromes in 2019. We used data obtained from the Global Burden of Diseases, Injuries, and Risk Factors Study (GBD) 2019^3^ and the Global Burden of Antimicrobial Resistance 2019 study^19^ to estimate global, regional, and national mortality and years of life lost (YLLs) associated with these 33 bacterial pathogens across 204 countries and territories and 286 underlying causes of death, by age and sex, in 2019. This manuscript was produced as part of the GBD Collaborator Network and in accordance with the GBD Protocol.^20^

## Methods

### Overview

In this study, we estimated the fatal burden associated with infection caused by 33 bacterial species or genera across 11 infectious syndromes using methods and data from the GBD 2019 and Global Burden of Antimicrobial Resistance studies.^3^, ^19^ Detailed methods have been published elsewhere.^19^ Briefly, using 343 million individual records or isolates covering 11 361 study-location-years, we implemented three modelling steps to estimate the number of deaths associated with each bacterial pathogen across 204 countries and territories for 2019. First, we estimated the overall number of deaths in which infection had a role using methods described in the Global Burden of Antimicrobial Resistance study.^19^ Second, we determined the infectious syndrome responsible for each death due to an infection. Finally, for each infectious syndrome we estimated the distribution of pathogens responsible. With the use of these components, we estimated the number of deaths associated with each of the 33 bacterial pathogens of interest in this study. A summarising flowchart and detailed approach description for each step of the estimation process are in appendix 1 (section 10). All estimates were produced by age, for males and females, and for 204 countries and territories.

We followed Preferred Reporting Items for Systematic Reviews and Meta-Analyses (PRISMA) guidelines^21^ throughout the study (detailed in appendix 1 [section 7]). This study complies with the Guidelines for Accurate and Transparent Health Estimates Reporting (GATHER) recommendations.^22^ The complete GATHER checklist is provided in appendix 1 (section 8).

### Input data

We used a subset of the input data described in the Global Burden of Antimicrobial Resistance study to estimate mortality burden by pathogen.^19^ We selected data inputs only if they were based on a representative sampling framework that would not bias the aetiology estimation towards a specific pathogen (eg, we did not deliberately sample 100 cases of every pathogen). The input data source types that met these criteria were: multiple-cause-of-death and vital registration data; hospital discharge data; linkage data sources; mortality surveillance in the Child Health and Mortality Prevention Surveillance (CHAMPS) study; literature reviews of the microbial cause of meningitis, neonatal sepsis, lower respiratory infections, urinary tract infections, skin infections, peritonitis, and bone and joint infections; and laboratory-based passive surveillance data. We used multiple-cause-of-death and vital registration data, hospital discharge data, CHAMPS, and linkage data sources to estimate the number of deaths for which infection had a role and the distribution of infectious syndromes (appendix 1 [section 4]). We used data from CHAMPS, literature reviews, and laboratory-based passive surveillance systems to estimate the pathogen distribution for each infectious syndrome (appendix 1 [section 6]). The number of individual records or isolates used in each step for each of the GBD regions is shown in appendix 1 (p 62).

### Deaths in which infection played a role

Detailed methods on how the number of deaths in which infection played a role were estimated have been published previously.^4^ Briefly, we estimated the number of deaths for which either the underlying cause of death was infectious (using GBD 2019 estimates) or for which a contributing factor in the death was sepsis and the underlying cause was non-infectious. For the identification of sepsis in non-infectious underlying causes of death, we used the following data inputs: 121 million cause-of-death records with multiple-cause-of-death data from eight countries and territories; 192 million hospital records with patient discharge status from seven countries and territories; 264 000 multiple-cause-of-death records linked to hospital records from ten countries and territories; and 849 deaths from CHAMPS sites across six countries. We developed a random-effects logistic regression model to predict the fraction of deaths involving sepsis for each underlying cause of death, age, sex, and geographical location using methods described previously.^4^, ^19^ Using this cause-fraction, we estimated the number of deaths for which the underlying cause was non-infectious and sepsis occurred. We then added this to the number of deaths in which the underlying cause was infectious from GBD 2019 to estimate the number of deaths in which infection had a role.

### Infectious syndrome estimates

Detailed methods on the estimation process for infectious syndromes have been published previously^19^ and are in appendix 1 (section 4). Briefly, we used the available data described in the Input data section (multiple cause of death, hospital data with patient discharge status, linkage data, and CHAMPS) to determine the infectious syndrome responsible for sepsis by underlying cause, age, sex, and geographical location. Within our modelling framework, an infectious syndrome is the infection directly responsible for sepsis and serves as the bridge between the underlying cause of death and sepsis. We estimated 11 infectious syndromes: meningitis and other bacterial CNS infections; cardiac infections; peritoneal and intra-abdominal infections; lower respiratory infections and all related infections in the thorax; bacterial infections of the skin and subcutaneous systems; infections of bones, joints, and related organs; typhoid, paratyphoid, and invasive non-typhoidal *Salmonella*; diarrhoea; urinary tract infections and pyelonephritis; bloodstream infections; and gonorrhoea and chlamydia. We then used syndrome-and-age-specific mixed effects logistic regression models (using sex, Healthcare Access and Quality Index, and syndrome-specific bias covariates and a nested random effect on underlying cause) to estimate the fraction of sepsis-related deaths that were caused by each infectious syndrome for each underlying cause of death, age, sex, and geographical location. Applying this fraction to the estimate of number of infection-related deaths from the previous step, we determined the number of deaths that occurred due to a given infectious syndrome by underlying cause of death, age, sex, and geographical location. We estimated deaths with an infectious syndrome as the sum of deaths with the syndrome as an underlying cause of death (ie, for those syndromes considered to be underlying causes) plus deaths with a non-infectious underlying cause where the syndrome was estimated to occur (eg, all deaths where the underlying cause was meningitis plus all road traffic injury deaths in which meningitis occurred). Bloodstream infections; infections of bones, joints, and related organs; and peritoneal and intra-abdominal infections are not estimated in GBD, so for these three infectious syndromes, we assumed they had a non-infectious underlying cause to estimate deaths.

### Pathogen distribution

Detailed methods on the estimation process for pathogen distribution have been published previously^19^ and are in appendix 1 (sections 5 and 6), including exceptions and special handling decisions. Briefly, we used data from 343 million isolates covering 11 361 study-location-years to estimate pathogen distributions for each infectious syndrome that varied by age and location, with a subset of this data adapted to calculate pathogen-specific and syndrome-specific case-fatality ratios (CFRs). We selected a set of pathogens to be explicitly estimated as part of the cause of each infectious syndrome. This selection was based on several factors. First, selection was influenced by the prevalence of each pathogen in the raw data, because the amount of available data restricts the number of pathogens that can be estimated successfully. Second, we aimed to produce estimates for the combination of pathogens that, collectively, represented at least 85% of the aetiological causes of a given infectious syndrome. We included three residual categories: (1) other bacteria and (2) polymicrobial—for bacteria that did not meet these criteria or had two or more bacteria isolated from a single isolate—and (3) non-bacterial pathogen, for pathogens that were not bacteria (ie, viruses, fungi, or parasites).

Much of the input data on pathogen distribution were only reported on a subset of pathogens, such that they did not have a complete denominator for all possible pathogens. For example, many surveillance systems for meningitis only monitor *S pneumoniae* and *Neisseria meningitidis* as the causative pathogen. To account for this partial distribution, we used a network meta-analysis, which allowed us to include any dataset reporting on two or more pathogens. We implemented this network meta-analysis using the multinomial estimation with partial and composite observations (MEPCO) modelling environment. This approach allowed us to include covariates in the network analysis, incorporate Bayesian priors (ie, prior probability distributions), and use data that compared one pathogen with all other pathogens. Input data for the MEPCO process consisted of ratios of sums of cases within a study (with each sum representing a specific pathogen or combination of pathogens). The model was fit by minimising the sum of the residuals between log-transformed observed ratios and predictions using a non-linear likelihood minimisation problem optimised using the Gauss-Newton method^23^ (appendix 1 [section 6.3.1]). The resultant MEPCO estimate was the non-fatal pathogen distribution for each infectious syndrome.

To estimate the fatal pathogen distribution, we calculated syndrome-specific and pathogen-specific CFRs using data that linked pathogen-specific disease incidence to deaths and the meta-regression–Bayesian regularised, trimmed (MR-BRT) tool. We estimated CFRs as a function of age, Healthcare Access and Quality Index, and various bias covariates that were specific to the nuances of the data for each infectious syndrome (appendix 1 [section 5]). We then used the pathogen-specific and syndrome-specific CFRs to produce a pathogen distribution of number of deaths estimated for each infectious syndrome by age and location. Our modelling framework accounted for both data-rich and data-sparse pathogens (appendix 1 [section 5.3]). In this analysis we do not report estimates for *Mycobacterium tuberculosis* because this specific pathogen is already part of a global strategy with well delineated surveillance and data-driven control plans, and the motivation for the current study was to provide insight into the public health burden of less well studied pathogens.

### Estimating mortality and YLLs

To estimate the number of deaths due to the pathogens of interest, we multiplied the number of deaths for each underlying cause, age, sex, and location by the fraction of deaths in which infection had a role, the fatal infectious syndrome fraction, and the pathogen fraction, and summed across all underlying causes of death and infectious syndromes to estimate the number of deaths due to a given pathogen by age, sex, and location. We estimated YLLs associated with each pathogen using previously published methods^3^ that convert age-specific deaths into YLLs using the standard counterfactual life expectancy at each age.

### Uncertainty and validity analysis

Following standard GBD methods,^16^ we propagated uncertainty from each step of the analysis into the final estimates of deaths associated with each pathogen by taking the 2·5th and 97·5th percentiles of 1000 draws from the posterior distribution of each quantity of interest. To assess model validity, we calculated the root mean square error and coefficient of determination (*R*^2^) for each pathogen distribution model in proportion space for both in-sample and out-of-sample predictions (appendix 1 [section 6.5]).

### Role of the funding source

The funders of the study had no role in study design, data collection, data analysis, data interpretation, or the writing of the report.

## Results

**Figure 1:**
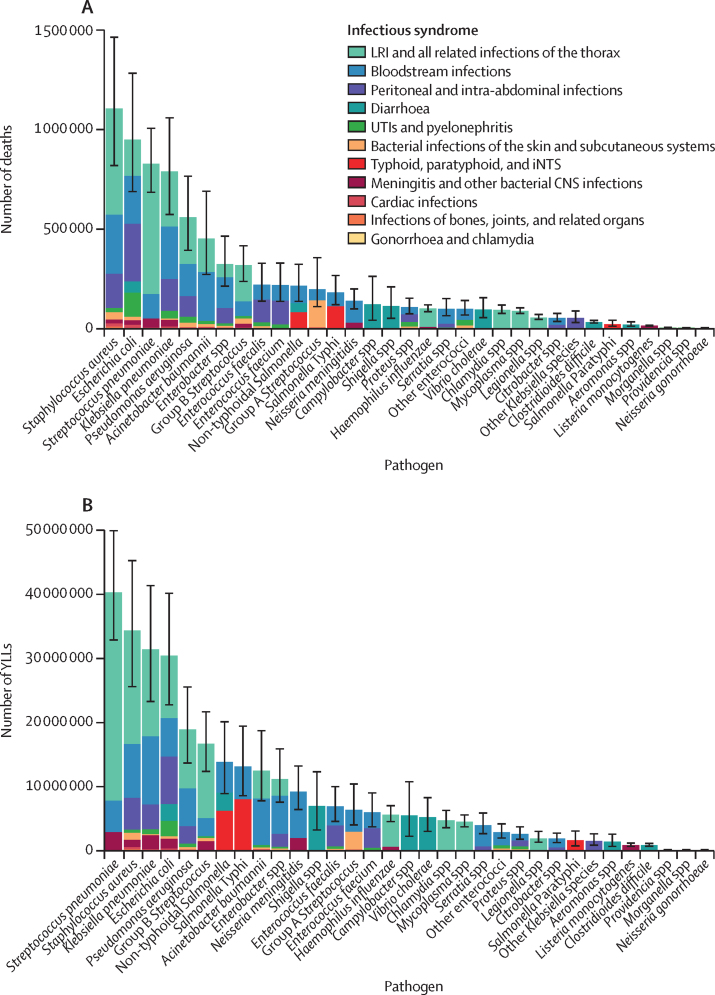
Global number of deaths (A) and YLLs (B), by pathogen and infectious syndrome, 2019 Columns show total number of deaths for each pathogen, with error bars showing 95% uncertainty intervals, with the bars split into infectious syndromes. LRI=lower respiratory infection. iNTS=invasive non-typhoidal *Salmonella. Salmonella* Typhi=*Salmonella enterica* serotype Typhi. *Salmonella* Paratyphi=*Salmonella enterica* serotype Paratyphi. UTI=urinary tract infection. YLLs=years of life lost.

In 2019, there were an estimated 13·7 million (95% UI 10·9–17·1) infection-related deaths globally, with 7·7 million (5·7–10·2) deaths associated with the 33 bacterial pathogens we investigated. These bacteria altogether were associated with 13·6% (10·2–18·1) of all global deaths in 2019 and 56·2% (52·1–60·1) of all infection-related deaths for that year. The all-age mortality rate was 99·6 deaths (74·2–132) per 100 000 population collectively for these pathogens. Only one organism, *Staphylococcus aureus,* was associated with more than 1 million deaths in 2019 (1 105 000 deaths [816 000–1 470 000]; table). Four additional pathogens were associated with more than 500 000 deaths each in 2019; these were *Escherichia coli*, *S pneumoniae, Klebsiella pneumoniae,* and *Pseudomonas aeruginosa* (table, figure 1A). These five leading pathogens were associated with 30·9% (28·6–33·1) of all infection-related deaths and were responsible for 54·9% (52·9–56·9) of all deaths among the investigated bacterial pathogens. Of the bacteria estimated, *Morganella* spp, *Providencia* spp, and *Neisseria gonorrhoeae* had the fewest associated deaths (table). There were 304 million (234–392) YLLs associated with the 33 bacterial pathogens globally in 2019, representing 18·1% (14·1–22·8) of the global YLLs for the year. The leading five organisms by YLL burden were similar to the mortality estimates but the order changed: *S pneumoniae* was associated with the greatest YLL burden with 40·3 million (32·8–50·0) YLLs, followed by *S aureus* with 34·3 million (25·5–45·3), *K pneumoniae* with 31·4 million (23·2–41·5), *E coli* with 30·4 million (22·7–40·2), and *P aeruginosa* with 18·9 million (13·6–25·7; figure 1B; appendix 1 [section 10]).

**Table:** Global number of deaths and age-standardised mortality rate per 100 000 population by bacterial pathogen and infectious syndrome, 2019

	**All 11 infectious syndromes**	**Lower respiratory infections and all related infections of the thorax**	**Meningitis and other bacterial CNS infections**	**Bloodstream infections**	**Skin and subcutaneous bacterial infections**	**Urinary tract infections and pyelonephritis**	**Peritoneal and intra-abdominal infections**	**Bone, joint, and related organ infections**	**Cardiac infections**	**Diarrhoea**	**Typhoid, paratyphoid, and iNTS**	**Chlamydia and gonorrhoea**
Staphylococcus aureus												
All-cause all-age death counts	1 105 000 (816 000–1 470 000)	532 000 (440 000–648 000)	18 400 (13 400–26 000)	299 000 (166 000–485 000)	37 500 (15 700–78 400)	21 300 (15 100–30 800)	169 000 (105 000–253 000)	9490 (2910–21 600)	19 000 (13 200–26 500)	..	..	..
All-cause age-standardised mortality rate	14·6 (10·8–19·4)	7·3 (6·0–8·8)	0·2 (0·2–0·4)	3·9 (2·1–6·3)	0·5 (0·2–1·0)	0·3 (0·2–0·4)	2·1 (1·3–3·2)	0·1 (0·0–0·3)	0·3 (0·2–0·3)	..	..	..
Escherichia coli												
All-age death counts	950 000 (685 000–1 290 000)	181 000 (142 000–230 000)	23 000 (16 200–33 200)	242 000 (133 000–398 000)	18 000 (6190–40 900)	120 000 (96 400–154 000)	290 000 (188 000–423 000)	3370 (1030–7770)	17 700 (12 000–24 500)	54 100 (27 500–95 100)	..	..
Age-standardised mortality rate	12·6 (9·1–16·9)	2·6 (2·0–3·2)	0·3 (0·2–0·5)	3·2 (1·7–5·2)	0·2 (0·1–0·5)	1·6 (1·3–2·0)	3·7 (2·4–5·4)	0·0 (0·0–0·1)	0·2 (0·2–0·3)	0·8 (0·4–1·3)	..	..
Streptococcus pneumoniae												
All-age death counts	829 000 (682 000–1 010 000)	653 000 (553 000–777 000)	44 500 (34 700–59 800)	125 000 (72 600–199 000)	..	..	..	..	6070 (4430–8470)	..	..	..
Age-standardised mortality rate	11·4 (9·4–13·9)	9·1 (7·7–10·8)	0·6 (0·5–0·8)	1·6 (1·0–2·6)	..	..	..	..	0·1 (0·1–0·1)	..	..	..
Klebsiella pneumoniae												
All-age death counts	790 000 (571 000–1 060 000)	276 000 (220 000–343 000)	33 400 (23 600–47 000)	265 000 (157 000–416 000)	7000 (1070–25 800)	38 700 (26 900–55 800)	158 000 (103 000–234 000)	1370 (389–3200)	11 200 (8090–15 400)	..	..	..
Age-standardised mortality rate	10·6 (7·7–14·2)	3·8 (3·1–4·8)	0·5 (0·3–0·7)	3·5 (2·1–5·5)	0·1 (0·0–0·3)	0·5 (0·4–0·7)	2·0 (1·3–2·9)	0·0 (0·0–0·0)	0·1 (0·1–0·2)	..	..	..
Pseudomonas aeruginosa												
All-age death counts	559 000 (390 000–769 000)	233 000 (181 000–302 000)	..	163 000 (94 200–255 000)	22 400 (7320–54 000)	29 900 (17 200–49 600)	103 000 (65 700–151 000)	1360 (368–3330)	7070 (5160–9840)	..	..	..
Age-standardised mortality rate	7·4 (5·2–10·2)	3·2 (2·5–4·1)	..	2·1 (1·2–3·3)	0·3 (0·1–0·7)	0·4 (0·2–0·6)	1·3 (0·8–1·9)	0·0 (0·0–0·0)	0·1 (0·1–0·1)	..	..	..
Acinetobacter baumannii												
All-age death counts	452 000 (269 000–693 000)	166 000 (91 800–267 000)	..	247 000 (138 000–405 000)	12 000 (671–52 900)	16 200 (6350–31 100)	..	..	10 700 (7570–15 200)	..	..	..
Age-standardised mortality rate	5·8 (3·5–8·9)	2·2 (1·2–3·5)	..	3·2 (1·8–5·2)	0·2 (0·0–0·7)	0·2 (0·1–0·4)	..	..	0·1 (0·1–0·2)	..	..	..
Enterobacter **spp**												
All-age death counts	324 000 (211 000–468 000)	64 700 (46 400–89 600)	..	156 000 (90 400–243 000)	7780 (2380–18 600)	13 100 (8350–20 300)	75 500 (47 200–115 000)	1680 (490–3710)	5460 (3770–7980)	..	..	..
Age-standardised mortality rate	4·2 (2·8–6·1)	0·9 (0·6–1·2)	..	2·0 (1·2–3·2)	0·1 (0·0–0·2)	0·2 (0·1–0·3)	0·9 (0·6–1·4)	0·0 (0·0–0·0)	0·1 (0·0–0·1)	..	..	..
**Group B** Streptococcus												
All-age death counts	320 000 (235 000–420 000)	182 000 (140 000–234 000)	19 800 (14 800–27 200)	75 900 (43 900–119 000)	26 500 (6620–70 100)	8870 (6680–12 000)	..	2970 (668–8250)	3940 (2790–5450)	..	..	..
Age-standardised mortality rate	4·4 (3·3–5·8)	2·6 (2·0–3·4)	0·3 (0·2–0·4)	1·0 (0·6–1·6)	0·3 (0·1–0·9)	0·1 (0·1–0·2)	..	0·0 (0·0–0·1)	0·1 (0·0–0·1)	..	..	..
Enterococcus faecalis												
All-age death counts	220 000 (135 000–332 000)	..	..	74 600 (43 900–118 000)	7460 (549–34 300)	19 700 (16 100–23 700)	113 000 (60 500–186 000)	2010 (589–4760)	3380 (2390–4710)	..	..	..
Age-standardised mortality rate	2·8 (1·7–4·3)	..	..	1·0 (0·6–1·6)	0·1 (0·0–0·4)	0·3 (0·2–0·3)	1·4 (0·7–2·3)	0·0 (0·0–0·1)	0·0 (0·0–0·1)	..	..	..
Enterococcus faecium												
All-age death counts	219 000 (134 000–333 000)	..	..	78 200 (44 200–126 000)	..	17 800 (9390–30 600)	118 000 (72 000–185 000)	647 (185–1530)	4480 (3150–6250)	..	..	..
Age-standardised mortality rate	2·8 (1·7–4·2)	..	..	1·0 (0·6–1·6)	..	0·2 (0·1–0·4)	1·5 (0·9–2·3)	0·0 (0·0–0·0)	0·1 (0·0–0·1)	..	..	..
**Non-typhoidal** Salmonella												
All-age death counts	215 000 (135 000–327 000)	..	..	87 100 (53 800–131 000)	..	..	..	..	2430 (1800–3320)	46 300 (3130–139 000)	79 100 (43 000–124 000)	..
Age-standardised mortality rate	3·0 (1·9–4·6)	..	..	1·2 (0·7–1·8)	..	..	..	..	0·0 (0·0–0·0)	0·7 (0·0–1·9)	1·1 (0·6–1·8)	..
**Group A** Streptococcus												
All-age death counts	198 000 (108 000–360 000)	..	..	56 400 (35 000–85 600)	134 000 (53 400–281 000)	..	..	5770 (1740–13 200)	2280 (1680–3150)	..	..	..
Age-standardised mortality rate	2·6 (1·4–4·7)	..	..	0·8 (0·5–1·2)	1·7 (0·7–3·6)	..	..	0·1 (0·0–0·2)	0·0 (0·0–0·0)	..	..	..
Salmonella **Typhi**												
All-age death counts	182 000 (118 000–271 000)	..	..	70 500 (44 600–105 000)	..	..	..	..	1330 (973–1810)	..	110 000 (52 800–191 000)	..
Age-standardised mortality rate	2·6 (1·7–3·8)	..	..	1·0 (0·6–1·5)	..	..	..	..	0·0 (0·0–0·0)	..	1·5 (0·7–2·7)	..
Neisseria meningitidis												
All-age death counts	141 000 (96 800–203 000)	..	31 100 (24 200–41 700)	110 000 (67 600–168 000)	..	..	..	..	..	..	..	..
Age-standardised mortality rate	2·0 (1·3–2·8)	..	0·4 (0·3–0·6)	1·5 (1·0–2·3)	..	..	..	..	..	..	..	..
Campylobacter **spp**												
All-age death counts	123 000 (39 300–266 000)	..	..	..	..	..	..	..	..	123 000 (39 300–266 000)	..	..
Age-standardised mortality rate	1·7 (0·6–3·7)	..	..	..	..	..	..	..	..	1·7 (0·6–3·7)	..	..
Shigella **spp**												
All-age death counts	113 000 (49 800–214 000)	..	..	..	..	..	..	..	..	113 000 (49 800–214 000)	..	..
Age-standardised mortality rate	1·6 (0·7–3·0)	..	..	..	..	..	..	..	..	1·6 (0·7–3·0)	..	..
Proteus **spp**												
All-age death counts	109 000 (72 200–157 000)	..	..	37 600 (21 100–61 900)	9770 (2990–23 600)	23 500 (17 900–31 700)	36 400 (21 700–57 200)	..	1990 (1410–2800)	..	..	..
Age-standardised mortality rate	1·4 (0·9–2·0)	..	..	0·5 (0·3–0·8)	0·1 (0·0–0·3)	0·3 (0·2–0·4)	0·5 (0·3–0·7)	..	0·0 (0·0–0·0)	..	..	..
**Haemophilus influenzae**												
All-age death counts	101 000 (82 800–124 000)	91 300 (74 700–112 000)	9700 (7080–13 500)	..	..	..	..	..	..	..	..	..
Age-standardised mortality rate	1·4 (1·2–1·7)	1·3 (1·0–1·6)	0·1 (0·1–0·2)	..	..	..	..	..	..	..	..	..
Serratia **spp**												
All-age death counts	100 000 (62 100–154 000)	..	..	76 700 (46 300–123 000)	..	4150 (2460–6730)	17 000 (10 200–26 400)	..	2540 (1830–3510)	..	..	..
Age-standardised mortality rate	1·3 (0·8–2·0)	..	..	1·0 (0·6–1·6)	..	0·1 (0·0–0·1)	0·2 (0·1–0·3)	..	0·0 (0·0–0·0)	..	..	..
**Other enterococci**												
All-age death counts	100 000 (65 800–145 000)	..	..	57 000 (32 600–91 000)	14 000 (4560–32 500)	26 600 (19 300–36 900)	..	..	2460 (1670–3440)	..	..	..
Age-standardised mortality rate	1·3 (0·9–1·9)	..	..	0·8 (0·4–1·2)	0·2 (0·1–0·4)	0·4 (0·3–0·5)	..	..	0·0 (0·0–0·0)	..	..	..
Vibrio cholerae												
All-age death counts	96 400 (52 700–159 000)	..	..	..	..	..	..	..	..	96 400 (52 700–159 000)	..	..
Age-standardised mortality rate	1·3 (0·7–2·2)	..	..	..	..	..	..	..	..	1·3 (0·7–2·2)	..	..
Chlamydia **spp**												
All-age death counts	95 300 (74 300–122 000)	94 300 (73 200–121 000)	..	..	..	..	..	..	..	..	..	972 (757–1110)
Age-standardised mortality rate	1·3 (1·0–1·7)	1·3 (1·0–1·7)	..	..	..	..	..	..	..	..	..	0·0 (0·0–0·0)
Mycoplasma **spp**												
All-age death counts	89 400 (74 400–108 000)	89 400 (74 400–108 000)	..	..	..	..	..	..	..	..	..	..
Age-standardised mortality rate	1·2 (1·0–1·5)	1·2 (1·0–1·5)	..	..	..	..	..	..	..	..	..	..
Legionella **spp**												
All-age death counts	56 400 (44 200–74 400)	56 400 (44 200–74 400)	..	..	..	..	..	..	..	..	..	..
Age-standardised mortality rate	0·8 (0·6–1·0)	0·8 (0·6–1·0)	..	..	..	..	..	..	..	..	..	..
Citrobacter **spp**												
All-age death counts	54 100 (33 200–80 400)	..	..	32 600 (18 000–51 700)	..	5210 (3580–7530)	16 300 (9590–25 300)	..	..	..	..	..
Age-standardised mortality rate	0·7 (0·4–1·0)	..	..	0·4 (0·2–0·7)	..	0·1 (0·0–0·1)	0·2 (0·1–0·3)	..	..	..	..	..
**Other *Klebsiella* species**												
All-age death counts	53 900 (28 600–92 700)	..	..	..	..	..	53 900 (28 600–92 700)	..	..	..	..	..
Age-standardised mortality rate	0·7 (0·4–1·2)	..	..	..	..	..	0·7 (0·4–1·2)	..	..	..	..	..
Clostridioides difficile												
All-age death counts	33 200 (25 300–44 900)	..	..	..	..	..	..	..	..	33 200 (25 300–44 900)	..	..
Age-standardised mortality rate	0·4 (0·3–0·6)	..	..	..	..	..	..	..	..	0·4 (0·3–0·6)	..	..
Salmonella **Paratyphi**												
All-age death counts	23 300 (9810–45 700)	..	..	..	..	..	..	..	..	..	23 300 (9810–45 700)	..
Age-standardised mortality rate	0·3 (0·1–0·6)	..	..	..	..	..	..	..	..	..	0·3 (0·1–0·6)	..
Aeromonas **spp**												
All-age death counts	21 300 (9920–38 100)	..	..	..	..	..	..	..	..	21 300 (9920–38 100)	..	..
Age-standardised mortality rate	0·3 (0·1–0·6)	..	..	..	..	..	..	..	..	0·3 (0·1–0·6)	..	..
Listeria monocytogenes												
All-age death counts	14 900 (10 100–21 600)	..	14 900 (10 100–21 600)	..	..	..	..	..	..	..	..	..
Age-standardised mortality rate	0·2 (0·1–0·3)	..	0·2 (0·1–0·3)	..	..	..	..	..	..	..	..	..
Morganella **spp**												
All-age death counts	5510 (3600–8200)	..	..	..	..	5510 (3600–8200)	..	..	..	..	..	..
Age-standardised mortality rate	0·1 (0·0–0·1)	..	..	..	..	0·1 (0·0–0·1)	..	..	..	..	..	..
Providencia **spp**												
All-age death counts	5030 (3110–7720)	..	..	..	..	5030 (3110–7720)	..	..	..	..	..	..
Age-standardised mortality rate	0·1 (0·0–0·1)	..	..	..	..	0·1 (0·0–0·1)	..	..	..	..	..	..
Neisseria gonorrhoeae												
All-age death counts	2960 (2320–3360)	..	..	..	..	..	..	..	..	..	..	2960 (2320–3360)
Age-standardised mortality rate	0·0 (0·0–0·0)	..	..	..	..	..	..	..	..	..	..	0·0 (0·0–0·0)

95% uncertainty intervals are shown in parentheses. Death counts are shown to three significant figures and age-standardised mortality rates are shown to one decimal place. iNTS=invasive non-typhoidal *Salmonella. Salmonella* Typhi=*Salmonella enterica* serotype Typhi. *Salmonella* Paratyphi=*Salmonella enterica* serotype Paratyphi.

**Figure 2:**
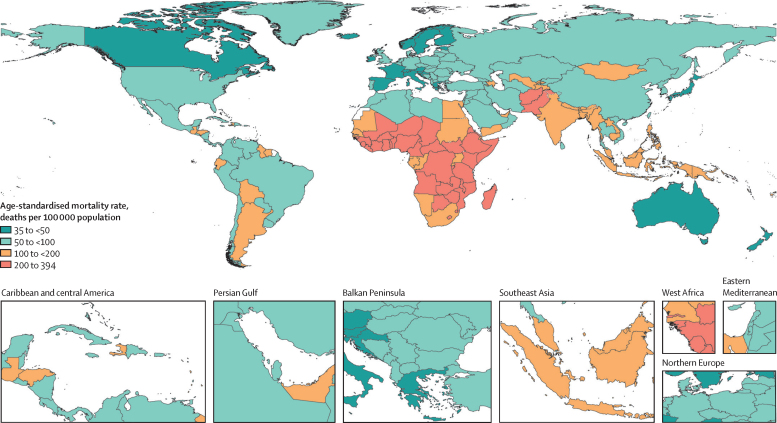
Overall age-standardised mortality rate per 100 000 population for 33 pathogens investigated, 2019

**Figure 3:**
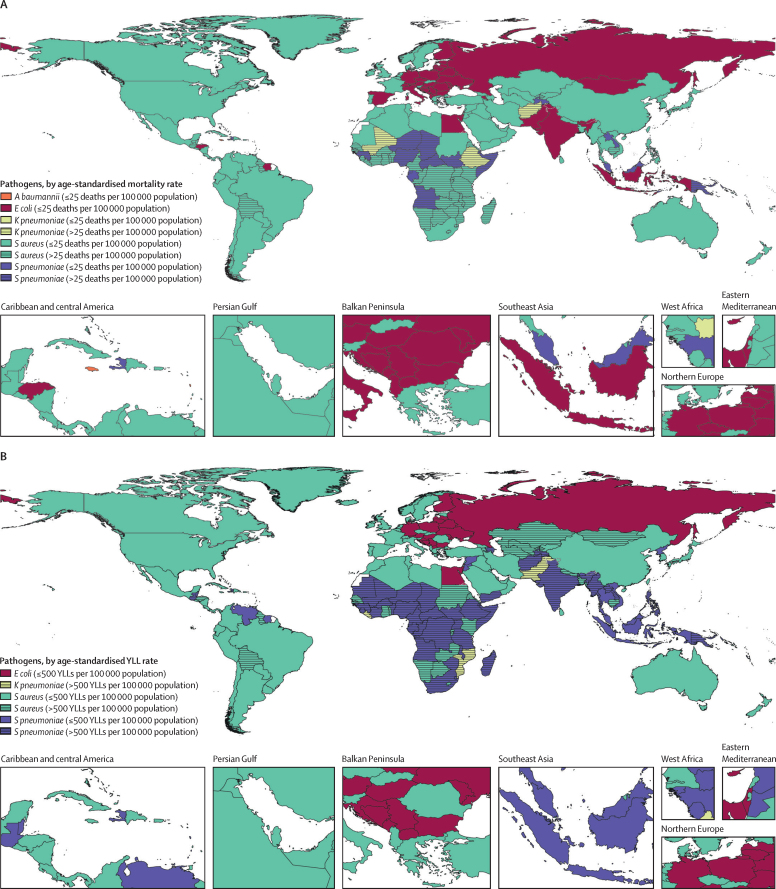
Pathogen responsible for the highest age-standardised mortality rate per 100 000 population (A) and for the highest age-standardised YLL rate per 100 000 population (B), for each country or territory, 2019 *A baumannii*=*Acinetobacter baumannii. E coli=Escherichia coli. K pneumoniae=Klebsiella pneumoniae. S aureus*=*Staphylococcus aureus. S pneumoniae*=*Streptococcus pneumoniae.* YLLs=years of life lost.

**Figure 4:**
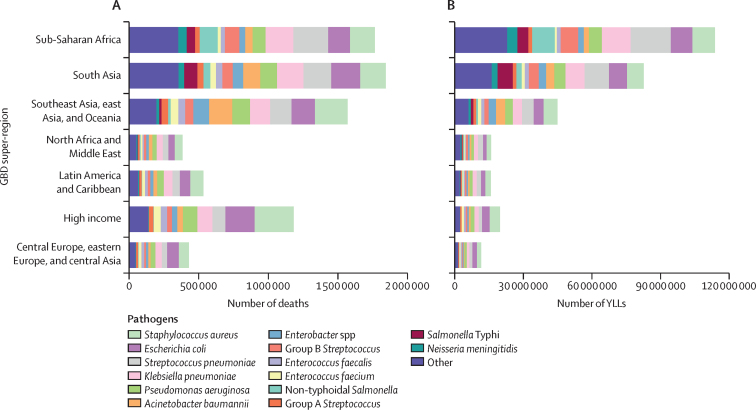
Global number of deaths (A) and YLLs (B), by pathogen and GBD super-region, 2019 Data are presented for the 14 pathogens with the largest number of global deaths; the Other group comprises the additional 19 bacteria estimated in this study. GBD=Global Burden of Diseases, Injuries, and Risk Factors. *Salmonella* Typhi=*Salmonella enterica* serotype Typhi. YLLs=years of life lost.

The age-standardised mortality rate associated with these 33 bacterial pathogens varied by super-region in 2019 but was highest in sub-Saharan Africa, at 230 deaths (95% UI 185–285) per 100 000 population, and lowest in the high-income super-region, at 52·2 deaths (37·4–71·5) per 100 000 population. Central African Republic was the country with the highest age-standardised mortality rate associated with these 33 bacterial pathogens, with 394 deaths (297–518) per 100 000 population, while Iceland had the lowest rate, with 35·7 deaths (25·1–49·3) per 100 000 population in 2019 (figure 2; appendix 1 [section 10]). The pathogens linked to the most deaths varied across locations. *S aureus* was the leading bacterial cause of death in 135 countries, followed by *E coli* (leading cause in 37 countries), *S pneumoniae* (leading cause in 24 countries), and *K Pneumoniae* and *Acinetobacter baumannii* (leading causes in four countries each; figure 3A; appendix 2). *S aureus, E coli, K pneumoniae,* and *S pneumoniae* were among the five leading pathogens associated with the greatest death count and the greatest YLL burden in every super-region (figure 4). *S aureus* was also the pathogen with the highest age-standardised mortality rate in 16 of 21 GBD regions, *S pneumoniae* had the highest rate in three regions (Oceania, South Asia, and western sub-Saharan Africa), and *E coli* had the highest rate in South Asia and central and eastern Europe (appendix 1 [section 10]). The pathogens associated with the greatest age-standardised YLL burden varied across locations (figure 3B). *S aureus* was the leading pathogen in 111 countries, followed by *S pneumoniae* in 69 countries, and *E coli* in 20 countries (appendix 2).

**Figure 5:**
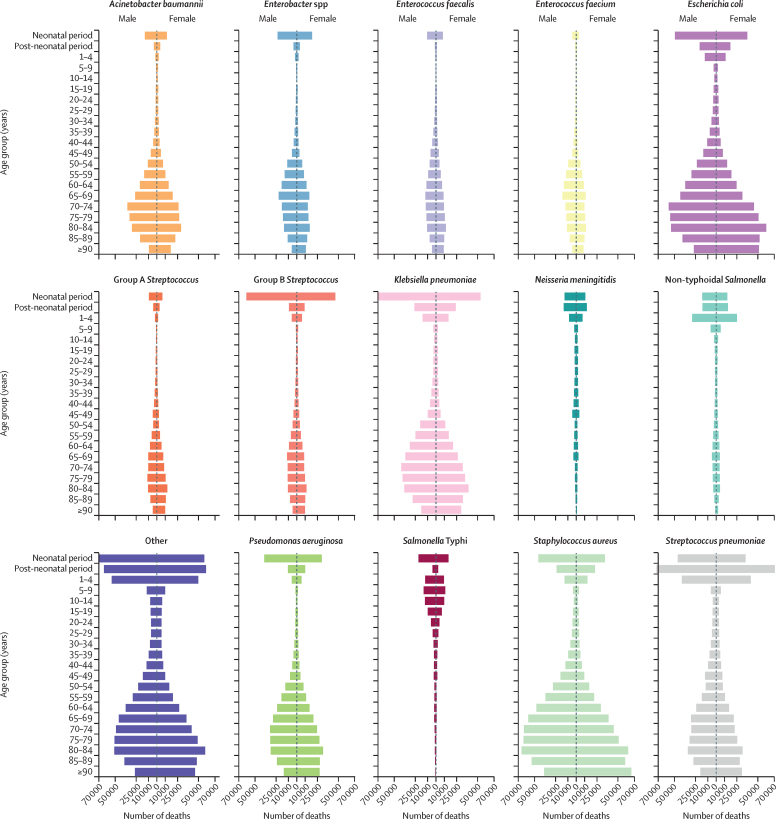
Global number of deaths, by pathogen, age, and sex groups, 2019 Data are presented for the 14 pathogens with the largest number of global deaths; the Other group comprises the additional 19 bacteria estimated in this study. Neonatal=0 days to 27 days old. Post-neonatal=28 days to <1 year old. *Salmonella* Typhi=*Salmonella enterica* serotype Typhi.

The pathogen associated with the most deaths differed by age. Globally, *S aureus* was the pathogen associated with the most deaths in individuals older than 15 years, with 940 000 deaths (95% UI 682 000–1 276 000) in that age group. *Salmonella enterica* serovar Typhi was associated with the most deaths in children aged 5–14 years (49 000 deaths [23 000–86 000]). *S pneumoniae* was associated with the most deaths among young children post-neonatal to age 4 years (225 000 [180 000–281 000]), whereas *K pneumoniae* was the pathogen associated with the most neonatal deaths (124 000 [89 000–167 000]). We found no differences between males and females in the ranking of deaths associated with the leading six bacteria (*S aureus*, *E coli*, *S pneumoniae*, *K pneumoniae*, *P aeruginosa*, and *A baumannii*). The absolute number of deaths associated with these pathogens was always smaller for females than for males, except among those aged 80 years and older, for whom the number of deaths in females exceeded those in males (figure 5; appendix 1 [section 10]). *S aureus* was estimated to have the largest number of deaths for both males (601 000 deaths [442 000–807 000]) and females (504 000 deaths [371 000–669 000]), and was closely followed by *E coli*, for which the difference in the number of deaths between females (450 000 deaths [329 000–602 000]) and males (500 000 deaths [355 000–684 000]) was smaller (figure 5; appendix 1 [section 10]).

Two infectious syndromes were responsible for more than 2 million deaths each in 2019: lower respiratory infections with 4·00 million (95% UI 3·33–4·89) deaths, and bloodstream infections with 2·91 million (1·74–4·53) deaths. Peritoneal and intra-abdominal infections were responsible for 1·28 million (0·826–1·86) deaths. The syndrome responsible for the most deaths due to bacterial infection varied across locations, while the three leading syndromes were consistently lower respiratory infections, bloodstream infections, and peritoneal and intra-abdominal infections. There was variation in terms of which of these three syndromes caused the most deaths across GBD super-regions, with lower respiratory infections being the leading syndrome in five super-regions and bloodstream infections being the leading syndrome in two super-regions (appendix 1 [section 10]).

There was substantial variation in which pathogen was the most dominant across different infectious syndromes, with *S pneumoniae* being the leading cause of fatal lower respiratory infections with 653 000 deaths (95% CI 553 000–777 000), *S aureus* being the leading cause of fatal bloodstream infections with 299 000 deaths (166 000–485 000), and *E coli* being the leading cause of fatal peritoneal and intra-abdominal infections with 290 000 deaths (188 000–423 000; table). Similarly, the most prevalent infectious syndrome varied across pathogens, with 78·9% (73·3–83·3) of deaths due to *S pneumoniae* occurring by way of lower respiratory infections, whereas *E coli* caused a wider range of syndromes, with 30·4% (26·1–35·5) of all *E coli*-associated deaths occurring via peritoneal and intra-abdominal infections, followed by 25·1% (18·7–32·1) occurring via bloodstream infection (figure 1). For each of these three infectious syndromes, the distribution of the responsible pathogen varied across locations (appendix 1 pp 67–68). The greatest differences by location were seen in the role of *S aureus* in bloodstream infection, with the pathogen being associated with 23% of deaths due to bloodstream infections caused by any bacteria in the high-income super-region in 2019 (appendix 1 p 72), but only 5% of deaths due to bloodstream infections caused by any bacteria in sub-Saharan Africa, where *K pneumoniae* caused the most deaths due to bloodstream infections, followed by *N meningitidis*.

## Discussion

To our knowledge, this is the first study to provide global estimates on mortality and YLLs for a wide range of bacterial genera and species across 11 major infectious syndromes. We found that, collectively, the 33 analysed bacteria were associated with 7·7 million (95% UI 5·7–10·2) deaths in 2019, with an all-age mortality rate of 99·6 deaths (74·2–132) per 100 000 population. These bacteria were involved in 13·6% (10·1–18·1) of global deaths in 2019 and, compared with Level 3 GBD underlying causes of death, would rank as the second leading cause of death globally, behind ischaemic heart disease.^3^ Individually, four pathogens were associated with more than 750 000 deaths and 30 million YLLs globally in 2019, and their ranks as leading Level 3 causes of death in 2019 would be as follows: *S aureus* would rank as the 15th, *E coli* as the 18th*, S pneumoniae* as the 20th, and *K pneumoniae* as the 21st leading Level 3 cause of death. There was considerable variation in the burden of bacterial infections, with the greatest number of deaths occurring in the sub-Saharan Africa super-region, where we clearly saw the effect of both Gram-positive and Gram-negative pathogens. The disparate burden in sub-Saharan Africa is magnified by the substantial YLL burden associated with these bacteria in this super-region compared with other super-regions.

By estimating mortality and YLLs for a broad range of pathogens and infectious syndromes, we have produced a global account of bacteria for which the burden was previously unknown and, perhaps, underappreciated. More than half of bacterial deaths in our study were caused by one of five pathogens: *S aureus, E coli, S pneumoniae, K pneumoniae,* and *P aeruginosa.* Of these pathogens, only *S pneumoniae* has been the focus of global surveillance and public health initiatives.^19^, ^24^ Although infectious diseases like HIV/AIDS, tuberculosis, and neglected tropical diseases each have their own SDG indicators (eg, SDG 3.3) and have substantial global public health investment (eg, The Global Fund to Fight AIDS, Tuberculosis and Malaria), the bacterial pathogens we found to be associated with a greater fatal burden are not a major focus of any global public health initiatives. Only recently have there been calls to expand the scope of The Global Fund to include more common bacteria, although in the context of antimicrobial resistance.^25^ *S aureus* was the leading bacterial pathogen in most countries and was the only pathogen associated with more than 1 million deaths and 34 million YLLs globally, yet there is no global public health investment directed at *S aureus.* Instead, *S aureus* is included in surgical site infection prevention^26^ and antimicrobial resistance initiatives,^27^ which focus on methicillin-resistant *S aureus* (known as MRSA), despite the fact that strains with such resistance comprise only a subset of the *S aureus* burden. Although WHO prioritised *S aureus* in 2014 as one of the seven bacteria of international concern, this was in the context of antimicrobial resistance and little has been done regarding the susceptible *S aureus* burden.^28^ Similarly, *E coli* and *K pneumoniae* are collectively associated with more deaths and YLLs than *S pneumoniae* or tuberculosis,^29^ yet they receive comparatively little public health attention relative to their burden, and minimal research funding relative to other diseases with a comparable, or lower, burden. A 2020 analysis of global funding for infectious disease research found that HIV research was awarded US$42 billion in funding compared with $1·4 billion for research on *Staphylococcus* spp and $800 million for *E coli* research over the same period (between 2000 and 2017).^30^ The investments in HIV research are certainly warranted and, although bacterial infections could be tackled with different overlapping strategies, this disparity in funding might have been driven, in part, by the shortage of global burden numbers for these bacterial pathogens.

The 33 bacterial agents investigated as part of this study comprise a significant cause of health loss globally, and strategies to address this substantial burden cover a wide range of interventions. First, infection prevention is the foundation to reducing the burden of infections. Infection prevention broadly includes in-hospital programmes aimed at reducing hospital-acquired infection,^31^ and community programmes that focus on health education, management of malnutrition in LMICs, and the core principles of access to clean water, sanitation, and hygiene.^32^, ^33^ Second, vaccination can have a substantial effect on the burden of bacterial infections through a number of routes. Implementation and uptake of vaccines for bacteria like *S pneumoniae* can directly reduce the burden of bacterial infections, and new generations of vaccines will target older age groups that we have found are significantly affected by this bacterial agent.^16^ Beyond this, uptake of vaccination for non-bacterial infections like influenza, where bacterial superinfection is a common complication, can also reduce the burden of bacterial infections.^34^ Additionally, vaccine development is crucial for bacteria for which no vaccine exists, and these estimates could help set vaccine development priorities.^9^ However, issues on how to tackle the bacteria that can be present as commensal microbiota have to be considered. For example, the alteration of commensal bacteria can influence susceptibility to gastrointestinal diseases, which might be an issue when developing a vaccine against *E coli*; different biology and vaccinology approaches hold the promise of resolving this conundrum.^35^ Third, availability of basic acute care services can reduce the number of deaths associated with these bacterial infections. Such services include timely access to appropriate antibiotics, microbiological capacity to identify the responsible pathogen of an infection, and provision of supportive care.^36^, ^37^ Finally, a strategic approach and ample investment in the development of new and effective antibiotics are essential to face the increasing threat posed by bacterial antimicrobial resistance and bacterial infections in general.^38^

Effective antimicrobials exist for all 33 of the investigated bacteria, yet much of the disproportionately high burden in LMICs might be attributable to inadequate access to effective antimicrobials, weak health systems, and insufficient prevention programmes.^39^, ^40^ Many barriers to accessing these effective antimicrobials have been described. First, health-care-seeking behaviours are impeded by high out-of-pocket costs, driven by deficiencies in government funding for health and unaffordable drug prices in LMICs.^41^ Second, unwarranted antibiotic use caused by poor education of health-care providers, regulatory issues, self-medication, and restricted availability of antibiotics can lead to the wrong antimicrobial being prescribed, which, if too broad, can promote resistance and, if ineffective, risks progression of infection.^41^ Third, unstable supply chains and poor quality control can result in the desired antibiotic being unavailable or the dissemination of substandard or counterfeit antimicrobials to the consumer.^42^ Improving access to antibiotics requires a nuanced and location-specific response because ease of access must be weighed against risk of antibiotic overuse (a problem compounded by the issue of self-medication in LMICs),^43^ which contributes to the increase in antimicrobial resistance.^44^ Furthermore, the use of antibiotics in animal husbandry must be taken into account.^44^ In this study, we addressed the overall burden of infections both susceptible and resistant to antimicrobials, but our previous study^19^ highlighted the issue of resistance and its compounding effect on mortality rates. We argue that robust surveillance mechanisms in conjunction with these types of studies will be indispensable to understand the true burden of bacterial infections.

Three syndromes are responsible for more than 75% of the estimated 7·7 million bacteria-related deaths that occurred in 2019. Lower respiratory infections, bloodstream infections, and peritoneal and intra-abdominal infections would rank as the third, seventh, and 13th leading causes of death globally for 2019, respectively, all ahead of other causes such as HIV, colorectal cancer, or self-harm. Lower respiratory infections have long been a global health priority,^45^ and bloodstream infections have arguably been included in the umbrella of more recent global sepsis initiatives;^46^, ^47^ however, intra-abdominal infections and peritonitis do not receive the same attention as other diseases with similar or lower fatal burden. Although overlap exists in the management of peritoneal and intra-abdominal infections with other bacterial infections (eg, antibiotics and identification of the infection's source), management of peritoneal and intra-abdominal infections poses unique challenges in that radiological imaging is often required to establish a source and surgical intervention might be needed to achieve source control.^48^ There is a substantial shortage of medical capacity and trained personnel in many LMICs to address peritoneal and intra-abdominal infections and other infections that require surgical intervention.^49^ Recent estimates suggest 4·8 billion people do not have access to timely surgical services, with low-income countries estimated to have fewer than one provider per 100 000 population.^50^ Compounding inadequate access to surgical services is the restricted availability of diagnostic radiology, with a recent analysis of ten LMICs across the Caribbean, South Asia, and sub-Saharan Africa finding that CT was available in only 6% of hospitals and ultrasound was available in only 50% of hospitals.^51^

The remarkable geographical variation of responsible pathogens for a given infectious syndrome is highlighted by *S aureus* as the causative pathogen of bloodstream infection. In the high-income super-region, *S aureus* caused 23% of deaths due to bloodstream infections that involved one of the 33 bacteria investigated, compared with only 5% of deaths due to bloodstream infections in the sub-Saharan Africa super-region. This variation has profound implications on the empirical management of infections when a responsible pathogen has not yet been identified and breadth of coverage must be balanced against risk of antibiotic resistance. The WHO essential medicines list provides global empirical antibiotic recommendations for various infectious syndromes;^52^ however, our findings suggest that a move towards region-specific empirical antibiotic recommendations might be more appropriate from an antibiotic stewardship and antimicrobial efficacy standpoint.^53^ Region-specific guidance will also help in addressing inappropriate antibiotic use in LMICs, which is one of the key drivers of antimicrobial resistance.^43^ We hope that these estimates might be used to guide empirical antibiotic use, yet data sparsity remains a major limitation in creating more granular estimates with sufficient confidence to inform individual clinicians in accordance with clinical needs and the aim to uphold antimicrobial stewardship.^54^

We should also acknowledge *M tuberculosis*, which was not included in our analysis. One reason we did not do additional estimations for this important pathogen is because the global burden estimates provided by GBD 2019 and WHO are quite concordant and well established.^29^, ^55^ A GBD study has shown that, in 2019, there were 9·65 million incident cases and 1·21 million deaths due to tuberculosis in both HIV-negative and HIV-positive individuals.^29^ There was also a greater incidence and an excess burden in males, which is comparable with the burden of bacterial agents estimated in this study. Geographically, most cases of tuberculosis in 2019 were found in the WHO regions of South-East Asia, Africa, and the Western Pacific,^29^, ^55^ which is comparable with the geographical spread of the burden of the 33 bacteria we investigated in this study.

Insufficient microbiological capacity has substantial effects on both population health estimates and the clinical care of individual patients. Correspondingly, an urgent need exists to build microbiology laboratory networks and develop innovative surveillance strategies.^56^ Identification of a responsible pathogen in sepsis and other severe infections can help inform optimal antibiotic choice and duration, and lead clinicians to probable sources of infection. Without microbiological data, patients might remain on inappropriate or ineffective antibiotics that contribute to worse health outcomes and fuel the spread of antimicrobial resistance. Practical antibiotic prescribing is also affected because the distribution of pathogens and local patterns of antimicrobial susceptibility are unknown, which hamper the development of dependable treatment protocols. In a recent study,^51^ investigators found that fewer than half of hospitals in ten LMICs had the capacity to do Gram staining, and we speculate that even fewer hospitals in this context could perform cultures and susceptibility testing.^51^ Many locations have little or no microbiology data to inform local burden estimates, and so they must rely on modelled estimates to approximate the burden, resulting in wide uncertainty intervals. The barriers to building microbiology capacity in LMICs have been well described,^57^ and overcoming these challenges requires greater investment and prioritisation of bacteriology capacity, and the development of national antimicrobial resistance surveillance networks.

Our study has several limitations, many of which are the result of data sparsity. Input data for each modelling step has incomplete geographical coverage and is of varying quality for many LMICs, and we did not have data for 61 countries or territories for all three of our modelling steps. Hence, the locations where the burden is estimated to be the greatest are where the data are most scarce, which is an issue exacerbated by age-targeted surveillance protocols; this data scarcity should underscore the urgency of improving capacity and surveillance systems in LMICs. The identification of deaths in which infection had a role relied on International Classification of Diseases (ICD) coded deaths, which does not perfectly correlate with expert chart review. Our estimates of lower respiratory infections and urinary tract infections split infections into community-acquired versus hospital-acquired infections on the basis of whether ICD coding indicated the infection was an underlying or intermediate cause of death. However, this approach has not previously been validated and has the risk of misclassification. We assumed the same pathogen distribution among culture-negative as among culture-positive infections. This assumption could overestimate pathogens that are easier to detect and underestimate pathogens that are difficult to culture with the use of standard microbiological techniques (eg, culture-negative endocarditis might be caused by *Bordetella* spp or *Coxiella* spp, bacteria that are notoriously difficult to culture, although we expect the effect of this particular example on overall bacteria aetiologies to be quite small). We have a residual polybacterial category in which multiple possible causative pathogens were identified for a single infection; however, because many of these infections involved one or more of the 33 bacteria we investigated, this approach leads to an underestimation of the specified bacteria. Additionally, passive microbial surveillance data could have had some selection bias, particularly if cultures were not routinely drawn. In some locations, cultures might be drawn only if someone is critically ill or has not responded to treatment, which might overestimate more virulent or more resistant pathogens. Finally, this study is supported by the framework of and estimates from the GBD study, which has its own limitations that have been discussed elsewhere.^3^

The 7·7 million deaths associated with the 33 pathogens we investigated are deaths that occurred in people with infections caused by one of these bacteria; however, we cannot conclusively state that if all infections due to these 33 pathogens were eliminated, then 7·7 million deaths would have been prevented. Many of these deaths were identified as deaths due to sepsis, when the underlying cause was non-infectious. In a subset of these deaths, the underlying cause of death might have been so severe that a death would have occurred whether or not the infection took place. For example, someone with terminal pancreatic cancer who dies from *E coli* peritonitis is counted the same as a neonate who dies of neonatal sepsis due to *E coli*. However, most of the estimated 7·7 million deaths occurred when the infection with one of the 33 bacteria was the underlying cause of death, and in those cases, we could reasonably assume that those deaths would have been prevented if the infection had not occurred. Placing infections into discrete categories of clinical syndromes opens up the discussion of how to address bloodstream infections, a syndrome that is not always distinct from other clinical syndromes and is often an intermediary between a precipitating infection and sepsis. Our approach to infectious syndromes used a hierarchy process in which bloodstream infections were ranked the lowest—ie, if bloodstream infection was reported alongside any other infectious syndrome, the other infectious syndrome was used. In other words, bloodstream infections as reported here were primary bloodstream infections for which the point of entry or other associated infectious syndromes could not be identified.

In summary, our analyses show that bacterial infections are a clinically significant cause of health loss globally. Five pathogens were each involved in more than 500 000 deaths in 2019: *S aureus, E coli, S pneumoniae, K pneumoniae,* and *P aeruginosa*. Three infectious syndromes, each responsible for more than 1 million deaths in 2019, comprised more than 75% of deaths due to bacterial infections. A sobering reality is that a high burden of treatable infections occurred in very young age groups. Building stronger health systems with more robust diagnostic infrastructure, improved diagnostic imaging and microbiological capacity, and standardised workflows are crucial steps to address this substantial burden, together with implementing appropriate infection control and antimicrobial stewardship measures. Essential prevention strategies include improved access to safe drinking water and sanitation facilities, increased rates of vaccination, new vaccine development, and improving access to the appropriate antibiotic for an infection. There is a need to reconcile the right to antimicrobial access with non-judicious use, particularly with regard to expensive and newer generation antimicrobials. Predictive mathematical modelling and further advancements in genomic epidemiology of infections will increase insights at the global level to understand pathogens' evolution, epidemiology, and pathogenesis, and will better inform future approaches.

## Data sharing

Citations for the data used in the study can be accessed from the Global Health Data Exchange website. Access to the data can also be provided as data use agreements permit.

## Declaration of interests

S Afzal reports support for the present manuscript from Department of Community Medicine and Epidemiology, King Edward Medical University; participation on a data and safety monitoring board or advisory board with Corona Expert Advisory Group and Dengue Expert Advisory Group; leadership or fiduciary roles in board, society, committee, or advocacy groups, unpaid with Pakistan Society of Community Medicine & Public Health, Pakistan Association of Medical Editors, and Pakistan Society of Medical Infectious Diseases. S Bhaskar reports support for the present manuscript from leadership or fiduciary roles in board, society, committee or advocacy groups, paid or unpaid with the Rotary Club of Sydney and Global Health and Migration Hub Community, Global Health Hub Germany as a Board Director and Co-Manager. X Dai reports support for the present manuscript from the Institute for Health Metrics and Evaluation (IHME) and University of Washington. S Das reports grants or contracts from The Department of Science & Technology (DST) Grant of 16 Lakh INR by the Government of India for COVID-19 Research; leadership or fiduciary roles in board, society, committee or advocacy groups, paid or unpaid with Program Chair The American Association for Clinical Chemistry (AACC) India Section as an executive member in Ambi, India. S J Dunachie reports support for the present manuscript from UK National Institute of Health and Care Research (NIHR), funded by an NIHR Global Research Professorship (NIHR300791); grants or contracts from UK Research and Innovation (UKRI; MR/W02067X/1 and MR/W020653/1), Wellcome Drug Resistant Infections Discretionary Award, UK Department of Health and Social Care, and US Defense Threat Reduction Agency; and consulting fees from the Scottish Parliament and funding committees for Wellcome. C Herteliu reports grants or contracts from Romanian Ministry of Research Innovation and Digitalization, MCID and Romanian National Authority for Scientific Research and Innovation, and CNDS-UEFISCDI. N E Ismail reports leadership or fiduciary roles in board, society, committee, or advocacy groups, unpaid with Malaysian Academy of Pharmacy as a Council Member. J J Jozwiak reports payment or honoraria for lectures, presentations, speakers bureaus, manuscript writing or educational events from Teva, Amgen, Synexus, Boehringer Ingelheim, ALAB Laboratories, and Zentiva. N J Kassebaum reports research support for the GBD Study and the present manuscript from The Bill & Melinda Gates Foundation. F Krapp Lopez reports support for the present manuscript from University of Oxford, and financial support provided to Universidad Peruana Cayetano Heredia for data extraction and preparation for the present manuscript. K Krishan reports other non-financial support from UGC Centre of Advanced Study, CAS II, Department of Anthropology, Panjab University, Chandigarh, India. A-F A Mentis reports grants or contracts from MilkSafe (A novel pipeline to enrich formula milk using omics technologies), a research co-financed by the European Regional Development Fund of the EU and Greek national funds through the Operational Program Competitiveness, Entrepreneurship and Innovation, under the call RESEARCH - CREATE - INNOVATE (project code: T2EDK-02222), as well as from ELIDEK (Hellenic Foundation for Research and Innovation, MIMS-860); stocks in a family winery; other financial or non-financial support from the BGI group as a scientific officer. N Milevska Kostova reports grants or contracts from BD Europe and Pfizer (institutional grant for patient education); payment or honoraria for lectures or educational events from Pfizer; and support for attending meetings or travel from Pfizer. S Mohammed reports support for the present manuscript from The Bill & Melinda Gates Foundation and grants or contracts from Alexander von Humboldt Foundation. L Monasta reports support for the present manuscript from the Italian Ministry of Health on project Ricerca Corrente (34/2017) and payments made to the Institute for Maternal and Child Health IRCCS Burlo Garofolo. C E Moore reports support for the present manuscript from the UK Department of Health and Social Care, Wellcome Trust, and The Bill & Melinda Gates Foundation. S B Munro reports stock or stock options in Invitae as an employee. V C F Pepito reports grants from Sanofi Consumer Healthcare and International Initiative for Impact Evaluation. M J Postma reports leadership or fiduciary roles in board, society, committee, or advocacy groups, paid or unpaid, with the Joint Committee of Vaccination and Immunization as a member and stock or stock options in PAG BV (Groningen, Netherlands) and HealthEcore (Zeist, Netherlands). A Radfar reports other support from Avicenna Medical and Clinical Research Institute. K E Rudd reports support for the present manuscript from the National Institutes of Health, National Institute of General Medical Sciences (grant K23GM141463), and consulting fees from Janssen Pharmaceuticals. B Sartorius reports grants or contracts from the Fleming Fund; leadership or fiduciary roles in board, society, committee, or advocacy groups, paid or unpaid, with WHO Reference Group on Health Statistics and GBD Scientific Council. S Shrestha reports other financial or non-financial support from the Graduate Research Merit Scholarship from the School of Pharmacy, Monash University Malaysia. L M L R Silva reports grants or contracts from project code CENTRO-04-3559-FSE-000162, Fundo Social Europeu (FSE). J A Singh reports consulting fees from Crealta/Horizon, Medisys, Fidia, PK Med, Two Labs, Adept Field Solutions, Clinical Care options, Clearview healthcare partners, Putnam associates, Focus forward, Navigant consulting, Spherix, MedIQ, Jupiter Life Science, UBM, Trio Health, Medscape, WebMD, Practice Point communications, the National Institutes of Health, and the American College of Rheumatology; payment or honoraria for lectures, presentations, speakers bureaus, manuscript writing, or educational events from Simply Speaking; support for attending meetings or travel from the steering committee of OMERACT; participation on a data safety monitoring board or advisory board with the US Food and Drug Administration Arthritis Advisory Committee; leadership or fiduciary role in board, society, committee or advocacy group, paid or unpaid, with OMERACT as a steering committee member, as Chair of the Veterans Affairs Rheumatology Field Advisory Committee, and as Editor and Director of the UAB Cochrane Musculoskeletal Group Satellite Center on Network Meta-analysis; stock or stock options in TPT Global Tech, Vaxart Pharmaceuticals, Atyu Biopharma, Adaptimmune Therapeutics, GeoVax Labs, Pieris Pharmaceuticals, Enzolytics, Seres Therapeutics, Tonix Pharmaceuticals, and Charlotte's Web Holdings; and previously owning stock options in Amarin, Viking, and Moderna pharmaceuticals. E Upadhyay reports patents published: “A system and method of reusable filters for anti-pollution mask” (patent application number 202011003559), and “A system and method for electricity generation through crop stubble by using microbial fuel cells” (patent application number 202011008531), patents filed: “A system for disposed personal protection equipment (PPE) into biofuel through pyrolysis and method” (patent application number 202111005659) and “A novel herbal pharmaceutical aid for formulation of gel and method thereof” (patent application number 202111023335); and leadership or fiduciary roles in board, society, committee, or advocacy groups, paid or unpaid, with the Indian Meteorological Society, Jaipur Chapter (India) as a joint secretary and life member. H R van Doorn reports participation on a data and safety monitoring board or advisory board with Wellcome SEDRIC (Surveillance and Epidemiology of Drug Resistant Infections).
