# Supplementary material for: Global mortality associated with 33 bacterial pathogens in 2019: a systematic analysis for the Global Burden of Disease Study 2019
Source: Lancet. 2022 Dec 17;400(10369):2221–48. doi: 10.1016/S0140-6736(22)02185-7 (PMC9763654; doi:10.1016/S0140-6736(22)02185-7)
Supplement: Supplementary appendix 1 [file mmc1.pdf]

THE LANCET

Supplementary appendix 1

This appendix formed part of the original submission and has been peer reviewed. We post it as supplied by the authors.

Supplement to: GBD 2019 Antimicrobial Resistance Collaborators. Global mortality associated with 33 bacterial pathogens in 2019: a systematic analysis for the Global Burden of Disease Study 2019. *Lancet* 2022; published online Nov 21. https://doi.org/10.1016/S0140-6736(22)02185-7.

Appendix 1: supplementary methods to “Global mortality associated with 33 bacterial pathogens in 2019: a systematic analysis for the Global Burden of Disease Study 2019”

This appendix provides further methodological details for “Global mortality associated with 33 bacterial pathogens in 2019: a systematic analysis for the Global Burden of Disease Study 2019”. Parts of this appendix are taken directly from the appendix of the paper “Global burden of bacterial antimicrobial resistance in 2019: a systematic analysis“,[1] which is also referenced throughout the text.

Table of contents

Section 1: List of abbreviations 5

Section 2: Data sources 6

- Section 2.1: Multiple causes of death and vital registration (MCoD-VR) 7
- Section 2.2: Hospital discharge 7
- Section 2.3: Microbial data with outcome 7
- Section 2.4: Microbial data without outcome 9
- Section 2.5: Literature review of the microbial aetiology of meningitis, maternal and neonatal sepsis, lower respiratory infections, urinary tract infections, skin infections, peritonitis, and bone and joint infections 10
  - Section 2.5.1: Meningitis infection aetiology 10
  - Section 2.5.2: Maternal sepsis 10
  - Section 2.5.3: Neonatal sepsis 10
  - Section 2.5.4: LRI aetiology 10
  - Section 2.5.5: Urinary tract infections aetiology 11
  - Section 2.5.6: Skin infections aetiology 11
  - Section 2.5.7: Intra-abdmoinal infection aetiology 11
  - Section 2.5.8: Bone and joint infections aetiology 11
  - Section 2.5.9: Exclusion criteria for literature reviews of microbial aetiologies 11
- Section 2.6: Mortality surveillance 12
- Section 2.7: Linkage (mortality only) 12

Section 3: Summary of GBD 2019 estimation process 12

- Section 3.1: GBD 2019 cause of death estimation process 12
- Section 3.2: GBD 2019 non-fatal estimation process 13

Section 4: Deaths where infection plays a role and infectious syndrome estimation 13

- Section 4.1: Input data 13
  - Section 4.1.1: Multiple causes of death 13
  - Section 4.1.2: Hospital record with multiple diagnoses and discharge status of death 13
  - Section 4.1.3: Linkage data 13
  - Section 4.1.4: Mortality surveillance (Child Health and Mortality Prevention Surveillance [CHAMPS]) 14
- Section 4.2: Data processing 15
- Section 4.3: Mapping the data 15
- Section 4.4: Intermediate cause and infectious syndrome mapping hierarchy 15
  - Section 4.4.1: Intermediate cause mapping 15
  - Section 4.4.2: Informative ranking 17

Section 4.4.3: Two modelling pathways 17
Section 4.5: First pathway: deaths where infection plays a role 17
Section 4.5.1: Sepsis model 17
Section 4.6: Second pathway: fraction of deaths where infection plays a role by infectious syndrome in each GBD cause 20
Section 4.6.1: Aggregation to the sepsis mortality envelope 22
Section 4.6.2: Infectious syndromes using GBD 2019 results 22
Section 4.7: Model validation 23
Section 5: Case fatality ratios 27
Section 5.1: Input data 27
Section 5.2: Data Processing 28
Section 5.3: Modelling overview 28
Section 5.4 Modelling framework 29
Section 5.5 Predictions and uncertainty 31
Section 6: Pathogen distribution 32
Section 6.1: Input data 32
Section 6.2: Data processing 32
Section 6.2.1: Extraction and standardisation 32
Section 6.2.2: Assigning infectious syndrome 33
Section 6.2.3: Contaminants and no aetiology detected 34
Section 6.2.4: Polymicrobial infections 34
Section 6.2.5: Selecting pathogens for estimation 35
Section 6.2.6: Estimating unbiased other and polymicrobial categories 35
Section 6.2.7: Age-sex splitting 37
Section 6.2.8: Standardising measures 37
Section 6.3: Modelling framework 37
Section 6.3.1: Overview 37
Section 6.4: Exceptions and special handling 41
Section 6.4.1: Cardiac infections 41
Section 6.4.2: Diarrhoea 41
Section 6.4.3: Bacterial infections of the skin and subcutaneous systems 41
Section 6.4.4: Lower respiratory infections and all related infections in the thorax 42
Section 6.4.5: Peritoneal and intra-abdominal infections 42
Section 6.4.6: Meningitis and other bacterial central nervous system infections 42
Section 6.4.7: Infectious syndromes not modelled 42
Section 6.5: Model validation 42

Section 7: PRISMA Compliance: Preferred Reporting Items for Systematic Reviews and Meta-Analyses 43
PRISMA 2020 Checklist 43
PRISMA 2020 for Abstracts Checklist 49
PRISMA Diagrams 51
PRISMA 2020 flow diagrams for new systematic reviews which included searches of databases and registers only 51
Section 8: GATHER Compliance: Guidelines for Accurate and Transparent Health Estimates Reporting 57
Section 9: References 59
Section 10: Appendix tables and figures 62
Table S1: Data inputs by source type. 62
Table S2: Number and ASMR per 100 000 by bacterial pathogen and GBD region, 2019 63
Table S3: YLLs by pathogen and infectious syndrome in 2019. 65
Table S4: Deaths by pathogen and the top 3 leading infectious syndromes by GBD super-region in 2019. 67
Figure S1: Pathogen distribution fatal and nonfatal estimation. 69
Figure S2: Age-standardised mortality rate per 100 000 population in 2019: *Staphylococcus aureus.* 70
Figure S3: Age-standardised mortality rate per 100 000 population in 2019: *Escherichia coli.* 70
Figure S4: Age−standardised mortality rate per 100 000 population in 2019: *Klebsiella pneumonia.* 71
Figure S5: Age−standardised mortality rate per 100 000 population in 2019: *Streptococcus pneumonia.* 71
Figure S6: Age−standardised mortality rate per 100 000 population in 2019: *Pseudomonas aeruginosa.* 72
Figure S7: Top 5 Leading BSI pathogens by GBD super−region, 2019. 72
Figure S8: Top 5 Leading LRI and thorax infections pathogens by GBD super−region, 2019. 73
Figure S9: Top 5 Leading Intra−abdominal infections pathogens by GBD super−region, 2019. 74
Figure S10: Global number of deaths by pathogen, age and sex group, 2019. 75
Section 11: Authors' Contributions 76

Section 1: List of abbreviations

Abbreviation	Full phrase
AHC	Angkor Hospital for Children
BARNARDS	Burden of Antibiotic Resistance in Neonates from Developing Societies
BD	Becton, Dickinson, and Company
BSI	bloodstream infections
CAI	community-acquired infection
CDC	Centers for Disease Control and Prevention
CFR	case fatality ratio
CHAIN	Childhood Acute Illness and Nutrition
CHAMPS	Child Health and Mortality Prevention Surveillance
cIAI	complicated intra-abdominal infection
COMRU	Cambodia Oxford Medical Research Unit
CTMRF	CHILDS Trust Medical Research Foundation
cUTI	complicated urinary tract infection
DHS	Demographic Health Surveys
EARS-Net	European Antimicrobial Resistance Surveillance Network
ECDC	European Centre for Disease Prevention and Control
GAM	generalised additive models
GBD	Global Burden of Diseases, Injuries, and Risk Factors Study
GBS	group B *Streptococcus*
GLM	generalised linear model
GPR	Gaussian process regression
HAI	hospital-acquired infection
HAQ Index	Healthcare Access and Quality Index
HHS	U.S. Department of Health and Human Services
ICD	International Classification of Diseases
ICU	intensive care unit
INICC	International Nosocomial Infection Control Consortium

iNTS	invasive non-typhoidal Salmonella
IORD	Infections in Oxfordshire Research Database
LRI	lower respiratory infection
MCoD	multiple causes of death data
MEPCO	multinomial estimation of partial and composite observations
MICS	Multiple Indicators Cluster Surveys
MITS	minimally invasive tissue sampling
MR-BRT	meta-regression—Bayesian, regularised, trimmed
MRC	Medical Research Council
OUCRU	Oxford University Clinical Research Unit
SDI	Socio-demographic Index
SEV	summary exposure value
SGUL-GARPEC	St. George's Hospital, University of London - Global Antimicrobial Resistance, Prescribing and Efficacy Among Neonates and Children
ST-GPR	spatiotemporal Gaussian process regression
TB	tuberculosis
TESSy	The European Surveillance System
UI	uncertainty interval
UPCH	Cayetano Heredia University
USDA	U.S. Department of Agriculture
UTI	urinary tract infection
VR	vital registration
WHO	World Health Organization

Section 2: Data sources

The data used for this study can be categorised into the following types: multiple causes of death (MCoD), hospital discharge, linkage, mortality surveillance, literature reviews, and microbial data; as well as estimates from the Global Burden of Diseases, Injuries, and Risk Factors Study (GBD) 2019.[2] More detailed information on data inputs are available at http://ghdx.healthdata.org/record/ihme-data/global-bacterial-antimicrobial-resistance-burden-estimates-2019.

Section 2.1: Multiple causes of death and vital registration (MCoD-VR)

Multiple cause of death (MCoD) data is a type of vital registration obtained from death certificates that contain the underlying cause of death, intermediate and immediate causes of death, and contributing conditions. ICD codes present in MCoD-VR also provided insight in many cases on the causative microorganism. MCoD data differ from other vital registration (VR) sources, because many countries have VR systems that only document the underlying cause of death. MCoD data were used in the sepsis, infectious syndrome, and pathogen distribution component models and data processing, and modelling methods can be found in sections 4 and 6. MCoD-VR data came from the following sources:

- United States National Vital Statistics System
- Brazil Mortality Information System
- National Institute of Statistics (Italy)
- Statistics South Africa
- National Institute of Statistics and Geography (Mexico)
- National Administrative Department of Statistics (Colombia)
- Taiwan Ministry of Health and Welfare

Section 2.2: Hospital discharge

Hospital admissions and discharge data are data sources collected from inpatient hospital and other clinical settings. These data include information on the primary and secondary diagnosis for each patient, as applicable, and were obtained from the sources listed below. ICD codes present in hospital discharge data also provided insight in many cases on the causative microorganism. Hospital data were used in the sepsis, infectious syndrome, pathogen distribution, and case fatality ratio component models and data processing, and modelling methods can be found in sections 4–6. Hospital discharge data came from the following sources:

- USA National Hospital Discharge Survey
- USA State Inpatient Databases
- Brazil Hospital Information System
- Italy Hospital Inpatient Discharges
- Sistema Automatizado de Egresos Hospitalarios (Mexico)
- Austria Hospital Inpatient Discharges
- New Zealand National Minimum Dataset
- Canada Discharge Abstract Database

Section 2.3: Microbial data with outcome

Microbial data are data sources from hospital and lab networks that collect pathogen cultures from patients. The culture results are linked to patient outcome, diagnoses, or both. Microbial data without these outcomes or diagnoses are listed in section 2.4. These data also include the specimen from which the pathogen was isolated and whether the infection was community- or hospital-acquired, if available. When hospital versus community acquisition was not specified, we used the difference between admission or diagnosis date and the specimen collection date, and if 48 hours or fewer had passed between those two dates, then the infection was assumed to be community-acquired. We assumed the infection was hospital-acquired when more than 48 hours had passed, consistent with CDC/National Healthcare Safety Network guidelines.[3] Microbial data with outcome were used in the case fatality ratio and pathogen distribution component models and data

processing, and modelling methods can be found in sections 5–6. Microbial data with outcome came from the following sources:

- **USA Becton, Dickinson, and Co. (BD) Insights, Research and Analytics Database microbiology test and in-patient hospital data:** data procured by BD via MedMined. Covers a range of regions in the United States from 2011 to 2017.
- **UK Infections in Oxfordshire Research Database (IORD):** patient microbiology and episodes data from Oxford University Hospitals NHS Foundation Trust.
- **International Nosocomial Infection Control Consortium (INICC) surveillance online system:** data from the INICC data collection software. ICU patient microbiology and hospital data from 50 countries across Latin America, Asia, the Middle East, eastern Europe, and Africa from 2009 to 2020.
- **Bulgaria antimicrobial resistance data**: Medical University of Varna in Varna, Bulgaria. Covers 2014–2020.
- **St. George's Hospital, University of London - Global Antimicrobial Resistance, Prescribing and Efficacy Among Neonates and Children (SGUL-GARPEC) Project bloodstream infection data:** Penta-sponsored global surveillance network focusing on neonatal and paediatric antimicrobial resistance and the organisms causing blood stream infections.
- **Burden of Antibiotic Resistance in Neonates from Developing Societies (BARNARDS)**: BARNARDS includes locations in Nigeria, South Africa, Pakistan, Rwanda, Bangladesh, Ethiopia and India from 2015 to 2018.
- **Lao-Oxford-Mahosot Hospital-Wellcome Trust Research Unit (LOMWRU). Children and adults with fever, inpatient admissions:** information from children and adults with fever who were admitted as inpatients between 1996 and 2019 to Mahosot Hospital, Vientiane, Laos. Microbial analysis was carried out by the Microbiology Laboratory at Mahosot Hospital.
- **Kumasi Centre for Collaborative Research in Tropical Medicine (KCCR), Kumasi, Ghana together with the Bernhard Nocht Institute for Tropical Medicine. Data on children and adults admitted in hospital with fever:** information from children and adults with fever admitted as inpatients at the Bernhard Nocht Institute for Tropical Medicine in Ghana between 2007 and 2015.
- **Vietnam Hospital for Tropical Diseases, Ho Chi Minh City. Hospital-acquired infections in ICU patients:** prospective observational study at the Oxford University Clinical Research Unit (OUCRU) in the Ho Chi Minh City Hospital for Tropical Diseases, Vietnam from November 2014 to January 2016 to assess the ICU-acquired colonisation and infections among adult patients with more than 48 hours of ICU stay.
- **Medical Research Council (MRC) Unit The Gambia. Diagnostic antimicrobial susceptibility testing:** information on hospital admission and discharge, pathogens cultured, resistance susceptibility test and antibiotics prescribed between 2005 and 2015 from the MCR Unit The Gambia, now part of the London School of Hygiene and Tropical Medicine.
- **Cambodia Oxford Medical Research Unit (COMRU) and Angkor Hospital for Children (AHC). Suspected invasive bacterial infection hospitalisations:** reports children aged 0–21 years who were hospitalised with suspected invasive bacterial infection between 2015 and 2018.
- **Taiwan hospital-acquired infections and outcomes:** infectious disease surveillance linked to vital registration from Taiwan (province of China).
- **Childhood Acute Illness and Nutrition (CHAIN) Network antimicrobial resistance data:** CHAIN Network study informs on hospitalised children under 2 years old with acute illness in Bangladesh, Burkina Faso, Pakistan, Kenya, Malawi, and Uganda.

- **Jordan King Abdulla University Hospital culture and sensitivity tests:** information on inpatients at the King Abdulla University Hospital in 2020 part of the Jordan University of Science and Technology.
- **Chennai, India Kanchi Kamakoti CHILDS Trust Medical Research Foundation (CTMRF) hospital inpatient data**

Section 2.4: Microbial data without outcome

Microbial data were also obtained from laboratories, which do not necessarily link to patients' hospital records nor information on their discharge disposition. These sources report specimen or site of infection, pathogens isolated, antimicrobial susceptibility tests, age and gender and other demographic characteristics. This information proved useful to inform pathogen distribution models and data processing, and modelling methods can be found in section 6. Microbial data without outcome came from the following sources:

- **SENTRY:** SENTRY Antimicrobial Surveillance Program established by JMI Labs in 1997. Sites are in the USA, Europe, Latin America, parts of Asia, and the Western Pacific
- **Germany National Point Prevalence Survey on Nosocomial Infections and Antibiotic Use (PPS HAI):** Point Prevalence Survey for 2016 data reporting the pathogen distribution for hospital-acquired infections.
- **Madagascar – Fondation Merieux:** data collected from inpatients with positive culture admitted in three hospital sites in Madagascar, funded by Fondation Merieux.
- **AMASS:** data collected in an automated tool by Oxford Tropical Network Research Units.
- **The European Surveillance System (TESSy):** managed by the European Centre for Disease Prevention and Control (ECDC), provided data from the following surveillance systems:
    - European Antimicrobial Resistance Surveillance Network (EARS-Net)
    - Food-and Waterborne Diseases and Zoonoses Surveillance Network.
    - Invasive Pneumococcal Disease Surveillance Network, including discharge disposition.
    - Gonococcal Antimicrobial Surveillance Programme.
    - Healthcare Associated Infections Surveillance Network (ICU protocol), including discharge disposition.
    - European Tuberculosis Surveillance Network
    - European Surveillance of Antimicrobial Consumption Network

The European Union/European Economic Area (EU/EEA) data were obtained from the European Surveillance System (TESSy) as provided by Austria, Belgium, Croatia, Cyprus, Czechia, Denmark, Estonia, Finland, France, Germany, Greece, Hungary, Iceland, Ireland, Italy, Latvia, Luxembourg, Malta, Netherlands, Norway, Poland, Portugal, Romania, Slovakia, Slovenia, Spain, Sweden, and the United Kingdom, and released by the European Centre for Disease Prevention and Control (ECDC).

- **Pfizer ATLAS Programme:** the Antimicrobial Testing Leadership and Surveillance (ATLAS) database includes the Tigecycline Evaluation Surveillance Trial (TEST), the Assessing Worldwide Antimicrobial Resistance Evaluation (AWARE) and the International Network for Optimal Resistance Monitoring (INFORM) programs. The study spans in coverage across more than 70 countries between 2004 and 2017.
- **Malawi Queen Elizabeth Hospital microbiology tests of blood specimens:** microbiology tests of blood specimens from inpatients at the Queen Elizabeth Hospital in Malawi from 1998 to 2016, part of the Institute of Infection and Global Health, University of Liverpool in

collaboration with the Malawi-Liverpool-Wellcome Trust and the Wellcome Trust Sanger Institute.

- **Central African Republic National Laboratory of Clinical Biology and Public Health:** data collected by the Laboratoire National de Biologie Clinique et de Sante Publique in Central African Republic between 2017 and 2020.
- **The Ethiopian AMR surveillance:** conducted from July 2018 to July 2020 across sentinel surveillance sites and the National AMR Surveillance Coordinating Centre for the Ethiopian Public Health Institute.
- **WHO Meningitis surveillance:** sentinel hospital surveillance of suspected meningitis cases among children under 5 years old and positive cultures, provided by the World Health Organization (WHO) Global Rotavirus, Invasive Bacterial Vaccine Preventable Diseases Surveillance Network Collaboration from 2008 to 2020.
- **United States Active Bacterial Core Surveillance (ABCs) Reports:** case reports on healthcare-associated Infections and community interface infections from the Emerging Infections Program Network coordinated by the Center for Disease Control and Prevention (CDC).

Section 2.5: Literature review of the microbial aetiology of meningitis, maternal and neonatal sepsis, lower respiratory infections, urinary tract infections, skin infections, peritonitis, and bone and joint infections

We conducted literature searches to obtain input data for the following components in the analysis: meningitis aetiology and case fatality, maternal and neonatal sepsis aetiology, lower respiratory infections (LRIs) aetiology, urinary tract infections (UTIs) aetiology, skin infections aetiology, intra-abdominal infection aetiology, and bone and joint infections aetiology. Search strings including boolean operators were used in PubMed to systematically search for the causative microorganisms for these infectious syndromes.

Section 2.5.1: Meningitis infection aetiology

((meningitis[title]) AND (1990/05/01[PDat] : 2018/12/31[PDat]) AND ((etiolog*[title/abstract]) AND Humans[MeSH Terms])

Section 2.5.2: Maternal sepsis

(('puerperal Sepsis'[title] OR 'maternal sepsis'[title] OR "Puerperal Infection/microbiology"[Mesh]) AND (1990/05/01[PDat] : 2018/12/31[PDat]) AND (etiolog*[title/abstract] OR microbiology[title/abstract] OR microbiology[Mesh] OR ("Bacteremia/microbiology"[Mesh])) NOT review[ptyp]

Section 2.5.3: Neonatal sepsis

(('neonatal sepsis'[title] OR ('infant'*[title] AND ('sepsis'[title] OR 'bacteremia'[title]) AND (etiolog*[title/abstract] OR microbiology[title/abstract] OR microbiology[Mesh] OR ("Bacteremia/microbiology"[Mesh]))

Section 2.5.4: LRI aetiology

((“lower respiratory”[title] OR pneumonia[title]) AND (1990/05/01[PDat] : 2018/12/31[PDat]) AND ((etiolog*[title/abstract]) AND Humans[MeSH Terms]) NOT(autoimmune[title/abstract] OR COPD [title/abstract] OR “cystic fibrosis”[title/abstract] OR Review[ptyp])

Section 2.5.5: Urinary tract infections aetiology

("complicated"[Title/Abstract] OR "uncomplicated"[Title/Abstract]) AND (("Cystitis/etiology"[majr:noexp] OR "Cystitis/microbiology"[majr:noexp]) OR ("Pyelonephritis/etiology"[marj:noexp] OR "Pyelonephritis/microbiology"[majr:noexp]) OR ("Urinary Tract Infections/etiology"[majr:noexp] OR "Urinary Tract Infections/microbiology"[majr:noexp])) OR ("Urinary tract infections"[tiab] AND ("etiology"[tiab] OR "microbiology"[tiab]))

(("urinary tract infection*"[title]) AND (1990/05/01[PDat] : 2018/12/31[PDat]) AND ((etiolog*[title/abstract] OR "Urinary Tract Infections/microbiology"[Mesh]) AND Humans[MeSH Terms]) NOT Review[ptyp]

Section 2.5.6: Skin infections aetiology

(("Cellulitis/epidemiology"[majr:noexp] OR "Cellulitis/etiology"[majr:noexp] OR "Cellulitis/microbiology"[majr:noexp]) OR ("Pyoderma/epidemiology"[majr:noexp] OR "Pyoderma/etiology"[marj:noexp] OR "Pyoderma/microbiology"[majr:noexp]) OR "Pressure Ulcer/microbiology"[majr:noexp])

(“skin and soft tissue infection”[title] OR cellulitis[title] OR erysipelas[title]) AND (1990/05/01[PDat] : 2018/12/31[PDat]) AND (etiolog*[title/abstract] OR "Cellulitis/microbiology"[Mesh]) AND Humans[MeSH Terms] NOT Review[ptyp]

Section 2.5.7: Intra-abdmoinal infection aetiology

(("Peritonitis/epidemiology"[majr:noexp] OR "Peritonitis /etiology"[majr:noexp] OR "Peritonitis /microbiology"[majr:noexp]) OR ("Intraabdominal infections/epidemiology"[majr:noexp] OR "Intraabdominal infections /etiology"[marj:noexp] OR "Intraabdominal infections /microbiology"[majr:noexp]) OR ("abdominal abscess/epidemiology"[majr:noexp] OR " abdominal abscess /etiology"[majr:noexp] OR "abdominal abscess/microbiology"[majr:noexp]))

Section 2.5.8: Bone and joint infections aetiology

("Osteomyelitis/etiology"[majr:noexp] OR "Osteomyelitis/microbiology"[majr:noexp] NOT 'chronic') OR ("Arthritis, infectious/etiology"[marj:noexp] OR "Arthritis, infectious/microbiology"[majr:noexp] NOT ‘lyme’)

Section 2.5.9: Exclusion criteria for literature reviews of microbial aetiologies

Exclusions Part 1: Excluded from initial database search:

- Non-peer reviewed papers or publications
- Case reports

Exclusions Part 2: Excluded from Title/Abstract screening:

- Exclusions by study type:
  - Case control studies
  - Intervention studies
  - Studies based entirely on modelling with no raw input data
- Sampling occurred in selected populations

Exclusions Part 3: Excluded from Full Text Review

- Sample size lower than 50
- Studies that included just 1 pathogen

- Studies that reported combinations of pathogens together without disaggregating by pathogen
- Studies based entirely on modelling with no raw input data
- Studies with a biased sampling framework

Limitations of Literature Reviews

- Only included studies in English
- Search strings included constraints on year

Section 2.6: Mortality surveillance

Mortality surveillance data were used in the sepsis, syndrome, and pathogen distribution models; full details on these models can be found in sections 4 and 6.

- **Child Health and Mortality Prevention Surveillance (CHAMPS):** Under-5 mortality surveillance sites in South Africa, Mali, Bangladesh, Kenya, Ethiopia, and Mozambique. Researchers use minimally invasive tissue sampling (MITS) to gather information about pathogens involved and are able to discern a more accurate cause of death.

Section 2.7: Linkage (mortality only)

Linkage data were used in sepsis and infectious syndrome models; full details on these models can be found in section 4. Mortality-only linkage data include:

- **Italy Friuli-Venezia Giulia MCoD data**
  New Zealand linked national minimum dataset to mortality collection data

Section 3: Summary of GBD 2019 estimation process

A comprehensive description of data sources, data quality, statistical modelling and analyses for GBD 2019 have been reported elsewhere.[2] A brief summary of the fatal and non-fatal estimation processes are briefly summarised below.

Section 3.1: GBD 2019 cause of death estimation process

The overarching steps for the fatal estimation process for each age, sex, location, and year are to first estimate all-cause mortality rates, then calculate cause-specific mortality rates, and finally scale the cause-specific mortality rates to the all-cause mortality rates for internal consistency. First, all-cause mortality is estimated using 7417 sources as data inputs for under-5 mortality estimation and 7355 sources as data inputs for adult mortality estimation. ST-GPR was used to produce estimates of HIV-free mortality rate for every location-year after adjusting for completeness and other known biases in the input data. Added to this HIV-free mortality rate are the HIV-specific mortality rate and deaths from fatal discontinuities, or shocks, which are events that are stochastic in nature and cannot be modelled, such as natural disasters and conflicts. GBD then estimated the cause-specific mortality rates of 301 diseases and injuries. This cause of death analysis utilises 19 354 sources covering 2525 country years in the cause of death (CoD) database. There are eight types of data sources in the CoD database: vital registration, verbal autopsy,[4] cancer registry, police records, sibling history, surveillance, survey/census, and minimally invasive tissue sampling (MITS) diagnoses. VR is considered the most comprehensive source of cause of death data, but less than half the world's population has deaths captured in a VR system (appendix figure S6), so causes of death statistics are supplemented with other data types. These various data sources are largely ICD coded causes of death and use heterogenous ICD versions so are standardised to GBD causes of death. Once standardised and adjusted for known biases due to ICD classification changes,[5] garbage

coding,[5–7] HIV correction,[8] stochastic noise,[2] and completeness,[9] causes of death are modelled using CODEm[10] to determine the cause fraction for each underlying cause of death by age, sex, year, and location. CODEm provides an ensemble prediction based on a combination of candidate models that vary across outcome and covariate combinations chosen for out-of-sample predictive performance. Because each cause is modelled independently, it is possible the sum of these models will not equal the all-cause mortality estimates, so cause-specific results are run through the CoDCorrect process to make cause-specific and all-cause mortality estimates internally consistent. This process rescales cause-specific estimates to the all-cause mortality envelope.

Section 3.2: GBD 2019 non-fatal estimation process

Non-fatal health outcomes are estimated using DisMod-MR 2.1, a Bayesian-regression analytical tool that synthesises various data inputs to produce estimates of disease incidence and prevalence. The data used for this analysis include systematic reviews done at the Institute of Health Metrics and Evaluation (IHME), data from household surveys including the demographic and health surveys, multiple indicator cluster surveys, living standards measurement surveys, reproductive health surveys, administrative claims data, inpatient hospital discharge records, outpatient hospital data, disease registries, programme-level data on disease burden from government agencies, surveillance system data on disease burden, and sources suggested to us by in-country collaborators and surveys identified in major multinational survey data catalogues such as the WHO Central data catalog. 51 272 sources were used for this analysis, 31 499 reporting incidence and 19 773 reporting prevalence. Data from these sources are extracted. Pre-modelling bias adjustments are made using crosswalking to account for various sources of bias, such as heterogeneous case definitions and methods of measurement. The pre-modelling bias adjustments are made using the MR-BRT environment, a meta-regression tool that allows for Bayesian priors, regularization, and trimming and has been described in greater detail previously.[11] Using these bias-adjusted data an estimate of prevalence and incidence for each cause is produced using the DisMod-MR 2.1 modelling framework. DisMod-MR 2.1 accepts all available data on mortality, incidence, prevalence, and remission and uses a compartmental model to enforce consistency between all quantities.

Section 4: Deaths where infection plays a role and infectious syndrome estimation

Section 4.1: Input data

Section 4.1.1: Multiple causes of death

MCoD data are individual-based records that provide underlying causes of death and two or more intermediate causes in the chain of death. Additionally, each record includes age, sex, residence, and the date of death.

Section 4.1.2: Hospital record with multiple diagnoses and discharge status of death

This type of data is an individual-based hospital record of a patient that provides the main diagnosis and two or more additional diagnoses. Additionally, each record includes age, sex, residence, date of admission, date of discharge, and outcome (dead or alive). Only hospital discharges with discharge status of death were used in this component model, since we aimed to estimate the fraction of deaths that involve infection and the infectious syndrome distribution of those deaths.

Section 4.1.3: Linkage data

Linkage data are generated using probabilistic methods in a defined population that link individual-based hospital data to individual-based MCoD data. Linkage data offer a wider dataset that includes

main diagnosis, other diagnoses, underlying cause of death, and intermediate causes of death in the chain.

Section 4.1.4: Mortality surveillance (Child Health and Mortality Prevention Surveillance [CHAMPS])

The CHAMPS network tracks the causes of under-5 mortality and stillbirths at sites in sub-Saharan Africa and south Asia through epidemiological surveillance of under-5 deaths and stillbirths utilising minimally invasive tissue sampling (MITS), laboratory diagnostics including conventional and advanced histopathology and molecular screening of various pathogens, verbal autopsy, and available clinical and demographic data.

Table 4.1.5: Input different data point for calculation of fraction of death by sepsis in different underlying causes

Location	Data type	Years	Year range	Deaths
United States	MCoD	38	1980–2017	82,453,798
	Hospital data with fatal outcome	31	1980–2010	2,028,371
	Linkage data			
Brazil	MCoD	19	1999–2017	16,930,050
	Hospital data with fatal outcome	2	2015–2016	294,461
	Linkage data			
Italy	MCoD	13	2003–2015	7,640,383
	Hospital data with fatal outcome	12	2005–2016	2,385,430
	Linkage data	16	2003–2018	112,555
South Africa	MCoD	20	1997–2016	4,696,348
	Hospital data with fatal outcome			
	Linkage data			
Mexico	MCoD	8	2009–2016	4,336,713
	Hospital data with fatal outcome	7	2003–2009	168,582
	Linkage data			
Colombia	MCoD	20	1998–2017	3,624,771
	Hospital data with fatal outcome			
	Linkage data			
Taiwan (province of China)	MCoD	10	2007–2016	1,189,309
	Hospital data with fatal outcome			
	Linkage data			
Austria	MCoD			
	Hospital data with fatal outcome	14	2001–2014	461,538
	Linkage data			
New Zealand	MCoD			
	Hospital data with fatal outcome	18	2000–2017	169,454
	Linkage data	11	2000–2010	151455
Canada	MCoD			
	Hospital data with fatal outcome	16	1994–2009	38,405
	Linkage data			
CHAMPS Surveillance Sites	MITS	3	2017–2019	870
Total	MCoD	128	1980–2017	120,871,372
	Hospital data with fatal outcome	100	1980–2017	5,546,241
	Linkage data	27	2000–2018	264,010

	MITS	3	2017–2019	870

Section 4.2: Data processing

Data for the USA, Brazil, Italy, South Africa, and Mexico were extracted at the subnational level by GBD 2019 age groups, sex, year, and causes of death and/or diagnoses, while data for the remaining countries and territories were analysed at the national level. This allowed us to expand the location-years of data that we had for each Socio-demographic Index (SDI)[12] value.

Section 4.3: Mapping the data

Prepared data were mapped to GBD causes. The GBD cause list is a mutually exclusive and collectively exhaustive list of diseases and injuries. The GBD cause list is organised hierarchically to accommodate different purposes and needs of various users. The first two levels aggregate causes into general groupings. At Level 1, there are three cause groups: communicable, maternal, neonatal, and nutritional diseases (Group 1 diseases); non-communicable diseases (Group 2); and injuries (Group 3). These Level 1 aggregates are subdivided at Level 2 of the hierarchy into 22 cause groupings (eg, neonatal disorders, neurological disorders, and transport injuries). The disaggregation into Levels 3 and 4 contains the finest level of detail for causes captured in GBD 2019.

The underlying cause of death or main diagnosis for each record in the data was mapped to a GBD cause. After the mapping of underlying cause, we used the GBD 2019 garbage code redistribution algorithm (see appendix 1, section 2.4 in Vos et al.[2]) to ensure that all deaths had a plausible and specific underlying cause of death. The redistribution of garbage codes for underlying causes of death followed the same age and sex restrictions as GBD 2019. We did not redistribute garbage codes in the chain causes because the concept of a garbage code applies only to plausible underlying cause of death (see Rudd et al.[13] and appendix 1, section 2.5 in Vos et al.[2]).

Section 4.4: Intermediate cause and infectious syndrome mapping hierarchy

Section 4.4.1: Intermediate cause mapping

Within our modelling framework, an infectious syndrome is the infection directly responsible for sepsis and serves as the bridge between the underlying cause of death and sepsis. Infectious syndromes can be both underlying causes of death and intermediate causes of death.

For mapping underlying and intermediate causes of death and hospital diagnoses to sepsis and infectious syndromes, we designed a new map, called "AMR, sepsis, and infectious syndrome map". This map is a list of mutually exclusive and collectively exhaustive infectious syndromes that we divided into four levels to form an infectious syndrome hierarchy.

Each level of infectious syndrome is mutually exclusive and collectively exhaustive. Furthermore, the infectious syndrome hierarchy is internally consistent across any metric (eg, number, cause fraction)—aggregating across Level 3 syndromes gives us Level 2 syndromes, aggregating the Level 2 syndromes gives us Level 1 syndromes, and the total of Level 1 syndromes is equal to the value of sepsis (figure 4.4.2.1).

Level 0: All International Classification of Diseases 9th (ICD-9) or 10th revision (ICD-10) coded deaths divided into three groups:

- Explicit sepsis (A40, R65.2 in ICD-10 and 039 in ICD-9): Any death has specific ICD code for sepsis in the MCoD chain or hospital diagnoses was considered explicit sepsis[13]

- Implicit sepsis: Any death that has an infectious disease code in the underlying cause or cause chain and a specific organ dysfunction code was considered implicit sepsis
- Non-sepsis: Any death that does not meet either of the two above criteria.

Of the estimated infection-related deaths with explicit sepsis or implicit sepsis and infectious diseases, 59.4% occur with communicable, maternal, neonatal, and nutritional underlying causes of death. 38.9% infection related deaths occur with non-communicable disease as the underlying cause of death, and 1.7% occur with injuries as the underlying cause of death.

Level 1: All implicit and explicit sepsis deaths were divided into 12 Level 1 infectious syndromes and an “other” category (table 4.4.1.1).

Table 4.4.1.1: Level 1 of infectious syndromes

	**Infectious syndrome**
1	Bacterial infections of the skin and subcutaneous systems
2	Bloodstream infections
3	Gonorrhoea and chlamydia
4	Diarrhoea
5	Endocarditis and other cardiac infections
6	Infections of bones, joints, and related organs
7	Lower respiratory infections and all related infections in the thorax
8	Meningitis and other bacterial central nervous system infections
9	Peritoneal and intra-abdominal infections
10	Tuberculosis
11	Typhoid, paratyphoid, and invasive non-typhoidal Salmonella
12	Urinary tract infections and pyelonephritis
13	Other infections

Level 2: Each Level 1 infectious syndrome was divided into Level 2 infectious syndromes based on the pathogen type (eg, bacterial, fungal, viral) causing the infection. Examples include specified bacterial, unspecified bacterial, fungal, viral, and unspecified pathogen.

Level 3: Each specified bacterial infectious syndrome in Level 2 was divided to Level 3 infectious syndromes by the bacterium causing infection.

Figure 4.4.2.1. Infectious syndrome hierarchy

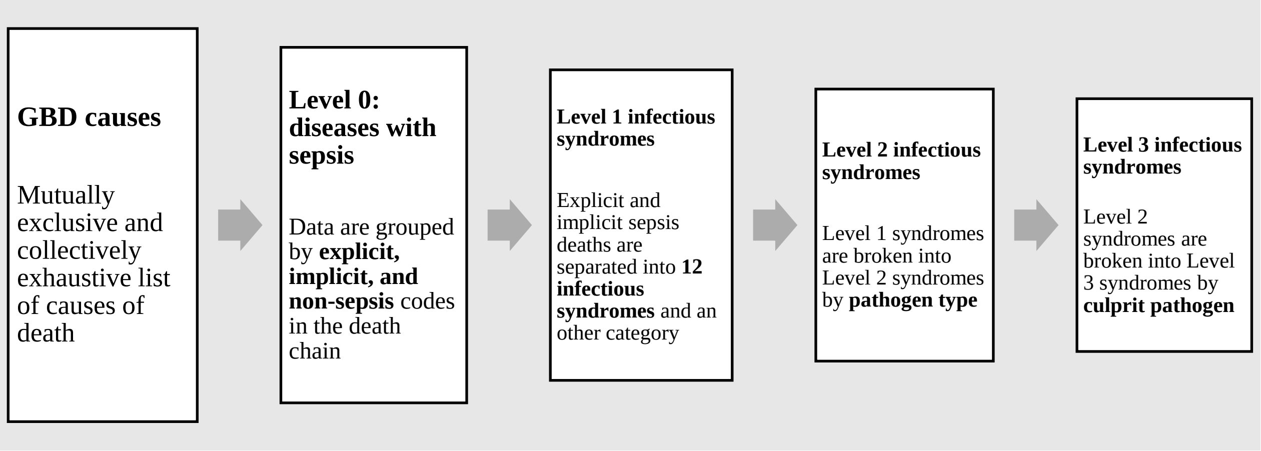

Section 4.4.2: Informative ranking

Due to our data often having multiple diagnoses associated with each record, a single case of sepsis could potentially map to multiple candidate infectious syndromes. Because multiple infectious syndrome assignments pose a risk of double counting, we employed an informative ranking hierarchy. The informative ranking allowed us to determine the infectious syndrome that provided the most information on the culprit pathogen. The goal of this hierarchy was to produce the most accurate pathogen burden estimate such that when there were multiple infectious syndromes, we prioritised the syndrome with the most distinctive distribution. For example, bloodstream infections (BSIs) are common infections in sepsis but there is often an earlier source of the infection such as a UTI, cellulitis, or LRI, and each has a unique pathogen distribution that provides more information than the distribution of BSI. In the event that a patient record reflected both BSI and LRI, we would assign the infectious syndrome based on the pathogen distribution that would be the most proximal aetiologic syndrome, LRI (see table 4.4.2.1).

Table 4.4.2.1. Level 1 Infectious syndrome informative ranking hierarchy

Organised from most informative (top) to least (bottom).

Level 1 infectious syndrome informative ranking hierarchy
Meningitis and other bacterial central nervous system infections
Endocarditis and other cardiac infections
Peritoneal and intra-abdominal infections
Lower respiratory infections and all related infections in the thorax
Bacterial infections of the skin and subcutaneous systems
Infections of bone, joints, and related organs
Diarrhoea
Urinary tract infections and pyelonephritis
Other infections
Bloodstream infections

Section 4.4.3: Two modelling pathways

After mapping the underlying and chain causes of death, our database went through two separate modelling pathways. The first model estimated the fraction of deaths that are sepsis-related in each GBD cause; these sepsis-related deaths for non-infectious GBD causes were combined with GBD deaths for infectious causes to create the total envelope of all deaths where infection plays a role. The second pathway estimated each infectious syndrome as a fraction of sepsis-related mortality in each GBD cause. In the last step of infectious syndrome estimation, the fractions of sepsis by Level 1 infectious syndromes were squeezed to sum to one so as to not exceed the sepsis mortality envelope and multiplied by the sepsis estimate in each GBD cause by country and territory, age, and sex in 2019.

Section 4.5: First pathway: deaths where infection plays a role

Section 4.5.1: Sepsis model

We used a mixed-effects binomial logistic regression to model the logit of the fraction of sepsis-related deaths by GBD cause-age-sex-location, consistent with the modelling approach used by Rudd et al.[13] Sex and Healthcare Access and Quality Index (HAQ Index)[14] were included as covariates and a nested random effect on underlying cause of death was included. A separate model was run for each

GBD 2019 age group (0–6, 7–27, 28-364 [days], 1–4, 5–9, 10–14, 15–19, 20–24, 25–29, 30–34, 35–39, 40–44, 45–49, 50–54, 55–59, 60–64, 65–69, 70–74, 75–79, 80–84, 85–89, 90–94, 95+ [years]):

$$sepsis\ related\ deaths \sim B(total\ deaths, sepsis\ fraction) \qquad (4.5.1.1)$$

$$logit(sepsis\ fraction) = \beta_0 + \beta_1 * HAQ\ Index + \beta_2 * sex + \pi_{level\ 1, level\ 2}$$

Where $\pi_{level\ 1, level\ 2}$ is a nested random effect on underlying cause of death. The nested random-effect's structure in the model on underlying cause of death allowed the prediction of sepsis fractions where data were limited by borrowing information from diseases within the same group. There were 22 groups of underlying causes of death, each categorised by physiological relatedness. We produced our predictions and uncertainty intervals (UIs) by generating 1000 draws from the normal distribution of the fixed coefficients, separately for each GBD location, age group, sex, and cause in 2019. The means of our results were used for the point estimates and the 95% UIs were delineated using the 2.5th and 97.5th percentiles of the draws. Uncertainty is attributable to sample size variability between data sources, data availability, and model specifications.

All underlying causes of death that are infectious diseases were included in the model; however, for these causes we used the GBD death estimates rather than the modelled sepsis estimate, since infection inherently plays a role in these deaths even if the pathway doesn't include sepsis. These causes and their associated infectious syndromes are listed in table 4.5.1.1.

Table 4.5.1.1. Underlying causes that are infectious diseases and their corresponding syndromes

Cause name	Infectious syndrome
Appendicitis	Peritoneal and intra-abdominal infections
Bacterial skin diseases	Bacterial infections of the skin and subcutaneous systems
Chlamydial infection	Gonorrhoea and chlamydia
Diarrhoeal diseases	Diarrhoea
Endocarditis	Endocarditis and other cardiac infections
Gonococcal infection	Gonorrhoea and chlamydia
Invasive non-typhoidal Salmonella	Typhoid, paratyphoid, and invasive non-typhoidal Salmonella
Lower respiratory infections	Lower respiratory infections and all related infections in the thorax
Maternal sepsis and other maternal infections	Bloodstream infections
Meningitis	Meningitis and other bacterial central nervous system infections
Neonatal sepsis and other neonatal infections	Bloodstream infections
Paratyphoid fever	Typhoid, paratyphoid, and invasive non-typhoidal Salmonella
Tuberculosis	Tuberculosis
Typhoid fever	Typhoid, paratyphoid, and invasive non-typhoidal Salmonella
Upper respiratory infections	Lower respiratory infections and all related infections in the thorax
Urinary tract infections and interstitial nephritis	Urinary tract infections and pyelonephritis

For all other causes, we calculated the number of sepsis-related deaths in 2019 by multiplying our predictions of cause-, age group-, sex-, year-, and location-specific sepsis fractions by GBD 2019 death estimates. Finally, we aggregated our results to arrive at regional and global sepsis-related mortality in non-infectious underlying causes of death, which we combined with the GBD infectious disease deaths estimates to create the mortality envelope of all deaths related to infection.

For transparency, histograms of the available input data by HAQ Index are shown below. MCoD input data is used to estimate the proportion of non-infectious disease that involves sepsis, while the GBD mortality data for Group 1 causes (communicable, maternal, neonatal, and nutritional diseases) is inclusive and representative of the input data used to estimate mortality associated with primary infection underlying cause.

Figure 4.5.1.1. MCoD input data by HAQ Index

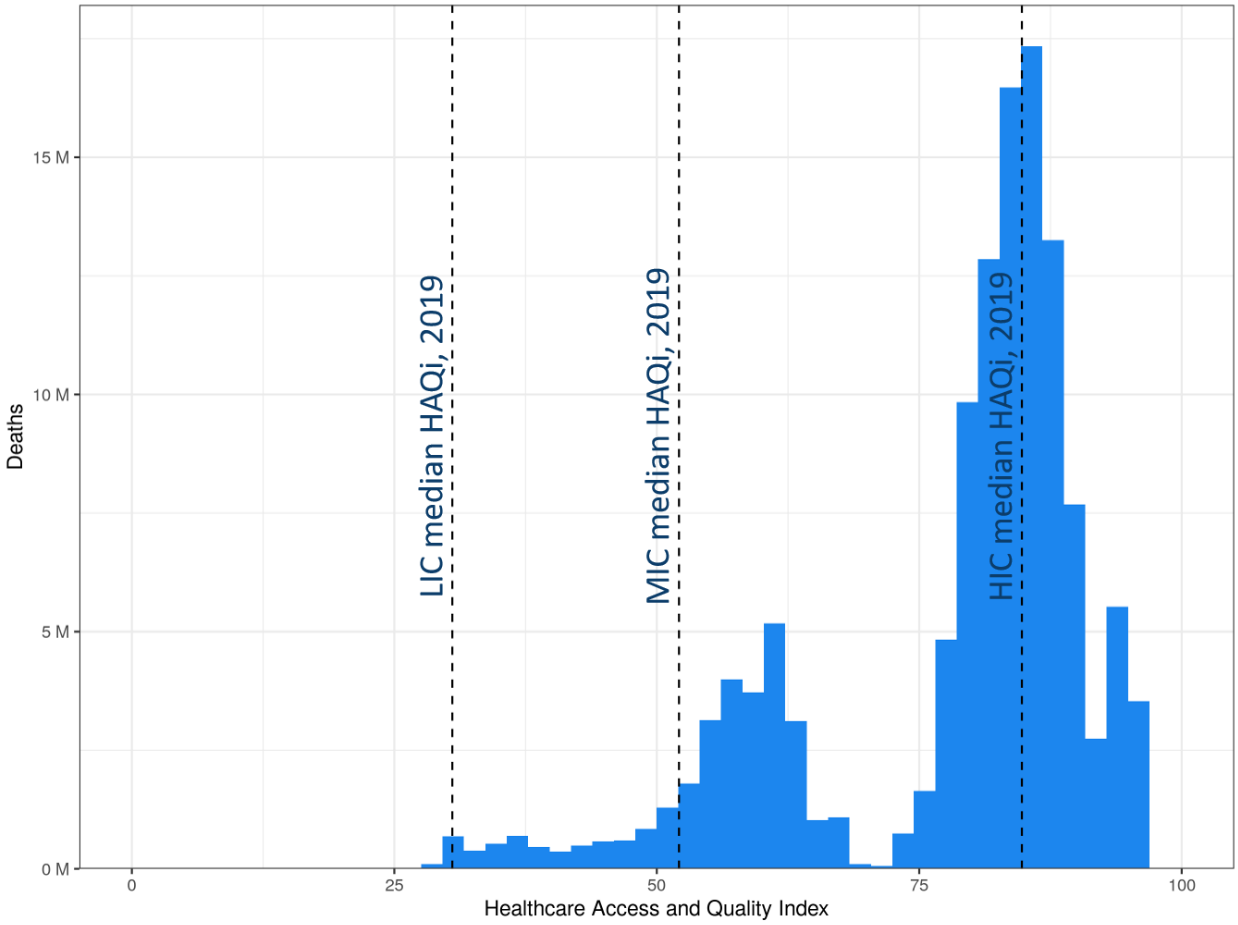

Figure 4.5.1.2. GBD mortality input data for Group 1 (communicable, maternal, neonatal, and nutritional diseases) by HAQ Index

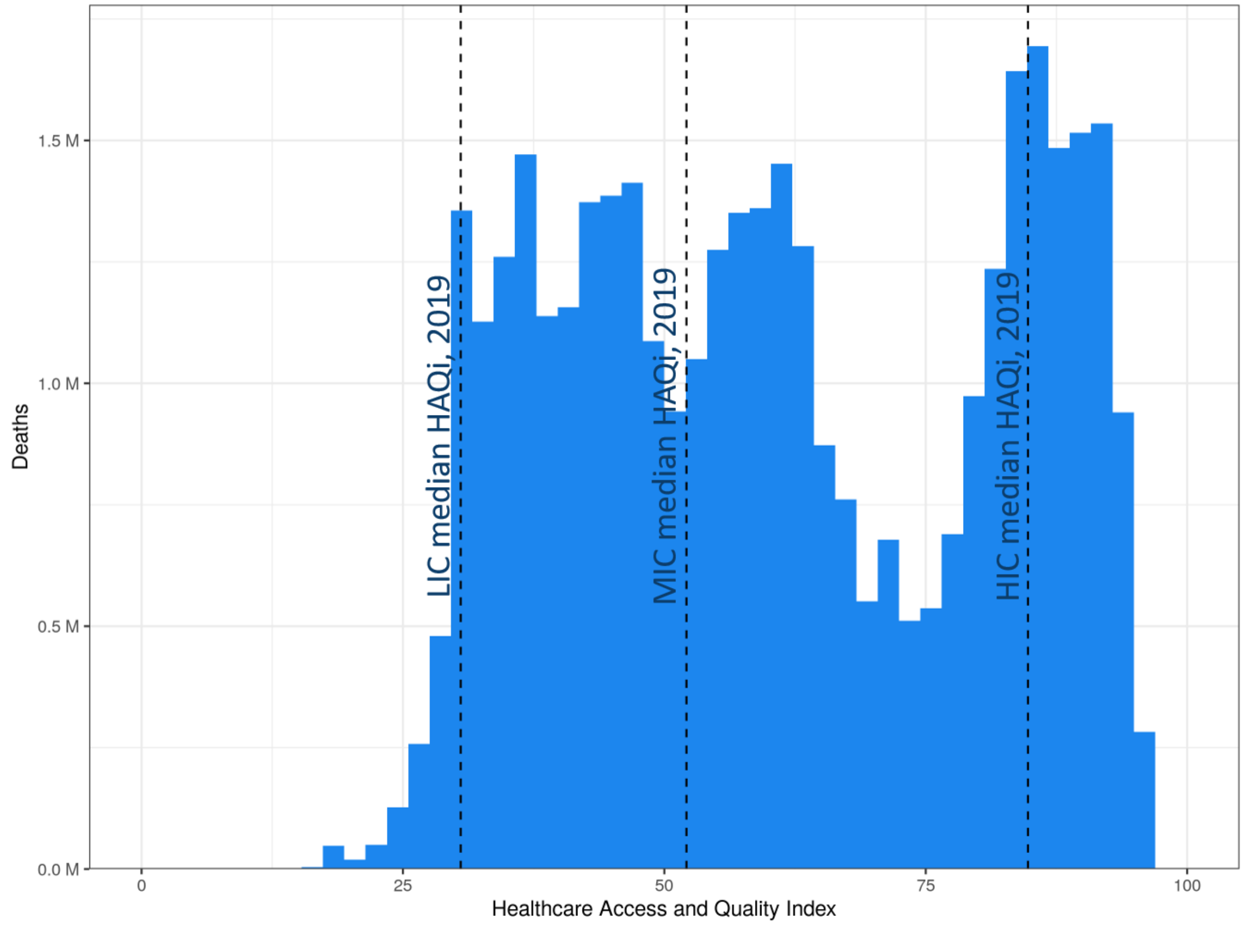

HIC = high-income country, LIC = low-income country, MIC = middle-income country

Section 4.6: Second pathway: fraction of deaths where infection plays a role by infectious syndrome in each GBD cause

We used a mixed-effects binomial logistic regression to model the logit of the infectious syndrome fraction of sepsis-related mortality by GBD cause. The model covariates varied by infectious syndrome (table 4.6.1). All models included HAQ Index as a covariate and most included a summary exposure value (SEV) scalar calculated for GBD 2019.

The pathogen distribution for hospital-acquired infections (HAIs) and community-acquired infections (CAIs) differs markedly for some infectious syndromes.[15–20] To more accurately estimate the burden of pathogens responsible for infection, we separated infectious syndromes into hospital-acquired and community-acquired for LRI+ and UTI. For all ICD-coded administrative datasets (hospital discharge, MCoD, and linkage), we assumed that an infection was community-acquired if it was the primary diagnosis or underlying cause of death. Similarly, an infection was considered hospital-acquired if it was not the primary diagnosis or underlying cause of death. We recognise that this is a strong assumption that will not always be correct; however, there is no established method for determining HAI versus CAI in administrative data.[21,22] We considered it to be more important to estimate hospital- and community-acquired separately to account for their distinct pathogen distributions despite the strong assumptions involved. We present the fraction of all infectious syndrome deaths in 2019 that our model predicted to be hospital-acquired for LRI+ and UTI in table

4.6.2 for transparency.

Table 4.6.1: Infectious syndrome model covariates and age groups

Infectious syndrome	Covariates	Age groups modelled
Bloodstream infections	HAQ Index[14]<br>Sex<br>SEV scalar of maternal sepsis[23]<br>SEV scalar of neonatal sepsis[23]	GBD 2019 age groups
Infections of bone, joints, and related organs	HAQ Index<br>Sex	0–9, 10–14, 15–19, 20–24, 25–39, 40–44, 45–49, 50–54, 55–59, 60–64. 65–69, 70–74, 75–79, 80–84, 85–89, 90–94, 95+
Endocarditis and other cardiac infections	HAQ Index<br>Sex<br>SEV scalar of age-standardised endocarditis[23]	GBD 2019 age groups
Meningitis and other bacterial central nervous system infections	HAQ Index<br>Sex<br>SEV scalar of age-standardised meningitis[23]	GBD 2019 age groups
Diarrhoea	HAQ Index<br>Sex<br>SEV scalar of age-standardised diarrhoea[23]	GBD 2019 age groups
Other infections	HAQ Index<br>Sex	GBD 2019 age groups
Peritoneal and intra-abdominal infections	HAQ Index<br>Sex	GBD 2019 age groups
CAI lower respiratory infections and all related infections in the thorax	HAQ Index<br>Sex<br>SEV scalar of age-standardised LRIs[23]	Neonatal, Post neonatal–5, 5–69, 70+
HAI lower respiratory infections and all related infections in the thorax	HAQ Index<br>Sex<br>SEV scalar of age-standardised LRIs	Neonatal, Post neonatal–5, 5–69, 70+
Bacterial infections of the skin and subcutaneous systems	HAQ Index<br>Sex<br>SEV scalar of age-standardised no access to handwashing facility[23]	GBD 2019 age groups
CAI urinary tract infections and pyelonephritis	HAQ Index<br>Sex<br>SEV scalar of age-standardised no access to handwashing facility	0–39, 40+
HAI urinary tract infections and pyelonephritis	HAQ Index<br>Sex<br>SEV scalar of age-standardised no access to handwashing facility	0–39, 40+

CAI=community-acquired infection. HAI=hospital-acquired infection. HAQ Index=Healthcare Access and Quality Index. LRI=lower respiratory infection. SEV=summary exposure value. SEVs are a risk-weighted prevalence calculated based on exposure, from 0, where the entire population (among the age groups where exposure is possible) is exposed at the minimum risk level, to 100, where the entire population is exposed at the maximum risk exposure level.

Table 4.6.2: Model predictions for proportion of deaths in 2019 that were hospital-acquired by GBD super-region and infectious syndrome

Infectious syndrome	Super-region	Proportion (95% UI)
Lower respiratory infections and all related infections in the thorax	Global	17.4% (13.1 - 22.4)
	Southeast Asia, east Asia, and Oceania	23.3% (18.0 - 28.9)
	Central Europe, eastern Europe, and central Asia	27.9% (20.5 - 35.8)

	High-income	17.8% (12.5 - 24.8)
	Latin America and Caribbean	24.8% (19.1 - 30.7)
	North Africa and Middle East	22.2% (17.7 - 26.8)
	South Asia	16.4% (13.0 - 20.1)
	Sub-Saharan Africa	9.3% (6.9 - 12.8)
Urinary tract infections and pyelonephritis	Global	39.1% (32.4 - 61.5)
	Southeast Asia, east Asia, and Oceania	64.5% (48.4 - 74.7)
	Central Europe, eastern Europe, and central Asia	48.4% (32.4 - 61.5)
	High-income	27.2% (15.8 - 40.7)
	Latin America and Caribbean	28.5% (20.2 - 40.6)
	North Africa and Middle East	73.0% (58.2 - 80.0)
	South Asia	29.9% (20.2 - 40.7)
	Sub-Saharan Africa	24.9% (18.0 - 31.1)

The infectious syndrome models were specified as mixed-effects binomial logistic regressions, one for each infectious syndrome and age group:

$$syndrome\ related\ deaths \sim B(total\ sepsis\ deaths, syndrome\ fraction) \qquad (4.6.2.1)$$

$$logit(syndrome\ fraction) = \beta_0 + \beta * X + \pi_{level\ 1, level\ 2}$$

where β and X are vectors of length $n + 1$ for n covariates and $\pi_{level\ 1, level\ 2}$ is a nested random effect on underlying cause of death. The granularity of the age groups estimated for each infectious syndrome was chosen based on the age pattern of the infectious syndrome and the limitations of data sparsity.

As in the first pathway, we derived our predictions and UIs by generating 1000 draws from the normal distribution of the fixed coefficients separately for each GBD location, age group, sex, and cause in 2019. We used the means of our results for the point estimates and the 95% UIs were delineated using the 2.5th and 97.5th percentiles of the draws.

Section 4.6.1: Aggregation to the sepsis mortality envelope

We calculated the number of deaths attributable to each infectious syndrome in 2019 by multiplying our predictions of cause-, age group-, sex-, year-, and location-specific infectious syndrome fractions by our sepsis-mortality estimates from the first pathway. All infectious syndrome fractions were squeezed to sum to one prior to multiplication in order to ensure that we did not exceed the sepsis mortality envelope.

Finally, we aggregated our results to arrive at regional and global sepsis-related mortality by infectious syndrome.

Section 4.6.2: Infectious syndromes using GBD 2019 results

Out of the 12 explicit Level 1 infectious syndromes included in our hierarchy, we excluded (i) tuberculosis (TB), (ii) typhoid, paratyphoid, and invasive non-typhoidal Salmonella, and (iii) gonorrhoea and chlamydia from our binomial mixed-effects linear regression model. Instead, we used the published results from GBD 2019[2] for these causes of death, as we believe the GBD 2019 estimates fully represent these infectious syndromes because they are usually not intermediate causes of death.

Section 4.7: Model validation

Infectious syndrome modelling aims to predict which cases of infection belong to a specific infectious syndrome, which is a multi-class classification problem. We therefore use the Area Under the Receiver Operating Characteristics (ROC) Curve (AUC) to evaluate model performance. The ROC Curve is determined by the sensitivity (or true positive rate) and the specificity (or false positive rate) of the model, and a higher AUC score indicates that the model is capable of discerning between the different categories. Accuracy is a related measure which considers the proportion of true positives and true negatives predicted by the model with respect to the total number of predictions.

We used 5-fold cross validation to assess out-of-sample model performance. This validation withheld 20% of the sample from the analysis and measured predictive performance on the withheld data. This procedure was repeated 5 times such that each data point was included in exactly one set of withheld data. Table 4.7.1 reports the Accuracy and AUC score[24] for each of the age groups within the infectious syndrome models and table 4.7.2 reports the same metrics for the sepsis models. 99% of the models have an AUC score between 0.7 and 1, indicating an overall excellent performance of this modelling framework.

Table 4.7.1: Accuracy and AUC score for out-of-sample validation of infectious syndromes models

Model	Age group name	Accuracy	AUC score
Bacterial infections of the skin and subcutaneous systems	1 to 4	1.00	0.87
Bacterial infections of the skin and subcutaneous systems	10 to 14	0.99	0.87
Bacterial infections of the skin and subcutaneous systems	15 to 19	0.98	0.90
Bacterial infections of the skin and subcutaneous systems	20 to 24	0.99	0.93
Bacterial infections of the skin and subcutaneous systems	25 to 29	0.99	0.94
Bacterial infections of the skin and subcutaneous systems	30 to 34	0.99	0.95
Bacterial infections of the skin and subcutaneous systems	35 to 39	0.99	0.94
Bacterial infections of the skin and subcutaneous systems	40 to 44	0.98	0.94
Bacterial infections of the skin and subcutaneous systems	45 to 49	0.98	0.93
Bacterial infections of the skin and subcutaneous systems	5 to 9	0.99	0.87
Bacterial infections of the skin and subcutaneous systems	50 to 54	0.98	0.92
Bacterial infections of the skin and subcutaneous systems	55 to 59	0.97	0.92
Bacterial infections of the skin and subcutaneous systems	60 to 64	0.97	0.92
Bacterial infections of the skin and subcutaneous systems	65 to 69	0.97	0.92
Bacterial infections of the skin and subcutaneous systems	70 to 74	0.98	0.92
Bacterial infections of the skin and subcutaneous systems	75 to 79	0.98	0.93
Bacterial infections of the skin and subcutaneous systems	80 to 84	0.98	0.94
Bacterial infections of the skin and subcutaneous systems	85 to 89	0.98	0.95
Bacterial infections of the skin and subcutaneous systems	90 to 94	0.98	0.96
Bacterial infections of the skin and subcutaneous systems	95 plus	0.98	0.97
Bacterial infections of the skin and subcutaneous systems	Early Neonatal	0.99	0.94
Bacterial infections of the skin and subcutaneous systems	Late Neonatal	0.99	0.98
Bacterial infections of the skin and subcutaneous systems	Post Neonatal	1.00	0.89
Bloodstream infections	1 to 4	0.91	0.95
Bloodstream infections	10 to 14	0.85	0.92
Bloodstream infections	15 to 19	0.84	0.91
Bloodstream infections	20 to 24	0.89	0.94

Bloodstream infections	25 to 29	0.92	0.94
Bloodstream infections	30 to 34	0.93	0.94
Bloodstream infections	35 to 39	0.92	0.93
Bloodstream infections	40 to 44	0.90	0.92
Bloodstream infections	45 to 49	0.89	0.90
Bloodstream infections	5 to 9	0.87	0.94
Bloodstream infections	50 to 54	0.88	0.89
Bloodstream infections	55 to 59	0.88	0.87
Bloodstream infections	60 to 64	0.88	0.87
Bloodstream infections	65 to 69	0.89	0.87
Bloodstream infections	70 to 74	0.90	0.88
Bloodstream infections	75 to 79	0.91	0.89
Bloodstream infections	80 to 84	0.92	0.91
Bloodstream infections	85 to 89	0.93	0.92
Bloodstream infections	90 to 94	0.94	0.93
Bloodstream infections	95 plus	0.94	0.95
Bloodstream infections	Early Neonatal	0.94	0.96
Bloodstream infections	Late Neonatal	0.95	0.96
Bloodstream infections	Post Neonatal	0.93	0.96
CAI lower respiratory infections and all related infections in the thorax	5 to 69	0.99	0.99
CAI lower respiratory infections and all related infections in the thorax	70+ years	0.99	1.00
CAI lower respiratory infections and all related infections in the thorax	Neonatal	0.95	0.96
CAI lower respiratory infections and all related infections in the thorax	Post Neonatal to 5	0.99	1.00
CAI urinary tract infections and pyelonephritis	0 to 39	1.00	1.00
CAI urinary tract infections and pyelonephritis	40 plus	1.00	1.00
Diarrhoea	1 to 4	0.99	1.00
Diarrhoea	10 to 14	0.99	0.99
Diarrhoea	15 to 19	0.99	0.99
Diarrhoea	20 to 24	0.99	1.00
Diarrhoea	25 to 29	0.99	1.00
Diarrhoea	30 to 34	0.99	1.00
Diarrhoea	35 to 39	0.99	1.00
Diarrhoea	40 to 44	0.99	0.99
Diarrhoea	45 to 49	0.99	0.99
Diarrhoea	5 to 9	0.99	0.99
Diarrhoea	50 to 54	0.99	0.98
Diarrhoea	55 to 59	0.99	0.97
Diarrhoea	60 to 64	0.99	0.97
Diarrhoea	65 to 69	0.99	0.96
Diarrhoea	70 to 74	0.99	0.96
Diarrhoea	75 to 79	0.99	0.97
Diarrhoea	80 to 84	0.99	0.97
Diarrhoea	85 to 89	0.99	0.98
Diarrhoea	90 to 94	0.99	0.98

Diarrhoea	95 plus	0.99	0.99
Diarrhoea	Early Neonatal	1.00	1.00
Diarrhoea	Late Neonatal	1.00	1.00
Diarrhoea	Post Neonatal	0.98	0.99
Endocarditis and other cardiac infections	1 to 4	0.99	0.94
Endocarditis and other cardiac infections	10 to 14	0.99	0.97
Endocarditis and other cardiac infections	15 to 19	0.99	0.96
Endocarditis and other cardiac infections	20 to 24	0.99	0.96
Endocarditis and other cardiac infections	25 to 29	0.99	0.97
Endocarditis and other cardiac infections	30 to 34	0.99	0.97
Endocarditis and other cardiac infections	35 to 39	0.99	0.97
Endocarditis and other cardiac infections	40 to 44	0.99	0.97
Endocarditis and other cardiac infections	45 to 49	0.99	0.96
Endocarditis and other cardiac infections	5 to 9	0.99	0.95
Endocarditis and other cardiac infections	50 to 54	0.99	0.96
Endocarditis and other cardiac infections	55 to 59	0.99	0.95
Endocarditis and other cardiac infections	60 to 64	0.99	0.95
Endocarditis and other cardiac infections	65 to 69	0.99	0.95
Endocarditis and other cardiac infections	70 to 74	0.99	0.96
Endocarditis and other cardiac infections	75 to 79	0.99	0.96
Endocarditis and other cardiac infections	80 to 84	0.99	0.97
Endocarditis and other cardiac infections	85 to 89	0.99	0.98
Endocarditis and other cardiac infections	90 to 94	0.99	0.98
Endocarditis and other cardiac infections	95 plus	0.99	0.98
Endocarditis and other cardiac infections	Early Neonatal	0.99	0.98
Endocarditis and other cardiac infections	Late Neonatal	0.99	0.98
Endocarditis and other cardiac infections	Post Neonatal	0.99	0.89
HAI lower respiratory infections and all related infections in the thorax	5 to 69	0.96	0.89
HAI lower respiratory infections and all related infections in the thorax	70+ years	0.96	0.89
HAI lower respiratory infections and all related infections in the thorax	Neonatal	0.99	0.50
HAI lower respiratory infections and all related infections in the thorax	Post Neonatal to 5	0.97	0.94
HAI urinary tract infections and pyelonephritis	0 to 39	0.99	0.77
HAI urinary tract infections and pyelonephritis	40 plus	0.99	0.86
Infections of bone, joints, and related organs	0 to 9	0.99	0.94
Infections of bone, joints, and related organs	10 to 14	0.99	0.95
Infections of bone, joints, and related organs	15 to 19	0.99	0.88
Infections of bone, joints, and related organs	20 to 24	0.99	0.82
Infections of bone, joints, and related organs	25 to 29	0.99	0.85
Infections of bone, joints, and related organs	30 to 34	0.99	0.83
Infections of bone, joints, and related organs	35 to 39	0.99	0.85
Infections of bone, joints, and related organs	40 to 44	0.99	0.84
Infections of bone, joints, and related organs	45 to 49	0.99	0.84
Infections of bone, joints, and related organs	50 to 54	0.99	0.88
Infections of bone, joints, and related organs	55 to 59	0.99	0.89

Infections of bone, joints, and related organs	60 to 64	0.99	0.90
Infections of bone, joints, and related organs	65 to 69	0.99	0.90
Infections of bone, joints, and related organs	70 to 74	0.99	0.91
Infections of bone, joints, and related organs	75 to 79	0.99	0.92
Infections of bone, joints, and related organs	80 to 84	0.99	0.93
Infections of bone, joints, and related organs	85 to 89	0.99	0.94
Infections of bone, joints, and related organs	90 to 94	0.99	0.94
Infections of bone, joints, and related organs	95 plus	0.99	0.95
Meningitis and other bacterial central nervous system infections	1 to 4	0.98	0.98
Meningitis and other bacterial central nervous system infections	10 to 14	0.97	0.98
Meningitis and other bacterial central nervous system infections	15 to 19	0.98	0.97
Meningitis and other bacterial central nervous system infections	20 to 24	0.99	0.97
Meningitis and other bacterial central nervous system infections	25 to 29	0.99	0.98
Meningitis and other bacterial central nervous system infections	30 to 34	0.99	0.98
Meningitis and other bacterial central nervous system infections	35 to 39	0.99	0.97
Meningitis and other bacterial central nervous system infections	40 to 44	0.99	0.96
Meningitis and other bacterial central nervous system infections	45 to 49	0.99	0.96
Meningitis and other bacterial central nervous system infections	5 to 9	0.97	0.97
Meningitis and other bacterial central nervous system infections	50 to 54	0.99	0.94
Meningitis and other bacterial central nervous system infections	55 to 59	0.99	0.93
Meningitis and other bacterial central nervous system infections	60 to 64	0.99	0.93
Meningitis and other bacterial central nervous system infections	65 to 69	0.99	0.92
Meningitis and other bacterial central nervous system infections	70 to 74	0.99	0.92
Meningitis and other bacterial central nervous system infections	75 to 79	0.99	0.91
Meningitis and other bacterial central nervous system infections	80 to 84	0.99	0.92
Meningitis and other bacterial central nervous system infections	85 to 89	0.99	0.92
Meningitis and other bacterial central nervous system infections	90 to 94	0.99	0.93
Meningitis and other bacterial central nervous system infections	95 plus	0.99	0.92
Meningitis and other bacterial central nervous system infections	Early Neonatal	0.99	0.99
Meningitis and other bacterial central nervous system infections	Late Neonatal	1.00	0.99
Meningitis and other bacterial central nervous system infections	Post Neonatal	0.99	0.98
Peritoneal and intra-abdominal infections	1 to 4	0.99	0.97
Peritoneal and intra-abdominal infections	10 to 14	0.97	0.96
Peritoneal and intra-abdominal infections	15 to 19	0.96	0.96
Peritoneal and intra-abdominal infections	20 to 24	0.97	0.97
Peritoneal and intra-abdominal infections	25 to 29	0.98	0.98
Peritoneal and intra-abdominal infections	30 to 34	0.98	0.98
Peritoneal and intra-abdominal infections	35 to 39	0.98	0.98
Peritoneal and intra-abdominal infections	40 to 44	0.97	0.97
Peritoneal and intra-abdominal infections	45 to 49	0.97	0.96
Peritoneal and intra-abdominal infections	5 to 9	0.98	0.97
Peritoneal and intra-abdominal infections	50 to 54	0.96	0.95
Peritoneal and intra-abdominal infections	55 to 59	0.96	0.95
Peritoneal and intra-abdominal infections	60 to 64	0.96	0.95

Peritoneal and intra-abdominal infections	65 to 69	0.96	0.95
Peritoneal and intra-abdominal infections	70 to 74	0.97	0.96
Peritoneal and intra-abdominal infections	75 to 79	0.97	0.97
Peritoneal and intra-abdominal infections	80 to 84	0.98	0.98
Peritoneal and intra-abdominal infections	85 to 89	0.98	0.98
Peritoneal and intra-abdominal infections	90 to 94	0.98	0.98
Peritoneal and intra-abdominal infections	95 plus	0.99	0.98
Peritoneal and intra-abdominal infections	Early Neonatal	0.99	0.97
Peritoneal and intra-abdominal infections	Late Neonatal	0.99	0.97
Peritoneal and intra-abdominal infections	Post Neonatal	0.98	0.94

Table 4.7.2: Accuracy and AUC score for out-of-sample validation of sepsis models

Model	Age group name	Accuracy	AUC score
Sepsis	1 to 4	0.89	0.93
Sepsis	10 to 14	0.90	0.92
Sepsis	15 to 19	0.95	0.94
Sepsis	20 to 24	0.95	0.94
Sepsis	25 to 29	0.94	0.95
Sepsis	30 to 34	0.93	0.94
Sepsis	35 to 39	0.93	0.93
Sepsis	40 to 44	0.93	0.91
Sepsis	45 to 49	0.93	0.88
Sepsis	5 to 9	0.89	0.92
Sepsis	50 to 54	0.93	0.86
Sepsis	55 to 59	0.94	0.84
Sepsis	60 to 64	0.94	0.83
Sepsis	65 to 69	0.94	0.83
Sepsis	70 to 74	0.95	0.84
Sepsis	75 to 79	0.95	0.85
Sepsis	80 to 84	0.96	0.87
Sepsis	85 to 89	0.96	0.88
Sepsis	90 to 94	0.96	0.90
Sepsis	95 plus	0.96	0.92
Sepsis	Early Neonatal	0.91	0.87
Sepsis	Late Neonatal	0.87	0.88
Sepsis	Post Neonatal	0.88	0.89

Section 5: Case fatality ratios

Section 5.1: Input data

Case fatality ratios (CFRs) were modelled for the pathogens and infectious syndromes of interest using all available data detailing the organism responsible for infection, the infectious syndrome, and patient outcome. This included hospital and microbial data, totalling 19.7 million isolates and cases.

We additionally included 52 907 cases from literature sources for CNS infections, which had been previously extracted for a systematic review in GBD.

Section 5.2: Data Processing

All input data sources were processed as described in sections 6.2.1–6.2.4 and section 6.2.7 and pathogens of interest were chosen as described in section 6.2.5. Input data for the CFR models were aggregated based on data source, year, GBD location, and age group (as well as hospital/community acquired status, in the case of the lower respiratory and urogenital infectious models). For lower respiratory and blood stream infections, for which CFRs could be vastly different in neonates, we modelled the following age groups: neonatal, post-neonatal–5 years, 5–50 years, 50–70 years, and 70 years and older. For all other infectious syndromes, we modelled the following age groups: neonatal–5 years, 5–50 years, 50–70 years, and 70 years and older. We excluded from the analysis any source-location-year-age with fewer than five cases and zero deaths.

To allow us to implement our Bayesian, mixed-effect linear modelling tool (described below), CFRs were logit-transformed. We used the delta method to compute the standard error of CFRs in logit space. To incorporate data with zero deaths, or with an equal number of deaths and cases, we applied a 1% offset, such that the CFRs for data with zero deaths was represented as 1% and the CFR for data with an equal number of deaths and cases was represented as 99%.

Section 5.3: Modelling overview

Pathogen-specific CFRs were modelled separately by infectious syndrome and were calculated as a function of HAQ Index and age. We used the HAQ Index to extrapolate CFRs determined from the input data, which often had a broad but not comprehensive geographic scope, to all 204 GBD countries and territories. To account for heterogeneity across the sources of input data, we implemented a mixed-effects meta-regression framework, modelling data source as a random effect. We further incorporated a binary fixed-effect denoting whether the data source only included intensive care unit (ICU) patients, for which CFRs were expected to be higher.

The pathogens of interest for each infectious syndrome were determined by prevalence in the data and expert opinion, with the goal of modelling approximately 90% of specified-pathogens associated with each infectious syndrome (see section 6.2.5). Because each data source generally reported only a set of the pathogens we evaluated in our research, the input data for the pathogens varied in geographic coverage; nearly all pathogens were well reported in high-income areas, but some pathogens were not well represented in the smaller subset of data we collected from low- and middle-income locations.

For those pathogens with ‘rich’ data, defined by our method as having at least ten high-quality data points below a moderate HAQ Index (0.7), we modelled a unique effect of HAQ Index, achieved by interacting the HAQ Index fixed-effect with the pathogen-specific fixed-effect. This process, referred to from here on as the ‘interaction model,’ allowed the relative deadliness of pathogens to vary depending on a location’s HAQ Index. For those pathogens with fewer than 10 high quality data points below 0.7 HAQ Index, or those whose results in the interaction models indicated an unrealistically large influence of HAQ Index (eg, 70% CFR in low HAQ Index countries, 1% CFR in high HAQ Index countries), we modelled a pathogen-specific intercept with an HAQ Index fixed-effect shared across the pathogens. As a consequence of the single fixed-effect on HAQ Index, a pathogen that was predicted to be the deadliest in low HAQ Index countries would also be predicted to be the deadliest in high HAQ Index countries in these ‘intercept models.’ To estimate the CFRs for other known bacteria, which either were not selected as a pathogen of interest or lacked sufficient data for inclusion in the intercept models, we pooled all bacterial data together and estimated a single

CFR curve from age, HAQ Index, and the data source heterogeneity covariates. Thus, up to three models were run for each infectious syndrome:

1) an interaction model including data for all data rich pathogens and 'other specified bacteria' (which was included to inform the overall influence of HAQ Index on CFR, predictions were only generated for the data rich pathogens),
2) an intercept model including data for data rich and data sparse pathogens, as well as 'other specified bacteria' (predictions were only generated for the data sparse pathogens), and
3) an 'other bacteria' model that included data for all bacterial pathogens (predictions were generated by HAQ Index and age, without any pathogen specific term).

Details of the CFR modelling framework used to assess the pathogens for each infectious syndrome have been published previously.[1] Whenever needed, the CFR for any bacterial pathogen "not explicitly modelled" was estimated using the 'other bacteria' model for subsequent steps of our modelling processes.

For some infectious syndromes, the relative deadliness of a pathogen may be strongly determined by either the age of the patient or whether the infection was community- or hospital-acquired. For bloodstream infections, we ran two distinct sets of CFR models, one for neonates (0-27 days) and another for post neonates, to capture the differing dynamics of pathogen deadliness in these two populations. As is done for our other modelling processes, we also separate community-acquired and hospital-acquired cases in our CFR models for lower respiratory and urogenital infections. Because some data sources did not provide enough information to infer whether an infection was community- or hospital-acquired, but still included important information on the relative pathogenesis and the difference in CFRs across varying HAQ indices, infections of unknown origin were included in both the community-acquired and hospital-acquired models for these two syndromes. Any bias in these 'unknown origin' infections was adjusted for using a binary fixed-effect representing an 'unknown origin' infection, and predictions were generated for the community- and hospital-acquired infections only.

Section 5.4 Modelling framework

The data were analysed using a meta-analytic mixed effects structure. The main model can be specified as follows:

$$logit(y_i) = X_i\beta + u_i 1 + \epsilon_i, \qquad \epsilon_i \sim N(0, \Sigma_i), \quad u_i \sim N(0, \gamma) \qquad (5.4.1)$$

where

- y_i contains CFRs for data source i
- Design matrix X_i contains as columns the following covariates
  - in all models:
    - HAQ Index
    - dummy-coded indicator for age group
    - dummy-coded ICU indicator for data source (1 if data source only compiles information on ICU patients, 0 if a mix between ICU/non-ICU patients)
  - in 'interaction' and 'intercept' models:
    - dummy-coded indicator for pathogen
  - in 'interaction' models only:
    - interaction between pathogen and HAQ Index (product of dummy-coded pathogen columns and HAQ Index)

- in models evaluating community/hospital acquired infection (LRI+, UTI):
  - dummy-coded variable indicating source of infection (1 if unknown source, 0 if community OR hospital acquired, depending on whether the model is evaluating community or hospital infections)
- β are fixed effect multipliers
- ϵ_i are observation error terms with known variances
- u_i are data source-specific random intercepts with unknown covariance γ

The underlying program used to fit the model (meta-regression, Bayesian, regularised, trimmed [MR-BRT]) is described elsewhere.[25] The program allows specification of priors on γ and β.

- Prior on γ, data source random effect: Many input data-sources cover only a single country, leading to low variability in HAQ Index within each data-source. Such collinearity adversely influenced the accuracy of the estimated effect of HAQ Index, which was instrumental in extrapolating trends from the input data to global results. To emphasise the contribution of HAQ Index over data-source in the modelled estimates, we implemented a strong Gaussian prior (mean 0, standard deviation 0.001) on γ.
- Prior on β for HAQ Index: There were a handful of cases in which the estimated effect of HAQ Index on CFRs given our data was clinically implausible. For skin and neonatal bloodstream infections, we had very limited data from low HAQ Index locations, with available data indicating a very intense influence of HAQ Index. Initial model results for these syndromes indicated more than 10-fold higher CFRs in low HAQ Index countries relative to high HAQ Index countries. To attenuate the effect of HAQ Index in these models we implemented a weakly informative Gaussian prior on the HAQ Index β with mean 0 and standard deviation 0.2.

  Similarly, the peritoneal and intra-abdominal infection CFR models did not have enough input data from low HAQ Index countries to estimate a sensible HAQ Index-CFR trend; initial models indicated very strong positive associations between CFR and HAQ Index such that the CFR in low HAQ Index countries were nearly zero. To amend this, we implemented an informative Gaussian prior on the HAQ Index β with mean equal to the coefficient estimate for HAQ Index in the adult BSI models. The standard deviation for this prior was 0.2.

  For the urogenital infection models (which were ran separately for community- and hospital-acquired infections) and those for hospital-acquired lower respiratory infections, there was substantial collinearity between HAQ Index and the indicator variable for infections of unknown origin; data that did not indicate the origin of infection were generally sourced from countries with much lower HAQ Indices and much higher baseline CFRs. To emphasise the attribution of this effect to HAQ Index, rather than 'unknown infection origin,' we implemented an informative Gaussian prior on the HAQ Index β. The mean of this prior was centered at a value estimated for the coefficient of HAQ Index on CFR from weighted simple linear regression, with the weights equal to the inverse of the standard error of the CFRs. The standard deviationfor this prior was 0.2.

Table 5.4.1: Number of data points and parameters estimated in each case fatality ratio model

Infectious syndrome	Sub-model	CFR model type	Data points (source-location-years)	Estimated parameters
CNS	-	Interaction	2094	13
CNS	-	Intercept	2756	15
CNS	-	Other	2756	7
Intra-abdominal	-	Intercept	639	12
Intra-abdominal	-	Other	639	6
LRI+	Community-acquired	Intercept	10485	23
LRI+	Community-acquired	Other	10485	8
LRI+	Hospital-acquired	Intercept	10122	23
LRI+	Hospital-acquired	Other	10122	8
Skin	-	Intercept	1866	15
Skin	-	Other	1897	6
Bone+	-	Intercept	432	12
Bone+	-	Other	432	5
UTI	Community-acquired	Intercept	1596	20
UTI	Community-acquired	Other	1596	7
UTI	Hospital-acquired	Intercept	1844	20
UTI	Hospital-acquired	Other	1844	7
BSI	Neonatal	Intercept	1413	18
BSI	Neonatal	Other	1271	3
BSI	Non-neonatal	Interaction	7468	24
BSI	Non-neonatal	Intercept	10842	25
BSI	Non-neonatal	Other	10842	6
Diarrhoea	-	Intercept	4041	14
Diarrhoea	-	Other	3525	6

BSI = Bloodstream infections. CNS = Meningitis and other bacterial central nervous system infections. LRI+ = Lower respiratory infections and all related infections in the thorax. Intra-abdominal = Peritoneal and intra-abdominal infections. Skin = Bacterial infections of the skin and subcutaneous systems. UTI = Urinary tract infections and pyelonephritis. Bone+ = Infections of bones, joints, and related organs.

Section 5.5 Predictions and uncertainty

Predictions for 2019 CFRs were generated for each country, age group, and pathogen as a function of each country's HAQ Index, assuming mixed ICU/non-ICU patients and, in the case of models for UTI and LRI+, that the infection was community- or hospital-acquired (in contrast to infections of unknown origin). For pathogens with insufficient data to estimate a syndrome-specific CFR, we predicted out using the 'other bacteria' CFR associated with the infectious syndrome. Importantly, all of the CFRs we calculate by infectious syndrome are independent of that syndrome's underlying cause.

Uncertainty estimates were generated using asymptotic uncertainty intervals. Specifically, for the model, the posterior uncertainty for the coefficients β is Gaussian, with mean and variance given below:

$$\hat{\beta} = \left(\sum_i X_i^T V_i^{-1} X_i\right)^{-1} \left(\sum_i X_i^T V_i^{-1} y_i\right) \qquad (5.5.1)$$

$$Var(\hat{\beta}) = \left(\sum_i X_i^T V_i^{-1} X_i\right)^{-1} \qquad (5.5.2)$$

where

$$V_i = 11^T + \hat{\gamma} I \qquad (5.5.2)$$

The variance-covariance matrix was used to obtain 1000 draws for the coefficients, which are then used to get intervals for the predictions.

Section 6: Pathogen distribution

Section 6.1: Input data

With this model, we aimed to estimate the distribution of pathogens causing each infectious syndrome. To get input data for this model, we gathered all available data sources described in section 2 that meet the following criteria:

- Sufficient diagnosis (for patient- or admission-level datasets) or sample specimen type (for isolate- or culture-level datasets) information for us to determine the infectious syndrome
- Information on which pathogen(s) caused the infection or which pathogen(s) were detected in an infectious sample, as determined through culture or genomic-based methods
- Did not have a strongly biased sampling framework across pathogens (for example, did not deliberately sample until 100 cases of every pathogen of interest had been obtained)

The input data source types that met these criteria were:

- Multiple causes of death data
- Hospital discharge
- Linkage data
- Microbial data with and without outcome information
- Literature studies from the aetiology literature reviews
- Mortality surveillance (Child Health and Mortality Prevention Surveillance [CHAMPS])

From these sources combined, there was a total of 343 million isolates and cases. Table S1 provides a detailed breakdown of this total.

Section 6.2: Data processing

Section 6.2.1: Extraction and standardisation

We extracted and standardised the location, year, age, sex, diagnoses, specimen type, pathogens, and hospital- and community-acquired (HAI and CAI) status of each record in every dataset. HAI or CAI status in microbial data was determined as described in section 2.3, while in MCoD, hospital discharge, and linkage data, a record was considered CAI if the infectious syndrome was the primary or underlying diagnosis and HAI otherwise, as described in section 4. These datasets report a variety of metrics, including deaths, admissions, cases, cultures, and isolates. While these metrics are not completely comparable (for example, a single patient may often have multiple cultures taken during a single hospital admission), we chose to standardise them into two categories: “deaths,” for any unit associated with an outcome of death, and “cases,” for any unit regardless of outcome. We assigned a unique identifier, sample ID, to track each unique unit of analysis whenever a dataset included enough line-level data to make this possible. We did not track the relationship between sample ID and patient or admission, in many cases because this was not possible; an improvement to future analyses may be to track this information and account for multiple isolates or cultures from a single admission. The majority of the data informing culprit pathogen were from microbiological analysis of various isolates, but we also considered antigen testing, such as the urinary strep antigen, and polymerase chain reaction (PCR)-based testing when assigning the pathogen responsible for infection.

Section 6.2.2: Assigning infectious syndrome

After standardising the data, we mapped every sample ID or tabulated figure in the data to infectious syndrome based on its diagnoses and specimen type. Infectious syndrome was assigned first based on any diagnosis associated with a given sample ID or tabulated figure. For samples IDs or tabulated figures with multiple diagnoses and/or an underlying diagnosis, we followed the rules laid out in section 4 for assigning infectious syndrome based on multiple causes. If a dataset contained no diagnoses or the diagnoses provided no information on infectious syndrome, we assigned infectious syndrome based on specimen type (table 6.2.2.1). This is an imprecise method because a patient may have a sample taken from an organ system that is not the site of their primary infection, most commonly from the blood. Finally, if neither diagnosis nor specimen information provided information on infectious syndrome, we assigned infectious syndrome based on pathogen for a select number of pathogens (table 6.2.2.2).

Table 6.2.2.1: Syndrome assignment based on standardised specimen types

Standard specimen	Assigned to syndrome
Blood	Bloodstream infections
Bone & joint	Infections of bones, joints, and related organs
Catheter	Bacterial infections of the skin and subcutaneous systems
Cerebrospinal fluid	Meningitis and other bacterial central nervous system infections
Gastrointestinal tract & bowel	Diarrhoea
Urinary tract infection	Urinary tract infections and pyelonephritis
Intra-abdominal	Peritoneal and intra-abdominal infections
Rectal/stool	Diarrhoea
Lower respiratory	Lower respiratory infections and all related infections in the thorax
Skin	Bacterial infections of the skin and subcutaneous systems
Upper respiratory	Other infections
Urogenital	Other infections
Other and unspecified specimens	No infectious syndrome

Table 6.2.2.2: Syndrome assignment based on pathogen for entries lacking diagnostic and specimen information

Pathogen	Assigned to syndrome
Salmonella Typhi	Typhoid, paratyphoid, and invasive non-typhoidal Salmonella
Salmonella Paratyphi	Typhoid, paratyphoid, and invasive non-typhoidal Salmonella
Salmonella Typhi or Paratyphi	Typhoid, paratyphoid, and invasive non-typhoidal Salmonella
Non-typhoidal *Salmonella* species	Typhoid, paratyphoid, and invasive non-typhoidal Salmonella
Mycobacterium tuberculosis	Tuberculosis
Neisseria meningitidis	Central nervous system infections
Neisseria gonorrhoeae	Gonorrhoea and chlamydia

Section 6.2.3: Contaminants and no aetiology detected

Some pathogens cause disease so rarely or are so commonly contaminants that we considered them to be contaminants, unlikely to be the true cause of disease. Examples include many *Corynebacterium* species and *Staphylococcus epidermidis*. We dropped all such contaminants from the analysis, as well as any record listed by treating clinicians in the data as a contaminant. Contaminants are identified at the most-detailed species or serotype level reported in the data; thus, in the broad pathogen categories that are eventually modelled, like fungi in LRI+, specific contaminant species have already been removed.

We also dropped from the analysis all records where no pathogen was detected, or the patient diagnosis indicated an unspecified bacterium. This assumes that the distribution of pathogens among cases with known aetiology are the same as those with unknown aetiology; in other words, that the probability of detection is the same for every pathogen. This assumption may break down if certain pathogens are more difficult to detect than others, or in cases where a pathogen is irregularly tested for within a laboratory.

Section 6.2.4: Polymicrobial infections

A single infection may be caused by multiple bacteria, and the co-occurrence of several bacteria can have significant effects on the treatment and outcome of disease. Some of our line-level data sources report multiple pathogens per individual record, allowing us to quantify the extent of polymicrobial infection. Other data sources tabulate over pathogen with no linking clinical information, thereby masking this information, or do not report the co-occurrence of additional bacteria.

For data sources where multiple pathogens were listed per sample ID, we classified these cases according to the following criteria. First, if a case contained more than one of “unspecified bacteria,” “virus,” “fungus,” and another pathogen(s), we chose to drop all these pathogens except the one(s) most likely to be responsible for disease, with the following ranking from most to least likely:

1. Another pathogen(s)
2. Unspecified bacteria
3. Virus
4. Fungus

For example, for a sample ID with pathogens *Escherichia coli, Acinetobacter baumannii*, and a virus, we would drop the virus and retain both the *E. coli* and *A. baumannii*. After applying this drop, we considered any sample ID that contained more than one pathogen to be polymicrobial and polymicrobial was treated as a distinct pathogen category in all further analysis. This scheme was designed to best interrogate the contribution of bacterial disease and thus certain co-occurrence profiles, like a viral infection co-occurring with a fungal infection, were considered to be uninformative. Futures studies will seek to better understand the interplay of a more varied set of polymicrobial co-infections.

By standardising all datasets that report polymicrobial infections into distinct mono-pathogen and poly-pathogen categories, we created an inconsistency between these datasets and datasets that do not report the co-occurrence of pathogens. For example, a dataset that reports the co-occurrence of *E. coli* and *A. baumannii* would be standardised into three groups, mono-*E.coli*, mono-*A. baumannii*, and co-occurring *E. coli* and *A. baumannii*, while a dataset that reports *E. coli* and *A. baumannii* separately would have two categories that both have some unknown overlap. In order to allow us to use both data types, we chose to assume that the relative prevalences of pathogens in datasets that

do not report co-occurrence would be comparable to their mono-pathogenic counterparts in datasets that do report co-occurrence. This assumes that the co-occurrence of pathogens is random and is not correlated for certain pathogens. We did not have sufficient data to fully test the validity of this assumption, given that few datasets report the full universe of pathogens which may co-occur.

Section 6.2.5: Selecting pathogens for estimation

For each infectious syndrome, we selected roughly 10–20 pathogens to estimate explicitly in the pathogen distribution based on the following criteria:

- The prevalence of each pathogen in the raw data
- Clinical knowledge about the primary aetiologies of each infectious syndrome
- The amount of available data, which limits the number of pathogens that can be estimated successfully

In addition to the n pathogens for a given syndrome that we estimate explicitly, we also included an "other specified pathogens" category for every infectious syndrome, to which we mapped all other aetiologies identified in the data. Thus, the set of estimated pathogens for each infectious syndrome is mutually exclusive and collectively exhaustive of all possible aetiologies. Polymicrobial infections were either estimated explicitly or included in the "other" category, making all explicitly estimated individual pathogens mono-pathogenic. In addition to these criteria, we also considered the following factors:

- Components of this work were motivated by an analysis on the burden of antimicrobial resistance in bacteria; we therefore erred on the side of estimating bacteria with strong evidence of AMR, rather than bacteria with low evidence of AMR or non-bacterial aetiologies. Estimates for diarrhoeal pathogens were sourced from affiliated research on the GBD and selected pathogens using a different procedure. Please refer to Section 6.4.2 for more details.
- Clinically relevant aetiologies differ from syndrome to syndrome, and we were unable to estimate all pathogens explicitly in every syndrome due to a lack of data. Therefore, the "other" pathogen category is composed of slightly different pathogens for every infectious syndrome, and can occasionally contain pathogens that are explicitly estimated for another infectious syndrome.
- We sought to include enough explicitly estimated pathogens to ensure that the "other" category remained below 10% for all infectious syndromes.

For a list of pathogens covered in each infectious syndrome model, please refer to table 6.3.2.

Section 6.2.6: Estimating unbiased other and polymicrobial categories

One of the central challenges of estimating pathogen distributions was that not every data source tested for or reported every possible aetiology of a given infectious syndrome. For example, many literature studies on the aetiologies of meningitis only report on bacterial aetiologies. Some surveillance systems, like the US Centers for Disease Control and Prevention (CDC) Active Bacterial Core surveillance (ABCs), only collect data on certain pathogens of interest. Only certain pathogens are referenced explicitly in the International Classification of Diseases (ICD), limiting which pathogens can be identified from ICD-based data types like MCoD and hospital discharge. Finally, some datasets reported only a subset of the pathogens that we are interested in for a given infectious syndrome, reporting the remaining aetiologies in an aggregate "other" category. These practices have led to inconsistencies in the "other" and "polymicrobial" categories across data

sources. Datasets can either over or under-report "other," and datasets that report fewer specific pathogens will automatically report fewer polymicrobial infections.

To address this problem, we maintained a list of data sources that we believe have sufficient testing and reporting to give unbiased estimates of "other" and "polymicrobial" for all syndromes. We dropped any data on "polymicrobial" or "other" that did not come from these data sources. These data sources all had a complete sampling framework (e.g., they do not limit the scope of aetologies that they test for) and reported their results without any deliberate aggregation. While we believe this list provided an accurate starting place for the estimation of "other" and "polymicrobial," future work to improve this method would involve a more detailed analysis of sampling framework and reporting categories in each dataset, specific to each infectious syndrome.

There were two major exceptions to this method for handling "other specified pathogens." First, determining the pathogenic aetiology of LRI with microbiology represents challenges that have been well described previously.[26,27] In order to account for this limitation, we utilised a vaccine probe design to inform the *Streptococcus pneumoniae* cause fraction of LRI, consistent with the approach used in the GBD aetiology estimation process.[28] In brief, we extracted the vaccine efficacy of the pneumococcal vaccine against all pneumonia from 18 vaccine probe studies with randomised-control trial, before-after, and cohort designs among children and adults. We then calculated the PAF of pneumonia due to *S. pneumoniae* in each study ($Strep\ Base\ PAF$) based on these vaccine efficacies ($VE_{all\ pnuemonia}$), the vaccine efficacy of pneumococcal vaccine against vaccine-type pneumococcal pneumonia as pooled from three studies (two in children and one in adults) (VE_{vtpp}), the percentage of the population covered by the pneumococcal vaccine as modelled in GBD (100% for RCTs) (Cov_{PCV3}),[29] and the percent of serotypes covered by the vaccine[30] ($Cov_{serotype}$) (equation 6.2.6.1). We modelled a global age-specific PAF for *S. pneumoniae* based on these data in the MR-BRT environment and finally adjusted this PAF based on the vaccine coverage in children in every GBD location in 2019 and optimal vaccine efficacy in children ($Strep\ Final\ PAF$) (equation 6.2.6.2). In adults (age 5+), we assumed the effects of vaccination on adults would be primarily indirect from vaccination in children, and included an adjustment factor on the vaccine efficacy to account for this, derived from Grijalva et al.[31]

$$Strep\ Base\ PAF = \frac{VE_{all\ pneumonia}}{VE_{vtpp}Cov_{PCV3}Cov_{serotype}} \qquad (6.2.6.1)$$

$$Strep\ Final\ PAF = \frac{Strep\ Base\ PAF\left(1 - Cov_{PCV3}Cov_{serotype}VE_{PCV3\ Optimal}\right)}{1 - (Strep\ Base\ PAF)Cov_{PCV3}Cov_{serotype}VE_{PCV3\ Optimal}} \qquad (6.2.6.2)$$

In this vaccine probe analysis, ($1 - Strep\ Final\ PAF$) is not consistent with the "other" category in our model, since it includes all non-*S. pneumoniae* aetiologies. We retained all of the data from the vaccine probe analysis as two categories, *S. pneumoniae* and "not *S. pneumoniae*" and addressed the inconsistencies between them and our other data using our modelling framework.

The second major exception involves several literature studies on the proportion of neonatal bacterial meningitis caused by *Streptococcus agalactiae* (Group B *Streptococcus;* GBS). We found that these literature studies were important to our estimation of the pathogen distribution of neonatal meningitis, which is distinct from other age groups because of its high proportion of GBS. However, these studies either only reported or were only extracted with two categories, GBS and

"other bacterial, not GBS." We retained both these categories and addressed the inconsistencies between them and our other data using our modelling framework.

Section 6.2.7: Age-sex splitting

We standardised age and sex across all datasets to the following most-detailed groups using the GBD causes of death age-sex splitting algorithm for age:[32] 0–6, 7–27, and 28–364 days, and 1–4, 5–9, 10–14, 15–19, 20–24, 25–29, 30–34, 35–39, 40–44, 45–49, 50–54, 55–59, 60–64, 65–69, 70–74, 75–79, 80–84, 85–89, 90–94, 95+ years; and sex: male and female. This algorithm is based on the assumption that age-sex pattern of the death or case rate for a given infectious syndrome or pathogen is inherent to the pathology of the disease and is therefore constant across location and year.

To apply the algorithm, we first calculated distinct age-sex weights for every infectious syndrome and pathogen, separately for deaths and cases. These weights are the aggregate death and case rates across all datasets that report every detailed age-sex group. If we were to use a dataset that only reported some of the detailed age-sex groups, then the unreported age-sex groups would be biased downwards in the weight distribution. Calculating rates based on raw data counts could lead to extremely low rates, since we are typically comparing the entire population of a given location-year to deaths or cases captured within a single study, hospital, or surveillance system. Since the age-sex splitting algorithm only relies on the relative distribution of the weights, however, rather than their absolute level, this bias ultimately had no effect. For any infectious syndrome or pathogen combination for which we did not have enough data to create plausible age-sex weights, we used a set of all-pathogen weights for that infectious syndrome instead.

Since we split cases and deaths independently, it is possible for a detailed age-sex group produced by the splitting algorithm to contain fewer cases than deaths. When this occurred, we capped the deaths to match the cases. For future improvement, a possible solution to this problem may be to split deaths, survivors, and cases without indication of outcome separately.

Section 6.2.8: Standardising measures

The input data sources reported a variety of combinations of measures, including some that reported deaths only, some that reported cases only, and some that reported both cases and deaths. In order to standardise these measures to cases, we estimated infectious syndrome- and pathogen-specific CFRs (see section 5) and used these CFRs to convert all deaths-only datasets to cases. For any infectious syndrome or pathogen combination for which we did not have enough data to estimate plausible CFRs, we used a set of all-bacteria CFRs for that infectious syndrome instead. All modelling was done in case space.

Several of our microbial databases came exclusively from ICUs and were therefore heavily biased towards severe illness. In order to mitigate this bias, we dropped all information on cases in ICU-only datasets and recalculated implied cases based on reported deaths and our CFRs. No similar adjustment was made to attempt to account for biases between hospitalised and un-hospitalised populations, although we did account for HAI versus CAI for two infectious syndromes—LRI and thorax infections and UTI—within our modelling framework.

Section 6.3: Modelling framework

Section 6.3.1: Overview

To model the distribution of pathogens for each infectious syndrome, we developed a method for the multinomial estimation of partial and compositional observations (MEPCO). We assumed that the aetiologies of a given infectious syndrome followed a multinomial distribution. Due to

inconsistencies in which pathogens are tested for and reported by different data sources, each data source contained partial observations of the possible outcomes of the underlying multinomial distribution. Certain data sources like the vaccine probe estimates and the GBS neonatal meningitis studies represent compositional observations, where pathogens like "not *S. pneumoniae"* and "other bacterial, not GBS" represent aggregates of more detailed pathogens.

In order to use both partial and compositional data, we constructed a network model with the dependent variable as the log ratio of cases between different pathogens and estimated over a flexible parameterisation of multinomial parameters using a maximum likelihood approach. Consider a given infectious syndrome with a multinomial distribution of n mutually exclusive, collectively exhaustive aetiologies with probabilities $p = (p_1, \dots, p_n)$, so that each $p_j \in (0,1)$ and $\sum_j p_j = 1$. The likelihood of an observation of $c = (c_1, \dots, c_n)$, where c_j = number of cases of pathogen j in a total sample of N infections ($\sum_j c_j = N$), is:

$$P(c|p) = N! \prod_{j=1}^{n} \frac{p_j^{c_j}}{c_j!} \qquad (6.3.1.1)$$

We modelled the probabilities using a composition of a link function with a linear predictor:

$$p_{i,j} = \exp\left(x_{i,j}^T \beta_j\right) \qquad (6.3.1.2)$$

for observations i, a vector of covariates $x_{i,j}$, and a vector of coefficientsβ_j for each pathogen j. Table 6.3.2 shows the covariates used for infectious syndrome model; a typical specification included an intercept term, HAQ Index, a categorical age group dummy for large age bins, and any relevant vaccine coverage proportions by country.

Aetiology probabilities were not directly observed. Rather, we observed ratios between sums of these probabilities, which reduce to ratios between sums of cases within each study. These observations therefore take the form:

$$y_i = \frac{cases\ of\ pathogen\ A}{cases\ of\ pathogen\ B} = \frac{\sum_{j=1}^{n} w_{i,j}^{a} \exp\left(x_{i,j}^T \beta_j\right)}{\sum_{j=1}^{n} w_{i,j}^{b} \exp\left(x_{i,j}^T \beta_j\right)} \qquad (6.3.1.3)$$

where $w_{i,j}^{a}$ is a weight of 0 or 1 that selects the mutually exclusive, collectively exhaustive most-detailed pathogens that make up observed pathogen A, which may be a composite observation. For example, for the "other bacterial, non-GBS" pathogen, $w_{i,j}$ would be 1 for *Staphyloccocus aureus, S. pneumoniae, Haemophilus influenzae, Neisseria meningitidis, Listeria monocytogenes, K. pneumoniae, E. coli*, and other pathogens and 0 for GBS and virus. We dropped all observations where either the numerator or denominator had 0 observed cases in order to make this calculation and a forthcoming log transform possible. This may bias the model towards overestimating less common pathogens.

It is not possible to infer all coefficients β_j from the observations, since they are all relative. However, if we fix all of the coefficients for one pathogen to 0 as a reference group, then we obtain a well-posed inverse problem, as long as there is enough data to estimate the remaining coefficients. Without loss of generality, we assumed $\beta_1 = 0$ for all elements and obtain estimates of the remaining $\beta_2, \dots, \beta_n$ by minimising the sum of the residuals between log-transformed observations y and corresponding log-transformed predictions from equation 6.3.1.3:

$$\min_{\beta_2,\dots,\beta_n} f(\beta) := \sum_i \frac{1}{\sigma_i^2}\left[\ln(y_i) - \ln\left(\sum_{j=1}^{n} w_{i,j}^{a}\, exp\left(x_{i,j}^T\beta_j\right)\right) + \ln\left(\sum_{j=1}^{n} w_{i,j}^{b}\, exp\left(x_{i,j}^T\beta_j\right)\right)\right]^2 \qquad (6.3.1.4)$$

where σ_i^2 are variances corresponding to the data points. Equation 6.3.1.4 is a nonlinear likelihood minimisation problem that that we optimised using a standard implementation of the Gauss-Newton method.[33] We then re-normalised the optimal coefficients to obtain final predictions of the probabilities of each pathogen:

$$p_{i,j} = \frac{\exp\left(x_{i,j}^T\beta_j\right)}{\sum_{\hat{j}} \exp\left(x_{i,\hat{j}}^T\beta_{\hat{j}}\right)} \qquad (6.3.1.5)$$

To quantify the uncertainty of this estimate, we used the Fisher information matrix to obtain the posterior distribution of $(\beta_2, \dots, \beta_n)$. The Fisher information is a simple and efficient technique, widely used in both frequentist and Bayesian analysis.[34] The Fisher information matrix for all pathogens except for the reference was obtained by inverting the Gauss-Newton Hessian approximation at the final iterate. This technique gave us a posterior distribution that allowed us to sample draws of $\beta = (\beta_1 = 0, \beta_2, \dots, \beta_n)$. For each β draw and given feature x, we obtained a corresponding draw of p using equation 6.3.1.5.

Finally, to convert $p_{i,j}$ for a given demographic group i from case space to deaths space, we transformed using our CFR estimate for demographic i:

$$p_{i,j}^{deaths} = \frac{p_{i,j} \times CFR_i}{\sum_{\hat{j}} p_{i,\hat{j}} \times CFR_i} \qquad (6.3.1.6)$$

This network regression with covariates framework allowed us to use partial and composite data that reported on one or only a few pathogens, or that reported multiple pathogens aggregated together. Networks, however, can be unstable with sparse data and stable estimates have in some cases required the use of Bayesian priors in these models. In particular, we imposed weakly informative Gaussian priors with mean 0 and non-zero variance on all coefficients except intercepts, to drive the model away from spurious effects driven by data sparsity. These priors were based on expert opinion and can be improved with further empirical validation in the future. Table 6.3.4 provides a list of these priors.

Table 6.3.2: Pathogens assessed, covariates, and age groups for each infectious syndrome

Infectious syndrome	Pathogens assessed	Model covariates	Age groups
Bloodstream infections	*Acinetobacter baumannii*, *Citrobacter* spp., *Enterobacter* spp., *Enterococcus faecalis*, *Enterococcus faecium*, other enterococci, *Escherichia coli*, fungus, group A *Streptococcus*, group B *Streptococcus*, *Klebsiella pneumoniae*, *Neisseria meningitidis*, non-typhoidal *Salmonella*, polymicrobial, *Proteus* spp., *Pseudomonas aeruginosa*, *Salmonella* Typhi, *Serratia* spp., *Staphylococcus aureus*, *Streptococcus pneumoniae*	HAQ Index,[14] age group, age-standardised proportion of intravenous drug use,[23] proportion coverage by PCV3 vaccine,[35] indicator variable for Europe	Neonatal, Post-neonatal–5, 5–50, 50–70, 70+
Infections of bones, joints, and related organs	*Enterococcus faecalis*, *Enterococcus faecium*, other enterococci, *Escherichia coli*, group A *Streptococcus*, group B *Streptococcus*, *Klebsiella pneumoniae*, *Pseudomonas aeruginosa*, *Staphylococcus aureus*	HAQ Index, age group	Under 5, 5–50, 50–70, 70+

Endocarditis and other cardiac infections	See bloodstream infection pathogens	Not explicitly modelled. Pathogen distribution for bloodstream infections is used.	Neonatal, Post-neonatal–5, 5–50, 50–70, 70+
Diarrhoea	Adenovirus, *Aeromonas* spp., Amebiasis, *Campylobacter* spp., *Clostridium difficile*, cryptosporidium, enteropathogenic *Escherichia coli*, enterotoxigenic *Escherichia coli*, non-typhoidal *Salmonella*, norovirus, rotavirus, *Shigella* spp., *Vibrio cholerae*	Not modelled here. GBD diarrhoea aetiology estimates are used.	GBD most detailed age groups
Lower respiratory infections and all related infections in the thorax	*Acinetobacter baumannii*, *Chlamydia* spp., *Enterobacter* spp., *Escherichia coli*, fungus, group B *Streptococcus*, *Haemophilus influenzae*, *Klebsiella pneumoniae*, *Legionella* spp., *Mycoplasma* spp., polymicrobial, *Pseudomonas aeruginosa*, *Staphylococcus aureus*, *Streptococcus pneumoniae*, virus	HAQ Index, proportion coverage by PCV3 vaccine, proportion coverage by Hib3 vaccine,[35] age group, HAI/CAI	Neonatal, Post-neonatal–5, 5–50, 50–70, 70+
Meningitis and other bacterial central nervous system infections	*Escherichia coli*, group B *Streptococcus*, *Haemophilus influenzae*, *Klebsiella pneumoniae*, *Listeria monocytogenes*, *Neisseria meningitidis*, *Staphylococcus aureus*, *Streptococcus pneumoniae*, virus	HAQ Index, proportion coverage by PCV3 vaccine, proportion coverage by Hib3 vaccine, age group, proportion of population covered by '10-'15 MenAfriVac rollout[32,36]	Neonatal, Post-neonatal–5, 5-50, 50-70, 70+
Peritoneal and intra-abdominal infections	*Citrobacter* spp., *Enterobacter* spp., *Enterococcus faecalis*, *Enterococcus faecium*, *Escherichia coli*, *Klebsiella pneumoniae*, other *Klebsiella* species, *Proteus* spp., *Pseudomonas aeruginosa*, *Serratia* spp., *Staphylococcus aureus*	HAQ Index, age group	Under 5, 5–50, 50–70, 70+
Bacterial infections of the skin and subcutaneous systems	*Acinetobacter baumannii*, *Enterobacter* spp., *Enterococcus faecalis*, other enterococci, *Escherichia coli*, group A *Streptococcus*, group B *Streptococcus*, *Klebsiella pneumoniae*, *Proteus* spp., *Pseudomonas aeruginosa*, *Staphylococcus aureus*	HAQ Index, age group	Under 5, 5–50, 50–70, 70+
Urinary tract infections and pyelonephritis	*Acinetobacter baumannii*, *Citrobacter* spp., *Enterobacter* spp., *Enterococcus faecalis*, *Enterococcus faecium*, other enterococci, *Escherichia coli*, group B *Streptococcus*, *Klebsiella pneumoniae*, *Morganella* spp., *Proteus* spp., *Providencia* spp., *Pseudomonas aeruginosa*, *Serratia* spp., *Staphylococcus aureus*	HAQ Index, age group, HAI/CAI	Under 5, 5–50, 50–70, 70+

Group A *Streptococcus* = *Streptococcus pyogenes.* Group B *Streptococcus* = *Streptococcus agalactiae.* HAQ Index = Healthcare Access and Quality Index. HAI/CAI = hospital-acquired infection/community-acquired infection.

Table 6.3.3: Number of data points and parameters in each pathogen distribution model

Infectious syndrome	Subtype	Number of data points	Number of parameters
LRI+		158967	135
BSI		126417	180
Skin		1105	55
CNS	Neonatal	25615	81
CNS	Post neonatal	25579	81
UTI		23662	96
Bone+		1870	45
Intra-abdominal		2458	55

BSI = Bloodstream infections. CNS = Meningitis and other bacterial central nervous system infections. LRI+ = Lower respiratory infections and all related infections in the thorax. Intra-abdominal = Peritoneal and intra-abdominal infections. Skin = Bacterial infections of the skin and subcutaneous systems. UTI = Urinary tract infections and pyelonephritis. Bone+ = Infections of bones, joints, and related organs.

Table 6.3.4. Gaussian prior standard deviations for non-intercept coefficients for each pathogen distribution model

Infectious syndrome	Sub-type	Gaussian prior mean	Gaussian prior standard deviation
BSI		0	0.1
CNS	Neonatal	0	0.1

CNS	Non-neonatal	0	0.1
LRI+		0	0.1
Intra-abdominal		0	0.3
Skin		0	0.3
UTI		0	0.02 for *A. baumannii*/HAQ Index coefficient<br>0.1 for all others
Bone+		0	0.5

BSI = Bloodstream infections. CNS = Meningitis and other bacterial central nervous system infections. LRI+ = Lower respiratory infections and all related infections in the thorax. Intra-abdominal = Peritoneal and intra-abdominal infections. Skin = Bacterial infections of the skin and subcutaneous systems. UTI = Urinary tract infections and pyelonephritis. Bone+ = Infections of bones, joints, and related organs.

Section 6.4: Exceptions and special handling

There were several notable exceptions and special handling decisions made for each individual pathogen distribution model. We hope to address many of these exceptions with more sustainable methods in our future work.

Section 6.4.1: Cardiac infections

For cardiac infections, we used the pathogen distribution for bloodstream infections rather than estimating specific distributions for these syndromes, due to a lack of complete literature reviews on the aetiologies and case-fatality rates of these syndromes. We consider this to be a serious limitation of our methodology, but do not anticipate that is seriously impactful on our final estimates, since cardiac infections are the third-lowest syndrome by deaths, comprising just 0.91% (95% UI 0.69–1.17) of all sepsis deaths.

Section 6.4.2: Diarrhoea

In diarrhoea patients, cultures of specimens taken from the gastrointestinal tract, bowels, rectum, or stool are almost always affected by contaminants or pathogens that are not the cause of diarrhoea. For this reason, we believe that our input data and modelling framework are not able to accurately capture the aetiologies of diarrhoea. We chose to use GBD estimates of the aetiologies of diarrhoea in deaths instead of running our own model.[37] These estimates are based on the odds ratio of having diarrhoea given the detection of a pathogen, obtained from the Global Enteric Multicenter Study, therefore removing the influence of any pathogen that does not increase the risk of diarrhoea.

A major limitation of using this study is that the GBD diarrhoea aetiology estimates are population attributable fractions (PAFs) for each pathogen. These PAFs may add to greater than 1 and the authors made no attempt to quantify the extent of co-occurrence of pathogens. This is inconsistent with the pathogen distribution estimation method used in our study, which quantifies polymicrobial infections and estimates all pathogens as mono-infections. In order to avoid duplication of cases in our framework, we had to make some assumptions about the co-occurrence of pathogens in diarrhoea. We chose to normalise the PAFs to 1 for any demographic where the sum of GBD diarrhoea aetiology PAFs was greater than 1. This assumed that co-occurrence of pathogens was random and that the “other” pathogens category was negligible in these demographics. We made no adjustment to demographics where the PAFs added to less than 1. To convert the fatal PAFs to a distribution of aetologies in incidence, we rescaled the distribution according to our estimates of the pathogen-specific case fatality ratios of diarrhoea, calculated as described in section 5.

Section 6.4.3: Bacterial infections of the skin and subcutaneous systems

Certain skin and subcutaneous samples are easily affected by contaminants, colonization, and other pathogens that are not the cause of infection. For this reason, we considered microbial data and mortality surveillance to be too difficult to extract meaningful aetiology information from, and instead used only ICD-coded databases (multiple cause of death, hospital discharge, and linkage data) and literature studies as inputs into our model of the pathogen distribution of skin infections.

Section 6.4.4: Lower respiratory infections and all related infections in the thorax

We dropped all laboratory-based microbiological data on *S. pneumoniae* for community-acquired LRI and thorax infections in non-neonatal age groups. This ensured that data from the vaccine probe analysis for *S. pneumoniae*, which we trusted to be most accurate, would predominate. Our model also predicted a high fraction of polymicrobial in neonates for community-acquired infections for this infectious syndrome based off of only 1 study, CHAMPS. We found this to be implausible and so dropped polymicrobial from the estimates for this age group in community-acquired infections and renormalised the proportions for all other pathogens to 1.

Section 6.4.5: Peritoneal and intra-abdominal infections

Because dedicated anaerobic cultures were not routinely performed for peritoneal samples, we dropped all anaerobes observed in the data for and excluded anaerobes as an aetiology of intra-abdominal infections.

Section 6.4.6: Meningitis and other bacterial central nervous system infections

Due to the unique pattern of meningitis in neonates, particularly the high prevalence of GBS, we modeled neonatal and adult central nervous syndrome infections separately.

Section 6.4.7: Infectious syndromes not modelled

For three infectious syndromes, we did not run a pathogen distribution model. These syndromes are all caused by distinct pathogens whose individual burdens are already estimated in GBD as separate causes of death. For these syndromes, we simply used GBD estimates (table 6.4.7.1)

Table 6.4.7.1: Infectious syndromes for which we used GBD estimates to obtain the pathogen distribution

Infectious syndrome	Pathogens	GBD causes
Typhoid, paratyphoid, and invasive non-typhoidal Salmonella	*Salmonella* Typhi	Typhoid fever
	Salmonella Paratyphi	Paratyphoid fever
	Non-typhoidal *Salmonella*	Invasive non-typhoidal Salmonella
Tuberculosis	*Mycobacterium tuberculosis*	Tuberculosis
Gonorrhoea and chlamydia	*Neisseria gonorrhoeae*	Gonococcal infection
	Chlamydia trachomatis	Chlamydial infection

Section 6.5: Model validation

To assess model validity, we calculated the root mean square error (RMSE) and coefficient of determination (R^2) for each pathogen distribution model in proportion space for both in-sample and out-of-sample predictions (Table 6.5.1). Proportions were predicted for each observation using the specific denominator observed from that study. For example, if a given study reported on only *E. coli* and *S. pneumoniae,* the predictions for model validation for this study were calculated as proportions of the total for *E. coli* and *S. pneumoniae.* In order to calculate out-of-sample fit, we perform non-exhaustive cross-validation, with each round of the validation holding out 1 country of data at a time. This leave-one-country-out approach simulates the prediction task of estimating the pathogen distribution of a country for which we have no data.

R^2 ranges from 0.784 to 0.867 in-sample and from 0.755 to 0.837 out of sample, indicating good model fit with only modest losses when data are moved out of sample. RMSE ranges from 0.129 to 0.149 in-sample and from 0.141 to 0.159 out of sample. Given that the data are expected to vary from the model predictions according to the observation-level variance, and the fact that the RMSEs are relatively consistent between in-sample and out-of-sample, these RMSEs are reasonable. Overall, these metrics show that these models have good fit and good out-of-sample predictive ability.

Table 6.5.1 In-sample and out-of-sample validation metrics for pathogen distribution models

Infectious syndrome	Model type	R^2		RMSE	
		In sample	Out of sample	In sample	Out of sample
Bacterial infections of the skin and subcutaneous systems		0.808	0.771	0.129	0.141
Bloodstream infections		0.822	0.785	0.128	0.141
Infections of bones, joints, and related organs		0.858	0.837	0.141	0.151
Lower respiratory infections and all related infections in the thorax		0.810	0.780	0.142	0.153
Meningitis and other bacterial central nervous system infections	Neonatal	0.858	0.803	0.134	0.158
	Non-neonatal	0.867	0.822	0.129	0.150
Peritoneal and intra-abdominal infections		0.815	0.812	0.147	0.148
Urinary tract infections and pyelonephritis		0.784	0.755	0.149	0.159

Out of sample metrics calculated using leave-one-country-out cross validation

Section 7: PRISMA Compliance: Preferred Reporting Items for Systematic Reviews and Meta-Analyses

PRISMA 2020 Checklist

Section and Topic	Item #	Checklist item	Location where item is reported
**TITLE**			
Title	1	Identify the report as a systematic review.	This report is not a systematic review, so it is not titled as such. The report does contain input data sought from systematic literature reviews, which have been documented in the appendix in Sections 2 and 6.
**ABSTRACT**			
Abstract	2	See the PRISMA 2020 for Abstracts checklist.	See PRISMA 2020 for Abstracts Checklist below (appendix pp36-37)
**INTRODUCTION**			
Rationale	3	Describe the rationale for the review in the context of existing knowledge.	The rationale can be found in the "Introduction" section in the main text in paragraphs 1 & 2.
Objectives	4	Provide an explicit statement of the objective(s) or question(s) the review addresses.	The objective can be found in the "Introduction" section in the main text in paragraph 3.

Section and Topic	Item #	Checklist item	Location where item is reported
**METHODS**			
Eligibility criteria	5	Specify the inclusion and exclusion criteria for the review and how studies were grouped for the syntheses.	The inclusion and exclusion criteria is described in Section 2 of the appendix for systematic reviews and in the Data Inputs section of the main text for all other data types.
Information sources	6	Specify all databases, registers, websites, organisations, reference lists and other sources searched or consulted to identify studies. Specify the date when each source was last searched or consulted.	All searches were conducted in PubMed and this is in section 2.5 of the appendix.
Search strategy	7	Present the full search strategies for all databases, registers and websites, including any filters and limits used.	The search strings and inclusion and exclusion criteria for systematic reviews are described in Section 2 of the appendix.<br><br>The inclusion criteria for all other data types is found in the Data Inputs section of the main text.
Selection process	8	Specify the methods used to decide whether a study met the inclusion criteria of the review, including how many reviewers screened each record and each report retrieved, whether they worked independently, and if applicable, details of automation tools used in the process.	The exclusion criteria for the systematic reviews are documented in Section 2 of the appendix and were screened by a Researcher and corresponding author on this report. No automation was used.<br><br>The inclusion criteria for all other data types is found in the Data Inputs section of the main text.
Data collection process	9	Specify the methods used to collect data from reports, including how many reviewers collected data from each report, whether they worked independently, any processes for obtaining or confirming data from study investigators, and if applicable, details of automation tools used in the process.	The extraction template for the systematic reviews will be published along with the GHDx upon publication. Articles were screened by a Researcher and corresponding author on this report. No automation was used.

Section and Topic	Item #	Checklist item	Location where item is reported
Data items	10a	List and define all outcomes for which data were sought. Specify whether all results that were compatible with each outcome domain in each study were sought (e.g. for all measures, time points, analyses), and if not, the methods used to decide which results to collect.	The information on outcomes for input data can be found in the "Infectious Syndrome" and "Pathogen Distribution" sections of the main text and in Section 2 of the appendix.
	10b	List and define all other variables for which data were sought (e.g. participant and intervention characteristics, funding sources). Describe any assumptions made about any missing or unclear information.	All information on the variables in the input data are captured above.
Study risk of bias assessment	11	Specify the methods used to assess risk of bias in the included studies, including details of the tool(s) used, how many reviewers assessed each study and whether they worked independently, and if applicable, details of automation tools used in the process.	The potential bias of the input data, modeling, and the associated limitations can be found in the "Limitations" section of the main text.
Effect measures	12	Specify for each outcome the effect measure(s) (e.g. risk ratio, mean difference) used in the synthesis or presentation of results.	NA
Synthesis methods	13a	Describe the processes used to decide which studies were eligible for each synthesis (e.g. tabulating the study intervention characteristics and comparing against the planned groups for each synthesis (item #5)).	This information is available in the "Data Inputs" section of the main text and in Section 2 of the appendix.
	13b	Describe any methods required to prepare the data for presentation or synthesis, such as handling of missing summary statistics, or data conversions.	Detailed methods on the estimation process have been published previously[1] and are available in the "Pathogen Distribution" section of the main text and sections 4 and 5 of the appendix, together with

Section and Topic	Item #	Checklist item	Location where item is reported
			exceptions and special handling decisions.
	13c	Describe any methods used to tabulate or visually display results of individual studies and syntheses.	NA
	13d	Describe any methods used to synthesize results and provide a rationale for the choice(s). If meta-analysis was performed, describe the model(s), method(s) to identify the presence and extent of statistical heterogeneity, and software package(s) used.	Details on the methods can be found in the "Methods" section of the main text, Section 4 and 5 of the appendix and have been published previously.[1]
	13e	Describe any methods used to explore possible causes of heterogeneity among study results (e.g. subgroup analysis, meta-regression).	Details on how heterogeneity of studies was accounted for can be found in Section 4.4 of the appendix.
	13f	Describe any sensitivity analyses conducted to assess robustness of the synthesized results.	This can be found in the "Uncertainty and validity analysis" section of the main text and Section 5.5 of the appendix.
Reporting bias assessment	14	Describe any methods used to assess risk of bias due to missing results in a synthesis (arising from reporting biases).	This can be found in the second paragraph of the "Pathogen Distribution" section of the main text. Further information can be found in Section 4 and 5 of the appendix.
Certainty assessment	15	Describe any methods used to assess certainty (or confidence) in the body of evidence for an outcome.	The method for determining uncertainty is described for each component model in Sections 3.3-5 of the appendix.
**RESULTS**			
Study selection	16a	Describe the results of the search and selection process, from the number of records identified in the search to the number of studies included in the review, ideally using a flow diagram.	Flow diagrams for every infectious syndrome and underlying pathogen are available in Section 6.

**Section and Topic**	**Item #**	**Checklist item**	**Location where item is reported**
	16b	Cite studies that might appear to meet the inclusion criteria, but which were excluded, and explain why they were excluded.	We did not encounter studies that meet this definition. Any studies outliered will be included in the citation list on the GHDx and will be available upon publication.
Study characteristics	17	Cite each included study and present its characteristics.	All study citations will be included in the GHDx record for the manuscript and will be available upon publication.
Risk of bias in studies	18	Present assessments of risk of bias for each included study.	The assessment of bias in the input data is available in the limitations section and previously published.[1]
Results of individual studies	19	For all outcomes, present, for each study: (a) summary statistics for each group (where appropriate) and (b) an effect estimate and its precision (e.g. confidence/credible interval), ideally using structured tables or plots.	Because this report is not a systematic review, this was not included.
Results of syntheses	20a	For each synthesis, briefly summarise the characteristics and risk of bias among contributing studies.	The bias of input data and the overall bias in our study can be found in the “Limitations” section of the main text.
	20b	Present results of all statistical syntheses conducted. If meta-analysis was done, present for each the summary estimate and its precision (e.g. confidence/credible interval) and measures of statistical heterogeneity. If comparing groups, describe the direction of the effect.	The results can be found in the “Results” section of the main text, throughout the text in the manuscript and in the “Uncertainty and validity analysis” section of the main text and Sections 3-6 of the appendix.
	20c	Present results of all investigations of possible causes of heterogeneity among study results.	Because this report is not a systematic review, this was not included.
	20d	Present results of all sensitivity analyses conducted to assess the	This can be found in the Uncertainty and validity analysis

Section and Topic	Item #	Checklist item	Location where item is reported
		robustness of the synthesized results.	section of the main text and Section 5.5 of the appendix.
Reporting biases	21	Present assessments of risk of bias due to missing results (arising from reporting biases) for each synthesis assessed.	Assessments and mediation of risk can be found in the pathogen distribution modelling framework and can be found in the "Pathogen Distribution" section in the main text.
Certainty of evidence	22	Present assessments of certainty (or confidence) in the body of evidence for each outcome assessed.	This can be found in the "Uncertainty and validity analysis" section of the main text and Section 3.3-6 of the appendix.
**DISCUSSION**			
Discussion	23a	Provide a general interpretation of the results in the context of other evidence.	This can be found in the "Research In Context" section of the main text.
	23b	Discuss any limitations of the evidence included in the review.	This can be found in the "Discussion" section of the main text in paragraph 9.
	23c	Discuss any limitations of the review processes used.	The limitations and the exclusion criteria can be found in Section 2 of the appendix.
	23d	Discuss implications of the results for practice, policy, and future research.	This can be found in the "Discussion" section of the main text.
**OTHER INFORMATION**			
Registration and protocol	24a	Provide registration information for the review, including register name and registration number, or state that the review was not registered.	The entirety of the Global Burden of Disease, Injuries, and Risk Factors Study has been registered and approved through the UW IRB. The systematic reviews contained in this manuscript were not registered on its own.
	24b	Indicate where the review protocol can be accessed, or state that a protocol was not prepared.	We did not prepare a review protocol.

Section and Topic	Item #	Checklist item	Location where item is reported
	24c	Describe and explain any amendments to information provided at registration or in the protocol.	NA
Support	25	Describe sources of financial or non-financial support for the review, and the role of the funders or sponsors in the review.	Financial support can be found in the “Acknowledgments” section of the main text.
Competing interests	26	Declare any competing interests of review authors.	These can be found in the “Competing interests” section of the main text.
Availability of data, code and other materials	27	Report which of the following are publicly available and where they can be found: template data collection forms; data extracted from included studies; data used for all analyses; analytic code; any other materials used in the review.	The data collection forms, citations for all data used, analytic code and the results will be available on the GHDx upon publication.

From: Page MJ, McKenzie JE, Bossuyt PM, Boutron I, Hoffmann TC, Mulrow CD, et al. The PRISMA 2020 statement: an updated guideline for reporting systematic reviews. BMJ 2021;372:n71. doi: 10.1136/bmj.n71

For more information, visit: http://www.prisma-statement.org/

PRISMA 2020 for Abstracts Checklist

Section and Topic	Item #	Checklist item	Reported (Yes/No)
**TITLE**			
Title	1	Identify the report as a systematic review.	No, this is not a systematic review
**BACKGROUND**			
Objectives	2	Provide an explicit statement of the main objective(s) or question(s) the review addresses.	Yes
**METHODS**			
Eligibility criteria	3	Specify the inclusion and exclusion criteria for the review.	No
Information sources	4	Specify the information sources (e.g. databases, registers) used to identify studies and the date when each was last searched.	No
Risk of bias	5	Specify the methods used to assess risk of bias in	No

Section and Topic	Item #	Checklist item	Reported (Yes/No)
		the included studies.	
Synthesis of results	6	Specify the methods used to present and synthesise results.	Yes
**RESULTS**			
Included studies	7	Give the total number of included studies and participants and summarise relevant characteristics of studies.	No
Synthesis of results	8	Present results for main outcomes, preferably indicating the number of included studies and participants for each. If meta-analysis was done, report the summary estimate and confidence/credible interval. If comparing groups, indicate the direction of the effect (i.e. which group is favoured).	Yes
**DISCUSSION**			
Limitations of evidence	9	Provide a brief summary of the limitations of the evidence included in the review (e.g. study risk of bias, inconsistency and imprecision).	Yes
Interpretation	10	Provide a general interpretation of the results and important implications.	Yes
**OTHER**			
Funding	11	Specify the primary source of funding for the review.	Yes
Registration	12	Provide the register name and registration number.	N/A

From: Page MJ, McKenzie JE, Bossuyt PM, Boutron I, Hoffmann TC, Mulrow CD, et al. The PRISMA 2020 statement: an updated guideline for reporting systematic reviews. BMJ 2021;372:n71. doi: 10.1136/bmj.n71

For more information, visit: http://www.prisma-statement.org/

PRISMA Diagrams

PRISMA 2020 flow diagrams for new systematic reviews which included searches of databases and registers only

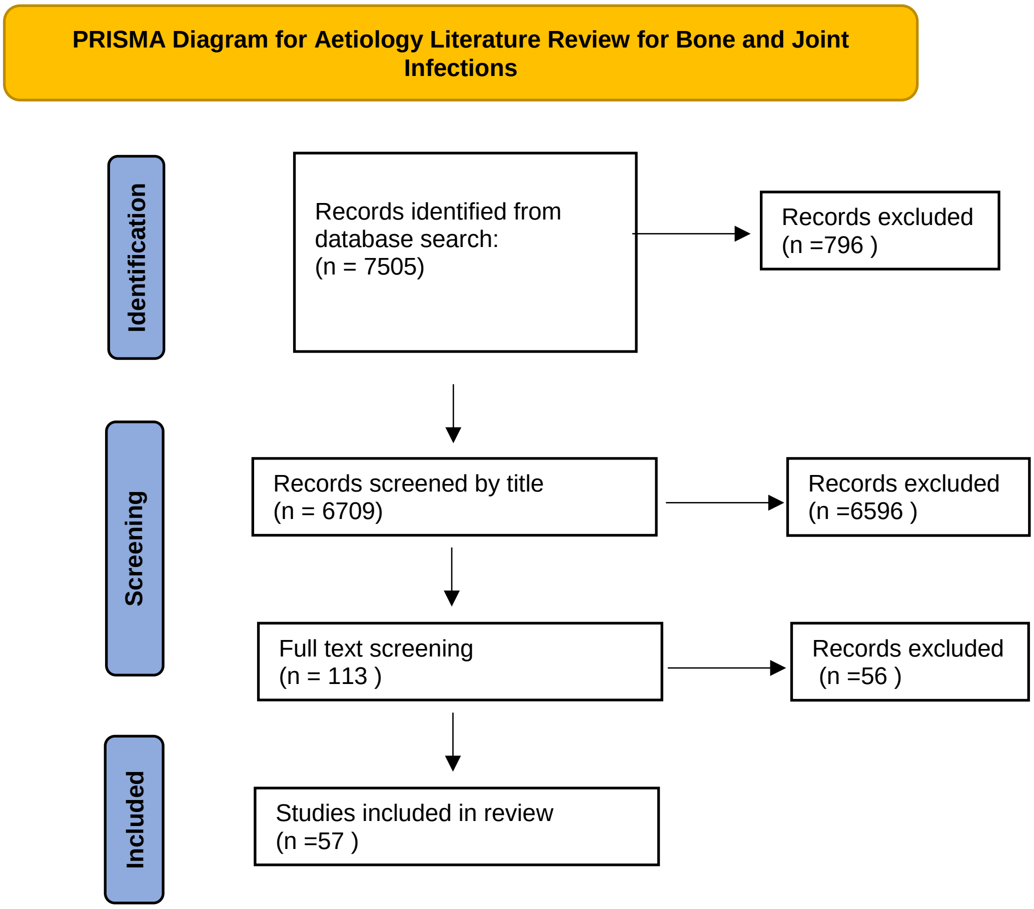

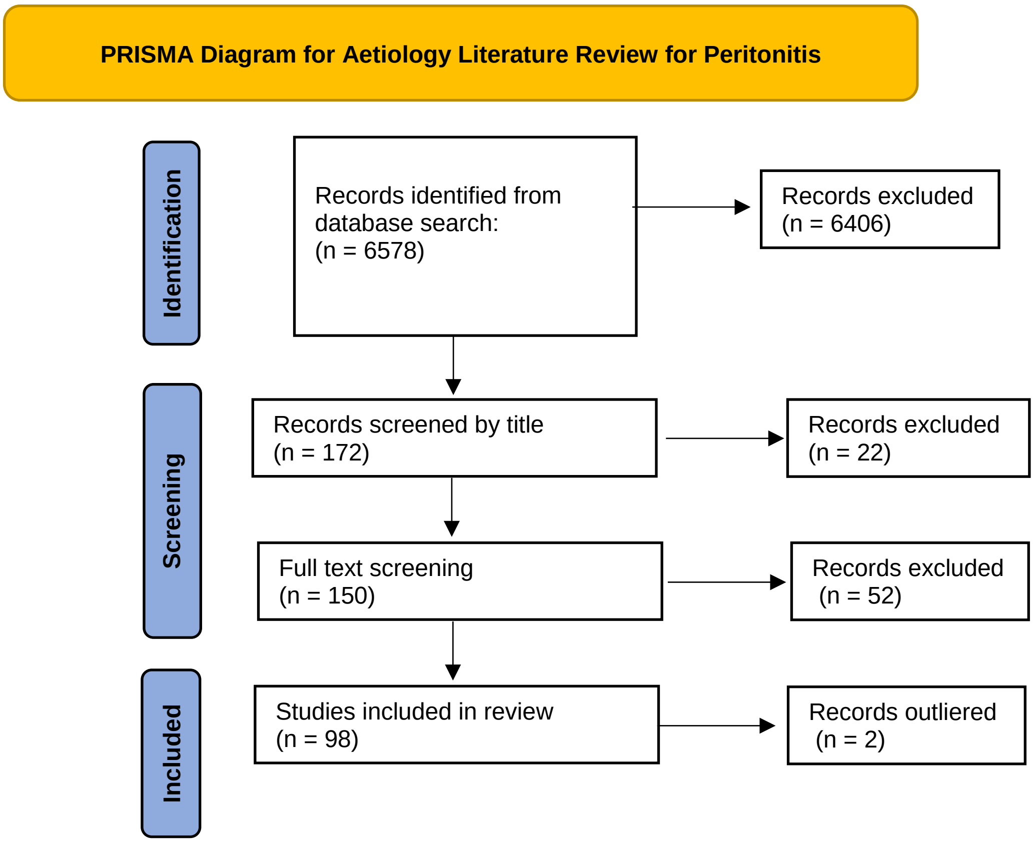
PRISMA Diagram for Aetiology Literature Review for Peritonitis
Identification
Records identified from database search: (n = 6578)
Records excluded (n = 6406)
Screening
Records screened by title (n = 172)
Records excluded (n = 22)
Full text screening (n = 150)
Records excluded (n = 52)
Included
Studies included in review (n = 98)
Records outliered (n = 2)

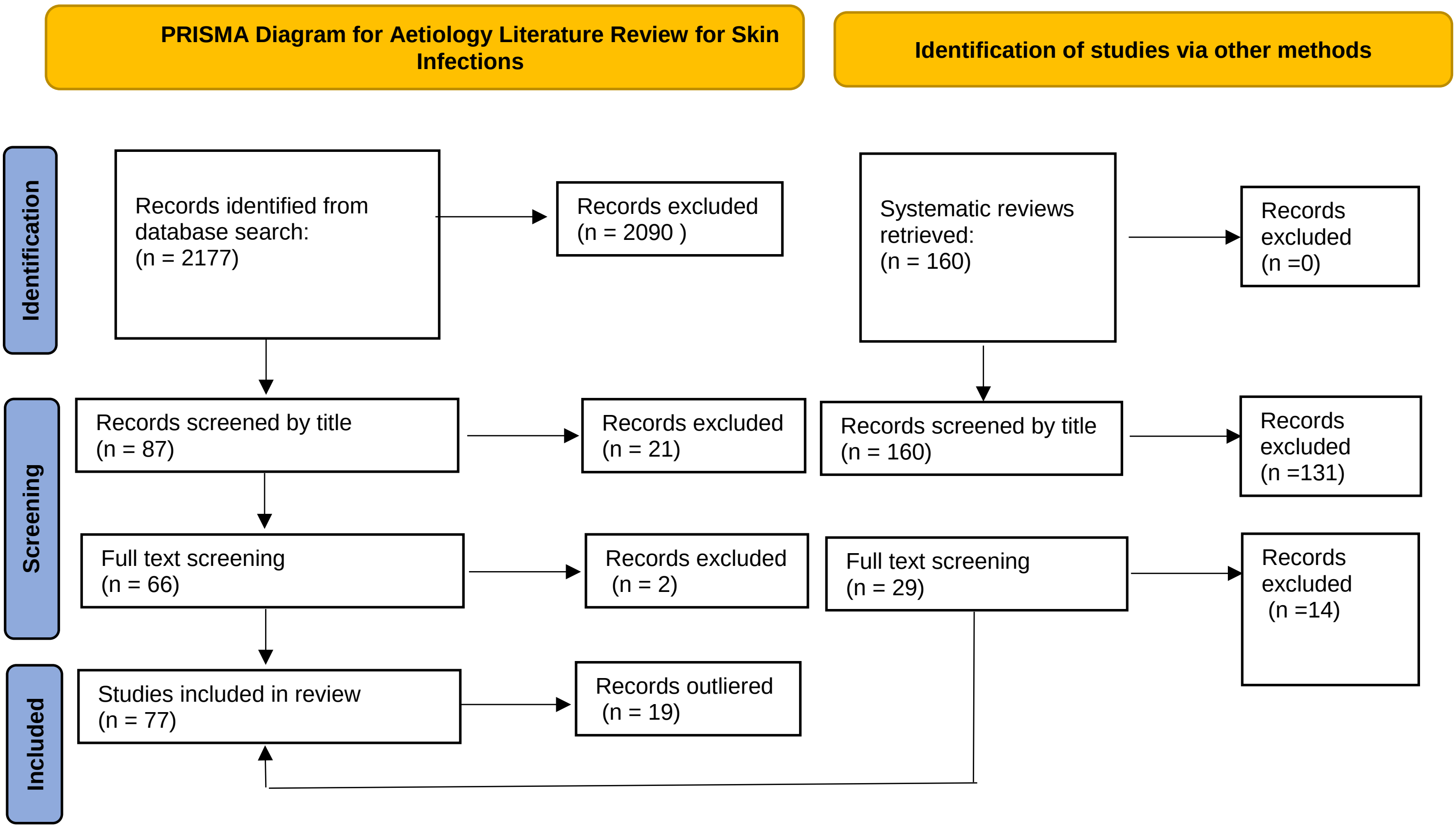
PRISMA Diagram for Aetiology Literature Review for Skin Infections
Identification of studies via other methods
Identification
Screening
Included
Records identified from database search: (n = 2177)
Records excluded (n = 2090)
Systematic reviews retrieved: (n = 160)
Records excluded (n =0)
Records screened by title (n = 87)
Records excluded (n = 21)
Records screened by title (n = 160)
Records excluded (n =131)
Full text screening (n = 66)
Records excluded (n = 2)
Full text screening (n = 29)
Records excluded (n =14)
Studies included in review (n = 77)
Records outliered (n = 19)

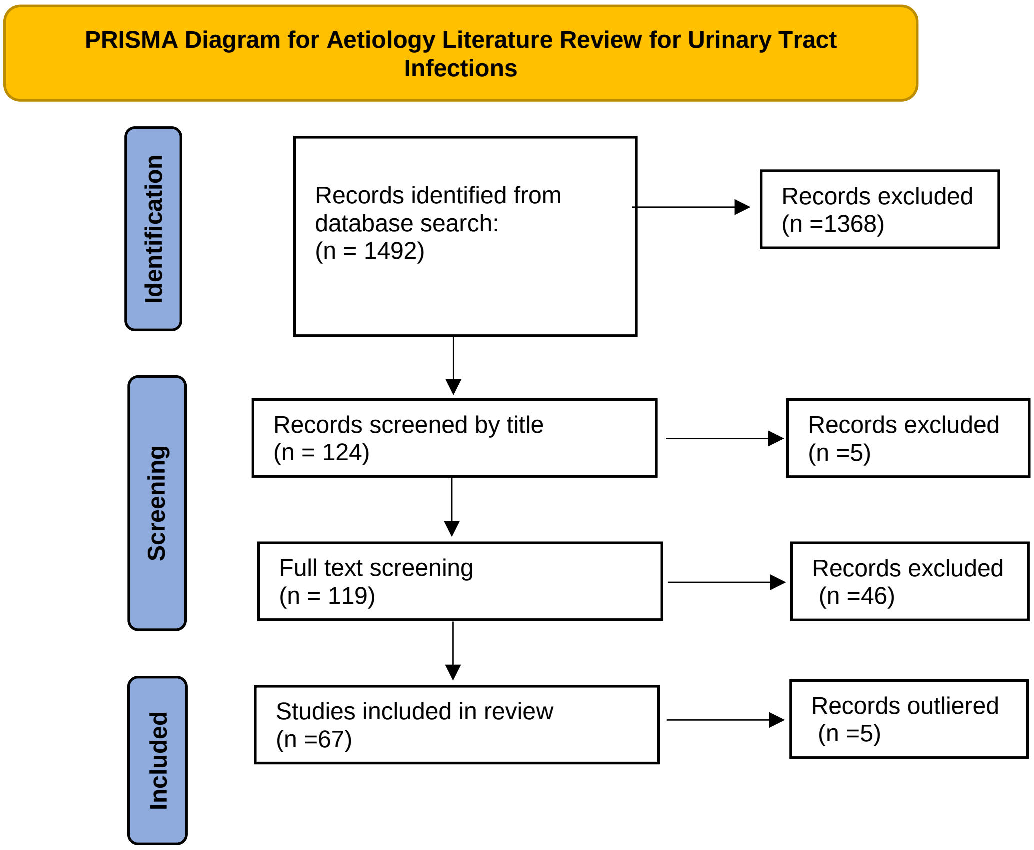
PRISMA Diagram for Aetiology Literature Review for Urinary Tract Infections
Identification
Screening
Included
Records identified from database search: (n = 1492)
Records excluded (n =1368)
Records screened by title (n = 124)
Records excluded (n =5)
Full text screening (n = 119)
Records excluded (n =46)
Studies included in review (n =67)
Records outliered (n =5)

PRISMA flow diagram of neonatal sepsis review

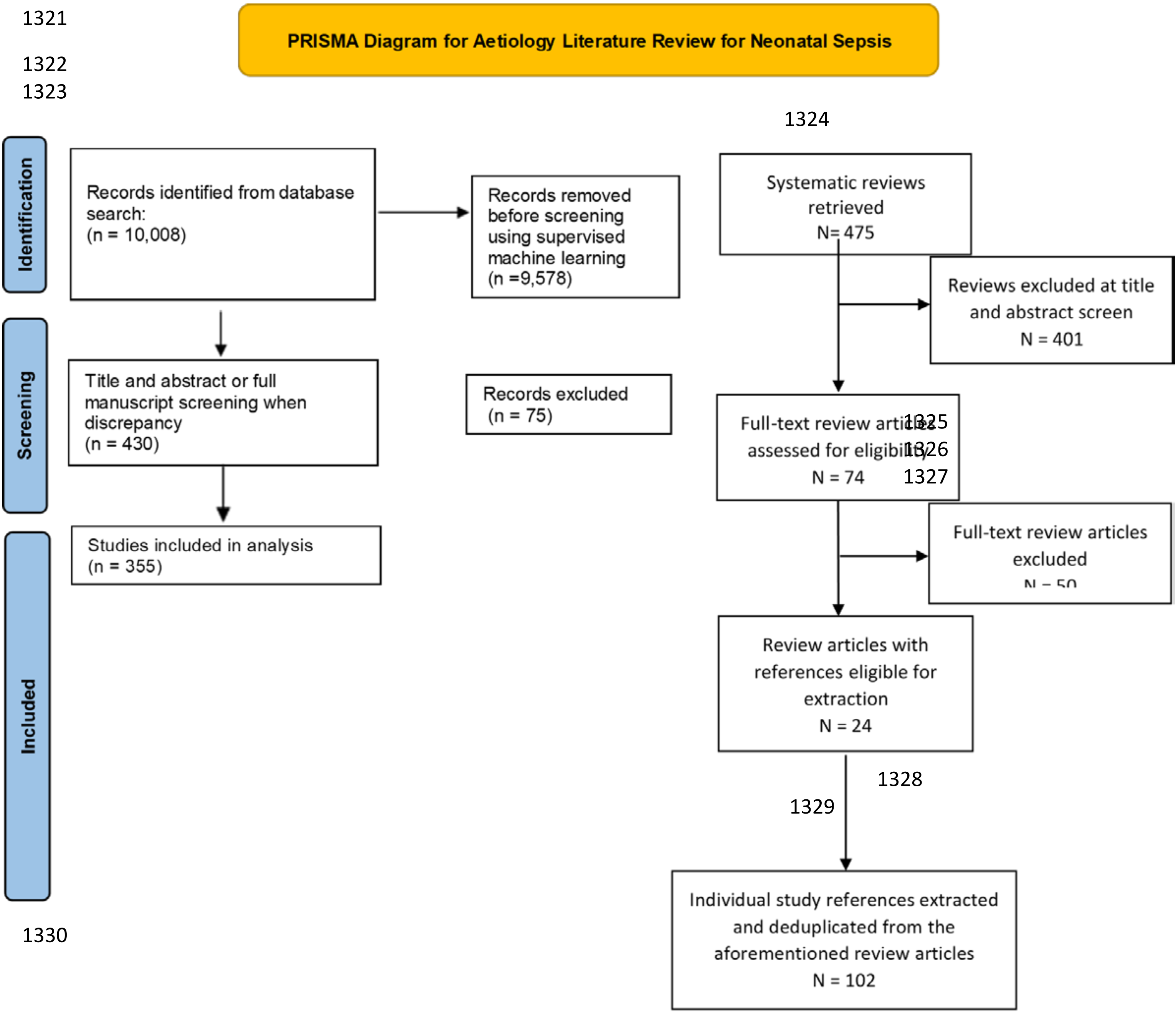

PRISMA flow diagram of lower respiratory infections review

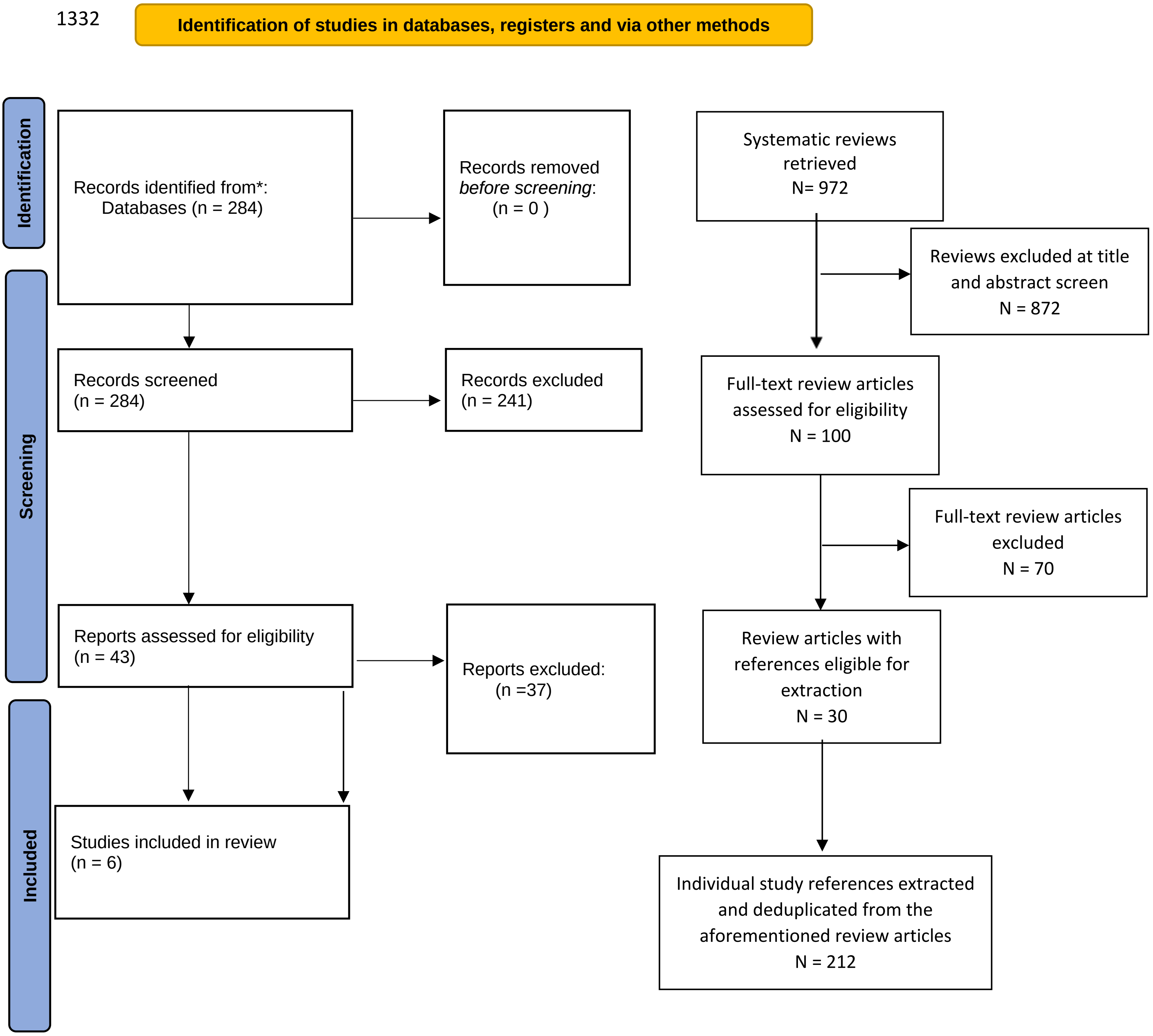

Section 8: GATHER Compliance: Guidelines for Accurate and Transparent Health Estimates Reporting

Item #	Checklist item	Reported on page #
Objectives and funding		
1	Define the indicator(s), populations (including age, sex, and geographic entities), and time period(s) for which estimates were made.	Main text methods section (overview)
2	List the funding sources for the work.	Main text funding statement and acknowledgements
Data Inputs		
For all data inputs from multiple sources that are synthesised as part of the study:		
3	Describe how the data were identified and how the data were accessed.	Main text methods section and supplementary appendix sections
4	Specify the inclusion and exclusion criteria. Identify all ad-hoc exclusions.	Supplementary appendix
5	Provide information on all included data sources and their main characteristics. For each data source used, report reference information or contact name/institution, population represented, data collection method, year(s) of data collection, sex and age range, diagnostic criteria or measurement method, and sample size, as relevant.	Some data source information in supplementary appendix; main characteristics of data, metadata, and/or NIDs available at GHDX LINK
6	Identify and describe any categories of input data that have potentially important biases (e.g., based on characteristics listed in item 5).	Main text limitations section and supplementary appendix (biases for input data in each modelling step identified in each section)
For data inputs that contribute to the analysis but were not synthesised as part of the study:		
7	Describe and give sources for any other data inputs.	GBD 2019 estimates
For all data inputs:		
8	Provide all data inputs in a file format from which data can be efficiently extracted (e.g., a spreadsheet rather than a PDF), including all relevant meta-data listed in item 5. For any data inputs that cannot be shared because of ethical or legal reasons, such as third-party ownership, provide a contact name or the name of the institution that retains the right to the data.	Data inputs and/or contact information available at<br>http://ghdx.healthdata.org/gbd-2019/data-input-sources

Data analysis		
9	Provide a conceptual overview of the data analysis method. A diagram may be helpful.	Main text methods section
10	Provide a detailed description of all steps of the analysis, including mathematical formulae. This description should cover, as relevant, data cleaning, data pre-processing, data adjustments and weighting of data sources, and mathematical or statistical model(s).	Supplementary appendix
11	Describe how candidate models were evaluated and how the final model(s) were selected.	Supplementary appendix
12	Provide the results of an evaluation of model performance, if done, as well as the results of any relevant sensitivity analysis.	Supplementary appendix
13	Describe methods for calculating uncertainty of the estimates. State which sources of uncertainty were, and were not, accounted for in the uncertainty analysis.	Main text methods section (uncertainty analysis) and limitations section (scarcity of data) and supplementary appendix
14	State how analytic or statistical source code used to generate estimates can be accessed.	Link to GitHub code found in main text methods section (overview)
Results and Discussion		
15	Provide published estimates in a file format from which data can be efficiently extracted.	Published estimates are available in the main text results section and in the supplementary appendix. CSV files are available upon request to the corresponding author.
16	Report a quantitative measure of the uncertainty of the estimates (e.g. uncertainty intervals).	Uncertainty intervals are provided for all estimates throughout the main text
17	Interpret results in light of existing evidence. If updating a previous set of estimates, describe the reasons for changes in estimates.	Main text introduction and discussion sections
18	Discuss limitations of the estimates. Include a discussion of any modelling assumptions or data limitations that affect interpretation of the estimates.	Main text limitations section and supplementary appendix

Section 9: References

1 Antimicrobial Resistance Collaborators. Global burden of bacterial antimicrobial resistance in 2019: a systematic analysis. *The Lancet* 2022; **399**: 629–55.

2 GBD 2019 Diseases and Injuries Collaborators. Global burden of 369 diseases and injuries in 204 countries and territories, 1990–2019: a systematic analysis for the Global Burden of Disease Study 2019. *The Lancet* 2020; **396**: 1204–22.

3 NHSN. National Healthcare Safety Network (NHSN) Patient safety component manual. 2022; published online Jan.

4 Lozano R, Lopez AD, Atkinson C, Naghavi M, Flaxman AD, Murray CJ. Performance of physician-certified verbal autopsies: multisite validation study using clinical diagnostic gold standards. *Popul Health Metr* 2011; **9**: 32.

5 Naghavi M, Makela S, Foreman K, O'Brien J, Pourmalek F, Lozano R. Algorithms for enhancing public health utility of national causes-of-death data. *Popul Health Metr* 2010; **8**: 9.

6 Johnson SC, Cunningham M, Dippenaar IN, *et al.* Public health utility of cause of death data: applying empirical algorithms to improve data quality. *BMC Med Inform Decis Mak* 2021; **21**: 175.

7 Iburg KM, Mikkelsen L, Adair T, Lopez AD. Are cause of death data fit for purpose? evidence from 20 countries at different levels of socio-economic development. *PLOS ONE* 2020; **15**: e0237539.

8 Birnbaum JK, Murray CJ, Lozano R. Exposing misclassified HIV/AIDS deaths in South Africa. *Bull World Health Organ* 2011; **89**: 278–85.

9 Murray CJL, Rajaratnam JK, Marcus J, Laakso T, Lopez AD. What can we conclude from death registration? Improved methods for evaluating completeness. *PLOS Med* 2010; **7**: e1000262.

10 Foreman KJ, Lozano R, Lopez AD, Murray CJ. Modeling causes of death: an integrated approach using CODEm. *Popul Health Metr* 2012; **10**: 1.

11 Zheng P, Barber R, Sorensen RJD, Murray CJL, Aravkin AY. Trimmed constrained mixed effects models: formulations and algorithms. J Comput Graph Stat. 2021; **30**. DOI:10.48550/arXiv.1909.10700.

12 GBD 2019 Demographics Collaborators. Global age-sex-specific fertility, mortality, healthy life expectancy (HALE), and population estimates in 204 countries and territories, 1950–2019: a comprehensive demographic analysis for the Global Burden of Disease Study 2019. *The Lancet* 2020; **396**: 1160–203.

13 Rudd KE, Johnson SC, Agesa KM, *et al.* Global, regional, and national sepsis incidence and mortality, 1990–2017: analysis for the Global Burden of Disease Study. *The Lancet* 2020; **395**: 200–11.

14 GBD 2016 Healthcare Access and Quality Collaborators. Measuring performance on the Healthcare Access and Quality Index for 195 countries and territories and selected subnational locations: a systematic analysis from the Global Burden of Disease Study 2016. *The Lancet* 2018; **391**: 2236–71.

15 Weiner LM, Webb AK, Limbago B, *et al.* Antimicrobial-resistant pathogens associated with healthcare-associated infections: summary of data reported to the National Healthcare Safety Network at the Centers for Disease Control and Prevention, 2011-2014. *Infect Control Hosp Epidemiol* 2016; **37**: 1288–301.

16 Jones RN. Microbial etiologies of hospital-acquired bacterial pneumonia and ventilator-associated bacterial pneumonia. *Clin Infect Dis Off Publ Infect Dis Soc Am* 2010; **51 Suppl 1**: S81-87.

17 Jain S, Self WH, Wunderink RG, *et al.* Community-acquired pneumonia requiring hospitalization among U.S. adults. *N Engl J Med* 2015; **373**: 415–27.

18 Johansson N, Kalin M, Tiveljung-Lindell A, Giske CG, Hedlund J. Etiology of community-acquired pneumonia: increased microbiological yield with new diagnostic methods. *Clin Infect Dis Off Publ Infect Dis Soc Am* 2010; **50**: 202–9.

19 Czaja CA, Scholes D, Hooton TM, Stamm WE. Population-based epidemiologic analysis of acute pyelonephritis. *Clin Infect Dis Off Publ Infect Dis Soc Am* 2007; **45**: 273–80.

20 Nicolle LE. A practical guide to antimicrobial management of complicated urinary tract infection. *Drugs Aging* 2001; **18**: 243–54.

21 Goto M, Ohl ME, Schweizer ML, Perencevich EN. Accuracy of administrative code data for the surveillance of healthcare-associated infections: a systematic review and meta-analysis. *Clin Infect Dis Off Publ Infect Dis Soc Am* 2014; **58**: 688–96.

22 Wright SB, Huskins WC, Dokholyan RS, Goldmann DA, Platt R. Administrative databases provide inaccurate data for surveillance of long-term central venous catheter-associated infections. *Infect Control Hosp Epidemiol* 2003; **24**: 946–9.

23 GBD 2019 Risk Factors Collaborators. Global burden of 87 risk factors in 204 countries and territories, 1990-2019: a systematic analysis for the Global Burden of Disease Study 2019. *Lancet Lond Engl* 2020; **396**: 1223–49.

24 Mandrekar JN. Receiver operating characteristic curve in diagnostic test assessment. *J Thorac Oncol* 2010; **5**: 1315–6.

25 Zheng P, Barber R, Sorensen RJD, Murray CJL, Aravkin AY. Trimmed constrained mixed effects models: formulations and algorithms. *J Comput Graph Stat* 2021; **0**: 1–13.

26 Ewig S, Schlochtermeier M, Göke N, Niederman MS. Applying sputum as a diagnostic tool in pneumonia: limited yield, minimal impact on treatment decisions. *Chest* 2002; **121**: 1486–92.

27 Ogawa H, Kitsios GD, Iwata M, Terasawa T. Sputum gram stain for bacterial pathogen diagnosis in community-acquired pneumonia: a systematic review and Bayesian meta-analysis of diagnostic accuracy and yield. *Clin Infect Dis* 2020; **71**: 499–513.

28 Feikin DR, Scott JAG, Gessner BD. Use of vaccines as probes to define disease burden. *Lancet Lond Engl* 2014; **383**: 1762–70.

29 GBD 2016 Lower Respiratory Infections Collaborators. Estimates of the global, regional, and national morbidity, mortality, and aetiologies of lower respiratory infections in 195 countries, 1990-2016: a systematic analysis for the Global Burden of Disease Study 2016. *Lancet Infect Dis* 2018; **18**: 1191–210.

30 Johnson HL, Deloria-Knoll M, Levine OS, *et al.* Systematic evaluation of serotypes causing invasive pneumococcal disease among ahildren under five: The Pneumococcal Global Serotype Project. *PLOS Med* 2010; **7**: e1000348.

31 Grijalva CG, Nuorti JP, Arbogast PG, Martin SW, Edwards KM, Griffin MR. Decline in pneumonia admissions after routine childhood immunisation with pneumococcal conjugate vaccine in the USA: a time-series analysis. *The Lancet* 2007; **369**: 1179–86.

32 GBD 2019 Diseases and Injuries Collaborators. Global burden of 369 diseases and injuries in 204 countries and territories, 1990–2019: a systematic analysis for the Global Burden of Disease Study 2019. *The Lancet* 2020; **396**: 1204–22.

33 Nocedal J, Wright S. Numerical optimization, 2nd edn. New York: Springer-Verlag, 2006 DOI:10.1007/978-0-387-40065-5.

34 Ly A, Marsman M, Verhagen J, Grasman RPPP, Wagenmakers E-J. A Tutorial on Fisher information. *J Math Psychol* 2017; **80**: 40–55.

35 GBD 2020, Release 1, Vaccine Coverage Collaborators. Measuring routine childhood vaccination coverage in 204 countries and territories, 1980-2019: a systematic analysis for the Global Burden of Disease Study 2020, Release 1. *Lancet Lond Engl* 2021; **398**: 503–21.

36 Preziosi M, Department of Immunization, Vaccines, and Biologicals (World Health Organization). Update on meningococcal A vaccine development and introduction. Presented at: WHO Product Development for Vaccines Advisory Committee Meeting (PD-VAC). Geneva, Switzerland: WHO, 2015.

37 GBD Diarrhoeal Diseases Collaborators. Estimates of global, regional, and national morbidity, mortality, and aetiologies of diarrhoeal diseases: a systematic analysis for the Global Burden of Disease Study 2015. *Lancet Infect Dis* 2017; **17**: 909–48.

Section 10: Appendix tables and figures

Table S1: Data inputs by source type.

Table 1. Data inputs by source type

Source type	Number of study-GBD location-years	Sample size	Sample size units	Estimation step used
Multiple cause of death (MCoD)	2980	120,871,372	Deaths	1. Sepsis<br>2. Infectious syndrome
Hospital discharge	391	192,533,415	Discharges	1. Sepsis<br>2. Infectious syndrome<br>3. Case fatality rate<br>4. Pathogen distribution
Microbial or laboratory data with outcome	1448	3,038,363	Isolates	3. Case fatality rate<br>4. Pathogen distribution
Microbial or laboratory data without outcome	3767	16,251,075	Isolates	4. Pathogen distribution
Literature studies	811	803,175	Cases, isolates, or pathogen-drug susceptibility tests	3. Case fatality rate<br>4. Pathogen distribution
Mortality surveillance (Minimally invasive tissue sampling [MITS] from Child Health and Mortality Prevention Surveillance [CHAMPS])	29	2,163	Deaths	1. Sepsis<br>2. Infectious syndrome<br>4. Pathogen distribution
Linkage (mortality only)	38	264,010	Deaths	1. Sepsis<br>2. Infectious syndrome<br>4. Pathogen distribution
**Grand Total**	**11,361**	**342,564,954**		

Table S2: Number and ASMR per 100 000 by bacterial pathogen and GBD region, 2019

Number and age-standardised mortality rate per 100 000 (ASMR) by bacterial pathogen and GBD region, all ages, 2019 (95% uncertainty intervals in parenthesis)

Pathogen	Central Asia		Central Europe		Eastern Europe		Australasia		High-income Asia Pacific		High-income North America		South Latin America	
	Deaths	ASMR	Deaths	ASMR	Deaths	ASMR	Deaths	ASMR	Deaths	ASMR	Deaths	ASMR	Deaths	ASMR
S. aureus	12,000 (9,000-17,000)	17.8 (12.5-25.0)	23,000 (16,000-33,000)	11.2 (7.5-16.1)	37,000 (24,000-52,000)	11.5 (7.6-16.5)	5,000 (4,000-7,000)	10.1 (6.8-14.5)	68,000 (53,000-86,000)	12.5 (9.6-16.3)	81,000 (57,000-113,000)	12.7 (8.7-17.9)	18,000 (15,000-22,000)	21.4 (17.2-26.5)
E. coli	10,000 (7,000-14,000)	14.9 (10.0-21.2)	27,000 (17,000-41,000)	12.6 (7.8-19.3)	48,000 (30,000-72,000)	14.7 (9.3-22.1)	3,000 (2,000-4,000)	6.0 (4.1-8.5)	31,000 (22,000-43,000)	5.9 (4.1-8.2)	54,000 (38,000-74,000)	8.3 (5.8-11.5)	12,000 (9,000-15,000)	14.0 (10.6-18.3)
S. pneumoniae	8,000 (7,000-10,000)	10.5 (8.3-13.4)	10,000 (8,000-14,000)	5.4 (4.0-7.4)	20,000 (15,000-27,000)	6.9 (5.2-9.0)	1,000 (1,000-2,000)	2.5 (1.9-3.3)	22,000 (18,000-26,000)	4.0 (3.4-4.7)	24,000 (19,000-31,000)	3.9 (3.0-5.1)	9,000 (7,000-10,000)	10.4 (9.0-12.2)
K. pneumoniae	9,000 (6,000-12,000)	12.3 (8.3-17.7)	13,000 (8,000-19,000)	6.2 (4.0-9.2)	24,000 (16,000-35,000)	7.5 (4.8-10.8)	2,000 (1,000-3,000)	3.5 (2.3-5.0)	20,000 (15,000-27,000)	3.8 (2.8-5.2)	33,000 (23,000-47,000)	5.2 (3.5-7.4)	9,000 (7,000-12,000)	10.8 (8.1-14.1)
P. aeruginosa	6,000 (4,000-9,000)	9.4 (6.2-13.8)	10,000 (7,000-15,000)	4.9 (3.2-7.2)	18,000 (12,000-27,000)	5.6 (3.7-8.3)	2,000 (1,000-3,000)	3.6 (2.4-5.1)	22,000 (17,000-29,000)	4.1 (3.1-5.4)	33,000 (22,000-46,000)	5.1 (3.4-7.2)	8,000 (6,000-10,000)	9.2 (6.9-12.1)
A. baumannii	4,000 (2,000-6,000)	5.8 (3.3-9.3)	5,000 (3,000-8,000)	2.2 (1.2-3.8)	8,000 (4,000-13,000)	2.3 (1.2-4.0)	1,000 (1,000-2,000)	2.0 (1.1-3.2)	9,000 (5,000-14,000)	1.8 (1.1-2.8)	17,000 (10,000-27,000)	2.7 (1.6-4.4)	4,000 (2,000-5,000)	4.2 (2.5-6.6)
Enterobacter spp.	3,000 (2,000-4,000)	4.2 (2.6-6.3)	4,000 (3,000-6,000)	2.0 (1.3-3.0)	9,000 (5,000-13,000)	2.7 (1.7-4.0)	1,000 (1,000-1,000)	1.5 (1.0-2.2)	8,000 (5,000-11,000)	1.6 (1.1-2.1)	14,000 (9,000-19,000)	2.2 (1.4-3.2)	3,000 (2,000-4,000)	3.5 (2.5-4.8)
Group B *Streptococcus*	3,000 (2,000-4,000)	3.4 (2.3-4.9)	3,000 (2,000-5,000)	1.6 (1.0-2.5)	7,000 (5,000-11,000)	2.4 (1.6-3.6)	1,000 (1,000-1,000)	1.6 (1.0-2.4)	7,000 (5,000-10,000)	1.5 (1.0-2.1)	12,000 (8,000-18,000)	2.0 (1.3-2.9)	3,000 (2,000-4,000)	3.4 (2.6-4.5)
E. faecalis	3,000 (2,000-4,000)	3.9 (2.3-6.1)	6,000 (3,000-9,000)	2.8 (1.6-4.5)	11,000 (7,000-18,000)	3.6 (2.1-5.6)	1,000 (0-1,000)	1.5 (0.9-2.4)	7,000 (4,000-11,000)	1.4 (0.8-2.3)	13,000 (8,000-20,000)	2.1 (1.3-3.3)	3,000 (2,000-4,000)	3.1 (2.0-4.7)
E. faecium	3,000 (2,000-5,000)	4.2 (2.5-6.7)	6,000 (4,000-9,000)	2.9 (1.8-4.5)	11,000 (7,000-17,000)	3.4 (2.1-5.2)	1,000 (1,000-1,000)	1.9 (1.2-2.9)	9,000 (5,000-13,000)	1.8 (1.2-2.7)	16,000 (10,000-24,000)	2.6 (1.6-3.8)	3,000 (2,000-4,000)	3.3 (2.1-4.9)
Non-typhoidal *Salmonella*	1,000 (0-1,000)	0.7 (0.4-1.1)	1,000 (1,000-2,000)	0.5 (0.3-0.8)	2,000 (1,000-4,000)	0.8 (0.4-1.3)	0 (0-0)	0.1 (0.0-0.1)	0 (0-0)	0.1 (0.0-0.1)	1,000 (0-2,000)	0.2 (0.1-0.4)	0 (0-0)	0.3 (0.2-0.4)
Group A *Streptococcus*	2,000 (1,000-3,000)	2.5 (1.0-5.0)	4,000 (2,000-7,000)	2.0 (1.0-3.7)	9,000 (5,000-16,000)	2.9 (1.6-5.2)	1,000 (0-1,000)	1.5 (0.8-2.5)	4,000 (2,000-8,000)	0.9 (0.5-1.7)	10,000 (5,000-18,000)	1.6 (0.9-2.9)	2,000 (1,000-4,000)	2.9 (1.8-4.6)
Salmonella Typhi	0 (0-0)	0.3 (0.2-0.6)	0 (0-0)	0.1 (0.0-0.2)	1,000 (0-1,000)	0.2 (0.1-0.4)	0 (0-0)	0.0 (0.0-0.0)	0 (0-0)	0.0 (0.0-0.0)	0 (0-0)	0.0 (0.0-0.1)	0 (0-0)	0.1 (0.1-0.2)
N. meningitidis	1,000 (1,000-2,000)	1.1 (0.6-1.7)	1,000 (0-1,000)	0.5 (0.3-0.9)	2,000 (1,000-4,000)	0.9 (0.5-1.5)	0 (0-0)	0.2 (0.1-0.2)	0 (0-1,000)	0.1 (0.1-0.2)	1,000 (1,000-2,000)	0.3 (0.1-0.4)	0 (0-1,000)	0.6 (0.4-0.9)
Campylobacter spp.	0 (0-0)	0.2 (0.1-0.3)	0 (0-0)	0.0 (0.0-0.1)	0 (0-0)	0.0 (0.0-0.1)	0 (0-0)	0.0 (0.0-0.1)	0 (0-0)	0.0 (0.0-0.1)	1,000 (0-2,000)	0.1 (0.0-0.3)	0 (0-0)	0.0 (0.0-0.1)
Shigella spp.	0 (0-0)	0.2 (0.1-0.3)	0 (0-0)	0.0 (0.0-0.1)	0 (0-0)	0.0 (0.0-0.1)	0 (0-0)	0.0 (0.0-0.0)	0 (0-0)	0.0 (0.0-0.0)	0 (0-1,000)	0.0 (0.0-0.1)	0 (0-0)	0.1 (0.0-0.2)
Proteus spp.	1,000 (1,000-2,000)	2.0 (1.3-2.9)	3,000 (2,000-4,000)	1.2 (0.7-1.8)	5,000 (3,000-7,000)	1.5 (0.9-2.1)	0 (0-0)	0.7 (0.5-0.9)	3,000 (2,000-4,000)	0.6 (0.4-0.8)	6,000 (4,000-8,000)	0.9 (0.7-1.3)	2,000 (1,000-2,000)	1.8 (1.3-2.3)
H. influenzae	1,000 (1,000-1,000)	1.2 (1.0-1.5)	1,000 (1,000-1,000)	0.5 (0.4-0.6)	2,000 (1,000-2,000)	0.6 (0.5-0.8)	0 (0-0)	0.3 (0.2-0.3)	3,000 (2,000-3,000)	0.5 (0.4-0.5)	3,000 (2,000-3,000)	0.4 (0.3-0.5)	1,000 (1,000-1,000)	1.2 (1.1-1.4)
Serratia spp.	1,000 (1,000-2,000)	1.5 (0.9-2.4)	1,000 (1,000-1,000)	0.5 (0.3-0.7)	2,000 (1,000-3,000)	0.6 (0.4-0.9)	0 (0-0)	0.3 (0.2-0.5)	1,000 (1,000-2,000)	0.3 (0.2-0.5)	3,000 (2,000-5,000)	0.5 (0.3-0.8)	1,000 (0-1,000)	0.9 (0.6-1.4)
Other *enterococci*	1,000 (1,000-1,000)	1.6 (1.0-2.3)	2,000 (1,000-3,000)	1.0 (0.5-1.6)	4,000 (2,000-6,000)	1.2 (0.7-1.9)	0 (0-0)	0.7 (0.4-0.9)	3,000 (2,000-4,000)	0.5 (0.4-0.8)	6,000 (4,000-8,000)	0.9 (0.6-1.2)	1,000 (1,000-2,000)	1.6 (1.1-2.1)
V. cholerae	0 (0-0)	0.3 (0.2-0.6)	0 (0-0)	0.0 (0.0-0.0)	0 (0-0)	0.0 (0.0-0.0)	0 (0-0)	0.0 (0.0-0.0)	0 (0-0)	0.0 (0.0-0.0)	0 (0-0)	0.0 (0.0-0.0)	0 (0-0)	0.0 (0.0-0.0)
Chlamydia spp.	1,000 (1,000-1,000)	0.9 (0.7-1.2)	1,000 (1,000-1,000)	0.4 (0.3-0.6)	1,000 (1,000-2,000)	0.4 (0.3-0.6)	0 (0-0)	0.2 (0.1-0.2)	2,000 (2,000-2,000)	0.4 (0.3-0.4)	2,000 (2,000-3,000)	0.3 (0.2-0.4)	1,000 (1,000-1,000)	1.0 (0.8-1.2)
Mycoplasma spp.	1,000 (1,000-1,000)	1.2 (1.0-1.5)	1,000 (1,000-2,000)	0.7 (0.6-0.9)	2,000 (2,000-3,000)	0.8 (0.7-1.0)	0 (0-0)	0.4 (0.3-0.5)	4,000 (3,000-4,000)	0.6 (0.5-0.7)	3,000 (3,000-4,000)	0.5 (0.4-0.7)	1,000 (1,000-1,000)	1.1 (1.0-1.3)

Pathogen	Western Europe		Andean Latin America		Caribbean		Central Latin America		Tropical Latin America		North Africa and Middle East		South Asia	
	Deaths	ASMR	Deaths	ASMR	Deaths	ASMR	Deaths	ASMR	Deaths	ASMR	Deaths	ASMR	Deaths	ASMR
S. aureus	110,000 (82,000-147,000)	10.8 (7.8-14.8)	11,000 (9,000-15,000)	20.9 (15.7-27.2)	7,000 (5,000-9,000)	13.7 (9.8-18.8)	34,000 (24,000-48,000)	14.8 (10.4-20.7)	43,000 (33,000-54,000)	18.9 (14.9-23.9)	57,000 (40,000-80,000)	14.4 (10.2-20.0)	185,000 (134,000-252,000)	14.3 (10.4-19.5)
E. coli	109,000 (73,000-157,000)	10.7 (7.1-15.4)	7,000 (5,000-10,000)	13.5 (9.4-18.7)	5,000 (4,000-8,000)	11.2 (7.8-15.5)	30,000 (21,000-41,000)	13.0 (9.0-18.1)	32,000 (24,000-42,000)	14.3 (10.8-18.5)	45,000 (30,000-64,000)	11.4 (7.6-16.4)	209,000 (154,000-277,000)	16.7 (12.2-22.2)
S. pneumoniae	36,000 (29,000-46,000)	3.6 (2.8-4.7)	7,000 (5,000-9,000)	12.4 (9.7-15.8)	6,000 (5,000-8,000)	13.1 (10.1-16.7)	17,000 (13,000-23,000)	7.7 (5.8-10.0)	22,000 (19,000-27,000)	10.2 (8.5-12.5)	42,000 (32,000-54,000)	9.1 (7.0-11.7)	199,000 (157,000-250,000)	14.4 (11.4-18.2)
K. pneumoniae	44,000 (31,000-61,000)	4.4 (3.0-6.1)	7,000 (5,000-10,000)	12.7 (8.9-17.4)	5,000 (4,000-7,000)	11.1 (7.7-15.6)	23,000 (16,000-33,000)	10.3 (7.0-14.5)	26,000 (19,000-35,000)	11.6 (8.6-15.5)	43,000 (29,000-61,000)	10.3 (7.0-14.9)	191,000 (135,000-260,000)	14.5 (10.2-20.0)
P. aeruginosa	40,000 (29,000-54,000)	3.9 (2.8-5.3)	5,000 (4,000-7,000)	9.4 (6.7-13.2)	4,000 (3,000-6,000)	8.1 (5.5-11.4)	18,000 (12,000-26,000)	8.0 (5.3-11.4)	20,000 (15,000-27,000)	9.0 (6.6-12.2)	31,000 (21,000-45,000)	7.8 (5.2-11.2)	120,000 (82,000-167,000)	9.3 (6.3-13.1)
A. baumannii	11,000 (6,000-19,000)	1.1 (0.6-1.9)	3,000 (2,000-4,000)	4.8 (2.8-7.5)	4,000 (2,000-6,000)	8.1 (4.8-12.7)	11,000 (6,000-17,000)	4.8 (2.7-7.4)	11,000 (6,000-17,000)	4.8 (2.9-7.5)	25,000 (15,000-40,000)	6.6 (3.8-10.2)	122,000 (73,000-193,000)	10.2 (6.1-16.0)
Enterobacter spp.	13,000 (9,000-18,000)	1.3 (0.9-1.9)	2,000 (1,000-3,000)	3.8 (2.5-5.4)	3,000 (2,000-4,000)	5.3 (3.4-7.7)	9,000 (6,000-13,000)	4.0 (2.5-5.7)	8,000 (6,000-11,000)	3.6 (2.6-5.0)	18,000 (11,000-28,000)	4.4 (2.8-6.6)	75,000 (48,000-110,000)	5.6 (3.6-8.2)
Group B *Streptococcus*	13,000 (9,000-19,000)	1.4 (0.9-2.0)	2,000 (1,000-3,000)	3.6 (2.6-4.9)	2,000 (1,000-3,000)	4.8 (3.3-6.6)	7,000 (5,000-10,000)	3.1 (2.1-4.4)	8,000 (6,000-10,000)	3.5 (2.6-4.8)	16,000 (11,000-23,000)	3.5 (2.3-5.0)	73,000 (53,000-98,000)	5.2 (3.7-7.1)
E. faecalis	21,000 (13,000-33,000)	2.1 (1.3-3.3)	2,000 (1,000-3,000)	3.2 (1.9-5.1)	1,000 (1,000-2,000)	3.0 (1.8-4.6)	8,000 (5,000-13,000)	3.5 (2.2-5.5)	7,000 (5,000-11,000)	3.3 (2.1-4.7)	12,000 (7,000-19,000)	2.9 (1.6-4.7)	47,000 (29,000-71,000)	3.5 (2.1-5.3)
E. faecium	22,000 (14,000-32,000)	2.3 (1.5-3.4)	2,000 (1,000-3,000)	3.4 (2.0-5.1)	1,000 (1,000-2,000)	2.8 (1.7-4.3)	9,000 (5,000-13,000)	3.8 (2.3-5.6)	7,000 (5,000-11,000)	3.2 (2.1-4.7)	13,000 (7,000-20,000)	3.2 (1.9-5.1)	40,000 (24,000-63,000)	3.0 (1.8-4.8)
Non-typhoidal *Salmonella*	1,000 (1,000-2,000)	0.2 (0.1-0.3)	0 (0-1,000)	0.6 (0.4-0.9)	1,000 (1,000-2,000)	2.4 (1.5-3.6)	2,000 (1,000-4,000)	0.8 (0.4-1.6)	1,000 (1,000-2,000)	0.5 (0.3-0.8)	7,000 (5,000-11,000)	1.4 (0.9-2.0)	49,000 (28,000-82,000)	3.6 (1.9-6.5)
Group A *Streptococcus*	16,000 (9,000-27,000)	1.7 (1.0-2.8)	1,000 (1,000-2,000)	2.1 (1.1-3.8)	2,000 (1,000-3,000)	3.8 (2.2-6.4)	7,000 (4,000-12,000)	2.9 (1.6-5.1)	7,000 (4,000-12,000)	3.3 (2.0-5.2)	11,000 (5,000-20,000)	2.6 (1.2-5.0)	44,000 (23,000-79,000)	3.5 (1.8-6.4)
Salmonella Typhi	0 (0-1,000)	0.0 (0.0-0.1)	0 (0-0)	0.5 (0.3-0.7)	0 (0-1,000)	1.1 (0.7-1.6)	1,000 (0-1,000)	0.3 (0.2-0.5)	1,000 (0-1,000)	0.3 (0.2-0.5)	5,000 (3,000-8,000)	0.9 (0.6-1.3)	95,000 (54,000-150,000)	5.3 (3.2-8.3)
N. meningitidis	1,000 (1,000-2,000)	0.2 (0.1-0.3)	1,000 (0-1,000)	1.1 (0.7-1.7)	1,000 (1,000-2,000)	3.0 (2.0-4.3)	2,000 (1,000-4,000)	1.0 (0.6-1.5)	2,000 (1,000-3,000)	1.1 (0.7-1.7)	8,000 (5,000-12,000)	1.4 (0.8-2.2)	41,000 (27,000-61,000)	2.6 (1.7-3.9)
Campylobacter spp.	1,000 (0-2,000)	0.1 (0.0-0.2)	0 (0-1,000)	0.6 (0.2-1.3)	0 (0-0)	0.2 (0.1-0.6)	1,000 (0-3,000)	0.6 (0.2-1.2)	1,000 (0-1,000)	0.3 (0.1-0.6)	1,000 (0-2,000)	0.2 (0.1-0.4)	64,000 (19,000-154,000)	5.9 (1.5-15.0)
Shigella spp.	0 (0-0)	0.0 (0.0-0.0)	0 (0-0)	0.2 (0.1-0.4)	0 (0-0)	0.4 (0.1-1.0)	1,000 (0-2,000)	0.4 (0.2-0.8)	1,000 (0-1,000)	0.4 (0.2-0.7)	2,000 (1,000-5,000)	0.5 (0.2-1.0)	34,000 (13,000-74,000)	3.0 (1.0-6.9)
Proteus spp.	9,000 (6,000-12,000)	0.9 (0.6-1.2)	1,000 (1,000-1,000)	1.7 (1.1-2.5)	1,000 (1,000-1,000)	1.5 (1.0-2.3)	4,000 (3,000-6,000)	1.8 (1.2-2.5)	4,000 (3,000-6,000)	2.0 (1.5-2.6)	6,000 (3,000-9,000)	1.5 (0.9-2.3)	27,000 (17,000-39,000)	2.2 (1.4-3.2)
H. influenzae	4,000 (3,000-4,000)	0.3 (0.3-0.4)	1,000 (1,000-1,000)	1.5 (1.2-1.9)	1,000 (1,000-1,000)	1.6 (1.2-2.1)	2,000 (1,000-3,000)	0.8 (0.6-1.1)	3,000 (2,000-3,000)	1.2 (1.0-1.5)	5,000 (4,000-7,000)	1.1 (0.8-1.4)	25,000 (20,000-31,000)	1.8 (1.4-2.2)
Serratia spp.	2,000 (1,000-3,000)	0.2 (0.2-0.4)	1,000 (0-1,000)	1.2 (0.7-1.8)	1,000 (0-1,000)	1.7 (1.0-2.6)	3,000 (2,000-4,000)	1.3 (0.8-1.9)	3,000 (2,000-4,000)	1.1 (0.7-1.7)	6,000 (3,000-9,000)	1.3 (0.8-2.1)	30,000 (18,000-48,000)	2.3 (1.3-3.5)
Other *enterococci*	7,000 (5,000-11,000)	0.7 (0.5-1.1)	1,000 (0-1,000)	1.2 (0.8-1.8)	1,000 (0-1,000)	1.5 (1.0-2.3)	3,000 (2,000-5,000)	1.4 (1.0-2.1)	4,000 (3,000-5,000)	1.8 (1.3-2.3)	5,000 (3,000-7,000)	1.3 (0.7-1.9)	24,000 (16,000-35,000)	2.0 (1.3-2.9)
V. cholerae	0 (0-0)	0.0 (0.0-0.0)	0 (0-0)	0.0 (0.0-0.0)	1,000 (0-2,000)	1.8 (1.0-3.2)	0 (0-0)	0.0 (0.0-0.1)	0 (0-0)	0.1 (0.0-0.1)	6,000 (4,000-11,000)	1.4 (0.8-2.3)	17,000 (7,000-33,000)	1.2 (0.5-2.5)
Chlamydia spp.	3,000 (2,000-3,000)	0.3 (0.2-0.3)	1,000 (0-1,000)	1.2 (0.9-1.5)	1,000 (1,000-1,000)	1.8 (1.4-2.4)	1,000 (1,000-2,000)	0.7 (0.5-0.9)	2,000 (2,000-2,000)	0.9 (0.8-1.1)	5,000 (3,000-6,000)	1.0 (0.8-1.3)	34,000 (25,000-45,000)	2.4 (1.8-3.3)
Mycoplasma spp.	5,000 (4,000-6,000)	0.5 (0.4-0.6)	1,000 (1,000-1,000)	1.2 (0.9-1.5)	1,000 (1,000-1,000)	1.6 (1.2-2.0)	2,000 (1,000-2,000)	0.8 (0.6-1.0)	2,000 (2,000-3,000)	1.0 (0.9-1.2)	5,000 (4,000-6,000)	1.0 (0.8-1.3)	23,000 (18,000-29,000)	1.5 (1.2-2.0)

Pathogen	East Asia		Oceania		Southeast Asia		Central Sub-Saharan Africa		Eastern Sub-Saharan Africa		Southern Sub-Saharan Africa		Western Sub-Saharan Africa	
	Deaths	ASMR	Deaths	ASMR	Deaths	ASMR	Deaths	ASMR	Deaths	ASMR	Deaths	ASMR	Deaths	ASMR
S. aureus	156,000 (99,000-240,000)	8.8 (5.7-13.4)	2,000 (1,000-2,000)	19.9 (13.8-28.0)	78,000 (58,000-105,000)	14.9 (11.2-19.7)	18,000 (14,000-23,000)	28.5 (21.4-37.2)	63,000 (50,000-79,000)	30.1 (24.4-37.3)	14,000 (11,000-18,000)	26.0 (21.0-32.3)	83,000 (66,000-105,000)	28.4 (22.6-36.2)
E. coli	97,000 (60,000-149,000)	5.4 (3.4-8.3)	1,000 (1,000-2,000)	17.3 (11.8-24.2)	71,000 (51,000-97,000)	13.6 (9.9-18.5)	16,000 (12,000-21,000)	22.7 (16.9-30.3)	55,000 (43,000-71,000)	24.7 (18.9-31.5)	10,000 (7,000-12,000)	17.4 (12.8-23.0)	78,000 (59,000-101,000)	24.2 (18.3-31.2)
S. pneumoniae	83,000 (60,000-117,000)	5.3 (4.0-7.3)	3,000 (2,000-4,000)	26.2 (19.4-34.7)	67,000 (54,000-84,000)	12.8 (10.3-15.8)	24,000 (18,000-30,000)	26.8 (21.2-33.6)	77,000 (63,000-95,000)	25.1 (21.4-29.6)	14,000 (12,000-17,000)	23.5 (20.3-27.7)	136,000 (108,000-169,000)	30.9 (25.6-37.1)
K. pneumoniae	77,000 (49,000-118,000)	4.4 (2.8-6.6)	2,000 (1,000-3,000)	21.2 (14.5-29.6)	65,000 (46,000-90,000)	12.4 (8.8-17.1)	20,000 (15,000-27,000)	27.4 (20.2-36.7)	68,000 (52,000-87,000)	27.4 (21.1-35.0)	12,000 (9,000-16,000)	21.2 (16.2-27.4)	98,000 (75,000-125,000)	27.9 (21.4-36.4)
P. aeruginosa	79,000 (49,000-122,000)	4.5 (2.8-6.8)	1,000 (1,000-2,000)	13.4 (8.6-19.5)	48,000 (33,000-67,000)	9.1 (6.3-12.7)	10,000 (7,000-13,000)	14.3 (10.2-20.0)	32,000 (24,000-42,000)	14.3 (10.6-18.9)	7,000 (6,000-10,000)	13.5 (9.9-18.0)	45,000 (34,000-58,000)	14.3 (10.5-19.0)
A. baumannii	117,000 (65,000-186,000)	6.4 (3.5-10.4)	1,000 (0-1,000)	13.7 (7.6-22.5)	49,000 (29,000-76,000)	9.5 (5.7-14.6)	6,000 (4,000-9,000)	13.0 (7.7-20.2)	20,000 (13,000-30,000)	11.8 (7.3-18.0)	5,000 (3,000-7,000)	9.4 (5.7-14.4)	21,000 (14,000-31,000)	9.6 (5.8-14.6)
Enterobacter spp.	79,000 (46,000-129,000)	4.3 (2.5-7.0)	1,000 (0-1,000)	6.6 (4.1-9.9)	34,000 (22,000-50,000)	6.2 (4.1-9.1)	4,000 (3,000-6,000)	6.3 (4.3-9.3)	14,000 (10,000-19,000)	6.3 (4.4-8.8)	3,000 (2,000-4,000)	5.5 (3.9-7.6)	20,000 (14,000-27,000)	6.5 (4.5-8.9)
Group B *Streptococcus*	33,000 (20,000-52,000)	1.9 (1.2-3.0)	1,000 (1,000-2,000)	11.3 (7.6-16.1)	23,000 (17,000-32,000)	4.5 (3.2-6.1)	12,000 (8,000-15,000)	11.4 (8.3-15.2)	30,000 (23,000-40,000)	9.3 (7.2-12.3)	5,000 (4,000-6,000)	8.3 (6.3-10.9)	57,000 (43,000-75,000)	12.1 (9.4-15.7)
E. faecalis	29,000 (16,000-48,000)	1.5 (0.9-2.5)	0 (0-0)	3.4 (1.9-5.5)	19,000 (12,000-30,000)	3.5 (2.1-5.5)	3,000 (2,000-5,000)	4.7 (2.6-7.4)	11,000 (7,000-16,000)	5.2 (3.0-8.0)	2,000 (1,000-3,000)	3.8 (2.3-5.8)	13,000 (8,000-19,000)	4.9 (2.9-7.5)
E. faecium	35,000 (20,000-56,000)	1.8 (1.1-3.0)	0 (0-0)	3.0 (1.7-4.9)	19,000 (12,000-30,000)	3.5 (2.1-5.3)	2,000 (1,000-4,000)	4.0 (2.3-6.8)	8,000 (5,000-13,000)	4.6 (2.8-7.4)	2,000 (1,000-3,000)	3.5 (2.2-5.4)	10,000 (6,000-15,000)	4.2 (2.4-6.7)
Non-typhoidal *Salmonella*	7,000 (4,000-10,000)	0.4 (0.2-0.6)	0 (0-1,000)	3.9 (2.4-6.0)	11,000 (7,000-18,000)	2.1 (1.3-3.3)	10,000 (7,000-15,000)	8.9 (5.5-13.7)	21,000 (13,000-33,000)	5.4 (3.0-9.8)	3,000 (1,000-6,000)	4.8 (2.1-10.7)	95,000 (55,000-150,000)	18.2 (9.7-29.9)
Group A *Streptococcus*	29,000 (12,000-62,000)	1.7 (0.7-3.4)	0 (0-1,000)	5.2 (2.7-9.4)	18,000 (10,000-33,000)	3.5 (2.0-6.2)	3,000 (2,000-5,000)	5.6 (2.6-10.9)	12,000 (7,000-19,000)	5.6 (2.7-10.3)	3,000 (2,000-5,000)	5.4 (2.8-9.5)	14,000 (9,000-24,000)	5.6 (2.8-10.2)
Salmonella Typhi	1,000 (1,000-2,000)	0.1 (0.0-0.1)	1,000 (0-1,000)	4.6 (2.8-6.9)	15,000 (9,000-25,000)	2.4 (1.4-3.8)	6,000 (4,000-8,000)	4.3 (2.8-6.2)	26,000 (18,000-36,000)	5.3 (3.7-7.3)	1,000 (1,000-2,000)	2.0 (1.3-2.9)	28,000 (19,000-42,000)	5.0 (3.4-7.1)
N. meningitidis	8,000 (5,000-14,000)	0.5 (0.3-0.9)	1,000 (0-1,000)	4.0 (2.5-6.0)	10,000 (7,000-15,000)	1.7 (1.1-2.5)	6,000 (4,000-9,000)	4.7 (3.3-6.4)	22,000 (16,000-30,000)	5.0 (3.7-6.7)	3,000 (2,000-4,000)	3.5 (2.5-4.8)	29,000 (21,000-40,000)	5.4 (3.9-7.4)
Campylobacter spp.	0 (0-1,000)	0.0 (0.0-0.0)	0 (0-1,000)	4.2 (0.9-10.4)	6,000 (1,000-15,000)	1.2 (0.3-3.2)	2,000 (1,000-4,000)	2.5 (0.6-6.2)	21,000 (7,000-43,000)	9.6 (2.5-22.5)	3,000 (1,000-6,000)	4.9 (1.5-11.3)	21,000 (7,000-44,000)	6.3 (1.8-14.2)
Shigella spp.	0 (0-1,000)	0.0 (0.0-0.1)	1,000 (0-1,000)	4.7 (1.9-9.3)	4,000 (1,000-7,000)	0.7 (0.3-1.5)	6,000 (2,000-13,000)	5.5 (2.1-11.2)	15,000 (6,000-29,000)	5.1 (1.9-10.5)	4,000 (2,000-7,000)	6.4 (2.9-12.4)	45,000 (20,000-78,000)	10.1 (4.5-18.5)
Proteus spp.	14,000 (8,000-21,000)	0.8 (0.5-1.2)	0 (0-0)	2.4 (1.5-3.8)	10,000 (7,000-15,000)	2.0 (1.3-3.0)	1,000 (1,000-2,000)	3.0 (1.8-4.9)	5,000 (3,000-8,000)	3.2 (2.0-4.8)	1,000 (1,000-2,000)	2.2 (1.4-3.3)	6,000 (3,000-9,000)	2.9 (1.7-4.4)
H. influenzae	8,000 (5,000-11,000)	0.5 (0.4-0.7)	0 (0-1,000)	3.3 (2.4-4.5)	8,000 (6,000-10,000)	1.5 (1.2-1.8)	3,000 (3,000-4,000)	3.4 (2.6-4.3)	11,000 (8,000-14,000)	3.1 (2.6-3.8)	2,000 (1,000-2,000)	2.8 (2.4-3.4)	20,000 (15,000-25,000)	4.0 (3.2-4.9)
Serratia spp.	15,000 (8,000-24,000)	0.8 (0.4-1.3)	0 (0-0)	2.6 (1.5-4.3)	10,000 (6,000-16,000)	1.8 (1.1-2.8)	2,000 (1,000-3,000)	2.7 (1.6-4.2)	7,000 (5,000-11,000)	2.7 (1.7-4.1)	1,000 (1,000-2,000)	2.3 (1.5-3.5)	9,000 (6,000-14,000)	2.8 (1.8-4.2)
Other *enterococci*	18,000 (10,000-28,000)	1.0 (0.6-1.5)	0 (0-0)	2.2 (1.3-3.5)	9,000 (6,000-13,000)	1.7 (1.1-2.6)	1,000 (1,000-2,000)	1.8 (1.1-3.0)	4,000 (3,000-6,000)	1.8 (1.1-3.0)	1,000 (1,000-1,000)	1.9 (1.1-2.8)	5,000 (3,000-8,000)	1.9 (1.2-3.0)
V. cholerae	0 (0-0)	0.0 (0.0-0.0)	0 (0-0)	1.1 (0.5-2.3)	3,000 (1,000-6,000)	0.5 (0.2-1.1)	7,000 (3,000-14,000)	9.9 (4.2-21.2)	14,000 (7,000-28,000)	6.7 (3.0-14.3)	1,000 (0-3,000)	2.3 (0.8-4.9)	46,000 (21,000-78,000)	15.1 (6.7-27.5)
Chlamydia spp.	10,000 (7,000-14,000)	0.7 (0.5-0.9)	0 (0-0)	2.4 (1.6-3.4)	8,000 (6,000-11,000)	1.6 (1.3-2.2)	2,000 (2,000-3,000)	2.7 (2.0-3.7)	7,000 (5,000-9,000)	2.3 (1.8-3.0)	1,000 (1,000-2,000)	2.2 (1.7-2.7)	12,000 (8,000-16,000)	2.7 (2.1-3.5)
Mycoplasma spp.	13,000 (10,000-18,000)	0.9 (0.7-1.2)	0 (0-0)	1.8 (1.3-2.3)	8,000 (6,000-9,000)	1.4 (1.1-1.7)	2,000 (1,000-2,000)	1.5 (1.1-1.9)	5,000 (4,000-7,000)	1.4 (1.1-1.7)	1,000 (1,000-1,000)	1.8 (1.5-2.2)	10,000 (8,000-13,000)	1.9 (1.5-2.4)

Legionella spp.	1,000 (0-1,000)	0.8 (0.6-1.1)	1,000 (1,000-2,000)	0.6 (0.5-0.8)	1,000 (1,000-1,000)	0.4 (0.3-0.5)	0 (0-0)	0.5 (0.4-0.7)	6,000 (5,000-7,000)	1.1 (0.9-1.2)	4,000 (4,000-6,000)	0.7 (0.6-0.9)	1,000 (1,000-1,000)	1.2 (1.0-1.4)	8,000 (7,000-9,000)	0.7 (0.6-0.9)	1,000 (0-1,000)	1.0 (0.7-1.3)	0 (0-0)	0.8 (0.6-1.0)	1,000 (1,000-2,000)
Citrobacter spp.	1,000 (0-1,000)	0.7 (0.4-1.1)	1,000 (1,000-2,000)	0.5 (0.3-1.0)	2,000 (1,000-4,000)	0.7 (0.3-1.2)	0 (0-0)	0.2 (0.1-0.3)	1,000 (1,000-1,000)	0.2 (0.1-0.3)	2,000 (1,000-3,000)	0.3 (0.2-0.5)	0 (0-1,000)	0.5 (0.4-0.7)	3,000 (2,000-4,000)	0.3 (0.2-0.5)	0 (0-0)	0.6 (0.4-0.9)	0 (0-1,000)	0.9 (0.6-1.4)	2,000 (1,000-2,000)
Other *Klebsiella* species	1,000 (0-1,000)	1.0 (0.5-1.8)	1,000 (1,000-2,000)	0.6 (0.3-0.9)	2,000 (1,000-4,000)	0.8 (0.4-1.3)	0 (0-0)	0.3 (0.2-0.5)	1,000 (1,000-2,000)	0.3 (0.2-0.5)	3,000 (2,000-4,000)	0.4 (0.3-0.7)	1,000 (0-1,000)	0.7 (0.4-1.2)	4,000 (2,000-6,000)	0.4 (0.2-0.6)	1,000 (0-1,000)	0.9 (0.5-1.6)	0 (0-1,000)	0.7 (0.4-1.3)	2,000 (1,000-4,000)
C. difficile	0 (0-0)	0.1 (0.1-0.2)	0 (0-1,000)	0.2 (0.1-0.3)	1,000 (0-1,000)	0.2 (0.1-0.4)	0 (0-0)	0.5 (0.3-0.7)	3,000 (2,000-4,000)	0.5 (0.4-0.7)	10,000 (8,000-12,000)	1.4 (1.1-1.7)	1,000 (0-1,000)	0.6 (0.5-0.9)	8,000 (6,000-10,000)	0.8 (0.6-1.0)	0 (0-0)	0.3 (0.1-0.6)	0 (0-0)	0.2 (0.1-0.3)	1,000 (0-1,000)
Salmonella Paratyphi	0 (0-0)	0.0 (0.0-0.0)	0 (0-0)	0.0 (0.0-0.0)	0 (0-0)	0.0 (0.0-0.0)	0 (0-0)	0.0 (0.0-0.0)	0 (0-0)	0.0 (0.0-0.0)	0 (0-0)	0.0 (0.0-0.0)	0 (0-0)	0.0 (0.0-0.0)	0 (0-0)	0.0 (0.0-0.0)	0 (0-0)	0.0 (0.0-0.0)	0 (0-0)	0.0 (0.0-0.0)	0 (0-0)
Aeromonas spp.	0 (0-0)	0.0 (0.0-0.0)	0 (0-0)	0.0 (0.0-0.0)	0 (0-0)	0.0 (0.0-0.0)	0 (0-0)	0.0 (0.0-0.0)	0 (0-0)	0.0 (0.0-0.0)	0 (0-0)	0.0 (0.0-0.0)	0 (0-0)	0.0 (0.0-0.0)	0 (0-0)	0.0 (0.0-0.0)	0 (0-0)	0.0 (0.0-0.1)	0 (0-0)	0.0 (0.0-0.1)	0 (0-0)
L. monocytogenes	0 (0-0)	0.1 (0.0-0.1)	0 (0-0)	0.0 (0.0-0.1)	0 (0-0)	0.1 (0.0-0.1)	0 (0-0)	0.0 (0.0-0.0)	0 (0-0)	0.0 (0.0-0.0)	0 (0-0)	0.0 (0.0-0.1)	0 (0-0)	0.1 (0.1-0.1)	0 (0-0)	0.0 (0.0-0.0)	0 (0-0)	0.1 (0.0-0.1)	0 (0-0)	0.2 (0.2-0.4)	0 (0-0)
Morganella spp.	0 (0-0)	0.1 (0.1-0.2)	0 (0-0)	0.1 (0.0-0.1)	0 (0-0)	0.1 (0.0-0.1)	0 (0-0)	0.0 (0.0-0.0)	0 (0-0)	0.0 (0.0-0.0)	0 (0-0)	0.0 (0.0-0.1)	0 (0-0)	0.1 (0.1-0.2)	0 (0-1,000)	0.0 (0.0-0.1)	0 (0-0)	0.1 (0.1-0.1)	0 (0-0)	0.1 (0.0-0.1)	0 (0-0)
Providencia spp.	0 (0-0)	0.1 (0.1-0.2)	0 (0-0)	0.0 (0.0-0.0)	0 (0-0)	0.0 (0.0-0.1)	0 (0-0)	0.0 (0.0-0.0)	0 (0-0)	0.0 (0.0-0.0)	0 (0-0)	0.0 (0.0-0.0)	0 (0-0)	0.1 (0.1-0.1)	0 (0-0)	0.0 (0.0-0.0)	0 (0-0)	0.1 (0.0-0.1)	0 (0-0)	0.1 (0.0-0.1)	0 (0-0)
N. gonorrhoeae	0 (0-0)	0.0 (0.0-0.0)	0 (0-0)	0.0 (0.0-0.0)	0 (0-0)	0.0 (0.0-0.0)	0 (0-0)	0.0 (0.0-0.0)	0 (0-0)	0.0 (0.0-0.0)	0 (0-0)	0.0 (0.0-0.0)	0 (0-0)	0.0 (0.0-0.0)	0 (0-0)	0.0 (0.0-0.0)	0 (0-0)	0.0 (0.0-0.0)	0 (0-0)	0.1 (0.0-0.1)	0 (0-0)

Legionella spp.	0.5 (0.4-0.7)	2,000 (2,000-2,000)	0.9 (0.7-1.1)	3,000 (2,000-4,000)	0.7 (0.5-0.9)	10,000 (6,000-15,000)	0.7 (0.4-1.1)	7,000 (5,000-10,000)	0.5 (0.3-0.7)	0 (0-0)	0.5 (0.3-0.7)	3,000 (2,000-4,000)	0.6 (0.4-0.8)	1,000 (0-1,000)	0.7 (0.4-1.0)	2,000 (1,000-4,000)	0.8 (0.5-1.1)	1,000 (0-1,000)	0.9 (0.7-1.2)	3,000 (2,000-6,000)	0.8 (0.5-1.1)
Citrobacter spp.	0.7 (0.4-1.0)	1,000 (1,000-2,000)	0.6 (0.4-0.8)	3,000 (2,000-5,000)	0.7 (0.4-1.1)	14,000 (9,000-22,000)	1.1 (0.7-1.7)	10,000 (6,000-17,000)	0.6 (0.3-0.9)	0 (0-0)	1.0 (0.6-1.6)	6,000 (3,000-9,000)	1.0 (0.6-1.6)	1,000 (0-1,000)	1.0 (0.6-1.6)	3,000 (2,000-4,000)	1.1 (0.7-1.6)	0 (0-1,000)	0.8 (0.5-1.2)	4,000 (2,000-5,000)	1.1 (0.7-1.7)
Other *Klebsiella* species	1.0 (0.5-1.6)	2,000 (1,000-3,000)	0.7 (0.4-1.3)	3,000 (2,000-6,000)	0.8 (0.4-1.4)	13,000 (6,000-23,000)	1.0 (0.5-1.7)	6,000 (3,000-11,000)	0.3 (0.2-0.6)	0 (0-0)	0.7 (0.3-1.4)	6,000 (3,000-10,000)	1.1 (0.5-1.8)	1,000 (0-2,000)	1.7 (0.7-3.2)	3,000 (1,000-6,000)	1.9 (0.8-3.5)	1,000 (0-1,000)	0.9 (0.5-1.7)	3,000 (1,000-6,000)	1.6 (0.7-3.1)
C. difficile	0.3 (0.2-0.5)	1,000 (0-1,000)	0.3 (0.2-0.4)	0 (0-1,000)	0.1 (0.0-0.1)	2,000 (1,000-3,000)	0.1 (0.0-0.2)	4,000 (2,000-9,000)	0.2 (0.1-0.5)	0 (0-0)	0.1 (0.0-0.1)	1,000 (1,000-3,000)	0.2 (0.1-0.4)	0 (0-0)	0.1 (0.0-0.1)	0 (0-1,000)	0.1 (0.0-0.1)	1,000 (0-1,000)	0.8 (0.4-1.5)	1,000 (0-1,000)	0.1 (0.1-0.3)
Salmonella Paratyphi	0.0 (0.0-0.0)	0 (0-0)	0.0 (0.0-0.0)	0 (0-0)	0.0 (0.0-0.0)	22,000 (9,000-42,000)	1.2 (0.5-2.2)	0 (0-0)	0.0 (0.0-0.0)	0 (0-0)	0.1 (0.0-0.2)	1,000 (0-2,000)	0.2 (0.1-0.4)	0 (0-0)	0.0 (0.0-0.0)	0 (0-0)	0.0 (0.0-0.0)	0 (0-0)	0.0 (0.0-0.0)	0 (0-1,000)	0.0 (0.0-0.1)
Aeromonas spp.	0.0 (0.0-0.0)	0 (0-0)	0.0 (0.0-0.1)	0 (0-1,000)	0.1 (0.0-0.2)	8,000 (4,000-14,000)	0.7 (0.3-1.2)	0 (0-0)	0.0 (0.0-0.0)	0 (0-0)	0.2 (0.1-0.4)	0 (0-0)	0.0 (0.0-0.1)	1,000 (0-2,000)	1.0 (0.4-1.8)	4,000 (2,000-7,000)	1.1 (0.5-2.0)	0 (0-1,000)	0.7 (0.3-1.3)	7,000 (3,000-14,000)	1.4 (0.7-2.6)
L. monocytogenes	0.1 (0.0-0.1)	0 (0-0)	0.1 (0.1-0.1)	1,000 (0-1,000)	0.1 (0.1-0.2)	4,000 (2,000-5,000)	0.2 (0.2-0.3)	1,000 (0-1,000)	0.0 (0.0-0.1)	0 (0-0)	0.2 (0.2-0.4)	1,000 (0-1,000)	0.1 (0.1-0.2)	1,000 (0-1,000)	0.5 (0.3-0.7)	2,000 (2,000-3,000)	0.8 (0.6-1.1)	0 (0-0)	0.4 (0.3-0.5)	5,000 (3,000-8,000)	1.1 (0.7-1.6)
Morganella spp.	0.1 (0.1-0.1)	0 (0-0)	0.2 (0.1-0.2)	0 (0-0)	0.1 (0.0-0.1)	2,000 (1,000-2,000)	0.1 (0.1-0.2)	1,000 (0-1,000)	0.0 (0.0-0.1)	0 (0-0)	0.2 (0.1-0.3)	1,000 (0-1,000)	0.1 (0.1-0.2)	0 (0-0)	0.1 (0.0-0.1)	0 (0-0)	0.1 (0.0-0.1)	0 (0-0)	0.1 (0.0-0.1)	0 (0-0)	0.1 (0.0-0.1)
Providencia spp.	0.1 (0.0-0.1)	0 (0-0)	0.1 (0.1-0.2)	0 (0-0)	0.1 (0.0-0.1)	2,000 (1,000-3,000)	0.2 (0.1-0.3)	0 (0-1,000)	0.0 (0.0-0.0)	0 (0-0)	0.2 (0.1-0.4)	1,000 (0-1,000)	0.1 (0.1-0.2)	0 (0-0)	0.1 (0.1-0.2)	0 (0-0)	0.1 (0.1-0.2)	0 (0-0)	0.1 (0.0-0.1)	0 (0-0)	0.1 (0.1-0.2)
N. gonorrhoeae	0.0 (0.0-0.0)	0 (0-0)	0.0 (0.0-0.0)	0 (0-0)	0.0 (0.0-0.0)	1,000 (1,000-2,000)	0.1 (0.1-0.1)	0 (0-0)	0.0 (0.0-0.0)	0 (0-0)	0.0 (0.0-0.1)	0 (0-0)	0.0 (0.0-0.0)	0 (0-0)	0.1 (0.1-0.2)	0 (0-0)	0.1 (0.1-0.1)	0 (0-0)	0.1 (0.1-0.1)	0 (0-0)	0.0 (0.0-0.1)

Note: Rows are sorted from largest to lowest global mortality rate.

Table S3: YLLs by pathogen and infectious syndrome in 2019.

Pathogen	Bloodstream infections	Cardiac infections	Meningitis and other bacterial central nervous system infections	Diarrhoea	LRI and all related infections of the thorax	Bacterial infections of the skin and subcutaneous systems	Urinary tract infections and pyelonephritis	Typhoid, paratyphoid, and invasive non-typhoidal Salmonella	Gonorrhoea and chlamydia	Peritoneal and intra-abdominal infections	Infections of bones, joints, and related organs
Acinetobacter baumannii	7,300,000 (4,260,000-11,200,000)	259,000 (185,000-369,000)	--	--	4,310,000 (2,520,000-6,770,000)	244,000 (15,500-1,070,000)	405,000 (158,000-795,000)	--	--	--	--
Aeromonas spp.	--	--	--	1,410,000 (601,000-2,710,000)	--	--	--	--	--	--	--
Campylobacter spp.	--	--	--	5,540,000 (2,190,000-10,900,000)	--	--	--	--	--	--	--
Chlamydia spp.	--	--	--	--	4,740,000 (3,480,000-6,360,000)	--	--	--	35,200 (27,300-40,600)	--	--
Citrobacter spp.	1,410,000 (837,000-2,220,000)	--	--	--	--	--	102,000 (69,400-152,000)	--	--	429,000 (241,000-687,000)	--
Clostridioides difficile	--	--	--	871,000 (615,000-1,240,000)	--	--	--	--	--	--	--
Enterobacter spp.	5,990,000 (3,770,000-8,940,000)	152,000 (107,000-221,000)	--	--	2,570,000 (1,820,000-3,530,000)	184,000 (56,200-444,000)	281,000 (180,000-437,000)	--	--	1,960,000 (1,180,000-3,060,000)	43,000 (11,500-98,700)
Enterococcus faecalis	3,000,000 (1,950,000-4,430,000)	71,700 (52,500-98,800)	--	--	--	167,000 (13,500-728,000)	443,000 (364,000-546,000)	--	--	3,180,000 (1,770,000-5,020,000)	56,400 (16,300-137,000)
Enterococcus faecium	2,470,000 (1,490,000-3,760,000)	99,900 (72,000-140,000)	--	--	--	--	368,000 (192,000-633,000)	--	--	3,030,000 (1,760,000-4,940,000)	14,700 (4,110-38,200)
Escherichia coli	6,070,000 (3,630,000-9,270,000)	304,000 (216,000-426,000)	1,510,000 (1,040,000-2,230,000)	2,590,000 (1,420,000-4,310,000)	9,700,000 (7,200,000-12,800,000)	390,000 (133,000-864,000)	2,400,000 (1,920,000-3,130,000)	--	--	7,390,000 (4,590,000-11,100,000)	76,500 (21,700-181,000)
Group A *Streptococcus*	3,430,000 (2,240,000-5,160,000)	97,200 (71,800-137,000)	--	--	--	2,730,000 (1,140,000-5,640,000)	--	--	--	--	145,000 (42,000-340,000)
Group B *Streptococcus*	2,910,000 (1,800,000-4,330,000)	105,000 (76,900-144,000)	1,340,000 (969,000-1,870,000)	--	11,500,000 (8,510,000-15,200,000)	492,000 (122,000-1,340,000)	243,000 (182,000-328,000)	--	--	--	76,900 (19,100-192,000)
Haemophilus influenzae	--	--	679,000 (489,000-957,000)	--	4,920,000 (3,880,000-6,260,000)	--	--	--	--	--	--
Klebsiella pneumoniae	10,700,000 (6,840,000-15,500,000)	261,000 (194,000-361,000)	2,190,000 (1,520,000-3,150,000)	--	13,500,000 (10,600,000-17,100,000)	148,000 (23,600-532,000)	734,000 (505,000-1,060,000)	--	--	3,860,000 (2,420,000-5,820,000)	37,400 (10,400-90,200)
Legionella spp.	--	--	--	--	1,950,000 (1,240,000-3,130,000)	--	--	--	--	--	--
Listeria monocytogenes	--	--	903,000 (596,000-1,320,000)	--	--	--	--	--	--	--	--
Morganella spp.	--	--	--	--	--	--	107,000 (68,200-163,000)	--	--	--	--
Mycoplasma spp.	--	--	--	--	4,550,000 (3,650,000-5,710,000)	--	--	--	--	--	--
Neisseria gonorrhoeae	--	--	--	--	--	--	--	--	104,000 (80,900-119,000)	--	--
Neisseria meningitidis	7,240,000 (4,530,000-11,100,000)	--	2,000,000 (1,530,000-2,670,000)	--	--	--	--	--	--	--	--
Non-typhoidal *Salmonella*	4,760,000 (3,090,000-7,100,000)	86,900 (64,200-121,000)	--	2,880,000 (277,000-8,060,000)	--	--	--	6,120,000 (3,320,000-9,710,000)	--	--	--
Other *Klebsiella* species	--	--	--	--	--	--	--	--	--	1,550,000 (797,000-2,760,000)	--
Other enterococci	2,090,000 (1,300,000-3,190,000)	55,200 (38,700-78,200)	--	--	--	263,000 (86,500-626,000)	494,000 (356,000-692,000)	--	--	--	--
Proteus spp.	1,050,000 (627,000-1,640,000)	38,500 (27,800-53,800)	--	--	--	226,000 (73,000-556,000)	450,000 (341,000-609,000)	--	--	878,000 (495,000-1,420,000)	--
Providencia spp.	--	--	--	--	--	--	116,000 (73,200-181,000)	--	--	--	--
Pseudomonas aeruginosa	5,970,000 (3,770,000-8,910,000)	169,000 (126,000-233,000)	--	--	9,140,000 (7,140,000-11,600,000)	424,000 (132,000-1,030,000)	547,000 (312,000-918,000)	--	--	2,620,000 (1,590,000-3,970,000)	42,300 (9,940-109,000)
Salmonella Paratyphi	--	--	--	--	--	--	--	1,630,000 (673,000-3,190,000)	--	--	--
Salmonella Typhi	5,100,000 (3,170,000-7,540,000)	67,100 (48,200-95,100)	--	--	--	--	--	7,970,000 (3,780,000-13,800,000)	--	--	--
Serratia spp.	3,340,000 (2,130,000-5,100,000)	71,500 (52,400-99,300)	--	--	--	--	100,000 (59,600-163,000)	--	--	489,000 (279,000-780,000)	--
Shigella spp.	--	--	--	7,010,000 (3,150,000-12,400,000)	--	--	--	--	--	--	--
Staphylococcus aureus	8,470,000 (5,090,000-12,900,000)	388,000 (277,000-543,000)	1,110,000 (798,000-1,560,000)	--	17,600,000 (14,400,000-21,600,000)	1,110,000 (503,000-2,410,000)	486,000 (342,000-714,000)	--	--	4,910,000 (2,900,000-7,490,000)	232,000 (68,400-542,000)

Streptococcus pneumoniae	4,960,000 (3,070,000-7,410,000)	165,000 (122,000-229,000)	2,720,000 (2,080,000-3,630,000)	--	32,500,000 (27,100,000-39,500,000)	--	--	--	--	--	--
Vibrio cholerae	--	--	--	5,240,000 (2,950,000-8,390,000)	--	--	--	--	--	--	--

Table S4: Deaths by pathogen and the top 3 leading infectious syndromes by GBD super-region in 2019.

**Pathogen**	**Southeast Asia, East Asia, and Oceania**			**Central Europe, Eastern Europe, and Central Asia**			**High-income**			**Latin America and Caribbean**			**North Africa and Middle East**			**South Asia**			**Sub-Saharan Africa**		
	**BSI**	**LRI and thorax infections**	**Intra-abdominal infections**	**BSI**	**LRI and thorax infections**	**Intra-abdominal infections**	**BSI**	**LRI and thorax infections**	**Intra-abdominal infections**	**BSI**	**LRI and thorax infections**	**Intra-abdominal infections**	**BSI**	**LRI and thorax infections**	**Intra-abdominal infections**	**BSI**	**LRI and thorax infections**	**Intra-abdominal infections**	**BSI**	**LRI and thorax infections**	**Intra-abdominal infections**
A. baumannii	121,000 (65,100-201,000)	32,800 (17,200-55,700)	--	7,940 (3,830-14,500)	5,010 (2,460-8,570)	--	23,600 (12,400-40,000)	10,100 (5,520-16,900)	--	12,600 (6,870-20,600)	12,700 (6,890-21,300)	--	13,900 (7,480-22,400)	9,280 (4,810-15,300)	--	51,600 (27,900-84,900)	63,100 (34,600-104,000)	--	16,900 (10,900-25,100)	33,200 (19,900-51,600)	--
Chlamydia spp.	--	18,700 (14,200-25,100)	--	--	2,800 (2,240-3,600)	--	--	7,840 (6,480-9,350)	--	--	4,920 (3,910-6,230)	--	--	4,550 (3,420-5,960)	--	--	33,400 (24,500-44,300)	--	--	22,100 (16,100-29,800)	--
Citrobacter spp.	11,000 (5,960-18,400)	--	3,760 (2,210-5,960)	1,930 (648-4,350)	--	1,350 (826-2,010)	2,050 (962-3,730)	--	2,810 (1,840-4,150)	1,500 (850-2,390)	--	1,510 (937-2,290)	1,780 (975-2,920)	--	976 (540-1,570)	9,370 (5,100-15,400)	--	3,700 (1,980-6,430)	5,060 (2,980-8,050)	--	2,160 (1,070-3,860)
Enterobacter spp.	75,200 (41,900-123,000)	12,700 (8,230-19,200)	17,400 (10,500-26,500)	3,640 (1,900-6,430)	3,820 (2,500-5,770)	6,150 (3,840-9,100)	8,790 (4,800-14,500)	10,800 (7,770-14,700)	13,000 (8,490-18,900)	6,800 (3,980-10,500)	5,470 (3,740-7,720)	7,040 (4,540-10,300)	8,960 (5,060-14,100)	3,350 (2,250-4,940)	4,550 (2,610-7,090)	38,900 (22,900-60,500)	12,800 (8,690-18,300)	17,300 (10,100-27,300)	13,700 (9,090-19,800)	15,800 (10,900-22,400)	10,100 (5,450-16,300)
E. faecalis	15,300 (8,610-24,900)	--	26,900 (13,900-46,400)	6,660 (3,560-11,400)	--	10,300 (5,470-17,200)	11,500 (6,120-19,500)	--	22,300 (10,900-41,500)	5,100 (2,930-8,140)	--	10,500 (5,750-17,000)	3,940 (2,240-6,190)	--	6,570 (3,370-11,300)	17,200 (9,890-27,200)	--	23,500 (12,200-39,100)	15,000 (10,100-21,600)	--	12,800 (6,400-21,700)
E. faecium	20,400 (11,200-33,300)	--	28,400 (17,000-45,700)	7,160 (3,800-12,000)	--	10,800 (6,650-16,100)	18,900 (10,200-31,700)	--	24,900 (16,400-36,300)	6,250 (3,470-10,300)	--	11,000 (6,940-16,400)	4,490 (2,450-7,360)	--	6,990 (4,040-11,200)	12,300 (6,870-20,100)	--	23,400 (12,900-40,400)	8,770 (5,620-13,000)	--	12,600 (6,620-22,100)
E. coli	41,900 (22,800-69,200)	24,600 (18,800-32,400)	69,000 (42,600-107,000)	38,300 (20,100-65,700)	5,640 (4,320-7,400)	25,700 (16,500-37,300)	76,200 (40,100-130,000)	17,100 (14,000-20,800)	61,600 (40,000-90,000)	17,400 (9,420-29,000)	11,500 (9,140-14,600)	27,000 (18,400-38,400)	11,400 (6,230-18,700)	9,010 (6,720-12,000)	17,100 (10,500-26,300)	33,400 (18,100-54,800)	48,400 (36,300-64,200)	58,000 (36,300-84,500)	23,600 (15,100-35,000)	64,600 (48,900-84,700)	31,600 (20,200-46,000)
Group A *Streptococcus*	10,200 (5,920-16,200)	--	--	4,780 (2,560-7,940)	--	--	6,350 (3,410-10,600)	--	--	4,070 (2,440-6,270)	--	--	3,570 (2,030-5,760)	--	--	11,800 (7,140-18,500)	--	--	15,600 (10,500-22,700)	--	--
Group B *Streptococcus*	22,800 (12,600-37,300)	22,800 (17,300-30,500)	--	4,850 (2,580-8,130)	4,690 (3,590-6,190)	--	16,200 (8,670-27,000)	8,590 (7,050-10,400)	--	5,970 (3,400-9,470)	8,410 (6,520-10,800)	--	4,640 (2,580-7,460)	8,630 (6,170-11,700)	--	11,800 (6,860-18,400)	48,600 (35,200-65,400)	--	9,590 (6,450-13,800)	79,900 (59,600-106,000)	--
H. influenzae	--	14,600 (11,300-19,100)	--	--	3,410 (2,730-4,380)	--	--	9,880 (8,180-11,900)	--	--	5,840 (4,660-7,280)	--	--	4,660 (3,580-5,960)	--	--	22,700 (17,800-28,700)	--	--	30,100 (23,600-38,900)	--
Klebsiella pneumoniae	53,100 (29,700-87,400)	38,900 (29,200-51,400)	36,400 (22,700-55,400)	17,500 (9,370-29,700)	9,300 (7,090-12,500)	12,700 (8,240-18,500)	34,100 (18,300-57,200)	28,000 (22,700-34,400)	28,100 (18,500-41,100)	20,500 (11,700-32,900)	19,300 (15,100-24,500)	14,800 (9,810-21,200)	15,500 (8,790-24,500)	13,700 (10,400-17,900)	9,560 (5,880-14,500)	64,800 (37,200-103,000)	70,600 (53,700-90,700)	36,000 (22,800-55,100)	59,300 (39,500-85,200)	95,700 (76,200-119,000)	20,300 (13,000-31,200)
Legionella spp.	--	10,500 (7,730-14,300)	--	--	2,890 (2,330-3,700)	--	--	20,200 (16,900-23,500)	--	--	4,010 (3,170-5,090)	--	--	2,930 (2,110-4,220)	--	--	9,520 (5,730-15,400)	--	--	6,370 (3,750-10,900)	--
Mycoplasma spp.	--	21,000 (16,500-27,000)	--	--	4,510 (3,800-5,410)	--	--	12,800 (10,800-15,000)	--	--	5,390 (4,410-6,580)	--	--	5,100 (4,030-6,400)	--	--	22,600 (17,800-28,800)	--	--	18,000 (14,100-23,100)	--
N. meningitidis	16,500 (9,380-26,000)	--	--	3,650 (1,950-6,070)	--	--	2,930 (1,560-4,730)	--	--	5,670 (3,320-8,710)	--	--	6,420 (3,550-10,400)	--	--	31,300 (18,000-50,300)	--	--	43,400 (28,100-64,400)	--	--

Non-typhoidal *Salmonella*	13,300 (7,640-21,200)	--	--	3,370 (1,790-5,750)	--	--	1,680 (900-2,820)	--	--	3,090 (1,870-4,780)	--	--	3,820 (2,210-6,020)	--	--	32,900 (19,100-51,600)	--	--	29,000 (19,100-42,800)	--	--
Other *Klebsiella* species	--	--	12,100 (6,540-20,900)	--	--	4,320 (2,440-7,140)	--	--	8,340 (4,750-14,000)	--	--	4,940 (2,680-8,190)	--	--	3,170 (1,630-5,570)	--	--	13,200 (6,350-23,100)	--	--	7,780 (3,630-14,200)
Other *enterococci*	17,300 (9,490-28,800)	--	--	3,640 (1,670-6,850)	--	--	7,420 (3,770-13,000)	--	--	3,710 (2,080-5,940)	--	--	3,170 (1,750-5,100)	--	--	13,400 (7,440-22,000)	--	--	8,450 (5,340-13,100)	--	--
Proteus spp.	9,160 (5,030-15,500)	--	8,020 (4,860-12,700)	3,340 (1,730-5,800)	--	2,600 (1,640-3,850)	6,570 (3,440-11,200)	--	5,520 (3,670-7,990)	2,880 (1,530-4,940)	--	3,360 (2,120-5,020)	2,070 (1,140-3,470)	--	2,210 (1,290-3,610)	8,410 (4,420-14,300)	--	9,180 (4,920-15,700)	5,150 (3,180-8,020)	--	5,510 (2,830-9,730)
P. aeruginosa	44,300 (24,400-72,400)	42,400 (29,400-60,300)	25,200 (15,400-38,700)	8,440 (4,460-14,400)	11,500 (8,120-16,500)	9,840 (6,250-14,400)	21,200 (11,400-35,600)	43,100 (33,500-56,000)	25,200 (16,500-36,600)	12,700 (7,200-20,400)	19,700 (14,700-26,400)	9,420 (6,170-13,600)	9,820 (5,420-15,600)	12,300 (9,010-16,800)	5,920 (3,560-9,220)	38,900 (21,800-63,200)	50,300 (37,900-66,100)	17,900 (10,900-28,600)	27,400 (18,100-39,700)	53,600 (42,900-66,600)	9,300 (5,390-14,800)
Salmonella Typhi	3,950 (2,330-6,190)	--	--	904 (389-1,840)	--	--	491 (239-904)	--	--	1,990 (1,210-3,040)	--	--	3,210 (1,880-4,950)	--	--	18,100 (10,500-28,700)	--	--	41,900 (26,700-60,800)	--	--
Serratia spp.	19,700 (11,000-32,300)	--	3,700 (2,240-5,880)	2,210 (1,190-3,810)	--	1,250 (780-1,880)	4,640 (2,530-7,680)	--	2,170 (1,390-3,200)	4,820 (2,770-7,730)	--	1,530 (962-2,280)	4,380 (2,510-7,070)	--	1,010 (578-1,620)	24,000 (13,800-39,300)	--	4,480 (2,380-7,600)	17,000 (10,900-26,000)	--	2,820 (1,420-5,010)
S. aureus	94,500 (52,500-156,000)	80,900 (62,500-105,000)	40,000 (24,000-61,500)	23,900 (12,700-40,700)	26,300 (20,800-34,200)	15,200 (9,360-22,400)	91,800 (48,900-154,000)	137,000 (114,000-163,000)	31,600 (20,500-46,200)	20,600 (11,400-33,800)	50,600 (41,500-62,100)	15,700 (10,100-23,000)	15,600 (8,520-25,400)	26,600 (20,900-34,000)	9,960 (5,910-15,700)	32,000 (18,000-52,200)	93,400 (72,800-120,000)	36,000 (20,800-56,900)	20,400 (13,700-29,700)	117,000 (95,500-143,000)	20,700 (11,900-32,700)
S. pneumoniae	28,600 (15,800-46,500)	120,000 (95,800-150,000)	--	11,300 (6,020-19,100)	25,900 (21,800-31,500)	--	18,800 (10,000-31,500)	70,800 (60,100-82,300)	--	9,270 (5,270-14,800)	42,000 (35,300-50,200)	--	7,320 (4,060-11,900)	32,100 (25,800-39,500)	--	26,900 (15,100-42,800)	160,000 (131,000-197,000)	--	23,200 (15,300-34,000)	203,000 (166,000-248,000)	--

Figure S1: Pathogen distribution fatal and nonfatal estimation.

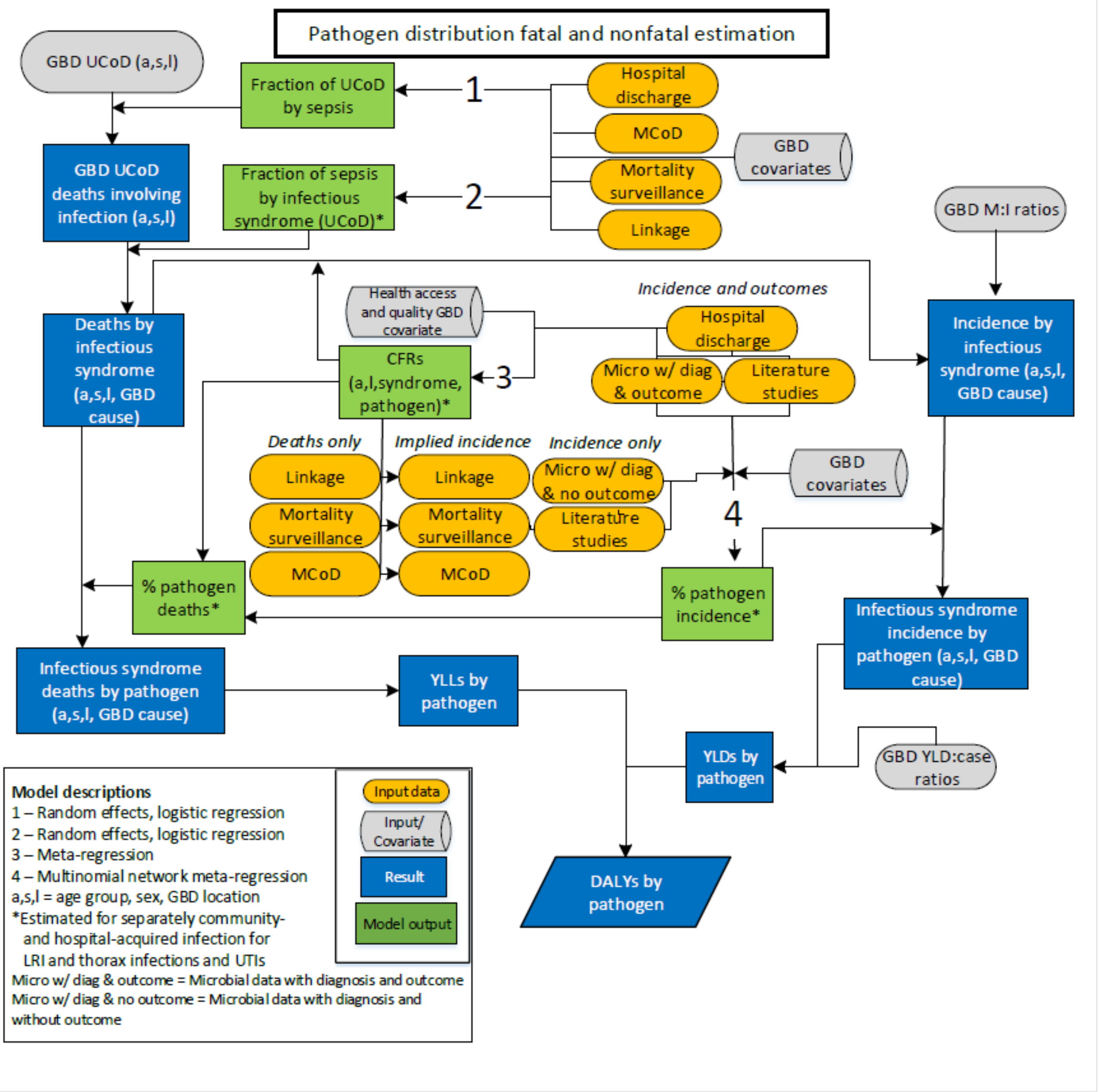

Figure S2: Age-standardised mortality rate per 100 000 population in 2019: *Staphylococcus aureus.*

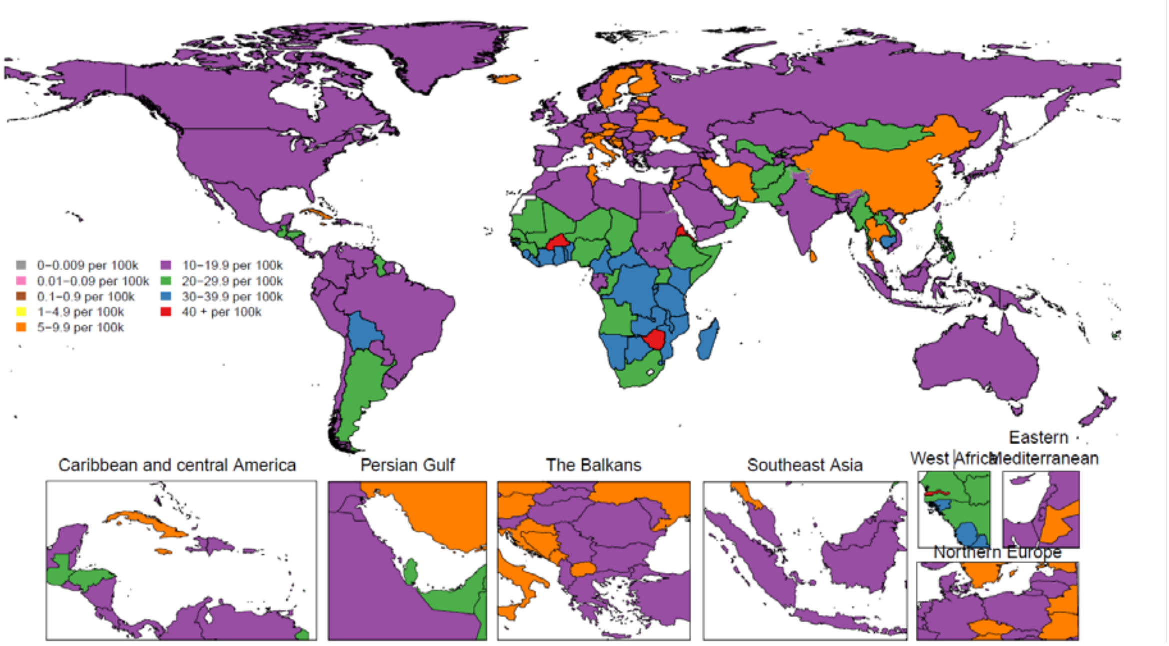

Figure S3: Age-standardised mortality rate per 100 000 population in 2019: *Escherichia coli.*

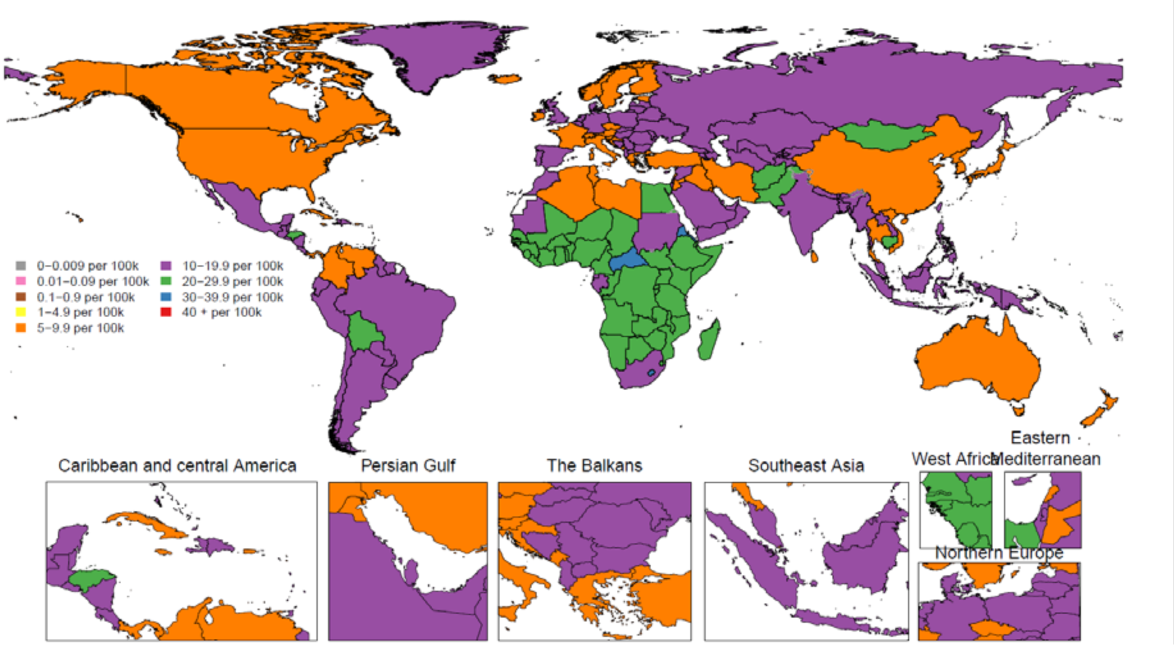

Figure S4: Age−standardised mortality rate per 100 000 population in 2019: *Klebsiella pneumonia.*

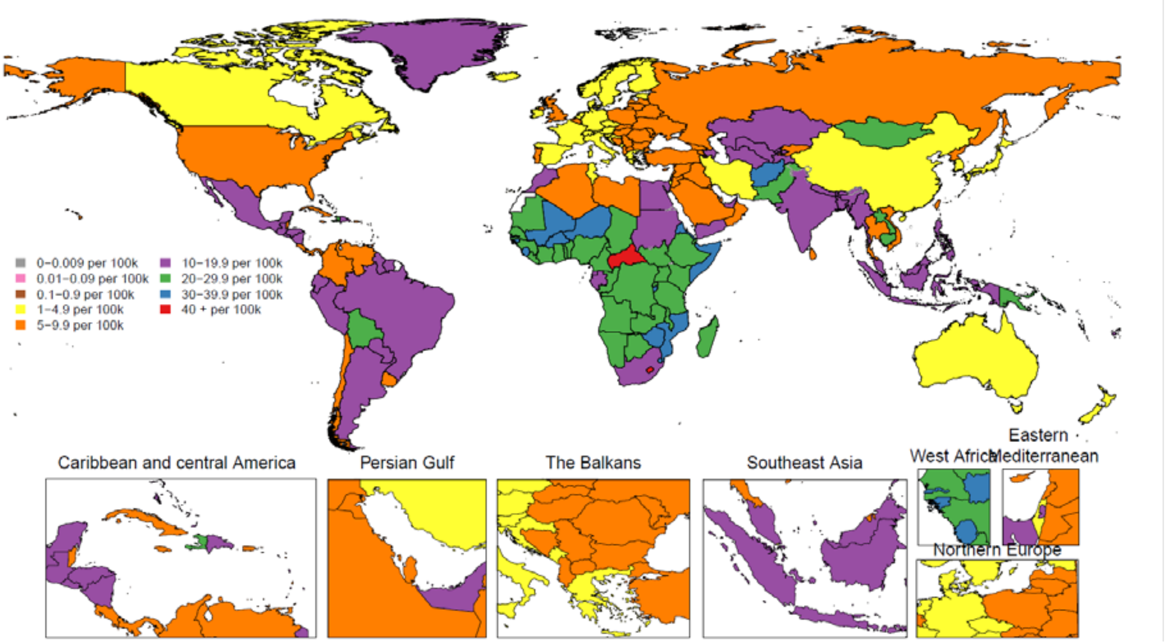

Figure S5: Age−standardised mortality rate per 100 000 population in 2019: *Streptococcus pneumonia.*

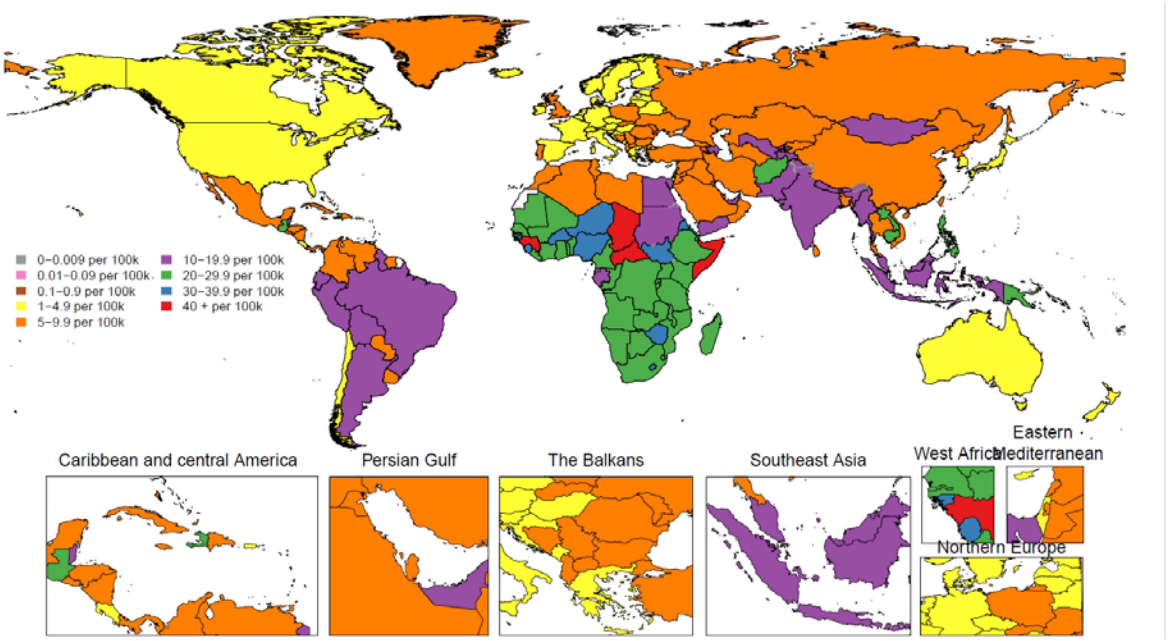

Figure S6: Age−standardised mortality rate per 100 000 population in 2019: *Pseudomonas aeruginosa.*

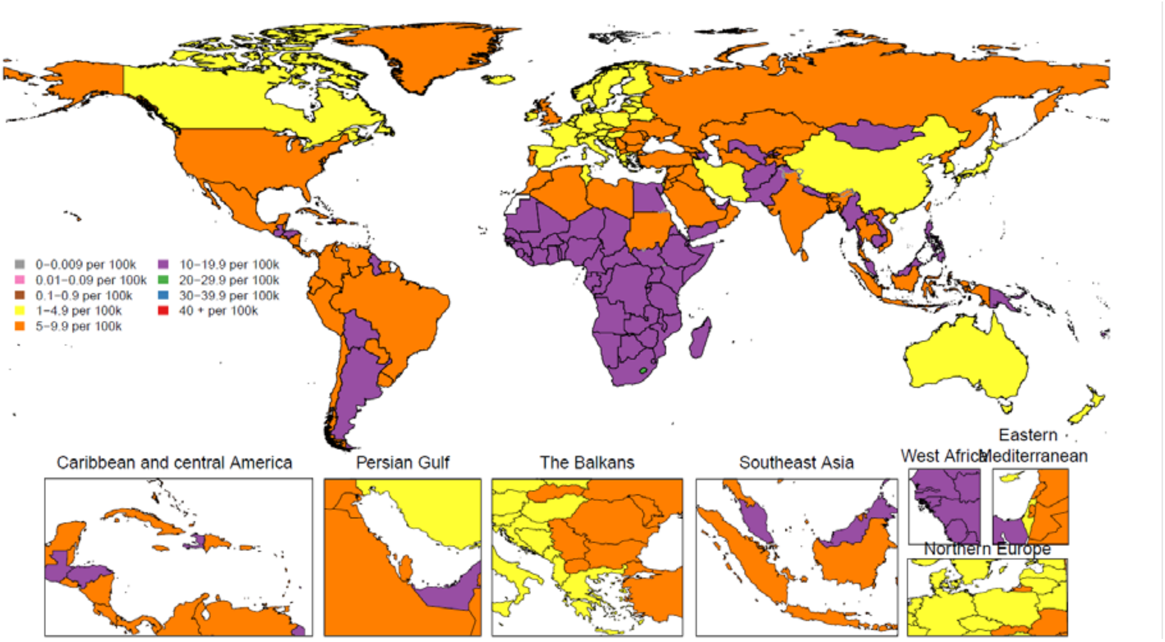

Figure S7: Top 5 Leading BSI pathogens by GBD super−region, 2019.

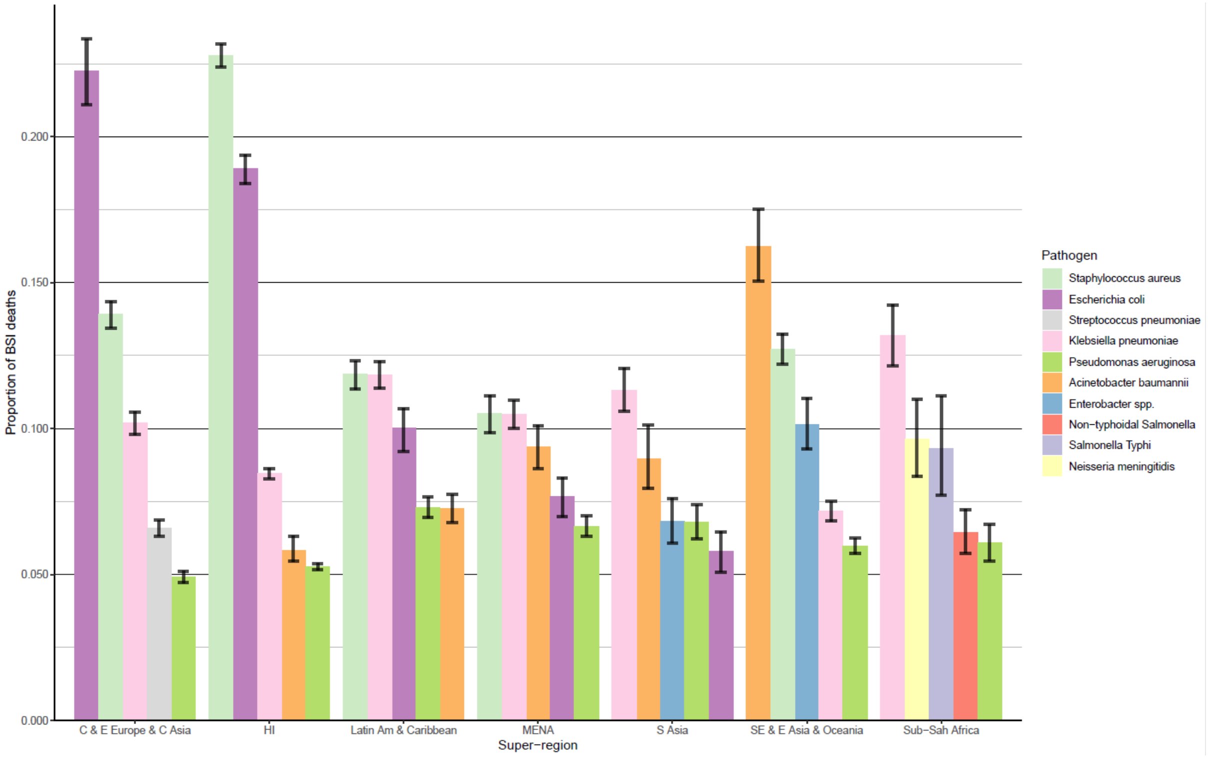

Figure S7. Of all possible 33 pathogens, the top 5 leading pathogens by proportion of deaths in bloodstream infections (BSI) are depicted above for each super region. Across all 7 super-regions, only the pathogens listed in the legend appeared in the top 5 for any given super-region

Figure S8: Top 5 Leading LRI and thorax infections pathogens by GBD super−region, 2019.

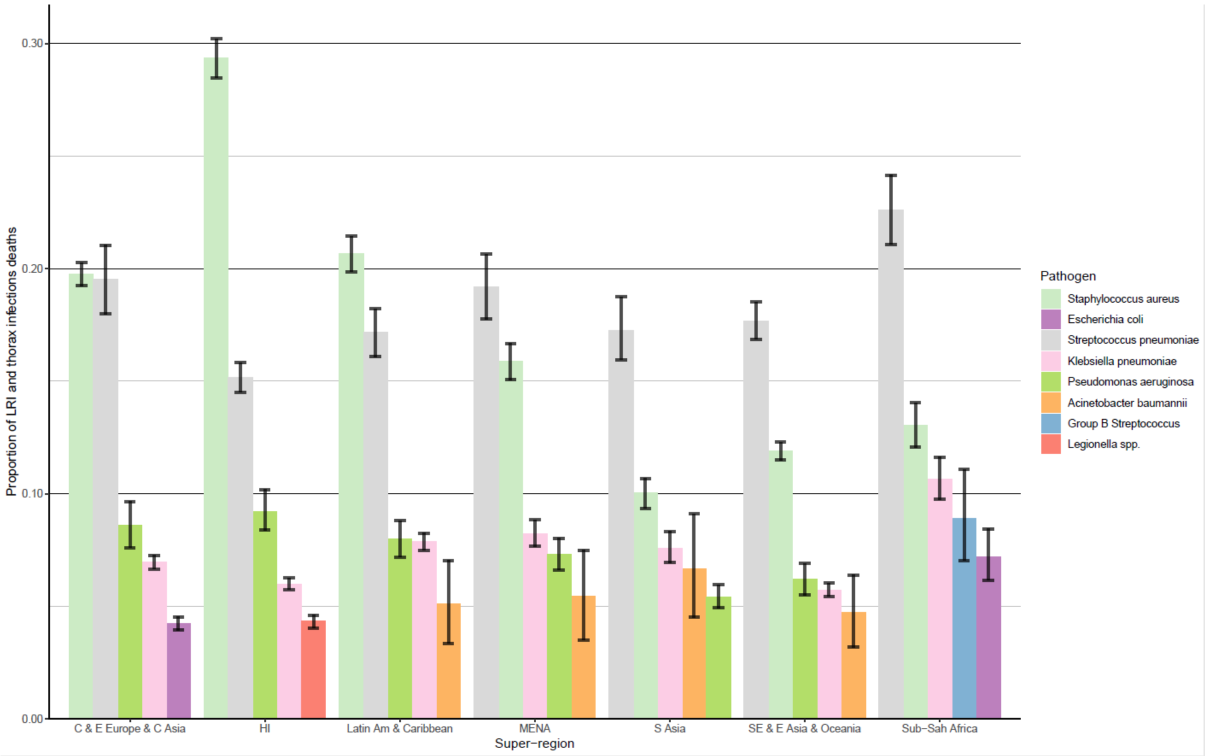

Figure S8. Of all possible 33 pathogens, the top 5 leading pathogens by proportion of deaths in Lower respiratory infections (LRI) and thorax infections are depicted above for each super region. Across all 7 super-regions, only the pathogens listed in the legend appeared in the top 5 for any given super-region.

Figure S9: Top 5 Leading Intra–abdominal infections pathogens by GBD super–region, 2019.

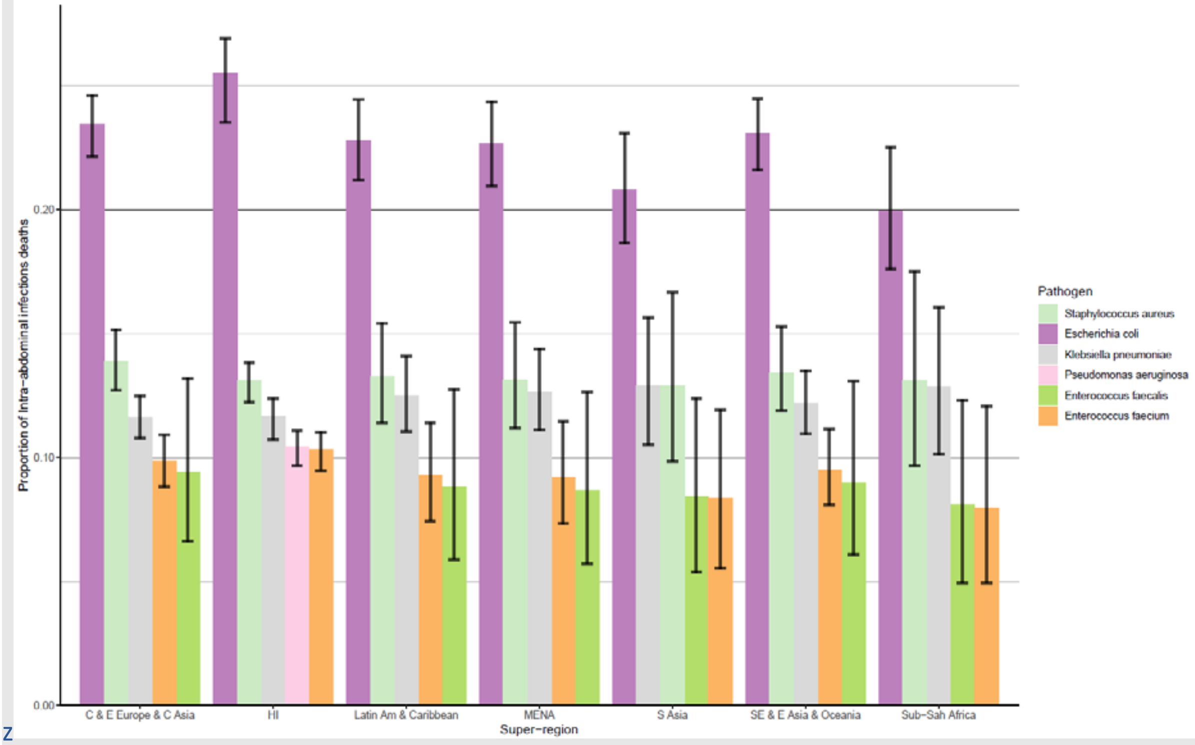

z

Figure S9. Of all possible 33 pathogens, the top 5 leading pathogens by proportion of deaths in intra-abdominal infections are depicted above for each super region. Across all 7 super-regions, only the pathogens listed in the legend appeared in the top 5 of any given super-region.

Figure S10: Global number of deaths by pathogen, age and sex group, 2019.

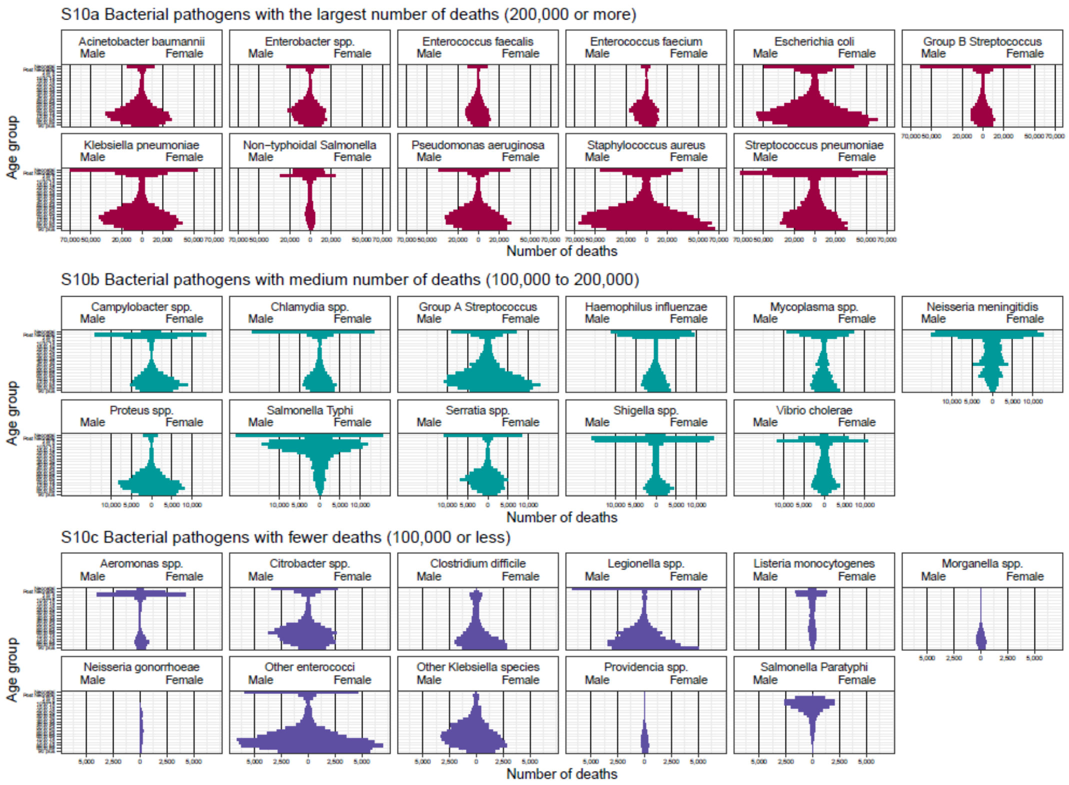

Section 11: Authors' Contributions

Managing the overall research enterprise

Christiane Dolececk, Simon I Hay, Kevin Ikuta, Christopher JL Murray, Mohsen Naghavi, Benn Sartorius, and Eve Wool.

Writing the first draft of the manuscript

Kevin Ikuta, Tomislav Mestrovic, Gisela Robles Aguilar, Lucien Swetschinski, and Nicole Davis Weaver.

Primary responsibility for applying analytical methods to produce estimates

Authia Gray, Chieh Han, Kevin Ikuta, Gisela Robles Aguilar, Lucien Swetschinski, Benn Sartorius, and Fablina Sharara.

Primary responsibility for seeking, cataloguing, extracting, or cleaning data; designing or coding figures and tables

Anna Gershberg Hayoon, Authia Gray, Chieh Han, Gisela Robles Aguilar, and Fablina Sharara.

Providing data or critical feedback on data sources

Semagn Mekonnen Abate, Rizwan Suliankatchi Abdulkader, Aidin Abedi, Hassan Abidi, Hiwa Abubaker Ali, Juan Manuel Acuna, Isaac Yeboah Addo, Qorinah Estiningtyas Sakilah Adnani, Saira Afzal, Gisela Robles Aguilar, Bright Opoku Ahinkorah, Rizwan Ahmad, Sajjad Ahmad, Sohail Ahmad, Sepideh Ahmadi, Haroon Ahmed, Budi Aji, Chisom Joyqueenet Akunna, Hanadi Al Hamad, Fares Alahdab, Beriwan Abdulqadir Ali, Liaqat Ali, Atiyeh Alizadeh, Syed Mohamed Aljunid, Kasim Allel, Sami Almustanyir, Edward Kwabena Ameyaw, Catalina Liliana Andrei, Tudorel Andrei, Jalal Arabloo, Timur Aripov, Anton A Artamonov, Judie Arulappan, Tahira Ashraf, Seyyed Shamsadin Athari, Sameh Attia, Marcel Ausloos, Beatriz Paulina Ayala Quintanilla, Sara Bagherieh, Atif Amin Baig, Luchuo Engelbert Bain, Ahmed Omar Bali, Indrajit Banerjee, Mainak Bardhan, Hiba Jawdat Barqawi, Amadou Barrow, Abdul-Monim Mohammad Batiha, Melaku Ashagrie Belete, Rose G Bender, Dinesh Bhandari, Sonu Bhaskar, Negussie Boti Sidemo, Jiao Cai, Luis Alberto Cámera, Muge Cevik, Akhilanand Chaurasia, Fazle Rabbi Chowdhury, Dinh-Toi Chu, Saswati Das, Fitsum Wolde Demisse, Tigist Demssew Adane, Hardik Dineshbhai Desai, Fikadu Nugusu Dessalegn, Samuel Abebe A Dessalegni, Meghnath Dhimal, Sameer Dhingra, Nancy Diao, Milad Dodangeh, Christiane Dolecek, Fariba Dorostkar, Haneil Larson Dsouza, Susanna J Dunachie, Michael Ekholuenetale, Temitope Cyrus Ekundayo, Hassan El-Abid, Amir Emami, Adeniyi Francis Fagbamigbe, Shahab Falahi, Ali Fatehizadeh, Getahun Fetensa, Peter Andras Gaal, Muktar A Gadanya, Balasankar Ganesan, Reza Ghanbari, Ahmad Ghashghaee, Mahaveer Golechha, Pouya Goleij, Amador Goodridge, Authia P Gray, Rajat Das Gupta, Sapna Gupta, Vivek Kumar Gupta, Chieh Han, Hadi Hassankhani, Simon I Hay, Mohammad Heidari, Reza Heidari-Soureshjani, Claudiu Herteliu, Demisu Zenbaba Heyi, Kamal Hezam, Hmwe Hmwe Kyu, Praveen Hoogar, Nobuyuki Horita, Mehdi Hosseinzadeh, Nawfal R Hussein, Segun Emmanuel Ibitoye, Kevin S Ikuta, Arnaud Iradukunda, Nahlah Elkudssiah Ismail, Mihajlo Jakovljevic, Tahereh Javaheri, Fatemeh Javanmardi, Javad Javidnia, Sathish Kumar Jayapal, Tamas Joo, Farahnaz Joukar, Jacek Jerzy Jozwiak, Vidya Kadashetti, Himal Kandel, Sandhya Neupane Kandel, Samad Karkhah, Bekalu Getnet Kassa, Nicholas J Kassebaum, Himanshu Khajuria, Imteyaz A Khan, Maseer Khan, Md Nuruzzaman Khan, Mona M Khater, Jagdish Khubchandani, Niranjan Kissoon, Farzad Kompani, Soewarta Kosen, Sindhura Lakshmi Koulmane Laxminarayana, Fiorella Krapp Lopez, Kewal Krishan, Vijay Krishnamoorthy, Dharmesh Kumar Lal, Judit Lám, Savita Lasrado, Sang-woong Lee, Michael J Loftus, László Lorenzovici, Mojgan Lotfi, Ata Mahmoodpoor, Alaa Makki, Clara N Matei,

Barney McManigal, Addisu Melese, Oliver Mendoza-Cano, Ritesh G Menezes, Linda Merin J, Tomislav Mestrovic, Erkin M Mirrakhimov, Awoke Misganaw, Mokhtar Mohammadi, Shafiu Mohammed, Syam Mohan, Ali H Mokdad, Lorenzo Monasta, Catrin E Moore, Maryam Moradi, Sumaira Mubarik, Francesk Mulita, Getaneh Baye B Mulu, Christopher J L Murray, Mohsen Naghavi, Himanshi Narang, Entezar Mehrabi Nasab, Zuhair S Natto, Biswa Prakash Nayak, Ionut Negoi, Robina Khan Niazi, Antonio Tolentino Nogueira de Sá, Hasti Nouraei, Chimezie Igwegbe Nzoputam, Bogdan Oancea, Osaretin Christabel Okonji, Andrew T Olagunju, Bolajoko Olubukunola Olusanya, Emad Omer, Bilcha Oumer, Salah Eddine Oussama Kacimi, Mahesh P A, Keyvan Pakshir, Tamás Palicz, Adrian Pana, Shahina Pardhan, Jose L Paredes, Eun-Cheol Park, Uttam Paudel, Shrikant Pawar, Zahra Zahid Piracha, Maarten J Postma, Naeimeh Pourtaheri, Ibrahim Qattea, Mohammad Rabiee, Navid Rabiee, Amir Radfar, Saber Raeghi, Vafa Rahimi-Movaghar, Amir Masoud Rahmani, Pradhum Ram, Sowmya J Rao, Jagadish Rao Padubidri, Tarik Ahmed Rashid, Salman Rawaf, Reza Rawassizadeh, Mohammad Sadegh Razeghinia, Nazila Rezaei, Kristina E Rudd, Basema Saddik, Umar Saeed, Sher Zaman Safi, Maryam Sahebazzamani, Harihar Sahoo, Hedayat Salari, Abdallah M Samy, Benn Sartorius, Brijesh Sathian, Monika Sawhney, Ganesh Kumar Saya, Subramanian Senthilkumaran, Allen Seylani, Masood Ali Shaikh, Murad Ziyaudinovich Shakhmardanov, Purva Sharma, Jae Il Shin, Hesamaddin Shirzad-Aski, K M Shivakumar, Parnian Shobeiri, Sunil Shrestha, Syed Shujait Ali, Luís Manuel Lopes Rodrigues Silva, Jasvinder A Singh, Samarjeet Singh Siwal, Valentin Yurievich Skryabin, Anna Aleksandrovna Skryabina, Chandrashekhar T Sreeramareddy, Muhammad Suleman, Lucien R Swetschinski, Miklós Szócska, Mohammad Tabish, Ker-Kan Tan, Belay Negash Tefera, Yibekal Manaye Tefera, Gebremaryam Temesgen, Mohamad-Hani Temsah, Misganu Teshoma Regasa, Krishna Kishore Umapathi, Era Upadhyay, Sahel Valadan Tahbaz, Pascual R Valdez, Jef Van den Eynde, Georgios-Ioannis Verras, Harimadhav Viswanathan, Bay Vo, Abdul Waris, Sajad Yaghoubi, Seyed Hossein Yahyazadeh Jabbari, Dong Keon Yon, Naohiro Yonemoto, Iman Zare, Zhi-Jiang Zhang, and Mohammad Zoladl.

Developing methods or computational machinery

Richard Gyan Aboagye, Hiwa Abubaker Ali, Qorinah Estiningtyas Sakilah Adnani, Saira Afzal, Gisela Robles Aguilar, Araz Ramazan Ahmad, Ali Ahmed, Liaqat Ali, Aleksandr Y Aravkin, Ahmed Omar Bali, Indrajit Banerjee, Negussie Boti Sidemo, Xiaochen Dai, Hardik Dineshbhai Desai, Christiane Dolecek, Ali Fatehizadeh, Authia P Gray, Chieh Han, Simon I Hay, Mehdi Hosseinzadeh, Kevin S Ikuta, Tahereh Javaheri, Samad Karkhah, Nicholas J Kassebaum, Niranjan Kissoon, Sang-woong Lee, Razzagh Mahmoudi, Alaa Makki, Mokhtar Mohammadi, Ali H Mokdad, Francesk Mulita, Christopher J L Murray, Mohsen Naghavi, Himanshi Narang, Bolajoko Olubukunola Olusanya, Emad Omer, Bilcha Oumer, Saber Raeghi, Amir Masoud Rahmani, Tarik Ahmed Rashid, Reza Rawassizadeh, Kristina E Rudd, Umar Saeed, Abdallah M Samy, Benn Sartorius, Ganesh Kumar Saya, Fablina Sharara, Syed Shujait Ali, Muhammad Suleman, Lucien R Swetschinski, Belay Negash Tefera, Yibekal Manaye Tefera, Christopher E Troeger, Bay Vo, and Peng Zheng.

Providing critical feedback on methods or results

Amirali Aali, Atta Abbas Naqvi, Sherief Abd-Elsalam, Rizwan Suliankatchi Abdulkader, Sisay Abebe Debela, Aidin Abedi, Amir Parsa Abhari, Hassan Abidi, Richard Gyan Aboagye, Hiwa Abubaker Ali, Juan Manuel Acuna, Isaac Yeboah Addo, Oyelola A Adegboye, Mohammad Adnan, Qorinah Estiningtyas Sakilah Adnani, Muhammad Sohail Afzal, Saira Afzal, Bright Opoku Ahinkorah, Aqeel Ahmad, Araz Ramazan Ahmad, Sajjad Ahmad, Sohail Ahmad, Ali Ahmed, Haroon Ahmed, Jivan Qasim Ahmed, Budi Aji, Mostafa Akbarzadeh-Khiavi, Chisom Joyqueenet Akunna, Hanadi Al Hamad, Fares Alahdab, Ziyad Al-Aly, Mamoon A Aldeyab, Robert Kaba Alhassan, Liaqat Ali, Yousef Alimohamadi, Vahid Alipour, Syed Mohamed Aljunid, Kasim Allel, Sami Almustanyir, Edward Kwabena Ameyaw,

Arianna Maever L Amit, Nivedita Anandavelane, Robert Ancuceanu, Catalina Liliana Andrei, Tudorel Andrei, Adnan Ansar, Anayochukwu Edward Anyasodor, Jalal Arabloo, Demelash Areda, Timur Aripov, Anton A Artamonov, Judie Arulappan, Raphael Taiwo Aruleba, Muhammad Asaduzzaman, Tahira Ashraf, Seyyed Shamsadin Athari, Daniel Atlaw, Sameh Attia, Marcel Ausloos, Tewachew Awoke, Beatriz Paulina Ayala Quintanilla, Tegegn Mulatu Ayana, Sina Azadnajafabad, Darshan B B, Muhammad Badar, Ashish D Badiye, Nayereh Baghcheghi, Sara Bagherieh, Atif Amin Baig, Luchuo Engelbert Bain, Ahmed Omar Bali, Indrajit Banerjee, Aleksandra Barac, Mainak Bardhan, Hiba Jawdat Barqawi, Amadou Barrow, Pritish Baskaran, Saurav Basu, Abdul-Monim Mohammad Batiha, Melaku Ashagrie Belete, Uzma Iqbal Belgaumi, Rose G Bender, Bharti Bhandari, Dinesh Bhandari, Pankaj Bhardwaj, Sonu Bhaskar, Krittika Bhattacharyya, Suraj Bhattarai, Negussie Boti Sidemo, Danilo Buonsenso, Zahid A Butt, Florentino Luciano Caetano dos Santos, Jiao Cai, Paulo Camargos, Rosario Cárdenas, Muge Cevik, Joshua Chadwick, Jaykaran Charan, Akhilanand Chaurasia, Sonali Gajanan Choudhari, Enayet Karim Chowdhury, Dinh-Toi Chu, Isaac Sunday Chukwu, Omid Dadras, Xiaochen Dai, Saswati Das, Anna Dastiridou, Fitsum Wolde Demisse, Solomon Demissie, Tigist Demssew Adane, Msganaw Derese, Hardik Dineshbhai Desai, Fikadu Nugusu Dessalegn, Samuel Abebe A Dessalegni, Belay Desye, Kartik Dhaduk, Meghnath Dhimal, Sameer Dhingra, Nancy Diao, Daniel Diaz, Shirin Djalalinia, Milad Dodangeh, Christiane Dolecek, Deepa Dongarwar, Bezabih Terefe Dora, Fariba Dorostkar, Haneil Larson Dsouza, Susanna J Dunachie, Oyewole Christopher Durojaiye, Hisham Atan Edinur, Habtamu Bekele Ejigu, Michael Ekholuenetale, Temitope Cyrus Ekundayo, Hassan El-Abid, Muhammed Elhadi, Mohamed A Elmonem, Daniel Berhanie Enyew, Babak Eshrati, Farshid Etaee, Adeniyi Francis Fagbamigbe, Shahab Falahi, Aida Fallahzadeh, Emerito Jose A Faraon, Ali Fatehizadeh, Ginenus Fekadu, João C Fernandes, Allegra Ferrari, Getahun Fetensa, Irina Filip, Florian Fischer, Masoud Foroutan, Peter Andras Gaal, Muktar A Gadanya, Abhay Motiramji Gaidhane, Balasankar Ganesan, Mesfin Gebrehiwot, Reza Ghanbari, Ahmad Ghashghaee, Abdolmajid Gholizadeh, Mahaveer Golechha, Davide Golinelli, Ana Carolina Micheletti Gomide Nogueira de Sá, Amador Goodridge, Authia P Gray, Damitha Asanga Gunawardane, Yuming Guo, Rajat Das Gupta, Sapna Gupta, Vivek Kumar Gupta, Alemu Guta, Parham Habibzadeh, Atlas Haddadi Avval, Rabih Halwani, Asif Hanif, Harapan Harapan, Shoaib Hassan, Hadi Hassankhani, Simon I Hay, Khezar Hayat, Behzad Heibati, Golnaz Heidari, Mohammad Heidari, Reza Heidari-Soureshjani, Claudiu Herteliu, Demisu Zenbaba Heyi, Kamal Hezam, Hmwe Hmwe Kyu, Praveen Hoogar, Nobuyuki Horita, Md Mahbub Hossain, Mehdi Hosseinzadeh, Mihaela Hostiuc, Soodabeh Hoveidamanesh, Salman Hussain, Nawfal R Hussein, Segun Emmanuel Ibitoye, Kevin S Ikuta, Olayinka Stephen Ilesanmi, Irena M Ilic, Milena D Ilic, Mohammad Tarique Imam, Mustapha Immurana, Leeberk Raja Inbaraj, Arnaud Iradukunda, Haleh Mousavi Isfahani, Nahlah Elkudssiah Ismail, Chidozie C D Iwu, Chinwe Juliana Iwu, Amirhossein Azari Jafari, Mihajlo Jakovljevic, Elham Jamshidi, Tahereh Javaheri, Javad Javidnia, Sathish Kumar Jayapal, Umesh Jayarajah, Rime Jebai, Ravi Prakash Jha, Tamas Joo, Nitin Joseph, Farahnaz Joukar, Jacek Jerzy Jozwiak, Vidya Kadashetti, Laleh R Kalankesh, Rohollah Kalhor, Vineet Kumar Kamal, Himal Kandel, Sandhya Neupane Kandel, Neeti Kapoor, Samad Karkhah, Hamid Reza Khayat Kashani, Bekalu Getnet Kassa, Nicholas J Kassebaum, Patrick DMC Katoto, Mohammad Keykhaei, Himanshu Khajuria, Abbas Khan, Imteyaz A Khan, Maseer Khan, Moien AB Khan, Moawiah Mohammad Khatatbeh, Mona M Khater, Jagdish Khubchandani, Hanna Kim, Min Seo Kim, Ruth W Kimokoti, Niranjan Kissoon, Sonali Kochhar, Farzad Kompani, Neda Milevska Kostova, Sindhura Lakshmi Koulmane Laxminarayana, Kewal Krishan, Vijay Krishnamoorthy, Vishnutheertha Vishnutheertha Kulkarni, Naveen Kumar, Om P Kurmi, Ambily Kuttikkattu, Dharmesh Kumar Lal, Judit Lám, Savita Lasrado, Sang-woong Lee, Jacopo Lenzi, Sonia Lewycka, Shanshan Li, Stephen S Lim, Wei Liu, Rakesh Lodha, László Lorenzovici, Mojgan Lotfi, Ata Mahmoodpoor, Mansour Adam Mahmoud, Razzagh Mahmoudi, Azeem Majeed, Alaa Makki, Galana Ayana Galana Mamo, Yosef Manla, Miquel Martorell, Clara N Matei, Rahman Md Obaidur, Ravi Mehrotra, Addisu Melese, Oliver Mendoza-

Cano, Ritesh G Menezes, Alexios-Fotios A Mentis, Linda Merin J, Tomislav Mestrovic, Georgia Micha, Irmina Maria Michalek, Shabir Ahmad Mir, Seyyedmohammadsadeq Mirmoeeni, Erkin M Mirrakhimov, Mohammad Mirza-Aghazadeh-Attari, Abay Sisay Misganaw, Sanjeev Misra, Esmaeil Mohammadi, Mokhtar Mohammadi, Abdollah Mohammadian-Hafshejani, Shafiu Mohammed, Syam Mohan, Mohammad Mohseni, Ali H Mokdad, Sara Momtazmanesh, Catrin E Moore, Maryam Moradi, Mostafa Moradi Sarabi, Majid Motaghinejad, Seyed Ali Mousavi-Aghdas, Sumaira Mubarik, Francesk Mulita, Getaneh Baye B Mulu, Sandra B Munro, Christopher J L Murray, Saravanan Muthupandian, Mohsen Naghavi, Tapas Sadasivan Nair, Himanshi Narang, Entezar Mehrabi Nasab, Zuhair S Natto, Muhammad Naveed, Biswa Prakash Nayak, Shumaila Naz, Ionut Negoi, Seyed Aria Nejadghaderi, Che Henry Ngwa, Robina Khan Niazi, Nafise Noroozi, Mohammad Ghasemi Nour, Ali Nowroozi, Jerry John Nutor, Chimezie Igwegbe Nzoputam, Ogochukwu Janet Nzoputam, Bogdan Oancea, Vivek Anand Ojha, Akinkunmi Paul Okekunle, Osaretin Christabel Okonji, Andrew T Olagunju, Bolajoko Olubukunola Olusanya, Emad Omer, Nikita Otstavnov, Bilcha Oumer, Salah Eddine Oussama Kacimi, Mahesh P A, Tamás Palicz, Adrian Pana, Shahina Pardhan, Eun-Cheol Park, Seoyeon Park, Ashish Pathak, Rajan Paudel, Uttam Paudel, Shrikant Pawar, Minjin Peng, Veincent Christian Filipino Pepito, Marcos Pereira, Mario F P Peres, Ionela-Roxana Petcu, Zahra Zahid Piracha, Nayanum Pokhrel, Ramesh Poluru, Maarten J Postma, Naeimeh Pourtaheri, Ibrahim Qattea, Amir Radfar, Saber Raeghi, Sima Rafiei, Pankaja Raghav Raghav, Vafa Rahimi-Movaghar, Mosiur Rahman, Muhammad Aziz Rahman, Amir Masoud Rahmani, Vahid Rahmanian, Pradhum Ram, Muhammad Modassar Ali Nawaz Ranjha, Sowmya J Rao, Jagadish Rao Padubidri, Tarik Ahmed Rashid, Mohammad-Mahdi Rashidi, Azad Rasul, Zubair Ahmed Ratan, Salman Rawaf, Reza Rawassizadeh, Mohammad Sadegh Razeghinia, Melese Abate Reta, Nazila Rezaei, Aziz Rezapour, Abanoub Riad, Rezaul Karim Ripon, Kristina E Rudd, Basema Saddik, Umar Saeed, Azam Safary, Sher Zaman Safi, Maryam Sahebazzamani, Saina Salahi, Sarvenaz Salahi, Hedayat Salari, Sana Salehi, Hossein Samadi Kafil, Abdallah M Samy, Senthilkumar Sankararaman, Francesco Sanmarchi, Benn Sartorius, Brijesh Sathian, Monika Sawhney, Ganesh Kumar Saya, Pritik A Shah, Fablina Sharara, Masood Ali Shaikh, Elaheh Shaker, Murad Ziyaudinovich Shakhmardanov, Mequannent Melaku Sharew, Purva Sharma, Rahim Ali Sheikhi, Ali Sheikhy, Mika Shigematsu, Jae Il Shin, Hesamaddin Shirzad-Aski, K M Shivakumar, Parnian Shobeiri, Seyed Afshin Shorofi, Sunil Shrestha, Syed Shujait Ali, Migbar Mekonnen Sibhat, Mustafa Kamal Sikder, Luís Manuel Lopes Rodrigues Silva, Jasvinder A Singh, Paramdeep Singh, Surjit Singh, Md Shahjahan Siraj, Samarjeet Singh Siwal, Valentin Yurievich Skryabin, Anna Aleksandrovna Skryabina, Bogdan Socea, Damtew Damtew Solomon, Yimeng Song, Chandrashekhar T Sreeramareddy, Muhammad Suleman, Saima Sultana, Lucien R Swetschinski, Miklós Szócska, Seyed-Amir Tabatabaeizadeh, Mohammad Tabish, Majid Taheri, Elahe Taki, Ker-Kan Tan, Belay Negash Tefera, Yibekal Manaye Tefera, Gebremaryam Temesgen, Mohamad-Hani Temsah, Misganu Teshoma Regasa, Arulmani Thiyagarajan, Imad I Tleyjeh, Christopher E Troeger, Krishna Kishore Umapathi, Era Upadhyay, Sahel Valadan Tahbaz, Pascual R Valdez, Jef Van den Eynde, Siavash Vaziri, Georgios-Ioannis Verras, Harimadhav Viswanathan, Bay Vo, Abdul Waris, Gizachew Tadesse Wassie, Nuwan Darshana Wickramasinghe, Sajad Yaghoubi, Seyed Hossein Yahyazadeh Jabbari, Arzu Yigit, Vahit Yiğit, Dong Keon Yon, Naohiro Yonemoto, Mazyar Zahir, Burhan Abdullah Zaman, Sojib Bin Zaman, Mikhail Sergeevich Zastrozhin, Zhi-Jiang Zhang, Peng Zheng, Chenwen Zhong, Mohammad Zoladl, and Alimuddin Zumla.

Drafting the work or revising is critically for important intellectual content

Amirali Aali, Semagn Mekonnen Abate, Mohsen Abbasi-Kangevari, Zeinab Abbasi-Kangevari, Sherief Abd-Elsalam, Getachew Abebe, Aidin Abedi, Hassan Abidi, Abdorrahim Absalan, Juan Manuel Acuna, Oyelola A Adegboye, Mohammad Adnan, Qorinah Estiningtyas Sakilah Adnani, Muhammad Sohail Afzal, Saira Afzal, Zahra Babaei Aghdam, Gisela Robles Aguilar, Bright Opoku Ahinkorah, Araz

Ramazan Ahmad, Rizwan Ahmad, Sohail Ahmad, Sepideh Ahmadi, Jivan Qasim Ahmed, Marjan Ajami, Fares Alahdab, Mamoon A Aldeyab, Alicia V Aleman, Fadwa Alhalaiqa Naji Alhalaiqa, Robert Kaba Alhassan, Liaqat Ali, Kasim Allel, Sami Almustanyir, Nivedita Anandavelane, Robert Ancuceanu, Catalina Liliana Andrei, Tudorel Andrei, Dewi Anggraini, Adnan Ansar, Anayochukwu Edward Anyasodor, Jalal Arabloo, Demelash Areda, Timur Aripov, Judie Arulappan, Raphael Taiwo Aruleba, Muhammad Asaduzzaman, Daniel Atlaw, Marcel Ausloos, Beatriz Paulina Ayala Quintanilla, Tegegn Mulatu Ayana, Sina Azadnajafabad, Muhammad Badar, Ashish D Badiye, Sara Bagherieh, Atif Amin Baig, Indrajit Banerjee, Aleksandra Barac, Mainak Bardhan, Francesco Barone-Adesi, Hiba Jawdat Barqawi, Amadou Barrow, Pritish Baskaran, Saurav Basu, Abdul-Monim Mohammad Batiha, Neeraj Bedi, Melaku Ashagrie Belete, Uzma Iqbal Belgaumi, Bharti Bhandari, Dinesh Bhandari, Sonu Bhaskar, Krittika Bhattacharyya, Saeid Bitaraf, Negussie Boti Sidemo, Danilo Buonsenso, Florentino Luciano Caetano dos Santos, Jiao Cai, Daniela Calina, Muge Cevik, Joshua Chadwick, Akhilanand Chaurasia, Patrick R Ching, Dinh-Toi Chu, Fentaw Teshome Dagnaw, Saswati Das, Nicole Davis Weaver, Solomon Demissie, Diriba Dereje, Msganaw Derese, Hardik Dineshbhai Desai, Fikadu Nugusu Dessalegn, Meghnath Dhimal, Sameer Dhingra, Daniel Diaz, Milad Dodangeh, Christiane Dolecek, Deepa Dongarwar, Bezabih Terefe Dora, Haneil Larson Dsouza, Eleonora Dubljanin, Oyewole Christopher Durojaiye, Hassan El-Abid, Muhammed Elhadi, Mohamed A Elmonem, Amir Emami, Ryenchindorj Erkhembayar, Farshid Etaee, Adeniyi Francis Fagbamigbe, Aida Fallahzadeh, Emerito Jose A Faraon, Ali Fatehizadeh, Ginenus Fekadu, João C Fernandes, Getahun Fetensa, Irina Filip, Florian Fischer, Masoud Foroutan, Peter Andras Gaal, Muktar A Gadanya, Balasankar Ganesan, Anna Gershberg Hayoon, Reza Ghanbari, Ahmad Ghashghaee, Ali Gholamrezanezhad, Davide Golinelli, Ana Carolina Micheletti Gomide Nogueira de Sá, Amador Goodridge, Rajat Das Gupta, Sapna Gupta, Veer Bala Gupta, Vivek Kumar Gupta, Alemu Guta, Parham Habibzadeh, Atlas Haddadi Avval, Rabih Halwani, Md. Abdul Hannan, Harapan Harapan, Simon I Hay, Khezar Hayat, Golnaz Heidari, Claudiu Herteliu, Demisu Zenbaba Heyi, Kamal Hezam, Nobuyuki Horita, Md Mahbub Hossain, Sorin Hostiuc, Junjie Huang, Salman Hussain, Nawfal R Hussein, Segun Emmanuel Ibitoye, Kevin S Ikuta, Olayinka Stephen Ilesanmi, Irena M Ilic, Milena D Ilic, Mustapha Immurana, Arnaud Iradukunda, Nahlah Elkudssiah Ismail, Chidozie C D Iwu, Chinwe Juliana Iwu, Amirhossein Azari Jafari, Mihajlo Jakovljevic, Fatemeh Javanmardi, Sathish Kumar Jayapal, Umesh Jayarajah, Rime Jebai, Ravi Prakash Jha, Tamas Joo, Nitin Joseph, Jacek Jerzy Jozwiak, Vidya Kadashetti, Vineet Kumar Kamal, Himal Kandel, Sandhya Neupane Kandel, Neeti Kapoor, Samad Karkhah, Bekalu Getnet Kassa, Nicholas J Kassebaum, Patrick DMC Katoto, Himanshu Khajuria, Abbas Khan, Imteyaz A Khan, Maseer Khan, Md Nuruzzaman Khan, Moien AB Khan, Amin Mousavi Khaneghah, Moawiah Mohammad Khatatbeh, Mona M Khater, Jagdish Khubchandani, Min Seo Kim, Niranjan Kissoon, Sonali Kochhar, Parvaiz A Koul, Sindhura Lakshmi Koulmane Laxminarayana, Fiorella Krapp Lopez, Kewal Krishan, Vishnutheertha Vishnutheertha Kulkarni, Naveen Kumar, Om P Kurmi, Ambily Kuttikkattu, Judit Lám, Iván Landires, Savita Lasrado, Jacopo Lenzi, Wei Liu, Rakesh Lodha, Michael J Loftus, Ayush Lohiya, László Lorenzovici, Ata Mahmoodpoor, Mansour Adam Mahmoud, Razzagh Mahmoudi, Jamal Majidpoor, Miquel Martorell, Clara N Matei, Oliver Mendoza-Cano, Ritesh G Menezes, Alexios-Fotios A Mentis, Linda Merin J, Tomislav Mestrovic, Georgia Micha, Irmina Maria Michalek, Shabir Ahmad Mir, Mojgan Mirghafourvand, Seyyedmohammadsadeq Mirmoeeni, Mohammad Mirza-Aghazadeh-Attari, Abay Sisay Misganaw, Awoke Misganaw, Esmaeil Mohammadi, Abdollah Mohammadian-Hafshejani, Shafiu Mohammed, Syam Mohan, Ali H Mokdad, Sara Momtazmanesh, Lorenzo Monasta, Catrin E Moore, Maryam Moradi, Mostafa Moradi Sarabi, Shane Douglas Morrison, Majid Motaghinejad, Seyed Ali Mousavi-Aghdas, Sandra B Munro, Christopher J L Murray, Saravanan Muthupandian, Mohsen Naghavi, Tapas Sadasivan Nair, Himanshi Narang, Zuhair S Natto, Biswa Prakash Nayak, Shumaila Naz, Ionut Negoi, Seyed Aria Nejadghaderi, Robina Khan Niazi, Antonio Tolentino Nogueira de Sá, Mohammad Ghasemi Nour, Ali Nowroozi, Virginia Nuñez-Samudio, Jerry

John Nutor, Chimezie Igwegbe Nzoputam, Ogochukwu Janet Nzoputam, Bogdan Oancea, Osaretin Christabel Okonji, Andrew T Olagunju, Bolajoko Olubukunola Olusanya, Nikita Otstavnov, Bilcha Oumer, Salah Eddine Oussama Kacimi, Mahesh P A, Tamás Palicz, Adrian Pana, Shahina Pardhan, Utsav Parekh, Seoyeon Park, Uttam Paudel, Shrikant Pawar, Hamidreza Pazoki Toroudi, Umberto Pensato, Veincent Christian Filipino Pepito, Marcos Pereira, Norberto Perico, Ionela-Roxana Petcu, Zahra Zahid Piracha, Indrashis Podder, Nayanum Pokhrel, Maarten J Postma, Akila Prashant, Ibrahim Qattea, Mohammad Rabiee, Navid Rabiee, Amir Radfar, Saber Raeghi, Pankaja Raghav Raghav, Leila Rahbarnia, Vafa Rahimi-Movaghar, Vahid Rahmanian, Pradhum Ram, Muhammad Modassar Ali Nawaz Ranjha, Sowmya J Rao, Jagadish Rao Padubidri, Zubair Ahmed Ratan, Salman Rawaf, Elrashdy Moustafa Mohamed Redwan, Giuseppe Remuzzi, Abanoub Riad, H. Rogier van Doorn, Kristina E Rudd, Basema Saddik, Saeid Sadeghian, Umar Saeed, Mohsen Safaei, Azam Safary, Sher Zaman Safi, Amirhossein Sahebkar, Harihar Sahoo, Hedayat Salari, Hossein Samadi Kafil, Abdallah M Samy, Nima Sanadgol, Francesco Sanmarchi, Benn Sartorius, Ganesh Kumar Saya, Allen Seylani, Elaheh Shaker, Murad Ziyaudinovich Shakhmardanov, Mequannent Melaku Sharew, Athena Sharifi-Razavi, Ali Sheikhy, Pavanchand H Shetty, Mika Shigematsu, K M Shivakumar, Parnian Shobeiri, Seyed Afshin Shorofi, Sunil Shrestha, Syed Shujait Ali, Luís Manuel Lopes Rodrigues Silva, Jasvinder A Singh, Paramdeep Singh, Surjit Singh, Valentin Yurievich Skryabin, Anna Aleksandrovna Skryabina, Bogdan Socea, Chandrashekhar T Sreeramareddy, Muhammad Suleman, Saima Sultana, Lucien R Swetschinski, Miklós Szócska, Seyed-Amir Tabatabaeizadeh, Mohammad Tabish, Majid Taheri, Ker-Kan Tan, Sarmila Tandukar, Nathan Y Tat, Vivian Y Tat, Belay Negash Tefera, Yibekal Manaye Tefera, Gebremaryam Temesgen, Mohamad-Hani Temsah, Samar Tharwat, Arulmani Thiyagarajan, Imad I Tleyjeh, Krishna Kishore Umapathi, Era Upadhyay, Sahel Valadan Tahbaz, Jef Van den Eynde, Georgios-Ioannis Verras, Gizachew Tadesse Wassie, Nuwan Darshana Wickramasinghe, Sajad Yaghoubi, Seyed Hossein Yahyazadeh Jabbari, Arzu Yigit, Vahit Yiğit, Dong Keon Yon, Naohiro Yonemoto, Mazyar Zahir, Burhan Abdullah Zaman, Sojib Bin Zaman, Moein Zangiabadian, Iman Zare, Mikhail Sergeevich Zastrozhin, Chenwen Zhong, and Mohammad Zoladl.

Managing the estimation or publications process

Saira Afzal, Indrajit Banerjee, Negussie Boti Sidemo, Nicole Davis Weaver, Fikadu Nugusu Dessalegn, Christiane Dolecek, Ali Fatehizadeh, Alemu Guta, Simon I Hay, Kevin S Ikuta, Samad Karkhah, Nicholas J Kassebaum, Ali H Mokdad, Catrin E Moore, Christopher J L Murray, Mohsen Naghavi, Chimezie Igwegbe Nzoputam, Bilcha Oumer, Mahesh P A, Saber Raeghi, Leila Rahbarnia, Abdallah M Samy, Benn Sartorius, Monika Sawhney, Pritik A Shah, Syed Shujait Ali, Lucien R Swetschinski, Belay Negash Tefera, Eve E Wool, Sajad Yaghoubi, and Mikhail Sergeevich Zastrozhin.
