# Supplementary material for: Global mortality associated with 33 bacterial pathogens in 2019: a systematic analysis for the Global Burden of Disease Study 2019
Source: Lancet. 2022 Dec 17;400(10369):2221–48. doi: 10.1016/S0140-6736(22)02185-7 (PMC9763654; doi:10.1016/S0140-6736(22)02185-7)
Supplement: Supplementary appendix 2 [file mmc2.pdf]

THE LANCET

Supplementary appendix 2

This appendix formed part of the original submission and has been peer reviewed. We post it as supplied by the authors.

Supplement to: GBD 2019 Antimicrobial Resistance Collaborators. Global mortality associated with 33 bacterial pathogens in 2019: a systematic analysis for the Global Burden of Disease Study 2019. *Lancet* 2022; published online Nov 21. https://doi.org/10.1016/S0140-6736(22)02185-7.

Appendix 2: supplementary methods to "Global mortality associated with 33 bacterial pathogens in 2019: a systematic analysis for the Global Burden of Disease Study 2019"

Number of deaths and mortality rate per 100 000 by pathogen and country in 2019

Country	Pathogen	Death counts in 2019	Death rate per 100 000 in 2019
Armenia	Acinetobacter baumannii	136 (76 - 220)	3.5 (2.0 - 5.7)
Armenia	Aeromonas spp.	0 (0 - 0)	0.0 (0.0 - 0.0)
Armenia	Campylobacter spp.	2 (0 - 4)	0.1 (0.0 - 0.1)
Armenia	Chlamydia spp.	14 (11 - 19)	0.4 (0.3 - 0.6)
Armenia	Citrobacter spp.	24 (15 - 35)	0.6 (0.4 - 0.9)
Armenia	Clostridioides difficile	6 (3 - 11)	0.2 (0.1 - 0.3)
Armenia	Enterobacter spp.	122 (79 - 174)	3.2 (2.1 - 4.6)
Armenia	Enterococcus faecalis	142 (87 - 215)	3.7 (2.2 - 5.6)
Armenia	Enterococcus faecium	154 (94 - 234)	3.9 (2.4 - 5.9)
Armenia	Escherichia coli	568 (378 - 801)	14.7 (9.9 - 20.6)
Armenia	Group A Streptococcus	73 (31 - 148)	2.0 (0.9 - 4.0)
Armenia	Group B Streptococcus	92 (60 - 138)	2.6 (1.7 - 3.8)
Armenia	Haemophilus influenzae	20 (15 - 26)	0.6 (0.5 - 0.8)
Armenia	Klebsiella pneumoniae	361 (237 - 516)	9.6 (6.4 - 13.6)
Armenia	Legionella spp.	19 (14 - 25)	0.6 (0.4 - 0.8)
Armenia	Listeria monocytogenes	1 (0 - 2)	0.0 (0.0 - 0.1)
Armenia	Morganella spp.	6 (3 - 9)	0.2 (0.1 - 0.2)
Armenia	Mycoplasma spp.	19 (15 - 24)	0.6 (0.5 - 0.8)
Armenia	Neisseria gonorrhoeae	2 (1 - 2)	0.0 (0.0 - 0.1)
Armenia	Neisseria meningitidis	19 (10 - 30)	0.6 (0.4 - 1.0)
Armenia	Non-typhoidal Salmonella	10 (6 - 16)	0.3 (0.2 - 0.4)
Armenia	Other Klebsiella species	32 (17 - 56)	0.8 (0.4 - 1.4)
Armenia	Other enterococci	65 (40 - 92)	1.7 (1.0 - 2.4)
Armenia	Proteus spp.	79 (51 - 110)	2.0 (1.3 - 2.8)
Armenia	Providencia spp.	4 (2 - 6)	0.1 (0.1 - 0.2)
Armenia	Pseudomonas aeruginosa	278 (183 - 407)	7.4 (4.9 - 10.7)
Armenia	Salmonella Paratyphi	0 (0 - 1)	0.0 (0.0 - 0.0)
Armenia	Salmonella Typhi	6 (3 - 9)	0.2 (0.1 - 0.3)
Armenia	Serratia spp.	41 (25 - 63)	1.1 (0.6 - 1.6)
Armenia	Shigella spp.	1 (0 - 3)	0.1 (0.0 - 0.1)
Armenia	Staphylococcus aureus	546 (364 - 779)	14.5 (9.8 - 20.6)

Armenia	Streptococcus pneumoniae	193 (141 - 259)	5.8 (4.3 - 7.6)
Armenia	Vibrio cholerae	0 (0 - 1)	0.0 (0.0 - 0.0)
Azerbaijan	Acinetobacter baumannii	370 (208 - 606)	5.5 (3.0 - 9.0)
Azerbaijan	Aeromonas spp.	1 (0 - 3)	0.0 (0.0 - 0.0)
Azerbaijan	Campylobacter spp.	11 (3 - 29)	0.2 (0.0 - 0.4)
Azerbaijan	Chlamydia spp.	69 (52 - 93)	1.0 (0.8 - 1.4)
Azerbaijan	Citrobacter spp.	55 (34 - 87)	0.7 (0.4 - 1.1)
Azerbaijan	Clostridioides difficile	7 (3 - 14)	0.1 (0.0 - 0.2)
Azerbaijan	Enterobacter spp.	316 (200 - 475)	4.2 (2.7 - 6.3)
Azerbaijan	Enterococcus faecalis	293 (172 - 460)	3.8 (2.2 - 6.2)
Azerbaijan	Enterococcus faecium	333 (198 - 531)	4.3 (2.5 - 6.8)
Azerbaijan	Escherichia coli	1,119 (738 - 1,627)	15.9 (10.5 - 23.3)
Azerbaijan	Group A Streptococcus	181 (76 - 372)	2.5 (1.0 - 5.3)
Azerbaijan	Group B Streptococcus	304 (206 - 435)	4.2 (2.8 - 6.0)
Azerbaijan	Haemophilus influenzae	106 (81 - 138)	1.5 (1.2 - 1.9)
Azerbaijan	Klebsiella pneumoniae	964 (639 - 1,416)	13.7 (9.0 - 20.1)
Azerbaijan	Legionella spp.	58 (38 - 93)	0.8 (0.5 - 1.3)
Azerbaijan	Listeria monocytogenes	6 (4 - 12)	0.1 (0.0 - 0.1)
Azerbaijan	Morganella spp.	9 (5 - 15)	0.1 (0.1 - 0.2)
Azerbaijan	Mycoplasma spp.	100 (76 - 129)	1.3 (1.0 - 1.7)
Azerbaijan	Neisseria gonorrhoeae	3 (2 - 6)	0.0 (0.0 - 0.1)
Azerbaijan	Neisseria meningitidis	92 (50 - 154)	1.1 (0.6 - 1.7)
Azerbaijan	Non-typhoidal Salmonella	50 (28 - 82)	0.7 (0.4 - 1.1)
Azerbaijan	Other Klebsiella species	78 (40 - 136)	1.0 (0.5 - 1.8)
Azerbaijan	Other enterococci	113 (70 - 177)	1.7 (1.0 - 2.7)
Azerbaijan	Proteus spp.	147 (90 - 220)	2.2 (1.3 - 3.3)
Azerbaijan	Providencia spp.	8 (4 - 14)	0.1 (0.1 - 0.2)
Azerbaijan	Pseudomonas aeruginosa	709 (463 - 1,042)	10.1 (6.6 - 14.9)
Azerbaijan	Salmonella Paratyphi	0 (0 - 0)	0.0 (0.0 - 0.0)
Azerbaijan	Salmonella Typhi	31 (16 - 52)	0.4 (0.2 - 0.6)
Azerbaijan	Serratia spp.	121 (71 - 197)	1.6 (0.9 - 2.6)
Azerbaijan	Shigella spp.	10 (2 - 27)	0.1 (0.0 - 0.4)
Azerbaijan	Staphylococcus aureus	1,291 (893 - 1,851)	18.2 (12.7 - 25.8)
Azerbaijan	Streptococcus pneumoniae	850 (645 - 1,121)	11.7 (8.9 - 15.4)
Azerbaijan	Vibrio cholerae	46 (24 - 86)	0.6 (0.3 - 1.1)
Georgia	Acinetobacter baumannii	292 (155 - 480)	4.8 (2.6 - 7.9)
Georgia	Aeromonas spp.	0 (0 - 0)	0.0 (0.0 - 0.0)
Georgia	Campylobacter spp.	2 (1 - 6)	0.1 (0.0 - 0.1)
Georgia	Chlamydia spp.	26 (18 - 37)	0.5 (0.3 - 0.7)
Georgia	Citrobacter spp.	31 (18 - 48)	0.6 (0.3 - 0.9)

Georgia	Clostridioides difficile	5 (2 - 10)	0.1 (0.1 - 0.2)
Georgia	Enterobacter spp.	186 (110 - 281)	3.3 (1.9 - 4.9)
Georgia	Enterococcus faecalis	164 (90 - 263)	2.9 (1.6 - 4.7)
Georgia	Enterococcus faecium	195 (111 - 311)	3.4 (1.9 - 5.4)
Georgia	Escherichia coli	610 (368 - 918)	10.4 (6.2 - 15.5)
Georgia	Group A Streptococcus	119 (48 - 248)	2.1 (1.0 - 4.3)
Georgia	Group B Streptococcus	133 (81 - 209)	2.4 (1.5 - 3.8)
Georgia	Haemophilus influenzae	33 (23 - 47)	0.6 (0.4 - 0.9)
Georgia	Klebsiella pneumoniae	511 (312 - 771)	8.8 (5.4 - 13.2)
Georgia	Legionella spp.	22 (16 - 31)	0.4 (0.3 - 0.6)
Georgia	Listeria monocytogenes	2 (1 - 5)	0.1 (0.0 - 0.1)
Georgia	Morganella spp.	5 (2 - 8)	0.1 (0.0 - 0.1)
Georgia	Mycoplasma spp.	27 (20 - 36)	0.6 (0.4 - 0.7)
Georgia	Neisseria gonorrhoeae	1 (1 - 1)	0.0 (0.0 - 0.0)
Georgia	Neisseria meningitidis	37 (21 - 61)	0.9 (0.5 - 1.5)
Georgia	Non-typhoidal Salmonella	23 (12 - 39)	0.4 (0.3 - 0.7)
Georgia	Other Klebsiella species	43 (22 - 73)	0.8 (0.4 - 1.4)
Georgia	Other enterococci	70 (40 - 115)	1.2 (0.7 - 1.9)
Georgia	Proteus spp.	86 (50 - 134)	1.4 (0.8 - 2.2)
Georgia	Providencia spp.	4 (2 - 7)	0.1 (0.0 - 0.1)
Georgia	Pseudomonas aeruginosa	418 (251 - 646)	7.1 (4.3 - 10.9)
Georgia	Salmonella Paratyphi	1 (0 - 1)	0.0 (0.0 - 0.0)
Georgia	Salmonella Typhi	11 (6 - 19)	0.3 (0.1 - 0.4)
Georgia	Serratia spp.	67 (37 - 108)	1.2 (0.7 - 1.9)
Georgia	Shigella spp.	2 (1 - 3)	0.0 (0.0 - 0.1)
Georgia	Staphylococcus aureus	751 (478 - 1,111)	12.8 (8.1 - 18.9)
Georgia	Streptococcus pneumoniae	320 (224 - 453)	6.0 (4.2 - 8.2)
Georgia	Vibrio cholerae	2 (1 - 5)	0.0 (0.0 - 0.1)
Kazakhstan	Acinetobacter baumannii	751 (421 - 1,226)	5.0 (2.8 - 8.1)
Kazakhstan	Aeromonas spp.	1 (0 - 2)	0.0 (0.0 - 0.0)
Kazakhstan	Campylobacter spp.	8 (2 - 19)	0.1 (0.0 - 0.1)
Kazakhstan	Chlamydia spp.	110 (84 - 143)	0.7 (0.5 - 0.9)
Kazakhstan	Citrobacter spp.	99 (60 - 152)	0.6 (0.4 - 0.9)
Kazakhstan	Clostridioides difficile	38 (18 - 74)	0.2 (0.1 - 0.4)
Kazakhstan	Enterobacter spp.	580 (365 - 871)	3.5 (2.2 - 5.3)
Kazakhstan	Enterococcus faecalis	603 (354 - 921)	3.6 (2.1 - 5.5)
Kazakhstan	Enterococcus faecium	690 (409 - 1,086)	4.1 (2.4 - 6.5)
Kazakhstan	Escherichia coli	2,168 (1,415 - 3,204)	13.6 (8.9 - 20.2)
Kazakhstan	Group A Streptococcus	361 (164 - 709)	2.3 (1.0 - 4.6)
Kazakhstan	Group B Streptococcus	494 (325 - 726)	3.0 (2.0 - 4.5)
Kazakhstan	Haemophilus influenzae	149 (114 - 193)	0.9 (0.7 - 1.2)

Kazakhstan	Klebsiella pneumoniae	1,730 (1,140 - 2,555)	10.9 (7.1 - 16.2)
Kazakhstan	Legionella spp.	149 (113 - 195)	0.9 (0.7 - 1.2)
Kazakhstan	Listeria monocytogenes	13 (8 - 21)	0.1 (0.0 - 0.1)
Kazakhstan	Morganella spp.	14 (8 - 22)	0.1 (0.1 - 0.2)
Kazakhstan	Mycoplasma spp.	162 (131 - 202)	0.9 (0.7 - 1.2)
Kazakhstan	Neisseria gonorrhoeae	10 (8 - 13)	0.1 (0.0 - 0.1)
Kazakhstan	Neisseria meningitidis	152 (86 - 246)	0.8 (0.5 - 1.3)
Kazakhstan	Non-typhoidal Salmonella	58 (34 - 95)	0.4 (0.2 - 0.6)
Kazakhstan	Other Klebsiella species	159 (85 - 277)	0.9 (0.5 - 1.6)
Kazakhstan	Other enterococci	202 (126 - 303)	1.3 (0.8 - 2.0)
Kazakhstan	Proteus spp.	263 (166 - 402)	1.7 (1.1 - 2.6)
Kazakhstan	Providencia spp.	11 (6 - 18)	0.1 (0.0 - 0.1)
Kazakhstan	Pseudomonas aeruginosa	1,410 (911 - 2,055)	8.9 (5.7 - 13.2)
Kazakhstan	Salmonella Paratyphi	1 (0 - 3)	0.0 (0.0 - 0.0)
Kazakhstan	Salmonella Typhi	40 (21 - 67)	0.2 (0.1 - 0.4)
Kazakhstan	Serratia spp.	203 (116 - 323)	1.2 (0.7 - 2.0)
Kazakhstan	Shigella spp.	6 (2 - 14)	0.0 (0.0 - 0.1)
Kazakhstan	Staphylococcus aureus	2,980 (2,076 - 4,259)	18.8 (13.1 - 27.0)
Kazakhstan	Streptococcus pneumoniae	1,355 (1,036 - 1,779)	8.3 (6.3 - 10.9)
Kazakhstan	Vibrio cholerae	6 (3 - 13)	0.0 (0.0 - 0.1)
Kyrgyzstan	Acinetobacter baumannii	174 (93 - 288)	4.2 (2.2 - 6.9)
Kyrgyzstan	Aeromonas spp.	1 (1 - 3)	0.0 (0.0 - 0.0)
Kyrgyzstan	Campylobacter spp.	9 (3 - 19)	0.1 (0.1 - 0.3)
Kyrgyzstan	Chlamydia spp.	27 (19 - 38)	0.5 (0.4 - 0.7)
Kyrgyzstan	Citrobacter spp.	26 (16 - 40)	0.5 (0.3 - 0.8)
Kyrgyzstan	Clostridioides difficile	2 (1 - 4)	0.0 (0.0 - 0.1)
Kyrgyzstan	Enterobacter spp.	144 (89 - 218)	3.0 (1.9 - 4.6)
Kyrgyzstan	Enterococcus faecalis	161 (96 - 244)	3.3 (1.9 - 5.1)
Kyrgyzstan	Enterococcus faecium	163 (94 - 261)	3.4 (2.0 - 5.5)
Kyrgyzstan	Escherichia coli	551 (360 - 804)	11.9 (7.8 - 17.7)
Kyrgyzstan	Group A Streptococcus	86 (41 - 166)	1.9 (0.8 - 3.7)
Kyrgyzstan	Group B Streptococcus	124 (80 - 184)	2.4 (1.5 - 3.6)
Kyrgyzstan	Haemophilus influenzae	43 (32 - 55)	0.8 (0.6 - 1.0)
Kyrgyzstan	Klebsiella pneumoniae	440 (281 - 653)	9.5 (6.0 - 14.3)
Kyrgyzstan	Legionella spp.	26 (16 - 42)	0.5 (0.3 - 0.7)
Kyrgyzstan	Listeria monocytogenes	3 (2 - 6)	0.1 (0.0 - 0.1)
Kyrgyzstan	Morganella spp.	4 (3 - 6)	0.1 (0.1 - 0.2)
Kyrgyzstan	Mycoplasma spp.	39 (31 - 49)	0.6 (0.5 - 0.8)
Kyrgyzstan	Neisseria gonorrhoeae	1 (0 - 1)	0.0 (0.0 - 0.0)
Kyrgyzstan	Neisseria meningitidis	46 (26 - 75)	0.8 (0.4 - 1.2)

Kyrgyzstan	Non-typhoidal Salmonella	22 (13 - 36)	0.4 (0.2 - 0.7)
Kyrgyzstan	Other Klebsiella species	46 (23 - 82)	0.9 (0.5 - 1.6)
Kyrgyzstan	Other enterococci	49 (32 - 73)	1.2 (0.7 - 1.7)
Kyrgyzstan	Proteus spp.	68 (43 - 102)	1.6 (1.0 - 2.4)
Kyrgyzstan	Providencia spp.	4 (2 - 6)	0.1 (0.1 - 0.1)
Kyrgyzstan	Pseudomonas aeruginosa	327 (203 - 489)	7.1 (4.3 - 10.7)
Kyrgyzstan	Salmonella Paratyphi	0 (0 - 0)	0.0 (0.0 - 0.0)
Kyrgyzstan	Salmonella Typhi	17 (9 - 28)	0.3 (0.2 - 0.5)
Kyrgyzstan	Serratia spp.	54 (31 - 87)	1.1 (0.6 - 1.9)
Kyrgyzstan	Shigella spp.	9 (3 - 20)	0.1 (0.0 - 0.3)
Kyrgyzstan	Staphylococcus aureus	615 (417 - 889)	13.1 (8.8 - 19.2)
Kyrgyzstan	Streptococcus pneumoniae	339 (263 - 443)	6.3 (4.7 - 8.4)
Kyrgyzstan	Vibrio cholerae	17 (10 - 30)	0.3 (0.2 - 0.6)
Mongolia	Acinetobacter baumannii	318 (193 - 494)	16.4 (10.0 - 25.6)
Mongolia	Aeromonas spp.	0 (0 - 1)	0.0 (0.0 - 0.0)
Mongolia	Campylobacter spp.	4 (1 - 10)	0.1 (0.0 - 0.3)
Mongolia	Chlamydia spp.	31 (22 - 45)	1.2 (0.8 - 1.7)
Mongolia	Citrobacter spp.	43 (26 - 66)	1.9 (1.2 - 2.9)
Mongolia	Clostridioides difficile	3 (1 - 8)	0.1 (0.0 - 0.3)
Mongolia	Enterobacter spp.	217 (136 - 325)	9.8 (6.2 - 14.6)
Mongolia	Enterococcus faecalis	153 (91 - 236)	7.1 (4.1 - 11.2)
Mongolia	Enterococcus faecium	160 (96 - 252)	7.5 (4.5 - 11.7)
Mongolia	Escherichia coli	446 (286 - 656)	22.0 (14.2 - 31.9)
Mongolia	Group A Streptococcus	92 (43 - 184)	4.3 (1.9 - 8.9)
Mongolia	Group B Streptococcus	102 (66 - 150)	4.3 (2.7 - 6.4)
Mongolia	Haemophilus influenzae	29 (21 - 39)	1.0 (0.7 - 1.4)
Mongolia	Klebsiella pneumoniae	424 (279 - 627)	20.4 (13.3 - 29.9)
Mongolia	Legionella spp.	13 (8 - 21)	0.5 (0.3 - 0.7)
Mongolia	Listeria monocytogenes	2 (1 - 4)	0.1 (0.0 - 0.2)
Mongolia	Morganella spp.	3 (1 - 4)	0.1 (0.1 - 0.2)
Mongolia	Mycoplasma spp.	35 (26 - 47)	1.1 (0.8 - 1.5)
Mongolia	Neisseria gonorrhoeae	1 (0 - 2)	0.0 (0.0 - 0.0)
Mongolia	Neisseria meningitidis	81 (48 - 130)	2.7 (1.6 - 4.3)
Mongolia	Non-typhoidal Salmonella	65 (41 - 101)	2.8 (1.7 - 4.2)
Mongolia	Other Klebsiella species	46 (23 - 85)	2.1 (1.0 - 3.9)
Mongolia	Other enterococci	61 (38 - 92)	3.1 (1.9 - 4.6)
Mongolia	Proteus spp.	72 (44 - 110)	3.8 (2.4 - 5.8)
Mongolia	Providencia spp.	3 (1 - 5)	0.1 (0.1 - 0.3)
Mongolia	Pseudomonas aeruginosa	306 (197 - 453)	14.6 (9.5 - 21.4)
Mongolia	Salmonella Paratyphi	0 (0 - 0)	0.0 (0.0 - 0.0)

Mongolia	Salmonella Typhi	22 (13 - 36)	0.8 (0.5 - 1.3)
Mongolia	Serratia spp.	92 (57 - 143)	4.1 (2.6 - 6.5)
Mongolia	Shigella spp.	4 (1 - 10)	0.1 (0.0 - 0.3)
Mongolia	Staphylococcus aureus	451 (294 - 668)	20.8 (13.6 - 30.5)
Mongolia	Streptococcus pneumoniae	310 (226 - 424)	11.9 (8.5 - 16.5)
Mongolia	Vibrio cholerae	36 (18 - 64)	1.3 (0.6 - 2.6)
Tajikistan	Acinetobacter baumannii	467 (271 - 761)	12.0 (6.8 - 19.6)
Tajikistan	Aeromonas spp.	11 (4 - 23)	0.1 (0.0 - 0.2)
Tajikistan	Campylobacter spp.	68 (21 - 157)	0.7 (0.2 - 1.5)
Tajikistan	Chlamydia spp.	168 (125 - 230)	2.2 (1.7 - 3.0)
Tajikistan	Citrobacter spp.	72 (40 - 120)	1.4 (0.8 - 2.2)
Tajikistan	Clostridioides difficile	2 (1 - 5)	0.0 (0.0 - 0.1)
Tajikistan	Enterobacter spp.	365 (218 - 578)	6.9 (4.0 - 10.8)
Tajikistan	Enterococcus faecalis	195 (115 - 300)	3.8 (2.3 - 6.0)
Tajikistan	Enterococcus faecium	168 (96 - 275)	3.6 (2.1 - 5.8)
Tajikistan	Escherichia coli	728 (528 - 994)	14.4 (10.2 - 20.1)
Tajikistan	Group A Streptococcus	128 (57 - 260)	2.9 (1.1 - 6.4)
Tajikistan	Group B Streptococcus	240 (175 - 336)	3.6 (2.5 - 5.2)
Tajikistan	Haemophilus influenzae	141 (105 - 189)	1.6 (1.2 - 2.1)
Tajikistan	Klebsiella pneumoniae	669 (466 - 947)	12.6 (8.5 - 18.5)
Tajikistan	Legionella spp.	43 (27 - 72)	0.6 (0.4 - 0.9)
Tajikistan	Listeria monocytogenes	10 (6 - 17)	0.1 (0.1 - 0.2)
Tajikistan	Morganella spp.	8 (5 - 12)	0.2 (0.1 - 0.3)
Tajikistan	Mycoplasma spp.	208 (156 - 281)	2.2 (1.7 - 2.9)
Tajikistan	Neisseria gonorrhoeae	5 (4 - 8)	0.1 (0.1 - 0.1)
Tajikistan	Neisseria meningitidis	167 (95 - 282)	1.9 (1.1 - 3.2)
Tajikistan	Non-typhoidal Salmonella	155 (87 - 254)	2.4 (1.3 - 4.0)
Tajikistan	Other Klebsiella species	54 (26 - 97)	1.1 (0.5 - 1.9)
Tajikistan	Other enterococci	96 (62 - 147)	2.3 (1.5 - 3.6)
Tajikistan	Proteus spp.	98 (64 - 147)	2.5 (1.6 - 3.7)
Tajikistan	Providencia spp.	9 (6 - 14)	0.2 (0.1 - 0.4)
Tajikistan	Pseudomonas aeruginosa	463 (315 - 668)	9.2 (6.0 - 13.8)
Tajikistan	Salmonella Paratyphi	0 (0 - 0)	0.0 (0.0 - 0.0)
Tajikistan	Salmonella Typhi	31 (16 - 54)	0.4 (0.2 - 0.8)
Tajikistan	Serratia spp.	113 (64 - 191)	2.3 (1.3 - 3.9)
Tajikistan	Shigella spp.	78 (25 - 177)	0.7 (0.2 - 1.6)
Tajikistan	Staphylococcus aureus	702 (500 - 981)	13.1 (9.0 - 18.6)
Tajikistan	Streptococcus pneumoniae	1,267 (972 - 1,702)	15.5 (12.1 - 20.3)
Tajikistan	Vibrio cholerae	100 (52 - 188)	1.3 (0.7 - 2.6)
Turkmenistan	Acinetobacter baumannii	190 (103 - 322)	5.2 (2.8 - 9.0)

Turkmenistan	Aeromonas spp.	1 (0 - 2)	0.0 (0.0 - 0.0)
Turkmenistan	Campylobacter spp.	7 (2 - 15)	0.1 (0.0 - 0.3)
Turkmenistan	Chlamydia spp.	37 (28 - 50)	0.8 (0.6 - 1.1)
Turkmenistan	Citrobacter spp.	29 (17 - 46)	0.7 (0.4 - 1.1)
Turkmenistan	Clostridioides difficile	2 (1 - 5)	0.1 (0.0 - 0.1)
Turkmenistan	Enterobacter spp.	156 (97 - 240)	3.7 (2.3 - 5.7)
Turkmenistan	Enterococcus faecalis	175 (102 - 276)	4.1 (2.4 - 6.5)
Turkmenistan	Enterococcus faecium	170 (98 - 277)	4.1 (2.4 - 6.7)
Turkmenistan	Escherichia coli	589 (387 - 864)	14.5 (9.4 - 21.7)
Turkmenistan	Group A Streptococcus	89 (41 - 176)	2.3 (1.0 - 4.6)
Turkmenistan	Group B Streptococcus	136 (92 - 196)	3.1 (2.0 - 4.5)
Turkmenistan	Haemophilus influenzae	59 (46 - 76)	1.2 (0.9 - 1.6)
Turkmenistan	Klebsiella pneumoniae	480 (313 - 706)	11.7 (7.5 - 17.6)
Turkmenistan	Legionella spp.	32 (20 - 52)	0.7 (0.4 - 1.1)
Turkmenistan	Listeria monocytogenes	4 (3 - 7)	0.1 (0.1 - 0.1)
Turkmenistan	Morganella spp.	4 (3 - 7)	0.1 (0.1 - 0.2)
Turkmenistan	Mycoplasma spp.	60 (47 - 78)	1.2 (0.9 - 1.5)
Turkmenistan	Neisseria gonorrhoeae	2 (1 - 2)	0.0 (0.0 - 0.0)
Turkmenistan	Neisseria meningitidis	57 (32 - 93)	1.1 (0.6 - 1.9)
Turkmenistan	Non-typhoidal Salmonella	25 (14 - 41)	0.6 (0.3 - 0.9)
Turkmenistan	Other Klebsiella species	51 (25 - 91)	1.2 (0.6 - 2.0)
Turkmenistan	Other enterococci	53 (34 - 80)	1.4 (0.9 - 2.2)
Turkmenistan	Proteus spp.	72 (45 - 109)	1.9 (1.2 - 2.9)
Turkmenistan	Providencia spp.	4 (3 - 7)	0.1 (0.1 - 0.2)
Turkmenistan	Pseudomonas aeruginosa	354 (230 - 532)	8.7 (5.5 - 13.2)
Turkmenistan	Salmonella Paratyphi	0 (0 - 0)	0.0 (0.0 - 0.0)
Turkmenistan	Salmonella Typhi	19 (11 - 33)	0.4 (0.2 - 0.7)
Turkmenistan	Serratia spp.	59 (33 - 96)	1.4 (0.8 - 2.3)
Turkmenistan	Shigella spp.	7 (3 - 15)	0.1 (0.0 - 0.3)
Turkmenistan	Staphylococcus aureus	667 (455 - 962)	15.9 (10.6 - 23.3)
Turkmenistan	Streptococcus pneumoniae	461 (358 - 601)	9.7 (7.5 - 12.9)
Turkmenistan	Vibrio cholerae	19 (10 - 33)	0.4 (0.2 - 0.8)
Uzbekistan	Acinetobacter baumannii	968 (532 - 1,600)	6.5 (3.5 - 10.6)
Uzbekistan	Aeromonas spp.	2 (1 - 4)	0.0 (0.0 - 0.0)
Uzbekistan	Campylobacter spp.	17 (6 - 39)	0.1 (0.0 - 0.2)
Uzbekistan	Chlamydia spp.	244 (191 - 318)	1.0 (0.8 - 1.3)
Uzbekistan	Citrobacter spp.	154 (91 - 238)	0.8 (0.5 - 1.2)
Uzbekistan	Clostridioides difficile	23 (10 - 45)	0.1 (0.0 - 0.2)
Uzbekistan	Enterobacter spp.	898 (585 - 1,361)	4.5 (2.9 - 6.8)
Uzbekistan	Enterococcus faecalis	964 (570 - 1,501)	4.7 (2.7 - 7.4)
Uzbekistan	Enterococcus faecium	995 (578 - 1,597)	5.1 (3.0 - 8.1)

Uzbekistan	Escherichia coli	3,408 (2,298 - 4,914)	18.4 (12.1 - 27.0)
Uzbekistan	Group A Streptococcus	530 (239 - 1,033)	3.1 (1.2 - 6.5)
Uzbekistan	Group B Streptococcus	899 (647 - 1,250)	4.1 (2.8 - 5.9)
Uzbekistan	Haemophilus influenzae	404 (329 - 503)	1.5 (1.2 - 1.9)
Uzbekistan	Klebsiella pneumoniae	2,934 (2,042 - 4,210)	15.4 (10.3 - 22.7)
Uzbekistan	Legionella spp.	244 (164 - 372)	1.0 (0.7 - 1.4)
Uzbekistan	Listeria monocytogenes	17 (10 - 31)	0.1 (0.0 - 0.1)
Uzbekistan	Morganella spp.	23 (14 - 35)	0.1 (0.1 - 0.2)
Uzbekistan	Mycoplasma spp.	415 (338 - 514)	1.4 (1.1 - 1.7)
Uzbekistan	Neisseria gonorrhoeae	6 (4 - 7)	0.0 (0.0 - 0.0)
Uzbekistan	Neisseria meningitidis	285 (157 - 480)	1.0 (0.6 - 1.7)
Uzbekistan	Non-typhoidal Salmonella	117 (68 - 193)	0.6 (0.3 - 1.0)
Uzbekistan	Other Klebsiella species	277 (138 - 490)	1.2 (0.6 - 2.1)
Uzbekistan	Other enterococci	280 (178 - 428)	1.8 (1.1 - 2.9)
Uzbekistan	Proteus spp.	394 (244 - 600)	2.5 (1.5 - 3.8)
Uzbekistan	Providencia spp.	22 (13 - 34)	0.1 (0.1 - 0.2)
Uzbekistan	Pseudomonas aeruginosa	2,176 (1,489 - 3,147)	11.5 (7.6 - 17.0)
Uzbekistan	Salmonella Paratyphi	2 (0 - 5)	0.0 (0.0 - 0.1)
Uzbekistan	Salmonella Typhi	108 (58 - 187)	0.4 (0.2 - 0.7)
Uzbekistan	Serratia spp.	332 (193 - 548)	1.7 (1.0 - 2.8)
Uzbekistan	Shigella spp.	16 (6 - 34)	0.1 (0.0 - 0.1)
Uzbekistan	Staphylococcus aureus	4,394 (3,213 - 6,071)	22.2 (15.7 - 31.5)
Uzbekistan	Streptococcus pneumoniae	3,122 (2,536 - 3,892)	12.3 (9.7 - 15.8)
Uzbekistan	Vibrio cholerae	54 (26 - 104)	0.2 (0.1 - 0.5)
Albania	Acinetobacter baumannii	58 (28 - 106)	1.4 (0.7 - 2.6)
Albania	Aeromonas spp.	0 (0 - 0)	0.0 (0.0 - 0.0)
Albania	Campylobacter spp.	1 (0 - 3)	0.0 (0.0 - 0.1)
Albania	Chlamydia spp.	10 (7 - 14)	0.3 (0.2 - 0.4)
Albania	Citrobacter spp.	11 (5 - 22)	0.3 (0.1 - 0.6)
Albania	Clostridioides difficile	3 (1 - 6)	0.1 (0.0 - 0.2)
Albania	Enterobacter spp.	47 (27 - 76)	1.2 (0.7 - 1.9)
Albania	Enterococcus faecalis	80 (42 - 135)	2.0 (1.1 - 3.4)
Albania	Enterococcus faecium	88 (47 - 144)	2.2 (1.2 - 3.6)
Albania	Escherichia coli	471 (262 - 759)	11.7 (6.5 - 18.8)
Albania	Group A Streptococcus	74 (31 - 148)	2.1 (0.9 - 4.0)
Albania	Group B Streptococcus	56 (32 - 90)	1.5 (0.9 - 2.4)
Albania	Haemophilus influenzae	15 (11 - 19)	0.5 (0.4 - 0.6)
Albania	Klebsiella pneumoniae	214 (125 - 336)	5.5 (3.3 - 8.6)
Albania	Legionella spp.	18 (13 - 24)	0.5 (0.4 - 0.7)
Albania	Listeria monocytogenes	2 (1 - 5)	0.1 (0.0 - 0.2)
Albania	Morganella spp.	2 (1 - 3)	0.0 (0.0 - 0.1)

Albania	Mycoplasma spp.	15 (11 - 20)	0.5 (0.4 - 0.7)
Albania	Neisseria gonorrhoeae	0 (0 - 0)	0.0 (0.0 - 0.0)
Albania	Neisseria meningitidis	16 (8 - 27)	0.6 (0.3 - 1.0)
Albania	Non-typhoidal Salmonella	13 (7 - 21)	0.4 (0.2 - 0.6)
Albania	Other Klebsiella species	12 (6 - 21)	0.3 (0.1 - 0.5)
Albania	Other enterococci	31 (15 - 54)	0.8 (0.4 - 1.3)
Albania	Proteus spp.	39 (22 - 63)	0.9 (0.5 - 1.5)
Albania	Providencia spp.	1 (0 - 2)	0.0 (0.0 - 0.0)
Albania	Pseudomonas aeruginosa	152 (92 - 237)	3.9 (2.4 - 6.1)
Albania	Salmonella Paratyphi	0 (0 - 0)	0.0 (0.0 - 0.0)
Albania	Salmonella Typhi	5 (1 - 11)	0.1 (0.0 - 0.3)
Albania	Serratia spp.	13 (7 - 21)	0.3 (0.2 - 0.5)
Albania	Shigella spp.	1 (0 - 2)	0.0 (0.0 - 0.1)
Albania	Staphylococcus aureus	385 (244 - 577)	10.0 (6.5 - 15.0)
Albania	Streptococcus pneumoniae	169 (114 - 243)	5.0 (3.5 - 7.0)
Albania	Vibrio cholerae	0 (0 - 0)	0.0 (0.0 - 0.0)
Bosnia and Herzegovina	Acinetobacter baumannii	221 (111 - 381)	3.9 (1.9 - 6.8)
Bosnia and Herzegovina	Aeromonas spp.	0 (0 - 0)	0.0 (0.0 - 0.0)
Bosnia and Herzegovina	Campylobacter spp.	1 (0 - 4)	0.0 (0.0 - 0.1)
Bosnia and Herzegovina	Chlamydia spp.	22 (14 - 35)	0.5 (0.3 - 0.7)
Bosnia and Herzegovina	Citrobacter spp.	54 (21 - 115)	1.0 (0.4 - 2.0)
Bosnia and Herzegovina	Clostridioides difficile	7 (3 - 16)	0.1 (0.1 - 0.3)
Bosnia and Herzegovina	Enterobacter spp.	153 (87 - 251)	2.7 (1.5 - 4.5)
Bosnia and Herzegovina	Enterococcus faecalis	149 (82 - 245)	2.6 (1.5 - 4.3)
Bosnia and Herzegovina	Enterococcus faecium	149 (86 - 232)	2.6 (1.5 - 4.0)
Bosnia and Herzegovina	Escherichia coli	585 (338 - 903)	10.3 (5.9 - 16.1)
Bosnia and Herzegovina	Group A Streptococcus	122 (52 - 248)	2.3 (1.0 - 4.4)
Bosnia and Herzegovina	Group B Streptococcus	95 (53 - 154)	1.9 (1.0 - 3.0)
Bosnia and Herzegovina	Haemophilus influenzae	19 (11 - 31)	0.4 (0.3 - 0.7)
Bosnia and Herzegovina	Klebsiella pneumoniae	327 (191 - 504)	5.9 (3.4 - 9.2)
Bosnia and Herzegovina	Legionella spp.	10 (6 - 15)	0.2 (0.1 - 0.3)
Bosnia and Herzegovina	Listeria monocytogenes	5 (2 - 11)	0.1 (0.0 - 0.3)
Bosnia and Herzegovina	Morganella spp.	3 (1 - 6)	0.1 (0.0 - 0.1)
Bosnia and Herzegovina	Mycoplasma spp.	24 (16 - 35)	0.5 (0.4 - 0.8)
Bosnia and Herzegovina	Neisseria gonorrhoeae	0 (0 - 0)	0.0 (0.0 - 0.0)
Bosnia and Herzegovina	Neisseria meningitidis	40 (21 - 67)	0.9 (0.5 - 1.5)
Bosnia and Herzegovina	Non-typhoidal Salmonella	71 (37 - 118)	1.3 (0.7 - 2.2)
Bosnia and Herzegovina	Other Klebsiella species	26 (14 - 46)	0.5 (0.2 - 0.8)
Bosnia and Herzegovina	Other enterococci	77 (38 - 135)	1.4 (0.7 - 2.4)
Bosnia and Herzegovina	Proteus spp.	74 (43 - 114)	1.3 (0.7 - 2.0)

Bosnia and Herzegovina	Providencia spp.	2 (1 - 4)	0.0 (0.0 - 0.1)
Bosnia and Herzegovina	Pseudomonas aeruginosa	251 (150 - 391)	4.5 (2.7 - 7.0)
Bosnia and Herzegovina	Salmonella Paratyphi	0 (0 - 0)	0.0 (0.0 - 0.0)
Bosnia and Herzegovina	Salmonella Typhi	5 (1 - 12)	0.1 (0.0 - 0.2)
Bosnia and Herzegovina	Serratia spp.	36 (20 - 58)	0.6 (0.3 - 1.0)
Bosnia and Herzegovina	Shigella spp.	1 (0 - 2)	0.0 (0.0 - 0.1)
Bosnia and Herzegovina	Staphylococcus aureus	443 (261 - 681)	7.9 (4.6 - 12.2)
Bosnia and Herzegovina	Streptococcus pneumoniae	261 (163 - 394)	5.1 (3.2 - 7.7)
Bosnia and Herzegovina	Vibrio cholerae	0 (0 - 0)	0.0 (0.0 - 0.0)
Bulgaria	Acinetobacter baumannii	370 (181 - 656)	2.6 (1.3 - 4.7)
Bulgaria	Aeromonas spp.	0 (0 - 1)	0.0 (0.0 - 0.0)
Bulgaria	Campylobacter spp.	4 (1 - 11)	0.0 (0.0 - 0.1)
Bulgaria	Chlamydia spp.	54 (38 - 76)	0.4 (0.3 - 0.6)
Bulgaria	Citrobacter spp.	72 (36 - 129)	0.5 (0.3 - 1.0)
Bulgaria	Clostridioides difficile	32 (15 - 62)	0.3 (0.1 - 0.5)
Bulgaria	Enterobacter spp.	308 (186 - 482)	2.3 (1.4 - 3.6)
Bulgaria	Enterococcus faecalis	473 (261 - 777)	3.5 (1.9 - 5.8)
Bulgaria	Enterococcus faecium	503 (290 - 787)	3.7 (2.1 - 5.9)
Bulgaria	Escherichia coli	2,434 (1,378 - 3,868)	17.2 (9.7 - 27.5)
Bulgaria	Group A Streptococcus	356 (148 - 721)	2.9 (1.3 - 5.5)
Bulgaria	Group B Streptococcus	271 (157 - 436)	2.1 (1.3 - 3.4)
Bulgaria	Haemophilus influenzae	71 (49 - 99)	0.6 (0.4 - 0.8)
Bulgaria	Klebsiella pneumoniae	1,183 (710 - 1,805)	8.7 (5.2 - 13.2)
Bulgaria	Legionella spp.	76 (55 - 102)	0.6 (0.5 - 0.8)
Bulgaria	Listeria monocytogenes	6 (3 - 11)	0.1 (0.0 - 0.1)
Bulgaria	Morganella spp.	11 (5 - 21)	0.1 (0.0 - 0.1)
Bulgaria	Mycoplasma spp.	70 (51 - 95)	0.7 (0.5 - 0.9)
Bulgaria	Neisseria gonorrhoeae	1 (0 - 1)	0.0 (0.0 - 0.0)
Bulgaria	Neisseria meningitidis	71 (36 - 124)	0.8 (0.4 - 1.3)
Bulgaria	Non-typhoidal Salmonella	74 (37 - 128)	0.6 (0.3 - 1.0)
Bulgaria	Other Klebsiella species	90 (47 - 153)	0.7 (0.4 - 1.2)
Bulgaria	Other enterococci	168 (83 - 288)	1.2 (0.6 - 2.0)
Bulgaria	Proteus spp.	226 (130 - 354)	1.6 (0.9 - 2.5)
Bulgaria	Providencia spp.	7 (3 - 14)	0.0 (0.0 - 0.1)
Bulgaria	Pseudomonas aeruginosa	872 (528 - 1,351)	6.4 (3.9 - 10.0)
Bulgaria	Salmonella Paratyphi	0 (0 - 0)	0.0 (0.0 - 0.0)
Bulgaria	Salmonella Typhi	25 (8 - 59)	0.2 (0.1 - 0.6)
Bulgaria	Serratia spp.	79 (44 - 129)	0.6 (0.3 - 1.0)
Bulgaria	Shigella spp.	2 (1 - 5)	0.0 (0.0 - 0.1)
Bulgaria	Staphylococcus aureus	1,882 (1,204 - 2,789)	14.0 (9.0 - 20.9)

Bulgaria	Streptococcus pneumoniae	822 (553 - 1,183)	6.8 (4.7 - 9.7)
Bulgaria	Vibrio cholerae	0 (0 - 0)	0.0 (0.0 - 0.0)
Croatia	Acinetobacter baumannii	289 (143 - 511)	3.3 (1.6 - 5.8)
Croatia	Aeromonas spp.	0 (0 - 1)	0.0 (0.0 - 0.0)
Croatia	Campylobacter spp.	3 (1 - 8)	0.0 (0.0 - 0.1)
Croatia	Chlamydia spp.	32 (21 - 47)	0.4 (0.2 - 0.5)
Croatia	Citrobacter spp.	63 (28 - 125)	0.8 (0.3 - 1.5)
Croatia	Clostridioides difficile	15 (8 - 28)	0.2 (0.1 - 0.3)
Croatia	Enterobacter spp.	203 (118 - 333)	2.4 (1.4 - 4.0)
Croatia	Enterococcus faecalis	221 (130 - 352)	2.6 (1.5 - 4.0)
Croatia	Enterococcus faecium	214 (127 - 328)	2.5 (1.4 - 3.8)
Croatia	Escherichia coli	871 (557 - 1,272)	9.6 (6.1 - 14.2)
Croatia	Group A Streptococcus	129 (54 - 258)	1.6 (0.7 - 3.1)
Croatia	Group B Streptococcus	110 (64 - 179)	1.3 (0.8 - 2.1)
Croatia	Haemophilus influenzae	19 (12 - 28)	0.2 (0.1 - 0.3)
Croatia	Klebsiella pneumoniae	391 (245 - 578)	4.4 (2.7 - 6.6)
Croatia	Legionella spp.	28 (19 - 39)	0.3 (0.2 - 0.5)
Croatia	Listeria monocytogenes	2 (1 - 4)	0.0 (0.0 - 0.1)
Croatia	Morganella spp.	6 (4 - 8)	0.1 (0.0 - 0.1)
Croatia	Mycoplasma spp.	42 (29 - 60)	0.5 (0.4 - 0.8)
Croatia	Neisseria gonorrhoeae	0 (0 - 0)	0.0 (0.0 - 0.0)
Croatia	Neisseria meningitidis	27 (14 - 46)	0.4 (0.2 - 0.7)
Croatia	Non-typhoidal Salmonella	44 (22 - 74)	0.5 (0.3 - 0.9)
Croatia	Other Klebsiella species	39 (21 - 65)	0.5 (0.2 - 0.8)
Croatia	Other enterococci	104 (59 - 172)	1.1 (0.6 - 1.9)
Croatia	Proteus spp.	100 (65 - 143)	1.1 (0.7 - 1.6)
Croatia	Providencia spp.	2 (2 - 4)	0.0 (0.0 - 0.0)
Croatia	Pseudomonas aeruginosa	335 (206 - 508)	3.8 (2.3 - 5.8)
Croatia	Salmonella Paratyphi	0 (0 - 0)	0.0 (0.0 - 0.0)
Croatia	Salmonella Typhi	2 (1 - 5)	0.0 (0.0 - 0.1)
Croatia	Serratia spp.	36 (21 - 57)	0.4 (0.2 - 0.7)
Croatia	Shigella spp.	2 (1 - 4)	0.0 (0.0 - 0.0)
Croatia	Staphylococcus aureus	689 (413 - 1,051)	7.9 (4.7 - 12.2)
Croatia	Streptococcus pneumoniae	280 (182 - 413)	3.3 (2.1 - 4.9)
Croatia	Vibrio cholerae	0 (0 - 0)	0.0 (0.0 - 0.0)
Czechia	Acinetobacter baumannii	656 (336 - 1,143)	3.1 (1.6 - 5.5)
Czechia	Aeromonas spp.	2 (1 - 3)	0.0 (0.0 - 0.0)
Czechia	Campylobacter spp.	21 (4 - 55)	0.1 (0.0 - 0.2)
Czechia	Chlamydia spp.	132 (101 - 173)	0.6 (0.5 - 0.8)
Czechia	Citrobacter spp.	134 (58 - 270)	0.7 (0.3 - 1.4)

Czechia	Clostridioides difficile	43 (27 - 67)	0.2 (0.2 - 0.4)
Czechia	Enterobacter spp.	457 (275 - 736)	2.3 (1.4 - 3.7)
Czechia	Enterococcus faecalis	470 (273 - 748)	2.3 (1.3 - 3.7)
Czechia	Enterococcus faecium	464 (279 - 704)	2.3 (1.4 - 3.5)
Czechia	Escherichia coli	1,846 (1,171 - 2,726)	8.7 (5.5 - 12.9)
Czechia	Group A Streptococcus	325 (153 - 619)	1.7 (0.8 - 3.1)
Czechia	Group B Streptococcus	271 (169 - 428)	1.3 (0.8 - 2.1)
Czechia	Haemophilus influenzae	74 (56 - 96)	0.4 (0.3 - 0.5)
Czechia	Klebsiella pneumoniae	897 (581 - 1,306)	4.3 (2.8 - 6.3)
Czechia	Legionella spp.	127 (97 - 161)	0.6 (0.5 - 0.8)
Czechia	Listeria monocytogenes	5 (3 - 9)	0.0 (0.0 - 0.1)
Czechia	Morganella spp.	10 (6 - 15)	0.0 (0.0 - 0.1)
Czechia	Mycoplasma spp.	187 (146 - 237)	1.0 (0.8 - 1.3)
Czechia	Neisseria gonorrhoeae	3 (2 - 3)	0.0 (0.0 - 0.0)
Czechia	Neisseria meningitidis	64 (33 - 107)	0.4 (0.2 - 0.7)
Czechia	Non-typhoidal Salmonella	108 (57 - 175)	0.6 (0.3 - 0.9)
Czechia	Other Klebsiella species	86 (48 - 141)	0.4 (0.2 - 0.7)
Czechia	Other enterococci	214 (119 - 365)	1.0 (0.5 - 1.7)
Czechia	Proteus spp.	204 (130 - 295)	0.9 (0.6 - 1.4)
Czechia	Providencia spp.	4 (3 - 7)	0.0 (0.0 - 0.0)
Czechia	Pseudomonas aeruginosa	812 (533 - 1,195)	3.9 (2.6 - 5.8)
Czechia	Salmonella Paratyphi	0 (0 - 1)	0.0 (0.0 - 0.0)
Czechia	Salmonella Typhi	11 (3 - 20)	0.1 (0.0 - 0.1)
Czechia	Serratia spp.	81 (46 - 127)	0.4 (0.2 - 0.6)
Czechia	Shigella spp.	11 (4 - 25)	0.1 (0.0 - 0.1)
Czechia	Staphylococcus aureus	1,721 (1,118 - 2,517)	8.4 (5.4 - 12.3)
Czechia	Streptococcus pneumoniae	946 (699 - 1,264)	4.7 (3.5 - 6.3)
Czechia	Vibrio cholerae	0 (0 - 0)	0.0 (0.0 - 0.0)
Hungary	Acinetobacter baumannii	298 (149 - 534)	1.6 (0.8 - 2.8)
Hungary	Aeromonas spp.	2 (1 - 3)	0.0 (0.0 - 0.0)
Hungary	Campylobacter spp.	19 (4 - 49)	0.1 (0.0 - 0.2)
Hungary	Chlamydia spp.	39 (26 - 55)	0.2 (0.1 - 0.3)
Hungary	Citrobacter spp.	75 (43 - 126)	0.4 (0.2 - 0.7)
Hungary	Clostridioides difficile	34 (22 - 52)	0.2 (0.2 - 0.4)
Hungary	Enterobacter spp.	322 (202 - 488)	1.7 (1.1 - 2.6)
Hungary	Enterococcus faecalis	571 (325 - 908)	3.1 (1.8 - 5.0)
Hungary	Enterococcus faecium	631 (378 - 956)	3.4 (2.0 - 5.2)
Hungary	Escherichia coli	2,840 (1,688 - 4,338)	14.7 (8.7 - 22.5)
Hungary	Group A Streptococcus	370 (179 - 703)	2.1 (1.1 - 3.9)
Hungary	Group B Streptococcus	279 (164 - 457)	1.5 (0.9 - 2.5)
Hungary	Haemophilus influenzae	50 (33 - 72)	0.3 (0.2 - 0.4)

Hungary	Klebsiella pneumoniae	1,191 (735 - 1,797)	6.3 (3.9 - 9.6)
Hungary	Legionella spp.	78 (55 - 109)	0.4 (0.3 - 0.6)
Hungary	Listeria monocytogenes	7 (4 - 12)	0.0 (0.0 - 0.1)
Hungary	Morganella spp.	9 (5 - 15)	0.0 (0.0 - 0.1)
Hungary	Mycoplasma spp.	54 (38 - 76)	0.3 (0.2 - 0.5)
Hungary	Neisseria gonorrhoeae	7 (6 - 9)	0.0 (0.0 - 0.1)
Hungary	Neisseria meningitidis	54 (28 - 93)	0.4 (0.2 - 0.7)
Hungary	Non-typhoidal Salmonella	55 (30 - 90)	0.3 (0.2 - 0.5)
Hungary	Other Klebsiella species	114 (63 - 184)	0.6 (0.4 - 1.0)
Hungary	Other enterococci	171 (95 - 280)	0.9 (0.5 - 1.5)
Hungary	Proteus spp.	241 (148 - 360)	1.2 (0.7 - 1.8)
Hungary	Providencia spp.	5 (3 - 8)	0.0 (0.0 - 0.0)
Hungary	Pseudomonas aeruginosa	888 (555 - 1,347)	4.7 (2.9 - 7.2)
Hungary	Salmonella Paratyphi	0 (0 - 0)	0.0 (0.0 - 0.0)
Hungary	Salmonella Typhi	16 (5 - 38)	0.1 (0.0 - 0.2)
Hungary	Serratia spp.	80 (47 - 125)	0.4 (0.3 - 0.7)
Hungary	Shigella spp.	10 (4 - 23)	0.1 (0.0 - 0.1)
Hungary	Staphylococcus aureus	2,097 (1,322 - 3,136)	11.2 (7.0 - 16.9)
Hungary	Streptococcus pneumoniae	681 (441 - 1,018)	3.8 (2.5 - 5.7)
Hungary	Vibrio cholerae	0 (0 - 0)	0.0 (0.0 - 0.0)
Montenegro	Acinetobacter baumannii	40 (20 - 69)	4.3 (2.2 - 7.6)
Montenegro	Aeromonas spp.	0 (0 - 0)	0.0 (0.0 - 0.0)
Montenegro	Campylobacter spp.	0 (0 - 0)	0.0 (0.0 - 0.1)
Montenegro	Chlamydia spp.	4 (3 - 6)	0.5 (0.3 - 0.7)
Montenegro	Citrobacter spp.	9 (3 - 19)	0.9 (0.4 - 2.0)
Montenegro	Clostridioides difficile	2 (1 - 5)	0.2 (0.1 - 0.5)
Montenegro	Enterobacter spp.	24 (14 - 40)	2.6 (1.5 - 4.4)
Montenegro	Enterococcus faecalis	22 (12 - 36)	2.4 (1.3 - 3.9)
Montenegro	Enterococcus faecium	23 (14 - 36)	2.5 (1.4 - 3.8)
Montenegro	Escherichia coli	86 (51 - 134)	9.3 (5.4 - 14.6)
Montenegro	Group A Streptococcus	18 (8 - 38)	2.1 (0.9 - 4.1)
Montenegro	Group B Streptococcus	14 (8 - 23)	1.5 (0.9 - 2.5)
Montenegro	Haemophilus influenzae	2 (2 - 4)	0.3 (0.2 - 0.4)
Montenegro	Klebsiella pneumoniae	46 (28 - 70)	5.0 (3.0 - 7.7)
Montenegro	Legionella spp.	3 (2 - 4)	0.3 (0.2 - 0.4)
Montenegro	Listeria monocytogenes	0 (0 - 1)	0.0 (0.0 - 0.1)
Montenegro	Morganella spp.	0 (0 - 1)	0.0 (0.0 - 0.1)
Montenegro	Mycoplasma spp.	5 (4 - 7)	0.6 (0.5 - 0.9)
Montenegro	Neisseria gonorrhoeae	0 (0 - 0)	0.0 (0.0 - 0.0)
Montenegro	Neisseria meningitidis	6 (3 - 9)	0.7 (0.4 - 1.2)

Montenegro	Non-typhoidal Salmonella	9 (5 - 15)	1.0 (0.5 - 1.7)
Montenegro	Other Klebsiella species	4 (2 - 6)	0.4 (0.2 - 0.7)
Montenegro	Other enterococci	12 (6 - 21)	1.3 (0.6 - 2.3)
Montenegro	Proteus spp.	10 (6 - 16)	1.1 (0.6 - 1.7)
Montenegro	Providencia spp.	0 (0 - 0)	0.0 (0.0 - 0.1)
Montenegro	Pseudomonas aeruginosa	38 (23 - 58)	4.1 (2.5 - 6.3)
Montenegro	Salmonella Paratyphi	0 (0 - 0)	0.0 (0.0 - 0.0)
Montenegro	Salmonella Typhi	1 (0 - 1)	0.1 (0.0 - 0.2)
Montenegro	Serratia spp.	5 (3 - 8)	0.6 (0.3 - 0.9)
Montenegro	Shigella spp.	0 (0 - 0)	0.0 (0.0 - 0.0)
Montenegro	Staphylococcus aureus	77 (47 - 118)	8.4 (5.1 - 12.8)
Montenegro	Streptococcus pneumoniae	39 (26 - 57)	4.5 (3.0 - 6.6)
Montenegro	Vibrio cholerae	0 (0 - 0)	0.0 (0.0 - 0.0)
North Macedonia	Acinetobacter baumannii	155 (79 - 266)	5.6 (2.8 - 9.7)
North Macedonia	Aeromonas spp.	0 (0 - 0)	0.0 (0.0 - 0.0)
North Macedonia	Campylobacter spp.	1 (0 - 3)	0.0 (0.0 - 0.1)
North Macedonia	Chlamydia spp.	16 (9 - 25)	0.7 (0.4 - 1.1)
North Macedonia	Citrobacter spp.	33 (13 - 72)	1.2 (0.5 - 2.6)
North Macedonia	Clostridioides difficile	5 (2 - 12)	0.2 (0.1 - 0.4)
North Macedonia	Enterobacter spp.	89 (49 - 150)	3.2 (1.8 - 5.4)
North Macedonia	Enterococcus faecalis	83 (45 - 138)	3.0 (1.6 - 4.9)
North Macedonia	Enterococcus faecium	85 (48 - 135)	2.9 (1.7 - 4.7)
North Macedonia	Escherichia coli	326 (181 - 516)	11.8 (6.6 - 18.7)
North Macedonia	Group A Streptococcus	77 (31 - 160)	2.9 (1.2 - 5.9)
North Macedonia	Group B Streptococcus	52 (28 - 87)	2.0 (1.1 - 3.3)
North Macedonia	Haemophilus influenzae	10 (6 - 16)	0.5 (0.3 - 0.7)
North Macedonia	Klebsiella pneumoniae	184 (107 - 287)	6.7 (3.9 - 10.5)
North Macedonia	Legionella spp.	9 (5 - 14)	0.4 (0.2 - 0.6)
North Macedonia	Listeria monocytogenes	1 (0 - 2)	0.0 (0.0 - 0.1)
North Macedonia	Morganella spp.	2 (1 - 4)	0.1 (0.0 - 0.1)
North Macedonia	Mycoplasma spp.	18 (11 - 26)	0.8 (0.6 - 1.2)
North Macedonia	Neisseria gonorrhoeae	0 (0 - 0)	0.0 (0.0 - 0.0)
North Macedonia	Neisseria meningitidis	25 (13 - 42)	1.0 (0.5 - 1.6)
North Macedonia	Non-typhoidal Salmonella	43 (22 - 73)	1.6 (0.8 - 2.7)
North Macedonia	Other Klebsiella species	13 (7 - 24)	0.5 (0.2 - 0.8)
North Macedonia	Other enterococci	46 (22 - 83)	1.7 (0.8 - 3.1)
North Macedonia	Proteus spp.	41 (23 - 65)	1.5 (0.8 - 2.4)
North Macedonia	Providencia spp.	1 (0 - 3)	0.0 (0.0 - 0.1)
North Macedonia	Pseudomonas aeruginosa	149 (86 - 239)	5.5 (3.2 - 8.8)
North Macedonia	Salmonella Paratyphi	0 (0 - 0)	0.0 (0.0 - 0.0)

North Macedonia	Salmonella Typhi	3 (1 - 7)	0.1 (0.0 - 0.3)
North Macedonia	Serratia spp.	21 (12 - 34)	0.7 (0.4 - 1.2)
North Macedonia	Shigella spp.	1 (0 - 1)	0.0 (0.0 - 0.1)
North Macedonia	Staphylococcus aureus	266 (154 - 414)	9.7 (5.6 - 15.0)
North Macedonia	Streptococcus pneumoniae	149 (92 - 229)	6.0 (3.8 - 9.0)
North Macedonia	Vibrio cholerae	0 (0 - 0)	0.0 (0.0 - 0.0)
Poland	Acinetobacter baumannii	1,326 (696 - 2,263)	1.9 (1.0 - 3.3)
Poland	Aeromonas spp.	2 (1 - 4)	0.0 (0.0 - 0.0)
Poland	Campylobacter spp.	25 (5 - 65)	0.0 (0.0 - 0.1)
Poland	Chlamydia spp.	304 (240 - 392)	0.4 (0.4 - 0.6)
Poland	Citrobacter spp.	278 (147 - 505)	0.4 (0.2 - 0.8)
Poland	Clostridioides difficile	119 (69 - 199)	0.2 (0.1 - 0.3)
Poland	Enterobacter spp.	1,155 (745 - 1,728)	1.7 (1.1 - 2.6)
Poland	Enterococcus faecalis	1,673 (946 - 2,711)	2.5 (1.4 - 4.0)
Poland	Enterococcus faecium	1,771 (1,053 - 2,781)	2.6 (1.5 - 4.1)
Poland	Escherichia coli	7,923 (4,900 - 12,270)	11.4 (7.0 - 17.7)
Poland	Group A Streptococcus	1,085 (512 - 2,032)	1.7 (0.9 - 3.1)
Poland	Group B Streptococcus	986 (635 - 1,509)	1.5 (1.0 - 2.3)
Poland	Haemophilus influenzae	316 (245 - 407)	0.5 (0.4 - 0.6)
Poland	Klebsiella pneumoniae	3,893 (2,518 - 5,832)	5.7 (3.6 - 8.6)
Poland	Legionella spp.	483 (387 - 601)	0.7 (0.6 - 0.9)
Poland	Listeria monocytogenes	19 (10 - 37)	0.0 (0.0 - 0.1)
Poland	Morganella spp.	34 (20 - 55)	0.0 (0.0 - 0.1)
Poland	Mycoplasma spp.	432 (346 - 538)	0.7 (0.6 - 0.9)
Poland	Neisseria gonorrhoeae	3 (2 - 4)	0.0 (0.0 - 0.0)
Poland	Neisseria meningitidis	234 (122 - 416)	0.5 (0.2 - 0.8)
Poland	Non-typhoidal Salmonella	235 (126 - 405)	0.4 (0.2 - 0.6)
Poland	Other Klebsiella species	322 (180 - 554)	0.5 (0.3 - 0.8)
Poland	Other enterococci	584 (321 - 965)	0.8 (0.4 - 1.4)
Poland	Proteus spp.	731 (455 - 1,117)	1.0 (0.6 - 1.6)
Poland	Providencia spp.	18 (10 - 31)	0.0 (0.0 - 0.0)
Poland	Pseudomonas aeruginosa	3,197 (2,109 - 4,711)	4.6 (3.0 - 6.9)
Poland	Salmonella Paratyphi	0 (0 - 0)	0.0 (0.0 - 0.0)
Poland	Salmonella Typhi	49 (17 - 117)	0.1 (0.0 - 0.2)
Poland	Serratia spp.	263 (153 - 426)	0.4 (0.2 - 0.6)
Poland	Shigella spp.	14 (5 - 31)	0.0 (0.0 - 0.0)
Poland	Staphylococcus aureus	7,503 (5,191 - 10,826)	10.9 (7.4 - 15.8)
Poland	Streptococcus pneumoniae	3,458 (2,601 - 4,688)	5.2 (3.9 - 7.1)
Poland	Vibrio cholerae	0 (0 - 0)	0.0 (0.0 - 0.0)
Romania	Acinetobacter baumannii	704 (368 - 1,206)	1.9 (1.0 - 3.3)

Romania	Aeromonas spp.	1 (0 - 2)	0.0 (0.0 - 0.0)
Romania	Campylobacter spp.	10 (2 - 27)	0.0 (0.0 - 0.1)
Romania	Chlamydia spp.	150 (116 - 195)	0.5 (0.4 - 0.6)
Romania	Citrobacter spp.	179 (98 - 306)	0.5 (0.3 - 0.9)
Romania	Clostridioides difficile	37 (18 - 76)	0.1 (0.1 - 0.2)
Romania	Enterobacter spp.	775 (489 - 1,162)	2.2 (1.4 - 3.3)
Romania	Enterococcus faecalis	1,167 (673 - 1,944)	3.3 (1.9 - 5.5)
Romania	Enterococcus faecium	1,253 (754 - 1,952)	3.5 (2.1 - 5.6)
Romania	Escherichia coli	5,417 (3,306 - 8,507)	14.9 (9.0 - 23.4)
Romania	Group A Streptococcus	711 (306 - 1,405)	2.1 (1.0 - 4.1)
Romania	Group B Streptococcus	622 (391 - 964)	1.9 (1.2 - 2.9)
Romania	Haemophilus influenzae	191 (148 - 249)	0.7 (0.6 - 0.9)
Romania	Klebsiella pneumoniae	2,676 (1,713 - 4,031)	7.6 (4.9 - 11.4)
Romania	Legionella spp.	222 (174 - 280)	0.7 (0.6 - 0.9)
Romania	Listeria monocytogenes	12 (5 - 26)	0.0 (0.0 - 0.1)
Romania	Morganella spp.	20 (10 - 35)	0.1 (0.0 - 0.1)
Romania	Mycoplasma spp.	235 (189 - 290)	1.0 (0.8 - 1.1)
Romania	Neisseria gonorrhoeae	1 (1 - 1)	0.0 (0.0 - 0.0)
Romania	Neisseria meningitidis	147 (76 - 260)	0.6 (0.3 - 1.0)
Romania	Non-typhoidal Salmonella	151 (83 - 261)	0.5 (0.2 - 0.8)
Romania	Other Klebsiella species	259 (143 - 421)	0.8 (0.4 - 1.3)
Romania	Other enterococci	348 (182 - 595)	0.9 (0.5 - 1.6)
Romania	Proteus spp.	498 (300 - 768)	1.3 (0.8 - 2.0)
Romania	Providencia spp.	12 (6 - 22)	0.0 (0.0 - 0.1)
Romania	Pseudomonas aeruginosa	2,042 (1,320 - 3,087)	5.9 (3.8 - 8.8)
Romania	Salmonella Paratyphi	0 (0 - 0)	0.0 (0.0 - 0.0)
Romania	Salmonella Typhi	42 (14 - 97)	0.1 (0.0 - 0.3)
Romania	Serratia spp.	185 (109 - 301)	0.5 (0.3 - 0.9)
Romania	Shigella spp.	6 (2 - 14)	0.0 (0.0 - 0.1)
Romania	Staphylococcus aureus	4,539 (3,084 - 6,732)	13.2 (9.0 - 19.3)
Romania	Streptococcus pneumoniae	2,111 (1,560 - 2,915)	7.1 (5.5 - 9.5)
Romania	Vibrio cholerae	0 (0 - 0)	0.0 (0.0 - 0.0)
Serbia	Acinetobacter baumannii	447 (229 - 768)	3.0 (1.5 - 5.2)
Serbia	Aeromonas spp.	0 (0 - 1)	0.0 (0.0 - 0.0)
Serbia	Campylobacter spp.	5 (1 - 15)	0.0 (0.0 - 0.1)
Serbia	Chlamydia spp.	59 (41 - 84)	0.4 (0.3 - 0.6)
Serbia	Citrobacter spp.	94 (43 - 186)	0.6 (0.3 - 1.3)
Serbia	Clostridioides difficile	21 (8 - 49)	0.2 (0.1 - 0.3)
Serbia	Enterobacter spp.	331 (201 - 522)	2.2 (1.3 - 3.5)
Serbia	Enterococcus faecalis	469 (261 - 760)	3.1 (1.7 - 5.1)
Serbia	Enterococcus faecium	503 (295 - 786)	3.3 (1.9 - 5.2)

Serbia	Escherichia coli	2,196 (1,276 - 3,378)	14.4 (8.3 - 22.5)
Serbia	Group A Streptococcus	363 (162 - 717)	2.6 (1.2 - 4.9)
Serbia	Group B Streptococcus	276 (160 - 447)	1.9 (1.1 - 3.1)
Serbia	Haemophilus influenzae	58 (39 - 82)	0.4 (0.3 - 0.6)
Serbia	Klebsiella pneumoniae	1,072 (652 - 1,629)	7.1 (4.3 - 10.9)
Serbia	Legionella spp.	65 (46 - 88)	0.5 (0.3 - 0.6)
Serbia	Listeria monocytogenes	5 (2 - 10)	0.0 (0.0 - 0.1)
Serbia	Morganella spp.	9 (4 - 17)	0.1 (0.0 - 0.1)
Serbia	Mycoplasma spp.	74 (53 - 102)	0.6 (0.4 - 0.8)
Serbia	Neisseria gonorrhoeae	1 (1 - 1)	0.0 (0.0 - 0.0)
Serbia	Neisseria meningitidis	75 (38 - 129)	0.6 (0.3 - 1.1)
Serbia	Non-typhoidal Salmonella	103 (54 - 172)	0.7 (0.4 - 1.2)
Serbia	Other Klebsiella species	83 (45 - 143)	0.5 (0.3 - 0.9)
Serbia	Other enterococci	192 (96 - 333)	1.3 (0.6 - 2.2)
Serbia	Proteus spp.	222 (130 - 342)	1.4 (0.8 - 2.3)
Serbia	Providencia spp.	5 (2 - 10)	0.0 (0.0 - 0.1)
Serbia	Pseudomonas aeruginosa	817 (502 - 1,244)	5.5 (3.3 - 8.4)
Serbia	Salmonella Paratyphi	0 (0 - 0)	0.0 (0.0 - 0.0)
Serbia	Salmonella Typhi	16 (5 - 37)	0.1 (0.0 - 0.3)
Serbia	Serratia spp.	86 (50 - 137)	0.6 (0.3 - 0.9)
Serbia	Shigella spp.	3 (1 - 6)	0.0 (0.0 - 0.0)
Serbia	Staphylococcus aureus	1,719 (1,070 - 2,572)	11.6 (7.1 - 17.4)
Serbia	Streptococcus pneumoniae	790 (526 - 1,144)	5.6 (3.7 - 8.1)
Serbia	Vibrio cholerae	0 (0 - 0)	0.0 (0.0 - 0.0)
Slovakia	Acinetobacter baumannii	133 (67 - 233)	1.5 (0.8 - 2.7)
Slovakia	Aeromonas spp.	0 (0 - 0)	0.0 (0.0 - 0.0)
Slovakia	Campylobacter spp.	2 (0 - 5)	0.0 (0.0 - 0.1)
Slovakia	Chlamydia spp.	35 (26 - 44)	0.4 (0.3 - 0.5)
Slovakia	Citrobacter spp.	34 (19 - 58)	0.4 (0.2 - 0.7)
Slovakia	Clostridioides difficile	19 (10 - 35)	0.2 (0.1 - 0.4)
Slovakia	Enterobacter spp.	161 (103 - 240)	1.8 (1.2 - 2.8)
Slovakia	Enterococcus faecalis	249 (140 - 399)	2.9 (1.6 - 4.6)
Slovakia	Enterococcus faecium	269 (154 - 419)	3.1 (1.7 - 4.8)
Slovakia	Escherichia coli	1,194 (715 - 1,831)	13.5 (8.1 - 20.7)
Slovakia	Group A Streptococcus	145 (69 - 273)	1.8 (0.9 - 3.3)
Slovakia	Group B Streptococcus	136 (86 - 207)	1.6 (1.0 - 2.5)
Slovakia	Haemophilus influenzae	45 (34 - 59)	0.6 (0.4 - 0.7)
Slovakia	Klebsiella pneumoniae	561 (358 - 828)	6.4 (4.1 - 9.5)
Slovakia	Legionella spp.	75 (58 - 96)	0.9 (0.7 - 1.1)
Slovakia	Listeria monocytogenes	3 (1 - 5)	0.0 (0.0 - 0.1)
Slovakia	Morganella spp.	4 (3 - 7)	0.0 (0.0 - 0.1)

Slovakia	Mycoplasma spp.	54 (42 - 70)	0.7 (0.5 - 0.9)
Slovakia	Neisseria gonorrhoeae	1 (1 - 1)	0.0 (0.0 - 0.0)
Slovakia	Neisseria meningitidis	27 (14 - 46)	0.4 (0.2 - 0.7)
Slovakia	Non-typhoidal Salmonella	22 (12 - 36)	0.3 (0.1 - 0.5)
Slovakia	Other Klebsiella species	52 (29 - 86)	0.6 (0.3 - 1.0)
Slovakia	Other enterococci	71 (39 - 116)	0.8 (0.4 - 1.3)
Slovakia	Proteus spp.	100 (61 - 150)	1.1 (0.7 - 1.7)
Slovakia	Providencia spp.	2 (1 - 4)	0.0 (0.0 - 0.0)
Slovakia	Pseudomonas aeruginosa	461 (305 - 670)	5.3 (3.5 - 7.7)
Slovakia	Salmonella Paratyphi	0 (0 - 0)	0.0 (0.0 - 0.0)
Slovakia	Salmonella Typhi	7 (2 - 16)	0.1 (0.0 - 0.2)
Slovakia	Serratia spp.	34 (20 - 54)	0.4 (0.2 - 0.6)
Slovakia	Shigella spp.	1 (0 - 3)	0.0 (0.0 - 0.0)
Slovakia	Staphylococcus aureus	1,179 (807 - 1,670)	13.6 (9.3 - 19.3)
Slovakia	Streptococcus pneumoniae	463 (337 - 628)	5.6 (4.1 - 7.7)
Slovakia	Vibrio cholerae	0 (0 - 0)	0.0 (0.0 - 0.0)
Slovenia	Acinetobacter baumannii	59 (31 - 103)	1.3 (0.7 - 2.3)
Slovenia	Aeromonas spp.	0 (0 - 0)	0.0 (0.0 - 0.0)
Slovenia	Campylobacter spp.	1 (0 - 2)	0.0 (0.0 - 0.0)
Slovenia	Chlamydia spp.	15 (11 - 19)	0.3 (0.2 - 0.4)
Slovenia	Citrobacter spp.	14 (8 - 24)	0.3 (0.2 - 0.6)
Slovenia	Clostridioides difficile	7 (4 - 14)	0.2 (0.1 - 0.3)
Slovenia	Enterobacter spp.	65 (42 - 96)	1.4 (0.9 - 2.2)
Slovenia	Enterococcus faecalis	93 (51 - 159)	2.2 (1.2 - 3.6)
Slovenia	Enterococcus faecium	106 (62 - 168)	2.5 (1.4 - 3.9)
Slovenia	Escherichia coli	443 (262 - 705)	9.7 (5.7 - 15.4)
Slovenia	Group A Streptococcus	54 (26 - 106)	1.3 (0.7 - 2.5)
Slovenia	Group B Streptococcus	54 (34 - 86)	1.2 (0.8 - 1.9)
Slovenia	Haemophilus influenzae	16 (12 - 21)	0.3 (0.2 - 0.4)
Slovenia	Klebsiella pneumoniae	197 (125 - 303)	4.3 (2.7 - 6.7)
Slovenia	Legionella spp.	32 (24 - 41)	0.6 (0.5 - 0.8)
Slovenia	Listeria monocytogenes	1 (0 - 2)	0.0 (0.0 - 0.1)
Slovenia	Morganella spp.	1 (1 - 2)	0.0 (0.0 - 0.0)
Slovenia	Mycoplasma spp.	24 (18 - 31)	0.5 (0.4 - 0.7)
Slovenia	Neisseria gonorrhoeae	0 (0 - 0)	0.0 (0.0 - 0.0)
Slovenia	Neisseria meningitidis	7 (4 - 12)	0.2 (0.1 - 0.4)
Slovenia	Non-typhoidal Salmonella	7 (4 - 12)	0.2 (0.1 - 0.3)
Slovenia	Other Klebsiella species	19 (11 - 33)	0.5 (0.2 - 0.8)
Slovenia	Other enterococci	28 (15 - 48)	0.6 (0.3 - 1.1)
Slovenia	Proteus spp.	36 (22 - 57)	0.8 (0.5 - 1.2)

Slovenia	Providencia spp.	1 (0 - 1)	0.0 (0.0 - 0.0)
Slovenia	Pseudomonas aeruginosa	179 (120 - 264)	3.9 (2.5 - 5.8)
Slovenia	Salmonella Paratyphi	0 (0 - 0)	0.0 (0.0 - 0.0)
Slovenia	Salmonella Typhi	1 (0 - 3)	0.0 (0.0 - 0.1)
Slovenia	Serratia spp.	12 (7 - 19)	0.3 (0.2 - 0.5)
Slovenia	Shigella spp.	0 (0 - 1)	0.0 (0.0 - 0.0)
Slovenia	Staphylococcus aureus	464 (309 - 686)	10.0 (6.5 - 15.1)
Slovenia	Streptococcus pneumoniae	173 (127 - 238)	3.7 (2.6 - 5.1)
Slovenia	Vibrio cholerae	0 (0 - 0)	0.0 (0.0 - 0.0)
Belarus	Acinetobacter baumannii	427 (210 - 769)	2.8 (1.4 - 5.1)
Belarus	Aeromonas spp.	0 (0 - 0)	0.0 (0.0 - 0.0)
Belarus	Campylobacter spp.	3 (0 - 8)	0.0 (0.0 - 0.1)
Belarus	Chlamydia spp.	42 (27 - 66)	0.3 (0.2 - 0.5)
Belarus	Citrobacter spp.	118 (50 - 251)	0.8 (0.3 - 1.7)
Belarus	Clostridioides difficile	28 (12 - 62)	0.2 (0.1 - 0.4)
Belarus	Enterobacter spp.	395 (234 - 645)	2.6 (1.5 - 4.3)
Belarus	Enterococcus faecalis	416 (238 - 668)	2.8 (1.6 - 4.5)
Belarus	Enterococcus faecium	407 (240 - 653)	2.6 (1.5 - 4.3)
Belarus	Escherichia coli	1,550 (946 - 2,445)	10.1 (6.1 - 16.0)
Belarus	Group A Streptococcus	291 (133 - 570)	2.1 (1.0 - 4.0)
Belarus	Group B Streptococcus	286 (171 - 454)	2.0 (1.2 - 3.1)
Belarus	Haemophilus influenzae	49 (33 - 72)	0.4 (0.2 - 0.5)
Belarus	Klebsiella pneumoniae	781 (475 - 1,239)	5.1 (3.1 - 8.1)
Belarus	Legionella spp.	19 (11 - 30)	0.1 (0.1 - 0.2)
Belarus	Listeria monocytogenes	5 (3 - 10)	0.0 (0.0 - 0.1)
Belarus	Morganella spp.	9 (5 - 14)	0.1 (0.0 - 0.1)
Belarus	Mycoplasma spp.	73 (52 - 102)	0.6 (0.4 - 0.8)
Belarus	Neisseria gonorrhoeae	4 (3 - 5)	0.0 (0.0 - 0.0)
Belarus	Neisseria meningitidis	96 (48 - 171)	0.8 (0.4 - 1.4)
Belarus	Non-typhoidal Salmonella	125 (64 - 217)	0.9 (0.5 - 1.5)
Belarus	Other Klebsiella species	78 (42 - 137)	0.5 (0.3 - 0.9)
Belarus	Other enterococci	176 (93 - 310)	1.1 (0.6 - 2.0)
Belarus	Proteus spp.	176 (108 - 274)	1.1 (0.7 - 1.8)
Belarus	Providencia spp.	5 (3 - 8)	0.0 (0.0 - 0.1)
Belarus	Pseudomonas aeruginosa	618 (373 - 966)	4.0 (2.4 - 6.4)
Belarus	Salmonella Paratyphi	0 (0 - 0)	0.0 (0.0 - 0.0)
Belarus	Salmonella Typhi	10 (3 - 26)	0.1 (0.0 - 0.2)
Belarus	Serratia spp.	81 (46 - 136)	0.5 (0.3 - 0.9)
Belarus	Shigella spp.	1 (0 - 4)	0.0 (0.0 - 0.0)
Belarus	Staphylococcus aureus	1,196 (708 - 1,911)	7.9 (4.7 - 12.7)

Belarus	Streptococcus pneumoniae	688 (456 - 1,034)	4.9 (3.3 - 7.3)
Belarus	Vibrio cholerae	0 (0 - 0)	0.0 (0.0 - 0.0)
Estonia	Acinetobacter baumannii	83 (44 - 147)	3.1 (1.6 - 5.6)
Estonia	Aeromonas spp.	0 (0 - 0)	0.0 (0.0 - 0.0)
Estonia	Campylobacter spp.	0 (0 - 1)	0.0 (0.0 - 0.0)
Estonia	Chlamydia spp.	11 (8 - 16)	0.5 (0.3 - 0.6)
Estonia	Citrobacter spp.	17 (8 - 36)	0.7 (0.3 - 1.5)
Estonia	Clostridioides difficile	5 (2 - 10)	0.2 (0.1 - 0.4)
Estonia	Enterobacter spp.	55 (32 - 90)	2.2 (1.3 - 3.6)
Estonia	Enterococcus faecalis	58 (34 - 98)	2.3 (1.4 - 3.9)
Estonia	Enterococcus faecium	58 (34 - 90)	2.3 (1.3 - 3.7)
Estonia	Escherichia coli	220 (137 - 343)	8.2 (5.1 - 13.0)
Estonia	Group A Streptococcus	40 (19 - 74)	1.7 (0.9 - 3.1)
Estonia	Group B Streptococcus	30 (18 - 48)	1.2 (0.7 - 1.9)
Estonia	Haemophilus influenzae	6 (5 - 9)	0.3 (0.2 - 0.4)
Estonia	Klebsiella pneumoniae	108 (67 - 165)	4.1 (2.6 - 6.4)
Estonia	Legionella spp.	10 (7 - 14)	0.4 (0.3 - 0.6)
Estonia	Listeria monocytogenes	1 (0 - 1)	0.0 (0.0 - 0.1)
Estonia	Morganella spp.	1 (1 - 2)	0.0 (0.0 - 0.1)
Estonia	Mycoplasma spp.	17 (12 - 22)	0.8 (0.6 - 1.1)
Estonia	Neisseria gonorrhoeae	0 (0 - 0)	0.0 (0.0 - 0.0)
Estonia	Neisseria meningitidis	10 (5 - 18)	0.6 (0.3 - 1.0)
Estonia	Non-typhoidal Salmonella	15 (8 - 26)	0.6 (0.3 - 1.1)
Estonia	Other Klebsiella species	11 (6 - 19)	0.5 (0.3 - 0.8)
Estonia	Other enterococci	26 (14 - 44)	0.9 (0.5 - 1.7)
Estonia	Proteus spp.	25 (16 - 39)	0.9 (0.6 - 1.4)
Estonia	Providencia spp.	1 (0 - 1)	0.0 (0.0 - 0.0)
Estonia	Pseudomonas aeruginosa	93 (58 - 142)	3.6 (2.2 - 5.5)
Estonia	Salmonella Paratyphi	0 (0 - 0)	0.0 (0.0 - 0.0)
Estonia	Salmonella Typhi	1 (0 - 2)	0.0 (0.0 - 0.1)
Estonia	Serratia spp.	11 (6 - 17)	0.4 (0.2 - 0.7)
Estonia	Shigella spp.	0 (0 - 1)	0.0 (0.0 - 0.0)
Estonia	Staphylococcus aureus	188 (116 - 295)	7.4 (4.5 - 11.7)
Estonia	Streptococcus pneumoniae	92 (64 - 133)	3.9 (2.8 - 5.6)
Estonia	Vibrio cholerae	0 (0 - 0)	0.0 (0.0 - 0.0)
Latvia	Acinetobacter baumannii	73 (37 - 128)	1.8 (0.9 - 3.2)
Latvia	Aeromonas spp.	0 (0 - 0)	0.0 (0.0 - 0.0)
Latvia	Campylobacter spp.	1 (0 - 2)	0.0 (0.0 - 0.1)
Latvia	Chlamydia spp.	10 (7 - 14)	0.3 (0.2 - 0.4)
Latvia	Citrobacter spp.	14 (8 - 24)	0.4 (0.2 - 0.7)

Latvia	Clostridioides difficile	7 (3 - 15)	0.2 (0.1 - 0.4)
Latvia	Enterobacter spp.	62 (40 - 92)	1.6 (1.0 - 2.4)
Latvia	Enterococcus faecalis	102 (60 - 167)	2.7 (1.6 - 4.4)
Latvia	Enterococcus faecium	105 (64 - 166)	2.8 (1.6 - 4.4)
Latvia	Escherichia coli	533 (333 - 831)	13.2 (8.1 - 20.4)
Latvia	Group A Streptococcus	83 (44 - 154)	2.4 (1.3 - 4.2)
Latvia	Group B Streptococcus	57 (35 - 90)	1.6 (1.0 - 2.4)
Latvia	Haemophilus influenzae	12 (9 - 18)	0.4 (0.3 - 0.5)
Latvia	Klebsiella pneumoniae	237 (152 - 354)	6.1 (3.9 - 9.1)
Latvia	Legionella spp.	17 (13 - 23)	0.5 (0.4 - 0.7)
Latvia	Listeria monocytogenes	1 (1 - 2)	0.0 (0.0 - 0.1)
Latvia	Morganella spp.	3 (2 - 4)	0.1 (0.0 - 0.1)
Latvia	Mycoplasma spp.	14 (11 - 19)	0.5 (0.4 - 0.6)
Latvia	Neisseria gonorrhoeae	1 (1 - 1)	0.0 (0.0 - 0.0)
Latvia	Neisseria meningitidis	13 (7 - 23)	0.5 (0.3 - 0.9)
Latvia	Non-typhoidal Salmonella	12 (6 - 20)	0.3 (0.2 - 0.6)
Latvia	Other Klebsiella species	18 (10 - 32)	0.5 (0.3 - 0.9)
Latvia	Other enterococci	38 (23 - 63)	0.9 (0.5 - 1.5)
Latvia	Proteus spp.	50 (33 - 76)	1.2 (0.8 - 1.8)
Latvia	Providencia spp.	2 (1 - 3)	0.0 (0.0 - 0.1)
Latvia	Pseudomonas aeruginosa	178 (115 - 276)	4.5 (2.9 - 7.1)
Latvia	Salmonella Paratyphi	0 (0 - 0)	0.0 (0.0 - 0.0)
Latvia	Salmonella Typhi	4 (1 - 10)	0.1 (0.0 - 0.3)
Latvia	Serratia spp.	15 (9 - 24)	0.4 (0.2 - 0.7)
Latvia	Shigella spp.	0 (0 - 1)	0.0 (0.0 - 0.0)
Latvia	Staphylococcus aureus	393 (260 - 592)	10.4 (6.8 - 15.7)
Latvia	Streptococcus pneumoniae	151 (104 - 222)	4.4 (3.1 - 6.4)
Latvia	Vibrio cholerae	0 (0 - 0)	0.0 (0.0 - 0.0)
Lithuania	Acinetobacter baumannii	100 (51 - 176)	1.7 (0.9 - 3.0)
Lithuania	Aeromonas spp.	0 (0 - 0)	0.0 (0.0 - 0.0)
Lithuania	Campylobacter spp.	2 (0 - 4)	0.0 (0.0 - 0.1)
Lithuania	Chlamydia spp.	14 (10 - 20)	0.3 (0.2 - 0.4)
Lithuania	Citrobacter spp.	22 (13 - 36)	0.4 (0.2 - 0.7)
Lithuania	Clostridioides difficile	10 (5 - 18)	0.2 (0.1 - 0.4)
Lithuania	Enterobacter spp.	96 (61 - 143)	1.7 (1.1 - 2.6)
Lithuania	Enterococcus faecalis	160 (95 - 265)	3.0 (1.8 - 4.9)
Lithuania	Enterococcus faecium	165 (100 - 258)	3.0 (1.8 - 4.7)
Lithuania	Escherichia coli	777 (482 - 1,214)	13.4 (8.3 - 21.0)
Lithuania	Group A Streptococcus	102 (52 - 188)	2.1 (1.1 - 3.6)
Lithuania	Group B Streptococcus	76 (47 - 118)	1.4 (0.9 - 2.2)
Lithuania	Haemophilus influenzae	18 (13 - 25)	0.4 (0.3 - 0.5)

Lithuania	Klebsiella pneumoniae	357 (227 - 538)	6.4 (4.0 - 9.6)
Lithuania	Legionella spp.	25 (18 - 32)	0.5 (0.4 - 0.6)
Lithuania	Listeria monocytogenes	2 (1 - 3)	0.0 (0.0 - 0.1)
Lithuania	Morganella spp.	3 (2 - 5)	0.1 (0.0 - 0.1)
Lithuania	Mycoplasma spp.	20 (15 - 26)	0.5 (0.4 - 0.6)
Lithuania	Neisseria gonorrhoeae	1 (1 - 1)	0.0 (0.0 - 0.0)
Lithuania	Neisseria meningitidis	19 (10 - 33)	0.5 (0.3 - 0.9)
Lithuania	Non-typhoidal Salmonella	18 (10 - 31)	0.4 (0.2 - 0.6)
Lithuania	Other Klebsiella species	34 (19 - 56)	0.7 (0.4 - 1.1)
Lithuania	Other enterococci	50 (28 - 82)	0.8 (0.5 - 1.4)
Lithuania	Proteus spp.	72 (45 - 108)	1.2 (0.8 - 1.8)
Lithuania	Providencia spp.	2 (1 - 3)	0.0 (0.0 - 0.1)
Lithuania	Pseudomonas aeruginosa	262 (168 - 396)	4.7 (3.0 - 7.1)
Lithuania	Salmonella Paratyphi	0 (0 - 0)	0.0 (0.0 - 0.0)
Lithuania	Salmonella Typhi	7 (2 - 15)	0.2 (0.1 - 0.4)
Lithuania	Serratia spp.	24 (14 - 38)	0.4 (0.3 - 0.7)
Lithuania	Shigella spp.	1 (0 - 2)	0.0 (0.0 - 0.0)
Lithuania	Staphylococcus aureus	574 (380 - 854)	10.5 (6.9 - 15.6)
Lithuania	Streptococcus pneumoniae	218 (150 - 320)	4.3 (3.0 - 6.3)
Lithuania	Vibrio cholerae	0 (0 - 0)	0.0 (0.0 - 0.0)
Republic of Moldova	Acinetobacter baumannii	113 (60 - 197)	2.1 (1.1 - 3.6)
Republic of Moldova	Aeromonas spp.	0 (0 - 0)	0.0 (0.0 - 0.0)
Republic of Moldova	Campylobacter spp.	2 (0 - 6)	0.0 (0.0 - 0.1)
Republic of Moldova	Chlamydia spp.	23 (18 - 29)	0.5 (0.4 - 0.6)
Republic of Moldova	Citrobacter spp.	34 (20 - 54)	0.6 (0.4 - 1.0)
Republic of Moldova	Clostridioides difficile	4 (1 - 11)	0.1 (0.0 - 0.2)
Republic of Moldova	Enterobacter spp.	144 (94 - 213)	2.7 (1.7 - 3.9)
Republic of Moldova	Enterococcus faecalis	224 (134 - 343)	4.2 (2.5 - 6.3)
Republic of Moldova	Enterococcus faecium	228 (138 - 351)	4.1 (2.4 - 6.3)
Republic of Moldova	Escherichia coli	895 (569 - 1,358)	16.5 (10.5 - 25.1)
Republic of Moldova	Group A Streptococcus	120 (61 - 222)	2.5 (1.4 - 4.4)
Republic of Moldova	Group B Streptococcus	94 (63 - 139)	2.0 (1.4 - 2.9)
Republic of Moldova	Haemophilus influenzae	29 (22 - 37)	0.6 (0.5 - 0.8)
Republic of Moldova	Klebsiella pneumoniae	481 (317 - 710)	9.2 (6.1 - 13.4)
Republic of Moldova	Legionella spp.	30 (24 - 38)	0.7 (0.5 - 0.9)
Republic of Moldova	Listeria monocytogenes	3 (2 - 5)	0.1 (0.0 - 0.1)
Republic of Moldova	Morganella spp.	4 (2 - 6)	0.1 (0.0 - 0.1)
Republic of Moldova	Mycoplasma spp.	34 (28 - 40)	0.8 (0.7 - 1.0)
Republic of Moldova	Neisseria gonorrhoeae	1 (1 - 1)	0.0 (0.0 - 0.0)
Republic of Moldova	Neisseria meningitidis	33 (17 - 55)	0.8 (0.5 - 1.3)

Republic of Moldova	Non-typhoidal Salmonella	30 (17 - 50)	0.6 (0.4 - 1.0)
Republic of Moldova	Other Klebsiella species	61 (34 - 100)	1.1 (0.6 - 1.8)
Republic of Moldova	Other enterococci	52 (31 - 82)	1.0 (0.6 - 1.5)
Republic of Moldova	Proteus spp.	90 (58 - 137)	1.6 (1.0 - 2.5)
Republic of Moldova	Providencia spp.	3 (2 - 5)	0.1 (0.0 - 0.1)
Republic of Moldova	Pseudomonas aeruginosa	335 (221 - 484)	6.3 (4.2 - 9.1)
Republic of Moldova	Salmonella Paratyphi	0 (0 - 0)	0.0 (0.0 - 0.0)
Republic of Moldova	Salmonella Typhi	13 (5 - 31)	0.3 (0.1 - 0.7)
Republic of Moldova	Serratia spp.	37 (23 - 58)	0.7 (0.4 - 1.1)
Republic of Moldova	Shigella spp.	1 (0 - 3)	0.0 (0.0 - 0.1)
Republic of Moldova	Staphylococcus aureus	688 (485 - 976)	13.0 (9.2 - 18.3)
Republic of Moldova	Streptococcus pneumoniae	314 (237 - 415)	6.6 (5.1 - 8.6)
Republic of Moldova	Vibrio cholerae	0 (0 - 0)	0.0 (0.0 - 0.0)
Russian Federation	Acinetobacter baumannii	3,853 (1,982 - 6,811)	1.7 (0.9 - 3.0)
Russian Federation	Aeromonas spp.	6 (3 - 11)	0.0 (0.0 - 0.0)
Russian Federation	Campylobacter spp.	67 (15 - 189)	0.0 (0.0 - 0.1)
Russian Federation	Chlamydia spp.	713 (523 - 967)	0.3 (0.3 - 0.5)
Russian Federation	Citrobacter spp.	1,173 (667 - 1,945)	0.5 (0.3 - 0.9)
Russian Federation	Clostridioides difficile	408 (208 - 826)	0.2 (0.1 - 0.4)
Russian Federation	Enterobacter spp.	5,572 (3,629 - 8,020)	2.5 (1.6 - 3.6)
Russian Federation	Enterococcus faecalis	7,864 (4,673 - 12,169)	3.6 (2.2 - 5.6)
Russian Federation	Enterococcus faecium	7,876 (4,786 - 11,942)	3.5 (2.1 - 5.4)
Russian Federation	Escherichia coli	35,517 (22,519 - 53,561)	15.9 (10.1 - 24.0)
Russian Federation	Group A Streptococcus	6,294 (3,339 - 11,472)	3.1 (1.7 - 5.4)
Russian Federation	Group B Streptococcus	5,440 (3,572 - 7,997)	2.6 (1.7 - 3.8)
Russian Federation	Haemophilus influenzae	1,244 (952 - 1,643)	0.6 (0.5 - 0.8)
Russian Federation	Klebsiella pneumoniae	17,292 (11,421 - 25,027)	7.9 (5.2 - 11.4)
Russian Federation	Legionella spp.	741 (528 - 1,036)	0.4 (0.3 - 0.5)
Russian Federation	Listeria monocytogenes	115 (74 - 201)	0.1 (0.0 - 0.1)
Russian Federation	Morganella spp.	181 (116 - 278)	0.1 (0.0 - 0.1)
Russian Federation	Mycoplasma spp.	1,330 (1,078 - 1,673)	0.7 (0.6 - 0.9)
Russian Federation	Neisseria gonorrhoeae	85 (68 - 105)	0.0 (0.0 - 0.1)
Russian Federation	Neisseria meningitidis	1,375 (759 - 2,265)	0.8 (0.4 - 1.3)
Russian Federation	Non-typhoidal Salmonella	1,051 (596 - 1,727)	0.5 (0.3 - 0.8)
Russian Federation	Other Klebsiella species	1,644 (941 - 2,765)	0.8 (0.4 - 1.3)
Russian Federation	Other enterococci	2,538 (1,527 - 3,983)	1.1 (0.7 - 1.8)
Russian Federation	Proteus spp.	3,406 (2,213 - 4,960)	1.5 (1.0 - 2.2)
Russian Federation	Providencia spp.	111 (68 - 177)	0.0 (0.0 - 0.1)
Russian Federation	Pseudomonas aeruginosa	12,907 (8,537 - 18,837)	5.9 (3.8 - 8.5)
Russian Federation	Salmonella Paratyphi	3 (0 - 7)	0.0 (0.0 - 0.0)

Russian Federation	Salmonella Typhi	382 (131 - 887)	0.2 (0.1 - 0.5)
Russian Federation	Serratia spp.	1,208 (723 - 1,836)	0.5 (0.3 - 0.8)
Russian Federation	Shigella spp.	41 (16 - 88)	0.0 (0.0 - 0.1)
Russian Federation	Staphylococcus aureus	26,778 (17,864 - 38,497)	12.3 (8.2 - 17.6)
Russian Federation	Streptococcus pneumoniae	14,121 (10,465 - 19,011)	7.0 (5.2 - 9.3)
Russian Federation	Vibrio cholerae	0 (0 - 0)	0.0 (0.0 - 0.0)
Ukraine	Acinetobacter baumannii	2,938 (1,507 - 5,062)	4.1 (2.1 - 7.1)
Ukraine	Aeromonas spp.	2 (1 - 4)	0.0 (0.0 - 0.0)
Ukraine	Campylobacter spp.	21 (4 - 67)	0.0 (0.0 - 0.1)
Ukraine	Chlamydia spp.	444 (318 - 615)	0.7 (0.5 - 1.0)
Ukraine	Citrobacter spp.	762 (311 - 1,587)	1.1 (0.5 - 2.4)
Ukraine	Clostridioides difficile	122 (45 - 290)	0.2 (0.1 - 0.4)
Ukraine	Enterobacter spp.	2,268 (1,305 - 3,578)	3.3 (1.9 - 5.3)
Ukraine	Enterococcus faecalis	2,544 (1,402 - 4,009)	3.8 (2.1 - 6.0)
Ukraine	Enterococcus faecium	2,340 (1,317 - 3,569)	3.4 (1.9 - 5.2)
Ukraine	Escherichia coli	8,590 (5,050 - 12,846)	12.3 (7.2 - 18.4)
Ukraine	Group A Streptococcus	1,862 (874 - 3,560)	2.9 (1.5 - 5.3)
Ukraine	Group B Streptococcus	1,340 (819 - 2,109)	2.1 (1.3 - 3.2)
Ukraine	Haemophilus influenzae	317 (230 - 438)	0.5 (0.4 - 0.7)
Ukraine	Klebsiella pneumoniae	4,765 (2,876 - 7,170)	6.9 (4.2 - 10.4)
Ukraine	Legionella spp.	218 (155 - 296)	0.4 (0.3 - 0.5)
Ukraine	Listeria monocytogenes	35 (22 - 59)	0.1 (0.0 - 0.1)
Ukraine	Morganella spp.	47 (25 - 79)	0.1 (0.0 - 0.1)
Ukraine	Mycoplasma spp.	727 (563 - 925)	1.3 (1.1 - 1.7)
Ukraine	Neisseria gonorrhoeae	24 (18 - 31)	0.0 (0.0 - 0.0)
Ukraine	Neisseria meningitidis	742 (387 - 1,242)	1.4 (0.8 - 2.4)
Ukraine	Non-typhoidal Salmonella	1,062 (562 - 1,786)	1.7 (0.9 - 2.8)
Ukraine	Other Klebsiella species	572 (301 - 976)	0.9 (0.4 - 1.6)
Ukraine	Other enterococci	979 (514 - 1,680)	1.4 (0.7 - 2.4)
Ukraine	Proteus spp.	1,031 (611 - 1,564)	1.4 (0.8 - 2.2)
Ukraine	Providencia spp.	32 (16 - 55)	0.0 (0.0 - 0.1)
Ukraine	Pseudomonas aeruginosa	3,781 (2,267 - 5,707)	5.5 (3.3 - 8.3)
Ukraine	Salmonella Paratyphi	0 (0 - 0)	0.0 (0.0 - 0.0)
Ukraine	Salmonella Typhi	94 (30 - 223)	0.2 (0.1 - 0.4)
Ukraine	Serratia spp.	536 (301 - 848)	0.8 (0.4 - 1.2)
Ukraine	Shigella spp.	12 (4 - 29)	0.0 (0.0 - 0.1)
Ukraine	Staphylococcus aureus	6,740 (4,078 - 10,062)	10.0 (6.0 - 14.9)
Ukraine	Streptococcus pneumoniae	4,566 (3,257 - 6,290)	7.5 (5.5 - 10.2)
Ukraine	Vibrio cholerae	0 (0 - 0)	0.0 (0.0 - 0.0)
Australia	Acinetobacter baumannii	861 (484 - 1,399)	2.0 (1.1 - 3.2)

Australia	Aeromonas spp.	1 (0 - 1)	0.0 (0.0 - 0.0)
Australia	Campylobacter spp.	10 (2 - 26)	0.0 (0.0 - 0.1)
Australia	Chlamydia spp.	85 (66 - 109)	0.2 (0.1 - 0.2)
Australia	Citrobacter spp.	90 (59 - 132)	0.2 (0.1 - 0.3)
Australia	Clostridioides difficile	198 (140 - 281)	0.4 (0.3 - 0.6)
Australia	Enterobacter spp.	640 (421 - 924)	1.5 (1.0 - 2.2)
Australia	Enterococcus faecalis	656 (397 - 1,060)	1.5 (0.9 - 2.4)
Australia	Enterococcus faecium	810 (502 - 1,211)	1.9 (1.2 - 2.9)
Australia	Escherichia coli	2,723 (1,882 - 3,790)	6.0 (4.1 - 8.5)
Australia	Group A Streptococcus	629 (349 - 1,099)	1.5 (0.8 - 2.5)
Australia	Group B Streptococcus	667 (423 - 1,002)	1.6 (1.0 - 2.4)
Australia	Haemophilus influenzae	111 (85 - 144)	0.2 (0.2 - 0.3)
Australia	Klebsiella pneumoniae	1,524 (1,029 - 2,147)	3.4 (2.3 - 4.9)
Australia	Legionella spp.	250 (197 - 312)	0.5 (0.4 - 0.7)
Australia	Listeria monocytogenes	7 (4 - 15)	0.0 (0.0 - 0.0)
Australia	Morganella spp.	15 (11 - 21)	0.0 (0.0 - 0.0)
Australia	Mycoplasma spp.	154 (121 - 194)	0.4 (0.3 - 0.5)
Australia	Neisseria gonorrhoeae	4 (3 - 5)	0.0 (0.0 - 0.0)
Australia	Neisseria meningitidis	45 (24 - 73)	0.1 (0.1 - 0.2)
Australia	Non-typhoidal Salmonella	21 (13 - 33)	0.1 (0.0 - 0.1)
Australia	Other Klebsiella species	121 (66 - 210)	0.3 (0.2 - 0.5)
Australia	Other enterococci	302 (205 - 422)	0.7 (0.4 - 0.9)
Australia	Proteus spp.	304 (212 - 420)	0.7 (0.5 - 0.9)
Australia	Providencia spp.	6 (4 - 8)	0.0 (0.0 - 0.0)
Australia	Pseudomonas aeruginosa	1,572 (1,077 - 2,207)	3.5 (2.4 - 5.0)
Australia	Salmonella Paratyphi	0 (0 - 0)	0.0 (0.0 - 0.0)
Australia	Salmonella Typhi	5 (3 - 8)	0.0 (0.0 - 0.0)
Australia	Serratia spp.	141 (83 - 220)	0.3 (0.2 - 0.5)
Australia	Shigella spp.	3 (1 - 7)	0.0 (0.0 - 0.0)
Australia	Staphylococcus aureus	4,414 (2,982 - 6,266)	10.1 (6.7 - 14.4)
Australia	Streptococcus pneumoniae	1,076 (828 - 1,397)	2.5 (1.9 - 3.2)
Australia	Vibrio cholerae	0 (0 - 0)	0.0 (0.0 - 0.0)
New Zealand	Acinetobacter baumannii	171 (97 - 277)	2.2 (1.2 - 3.5)
New Zealand	Aeromonas spp.	0 (0 - 0)	0.0 (0.0 - 0.0)
New Zealand	Campylobacter spp.	3 (1 - 9)	0.0 (0.0 - 0.1)
New Zealand	Chlamydia spp.	19 (14 - 24)	0.2 (0.2 - 0.3)
New Zealand	Citrobacter spp.	17 (11 - 25)	0.2 (0.1 - 0.3)
New Zealand	Clostridioides difficile	53 (38 - 72)	0.6 (0.5 - 0.9)
New Zealand	Enterobacter spp.	124 (81 - 180)	1.6 (1.0 - 2.3)
New Zealand	Enterococcus faecalis	116 (69 - 188)	1.4 (0.9 - 2.3)
New Zealand	Enterococcus faecium	147 (89 - 224)	1.9 (1.1 - 2.9)

New Zealand	Escherichia coli	498 (342 - 707)	6.0 (4.1 - 8.6)
New Zealand	Group A Streptococcus	119 (66 - 208)	1.5 (0.9 - 2.6)
New Zealand	Group B Streptococcus	128 (82 - 191)	1.7 (1.1 - 2.5)
New Zealand	Haemophilus influenzae	24 (18 - 32)	0.3 (0.2 - 0.4)
New Zealand	Klebsiella pneumoniae	301 (204 - 434)	3.7 (2.5 - 5.4)
New Zealand	Legionella spp.	48 (37 - 60)	0.6 (0.4 - 0.7)
New Zealand	Listeria monocytogenes	2 (1 - 3)	0.0 (0.0 - 0.1)
New Zealand	Morganella spp.	3 (2 - 4)	0.0 (0.0 - 0.0)
New Zealand	Mycoplasma spp.	30 (24 - 39)	0.4 (0.3 - 0.5)
New Zealand	Neisseria gonorrhoeae	1 (0 - 1)	0.0 (0.0 - 0.0)
New Zealand	Neisseria meningitidis	10 (6 - 16)	0.2 (0.1 - 0.3)
New Zealand	Non-typhoidal Salmonella	5 (2 - 7)	0.1 (0.0 - 0.1)
New Zealand	Other Klebsiella species	21 (11 - 37)	0.3 (0.1 - 0.5)
New Zealand	Other enterococci	57 (38 - 81)	0.7 (0.5 - 1.0)
New Zealand	Proteus spp.	57 (39 - 80)	0.7 (0.5 - 1.0)
New Zealand	Providencia spp.	1 (1 - 2)	0.0 (0.0 - 0.0)
New Zealand	Pseudomonas aeruginosa	308 (210 - 436)	3.8 (2.5 - 5.4)
New Zealand	Salmonella Paratyphi	0 (0 - 0)	0.0 (0.0 - 0.0)
New Zealand	Salmonella Typhi	1 (1 - 2)	0.0 (0.0 - 0.0)
New Zealand	Serratia spp.	29 (17 - 46)	0.4 (0.2 - 0.6)
New Zealand	Shigella spp.	1 (0 - 3)	0.0 (0.0 - 0.0)
New Zealand	Staphylococcus aureus	843 (580 - 1,202)	10.4 (7.0 - 15.0)
New Zealand	Streptococcus pneumoniae	228 (173 - 298)	2.8 (2.1 - 3.7)
New Zealand	Vibrio cholerae	0 (0 - 0)	0.0 (0.0 - 0.0)
Brunei Darussalam	Acinetobacter baumannii	28 (16 - 44)	12.9 (7.5 - 20.0)
Brunei Darussalam	Aeromonas spp.	0 (0 - 0)	0.0 (0.0 - 0.0)
Brunei Darussalam	Campylobacter spp.	0 (0 - 0)	0.0 (0.0 - 0.1)
Brunei Darussalam	Chlamydia spp.	4 (3 - 5)	2.2 (1.7 - 2.8)
Brunei Darussalam	Citrobacter spp.	3 (1 - 4)	1.1 (0.7 - 1.7)
Brunei Darussalam	Clostridioides difficile	2 (1 - 3)	1.1 (0.6 - 1.9)
Brunei Darussalam	Enterobacter spp.	17 (11 - 26)	7.2 (4.5 - 10.8)
Brunei Darussalam	Enterococcus faecalis	7 (5 - 11)	3.3 (2.1 - 5.1)
Brunei Darussalam	Enterococcus faecium	7 (4 - 11)	3.2 (2.1 - 4.8)
Brunei Darussalam	Escherichia coli	25 (18 - 35)	13.8 (10.4 - 17.9)
Brunei Darussalam	Group A Streptococcus	7 (4 - 12)	3.4 (2.0 - 5.8)
Brunei Darussalam	Group B Streptococcus	7 (5 - 10)	3.3 (2.3 - 4.6)
Brunei Darussalam	Haemophilus influenzae	2 (2 - 3)	1.2 (0.9 - 1.5)
Brunei Darussalam	Klebsiella pneumoniae	20 (13 - 28)	9.9 (7.2 - 13.5)
Brunei Darussalam	Legionella spp.	2 (2 - 3)	1.1 (0.9 - 1.4)
Brunei Darussalam	Listeria monocytogenes	0 (0 - 0)	0.1 (0.0 - 0.1)
Brunei Darussalam	Morganella spp.	0 (0 - 0)	0.2 (0.1 - 0.3)

Brunei Darussalam	Mycoplasma spp.	5 (4 - 6)	2.0 (1.7 - 2.5)
Brunei Darussalam	Neisseria gonorrhoeae	0 (0 - 0)	0.1 (0.1 - 0.1)
Brunei Darussalam	Neisseria meningitidis	3 (2 - 5)	0.9 (0.5 - 1.4)
Brunei Darussalam	Non-typhoidal Salmonella	2 (1 - 3)	0.8 (0.5 - 1.3)
Brunei Darussalam	Other Klebsiella species	2 (1 - 3)	0.7 (0.4 - 1.2)
Brunei Darussalam	Other enterococci	4 (3 - 6)	2.5 (1.8 - 3.4)
Brunei Darussalam	Proteus spp.	4 (3 - 5)	2.2 (1.6 - 2.9)
Brunei Darussalam	Providencia spp.	0 (0 - 0)	0.1 (0.1 - 0.2)
Brunei Darussalam	Pseudomonas aeruginosa	19 (13 - 27)	9.5 (6.7 - 13.1)
Brunei Darussalam	Salmonella Paratyphi	0 (0 - 0)	0.0 (0.0 - 0.0)
Brunei Darussalam	Salmonella Typhi	0 (0 - 1)	0.1 (0.0 - 0.2)
Brunei Darussalam	Serratia spp.	4 (2 - 6)	1.5 (0.9 - 2.3)
Brunei Darussalam	Shigella spp.	0 (0 - 0)	0.0 (0.0 - 0.0)
Brunei Darussalam	Staphylococcus aureus	31 (22 - 43)	15.3 (11.3 - 20.6)
Brunei Darussalam	Streptococcus pneumoniae	24 (19 - 32)	11.9 (9.5 - 14.9)
Brunei Darussalam	Vibrio cholerae	0 (0 - 0)	0.0 (0.0 - 0.0)
Japan	Acinetobacter baumannii	6,750 (4,041 - 10,591)	1.7 (1.0 - 2.6)
Japan	Aeromonas spp.	9 (4 - 17)	0.0 (0.0 - 0.0)
Japan	Campylobacter spp.	127 (22 - 342)	0.0 (0.0 - 0.1)
Japan	Chlamydia spp.	1,806 (1,455 - 2,097)	0.4 (0.3 - 0.4)
Japan	Citrobacter spp.	725 (477 - 1,055)	0.2 (0.1 - 0.3)
Japan	Clostridioides difficile	1,926 (1,297 - 2,717)	0.5 (0.3 - 0.6)
Japan	Enterobacter spp.	6,021 (4,323 - 8,140)	1.5 (1.0 - 2.0)
Japan	Enterococcus faecalis	5,271 (3,086 - 8,759)	1.3 (0.8 - 2.1)
Japan	Enterococcus faecium	6,565 (4,079 - 9,801)	1.7 (1.1 - 2.5)
Japan	Escherichia coli	24,703 (17,465 - 34,277)	5.5 (3.8 - 7.7)
Japan	Group A Streptococcus	3,153 (1,438 - 6,110)	0.8 (0.4 - 1.6)
Japan	Group B Streptococcus	5,513 (3,944 - 7,702)	1.4 (1.0 - 2.0)
Japan	Haemophilus influenzae	2,236 (1,794 - 2,569)	0.5 (0.4 - 0.5)
Japan	Klebsiella pneumoniae	16,203 (11,939 - 21,508)	3.6 (2.6 - 4.9)
Japan	Legionella spp.	5,464 (4,364 - 6,258)	1.1 (0.9 - 1.2)
Japan	Listeria monocytogenes	55 (37 - 96)	0.0 (0.0 - 0.0)
Japan	Morganella spp.	119 (86 - 168)	0.0 (0.0 - 0.0)
Japan	Mycoplasma spp.	3,039 (2,429 - 3,462)	0.7 (0.6 - 0.7)
Japan	Neisseria gonorrhoeae	77 (57 - 90)	0.0 (0.0 - 0.0)
Japan	Neisseria meningitidis	261 (147 - 420)	0.1 (0.1 - 0.2)
Japan	Non-typhoidal Salmonella	173 (81 - 328)	0.0 (0.0 - 0.1)
Japan	Other Klebsiella species	1,070 (548 - 1,917)	0.3 (0.2 - 0.5)
Japan	Other enterococci	2,164 (1,469 - 3,074)	0.5 (0.3 - 0.7)
Japan	Proteus spp.	2,450 (1,673 - 3,437)	0.5 (0.4 - 0.8)

Japan	Providencia spp.	46 (32 - 68)	0.0 (0.0 - 0.0)
Japan	Pseudomonas aeruginosa	17,814 (13,500 - 23,026)	3.9 (3.0 - 5.2)
Japan	Salmonella Paratyphi	1 (0 - 1)	0.0 (0.0 - 0.0)
Japan	Salmonella Typhi	34 (18 - 56)	0.0 (0.0 - 0.0)
Japan	Serratia spp.	1,096 (651 - 1,683)	0.3 (0.2 - 0.5)
Japan	Shigella spp.	19 (6 - 46)	0.0 (0.0 - 0.0)
Japan	Staphylococcus aureus	55,565 (43,405 - 70,144)	12.2 (9.3 - 15.9)
Japan	Streptococcus pneumoniae	18,682 (15,243 - 21,649)	3.9 (3.3 - 4.6)
Japan	Vibrio cholerae	0 (0 - 0)	0.0 (0.0 - 0.0)
Republic of Korea	Acinetobacter baumannii	1,812 (1,106 - 2,804)	2.2 (1.3 - 3.4)
Republic of Korea	Aeromonas spp.	2 (1 - 5)	0.0 (0.0 - 0.0)
Republic of Korea	Campylobacter spp.	8 (1 - 22)	0.0 (0.0 - 0.0)
Republic of Korea	Chlamydia spp.	267 (182 - 324)	0.3 (0.2 - 0.4)
Republic of Korea	Citrobacter spp.	215 (139 - 312)	0.3 (0.2 - 0.4)
Republic of Korea	Clostridioides difficile	654 (359 - 1,222)	0.8 (0.4 - 1.5)
Republic of Korea	Enterobacter spp.	1,534 (1,063 - 2,143)	1.9 (1.3 - 2.6)
Republic of Korea	Enterococcus faecalis	1,478 (870 - 2,380)	1.8 (1.0 - 2.9)
Republic of Korea	Enterococcus faecium	1,959 (1,260 - 2,897)	2.3 (1.5 - 3.4)
Republic of Korea	Escherichia coli	5,850 (4,021 - 8,219)	7.0 (4.8 - 9.9)
Republic of Korea	Group A Streptococcus	1,074 (531 - 2,002)	1.4 (0.7 - 2.4)
Republic of Korea	Group B Streptococcus	1,437 (982 - 2,039)	1.8 (1.2 - 2.5)
Republic of Korea	Haemophilus influenzae	340 (233 - 413)	0.4 (0.3 - 0.5)
Republic of Korea	Klebsiella pneumoniae	3,737 (2,660 - 5,141)	4.5 (3.2 - 6.3)
Republic of Korea	Legionella spp.	738 (480 - 881)	0.9 (0.6 - 1.1)
Republic of Korea	Listeria monocytogenes	16 (8 - 35)	0.0 (0.0 - 0.0)
Republic of Korea	Morganella spp.	23 (14 - 37)	0.0 (0.0 - 0.0)
Republic of Korea	Mycoplasma spp.	443 (301 - 531)	0.6 (0.4 - 0.7)
Republic of Korea	Neisseria gonorrhoeae	11 (7 - 14)	0.0 (0.0 - 0.0)
Republic of Korea	Neisseria meningitidis	126 (72 - 198)	0.2 (0.1 - 0.3)
Republic of Korea	Non-typhoidal Salmonella	59 (36 - 91)	0.1 (0.0 - 0.1)
Republic of Korea	Other Klebsiella species	330 (185 - 558)	0.4 (0.2 - 0.7)
Republic of Korea	Other enterococci	544 (356 - 775)	0.7 (0.4 - 0.9)
Republic of Korea	Proteus spp.	606 (408 - 853)	0.7 (0.5 - 1.0)
Republic of Korea	Providencia spp.	10 (5 - 17)	0.0 (0.0 - 0.0)
Republic of Korea	Pseudomonas aeruginosa	3,864 (2,808 - 5,248)	4.7 (3.4 - 6.4)
Republic of Korea	Salmonella Paratyphi	0 (0 - 0)	0.0 (0.0 - 0.0)
Republic of Korea	Salmonella Typhi	15 (9 - 25)	0.0 (0.0 - 0.0)
Republic of Korea	Serratia spp.	360 (225 - 528)	0.4 (0.3 - 0.6)
Republic of Korea	Shigella spp.	3 (1 - 8)	0.0 (0.0 - 0.0)
Republic of Korea	Staphylococcus aureus	11,015 (8,183 - 14,687)	13.4 (9.9 - 17.9)

Republic of Korea	Streptococcus pneumoniae	3,149 (2,379 - 3,876)	4.0 (3.0 - 4.9)
Republic of Korea	Vibrio cholerae	0 (0 - 0)	0.0 (0.0 - 0.0)
Singapore	Acinetobacter baumannii	135 (85 - 203)	1.8 (1.2 - 2.8)
Singapore	Aeromonas spp.	0 (0 - 0)	0.0 (0.0 - 0.0)
Singapore	Campylobacter spp.	0 (0 - 1)	0.0 (0.0 - 0.0)
Singapore	Chlamydia spp.	56 (48 - 63)	0.8 (0.7 - 0.9)
Singapore	Citrobacter spp.	15 (11 - 20)	0.2 (0.1 - 0.3)
Singapore	Clostridioides difficile	30 (22 - 41)	0.4 (0.3 - 0.6)
Singapore	Enterobacter spp.	132 (102 - 169)	1.8 (1.4 - 2.3)
Singapore	Enterococcus faecalis	99 (70 - 140)	1.4 (1.0 - 1.9)
Singapore	Enterococcus faecium	101 (65 - 145)	1.3 (0.9 - 2.0)
Singapore	Escherichia coli	501 (402 - 623)	7.0 (5.6 - 8.8)
Singapore	Group A Streptococcus	87 (52 - 150)	1.2 (0.7 - 2.1)
Singapore	Group B Streptococcus	137 (107 - 176)	1.9 (1.5 - 2.5)
Singapore	Haemophilus influenzae	67 (57 - 75)	1.0 (0.8 - 1.1)
Singapore	Klebsiella pneumoniae	350 (287 - 429)	4.9 (4.0 - 6.0)
Singapore	Legionella spp.	143 (121 - 159)	2.0 (1.7 - 2.3)
Singapore	Listeria monocytogenes	1 (0 - 2)	0.0 (0.0 - 0.0)
Singapore	Morganella spp.	4 (3 - 5)	0.1 (0.0 - 0.1)
Singapore	Mycoplasma spp.	97 (82 - 108)	1.4 (1.2 - 1.6)
Singapore	Neisseria gonorrhoeae	1 (0 - 1)	0.0 (0.0 - 0.0)
Singapore	Neisseria meningitidis	8 (4 - 12)	0.1 (0.1 - 0.2)
Singapore	Non-typhoidal Salmonella	4 (2 - 8)	0.1 (0.0 - 0.1)
Singapore	Other Klebsiella species	13 (7 - 22)	0.2 (0.1 - 0.3)
Singapore	Other enterococci	55 (42 - 70)	0.8 (0.6 - 1.0)
Singapore	Proteus spp.	52 (41 - 66)	0.7 (0.6 - 0.9)
Singapore	Providencia spp.	2 (1 - 2)	0.0 (0.0 - 0.0)
Singapore	Pseudomonas aeruginosa	391 (323 - 472)	5.5 (4.6 - 6.7)
Singapore	Salmonella Paratyphi	0 (0 - 0)	0.0 (0.0 - 0.0)
Singapore	Salmonella Typhi	1 (1 - 1)	0.0 (0.0 - 0.0)
Singapore	Serratia spp.	22 (14 - 32)	0.3 (0.2 - 0.4)
Singapore	Shigella spp.	0 (0 - 1)	0.0 (0.0 - 0.0)
Singapore	Staphylococcus aureus	1,211 (1,008 - 1,437)	17.2 (14.3 - 20.3)
Singapore	Streptococcus pneumoniae	552 (474 - 618)	7.9 (6.8 - 8.9)
Singapore	Vibrio cholerae	0 (0 - 0)	0.0 (0.0 - 0.0)
Canada	Acinetobacter baumannii	1,539 (873 - 2,480)	2.2 (1.3 - 3.6)
Canada	Aeromonas spp.	9 (4 - 15)	0.0 (0.0 - 0.0)
Canada	Campylobacter spp.	33 (6 - 91)	0.0 (0.0 - 0.1)
Canada	Chlamydia spp.	188 (150 - 236)	0.3 (0.2 - 0.3)
Canada	Citrobacter spp.	170 (112 - 245)	0.2 (0.2 - 0.4)

Canada	Clostridioides difficile	1,004 (730 - 1,354)	1.4 (1.0 - 1.8)
Canada	Enterobacter spp.	1,232 (810 - 1,757)	1.8 (1.2 - 2.6)
Canada	Enterococcus faecalis	1,141 (706 - 1,832)	1.6 (1.0 - 2.6)
Canada	Enterococcus faecium	1,367 (859 - 2,019)	2.0 (1.2 - 2.9)
Canada	Escherichia coli	4,593 (3,213 - 6,293)	6.3 (4.4 - 8.7)
Canada	Group A Streptococcus	819 (432 - 1,494)	1.2 (0.7 - 2.2)
Canada	Group B Streptococcus	1,058 (695 - 1,564)	1.6 (1.0 - 2.4)
Canada	Haemophilus influenzae	231 (184 - 291)	0.3 (0.3 - 0.4)
Canada	Klebsiella pneumoniae	2,623 (1,822 - 3,669)	3.7 (2.5 - 5.2)
Canada	Legionella spp.	448 (364 - 546)	0.6 (0.5 - 0.7)
Canada	Listeria monocytogenes	13 (8 - 25)	0.0 (0.0 - 0.0)
Canada	Morganella spp.	27 (20 - 36)	0.0 (0.0 - 0.0)
Canada	Mycoplasma spp.	334 (268 - 411)	0.5 (0.4 - 0.6)
Canada	Neisseria gonorrhoeae	9 (7 - 10)	0.0 (0.0 - 0.0)
Canada	Neisseria meningitidis	74 (41 - 121)	0.2 (0.1 - 0.3)
Canada	Non-typhoidal Salmonella	50 (21 - 103)	0.1 (0.0 - 0.1)
Canada	Other Klebsiella species	222 (126 - 380)	0.3 (0.2 - 0.6)
Canada	Other enterococci	489 (335 - 676)	0.7 (0.5 - 0.9)
Canada	Proteus spp.	507 (358 - 692)	0.7 (0.5 - 0.9)
Canada	Providencia spp.	10 (7 - 14)	0.0 (0.0 - 0.0)
Canada	Pseudomonas aeruginosa	2,747 (1,902 - 3,824)	3.8 (2.6 - 5.4)
Canada	Salmonella Paratyphi	0 (0 - 0)	0.0 (0.0 - 0.0)
Canada	Salmonella Typhi	7 (4 - 12)	0.0 (0.0 - 0.0)
Canada	Serratia spp.	247 (146 - 383)	0.4 (0.2 - 0.6)
Canada	Shigella spp.	10 (4 - 25)	0.0 (0.0 - 0.0)
Canada	Staphylococcus aureus	7,278 (5,068 - 10,256)	10.2 (7.0 - 14.6)
Canada	Streptococcus pneumoniae	2,153 (1,721 - 2,738)	3.0 (2.4 - 3.9)
Canada	Vibrio cholerae	0 (0 - 0)	0.0 (0.0 - 0.0)
Greenland	Acinetobacter baumannii	3 (2 - 5)	5.7 (3.1 - 9.1)
Greenland	Aeromonas spp.	0 (0 - 0)	0.0 (0.0 - 0.0)
Greenland	Campylobacter spp.	0 (0 - 0)	0.1 (0.0 - 0.2)
Greenland	Chlamydia spp.	0 (0 - 1)	0.7 (0.5 - 1.0)
Greenland	Citrobacter spp.	0 (0 - 1)	0.6 (0.4 - 0.9)
Greenland	Clostridioides difficile	1 (0 - 1)	1.6 (0.8 - 2.6)
Greenland	Enterobacter spp.	2 (1 - 3)	3.5 (2.2 - 5.2)
Greenland	Enterococcus faecalis	2 (1 - 3)	3.4 (2.0 - 5.3)
Greenland	Enterococcus faecium	2 (1 - 4)	3.8 (2.3 - 5.7)
Greenland	Escherichia coli	8 (6 - 12)	14.9 (10.1 - 21.0)
Greenland	Group A Streptococcus	1 (1 - 2)	2.3 (1.3 - 4.1)
Greenland	Group B Streptococcus	2 (1 - 3)	3.0 (2.0 - 4.4)
Greenland	Haemophilus influenzae	0 (0 - 1)	0.9 (0.7 - 1.3)

Greenland	Klebsiella pneumoniae	7 (4 - 10)	11.6 (7.6 - 16.6)
Greenland	Legionella spp.	0 (0 - 1)	0.8 (0.6 - 1.1)
Greenland	Listeria monocytogenes	0 (0 - 0)	0.1 (0.1 - 0.2)
Greenland	Morganella spp.	0 (0 - 0)	0.1 (0.1 - 0.2)
Greenland	Mycoplasma spp.	0 (0 - 0)	0.7 (0.5 - 0.9)
Greenland	Neisseria gonorrhoeae	0 (0 - 0)	0.0 (0.0 - 0.0)
Greenland	Neisseria meningitidis	0 (0 - 1)	0.8 (0.4 - 1.2)
Greenland	Non-typhoidal Salmonella	0 (0 - 0)	0.4 (0.2 - 0.7)
Greenland	Other Klebsiella species	1 (0 - 1)	0.8 (0.4 - 1.4)
Greenland	Other enterococci	1 (1 - 1)	1.7 (1.2 - 2.3)
Greenland	Proteus spp.	1 (1 - 2)	2.0 (1.4 - 2.9)
Greenland	Providencia spp.	0 (0 - 0)	0.1 (0.1 - 0.2)
Greenland	Pseudomonas aeruginosa	5 (3 - 8)	9.2 (6.0 - 13.5)
Greenland	Salmonella Paratyphi	0 (0 - 0)	0.0 (0.0 - 0.0)
Greenland	Salmonella Typhi	0 (0 - 0)	0.2 (0.1 - 0.4)
Greenland	Serratia spp.	1 (0 - 1)	1.2 (0.7 - 1.9)
Greenland	Shigella spp.	0 (0 - 0)	0.0 (0.0 - 0.1)
Greenland	Staphylococcus aureus	11 (7 - 15)	18.9 (13.0 - 25.9)
Greenland	Streptococcus pneumoniae	4 (3 - 6)	7.8 (5.5 - 10.5)
Greenland	Vibrio cholerae	0 (0 - 0)	0.0 (0.0 - 0.0)
United States of America	Acinetobacter baumannii	15,506 (8,927 - 24,746)	2.8 (1.6 - 4.5)
United States of America	Aeromonas spp.	29 (13 - 54)	0.0 (0.0 - 0.0)
United States of America	Campylobacter spp.	737 (145 - 1,811)	0.1 (0.0 - 0.3)
United States of America	Chlamydia spp.	1,821 (1,426 - 2,345)	0.3 (0.2 - 0.4)
United States of America	Citrobacter spp.	1,786 (1,189 - 2,567)	0.3 (0.2 - 0.5)
United States of America	Clostridioides difficile	8,552 (6,765 - 10,304)	1.4 (1.1 - 1.7)
United States of America	Enterobacter spp.	12,304 (8,168 - 17,566)	2.2 (1.5 - 3.2)
United States of America	Enterococcus faecalis	12,095 (7,547 - 18,594)	2.2 (1.4 - 3.3)
United States of America	Enterococcus faecium	14,502 (9,078 - 21,694)	2.6 (1.6 - 3.9)
United States of America	Escherichia coli	49,182 (34,825 - 67,695)	8.5 (6.0 - 11.8)
United States of America	Group A Streptococcus	9,047 (4,910 - 16,037)	1.7 (0.9 - 2.9)
United States of America	Group B Streptococcus	10,947 (7,278 - 15,999)	2.1 (1.3 - 3.0)
United States of America	Haemophilus influenzae	2,404 (1,864 - 3,140)	0.4 (0.3 - 0.6)
United States of America	Klebsiella pneumoniae	30,250 (20,899 - 42,871)	5.3 (3.6 - 7.6)
United States of America	Legionella spp.	3,991 (3,241 - 4,967)	0.7 (0.6 - 0.9)
United States of America	Listeria monocytogenes	146 (93 - 270)	0.0 (0.0 - 0.1)
United States of America	Morganella spp.	320 (242 - 435)	0.1 (0.0 - 0.1)
United States of America	Mycoplasma spp.	2,868 (2,307 - 3,615)	0.5 (0.4 - 0.7)
United States of America	Neisseria gonorrhoeae	80 (71 - 85)	0.0 (0.0 - 0.0)
United States of America	Neisseria meningitidis	1,044 (577 - 1,697)	0.3 (0.1 - 0.4)

United States of America	Non-typhoidal Salmonella	980 (319 - 2,272)	0.2 (0.1 - 0.4)
United States of America	Other Klebsiella species	2,426 (1,416 - 3,969)	0.5 (0.3 - 0.8)
United States of America	Other enterococci	5,251 (3,683 - 7,146)	0.9 (0.6 - 1.2)
United States of America	Proteus spp.	5,707 (4,072 - 7,770)	1.0 (0.7 - 1.3)
United States of America	Providencia spp.	144 (102 - 206)	0.0 (0.0 - 0.0)
United States of America	Pseudomonas aeruginosa	29,751 (20,267 - 41,892)	5.3 (3.5 - 7.5)
United States of America	Salmonella Paratyphi	2 (1 - 2)	0.0 (0.0 - 0.0)
United States of America	Salmonella Typhi	153 (82 - 252)	0.0 (0.0 - 0.1)
United States of America	Serratia spp.	2,964 (1,769 - 4,576)	0.6 (0.3 - 0.8)
United States of America	Shigella spp.	333 (123 - 719)	0.1 (0.0 - 0.1)
United States of America	Staphylococcus aureus	73,807 (51,646 - 103,153)	13.0 (9.0 - 18.3)
United States of America	Streptococcus pneumoniae	21,995 (17,229 - 28,495)	4.0 (3.1 - 5.2)
United States of America	Vibrio cholerae	0 (0 - 0)	0.0 (0.0 - 0.0)
Argentina	Acinetobacter baumannii	2,605 (1,579 - 4,032)	4.8 (2.9 - 7.5)
Argentina	Aeromonas spp.	3 (1 - 4)	0.0 (0.0 - 0.0)
Argentina	Campylobacter spp.	17 (4 - 42)	0.0 (0.0 - 0.1)
Argentina	Chlamydia spp.	685 (586 - 807)	1.3 (1.1 - 1.5)
Argentina	Citrobacter spp.	298 (203 - 423)	0.6 (0.4 - 0.8)
Argentina	Clostridioides difficile	319 (217 - 446)	0.6 (0.4 - 0.8)
Argentina	Enterobacter spp.	2,104 (1,540 - 2,815)	3.9 (2.9 - 5.3)
Argentina	Enterococcus faecalis	1,736 (1,105 - 2,614)	3.2 (2.1 - 4.8)
Argentina	Enterococcus faecium	1,831 (1,183 - 2,681)	3.4 (2.2 - 5.0)
Argentina	Escherichia coli	8,291 (6,336 - 10,710)	15.2 (11.5 - 19.6)
Argentina	Group A Streptococcus	1,737 (1,085 - 2,774)	3.2 (2.0 - 5.1)
Argentina	Group B Streptococcus	2,113 (1,630 - 2,773)	4.0 (3.1 - 5.2)
Argentina	Haemophilus influenzae	834 (726 - 963)	1.6 (1.4 - 1.8)
Argentina	Klebsiella pneumoniae	6,717 (5,159 - 8,671)	12.4 (9.5 - 16.1)
Argentina	Legionella spp.	785 (670 - 920)	1.5 (1.2 - 1.7)
Argentina	Listeria monocytogenes	38 (28 - 55)	0.1 (0.1 - 0.1)
Argentina	Morganella spp.	78 (54 - 104)	0.1 (0.1 - 0.2)
Argentina	Mycoplasma spp.	748 (659 - 854)	1.4 (1.3 - 1.6)
Argentina	Neisseria gonorrhoeae	16 (14 - 17)	0.0 (0.0 - 0.0)
Argentina	Neisseria meningitidis	315 (190 - 485)	0.7 (0.4 - 1.1)
Argentina	Non-typhoidal Salmonella	166 (99 - 264)	0.3 (0.2 - 0.5)
Argentina	Other Klebsiella species	394 (220 - 665)	0.7 (0.4 - 1.2)
Argentina	Other enterococci	937 (676 - 1,242)	1.7 (1.2 - 2.3)
Argentina	Proteus spp.	1,060 (780 - 1,398)	1.9 (1.4 - 2.5)
Argentina	Providencia spp.	52 (36 - 74)	0.1 (0.1 - 0.1)
Argentina	Pseudomonas aeruginosa	5,699 (4,404 - 7,356)	10.5 (8.1 - 13.6)
Argentina	Salmonella Paratyphi	0 (0 - 0)	0.0 (0.0 - 0.0)

Argentina	Salmonella Typhi	86 (51 - 136)	0.2 (0.1 - 0.3)
Argentina	Serratia spp.	553 (345 - 844)	1.1 (0.7 - 1.6)
Argentina	Shigella spp.	42 (17 - 87)	0.1 (0.0 - 0.2)
Argentina	Staphylococcus aureus	13,641 (11,285 - 16,540)	25.0 (20.6 - 30.4)
Argentina	Streptococcus pneumoniae	7,048 (6,160 - 8,107)	13.2 (11.5 - 15.1)
Argentina	Vibrio cholerae	0 (0 - 0)	0.0 (0.0 - 0.0)
Chile	Acinetobacter baumannii	666 (385 - 1,063)	2.9 (1.7 - 4.6)
Chile	Aeromonas spp.	1 (1 - 3)	0.0 (0.0 - 0.0)
Chile	Campylobacter spp.	12 (2 - 33)	0.1 (0.0 - 0.1)
Chile	Chlamydia spp.	97 (80 - 119)	0.4 (0.4 - 0.5)
Chile	Citrobacter spp.	105 (72 - 146)	0.4 (0.3 - 0.6)
Chile	Clostridioides difficile	166 (115 - 231)	0.7 (0.5 - 1.0)
Chile	Enterobacter spp.	616 (424 - 853)	2.7 (1.8 - 3.7)
Chile	Enterococcus faecalis	683 (436 - 1,032)	2.9 (1.9 - 4.4)
Chile	Enterococcus faecium	740 (480 - 1,075)	3.1 (2.0 - 4.6)
Chile	Escherichia coli	2,736 (2,012 - 3,642)	11.6 (8.5 - 15.5)
Chile	Group A Streptococcus	456 (266 - 781)	2.0 (1.2 - 3.4)
Chile	Group B Streptococcus	488 (340 - 686)	2.2 (1.5 - 3.1)
Chile	Haemophilus influenzae	124 (100 - 153)	0.6 (0.4 - 0.7)
Chile	Klebsiella pneumoniae	1,691 (1,214 - 2,307)	7.3 (5.2 - 9.9)
Chile	Legionella spp.	162 (136 - 196)	0.7 (0.6 - 0.9)
Chile	Listeria monocytogenes	11 (8 - 17)	0.1 (0.0 - 0.1)
Chile	Morganella spp.	23 (18 - 30)	0.1 (0.1 - 0.1)
Chile	Mycoplasma spp.	125 (104 - 153)	0.6 (0.5 - 0.7)
Chile	Neisseria gonorrhoeae	5 (4 - 6)	0.0 (0.0 - 0.0)
Chile	Neisseria meningitidis	67 (39 - 106)	0.4 (0.2 - 0.5)
Chile	Non-typhoidal Salmonella	31 (17 - 52)	0.1 (0.1 - 0.2)
Chile	Other Klebsiella species	161 (92 - 267)	0.7 (0.4 - 1.1)
Chile	Other enterococci	287 (215 - 377)	1.2 (0.9 - 1.6)
Chile	Proteus spp.	344 (257 - 454)	1.5 (1.1 - 1.9)
Chile	Providencia spp.	12 (9 - 16)	0.1 (0.0 - 0.1)
Chile	Pseudomonas aeruginosa	1,485 (1,053 - 2,039)	6.4 (4.5 - 8.8)
Chile	Salmonella Paratyphi	0 (0 - 0)	0.0 (0.0 - 0.0)
Chile	Salmonella Typhi	14 (8 - 23)	0.1 (0.0 - 0.1)
Chile	Serratia spp.	161 (100 - 241)	0.7 (0.4 - 1.0)
Chile	Shigella spp.	20 (8 - 44)	0.1 (0.0 - 0.2)
Chile	Staphylococcus aureus	3,315 (2,455 - 4,398)	14.3 (10.5 - 18.9)
Chile	Streptococcus pneumoniae	1,114 (899 - 1,394)	4.9 (4.0 - 6.2)
Chile	Vibrio cholerae	0 (0 - 0)	0.0 (0.0 - 0.0)
Uruguay	Acinetobacter baumannii	229 (134 - 364)	4.0 (2.3 - 6.3)

Uruguay	Aeromonas spp.	1 (0 - 1)	0.0 (0.0 - 0.0)
Uruguay	Campylobacter spp.	6 (1 - 16)	0.1 (0.0 - 0.3)
Uruguay	Chlamydia spp.	34 (27 - 42)	0.6 (0.5 - 0.7)
Uruguay	Citrobacter spp.	29 (20 - 40)	0.5 (0.3 - 0.7)
Uruguay	Clostridioides difficile	40 (27 - 57)	0.7 (0.5 - 1.0)
Uruguay	Enterobacter spp.	176 (125 - 240)	3.1 (2.1 - 4.3)
Uruguay	Enterococcus faecalis	174 (111 - 263)	3.0 (1.9 - 4.5)
Uruguay	Enterococcus faecium	183 (120 - 268)	3.2 (2.1 - 4.7)
Uruguay	Escherichia coli	792 (592 - 1,035)	13.0 (9.6 - 17.2)
Uruguay	Group A Streptococcus	151 (92 - 249)	2.6 (1.6 - 4.2)
Uruguay	Group B Streptococcus	151 (107 - 212)	2.7 (1.9 - 3.8)
Uruguay	Haemophilus influenzae	43 (34 - 53)	0.7 (0.6 - 0.9)
Uruguay	Klebsiella pneumoniae	531 (388 - 718)	9.0 (6.5 - 12.3)
Uruguay	Legionella spp.	44 (36 - 54)	0.7 (0.6 - 0.9)
Uruguay	Listeria monocytogenes	2 (1 - 3)	0.0 (0.0 - 0.1)
Uruguay	Morganella spp.	9 (6 - 11)	0.1 (0.1 - 0.2)
Uruguay	Mycoplasma spp.	37 (30 - 45)	0.7 (0.6 - 0.8)
Uruguay	Neisseria gonorrhoeae	2 (2 - 2)	0.0 (0.0 - 0.0)
Uruguay	Neisseria meningitidis	19 (11 - 31)	0.5 (0.3 - 0.8)
Uruguay	Non-typhoidal Salmonella	12 (7 - 20)	0.2 (0.1 - 0.4)
Uruguay	Other Klebsiella species	37 (21 - 63)	0.6 (0.4 - 1.1)
Uruguay	Other enterococci	98 (72 - 129)	1.6 (1.2 - 2.1)
Uruguay	Proteus spp.	110 (82 - 145)	1.8 (1.3 - 2.3)
Uruguay	Providencia spp.	5 (4 - 7)	0.1 (0.1 - 0.1)
Uruguay	Pseudomonas aeruginosa	449 (326 - 611)	7.6 (5.5 - 10.4)
Uruguay	Salmonella Paratyphi	0 (0 - 0)	0.0 (0.0 - 0.0)
Uruguay	Salmonella Typhi	6 (3 - 9)	0.1 (0.1 - 0.2)
Uruguay	Serratia spp.	50 (31 - 75)	0.9 (0.6 - 1.4)
Uruguay	Shigella spp.	3 (1 - 6)	0.1 (0.0 - 0.1)
Uruguay	Staphylococcus aureus	962 (734 - 1,257)	16.1 (12.2 - 21.3)
Uruguay	Streptococcus pneumoniae	375 (305 - 467)	6.6 (5.3 - 8.3)
Uruguay	Vibrio cholerae	0 (0 - 0)	0.0 (0.0 - 0.0)
Andorra	Acinetobacter baumannii	2 (1 - 3)	1.1 (0.6 - 1.9)
Andorra	Aeromonas spp.	0 (0 - 0)	0.0 (0.0 - 0.0)
Andorra	Campylobacter spp.	0 (0 - 0)	0.0 (0.0 - 0.1)
Andorra	Chlamydia spp.	0 (0 - 0)	0.2 (0.2 - 0.3)
Andorra	Citrobacter spp.	0 (0 - 1)	0.3 (0.2 - 0.5)
Andorra	Clostridioides difficile	1 (0 - 1)	0.5 (0.3 - 0.8)
Andorra	Enterobacter spp.	2 (1 - 3)	1.2 (0.8 - 1.9)
Andorra	Enterococcus faecalis	3 (2 - 5)	2.1 (1.3 - 3.5)
Andorra	Enterococcus faecium	3 (2 - 5)	2.3 (1.4 - 3.7)

Andorra	Escherichia coli	16 (10 - 25)	10.8 (6.8 - 16.8)
Andorra	Group A Streptococcus	2 (1 - 3)	1.4 (0.7 - 2.6)
Andorra	Group B Streptococcus	2 (1 - 3)	1.3 (0.8 - 2.0)
Andorra	Haemophilus influenzae	0 (0 - 1)	0.3 (0.2 - 0.4)
Andorra	Klebsiella pneumoniae	6 (4 - 9)	4.2 (2.7 - 6.4)
Andorra	Legionella spp.	1 (1 - 2)	0.8 (0.6 - 1.0)
Andorra	Listeria monocytogenes	0 (0 - 0)	0.0 (0.0 - 0.0)
Andorra	Morganella spp.	0 (0 - 0)	0.0 (0.0 - 0.1)
Andorra	Mycoplasma spp.	1 (1 - 1)	0.5 (0.3 - 0.6)
Andorra	Neisseria gonorrhoeae	0 (0 - 0)	0.0 (0.0 - 0.0)
Andorra	Neisseria meningitidis	0 (0 - 0)	0.2 (0.1 - 0.4)
Andorra	Non-typhoidal Salmonella	0 (0 - 0)	0.1 (0.1 - 0.2)
Andorra	Other Klebsiella species	0 (0 - 1)	0.3 (0.2 - 0.6)
Andorra	Other enterococci	1 (1 - 2)	0.7 (0.4 - 1.1)
Andorra	Proteus spp.	1 (1 - 2)	0.8 (0.5 - 1.3)
Andorra	Providencia spp.	0 (0 - 0)	0.0 (0.0 - 0.0)
Andorra	Pseudomonas aeruginosa	6 (4 - 8)	3.7 (2.4 - 5.6)
Andorra	Salmonella Paratyphi	0 (0 - 0)	0.0 (0.0 - 0.0)
Andorra	Salmonella Typhi	0 (0 - 0)	0.0 (0.0 - 0.1)
Andorra	Serratia spp.	0 (0 - 1)	0.2 (0.1 - 0.4)
Andorra	Shigella spp.	0 (0 - 0)	0.0 (0.0 - 0.0)
Andorra	Staphylococcus aureus	17 (11 - 24)	11.1 (7.4 - 16.4)
Andorra	Streptococcus pneumoniae	5 (4 - 7)	3.4 (2.4 - 4.9)
Andorra	Vibrio cholerae	0 (0 - 0)	0.0 (0.0 - 0.0)
Austria	Acinetobacter baumannii	219 (114 - 378)	1.2 (0.6 - 2.0)
Austria	Aeromonas spp.	0 (0 - 0)	0.0 (0.0 - 0.0)
Austria	Campylobacter spp.	17 (4 - 36)	0.1 (0.0 - 0.2)
Austria	Chlamydia spp.	32 (24 - 43)	0.2 (0.1 - 0.2)
Austria	Citrobacter spp.	49 (27 - 85)	0.3 (0.1 - 0.5)
Austria	Clostridioides difficile	102 (74 - 144)	0.5 (0.4 - 0.7)
Austria	Enterobacter spp.	207 (133 - 306)	1.1 (0.7 - 1.7)
Austria	Enterococcus faecalis	333 (194 - 527)	1.8 (1.1 - 2.9)
Austria	Enterococcus faecium	370 (227 - 558)	2.1 (1.3 - 3.1)
Austria	Escherichia coli	1,672 (1,056 - 2,549)	8.7 (5.5 - 13.1)
Austria	Group A Streptococcus	248 (122 - 459)	1.4 (0.7 - 2.5)
Austria	Group B Streptococcus	207 (127 - 331)	1.2 (0.7 - 1.8)
Austria	Haemophilus influenzae	38 (28 - 51)	0.2 (0.1 - 0.3)
Austria	Klebsiella pneumoniae	660 (431 - 972)	3.5 (2.3 - 5.2)
Austria	Legionella spp.	69 (53 - 88)	0.3 (0.3 - 0.5)
Austria	Listeria monocytogenes	4 (3 - 7)	0.0 (0.0 - 0.1)
Austria	Morganella spp.	5 (3 - 9)	0.0 (0.0 - 0.0)

Austria	Mycoplasma spp.	54 (41 - 71)	0.3 (0.2 - 0.4)
Austria	Neisseria gonorrhoeae	1 (1 - 1)	0.0 (0.0 - 0.0)
Austria	Neisseria meningitidis	28 (15 - 45)	0.2 (0.1 - 0.4)
Austria	Non-typhoidal Salmonella	28 (15 - 46)	0.2 (0.1 - 0.3)
Austria	Other Klebsiella species	56 (32 - 95)	0.3 (0.2 - 0.6)
Austria	Other enterococci	122 (70 - 194)	0.6 (0.4 - 1.0)
Austria	Proteus spp.	140 (91 - 205)	0.7 (0.5 - 1.0)
Austria	Providencia spp.	2 (1 - 4)	0.0 (0.0 - 0.0)
Austria	Pseudomonas aeruginosa	567 (373 - 823)	3.0 (1.9 - 4.4)
Austria	Salmonella Paratyphi	0 (0 - 0)	0.0 (0.0 - 0.0)
Austria	Salmonella Typhi	4 (2 - 10)	0.0 (0.0 - 0.1)
Austria	Serratia spp.	42 (25 - 63)	0.2 (0.1 - 0.4)
Austria	Shigella spp.	1 (1 - 3)	0.0 (0.0 - 0.0)
Austria	Staphylococcus aureus	1,492 (979 - 2,188)	8.0 (5.2 - 11.8)
Austria	Streptococcus pneumoniae	471 (339 - 647)	2.5 (1.8 - 3.6)
Austria	Vibrio cholerae	0 (0 - 0)	0.0 (0.0 - 0.0)
Belgium	Acinetobacter baumannii	296 (167 - 509)	1.2 (0.7 - 2.0)
Belgium	Aeromonas spp.	1 (0 - 2)	0.0 (0.0 - 0.0)
Belgium	Campylobacter spp.	78 (14 - 210)	0.3 (0.0 - 0.7)
Belgium	Chlamydia spp.	104 (86 - 122)	0.4 (0.3 - 0.4)
Belgium	Citrobacter spp.	73 (47 - 110)	0.3 (0.2 - 0.5)
Belgium	Clostridioides difficile	321 (224 - 442)	1.2 (0.9 - 1.6)
Belgium	Enterobacter spp.	385 (284 - 516)	1.5 (1.1 - 2.0)
Belgium	Enterococcus faecalis	591 (378 - 916)	2.4 (1.5 - 3.6)
Belgium	Enterococcus faecium	605 (387 - 888)	2.5 (1.6 - 3.7)
Belgium	Escherichia coli	3,214 (2,201 - 4,516)	12.2 (8.3 - 17.4)
Belgium	Group A Streptococcus	461 (269 - 756)	1.9 (1.1 - 3.1)
Belgium	Group B Streptococcus	392 (275 - 571)	1.6 (1.1 - 2.3)
Belgium	Haemophilus influenzae	130 (109 - 153)	0.5 (0.4 - 0.6)
Belgium	Klebsiella pneumoniae	1,329 (975 - 1,775)	5.1 (3.6 - 6.9)
Belgium	Legionella spp.	314 (264 - 362)	1.1 (0.9 - 1.3)
Belgium	Listeria monocytogenes	6 (4 - 10)	0.0 (0.0 - 0.1)
Belgium	Morganella spp.	14 (9 - 18)	0.0 (0.0 - 0.1)
Belgium	Mycoplasma spp.	178 (151 - 206)	0.7 (0.6 - 0.8)
Belgium	Neisseria gonorrhoeae	3 (2 - 3)	0.0 (0.0 - 0.0)
Belgium	Neisseria meningitidis	33 (18 - 54)	0.2 (0.1 - 0.3)
Belgium	Non-typhoidal Salmonella	50 (19 - 119)	0.2 (0.1 - 0.4)
Belgium	Other Klebsiella species	98 (55 - 168)	0.4 (0.2 - 0.7)
Belgium	Other enterococci	222 (148 - 316)	0.8 (0.5 - 1.2)
Belgium	Proteus spp.	265 (185 - 358)	1.0 (0.7 - 1.3)

Belgium	Providencia spp.	5 (3 - 7)	0.0 (0.0 - 0.0)
Belgium	Pseudomonas aeruginosa	1,226 (937 - 1,607)	4.6 (3.5 - 6.1)
Belgium	Salmonella Paratyphi	0 (0 - 0)	0.0 (0.0 - 0.0)
Belgium	Salmonella Typhi	6 (2 - 15)	0.0 (0.0 - 0.1)
Belgium	Serratia spp.	62 (39 - 90)	0.3 (0.2 - 0.4)
Belgium	Shigella spp.	10 (3 - 23)	0.0 (0.0 - 0.1)
Belgium	Staphylococcus aureus	3,571 (2,783 - 4,578)	13.4 (10.2 - 17.6)
Belgium	Streptococcus pneumoniae	1,239 (1,018 - 1,509)	4.7 (3.8 - 5.8)
Belgium	Vibrio cholerae	0 (0 - 0)	0.0 (0.0 - 0.0)
Cyprus	Acinetobacter baumannii	23 (12 - 41)	1.3 (0.7 - 2.4)
Cyprus	Aeromonas spp.	0 (0 - 0)	0.0 (0.0 - 0.0)
Cyprus	Campylobacter spp.	2 (0 - 5)	0.1 (0.0 - 0.3)
Cyprus	Chlamydia spp.	4 (3 - 5)	0.2 (0.2 - 0.3)
Cyprus	Citrobacter spp.	6 (4 - 9)	0.3 (0.2 - 0.5)
Cyprus	Clostridioides difficile	15 (8 - 26)	0.9 (0.4 - 1.6)
Cyprus	Enterobacter spp.	25 (17 - 35)	1.5 (1.0 - 2.1)
Cyprus	Enterococcus faecalis	45 (29 - 69)	2.7 (1.7 - 4.1)
Cyprus	Enterococcus faecium	45 (29 - 67)	2.6 (1.7 - 3.9)
Cyprus	Escherichia coli	237 (162 - 343)	14.0 (9.6 - 20.2)
Cyprus	Group A Streptococcus	40 (24 - 66)	2.5 (1.5 - 4.0)
Cyprus	Group B Streptococcus	28 (18 - 42)	1.7 (1.1 - 2.5)
Cyprus	Haemophilus influenzae	5 (3 - 6)	0.3 (0.2 - 0.4)
Cyprus	Klebsiella pneumoniae	88 (62 - 125)	5.2 (3.7 - 7.4)
Cyprus	Legionella spp.	10 (8 - 13)	0.7 (0.5 - 0.8)
Cyprus	Listeria monocytogenes	1 (0 - 1)	0.0 (0.0 - 0.1)
Cyprus	Morganella spp.	1 (1 - 2)	0.1 (0.0 - 0.1)
Cyprus	Mycoplasma spp.	6 (5 - 8)	0.4 (0.3 - 0.5)
Cyprus	Neisseria gonorrhoeae	0 (0 - 0)	0.0 (0.0 - 0.0)
Cyprus	Neisseria meningitidis	3 (2 - 5)	0.2 (0.1 - 0.4)
Cyprus	Non-typhoidal Salmonella	3 (2 - 5)	0.2 (0.1 - 0.3)
Cyprus	Other Klebsiella species	7 (4 - 12)	0.4 (0.2 - 0.7)
Cyprus	Other enterococci	19 (12 - 27)	1.2 (0.7 - 1.6)
Cyprus	Proteus spp.	21 (15 - 29)	1.3 (0.9 - 1.7)
Cyprus	Providencia spp.	0 (0 - 1)	0.0 (0.0 - 0.0)
Cyprus	Pseudomonas aeruginosa	72 (51 - 102)	4.3 (3.1 - 6.1)
Cyprus	Salmonella Paratyphi	0 (0 - 0)	0.0 (0.0 - 0.0)
Cyprus	Salmonella Typhi	1 (0 - 1)	0.0 (0.0 - 0.1)
Cyprus	Serratia spp.	5 (3 - 7)	0.3 (0.2 - 0.4)
Cyprus	Shigella spp.	0 (0 - 1)	0.0 (0.0 - 0.0)
Cyprus	Staphylococcus aureus	190 (132 - 271)	11.4 (8.0 - 16.2)

Cyprus	Streptococcus pneumoniae	56 (41 - 77)	3.5 (2.6 - 4.8)
Cyprus	Vibrio cholerae	0 (0 - 0)	0.0 (0.0 - 0.0)
Denmark	Acinetobacter baumannii	143 (80 - 242)	1.2 (0.7 - 2.1)
Denmark	Aeromonas spp.	0 (0 - 1)	0.0 (0.0 - 0.0)
Denmark	Campylobacter spp.	50 (10 - 120)	0.4 (0.1 - 0.9)
Denmark	Chlamydia spp.	44 (36 - 53)	0.3 (0.3 - 0.4)
Denmark	Citrobacter spp.	34 (21 - 54)	0.3 (0.2 - 0.5)
Denmark	Clostridioides difficile	139 (92 - 192)	1.1 (0.8 - 1.5)
Denmark	Enterobacter spp.	172 (122 - 238)	1.4 (1.0 - 2.0)
Denmark	Enterococcus faecalis	262 (161 - 409)	2.3 (1.4 - 3.5)
Denmark	Enterococcus faecium	276 (171 - 413)	2.4 (1.5 - 3.6)
Denmark	Escherichia coli	1,437 (950 - 2,098)	11.8 (7.8 - 17.2)
Denmark	Group A Streptococcus	180 (97 - 309)	1.7 (0.9 - 2.8)
Denmark	Group B Streptococcus	171 (118 - 252)	1.5 (1.0 - 2.2)
Denmark	Haemophilus influenzae	56 (45 - 68)	0.4 (0.4 - 0.5)
Denmark	Klebsiella pneumoniae	596 (423 - 825)	4.9 (3.5 - 6.9)
Denmark	Legionella spp.	125 (103 - 150)	1.0 (0.8 - 1.2)
Denmark	Listeria monocytogenes	4 (3 - 5)	0.0 (0.0 - 0.1)
Denmark	Morganella spp.	6 (4 - 8)	0.0 (0.0 - 0.1)
Denmark	Mycoplasma spp.	73 (60 - 87)	0.6 (0.5 - 0.7)
Denmark	Neisseria gonorrhoeae	2 (1 - 2)	0.0 (0.0 - 0.0)
Denmark	Neisseria meningitidis	18 (10 - 29)	0.2 (0.1 - 0.4)
Denmark	Non-typhoidal Salmonella	22 (9 - 48)	0.2 (0.1 - 0.4)
Denmark	Other Klebsiella species	43 (24 - 72)	0.4 (0.2 - 0.6)
Denmark	Other enterococci	96 (63 - 142)	0.8 (0.5 - 1.2)
Denmark	Proteus spp.	117 (81 - 163)	0.9 (0.6 - 1.3)
Denmark	Providencia spp.	2 (2 - 3)	0.0 (0.0 - 0.0)
Denmark	Pseudomonas aeruginosa	541 (396 - 732)	4.4 (3.2 - 6.1)
Denmark	Salmonella Paratyphi	1 (0 - 1)	0.0 (0.0 - 0.0)
Denmark	Salmonella Typhi	3 (1 - 8)	0.0 (0.0 - 0.1)
Denmark	Serratia spp.	29 (18 - 43)	0.3 (0.2 - 0.4)
Denmark	Shigella spp.	4 (1 - 9)	0.0 (0.0 - 0.1)
Denmark	Staphylococcus aureus	1,559 (1,173 - 2,065)	12.8 (9.4 - 17.2)
Denmark	Streptococcus pneumoniae	540 (430 - 678)	4.4 (3.5 - 5.7)
Denmark	Vibrio cholerae	0 (0 - 0)	0.0 (0.0 - 0.0)
Finland	Acinetobacter baumannii	124 (65 - 219)	1.0 (0.5 - 1.7)
Finland	Aeromonas spp.	0 (0 - 0)	0.0 (0.0 - 0.0)
Finland	Campylobacter spp.	6 (1 - 14)	0.0 (0.0 - 0.1)
Finland	Chlamydia spp.	19 (15 - 26)	0.1 (0.1 - 0.2)
Finland	Citrobacter spp.	30 (18 - 50)	0.3 (0.1 - 0.4)

Finland	Clostridioides difficile	45 (31 - 64)	0.3 (0.2 - 0.5)
Finland	Enterobacter spp.	141 (91 - 205)	1.2 (0.7 - 1.7)
Finland	Enterococcus faecalis	226 (132 - 361)	1.9 (1.1 - 3.0)
Finland	Enterococcus faecium	251 (156 - 375)	2.1 (1.3 - 3.2)
Finland	Escherichia coli	1,109 (710 - 1,661)	8.6 (5.4 - 12.9)
Finland	Group A Streptococcus	142 (73 - 257)	1.3 (0.7 - 2.3)
Finland	Group B Streptococcus	125 (78 - 197)	1.1 (0.6 - 1.7)
Finland	Haemophilus influenzae	25 (18 - 34)	0.2 (0.1 - 0.3)
Finland	Klebsiella pneumoniae	435 (287 - 633)	3.4 (2.2 - 5.1)
Finland	Legionella spp.	45 (35 - 59)	0.3 (0.3 - 0.4)
Finland	Listeria monocytogenes	2 (1 - 4)	0.0 (0.0 - 0.0)
Finland	Morganella spp.	3 (2 - 5)	0.0 (0.0 - 0.0)
Finland	Mycoplasma spp.	32 (25 - 43)	0.3 (0.2 - 0.4)
Finland	Neisseria gonorrhoeae	1 (1 - 2)	0.0 (0.0 - 0.0)
Finland	Neisseria meningitidis	15 (8 - 24)	0.2 (0.1 - 0.3)
Finland	Non-typhoidal Salmonella	13 (7 - 21)	0.1 (0.1 - 0.2)
Finland	Other Klebsiella species	42 (24 - 71)	0.4 (0.2 - 0.6)
Finland	Other enterococci	71 (42 - 112)	0.5 (0.3 - 0.9)
Finland	Proteus spp.	91 (60 - 130)	0.7 (0.4 - 1.0)
Finland	Providencia spp.	1 (1 - 2)	0.0 (0.0 - 0.0)
Finland	Pseudomonas aeruginosa	386 (255 - 555)	3.1 (2.0 - 4.4)
Finland	Salmonella Paratyphi	0 (0 - 0)	0.0 (0.0 - 0.0)
Finland	Salmonella Typhi	2 (1 - 6)	0.0 (0.0 - 0.1)
Finland	Serratia spp.	26 (16 - 39)	0.2 (0.1 - 0.3)
Finland	Shigella spp.	1 (0 - 1)	0.0 (0.0 - 0.0)
Finland	Staphylococcus aureus	980 (651 - 1,424)	7.9 (5.1 - 11.6)
Finland	Streptococcus pneumoniae	287 (207 - 394)	2.3 (1.6 - 3.2)
Finland	Vibrio cholerae	0 (0 - 0)	0.0 (0.0 - 0.0)
France	Acinetobacter baumannii	1,585 (875 - 2,776)	1.0 (0.6 - 1.8)
France	Aeromonas spp.	2 (1 - 3)	0.0 (0.0 - 0.0)
France	Campylobacter spp.	59 (10 - 161)	0.0 (0.0 - 0.1)
France	Chlamydia spp.	399 (323 - 484)	0.2 (0.2 - 0.3)
France	Citrobacter spp.	373 (229 - 610)	0.3 (0.2 - 0.4)
France	Clostridioides difficile	1,193 (839 - 1,655)	0.7 (0.5 - 1.0)
France	Enterobacter spp.	1,898 (1,336 - 2,648)	1.2 (0.8 - 1.7)
France	Enterococcus faecalis	2,978 (1,769 - 4,809)	2.0 (1.2 - 3.1)
France	Enterococcus faecium	3,264 (2,056 - 4,769)	2.3 (1.4 - 3.3)
France	Escherichia coli	15,748 (10,339 - 22,793)	9.9 (6.5 - 14.5)
France	Group A Streptococcus	2,515 (1,474 - 4,221)	1.7 (1.0 - 2.8)
France	Group B Streptococcus	1,962 (1,313 - 2,980)	1.3 (0.9 - 2.0)
France	Haemophilus influenzae	505 (409 - 618)	0.3 (0.2 - 0.4)

France	Klebsiella pneumoniae	6,366 (4,483 - 8,832)	4.0 (2.8 - 5.7)
France	Legionella spp.	1,155 (940 - 1,364)	0.6 (0.5 - 0.8)
France	Listeria monocytogenes	33 (20 - 62)	0.0 (0.0 - 0.1)
France	Morganella spp.	50 (35 - 73)	0.0 (0.0 - 0.0)
France	Mycoplasma spp.	684 (557 - 817)	0.4 (0.3 - 0.5)
France	Neisseria gonorrhoeae	17 (14 - 21)	0.0 (0.0 - 0.0)
France	Neisseria meningitidis	193 (107 - 314)	0.2 (0.1 - 0.3)
France	Non-typhoidal Salmonella	184 (108 - 292)	0.1 (0.1 - 0.2)
France	Other Klebsiella species	532 (293 - 933)	0.4 (0.2 - 0.6)
France	Other enterococci	1,032 (651 - 1,518)	0.6 (0.4 - 1.0)
France	Proteus spp.	1,262 (870 - 1,765)	0.8 (0.5 - 1.1)
France	Providencia spp.	19 (13 - 29)	0.0 (0.0 - 0.0)
France	Pseudomonas aeruginosa	5,781 (4,194 - 7,825)	3.6 (2.5 - 5.0)
France	Salmonella Paratyphi	0 (0 - 0)	0.0 (0.0 - 0.0)
France	Salmonella Typhi	37 (14 - 83)	0.0 (0.0 - 0.1)
France	Serratia spp.	328 (203 - 485)	0.2 (0.1 - 0.3)
France	Shigella spp.	19 (7 - 44)	0.0 (0.0 - 0.0)
France	Staphylococcus aureus	16,237 (11,988 - 21,837)	10.1 (7.2 - 14.0)
France	Streptococcus pneumoniae	5,220 (4,105 - 6,664)	3.2 (2.5 - 4.2)
France	Vibrio cholerae	0 (0 - 0)	0.0 (0.0 - 0.0)
Germany	Acinetobacter baumannii	2,481 (1,367 - 4,210)	1.2 (0.7 - 2.1)
Germany	Aeromonas spp.	6 (3 - 12)	0.0 (0.0 - 0.0)
Germany	Campylobacter spp.	131 (22 - 346)	0.1 (0.0 - 0.1)
Germany	Chlamydia spp.	512 (415 - 636)	0.2 (0.2 - 0.3)
Germany	Citrobacter spp.	604 (373 - 967)	0.3 (0.2 - 0.5)
Germany	Clostridioides difficile	2,713 (1,975 - 3,563)	1.2 (0.9 - 1.5)
Germany	Enterobacter spp.	2,820 (1,945 - 3,982)	1.4 (0.9 - 2.0)
Germany	Enterococcus faecalis	4,596 (2,799 - 7,363)	2.3 (1.4 - 3.7)
Germany	Enterococcus faecium	4,944 (3,166 - 7,276)	2.5 (1.6 - 3.8)
Germany	Escherichia coli	23,667 (15,629 - 34,350)	11.3 (7.4 - 16.6)
Germany	Group A Streptococcus	3,247 (1,759 - 5,655)	1.8 (1.0 - 2.9)
Germany	Group B Streptococcus	2,634 (1,721 - 4,040)	1.4 (0.9 - 2.1)
Germany	Haemophilus influenzae	626 (504 - 793)	0.3 (0.2 - 0.4)
Germany	Klebsiella pneumoniae	9,390 (6,485 - 13,194)	4.6 (3.1 - 6.5)
Germany	Legionella spp.	1,347 (1,124 - 1,632)	0.6 (0.5 - 0.8)
Germany	Listeria monocytogenes	32 (19 - 63)	0.0 (0.0 - 0.0)
Germany	Morganella spp.	84 (60 - 119)	0.0 (0.0 - 0.1)
Germany	Mycoplasma spp.	853 (708 - 1,041)	0.4 (0.4 - 0.5)
Germany	Neisseria gonorrhoeae	14 (12 - 16)	0.0 (0.0 - 0.0)
Germany	Neisseria meningitidis	280 (150 - 456)	0.2 (0.1 - 0.4)

Germany	Non-typhoidal Salmonella	318 (171 - 522)	0.2 (0.1 - 0.3)
Germany	Other Klebsiella species	835 (468 - 1,418)	0.4 (0.3 - 0.7)
Germany	Other enterococci	1,572 (1,011 - 2,358)	0.7 (0.5 - 1.1)
Germany	Proteus spp.	1,969 (1,362 - 2,754)	0.9 (0.6 - 1.3)
Germany	Providencia spp.	34 (23 - 50)	0.0 (0.0 - 0.0)
Germany	Pseudomonas aeruginosa	8,212 (5,741 - 11,296)	4.0 (2.7 - 5.6)
Germany	Salmonella Paratyphi	0 (0 - 0)	0.0 (0.0 - 0.0)
Germany	Salmonella Typhi	54 (20 - 128)	0.0 (0.0 - 0.1)
Germany	Serratia spp.	522 (329 - 772)	0.3 (0.2 - 0.4)
Germany	Shigella spp.	45 (15 - 98)	0.0 (0.0 - 0.0)
Germany	Staphylococcus aureus	21,532 (15,425 - 29,791)	10.6 (7.4 - 14.9)
Germany	Streptococcus pneumoniae	6,832 (5,315 - 8,873)	3.4 (2.6 - 4.5)
Germany	Vibrio cholerae	0 (0 - 0)	0.0 (0.0 - 0.0)
Greece	Acinetobacter baumannii	292 (165 - 494)	1.1 (0.6 - 1.9)
Greece	Aeromonas spp.	0 (0 - 0)	0.0 (0.0 - 0.0)
Greece	Campylobacter spp.	4 (1 - 10)	0.0 (0.0 - 0.0)
Greece	Chlamydia spp.	99 (84 - 117)	0.3 (0.3 - 0.4)
Greece	Citrobacter spp.	59 (35 - 98)	0.2 (0.1 - 0.4)
Greece	Clostridioides difficile	61 (38 - 106)	0.2 (0.1 - 0.4)
Greece	Enterobacter spp.	314 (225 - 438)	1.2 (0.8 - 1.7)
Greece	Enterococcus faecalis	454 (270 - 733)	1.7 (1.0 - 2.8)
Greece	Enterococcus faecium	502 (305 - 760)	2.0 (1.2 - 3.1)
Greece	Escherichia coli	2,750 (1,767 - 4,085)	9.9 (6.2 - 14.8)
Greece	Group A Streptococcus	302 (137 - 582)	1.4 (0.6 - 2.5)
Greece	Group B Streptococcus	330 (225 - 493)	1.3 (0.9 - 2.0)
Greece	Haemophilus influenzae	124 (104 - 147)	0.4 (0.4 - 0.5)
Greece	Klebsiella pneumoniae	1,183 (836 - 1,658)	4.3 (3.0 - 6.1)
Greece	Legionella spp.	284 (240 - 332)	1.0 (0.8 - 1.1)
Greece	Listeria monocytogenes	5 (3 - 10)	0.0 (0.0 - 0.1)
Greece	Morganella spp.	10 (6 - 15)	0.0 (0.0 - 0.0)
Greece	Mycoplasma spp.	163 (139 - 191)	0.6 (0.5 - 0.7)
Greece	Neisseria gonorrhoeae	1 (1 - 1)	0.0 (0.0 - 0.0)
Greece	Neisseria meningitidis	36 (19 - 60)	0.2 (0.1 - 0.4)
Greece	Non-typhoidal Salmonella	32 (17 - 53)	0.1 (0.1 - 0.2)
Greece	Other Klebsiella species	69 (38 - 126)	0.3 (0.2 - 0.5)
Greece	Other enterococci	173 (103 - 273)	0.6 (0.4 - 1.0)
Greece	Proteus spp.	213 (140 - 306)	0.7 (0.5 - 1.1)
Greece	Providencia spp.	4 (3 - 6)	0.0 (0.0 - 0.0)
Greece	Pseudomonas aeruginosa	1,071 (796 - 1,440)	3.8 (2.7 - 5.2)
Greece	Salmonella Paratyphi	0 (0 - 0)	0.0 (0.0 - 0.0)

Greece	Salmonella Typhi	7 (2 - 17)	0.0 (0.0 - 0.1)
Greece	Serratia spp.	54 (32 - 82)	0.2 (0.1 - 0.3)
Greece	Shigella spp.	0 (0 - 1)	0.0 (0.0 - 0.0)
Greece	Staphylococcus aureus	3,288 (2,516 - 4,324)	11.7 (8.6 - 15.8)
Greece	Streptococcus pneumoniae	1,194 (982 - 1,476)	4.3 (3.5 - 5.5)
Greece	Vibrio cholerae	0 (0 - 0)	0.0 (0.0 - 0.0)
Iceland	Acinetobacter baumannii	5 (3 - 8)	0.8 (0.4 - 1.4)
Iceland	Aeromonas spp.	0 (0 - 0)	0.0 (0.0 - 0.0)
Iceland	Campylobacter spp.	1 (0 - 1)	0.1 (0.0 - 0.2)
Iceland	Chlamydia spp.	2 (1 - 2)	0.3 (0.2 - 0.3)
Iceland	Citrobacter spp.	1 (1 - 2)	0.2 (0.1 - 0.3)
Iceland	Clostridioides difficile	3 (2 - 4)	0.5 (0.4 - 0.7)
Iceland	Enterobacter spp.	6 (4 - 8)	0.9 (0.6 - 1.3)
Iceland	Enterococcus faecalis	7 (4 - 12)	1.3 (0.8 - 2.0)
Iceland	Enterococcus faecium	8 (5 - 13)	1.4 (0.9 - 2.2)
Iceland	Escherichia coli	41 (27 - 61)	6.9 (4.4 - 10.2)
Iceland	Group A Streptococcus	5 (3 - 10)	1.0 (0.5 - 1.8)
Iceland	Group B Streptococcus	6 (4 - 9)	1.0 (0.7 - 1.6)
Iceland	Haemophilus influenzae	2 (2 - 3)	0.3 (0.3 - 0.4)
Iceland	Klebsiella pneumoniae	18 (13 - 25)	3.0 (2.0 - 4.1)
Iceland	Legionella spp.	5 (4 - 6)	0.8 (0.6 - 0.9)
Iceland	Listeria monocytogenes	0 (0 - 0)	0.0 (0.0 - 0.0)
Iceland	Morganella spp.	0 (0 - 0)	0.0 (0.0 - 0.0)
Iceland	Mycoplasma spp.	3 (3 - 4)	0.5 (0.4 - 0.6)
Iceland	Neisseria gonorrhoeae	0 (0 - 0)	0.0 (0.0 - 0.0)
Iceland	Neisseria meningitidis	1 (0 - 1)	0.2 (0.1 - 0.3)
Iceland	Non-typhoidal Salmonella	1 (0 - 1)	0.1 (0.0 - 0.2)
Iceland	Other Klebsiella species	1 (1 - 2)	0.2 (0.1 - 0.3)
Iceland	Other enterococci	3 (2 - 4)	0.4 (0.3 - 0.7)
Iceland	Proteus spp.	3 (2 - 5)	0.5 (0.3 - 0.7)
Iceland	Providencia spp.	0 (0 - 0)	0.0 (0.0 - 0.0)
Iceland	Pseudomonas aeruginosa	18 (13 - 24)	2.9 (2.1 - 3.9)
Iceland	Salmonella Paratyphi	0 (0 - 0)	0.0 (0.0 - 0.0)
Iceland	Salmonella Typhi	0 (0 - 0)	0.0 (0.0 - 0.0)
Iceland	Serratia spp.	1 (1 - 1)	0.1 (0.1 - 0.2)
Iceland	Shigella spp.	0 (0 - 0)	0.0 (0.0 - 0.0)
Iceland	Staphylococcus aureus	55 (40 - 73)	8.8 (6.3 - 11.9)
Iceland	Streptococcus pneumoniae	21 (17 - 26)	3.3 (2.6 - 4.2)
Iceland	Vibrio cholerae	0 (0 - 0)	0.0 (0.0 - 0.0)
Ireland	Acinetobacter baumannii	78 (43 - 134)	1.0 (0.6 - 1.8)

Ireland	Aeromonas spp.	0 (0 - 0)	0.0 (0.0 - 0.0)
Ireland	Campylobacter spp.	2 (0 - 6)	0.0 (0.0 - 0.1)
Ireland	Chlamydia spp.	29 (24 - 34)	0.4 (0.3 - 0.4)
Ireland	Citrobacter spp.	17 (11 - 29)	0.2 (0.1 - 0.4)
Ireland	Clostridioides difficile	45 (34 - 61)	0.6 (0.4 - 0.8)
Ireland	Enterobacter spp.	94 (68 - 130)	1.3 (0.9 - 1.7)
Ireland	Enterococcus faecalis	137 (85 - 212)	1.8 (1.1 - 2.8)
Ireland	Enterococcus faecium	145 (90 - 219)	2.0 (1.2 - 3.0)
Ireland	Escherichia coli	746 (501 - 1,080)	9.8 (6.6 - 14.2)
Ireland	Group A Streptococcus	109 (61 - 188)	1.5 (0.9 - 2.6)
Ireland	Group B Streptococcus	103 (71 - 151)	1.4 (1.0 - 2.1)
Ireland	Haemophilus influenzae	36 (30 - 43)	0.5 (0.4 - 0.6)
Ireland	Klebsiella pneumoniae	315 (226 - 430)	4.2 (3.0 - 5.7)
Ireland	Legionella spp.	89 (74 - 104)	1.2 (1.0 - 1.3)
Ireland	Listeria monocytogenes	1 (1 - 3)	0.0 (0.0 - 0.0)
Ireland	Morganella spp.	3 (2 - 4)	0.0 (0.0 - 0.0)
Ireland	Mycoplasma spp.	53 (45 - 63)	0.7 (0.6 - 0.8)
Ireland	Neisseria gonorrhoeae	1 (1 - 1)	0.0 (0.0 - 0.0)
Ireland	Neisseria meningitidis	10 (5 - 17)	0.2 (0.1 - 0.3)
Ireland	Non-typhoidal Salmonella	8 (4 - 13)	0.1 (0.1 - 0.2)
Ireland	Other Klebsiella species	21 (12 - 36)	0.3 (0.2 - 0.5)
Ireland	Other enterococci	51 (33 - 76)	0.7 (0.4 - 1.0)
Ireland	Proteus spp.	59 (41 - 81)	0.8 (0.5 - 1.1)
Ireland	Providencia spp.	1 (1 - 1)	0.0 (0.0 - 0.0)
Ireland	Pseudomonas aeruginosa	306 (230 - 404)	4.0 (3.0 - 5.3)
Ireland	Salmonella Paratyphi	0 (0 - 0)	0.0 (0.0 - 0.0)
Ireland	Salmonella Typhi	2 (1 - 4)	0.0 (0.0 - 0.1)
Ireland	Serratia spp.	15 (9 - 22)	0.2 (0.1 - 0.3)
Ireland	Shigella spp.	1 (0 - 1)	0.0 (0.0 - 0.0)
Ireland	Staphylococcus aureus	943 (718 - 1,240)	12.4 (9.3 - 16.4)
Ireland	Streptococcus pneumoniae	341 (276 - 418)	4.5 (3.6 - 5.6)
Ireland	Vibrio cholerae	0 (0 - 0)	0.0 (0.0 - 0.0)
Israel	Acinetobacter baumannii	158 (84 - 301)	1.3 (0.7 - 2.4)
Israel	Aeromonas spp.	0 (0 - 0)	0.0 (0.0 - 0.0)
Israel	Campylobacter spp.	17 (3 - 48)	0.1 (0.0 - 0.4)
Israel	Chlamydia spp.	32 (27 - 39)	0.3 (0.2 - 0.3)
Israel	Citrobacter spp.	36 (24 - 55)	0.3 (0.2 - 0.5)
Israel	Clostridioides difficile	139 (99 - 193)	1.1 (0.8 - 1.6)
Israel	Enterobacter spp.	170 (123 - 229)	1.4 (1.0 - 1.9)
Israel	Enterococcus faecalis	279 (179 - 418)	2.3 (1.4 - 3.4)
Israel	Enterococcus faecium	259 (164 - 383)	2.1 (1.4 - 3.2)

Israel	Escherichia coli	1,506 (1,032 - 2,103)	12.0 (8.2 - 16.9)
Israel	Group A Streptococcus	392 (219 - 579)	3.2 (1.8 - 4.7)
Israel	Group B Streptococcus	210 (134 - 328)	1.7 (1.1 - 2.6)
Israel	Haemophilus influenzae	42 (34 - 52)	0.3 (0.3 - 0.4)
Israel	Klebsiella pneumoniae	596 (428 - 816)	4.8 (3.4 - 6.6)
Israel	Legionella spp.	80 (67 - 95)	0.6 (0.5 - 0.8)
Israel	Listeria monocytogenes	3 (2 - 5)	0.0 (0.0 - 0.0)
Israel	Morganella spp.	9 (6 - 11)	0.1 (0.0 - 0.1)
Israel	Mycoplasma spp.	52 (43 - 62)	0.4 (0.4 - 0.5)
Israel	Neisseria gonorrhoeae	1 (1 - 1)	0.0 (0.0 - 0.0)
Israel	Neisseria meningitidis	24 (13 - 39)	0.2 (0.1 - 0.4)
Israel	Non-typhoidal Salmonella	21 (10 - 39)	0.2 (0.1 - 0.3)
Israel	Other Klebsiella species	40 (22 - 70)	0.3 (0.2 - 0.6)
Israel	Other enterococci	139 (91 - 190)	1.1 (0.7 - 1.5)
Israel	Proteus spp.	144 (103 - 192)	1.1 (0.8 - 1.5)
Israel	Providencia spp.	4 (2 - 5)	0.0 (0.0 - 0.0)
Israel	Pseudomonas aeruginosa	513 (379 - 692)	4.1 (3.0 - 5.6)
Israel	Salmonella Paratyphi	0 (0 - 0)	0.0 (0.0 - 0.0)
Israel	Salmonella Typhi	5 (2 - 11)	0.0 (0.0 - 0.1)
Israel	Serratia spp.	30 (19 - 45)	0.3 (0.2 - 0.4)
Israel	Shigella spp.	6 (2 - 13)	0.0 (0.0 - 0.1)
Israel	Staphylococcus aureus	1,277 (950 - 1,689)	10.3 (7.6 - 13.8)
Israel	Streptococcus pneumoniae	437 (343 - 560)	3.6 (2.8 - 4.6)
Israel	Vibrio cholerae	0 (0 - 0)	0.0 (0.0 - 0.0)
Italy	Acinetobacter baumannii	1,406 (749 - 2,501)	0.9 (0.5 - 1.6)
Italy	Aeromonas spp.	1 (0 - 1)	0.0 (0.0 - 0.0)
Italy	Campylobacter spp.	75 (15 - 188)	0.0 (0.0 - 0.1)
Italy	Chlamydia spp.	248 (192 - 314)	0.1 (0.1 - 0.2)
Italy	Citrobacter spp.	331 (196 - 550)	0.2 (0.1 - 0.4)
Italy	Clostridioides difficile	796 (587 - 1,087)	0.4 (0.3 - 0.6)
Italy	Enterobacter spp.	1,577 (1,059 - 2,298)	1.0 (0.7 - 1.5)
Italy	Enterococcus faecalis	2,629 (1,507 - 4,410)	1.7 (1.0 - 2.8)
Italy	Enterococcus faecium	3,027 (1,894 - 4,529)	2.0 (1.3 - 3.0)
Italy	Escherichia coli	14,101 (8,865 - 21,321)	8.7 (5.4 - 13.1)
Italy	Group A Streptococcus	2,043 (1,041 - 3,672)	1.5 (0.8 - 2.5)
Italy	Group B Streptococcus	1,610 (1,016 - 2,561)	1.1 (0.7 - 1.7)
Italy	Haemophilus influenzae	320 (246 - 413)	0.2 (0.1 - 0.3)
Italy	Klebsiella pneumoniae	5,345 (3,560 - 7,741)	3.4 (2.2 - 4.9)
Italy	Legionella spp.	772 (616 - 957)	0.4 (0.4 - 0.6)
Italy	Listeria monocytogenes	27 (17 - 48)	0.0 (0.0 - 0.0)
Italy	Morganella spp.	36 (22 - 58)	0.0 (0.0 - 0.0)

Italy	Mycoplasma spp.	437 (347 - 549)	0.3 (0.2 - 0.4)
Italy	Neisseria gonorrhoeae	13 (11 - 15)	0.0 (0.0 - 0.0)
Italy	Neisseria meningitidis	162 (89 - 260)	0.2 (0.1 - 0.3)
Italy	Non-typhoidal Salmonella	182 (84 - 336)	0.1 (0.1 - 0.2)
Italy	Other Klebsiella species	482 (258 - 858)	0.3 (0.2 - 0.5)
Italy	Other enterococci	855 (490 - 1,340)	0.5 (0.3 - 0.8)
Italy	Proteus spp.	1,089 (716 - 1,580)	0.7 (0.4 - 1.0)
Italy	Providencia spp.	14 (8 - 24)	0.0 (0.0 - 0.0)
Italy	Pseudomonas aeruginosa	4,749 (3,224 - 6,740)	2.9 (2.0 - 4.2)
Italy	Salmonella Paratyphi	0 (0 - 0)	0.0 (0.0 - 0.0)
Italy	Salmonella Typhi	27 (10 - 65)	0.0 (0.0 - 0.1)
Italy	Serratia spp.	288 (178 - 432)	0.2 (0.1 - 0.3)
Italy	Shigella spp.	15 (6 - 34)	0.0 (0.0 - 0.0)
Italy	Staphylococcus aureus	13,233 (9,080 - 18,799)	8.3 (5.6 - 11.9)
Italy	Streptococcus pneumoniae	3,677 (2,761 - 4,932)	2.4 (1.7 - 3.2)
Italy	Vibrio cholerae	0 (0 - 0)	0.0 (0.0 - 0.0)
Luxembourg	Acinetobacter baumannii	10 (5 - 17)	1.0 (0.5 - 1.6)
Luxembourg	Aeromonas spp.	0 (0 - 0)	0.0 (0.0 - 0.0)
Luxembourg	Campylobacter spp.	1 (0 - 3)	0.1 (0.0 - 0.3)
Luxembourg	Chlamydia spp.	2 (2 - 3)	0.2 (0.2 - 0.3)
Luxembourg	Citrobacter spp.	2 (1 - 4)	0.2 (0.1 - 0.4)
Luxembourg	Clostridioides difficile	10 (7 - 14)	0.9 (0.7 - 1.3)
Luxembourg	Enterobacter spp.	12 (8 - 17)	1.1 (0.8 - 1.6)
Luxembourg	Enterococcus faecalis	20 (11 - 32)	1.9 (1.1 - 3.0)
Luxembourg	Enterococcus faecium	23 (14 - 34)	2.2 (1.4 - 3.3)
Luxembourg	Escherichia coli	104 (65 - 153)	9.7 (6.1 - 14.3)
Luxembourg	Group A Streptococcus	14 (8 - 25)	1.4 (0.8 - 2.4)
Luxembourg	Group B Streptococcus	12 (8 - 19)	1.2 (0.8 - 1.8)
Luxembourg	Haemophilus influenzae	3 (2 - 4)	0.3 (0.2 - 0.3)
Luxembourg	Klebsiella pneumoniae	41 (28 - 58)	3.8 (2.6 - 5.5)
Luxembourg	Legionella spp.	8 (6 - 10)	0.7 (0.5 - 0.8)
Luxembourg	Listeria monocytogenes	0 (0 - 0)	0.0 (0.0 - 0.0)
Luxembourg	Morganella spp.	0 (0 - 0)	0.0 (0.0 - 0.0)
Luxembourg	Mycoplasma spp.	4 (3 - 5)	0.4 (0.3 - 0.5)
Luxembourg	Neisseria gonorrhoeae	0 (0 - 0)	0.0 (0.0 - 0.0)
Luxembourg	Neisseria meningitidis	1 (1 - 2)	0.2 (0.1 - 0.3)
Luxembourg	Non-typhoidal Salmonella	2 (1 - 3)	0.2 (0.1 - 0.3)
Luxembourg	Other Klebsiella species	4 (2 - 6)	0.4 (0.2 - 0.6)
Luxembourg	Other enterococci	6 (3 - 9)	0.5 (0.3 - 0.9)
Luxembourg	Proteus spp.	8 (5 - 11)	0.7 (0.4 - 1.0)

Luxembourg	Providencia spp.	0 (0 - 0)	0.0 (0.0 - 0.0)
Luxembourg	Pseudomonas aeruginosa	37 (26 - 51)	3.4 (2.4 - 4.7)
Luxembourg	Salmonella Paratyphi	0 (0 - 0)	0.0 (0.0 - 0.0)
Luxembourg	Salmonella Typhi	0 (0 - 1)	0.0 (0.0 - 0.1)
Luxembourg	Serratia spp.	2 (1 - 3)	0.2 (0.1 - 0.3)
Luxembourg	Shigella spp.	0 (0 - 0)	0.0 (0.0 - 0.0)
Luxembourg	Staphylococcus aureus	109 (78 - 149)	10.2 (7.1 - 14.0)
Luxembourg	Streptococcus pneumoniae	33 (25 - 43)	3.1 (2.3 - 4.1)
Luxembourg	Vibrio cholerae	0 (0 - 0)	0.0 (0.0 - 0.0)
Malta	Acinetobacter baumannii	18 (10 - 31)	1.9 (1.0 - 3.2)
Malta	Aeromonas spp.	0 (0 - 0)	0.0 (0.0 - 0.0)
Malta	Campylobacter spp.	0 (0 - 1)	0.0 (0.0 - 0.1)
Malta	Chlamydia spp.	5 (4 - 6)	0.5 (0.4 - 0.7)
Malta	Citrobacter spp.	3 (2 - 6)	0.3 (0.2 - 0.7)
Malta	Clostridioides difficile	3 (2 - 4)	0.3 (0.2 - 0.5)
Malta	Enterobacter spp.	13 (9 - 19)	1.4 (1.0 - 2.1)
Malta	Enterococcus faecalis	16 (10 - 25)	1.7 (1.1 - 2.7)
Malta	Enterococcus faecium	15 (10 - 23)	1.7 (1.0 - 2.5)
Malta	Escherichia coli	76 (52 - 107)	7.9 (5.3 - 11.1)
Malta	Group A Streptococcus	25 (15 - 38)	2.7 (1.6 - 4.1)
Malta	Group B Streptococcus	14 (9 - 22)	1.6 (1.0 - 2.4)
Malta	Haemophilus influenzae	4 (3 - 5)	0.4 (0.3 - 0.5)
Malta	Klebsiella pneumoniae	36 (26 - 49)	3.7 (2.7 - 5.2)
Malta	Legionella spp.	8 (7 - 10)	0.8 (0.7 - 1.0)
Malta	Listeria monocytogenes	0 (0 - 0)	0.0 (0.0 - 0.1)
Malta	Morganella spp.	0 (0 - 1)	0.0 (0.0 - 0.1)
Malta	Mycoplasma spp.	8 (7 - 10)	0.9 (0.7 - 1.1)
Malta	Neisseria gonorrhoeae	0 (0 - 0)	0.0 (0.0 - 0.0)
Malta	Neisseria meningitidis	2 (1 - 3)	0.3 (0.2 - 0.5)
Malta	Non-typhoidal Salmonella	2 (1 - 3)	0.3 (0.1 - 0.4)
Malta	Other Klebsiella species	2 (1 - 4)	0.3 (0.1 - 0.4)
Malta	Other enterococci	9 (6 - 13)	0.9 (0.6 - 1.3)
Malta	Proteus spp.	8 (6 - 11)	0.8 (0.6 - 1.1)
Malta	Providencia spp.	0 (0 - 0)	0.0 (0.0 - 0.0)
Malta	Pseudomonas aeruginosa	35 (26 - 47)	3.6 (2.7 - 4.9)
Malta	Salmonella Paratyphi	0 (0 - 0)	0.0 (0.0 - 0.0)
Malta	Salmonella Typhi	0 (0 - 0)	0.0 (0.0 - 0.1)
Malta	Serratia spp.	2 (1 - 3)	0.2 (0.1 - 0.4)
Malta	Shigella spp.	0 (0 - 0)	0.0 (0.0 - 0.0)
Malta	Staphylococcus aureus	84 (62 - 112)	8.8 (6.4 - 11.9)

Malta	Streptococcus pneumoniae	43 (35 - 53)	4.6 (3.7 - 5.7)
Malta	Vibrio cholerae	0 (0 - 0)	0.0 (0.0 - 0.0)
Monaco	Acinetobacter baumannii	1 (1 - 2)	1.3 (0.7 - 2.2)
Monaco	Aeromonas spp.	0 (0 - 0)	0.0 (0.0 - 0.0)
Monaco	Campylobacter spp.	0 (0 - 0)	0.0 (0.0 - 0.1)
Monaco	Chlamydia spp.	0 (0 - 0)	0.3 (0.3 - 0.4)
Monaco	Citrobacter spp.	0 (0 - 0)	0.3 (0.1 - 0.5)
Monaco	Clostridioides difficile	0 (0 - 1)	0.3 (0.2 - 0.5)
Monaco	Enterobacter spp.	1 (1 - 2)	1.3 (0.9 - 2.0)
Monaco	Enterococcus faecalis	2 (1 - 3)	2.1 (1.2 - 3.5)
Monaco	Enterococcus faecium	2 (1 - 4)	2.6 (1.5 - 4.0)
Monaco	Escherichia coli	12 (7 - 18)	11.4 (6.9 - 17.7)
Monaco	Group A Streptococcus	1 (1 - 2)	1.6 (0.8 - 2.9)
Monaco	Group B Streptococcus	1 (1 - 2)	1.6 (1.0 - 2.4)
Monaco	Haemophilus influenzae	1 (0 - 1)	0.4 (0.3 - 0.6)
Monaco	Klebsiella pneumoniae	5 (3 - 7)	4.7 (3.1 - 6.9)
Monaco	Legionella spp.	1 (1 - 2)	1.1 (0.9 - 1.4)
Monaco	Listeria monocytogenes	0 (0 - 0)	0.0 (0.0 - 0.1)
Monaco	Morganella spp.	0 (0 - 0)	0.0 (0.0 - 0.0)
Monaco	Mycoplasma spp.	1 (1 - 1)	0.7 (0.5 - 0.8)
Monaco	Neisseria gonorrhoeae	0 (0 - 0)	0.0 (0.0 - 0.0)
Monaco	Neisseria meningitidis	0 (0 - 0)	0.3 (0.1 - 0.5)
Monaco	Non-typhoidal Salmonella	0 (0 - 0)	0.2 (0.1 - 0.2)
Monaco	Other Klebsiella species	0 (0 - 1)	0.4 (0.2 - 0.6)
Monaco	Other enterococci	1 (0 - 1)	0.6 (0.3 - 1.1)
Monaco	Proteus spp.	1 (0 - 1)	0.8 (0.5 - 1.2)
Monaco	Providencia spp.	0 (0 - 0)	0.0 (0.0 - 0.0)
Monaco	Pseudomonas aeruginosa	5 (3 - 6)	4.4 (3.1 - 6.3)
Monaco	Salmonella Paratyphi	0 (0 - 0)	0.0 (0.0 - 0.0)
Monaco	Salmonella Typhi	0 (0 - 0)	0.0 (0.0 - 0.1)
Monaco	Serratia spp.	0 (0 - 0)	0.3 (0.2 - 0.4)
Monaco	Shigella spp.	0 (0 - 0)	0.0 (0.0 - 0.0)
Monaco	Staphylococcus aureus	15 (11 - 20)	14.1 (10.1 - 19.9)
Monaco	Streptococcus pneumoniae	5 (4 - 6)	4.7 (3.5 - 6.2)
Monaco	Vibrio cholerae	0 (0 - 0)	0.0 (0.0 - 0.0)
Netherlands	Acinetobacter baumannii	416 (230 - 728)	1.2 (0.7 - 2.1)
Netherlands	Aeromonas spp.	0 (0 - 1)	0.0 (0.0 - 0.0)
Netherlands	Campylobacter spp.	37 (7 - 94)	0.1 (0.0 - 0.2)
Netherlands	Chlamydia spp.	110 (90 - 132)	0.3 (0.2 - 0.4)
Netherlands	Citrobacter spp.	100 (65 - 157)	0.3 (0.2 - 0.5)

Netherlands	Clostridioides difficile	250 (175 - 347)	0.7 (0.5 - 0.9)
Netherlands	Enterobacter spp.	494 (359 - 674)	1.4 (1.0 - 1.9)
Netherlands	Enterococcus faecalis	823 (536 - 1,260)	2.3 (1.5 - 3.5)
Netherlands	Enterococcus faecium	812 (518 - 1,192)	2.3 (1.5 - 3.4)
Netherlands	Escherichia coli	4,450 (3,063 - 6,214)	12.2 (8.3 - 17.1)
Netherlands	Group A Streptococcus	683 (402 - 1,104)	2.1 (1.2 - 3.3)
Netherlands	Group B Streptococcus	555 (380 - 826)	1.6 (1.1 - 2.4)
Netherlands	Haemophilus influenzae	141 (116 - 171)	0.4 (0.3 - 0.5)
Netherlands	Klebsiella pneumoniae	1,698 (1,230 - 2,286)	4.7 (3.4 - 6.4)
Netherlands	Legionella spp.	343 (286 - 408)	0.9 (0.8 - 1.1)
Netherlands	Listeria monocytogenes	11 (8 - 18)	0.0 (0.0 - 0.1)
Netherlands	Morganella spp.	21 (15 - 27)	0.1 (0.0 - 0.1)
Netherlands	Mycoplasma spp.	202 (168 - 241)	0.6 (0.5 - 0.7)
Netherlands	Neisseria gonorrhoeae	2 (2 - 2)	0.0 (0.0 - 0.0)
Netherlands	Neisseria meningitidis	46 (26 - 74)	0.2 (0.1 - 0.3)
Netherlands	Non-typhoidal Salmonella	45 (24 - 76)	0.1 (0.1 - 0.2)
Netherlands	Other Klebsiella species	115 (61 - 202)	0.3 (0.2 - 0.6)
Netherlands	Other enterococci	342 (236 - 478)	0.9 (0.6 - 1.3)
Netherlands	Proteus spp.	379 (270 - 503)	1.0 (0.7 - 1.4)
Netherlands	Providencia spp.	7 (5 - 10)	0.0 (0.0 - 0.0)
Netherlands	Pseudomonas aeruginosa	1,526 (1,140 - 2,035)	4.2 (3.1 - 5.7)
Netherlands	Salmonella Paratyphi	0 (0 - 0)	0.0 (0.0 - 0.0)
Netherlands	Salmonella Typhi	7 (3 - 17)	0.0 (0.0 - 0.1)
Netherlands	Serratia spp.	80 (51 - 118)	0.2 (0.1 - 0.3)
Netherlands	Shigella spp.	5 (2 - 11)	0.0 (0.0 - 0.0)
Netherlands	Staphylococcus aureus	4,401 (3,275 - 5,875)	12.3 (9.0 - 16.5)
Netherlands	Streptococcus pneumoniae	1,431 (1,144 - 1,790)	4.0 (3.2 - 5.1)
Netherlands	Vibrio cholerae	0 (0 - 0)	0.0 (0.0 - 0.0)
Norway	Acinetobacter baumannii	100 (56 - 176)	1.0 (0.5 - 1.7)
Norway	Aeromonas spp.	0 (0 - 1)	0.0 (0.0 - 0.0)
Norway	Campylobacter spp.	31 (6 - 82)	0.3 (0.0 - 0.6)
Norway	Chlamydia spp.	34 (28 - 40)	0.3 (0.2 - 0.3)
Norway	Citrobacter spp.	22 (14 - 34)	0.2 (0.1 - 0.3)
Norway	Clostridioides difficile	127 (95 - 165)	1.2 (0.9 - 1.5)
Norway	Enterobacter spp.	118 (86 - 160)	1.1 (0.8 - 1.5)
Norway	Enterococcus faecalis	177 (115 - 269)	1.7 (1.1 - 2.6)
Norway	Enterococcus faecium	175 (109 - 266)	1.7 (1.1 - 2.7)
Norway	Escherichia coli	1,009 (686 - 1,436)	9.3 (6.3 - 13.5)
Norway	Group A Streptococcus	153 (76 - 246)	1.5 (0.8 - 2.5)
Norway	Group B Streptococcus	132 (90 - 194)	1.3 (0.9 - 1.9)
Norway	Haemophilus influenzae	43 (35 - 51)	0.4 (0.3 - 0.4)

Norway	Klebsiella pneumoniae	403 (297 - 546)	3.7 (2.7 - 5.1)
Norway	Legionella spp.	108 (89 - 126)	0.9 (0.8 - 1.1)
Norway	Listeria monocytogenes	2 (2 - 4)	0.0 (0.0 - 0.0)
Norway	Morganella spp.	5 (3 - 6)	0.0 (0.0 - 0.1)
Norway	Mycoplasma spp.	61 (50 - 71)	0.5 (0.4 - 0.6)
Norway	Neisseria gonorrhoeae	2 (2 - 3)	0.0 (0.0 - 0.0)
Norway	Neisseria meningitidis	11 (6 - 18)	0.2 (0.1 - 0.3)
Norway	Non-typhoidal Salmonella	16 (5 - 42)	0.2 (0.1 - 0.4)
Norway	Other Klebsiella species	24 (13 - 42)	0.2 (0.1 - 0.4)
Norway	Other enterococci	76 (50 - 107)	0.7 (0.4 - 1.0)
Norway	Proteus spp.	83 (58 - 113)	0.8 (0.5 - 1.0)
Norway	Providencia spp.	2 (1 - 2)	0.0 (0.0 - 0.0)
Norway	Pseudomonas aeruginosa	386 (292 - 513)	3.5 (2.6 - 4.7)
Norway	Salmonella Paratyphi	0 (0 - 1)	0.0 (0.0 - 0.0)
Norway	Salmonella Typhi	2 (1 - 4)	0.0 (0.0 - 0.1)
Norway	Serratia spp.	17 (11 - 26)	0.2 (0.1 - 0.3)
Norway	Shigella spp.	4 (1 - 10)	0.0 (0.0 - 0.1)
Norway	Staphylococcus aureus	1,165 (893 - 1,526)	10.7 (7.9 - 14.3)
Norway	Streptococcus pneumoniae	401 (326 - 490)	3.6 (2.9 - 4.5)
Norway	Vibrio cholerae	0 (0 - 0)	0.0 (0.0 - 0.0)
Portugal	Acinetobacter baumannii	327 (190 - 540)	1.3 (0.7 - 2.1)
Portugal	Aeromonas spp.	0 (0 - 0)	0.0 (0.0 - 0.0)
Portugal	Campylobacter spp.	23 (5 - 56)	0.1 (0.0 - 0.2)
Portugal	Chlamydia spp.	134 (113 - 156)	0.5 (0.4 - 0.5)
Portugal	Citrobacter spp.	82 (54 - 123)	0.3 (0.2 - 0.5)
Portugal	Clostridioides difficile	167 (114 - 235)	0.6 (0.4 - 0.9)
Portugal	Enterobacter spp.	427 (320 - 567)	1.6 (1.2 - 2.2)
Portugal	Enterococcus faecalis	636 (416 - 960)	2.5 (1.6 - 3.7)
Portugal	Enterococcus faecium	617 (401 - 897)	2.5 (1.6 - 3.7)
Portugal	Escherichia coli	3,509 (2,475 - 4,885)	12.9 (9.0 - 18.1)
Portugal	Group A Streptococcus	402 (203 - 724)	1.7 (0.9 - 3.0)
Portugal	Group B Streptococcus	414 (299 - 593)	1.6 (1.2 - 2.3)
Portugal	Haemophilus influenzae	165 (141 - 193)	0.6 (0.5 - 0.7)
Portugal	Klebsiella pneumoniae	1,529 (1,151 - 2,016)	5.7 (4.2 - 7.6)
Portugal	Legionella spp.	366 (310 - 419)	1.3 (1.1 - 1.5)
Portugal	Listeria monocytogenes	5 (3 - 9)	0.0 (0.0 - 0.0)
Portugal	Morganella spp.	20 (12 - 25)	0.1 (0.0 - 0.1)
Portugal	Mycoplasma spp.	217 (185 - 249)	0.8 (0.7 - 0.9)
Portugal	Neisseria gonorrhoeae	3 (2 - 3)	0.0 (0.0 - 0.0)
Portugal	Neisseria meningitidis	39 (22 - 64)	0.3 (0.1 - 0.4)

Portugal	Non-typhoidal Salmonella	46 (21 - 86)	0.2 (0.1 - 0.4)
Portugal	Other Klebsiella species	100 (57 - 168)	0.4 (0.2 - 0.7)
Portugal	Other enterococci	256 (174 - 359)	0.9 (0.6 - 1.3)
Portugal	Proteus spp.	305 (220 - 407)	1.1 (0.8 - 1.5)
Portugal	Providencia spp.	8 (5 - 10)	0.0 (0.0 - 0.0)
Portugal	Pseudomonas aeruginosa	1,388 (1,076 - 1,787)	5.1 (3.8 - 6.6)
Portugal	Salmonella Paratyphi	0 (0 - 0)	0.0 (0.0 - 0.0)
Portugal	Salmonella Typhi	9 (3 - 20)	0.0 (0.0 - 0.1)
Portugal	Serratia spp.	68 (44 - 98)	0.3 (0.2 - 0.4)
Portugal	Shigella spp.	3 (1 - 6)	0.0 (0.0 - 0.0)
Portugal	Staphylococcus aureus	4,048 (3,214 - 5,128)	14.7 (11.4 - 19.0)
Portugal	Streptococcus pneumoniae	1,525 (1,273 - 1,836)	5.6 (4.6 - 6.9)
Portugal	Vibrio cholerae	0 (0 - 0)	0.0 (0.0 - 0.0)
San Marino	Acinetobacter baumannii	1 (0 - 2)	1.4 (0.7 - 2.6)
San Marino	Aeromonas spp.	0 (0 - 0)	0.0 (0.0 - 0.0)
San Marino	Campylobacter spp.	0 (0 - 0)	0.0 (0.0 - 0.1)
San Marino	Chlamydia spp.	0 (0 - 0)	0.2 (0.1 - 0.3)
San Marino	Citrobacter spp.	0 (0 - 0)	0.3 (0.1 - 0.6)
San Marino	Clostridioides difficile	0 (0 - 0)	0.3 (0.2 - 0.6)
San Marino	Enterobacter spp.	1 (0 - 1)	1.3 (0.7 - 2.1)
San Marino	Enterococcus faecalis	1 (1 - 2)	1.9 (1.0 - 3.4)
San Marino	Enterococcus faecium	1 (1 - 2)	2.2 (1.1 - 3.7)
San Marino	Escherichia coli	7 (4 - 11)	9.2 (5.0 - 15.4)
San Marino	Group A Streptococcus	1 (0 - 2)	1.4 (0.6 - 2.7)
San Marino	Group B Streptococcus	1 (0 - 1)	1.3 (0.7 - 2.2)
San Marino	Haemophilus influenzae	0 (0 - 0)	0.3 (0.2 - 0.4)
San Marino	Klebsiella pneumoniae	3 (2 - 4)	3.8 (2.1 - 6.3)
San Marino	Legionella spp.	0 (0 - 1)	0.5 (0.3 - 0.7)
San Marino	Listeria monocytogenes	0 (0 - 0)	0.0 (0.0 - 0.1)
San Marino	Morganella spp.	0 (0 - 0)	0.0 (0.0 - 0.0)
San Marino	Mycoplasma spp.	0 (0 - 0)	0.4 (0.3 - 0.7)
San Marino	Neisseria gonorrhoeae	0 (0 - 0)	0.0 (0.0 - 0.0)
San Marino	Neisseria meningitidis	0 (0 - 0)	0.3 (0.1 - 0.5)
San Marino	Non-typhoidal Salmonella	0 (0 - 0)	0.2 (0.1 - 0.3)
San Marino	Other Klebsiella species	0 (0 - 0)	0.3 (0.1 - 0.6)
San Marino	Other enterococci	0 (0 - 1)	0.7 (0.3 - 1.2)
San Marino	Proteus spp.	1 (0 - 1)	0.7 (0.4 - 1.2)
San Marino	Providencia spp.	0 (0 - 0)	0.0 (0.0 - 0.0)
San Marino	Pseudomonas aeruginosa	2 (1 - 4)	3.3 (1.9 - 5.4)
San Marino	Salmonella Paratyphi	0 (0 - 0)	0.0 (0.0 - 0.0)

San Marino	Salmonella Typhi	0 (0 - 0)	0.0 (0.0 - 0.1)
San Marino	Serratia spp.	0 (0 - 0)	0.3 (0.1 - 0.4)
San Marino	Shigella spp.	0 (0 - 0)	0.0 (0.0 - 0.0)
San Marino	Staphylococcus aureus	7 (4 - 10)	9.3 (5.3 - 15.3)
San Marino	Streptococcus pneumoniae	2 (1 - 3)	3.3 (2.0 - 5.0)
San Marino	Vibrio cholerae	0 (0 - 0)	0.0 (0.0 - 0.0)
Spain	Acinetobacter baumannii	1,117 (610 - 1,945)	1.0 (0.6 - 1.8)
Spain	Aeromonas spp.	1 (0 - 1)	0.0 (0.0 - 0.0)
Spain	Campylobacter spp.	71 (13 - 184)	0.1 (0.0 - 0.1)
Spain	Chlamydia spp.	270 (216 - 330)	0.2 (0.2 - 0.3)
Spain	Citrobacter spp.	309 (200 - 474)	0.3 (0.2 - 0.5)
Spain	Clostridioides difficile	823 (575 - 1,124)	0.7 (0.5 - 0.9)
Spain	Enterobacter spp.	1,416 (1,009 - 1,965)	1.3 (0.9 - 1.8)
Spain	Enterococcus faecalis	2,431 (1,569 - 3,781)	2.2 (1.4 - 3.4)
Spain	Enterococcus faecium	2,409 (1,566 - 3,467)	2.3 (1.5 - 3.4)
Spain	Escherichia coli	12,384 (8,646 - 17,224)	10.9 (7.5 - 15.3)
Spain	Group A Streptococcus	1,691 (984 - 2,809)	1.7 (1.0 - 2.7)
Spain	Group B Streptococcus	1,398 (955 - 2,091)	1.3 (0.9 - 2.0)
Spain	Haemophilus influenzae	332 (266 - 411)	0.3 (0.2 - 0.4)
Spain	Klebsiella pneumoniae	4,725 (3,392 - 6,429)	4.2 (3.0 - 5.8)
Spain	Legionella spp.	763 (619 - 919)	0.6 (0.5 - 0.8)
Spain	Listeria monocytogenes	20 (13 - 33)	0.0 (0.0 - 0.0)
Spain	Morganella spp.	60 (44 - 78)	0.0 (0.0 - 0.1)
Spain	Mycoplasma spp.	485 (395 - 589)	0.4 (0.4 - 0.5)
Spain	Neisseria gonorrhoeae	10 (8 - 11)	0.0 (0.0 - 0.0)
Spain	Neisseria meningitidis	130 (73 - 209)	0.2 (0.1 - 0.3)
Spain	Non-typhoidal Salmonella	132 (69 - 229)	0.1 (0.1 - 0.2)
Spain	Other Klebsiella species	395 (214 - 695)	0.4 (0.2 - 0.6)
Spain	Other enterococci	925 (635 - 1,299)	0.8 (0.5 - 1.1)
Spain	Proteus spp.	1,084 (793 - 1,435)	0.9 (0.7 - 1.2)
Spain	Providencia spp.	22 (15 - 29)	0.0 (0.0 - 0.0)
Spain	Pseudomonas aeruginosa	4,141 (3,036 - 5,587)	3.7 (2.6 - 5.0)
Spain	Salmonella Paratyphi	0 (0 - 0)	0.0 (0.0 - 0.0)
Spain	Salmonella Typhi	22 (9 - 50)	0.0 (0.0 - 0.1)
Spain	Serratia spp.	242 (155 - 351)	0.2 (0.2 - 0.3)
Spain	Shigella spp.	5 (2 - 13)	0.0 (0.0 - 0.0)
Spain	Staphylococcus aureus	11,125 (8,061 - 15,194)	10.0 (7.1 - 14.0)
Spain	Streptococcus pneumoniae	3,544 (2,759 - 4,566)	3.2 (2.5 - 4.2)
Spain	Vibrio cholerae	0 (0 - 0)	0.0 (0.0 - 0.0)
Sweden	Acinetobacter baumannii	213 (115 - 382)	0.9 (0.5 - 1.6)

Sweden	Aeromonas spp.	1 (1 - 2)	0.0 (0.0 - 0.0)
Sweden	Campylobacter spp.	118 (23 - 284)	0.4 (0.1 - 1.0)
Sweden	Chlamydia spp.	51 (41 - 62)	0.2 (0.2 - 0.2)
Sweden	Citrobacter spp.	44 (27 - 72)	0.2 (0.1 - 0.3)
Sweden	Clostridioides difficile	278 (196 - 379)	1.1 (0.8 - 1.5)
Sweden	Enterobacter spp.	229 (162 - 322)	1.0 (0.7 - 1.4)
Sweden	Enterococcus faecalis	359 (219 - 566)	1.6 (1.0 - 2.5)
Sweden	Enterococcus faecium	382 (238 - 580)	1.8 (1.1 - 2.7)
Sweden	Escherichia coli	2,040 (1,332 - 3,014)	8.6 (5.6 - 12.8)
Sweden	Group A Streptococcus	340 (193 - 563)	1.6 (0.9 - 2.5)
Sweden	Group B Streptococcus	258 (168 - 391)	1.2 (0.8 - 1.8)
Sweden	Haemophilus influenzae	65 (52 - 81)	0.3 (0.2 - 0.3)
Sweden	Klebsiella pneumoniae	786 (546 - 1,109)	3.4 (2.3 - 4.8)
Sweden	Legionella spp.	158 (131 - 189)	0.6 (0.5 - 0.8)
Sweden	Listeria monocytogenes	4 (3 - 7)	0.0 (0.0 - 0.0)
Sweden	Morganella spp.	8 (6 - 11)	0.0 (0.0 - 0.0)
Sweden	Mycoplasma spp.	89 (73 - 108)	0.4 (0.3 - 0.5)
Sweden	Neisseria gonorrhoeae	2 (2 - 2)	0.0 (0.0 - 0.0)
Sweden	Neisseria meningitidis	22 (12 - 36)	0.2 (0.1 - 0.3)
Sweden	Non-typhoidal Salmonella	47 (11 - 129)	0.2 (0.1 - 0.5)
Sweden	Other Klebsiella species	54 (29 - 94)	0.2 (0.1 - 0.4)
Sweden	Other enterococci	144 (94 - 208)	0.6 (0.4 - 0.9)
Sweden	Proteus spp.	163 (113 - 229)	0.7 (0.5 - 0.9)
Sweden	Providencia spp.	3 (2 - 4)	0.0 (0.0 - 0.0)
Sweden	Pseudomonas aeruginosa	728 (523 - 1,005)	3.1 (2.2 - 4.3)
Sweden	Salmonella Paratyphi	0 (0 - 0)	0.0 (0.0 - 0.0)
Sweden	Salmonella Typhi	4 (1 - 9)	0.0 (0.0 - 0.1)
Sweden	Serratia spp.	38 (23 - 57)	0.2 (0.1 - 0.3)
Sweden	Shigella spp.	17 (6 - 40)	0.1 (0.0 - 0.1)
Sweden	Staphylococcus aureus	2,100 (1,522 - 2,854)	8.9 (6.3 - 12.3)
Sweden	Streptococcus pneumoniae	658 (515 - 840)	2.8 (2.2 - 3.7)
Sweden	Vibrio cholerae	0 (0 - 0)	0.0 (0.0 - 0.0)
Switzerland	Acinetobacter baumannii	180 (101 - 308)	0.9 (0.5 - 1.6)
Switzerland	Aeromonas spp.	0 (0 - 1)	0.0 (0.0 - 0.0)
Switzerland	Campylobacter spp.	11 (2 - 32)	0.1 (0.0 - 0.1)
Switzerland	Chlamydia spp.	37 (30 - 46)	0.2 (0.1 - 0.2)
Switzerland	Citrobacter spp.	39 (24 - 64)	0.2 (0.1 - 0.4)
Switzerland	Clostridioides difficile	152 (108 - 210)	0.7 (0.5 - 1.0)
Switzerland	Enterobacter spp.	189 (132 - 266)	1.0 (0.7 - 1.4)
Switzerland	Enterococcus faecalis	320 (198 - 496)	1.7 (1.1 - 2.6)
Switzerland	Enterococcus faecium	345 (219 - 502)	1.8 (1.2 - 2.7)

Switzerland	Escherichia coli	1,766 (1,167 - 2,519)	8.8 (5.8 - 12.5)
Switzerland	Group A Streptococcus	236 (133 - 403)	1.3 (0.7 - 2.2)
Switzerland	Group B Streptococcus	206 (138 - 309)	1.1 (0.7 - 1.6)
Switzerland	Haemophilus influenzae	46 (36 - 57)	0.2 (0.2 - 0.3)
Switzerland	Klebsiella pneumoniae	661 (460 - 904)	3.3 (2.3 - 4.7)
Switzerland	Legionella spp.	110 (89 - 133)	0.5 (0.4 - 0.6)
Switzerland	Listeria monocytogenes	4 (2 - 6)	0.0 (0.0 - 0.0)
Switzerland	Morganella spp.	6 (5 - 9)	0.0 (0.0 - 0.0)
Switzerland	Mycoplasma spp.	65 (53 - 80)	0.3 (0.3 - 0.4)
Switzerland	Neisseria gonorrhoeae	2 (1 - 2)	0.0 (0.0 - 0.0)
Switzerland	Neisseria meningitidis	19 (11 - 31)	0.2 (0.1 - 0.2)
Switzerland	Non-typhoidal Salmonella	30 (11 - 66)	0.2 (0.1 - 0.3)
Switzerland	Other Klebsiella species	49 (27 - 86)	0.3 (0.1 - 0.4)
Switzerland	Other enterococci	120 (76 - 176)	0.6 (0.4 - 0.9)
Switzerland	Proteus spp.	141 (98 - 194)	0.7 (0.5 - 1.0)
Switzerland	Providencia spp.	2 (2 - 3)	0.0 (0.0 - 0.0)
Switzerland	Pseudomonas aeruginosa	579 (416 - 799)	2.9 (2.1 - 4.0)
Switzerland	Salmonella Paratyphi	0 (0 - 0)	0.0 (0.0 - 0.0)
Switzerland	Salmonella Typhi	7 (1 - 12)	0.0 (0.0 - 0.1)
Switzerland	Serratia spp.	34 (21 - 50)	0.2 (0.1 - 0.3)
Switzerland	Shigella spp.	4 (1 - 8)	0.0 (0.0 - 0.0)
Switzerland	Staphylococcus aureus	1,678 (1,200 - 2,284)	8.5 (5.9 - 11.7)
Switzerland	Streptococcus pneumoniae	508 (393 - 652)	2.6 (1.9 - 3.4)
Switzerland	Vibrio cholerae	0 (0 - 0)	0.0 (0.0 - 0.0)
United Kingdom	Acinetobacter baumannii	1,692 (973 - 2,844)	1.3 (0.7 - 2.2)
United Kingdom	Aeromonas spp.	1 (1 - 2)	0.0 (0.0 - 0.0)
United Kingdom	Campylobacter spp.	188 (38 - 446)	0.1 (0.0 - 0.3)
United Kingdom	Chlamydia spp.	700 (597 - 806)	0.5 (0.4 - 0.6)
United Kingdom	Citrobacter spp.	429 (284 - 648)	0.3 (0.2 - 0.5)
United Kingdom	Clostridioides difficile	840 (627 - 1,109)	0.6 (0.5 - 0.8)
United Kingdom	Enterobacter spp.	2,332 (1,751 - 3,066)	1.7 (1.3 - 2.3)
United Kingdom	Enterococcus faecalis	3,356 (2,174 - 5,165)	2.6 (1.7 - 4.0)
United Kingdom	Enterococcus faecium	3,270 (2,105 - 4,771)	2.6 (1.6 - 3.8)
United Kingdom	Escherichia coli	17,618 (12,246 - 24,535)	12.9 (8.9 - 18.1)
United Kingdom	Group A Streptococcus	2,357 (1,397 - 3,881)	1.9 (1.2 - 3.1)
United Kingdom	Group B Streptococcus	2,198 (1,598 - 3,088)	1.7 (1.2 - 2.4)
United Kingdom	Haemophilus influenzae	870 (746 - 1,014)	0.6 (0.5 - 0.7)
United Kingdom	Klebsiella pneumoniae	7,747 (5,771 - 10,261)	5.7 (4.2 - 7.7)
United Kingdom	Legionella spp.	1,938 (1,661 - 2,207)	1.4 (1.2 - 1.6)
United Kingdom	Listeria monocytogenes	30 (20 - 50)	0.0 (0.0 - 0.1)
United Kingdom	Morganella spp.	89 (67 - 111)	0.1 (0.0 - 0.1)

United Kingdom	Mycoplasma spp.	1,175 (1,019 - 1,353)	0.9 (0.8 - 1.0)
United Kingdom	Neisseria gonorrhoeae	31 (27 - 33)	0.0 (0.0 - 0.0)
United Kingdom	Neisseria meningitidis	206 (115 - 334)	0.2 (0.1 - 0.4)
United Kingdom	Non-typhoidal Salmonella	212 (99 - 389)	0.2 (0.1 - 0.3)
United Kingdom	Other Klebsiella species	573 (329 - 971)	0.5 (0.3 - 0.8)
United Kingdom	Other enterococci	1,252 (881 - 1,719)	0.9 (0.6 - 1.3)
United Kingdom	Proteus spp.	1,510 (1,115 - 1,994)	1.1 (0.8 - 1.4)
United Kingdom	Providencia spp.	34 (25 - 44)	0.0 (0.0 - 0.0)
United Kingdom	Pseudomonas aeruginosa	7,359 (5,705 - 9,499)	5.4 (4.1 - 7.1)
United Kingdom	Salmonella Paratyphi	0 (0 - 0)	0.0 (0.0 - 0.0)
United Kingdom	Salmonella Typhi	37 (13 - 88)	0.0 (0.0 - 0.1)
United Kingdom	Serratia spp.	344 (221 - 505)	0.3 (0.2 - 0.4)
United Kingdom	Shigella spp.	16 (6 - 37)	0.0 (0.0 - 0.0)
United Kingdom	Staphylococcus aureus	20,900 (16,588 - 26,528)	15.2 (11.8 - 19.6)
United Kingdom	Streptococcus pneumoniae	7,852 (6,645 - 9,370)	5.7 (4.8 - 7.0)
United Kingdom	Vibrio cholerae	0 (0 - 0)	0.0 (0.0 - 0.0)
Bolivia (Plurinational State of)	Acinetobacter baumannii	817 (461 - 1,298)	10.3 (5.8 - 16.3)
Bolivia (Plurinational State of)	Aeromonas spp.	7 (3 - 17)	0.1 (0.0 - 0.1)
Bolivia (Plurinational State of)	Campylobacter spp.	70 (20 - 169)	0.8 (0.2 - 1.9)
Bolivia (Plurinational State of)	Chlamydia spp.	160 (118 - 210)	1.8 (1.3 - 2.3)
Bolivia (Plurinational State of)	Citrobacter spp.	84 (51 - 127)	0.9 (0.6 - 1.4)
Bolivia (Plurinational State of)	Clostridioides difficile	27 (12 - 55)	0.2 (0.1 - 0.5)
Bolivia (Plurinational State of)	Enterobacter spp.	497 (327 - 707)	5.7 (3.7 - 8.2)
Bolivia (Plurinational State of)	Enterococcus faecalis	437 (261 - 704)	5.0 (2.8 - 8.1)
Bolivia (Plurinational State of)	Enterococcus faecium	436 (258 - 667)	5.1 (3.0 - 7.9)
Bolivia (Plurinational State of)	Escherichia coli	1,796 (1,224 - 2,495)	21.6 (14.8 - 29.7)
Bolivia (Plurinational State of)	Group A Streptococcus	296 (166 - 520)	3.2 (1.6 - 6.0)
Bolivia (Plurinational State of)	Group B Streptococcus	524 (378 - 709)	5.5 (4.0 - 7.4)
Bolivia (Plurinational State of)	Haemophilus influenzae	227 (172 - 290)	2.4 (1.8 - 3.0)
Bolivia (Plurinational State of)	Klebsiella pneumoniae	1,975 (1,388 - 2,690)	22.6 (15.8 - 30.5)
Bolivia (Plurinational State of)	Legionella spp.	105 (71 - 151)	1.2 (0.8 - 1.6)
Bolivia (Plurinational State of)	Listeria monocytogenes	11 (7 - 18)	0.1 (0.1 - 0.2)
Bolivia (Plurinational State of)	Morganella spp.	12 (7 - 19)	0.2 (0.1 - 0.3)
Bolivia (Plurinational State of)	Mycoplasma spp.	146 (110 - 185)	1.4 (1.1 - 1.8)
Bolivia (Plurinational State of)	Neisseria gonorrhoeae	5 (3 - 7)	0.1 (0.0 - 0.1)
Bolivia (Plurinational State of)	Neisseria meningitidis	241 (153 - 366)	1.9 (1.2 - 2.9)
Bolivia (Plurinational State of)	Non-typhoidal Salmonella	131 (86 - 195)	1.2 (0.8 - 1.9)
Bolivia (Plurinational State of)	Other Klebsiella species	130 (63 - 228)	1.6 (0.7 - 2.8)
Bolivia (Plurinational State of)	Other enterococci	161 (105 - 234)	1.9 (1.3 - 2.9)
Bolivia (Plurinational State of)	Proteus spp.	231 (148 - 346)	2.9 (1.9 - 4.4)

Bolivia (Plurinational State of)	Providencia spp.	13 (7 - 23)	0.2 (0.1 - 0.3)
Bolivia (Plurinational State of)	Pseudomonas aeruginosa	1,275 (885 - 1,761)	14.9 (10.4 - 20.6)
Bolivia (Plurinational State of)	Salmonella Paratyphi	0 (0 - 0)	0.0 (0.0 - 0.0)
Bolivia (Plurinational State of)	Salmonella Typhi	153 (95 - 229)	1.3 (0.8 - 1.9)
Bolivia (Plurinational State of)	Serratia spp.	201 (123 - 303)	2.1 (1.3 - 3.3)
Bolivia (Plurinational State of)	Shigella spp.	37 (12 - 87)	0.3 (0.1 - 0.7)
Bolivia (Plurinational State of)	Staphylococcus aureus	2,629 (1,955 - 3,444)	32.0 (23.9 - 41.8)
Bolivia (Plurinational State of)	Streptococcus pneumoniae	1,740 (1,345 - 2,211)	18.5 (14.5 - 23.4)
Bolivia (Plurinational State of)	Vibrio cholerae	0 (0 - 0)	0.0 (0.0 - 0.0)
Ecuador	Acinetobacter baumannii	709 (410 - 1,144)	5.1 (3.0 - 8.3)
Ecuador	Aeromonas spp.	4 (2 - 8)	0.0 (0.0 - 0.1)
Ecuador	Campylobacter spp.	26 (6 - 72)	0.2 (0.0 - 0.5)
Ecuador	Chlamydia spp.	132 (102 - 175)	1.0 (0.8 - 1.3)
Ecuador	Citrobacter spp.	92 (56 - 140)	0.6 (0.4 - 1.0)
Ecuador	Clostridioides difficile	50 (25 - 99)	0.3 (0.1 - 0.6)
Ecuador	Enterobacter spp.	564 (375 - 829)	4.0 (2.7 - 5.8)
Ecuador	Enterococcus faecalis	495 (293 - 791)	3.5 (2.0 - 5.6)
Ecuador	Enterococcus faecium	537 (328 - 825)	3.8 (2.3 - 5.8)
Ecuador	Escherichia coli	1,912 (1,263 - 2,771)	13.9 (9.2 - 20.0)
Ecuador	Group A Streptococcus	347 (180 - 653)	2.4 (1.2 - 4.6)
Ecuador	Group B Streptococcus	491 (340 - 698)	3.5 (2.4 - 4.9)
Ecuador	Haemophilus influenzae	176 (137 - 228)	1.3 (1.0 - 1.6)
Ecuador	Klebsiella pneumoniae	1,731 (1,172 - 2,478)	12.5 (8.5 - 17.9)
Ecuador	Legionella spp.	106 (80 - 143)	0.8 (0.6 - 1.0)
Ecuador	Listeria monocytogenes	11 (7 - 20)	0.1 (0.0 - 0.1)
Ecuador	Morganella spp.	11 (6 - 18)	0.1 (0.0 - 0.1)
Ecuador	Mycoplasma spp.	151 (118 - 193)	1.0 (0.8 - 1.3)
Ecuador	Neisseria gonorrhoeae	3 (2 - 4)	0.0 (0.0 - 0.0)
Ecuador	Neisseria meningitidis	177 (104 - 277)	1.1 (0.6 - 1.7)
Ecuador	Non-typhoidal Salmonella	89 (55 - 133)	0.6 (0.4 - 0.9)
Ecuador	Other Klebsiella species	146 (76 - 257)	1.0 (0.5 - 1.8)
Ecuador	Other enterococci	177 (111 - 268)	1.3 (0.8 - 1.9)
Ecuador	Proteus spp.	248 (157 - 377)	1.8 (1.2 - 2.8)
Ecuador	Providencia spp.	9 (5 - 16)	0.1 (0.0 - 0.1)
Ecuador	Pseudomonas aeruginosa	1,299 (878 - 1,869)	9.4 (6.4 - 13.5)
Ecuador	Salmonella Paratyphi	0 (0 - 0)	0.0 (0.0 - 0.0)
Ecuador	Salmonella Typhi	56 (33 - 88)	0.4 (0.2 - 0.6)
Ecuador	Serratia spp.	189 (112 - 296)	1.3 (0.8 - 2.1)
Ecuador	Shigella spp.	20 (7 - 45)	0.1 (0.0 - 0.3)
Ecuador	Staphylococcus aureus	2,596 (1,867 - 3,602)	19.1 (13.9 - 26.2)

Ecuador	Streptococcus pneumoniae	1,521 (1,174 - 1,985)	10.8 (8.4 - 14.0)
Ecuador	Vibrio cholerae	0 (0 - 0)	0.0 (0.0 - 0.0)
Peru	Acinetobacter baumannii	1,139 (661 - 1,800)	3.5 (2.0 - 5.5)
Peru	Aeromonas spp.	11 (4 - 21)	0.0 (0.0 - 0.1)
Peru	Campylobacter spp.	229 (75 - 512)	0.7 (0.2 - 1.6)
Peru	Chlamydia spp.	350 (260 - 468)	1.1 (0.8 - 1.4)
Peru	Citrobacter spp.	155 (95 - 237)	0.5 (0.3 - 0.7)
Peru	Clostridioides difficile	104 (46 - 207)	0.3 (0.1 - 0.6)
Peru	Enterobacter spp.	1,050 (679 - 1,553)	3.2 (2.1 - 4.8)
Peru	Enterococcus faecalis	868 (502 - 1,407)	2.7 (1.6 - 4.4)
Peru	Enterococcus faecium	893 (526 - 1,381)	2.8 (1.6 - 4.3)
Peru	Escherichia coli	3,694 (2,474 - 5,285)	11.4 (7.6 - 16.3)
Peru	Group A Streptococcus	544 (278 - 989)	1.7 (0.9 - 3.1)
Peru	Group B Streptococcus	998 (679 - 1,398)	3.1 (2.1 - 4.3)
Peru	Haemophilus influenzae	438 (321 - 586)	1.3 (1.0 - 1.8)
Peru	Klebsiella pneumoniae	3,311 (2,274 - 4,697)	10.2 (7.0 - 14.5)
Peru	Legionella spp.	342 (250 - 456)	1.0 (0.8 - 1.4)
Peru	Listeria monocytogenes	13 (7 - 24)	0.0 (0.0 - 0.1)
Peru	Morganella spp.	26 (17 - 37)	0.1 (0.1 - 0.1)
Peru	Mycoplasma spp.	408 (302 - 549)	1.2 (0.9 - 1.7)
Peru	Neisseria gonorrhoeae	9 (6 - 13)	0.0 (0.0 - 0.0)
Peru	Neisseria meningitidis	254 (145 - 398)	0.8 (0.4 - 1.2)
Peru	Non-typhoidal Salmonella	134 (82 - 209)	0.4 (0.3 - 0.7)
Peru	Other Klebsiella species	234 (120 - 413)	0.7 (0.4 - 1.3)
Peru	Other enterococci	343 (222 - 501)	1.1 (0.7 - 1.5)
Peru	Proteus spp.	433 (280 - 628)	1.3 (0.9 - 1.9)
Peru	Providencia spp.	19 (12 - 28)	0.1 (0.0 - 0.1)
Peru	Pseudomonas aeruginosa	2,634 (1,798 - 3,692)	8.1 (5.5 - 11.4)
Peru	Salmonella Paratyphi	0 (0 - 0)	0.0 (0.0 - 0.0)
Peru	Salmonella Typhi	78 (46 - 124)	0.2 (0.1 - 0.4)
Peru	Serratia spp.	296 (170 - 463)	0.9 (0.5 - 1.4)
Peru	Shigella spp.	55 (20 - 123)	0.2 (0.1 - 0.4)
Peru	Staphylococcus aureus	6,221 (4,496 - 8,536)	19.0 (13.7 - 26.1)
Peru	Streptococcus pneumoniae	3,735 (2,777 - 4,985)	11.4 (8.4 - 15.2)
Peru	Vibrio cholerae	0 (0 - 0)	0.0 (0.0 - 0.0)
Antigua and Barbuda	Acinetobacter baumannii	9 (5 - 14)	10.1 (5.7 - 15.8)
Antigua and Barbuda	Aeromonas spp.	0 (0 - 0)	0.0 (0.0 - 0.0)
Antigua and Barbuda	Campylobacter spp.	0 (0 - 0)	0.0 (0.0 - 0.1)
Antigua and Barbuda	Chlamydia spp.	1 (1 - 2)	1.6 (1.2 - 2.0)
Antigua and Barbuda	Citrobacter spp.	1 (0 - 1)	0.9 (0.5 - 1.4)

Antigua and Barbuda	Clostridioides difficile	0 (0 - 0)	0.3 (0.2 - 0.5)
Antigua and Barbuda	Enterobacter spp.	5 (3 - 8)	6.0 (3.6 - 9.0)
Antigua and Barbuda	Enterococcus faecalis	2 (1 - 4)	2.6 (1.6 - 4.2)
Antigua and Barbuda	Enterococcus faecium	2 (1 - 4)	2.5 (1.6 - 3.8)
Antigua and Barbuda	Escherichia coli	9 (6 - 12)	9.8 (7.0 - 13.3)
Antigua and Barbuda	Group A Streptococcus	5 (3 - 8)	5.4 (3.3 - 8.8)
Antigua and Barbuda	Group B Streptococcus	3 (2 - 4)	3.2 (2.1 - 4.9)
Antigua and Barbuda	Haemophilus influenzae	1 (1 - 1)	0.9 (0.7 - 1.2)
Antigua and Barbuda	Klebsiella pneumoniae	7 (5 - 10)	7.9 (5.4 - 11.0)
Antigua and Barbuda	Legionella spp.	1 (0 - 1)	0.7 (0.6 - 1.0)
Antigua and Barbuda	Listeria monocytogenes	0 (0 - 0)	0.1 (0.1 - 0.1)
Antigua and Barbuda	Morganella spp.	0 (0 - 0)	0.1 (0.1 - 0.2)
Antigua and Barbuda	Mycoplasma spp.	1 (1 - 2)	1.5 (1.1 - 1.9)
Antigua and Barbuda	Neisseria gonorrhoeae	0 (0 - 0)	0.0 (0.0 - 0.0)
Antigua and Barbuda	Neisseria meningitidis	1 (0 - 1)	0.9 (0.5 - 1.5)
Antigua and Barbuda	Non-typhoidal Salmonella	1 (0 - 1)	0.8 (0.5 - 1.3)
Antigua and Barbuda	Other Klebsiella species	1 (0 - 1)	0.6 (0.3 - 1.0)
Antigua and Barbuda	Other enterococci	2 (1 - 2)	2.0 (1.3 - 2.8)
Antigua and Barbuda	Proteus spp.	1 (1 - 2)	1.7 (1.2 - 2.4)
Antigua and Barbuda	Providencia spp.	0 (0 - 0)	0.1 (0.0 - 0.1)
Antigua and Barbuda	Pseudomonas aeruginosa	7 (4 - 10)	7.5 (5.1 - 10.8)
Antigua and Barbuda	Salmonella Paratyphi	0 (0 - 0)	0.0 (0.0 - 0.0)
Antigua and Barbuda	Salmonella Typhi	0 (0 - 0)	0.1 (0.1 - 0.2)
Antigua and Barbuda	Serratia spp.	1 (1 - 2)	1.3 (0.7 - 2.1)
Antigua and Barbuda	Shigella spp.	0 (0 - 0)	0.0 (0.0 - 0.1)
Antigua and Barbuda	Staphylococcus aureus	11 (7 - 14)	11.9 (8.5 - 16.2)
Antigua and Barbuda	Streptococcus pneumoniae	8 (6 - 10)	9.1 (7.1 - 11.6)
Antigua and Barbuda	Vibrio cholerae	0 (0 - 1)	0.5 (0.3 - 0.8)
Bahamas	Acinetobacter baumannii	20 (11 - 33)	5.7 (3.2 - 9.5)
Bahamas	Aeromonas spp.	0 (0 - 0)	0.0 (0.0 - 0.0)
Bahamas	Campylobacter spp.	0 (0 - 0)	0.0 (0.0 - 0.1)
Bahamas	Chlamydia spp.	2 (2 - 3)	0.7 (0.6 - 1.0)
Bahamas	Citrobacter spp.	2 (1 - 3)	0.6 (0.3 - 0.8)
Bahamas	Clostridioides difficile	2 (1 - 2)	0.5 (0.3 - 0.7)
Bahamas	Enterobacter spp.	12 (8 - 18)	3.5 (2.4 - 5.2)
Bahamas	Enterococcus faecalis	12 (7 - 18)	3.3 (2.0 - 5.1)
Bahamas	Enterococcus faecium	12 (7 - 19)	3.3 (2.0 - 5.0)
Bahamas	Escherichia coli	46 (32 - 66)	13.4 (9.2 - 19.0)
Bahamas	Group A Streptococcus	18 (11 - 30)	5.3 (3.3 - 8.8)
Bahamas	Group B Streptococcus	13 (8 - 19)	3.7 (2.5 - 5.6)
Bahamas	Haemophilus influenzae	3 (2 - 4)	1.0 (0.7 - 1.3)

Bahamas	Klebsiella pneumoniae	39 (26 - 56)	11.3 (7.7 - 16.3)
Bahamas	Legionella spp.	2 (2 - 3)	0.7 (0.5 - 0.9)
Bahamas	Listeria monocytogenes	0 (0 - 1)	0.1 (0.1 - 0.2)
Bahamas	Morganella spp.	0 (0 - 1)	0.1 (0.1 - 0.2)
Bahamas	Mycoplasma spp.	3 (2 - 3)	0.8 (0.6 - 1.0)
Bahamas	Neisseria gonorrhoeae	0 (0 - 0)	0.0 (0.0 - 0.0)
Bahamas	Neisseria meningitidis	4 (2 - 6)	1.1 (0.6 - 1.7)
Bahamas	Non-typhoidal Salmonella	2 (1 - 3)	0.5 (0.3 - 0.8)
Bahamas	Other Klebsiella species	3 (1 - 5)	0.7 (0.4 - 1.3)
Bahamas	Other enterococci	6 (4 - 9)	1.9 (1.3 - 2.7)
Bahamas	Proteus spp.	7 (5 - 10)	2.0 (1.4 - 2.9)
Bahamas	Providencia spp.	0 (0 - 1)	0.1 (0.1 - 0.2)
Bahamas	Pseudomonas aeruginosa	31 (21 - 45)	9.0 (6.1 - 13.1)
Bahamas	Salmonella Paratyphi	0 (0 - 0)	0.0 (0.0 - 0.0)
Bahamas	Salmonella Typhi	1 (1 - 2)	0.4 (0.2 - 0.6)
Bahamas	Serratia spp.	5 (3 - 7)	1.3 (0.7 - 2.0)
Bahamas	Shigella spp.	0 (0 - 0)	0.0 (0.0 - 0.0)
Bahamas	Staphylococcus aureus	61 (44 - 85)	17.7 (12.9 - 24.4)
Bahamas	Streptococcus pneumoniae	29 (21 - 39)	8.5 (6.4 - 11.3)
Bahamas	Vibrio cholerae	1 (0 - 1)	0.2 (0.1 - 0.4)
Barbados	Acinetobacter baumannii	26 (14 - 45)	5.5 (3.0 - 9.5)
Barbados	Aeromonas spp.	0 (0 - 0)	0.0 (0.0 - 0.0)
Barbados	Campylobacter spp.	0 (0 - 0)	0.0 (0.0 - 0.1)
Barbados	Chlamydia spp.	3 (3 - 4)	0.8 (0.6 - 1.0)
Barbados	Citrobacter spp.	3 (2 - 4)	0.6 (0.4 - 0.8)
Barbados	Clostridioides difficile	2 (1 - 2)	0.4 (0.2 - 0.6)
Barbados	Enterobacter spp.	17 (12 - 25)	3.8 (2.6 - 5.5)
Barbados	Enterococcus faecalis	17 (10 - 27)	3.7 (2.3 - 5.8)
Barbados	Enterococcus faecium	15 (10 - 24)	3.3 (2.0 - 5.0)
Barbados	Escherichia coli	75 (55 - 102)	16.0 (11.7 - 21.6)
Barbados	Group A Streptococcus	45 (28 - 74)	9.6 (6.1 - 15.8)
Barbados	Group B Streptococcus	22 (14 - 35)	4.9 (3.2 - 7.7)
Barbados	Haemophilus influenzae	4 (3 - 5)	1.0 (0.8 - 1.3)
Barbados	Klebsiella pneumoniae	52 (37 - 72)	11.4 (8.0 - 15.8)
Barbados	Legionella spp.	4 (3 - 5)	0.9 (0.7 - 1.2)
Barbados	Listeria monocytogenes	0 (0 - 1)	0.1 (0.1 - 0.1)
Barbados	Morganella spp.	1 (1 - 1)	0.2 (0.1 - 0.2)
Barbados	Mycoplasma spp.	4 (3 - 4)	0.9 (0.7 - 1.1)
Barbados	Neisseria gonorrhoeae	0 (0 - 0)	0.0 (0.0 - 0.0)
Barbados	Neisseria meningitidis	3 (1 - 4)	0.8 (0.5 - 1.3)

Barbados	Non-typhoidal Salmonella	2 (1 - 3)	0.5 (0.3 - 0.7)
Barbados	Other Klebsiella species	3 (1 - 5)	0.6 (0.3 - 1.0)
Barbados	Other enterococci	13 (9 - 18)	2.8 (1.9 - 3.9)
Barbados	Proteus spp.	12 (9 - 17)	2.5 (1.8 - 3.5)
Barbados	Providencia spp.	1 (0 - 1)	0.1 (0.1 - 0.2)
Barbados	Pseudomonas aeruginosa	47 (33 - 64)	10.1 (7.1 - 14.0)
Barbados	Salmonella Paratyphi	0 (0 - 0)	0.0 (0.0 - 0.0)
Barbados	Salmonella Typhi	1 (0 - 1)	0.2 (0.1 - 0.4)
Barbados	Serratia spp.	5 (3 - 8)	1.2 (0.7 - 1.8)
Barbados	Shigella spp.	0 (0 - 0)	0.0 (0.0 - 0.0)
Barbados	Staphylococcus aureus	96 (72 - 126)	20.7 (15.4 - 27.4)
Barbados	Streptococcus pneumoniae	38 (29 - 48)	8.7 (6.6 - 11.2)
Barbados	Vibrio cholerae	0 (0 - 1)	0.1 (0.0 - 0.2)
Belize	Acinetobacter baumannii	25 (15 - 40)	9.5 (5.5 - 14.8)
Belize	Aeromonas spp.	0 (0 - 0)	0.0 (0.0 - 0.0)
Belize	Campylobacter spp.	0 (0 - 1)	0.1 (0.0 - 0.2)
Belize	Chlamydia spp.	5 (4 - 7)	1.8 (1.4 - 2.4)
Belize	Citrobacter spp.	3 (2 - 4)	1.0 (0.6 - 1.6)
Belize	Clostridioides difficile	0 (0 - 0)	0.1 (0.0 - 0.1)
Belize	Enterobacter spp.	17 (10 - 25)	5.9 (3.7 - 8.8)
Belize	Enterococcus faecalis	8 (5 - 13)	3.0 (1.8 - 4.6)
Belize	Enterococcus faecium	8 (5 - 12)	2.8 (1.7 - 4.3)
Belize	Escherichia coli	29 (20 - 40)	10.8 (7.7 - 14.8)
Belize	Group A Streptococcus	9 (5 - 16)	3.2 (1.7 - 6.0)
Belize	Group B Streptococcus	9 (7 - 13)	3.3 (2.3 - 4.7)
Belize	Haemophilus influenzae	3 (2 - 4)	1.1 (0.9 - 1.4)
Belize	Klebsiella pneumoniae	25 (17 - 35)	9.4 (6.5 - 13.0)
Belize	Legionella spp.	2 (1 - 2)	0.6 (0.4 - 0.8)
Belize	Listeria monocytogenes	0 (0 - 0)	0.1 (0.0 - 0.1)
Belize	Morganella spp.	0 (0 - 0)	0.1 (0.1 - 0.2)
Belize	Mycoplasma spp.	5 (4 - 6)	1.5 (1.2 - 1.9)
Belize	Neisseria gonorrhoeae	0 (0 - 0)	0.0 (0.0 - 0.0)
Belize	Neisseria meningitidis	5 (3 - 8)	1.4 (0.8 - 2.2)
Belize	Non-typhoidal Salmonella	4 (3 - 7)	1.4 (0.8 - 2.2)
Belize	Other Klebsiella species	2 (1 - 4)	0.8 (0.4 - 1.4)
Belize	Other enterococci	4 (3 - 7)	1.7 (1.2 - 2.5)
Belize	Proteus spp.	5 (3 - 7)	1.8 (1.2 - 2.6)
Belize	Providencia spp.	0 (0 - 0)	0.1 (0.1 - 0.2)
Belize	Pseudomonas aeruginosa	21 (14 - 29)	7.6 (5.2 - 10.8)
Belize	Salmonella Paratyphi	0 (0 - 0)	0.0 (0.0 - 0.0)

Belize	Salmonella Typhi	1 (0 - 1)	0.2 (0.1 - 0.4)
Belize	Serratia spp.	5 (3 - 7)	1.6 (0.9 - 2.5)
Belize	Shigella spp.	0 (0 - 0)	0.1 (0.0 - 0.1)
Belize	Staphylococcus aureus	31 (22 - 43)	11.5 (8.2 - 15.6)
Belize	Streptococcus pneumoniae	32 (25 - 40)	10.9 (8.7 - 13.6)
Belize	Vibrio cholerae	4 (2 - 6)	1.3 (0.8 - 2.0)
Bermuda	Acinetobacter baumannii	6 (3 - 10)	5.0 (2.7 - 8.0)
Bermuda	Aeromonas spp.	0 (0 - 0)	0.0 (0.0 - 0.0)
Bermuda	Campylobacter spp.	0 (0 - 0)	0.0 (0.0 - 0.0)
Bermuda	Chlamydia spp.	0 (0 - 1)	0.3 (0.2 - 0.4)
Bermuda	Citrobacter spp.	1 (0 - 1)	0.4 (0.2 - 0.7)
Bermuda	Clostridioides difficile	0 (0 - 0)	0.2 (0.1 - 0.4)
Bermuda	Enterobacter spp.	5 (3 - 8)	4.0 (2.3 - 6.3)
Bermuda	Enterococcus faecalis	2 (1 - 3)	1.4 (0.8 - 2.3)
Bermuda	Enterococcus faecium	2 (1 - 3)	1.5 (0.9 - 2.4)
Bermuda	Escherichia coli	6 (4 - 9)	4.7 (3.1 - 6.9)
Bermuda	Group A Streptococcus	3 (1 - 5)	2.0 (1.0 - 3.6)
Bermuda	Group B Streptococcus	3 (2 - 4)	2.1 (1.4 - 3.2)
Bermuda	Haemophilus influenzae	0 (0 - 1)	0.3 (0.3 - 0.5)
Bermuda	Klebsiella pneumoniae	4 (3 - 6)	3.2 (2.0 - 4.7)
Bermuda	Legionella spp.	0 (0 - 0)	0.1 (0.1 - 0.2)
Bermuda	Listeria monocytogenes	0 (0 - 0)	0.0 (0.0 - 0.0)
Bermuda	Morganella spp.	0 (0 - 0)	0.0 (0.0 - 0.0)
Bermuda	Mycoplasma spp.	1 (0 - 1)	0.5 (0.3 - 0.7)
Bermuda	Neisseria gonorrhoeae	0 (0 - 0)	0.0 (0.0 - 0.0)
Bermuda	Neisseria meningitidis	0 (0 - 0)	0.3 (0.2 - 0.5)
Bermuda	Non-typhoidal Salmonella	0 (0 - 0)	0.2 (0.1 - 0.3)
Bermuda	Other Klebsiella species	0 (0 - 1)	0.3 (0.2 - 0.5)
Bermuda	Other enterococci	1 (1 - 2)	0.9 (0.5 - 1.3)
Bermuda	Proteus spp.	1 (1 - 1)	0.7 (0.4 - 1.0)
Bermuda	Providencia spp.	0 (0 - 0)	0.0 (0.0 - 0.0)
Bermuda	Pseudomonas aeruginosa	4 (3 - 6)	3.3 (2.1 - 5.0)
Bermuda	Salmonella Paratyphi	0 (0 - 0)	0.0 (0.0 - 0.0)
Bermuda	Salmonella Typhi	0 (0 - 0)	0.0 (0.0 - 0.0)
Bermuda	Serratia spp.	1 (0 - 1)	0.5 (0.3 - 0.9)
Bermuda	Shigella spp.	0 (0 - 0)	0.0 (0.0 - 0.0)
Bermuda	Staphylococcus aureus	8 (5 - 12)	6.1 (3.8 - 9.1)
Bermuda	Streptococcus pneumoniae	5 (3 - 6)	3.7 (2.7 - 5.0)
Bermuda	Vibrio cholerae	0 (0 - 0)	0.2 (0.1 - 0.3)
Cuba	Acinetobacter baumannii	1,247 (702 - 2,092)	6.6 (3.7 - 10.9)

Cuba	Aeromonas spp.	0 (0 - 1)	0.0 (0.0 - 0.0)
Cuba	Campylobacter spp.	7 (1 - 19)	0.0 (0.0 - 0.1)
Cuba	Chlamydia spp.	261 (199 - 341)	1.3 (1.0 - 1.7)
Cuba	Citrobacter spp.	103 (58 - 171)	0.6 (0.3 - 0.9)
Cuba	Clostridioides difficile	40 (24 - 63)	0.3 (0.2 - 0.4)
Cuba	Enterobacter spp.	782 (477 - 1,265)	4.3 (2.6 - 7.0)
Cuba	Enterococcus faecalis	346 (199 - 566)	1.9 (1.1 - 3.0)
Cuba	Enterococcus faecium	378 (234 - 585)	2.0 (1.2 - 3.1)
Cuba	Escherichia coli	1,245 (856 - 1,782)	6.5 (4.5 - 9.3)
Cuba	Group A Streptococcus	382 (188 - 714)	2.0 (1.0 - 3.8)
Cuba	Group B Streptococcus	344 (229 - 517)	1.9 (1.2 - 2.8)
Cuba	Haemophilus influenzae	138 (109 - 177)	0.7 (0.6 - 0.9)
Cuba	Klebsiella pneumoniae	994 (696 - 1,420)	5.2 (3.6 - 7.4)
Cuba	Legionella spp.	174 (136 - 217)	0.9 (0.7 - 1.1)
Cuba	Listeria monocytogenes	9 (6 - 14)	0.1 (0.0 - 0.1)
Cuba	Morganella spp.	8 (5 - 13)	0.0 (0.0 - 0.1)
Cuba	Mycoplasma spp.	267 (213 - 337)	1.5 (1.2 - 1.9)
Cuba	Neisseria gonorrhoeae	7 (6 - 9)	0.1 (0.0 - 0.1)
Cuba	Neisseria meningitidis	68 (39 - 115)	0.5 (0.3 - 0.8)
Cuba	Non-typhoidal Salmonella	64 (34 - 111)	0.4 (0.2 - 0.6)
Cuba	Other Klebsiella species	90 (50 - 151)	0.5 (0.3 - 0.8)
Cuba	Other enterococci	180 (110 - 282)	0.9 (0.6 - 1.5)
Cuba	Proteus spp.	170 (110 - 255)	0.9 (0.6 - 1.3)
Cuba	Providencia spp.	5 (3 - 8)	0.0 (0.0 - 0.0)
Cuba	Pseudomonas aeruginosa	1,016 (692 - 1,470)	5.3 (3.6 - 7.6)
Cuba	Salmonella Paratyphi	0 (0 - 0)	0.0 (0.0 - 0.0)
Cuba	Salmonella Typhi	6 (3 - 11)	0.0 (0.0 - 0.1)
Cuba	Serratia spp.	139 (80 - 233)	0.8 (0.4 - 1.3)
Cuba	Shigella spp.	3 (1 - 7)	0.0 (0.0 - 0.0)
Cuba	Staphylococcus aureus	1,869 (1,340 - 2,614)	9.7 (6.9 - 13.6)
Cuba	Streptococcus pneumoniae	1,411 (1,114 - 1,782)	7.4 (5.8 - 9.4)
Cuba	Vibrio cholerae	19 (10 - 33)	0.1 (0.1 - 0.2)
Dominica	Acinetobacter baumannii	12 (7 - 19)	14.4 (8.0 - 22.4)
Dominica	Aeromonas spp.	0 (0 - 0)	0.0 (0.0 - 0.0)
Dominica	Campylobacter spp.	0 (0 - 0)	0.1 (0.0 - 0.2)
Dominica	Chlamydia spp.	1 (1 - 2)	1.9 (1.3 - 2.7)
Dominica	Citrobacter spp.	1 (1 - 2)	1.3 (0.7 - 2.1)
Dominica	Clostridioides difficile	0 (0 - 0)	0.2 (0.1 - 0.4)
Dominica	Enterobacter spp.	6 (4 - 9)	7.8 (4.7 - 11.6)
Dominica	Enterococcus faecalis	3 (2 - 5)	3.3 (1.9 - 5.4)
Dominica	Enterococcus faecium	3 (2 - 4)	3.0 (1.8 - 4.7)

Dominica	Escherichia coli	9 (6 - 14)	11.0 (7.2 - 16.0)
Dominica	Group A Streptococcus	6 (4 - 10)	7.4 (4.7 - 11.5)
Dominica	Group B Streptococcus	3 (2 - 5)	4.2 (2.6 - 6.6)
Dominica	Haemophilus influenzae	1 (1 - 1)	1.2 (0.8 - 1.6)
Dominica	Klebsiella pneumoniae	9 (6 - 13)	11.0 (7.2 - 15.9)
Dominica	Legionella spp.	0 (0 - 1)	0.6 (0.4 - 0.9)
Dominica	Listeria monocytogenes	0 (0 - 0)	0.1 (0.1 - 0.2)
Dominica	Morganella spp.	0 (0 - 0)	0.1 (0.1 - 0.2)
Dominica	Mycoplasma spp.	1 (1 - 1)	1.5 (1.1 - 2.0)
Dominica	Neisseria gonorrhoeae	0 (0 - 0)	0.0 (0.0 - 0.0)
Dominica	Neisseria meningitidis	1 (1 - 2)	2.2 (1.3 - 3.5)
Dominica	Non-typhoidal Salmonella	2 (1 - 3)	2.1 (1.2 - 3.4)
Dominica	Other Klebsiella species	1 (0 - 1)	0.7 (0.4 - 1.4)
Dominica	Other enterococci	2 (1 - 3)	2.5 (1.5 - 3.9)
Dominica	Proteus spp.	2 (1 - 3)	2.1 (1.3 - 3.2)
Dominica	Providencia spp.	0 (0 - 0)	0.1 (0.1 - 0.2)
Dominica	Pseudomonas aeruginosa	8 (5 - 12)	9.6 (6.1 - 14.1)
Dominica	Salmonella Paratyphi	0 (0 - 0)	0.0 (0.0 - 0.0)
Dominica	Salmonella Typhi	0 (0 - 0)	0.4 (0.2 - 0.7)
Dominica	Serratia spp.	2 (1 - 3)	2.2 (1.2 - 3.5)
Dominica	Shigella spp.	0 (0 - 0)	0.0 (0.0 - 0.1)
Dominica	Staphylococcus aureus	11 (8 - 16)	13.4 (9.2 - 19.2)
Dominica	Streptococcus pneumoniae	9 (6 - 12)	11.2 (8.0 - 15.1)
Dominica	Vibrio cholerae	1 (0 - 2)	1.0 (0.5 - 1.9)
Dominican Republic	Acinetobacter baumannii	551 (309 - 891)	6.1 (3.4 - 9.8)
Dominican Republic	Aeromonas spp.	1 (0 - 2)	0.0 (0.0 - 0.0)
Dominican Republic	Campylobacter spp.	10 (3 - 25)	0.1 (0.0 - 0.3)
Dominican Republic	Chlamydia spp.	91 (59 - 132)	1.0 (0.6 - 1.4)
Dominican Republic	Citrobacter spp.	68 (40 - 106)	0.7 (0.4 - 1.1)
Dominican Republic	Clostridioides difficile	6 (2 - 11)	0.1 (0.0 - 0.1)
Dominican Republic	Enterobacter spp.	422 (259 - 639)	4.5 (2.7 - 6.8)
Dominican Republic	Enterococcus faecalis	312 (178 - 501)	3.3 (1.8 - 5.4)
Dominican Republic	Enterococcus faecium	299 (173 - 468)	3.2 (1.9 - 5.1)
Dominican Republic	Escherichia coli	1,058 (655 - 1,611)	11.6 (7.2 - 17.5)
Dominican Republic	Group A Streptococcus	312 (146 - 589)	3.4 (1.5 - 6.4)
Dominican Republic	Group B Streptococcus	401 (250 - 609)	4.1 (2.6 - 6.3)
Dominican Republic	Haemophilus influenzae	110 (73 - 158)	1.1 (0.8 - 1.6)
Dominican Republic	Klebsiella pneumoniae	1,099 (693 - 1,611)	11.8 (7.4 - 17.4)
Dominican Republic	Legionella spp.	50 (30 - 85)	0.5 (0.3 - 0.8)
Dominican Republic	Listeria monocytogenes	16 (10 - 25)	0.2 (0.1 - 0.3)
Dominican Republic	Morganella spp.	3 (1 - 5)	0.0 (0.0 - 0.1)

Dominican Republic	Mycoplasma spp.	89 (59 - 126)	0.9 (0.6 - 1.3)
Dominican Republic	Neisseria gonorrhoeae	7 (4 - 10)	0.1 (0.0 - 0.1)
Dominican Republic	Neisseria meningitidis	188 (116 - 285)	1.8 (1.1 - 2.7)
Dominican Republic	Non-typhoidal Salmonella	117 (71 - 183)	1.2 (0.7 - 1.9)
Dominican Republic	Other Klebsiella species	82 (39 - 150)	0.9 (0.4 - 1.6)
Dominican Republic	Other enterococci	123 (74 - 191)	1.3 (0.8 - 2.1)
Dominican Republic	Proteus spp.	141 (83 - 225)	1.6 (0.9 - 2.5)
Dominican Republic	Providencia spp.	3 (1 - 4)	0.0 (0.0 - 0.1)
Dominican Republic	Pseudomonas aeruginosa	791 (490 - 1,200)	8.6 (5.3 - 12.9)
Dominican Republic	Salmonella Paratyphi	1 (0 - 2)	0.0 (0.0 - 0.0)
Dominican Republic	Salmonella Typhi	66 (40 - 101)	0.6 (0.4 - 1.0)
Dominican Republic	Serratia spp.	159 (94 - 241)	1.7 (1.0 - 2.6)
Dominican Republic	Shigella spp.	9 (2 - 22)	0.1 (0.0 - 0.2)
Dominican Republic	Staphylococcus aureus	1,308 (842 - 1,945)	14.3 (9.3 - 21.1)
Dominican Republic	Streptococcus pneumoniae	904 (617 - 1,284)	9.5 (6.5 - 13.4)
Dominican Republic	Vibrio cholerae	202 (104 - 337)	2.2 (1.1 - 3.7)
Grenada	Acinetobacter baumannii	13 (8 - 20)	13.0 (7.7 - 20.2)
Grenada	Aeromonas spp.	0 (0 - 0)	0.0 (0.0 - 0.0)
Grenada	Campylobacter spp.	0 (0 - 0)	0.0 (0.0 - 0.1)
Grenada	Chlamydia spp.	2 (2 - 3)	2.2 (1.7 - 2.8)
Grenada	Citrobacter spp.	1 (1 - 2)	1.1 (0.7 - 1.8)
Grenada	Clostridioides difficile	0 (0 - 0)	0.3 (0.2 - 0.4)
Grenada	Enterobacter spp.	7 (5 - 11)	7.4 (4.8 - 10.9)
Grenada	Enterococcus faecalis	4 (2 - 5)	3.5 (2.1 - 5.4)
Grenada	Enterococcus faecium	3 (2 - 5)	3.2 (2.0 - 4.8)
Grenada	Escherichia coli	12 (9 - 17)	12.6 (9.1 - 16.7)
Grenada	Group A Streptococcus	7 (4 - 11)	7.3 (4.5 - 11.6)
Grenada	Group B Streptococcus	4 (3 - 6)	4.4 (2.9 - 6.5)
Grenada	Haemophilus influenzae	1 (1 - 2)	1.3 (1.0 - 1.6)
Grenada	Klebsiella pneumoniae	11 (7 - 15)	11.0 (7.7 - 15.1)
Grenada	Legionella spp.	1 (1 - 1)	0.8 (0.6 - 1.1)
Grenada	Listeria monocytogenes	0 (0 - 0)	0.1 (0.0 - 0.1)
Grenada	Morganella spp.	0 (0 - 0)	0.1 (0.1 - 0.2)
Grenada	Mycoplasma spp.	2 (1 - 2)	1.9 (1.5 - 2.3)
Grenada	Neisseria gonorrhoeae	0 (0 - 0)	0.0 (0.0 - 0.0)
Grenada	Neisseria meningitidis	1 (1 - 2)	1.3 (0.7 - 2.1)
Grenada	Non-typhoidal Salmonella	1 (1 - 2)	1.4 (0.8 - 2.2)
Grenada	Other Klebsiella species	1 (0 - 1)	0.8 (0.4 - 1.4)
Grenada	Other enterococci	2 (2 - 3)	2.5 (1.7 - 3.6)
Grenada	Proteus spp.	2 (2 - 3)	2.3 (1.6 - 3.2)

Grenada	Providencia spp.	0 (0 - 0)	0.1 (0.1 - 0.2)
Grenada	Pseudomonas aeruginosa	10 (7 - 13)	9.9 (6.9 - 13.8)
Grenada	Salmonella Paratyphi	0 (0 - 0)	0.0 (0.0 - 0.0)
Grenada	Salmonella Typhi	0 (0 - 0)	0.2 (0.1 - 0.4)
Grenada	Serratia spp.	2 (1 - 3)	1.9 (1.1 - 2.9)
Grenada	Shigella spp.	0 (0 - 0)	0.0 (0.0 - 0.1)
Grenada	Staphylococcus aureus	15 (11 - 20)	15.1 (11.2 - 20.0)
Grenada	Streptococcus pneumoniae	12 (10 - 15)	12.9 (10.5 - 16.0)
Grenada	Vibrio cholerae	0 (0 - 1)	0.4 (0.2 - 0.6)
Guyana	Acinetobacter baumannii	57 (30 - 95)	10.6 (5.6 - 17.3)
Guyana	Aeromonas spp.	0 (0 - 0)	0.0 (0.0 - 0.0)
Guyana	Campylobacter spp.	1 (0 - 3)	0.2 (0.1 - 0.5)
Guyana	Chlamydia spp.	8 (6 - 11)	1.4 (1.0 - 2.0)
Guyana	Citrobacter spp.	5 (3 - 8)	0.8 (0.5 - 1.3)
Guyana	Clostridioides difficile	1 (0 - 1)	0.1 (0.0 - 0.1)
Guyana	Enterobacter spp.	30 (18 - 47)	5.1 (3.2 - 7.8)
Guyana	Enterococcus faecalis	29 (16 - 46)	4.7 (2.7 - 7.7)
Guyana	Enterococcus faecium	28 (16 - 44)	4.6 (2.6 - 7.2)
Guyana	Escherichia coli	109 (69 - 160)	19.4 (12.6 - 28.1)
Guyana	Group A Streptococcus	29 (13 - 56)	5.2 (2.3 - 10.2)
Guyana	Group B Streptococcus	31 (20 - 46)	5.4 (3.5 - 8.0)
Guyana	Haemophilus influenzae	10 (7 - 14)	1.8 (1.2 - 2.4)
Guyana	Klebsiella pneumoniae	112 (72 - 164)	19.7 (12.9 - 28.4)
Guyana	Legionella spp.	6 (4 - 8)	1.0 (0.6 - 1.4)
Guyana	Listeria monocytogenes	1 (0 - 1)	0.1 (0.0 - 0.2)
Guyana	Morganella spp.	1 (0 - 1)	0.1 (0.1 - 0.2)
Guyana	Mycoplasma spp.	7 (5 - 9)	1.1 (0.8 - 1.5)
Guyana	Neisseria gonorrhoeae	1 (0 - 1)	0.1 (0.1 - 0.1)
Guyana	Neisseria meningitidis	13 (7 - 21)	1.9 (1.0 - 3.0)
Guyana	Non-typhoidal Salmonella	9 (5 - 16)	1.5 (0.8 - 2.6)
Guyana	Other Klebsiella species	8 (4 - 15)	1.3 (0.6 - 2.3)
Guyana	Other enterococci	11 (6 - 17)	2.0 (1.2 - 3.2)
Guyana	Proteus spp.	15 (9 - 24)	2.8 (1.7 - 4.3)
Guyana	Providencia spp.	1 (0 - 1)	0.1 (0.1 - 0.2)
Guyana	Pseudomonas aeruginosa	76 (48 - 114)	13.6 (8.7 - 19.9)
Guyana	Salmonella Paratyphi	0 (0 - 0)	0.0 (0.0 - 0.0)
Guyana	Salmonella Typhi	9 (5 - 15)	1.4 (0.8 - 2.2)
Guyana	Serratia spp.	13 (7 - 21)	2.1 (1.2 - 3.5)
Guyana	Shigella spp.	1 (0 - 2)	0.2 (0.1 - 0.3)
Guyana	Staphylococcus aureus	146 (98 - 209)	26.7 (18.6 - 37.5)

Guyana	Streptococcus pneumoniae	82 (58 - 113)	14.3 (10.2 - 19.4)
Guyana	Vibrio cholerae	7 (4 - 12)	1.3 (0.7 - 2.2)
Haiti	Acinetobacter baumannii	1,210 (736 - 1,919)	18.7 (11.1 - 30.2)
Haiti	Aeromonas spp.	11 (3 - 23)	0.1 (0.0 - 0.2)
Haiti	Campylobacter spp.	75 (22 - 184)	0.7 (0.2 - 1.8)
Haiti	Chlamydia spp.	359 (256 - 497)	3.5 (2.5 - 4.7)
Haiti	Citrobacter spp.	159 (93 - 248)	1.8 (1.0 - 2.9)
Haiti	Clostridioides difficile	4 (1 - 10)	0.0 (0.0 - 0.1)
Haiti	Enterobacter spp.	716 (468 - 1,033)	8.5 (5.2 - 12.6)
Haiti	Enterococcus faecalis	373 (222 - 586)	4.8 (2.7 - 7.8)
Haiti	Enterococcus faecium	280 (156 - 474)	3.9 (2.1 - 6.6)
Haiti	Escherichia coli	1,473 (1,056 - 2,024)	18.4 (12.8 - 25.8)
Haiti	Group A Streptococcus	500 (288 - 873)	7.7 (4.0 - 13.9)
Haiti	Group B Streptococcus	928 (653 - 1,264)	8.9 (6.2 - 12.6)
Haiti	Haemophilus influenzae	365 (270 - 483)	3.1 (2.3 - 4.2)
Haiti	Klebsiella pneumoniae	1,876 (1,331 - 2,564)	21.9 (15.1 - 30.9)
Haiti	Legionella spp.	49 (27 - 87)	0.5 (0.3 - 0.7)
Haiti	Listeria monocytogenes	71 (41 - 114)	0.6 (0.3 - 0.9)
Haiti	Morganella spp.	8 (4 - 13)	0.1 (0.1 - 0.2)
Haiti	Mycoplasma spp.	238 (176 - 316)	2.0 (1.5 - 2.6)
Haiti	Neisseria gonorrhoeae	12 (7 - 22)	0.1 (0.1 - 0.2)
Haiti	Neisseria meningitidis	883 (588 - 1,287)	6.9 (4.5 - 10.2)
Haiti	Non-typhoidal Salmonella	768 (488 - 1,146)	7.5 (4.5 - 11.4)
Haiti	Other Klebsiella species	111 (46 - 218)	1.5 (0.6 - 3.0)
Haiti	Other enterococci	195 (119 - 305)	2.8 (1.7 - 4.6)
Haiti	Proteus spp.	209 (123 - 323)	3.3 (2.0 - 5.2)
Haiti	Providencia spp.	13 (7 - 22)	0.2 (0.1 - 0.4)
Haiti	Pseudomonas aeruginosa	1,001 (693 - 1,387)	12.8 (8.5 - 18.5)
Haiti	Salmonella Paratyphi	1 (1 - 3)	0.0 (0.0 - 0.0)
Haiti	Salmonella Typhi	344 (207 - 529)	2.9 (1.7 - 4.5)
Haiti	Serratia spp.	327 (200 - 518)	3.8 (2.2 - 6.3)
Haiti	Shigella spp.	159 (46 - 373)	1.2 (0.4 - 2.7)
Haiti	Staphylococcus aureus	1,447 (1,032 - 1,992)	18.2 (12.8 - 25.5)
Haiti	Streptococcus pneumoniae	2,619 (1,946 - 3,405)	24.7 (18.5 - 32.6)
Haiti	Vibrio cholerae	518 (231 - 1,054)	6.2 (2.5 - 13.6)
Jamaica	Acinetobacter baumannii	296 (160 - 478)	9.7 (5.3 - 15.5)
Jamaica	Aeromonas spp.	0 (0 - 0)	0.0 (0.0 - 0.0)
Jamaica	Campylobacter spp.	1 (0 - 3)	0.0 (0.0 - 0.1)
Jamaica	Chlamydia spp.	33 (22 - 49)	1.2 (0.7 - 1.9)
Jamaica	Citrobacter spp.	24 (13 - 39)	0.8 (0.5 - 1.3)

Jamaica	Clostridioides difficile	3 (2 - 5)	0.1 (0.1 - 0.2)
Jamaica	Enterobacter spp.	158 (92 - 244)	5.5 (3.3 - 8.5)
Jamaica	Enterococcus faecalis	70 (41 - 116)	2.3 (1.4 - 3.8)
Jamaica	Enterococcus faecium	64 (37 - 102)	2.1 (1.2 - 3.3)
Jamaica	Escherichia coli	256 (171 - 370)	8.4 (5.5 - 12.1)
Jamaica	Group A Streptococcus	153 (86 - 263)	4.8 (2.6 - 8.4)
Jamaica	Group B Streptococcus	87 (52 - 139)	3.0 (1.8 - 4.8)
Jamaica	Haemophilus influenzae	19 (12 - 28)	0.7 (0.4 - 1.0)
Jamaica	Klebsiella pneumoniae	213 (134 - 316)	7.2 (4.5 - 10.7)
Jamaica	Legionella spp.	15 (9 - 23)	0.6 (0.3 - 1.0)
Jamaica	Listeria monocytogenes	3 (1 - 5)	0.1 (0.0 - 0.2)
Jamaica	Morganella spp.	3 (2 - 4)	0.1 (0.1 - 0.1)
Jamaica	Mycoplasma spp.	27 (18 - 39)	1.0 (0.7 - 1.5)
Jamaica	Neisseria gonorrhoeae	1 (1 - 2)	0.0 (0.0 - 0.1)
Jamaica	Neisseria meningitidis	32 (17 - 51)	1.2 (0.7 - 1.9)
Jamaica	Non-typhoidal Salmonella	28 (15 - 45)	1.0 (0.6 - 1.6)
Jamaica	Other Klebsiella species	13 (6 - 24)	0.4 (0.2 - 0.8)
Jamaica	Other enterococci	57 (36 - 86)	1.9 (1.2 - 2.8)
Jamaica	Proteus spp.	47 (31 - 69)	1.5 (1.0 - 2.2)
Jamaica	Providencia spp.	2 (1 - 3)	0.1 (0.0 - 0.1)
Jamaica	Pseudomonas aeruginosa	198 (122 - 304)	6.6 (4.0 - 10.1)
Jamaica	Salmonella Paratyphi	0 (0 - 0)	0.0 (0.0 - 0.0)
Jamaica	Salmonella Typhi	5 (3 - 8)	0.2 (0.1 - 0.3)
Jamaica	Serratia spp.	40 (22 - 65)	1.4 (0.8 - 2.2)
Jamaica	Shigella spp.	1 (0 - 1)	0.0 (0.0 - 0.0)
Jamaica	Staphylococcus aureus	292 (189 - 430)	9.6 (6.2 - 14.2)
Jamaica	Streptococcus pneumoniae	186 (125 - 268)	6.3 (4.2 - 9.2)
Jamaica	Vibrio cholerae	27 (17 - 43)	0.9 (0.6 - 1.4)
Puerto Rico	Acinetobacter baumannii	211 (112 - 349)	3.0 (1.6 - 4.9)
Puerto Rico	Aeromonas spp.	0 (0 - 0)	0.0 (0.0 - 0.0)
Puerto Rico	Campylobacter spp.	3 (0 - 7)	0.0 (0.0 - 0.1)
Puerto Rico	Chlamydia spp.	27 (20 - 36)	0.4 (0.3 - 0.5)
Puerto Rico	Citrobacter spp.	24 (15 - 37)	0.4 (0.2 - 0.6)
Puerto Rico	Clostridioides difficile	27 (17 - 41)	0.5 (0.3 - 0.7)
Puerto Rico	Enterobacter spp.	163 (102 - 246)	2.4 (1.5 - 3.7)
Puerto Rico	Enterococcus faecalis	157 (93 - 257)	2.3 (1.4 - 3.7)
Puerto Rico	Enterococcus faecium	168 (98 - 263)	2.5 (1.4 - 3.8)
Puerto Rico	Escherichia coli	659 (439 - 943)	8.8 (5.8 - 12.7)
Puerto Rico	Group A Streptococcus	219 (121 - 366)	3.0 (1.7 - 5.0)
Puerto Rico	Group B Streptococcus	159 (99 - 245)	2.4 (1.5 - 3.6)
Puerto Rico	Haemophilus influenzae	35 (25 - 47)	0.5 (0.4 - 0.7)

Puerto Rico	Klebsiella pneumoniae	421 (274 - 620)	5.9 (3.7 - 8.8)
Puerto Rico	Legionella spp.	44 (34 - 58)	0.6 (0.5 - 0.8)
Puerto Rico	Listeria monocytogenes	2 (1 - 3)	0.0 (0.0 - 0.1)
Puerto Rico	Morganella spp.	5 (4 - 7)	0.1 (0.0 - 0.1)
Puerto Rico	Mycoplasma spp.	37 (28 - 48)	0.6 (0.4 - 0.8)
Puerto Rico	Neisseria gonorrhoeae	1 (0 - 1)	0.0 (0.0 - 0.0)
Puerto Rico	Neisseria meningitidis	16 (8 - 26)	0.4 (0.2 - 0.7)
Puerto Rico	Non-typhoidal Salmonella	10 (5 - 19)	0.2 (0.1 - 0.3)
Puerto Rico	Other Klebsiella species	30 (16 - 51)	0.5 (0.2 - 0.8)
Puerto Rico	Other enterococci	86 (56 - 123)	1.1 (0.7 - 1.6)
Puerto Rico	Proteus spp.	87 (58 - 123)	1.1 (0.7 - 1.6)
Puerto Rico	Providencia spp.	3 (2 - 4)	0.0 (0.0 - 0.0)
Puerto Rico	Pseudomonas aeruginosa	400 (262 - 591)	5.5 (3.5 - 8.3)
Puerto Rico	Salmonella Paratyphi	0 (0 - 0)	0.0 (0.0 - 0.0)
Puerto Rico	Salmonella Typhi	3 (2 - 5)	0.1 (0.0 - 0.1)
Puerto Rico	Serratia spp.	41 (22 - 65)	0.6 (0.4 - 1.0)
Puerto Rico	Shigella spp.	1 (0 - 3)	0.0 (0.0 - 0.0)
Puerto Rico	Staphylococcus aureus	894 (603 - 1,280)	12.3 (8.1 - 17.9)
Puerto Rico	Streptococcus pneumoniae	315 (230 - 429)	4.6 (3.3 - 6.4)
Puerto Rico	Vibrio cholerae	11 (5 - 20)	0.2 (0.1 - 0.3)
Saint Kitts and Nevis	Acinetobacter baumannii	7 (4 - 11)	12.5 (7.2 - 19.6)
Saint Kitts and Nevis	Aeromonas spp.	0 (0 - 0)	0.0 (0.0 - 0.0)
Saint Kitts and Nevis	Campylobacter spp.	0 (0 - 0)	0.1 (0.0 - 0.3)
Saint Kitts and Nevis	Chlamydia spp.	1 (1 - 1)	1.9 (1.4 - 2.4)
Saint Kitts and Nevis	Citrobacter spp.	1 (0 - 1)	1.2 (0.7 - 1.9)
Saint Kitts and Nevis	Clostridioides difficile	0 (0 - 0)	0.4 (0.2 - 0.7)
Saint Kitts and Nevis	Enterobacter spp.	5 (3 - 7)	8.0 (4.9 - 11.9)
Saint Kitts and Nevis	Enterococcus faecalis	2 (1 - 3)	3.8 (2.3 - 5.9)
Saint Kitts and Nevis	Enterococcus faecium	2 (1 - 3)	3.6 (2.3 - 5.3)
Saint Kitts and Nevis	Escherichia coli	8 (5 - 10)	13.7 (10.1 - 18.2)
Saint Kitts and Nevis	Group A Streptococcus	3 (2 - 6)	6.6 (4.0 - 10.9)
Saint Kitts and Nevis	Group B Streptococcus	2 (1 - 3)	4.0 (2.6 - 6.1)
Saint Kitts and Nevis	Haemophilus influenzae	1 (0 - 1)	1.1 (0.8 - 1.4)
Saint Kitts and Nevis	Klebsiella pneumoniae	6 (4 - 8)	10.0 (6.9 - 14.0)
Saint Kitts and Nevis	Legionella spp.	1 (0 - 1)	1.0 (0.7 - 1.3)
Saint Kitts and Nevis	Listeria monocytogenes	0 (0 - 0)	0.1 (0.1 - 0.1)
Saint Kitts and Nevis	Morganella spp.	0 (0 - 0)	0.1 (0.1 - 0.2)
Saint Kitts and Nevis	Mycoplasma spp.	1 (1 - 1)	1.8 (1.4 - 2.4)
Saint Kitts and Nevis	Neisseria gonorrhoeae	0 (0 - 0)	0.1 (0.0 - 0.1)
Saint Kitts and Nevis	Neisseria meningitidis	1 (0 - 1)	1.1 (0.7 - 1.7)

Saint Kitts and Nevis	Non-typhoidal Salmonella	1 (0 - 1)	1.0 (0.6 - 1.7)
Saint Kitts and Nevis	Other Klebsiella species	1 (0 - 1)	0.8 (0.5 - 1.4)
Saint Kitts and Nevis	Other enterococci	1 (1 - 2)	2.5 (1.7 - 3.6)
Saint Kitts and Nevis	Proteus spp.	1 (1 - 2)	2.3 (1.6 - 3.1)
Saint Kitts and Nevis	Providencia spp.	0 (0 - 0)	0.1 (0.1 - 0.1)
Saint Kitts and Nevis	Pseudomonas aeruginosa	5 (4 - 7)	9.6 (6.4 - 13.4)
Saint Kitts and Nevis	Salmonella Paratyphi	0 (0 - 0)	0.0 (0.0 - 0.0)
Saint Kitts and Nevis	Salmonella Typhi	0 (0 - 0)	0.1 (0.1 - 0.2)
Saint Kitts and Nevis	Serratia spp.	1 (1 - 2)	1.6 (1.0 - 2.6)
Saint Kitts and Nevis	Shigella spp.	0 (0 - 0)	0.1 (0.0 - 0.2)
Saint Kitts and Nevis	Staphylococcus aureus	9 (6 - 12)	15.3 (10.8 - 21.1)
Saint Kitts and Nevis	Streptococcus pneumoniae	6 (4 - 7)	10.5 (8.1 - 13.6)
Saint Kitts and Nevis	Vibrio cholerae	0 (0 - 0)	0.3 (0.2 - 0.6)
Saint Lucia	Acinetobacter baumannii	22 (12 - 34)	10.9 (6.2 - 17.2)
Saint Lucia	Aeromonas spp.	0 (0 - 0)	0.0 (0.0 - 0.0)
Saint Lucia	Campylobacter spp.	0 (0 - 0)	0.1 (0.0 - 0.1)
Saint Lucia	Chlamydia spp.	3 (2 - 4)	1.5 (1.1 - 2.1)
Saint Lucia	Citrobacter spp.	2 (1 - 3)	1.0 (0.6 - 1.5)
Saint Lucia	Clostridioides difficile	0 (0 - 1)	0.2 (0.1 - 0.3)
Saint Lucia	Enterobacter spp.	12 (7 - 19)	6.5 (4.0 - 9.7)
Saint Lucia	Enterococcus faecalis	6 (3 - 9)	2.9 (1.7 - 4.5)
Saint Lucia	Enterococcus faecium	6 (3 - 9)	2.7 (1.7 - 4.2)
Saint Lucia	Escherichia coli	21 (14 - 29)	10.4 (7.2 - 14.5)
Saint Lucia	Group A Streptococcus	10 (6 - 17)	5.3 (3.2 - 8.8)
Saint Lucia	Group B Streptococcus	6 (4 - 10)	3.4 (2.2 - 5.3)
Saint Lucia	Haemophilus influenzae	2 (1 - 2)	0.9 (0.6 - 1.2)
Saint Lucia	Klebsiella pneumoniae	17 (11 - 24)	8.6 (5.7 - 12.4)
Saint Lucia	Legionella spp.	1 (1 - 1)	0.6 (0.4 - 0.9)
Saint Lucia	Listeria monocytogenes	0 (0 - 0)	0.1 (0.1 - 0.1)
Saint Lucia	Morganella spp.	0 (0 - 0)	0.1 (0.1 - 0.2)
Saint Lucia	Mycoplasma spp.	2 (2 - 3)	1.3 (1.0 - 1.8)
Saint Lucia	Neisseria gonorrhoeae	0 (0 - 0)	0.1 (0.0 - 0.1)
Saint Lucia	Neisseria meningitidis	2 (1 - 3)	1.2 (0.6 - 1.9)
Saint Lucia	Non-typhoidal Salmonella	2 (1 - 3)	1.1 (0.7 - 1.8)
Saint Lucia	Other Klebsiella species	1 (1 - 2)	0.6 (0.3 - 1.1)
Saint Lucia	Other enterococci	4 (3 - 6)	2.1 (1.4 - 3.1)
Saint Lucia	Proteus spp.	4 (2 - 5)	1.8 (1.2 - 2.6)
Saint Lucia	Providencia spp.	0 (0 - 0)	0.1 (0.1 - 0.2)
Saint Lucia	Pseudomonas aeruginosa	15 (10 - 23)	7.9 (5.1 - 11.6)
Saint Lucia	Salmonella Paratyphi	0 (0 - 0)	0.0 (0.0 - 0.0)

Saint Lucia	Salmonella Typhi	0 (0 - 1)	0.2 (0.1 - 0.3)
Saint Lucia	Serratia spp.	3 (2 - 5)	1.5 (0.9 - 2.4)
Saint Lucia	Shigella spp.	0 (0 - 0)	0.0 (0.0 - 0.1)
Saint Lucia	Staphylococcus aureus	23 (15 - 32)	11.6 (7.9 - 16.1)
Saint Lucia	Streptococcus pneumoniae	16 (12 - 21)	8.7 (6.4 - 11.6)
Saint Lucia	Vibrio cholerae	2 (1 - 3)	1.1 (0.7 - 1.7)
Saint Vincent and the Grenadines	Acinetobacter baumannii	16 (9 - 25)	13.2 (7.5 - 20.9)
Saint Vincent and the Grenadines	Aeromonas spp.	0 (0 - 0)	0.0 (0.0 - 0.0)
Saint Vincent and the Grenadines	Campylobacter spp.	0 (0 - 0)	0.1 (0.0 - 0.2)
Saint Vincent and the Grenadines	Chlamydia spp.	2 (1 - 3)	1.8 (1.3 - 2.5)
Saint Vincent and the Grenadines	Citrobacter spp.	1 (1 - 2)	1.1 (0.7 - 1.8)
Saint Vincent and the Grenadines	Clostridioides difficile	0 (0 - 0)	0.1 (0.1 - 0.2)
Saint Vincent and the Grenadines	Enterobacter spp.	8 (5 - 12)	6.8 (4.2 - 10.1)
Saint Vincent and the Grenadines	Enterococcus faecalis	4 (2 - 6)	3.2 (2.0 - 4.9)
Saint Vincent and the Grenadines	Enterococcus faecium	4 (2 - 6)	2.8 (1.7 - 4.4)
Saint Vincent and the Grenadines	Escherichia coli	14 (10 - 19)	11.6 (8.2 - 16.0)
Saint Vincent and the Grenadines	Group A Streptococcus	7 (4 - 12)	6.0 (3.6 - 10.2)
Saint Vincent and the Grenadines	Group B Streptococcus	4 (3 - 7)	3.8 (2.4 - 5.7)
Saint Vincent and the Grenadines	Haemophilus influenzae	1 (1 - 2)	1.1 (0.8 - 1.4)
Saint Vincent and the Grenadines	Klebsiella pneumoniae	12 (8 - 17)	10.2 (6.9 - 14.5)
Saint Vincent and the Grenadines	Legionella spp.	1 (0 - 1)	0.6 (0.4 - 0.9)
Saint Vincent and the Grenadines	Listeria monocytogenes	0 (0 - 0)	0.1 (0.1 - 0.2)
Saint Vincent and the Grenadines	Morganella spp.	0 (0 - 0)	0.2 (0.1 - 0.2)
Saint Vincent and the Grenadines	Mycoplasma spp.	2 (1 - 2)	1.4 (1.0 - 1.8)
Saint Vincent and the Grenadines	Neisseria gonorrhoeae	0 (0 - 0)	0.0 (0.0 - 0.0)
Saint Vincent and the Grenadines	Neisseria meningitidis	2 (1 - 3)	1.7 (1.0 - 2.6)
Saint Vincent and the Grenadines	Non-typhoidal Salmonella	2 (1 - 3)	1.7 (1.0 - 2.6)
Saint Vincent and the Grenadines	Other Klebsiella species	1 (0 - 2)	0.7 (0.4 - 1.3)
Saint Vincent and the Grenadines	Other enterococci	3 (2 - 4)	2.4 (1.6 - 3.5)
Saint Vincent and the Grenadines	Proteus spp.	3 (2 - 4)	2.2 (1.5 - 3.1)
Saint Vincent and the Grenadines	Providencia spp.	0 (0 - 0)	0.1 (0.1 - 0.2)
Saint Vincent and the Grenadines	Pseudomonas aeruginosa	11 (7 - 15)	8.8 (5.8 - 12.8)
Saint Vincent and the Grenadines	Salmonella Paratyphi	0 (0 - 0)	0.0 (0.0 - 0.0)
Saint Vincent and the Grenadines	Salmonella Typhi	0 (0 - 1)	0.3 (0.2 - 0.5)
Saint Vincent and the Grenadines	Serratia spp.	2 (1 - 4)	1.9 (1.1 - 2.9)
Saint Vincent and the Grenadines	Shigella spp.	0 (0 - 0)	0.1 (0.0 - 0.1)
Saint Vincent and the Grenadines	Staphylococcus aureus	15 (11 - 21)	12.7 (8.9 - 17.5)
Saint Vincent and the Grenadines	Streptococcus pneumoniae	12 (9 - 15)	10.1 (7.6 - 13.4)
Saint Vincent and the Grenadines	Vibrio cholerae	1 (1 - 2)	1.0 (0.6 - 1.5)
Suriname	Acinetobacter baumannii	67 (39 - 106)	12.3 (7.1 - 19.3)

Suriname	Aeromonas spp.	0 (0 - 0)	0.0 (0.0 - 0.0)
Suriname	Campylobacter spp.	1 (0 - 2)	0.1 (0.0 - 0.3)
Suriname	Chlamydia spp.	10 (7 - 14)	2.0 (1.4 - 2.8)
Suriname	Citrobacter spp.	7 (4 - 11)	1.3 (0.8 - 2.0)
Suriname	Clostridioides difficile	1 (0 - 1)	0.1 (0.1 - 0.2)
Suriname	Enterobacter spp.	39 (24 - 57)	7.2 (4.5 - 10.4)
Suriname	Enterococcus faecalis	21 (13 - 33)	3.7 (2.3 - 5.8)
Suriname	Enterococcus faecium	19 (12 - 29)	3.3 (2.0 - 5.0)
Suriname	Escherichia coli	73 (51 - 98)	13.2 (9.4 - 17.8)
Suriname	Group A Streptococcus	33 (21 - 52)	6.1 (3.9 - 9.5)
Suriname	Group B Streptococcus	20 (13 - 31)	3.9 (2.5 - 5.8)
Suriname	Haemophilus influenzae	6 (4 - 8)	1.1 (0.8 - 1.5)
Suriname	Klebsiella pneumoniae	61 (41 - 87)	11.3 (7.6 - 16.0)
Suriname	Legionella spp.	3 (2 - 5)	0.7 (0.4 - 1.1)
Suriname	Listeria monocytogenes	1 (0 - 1)	0.1 (0.1 - 0.2)
Suriname	Morganella spp.	1 (1 - 1)	0.2 (0.1 - 0.2)
Suriname	Mycoplasma spp.	7 (5 - 10)	1.5 (1.0 - 2.0)
Suriname	Neisseria gonorrhoeae	0 (0 - 0)	0.0 (0.0 - 0.0)
Suriname	Neisseria meningitidis	12 (7 - 18)	2.2 (1.3 - 3.4)
Suriname	Non-typhoidal Salmonella	12 (7 - 18)	2.2 (1.3 - 3.4)
Suriname	Other Klebsiella species	6 (3 - 10)	1.0 (0.5 - 1.7)
Suriname	Other enterococci	13 (9 - 19)	2.5 (1.7 - 3.6)
Suriname	Proteus spp.	13 (9 - 19)	2.4 (1.7 - 3.4)
Suriname	Providencia spp.	1 (1 - 1)	0.2 (0.1 - 0.2)
Suriname	Pseudomonas aeruginosa	48 (32 - 69)	8.9 (5.9 - 12.7)
Suriname	Salmonella Paratyphi	0 (0 - 0)	0.0 (0.0 - 0.0)
Suriname	Salmonella Typhi	2 (1 - 4)	0.5 (0.3 - 0.7)
Suriname	Serratia spp.	12 (7 - 18)	2.1 (1.2 - 3.2)
Suriname	Shigella spp.	0 (0 - 1)	0.1 (0.0 - 0.2)
Suriname	Staphylococcus aureus	70 (49 - 98)	12.8 (8.9 - 17.8)
Suriname	Streptococcus pneumoniae	53 (38 - 71)	10.0 (7.3 - 13.4)
Suriname	Vibrio cholerae	18 (10 - 31)	3.5 (1.8 - 5.8)
Trinidad and Tobago	Acinetobacter baumannii	93 (48 - 157)	5.4 (2.8 - 9.1)
Trinidad and Tobago	Aeromonas spp.	0 (0 - 0)	0.0 (0.0 - 0.0)
Trinidad and Tobago	Campylobacter spp.	1 (0 - 2)	0.0 (0.0 - 0.1)
Trinidad and Tobago	Chlamydia spp.	9 (6 - 13)	0.6 (0.4 - 0.9)
Trinidad and Tobago	Citrobacter spp.	9 (5 - 14)	0.5 (0.3 - 0.9)
Trinidad and Tobago	Clostridioides difficile	5 (3 - 8)	0.4 (0.2 - 0.6)
Trinidad and Tobago	Enterobacter spp.	57 (33 - 90)	3.4 (2.0 - 5.4)
Trinidad and Tobago	Enterococcus faecalis	47 (26 - 80)	2.8 (1.6 - 4.6)
Trinidad and Tobago	Enterococcus faecium	51 (28 - 82)	2.9 (1.6 - 4.7)

Trinidad and Tobago	Escherichia coli	188 (115 - 284)	11.1 (6.8 - 16.6)
Trinidad and Tobago	Group A Streptococcus	68 (35 - 128)	4.1 (2.1 - 7.4)
Trinidad and Tobago	Group B Streptococcus	52 (31 - 82)	3.3 (2.0 - 5.2)
Trinidad and Tobago	Haemophilus influenzae	11 (7 - 17)	0.8 (0.5 - 1.1)
Trinidad and Tobago	Klebsiella pneumoniae	163 (99 - 248)	9.8 (6.0 - 15.0)
Trinidad and Tobago	Legionella spp.	6 (4 - 9)	0.4 (0.3 - 0.7)
Trinidad and Tobago	Listeria monocytogenes	1 (1 - 2)	0.1 (0.0 - 0.1)
Trinidad and Tobago	Morganella spp.	2 (1 - 3)	0.1 (0.1 - 0.2)
Trinidad and Tobago	Mycoplasma spp.	9 (6 - 12)	0.6 (0.4 - 0.9)
Trinidad and Tobago	Neisseria gonorrhoeae	0 (0 - 1)	0.0 (0.0 - 0.0)
Trinidad and Tobago	Neisseria meningitidis	15 (8 - 25)	1.1 (0.6 - 1.8)
Trinidad and Tobago	Non-typhoidal Salmonella	10 (5 - 16)	0.6 (0.3 - 1.0)
Trinidad and Tobago	Other Klebsiella species	10 (5 - 18)	0.6 (0.3 - 1.0)
Trinidad and Tobago	Other enterococci	27 (16 - 42)	1.6 (1.0 - 2.5)
Trinidad and Tobago	Proteus spp.	30 (18 - 45)	1.7 (1.0 - 2.6)
Trinidad and Tobago	Providencia spp.	2 (1 - 3)	0.1 (0.0 - 0.1)
Trinidad and Tobago	Pseudomonas aeruginosa	130 (77 - 202)	7.8 (4.6 - 12.1)
Trinidad and Tobago	Salmonella Paratyphi	0 (0 - 0)	0.0 (0.0 - 0.0)
Trinidad and Tobago	Salmonella Typhi	5 (3 - 9)	0.4 (0.2 - 0.6)
Trinidad and Tobago	Serratia spp.	22 (12 - 36)	1.3 (0.7 - 2.1)
Trinidad and Tobago	Shigella spp.	0 (0 - 1)	0.0 (0.0 - 0.1)
Trinidad and Tobago	Staphylococcus aureus	225 (143 - 340)	13.3 (8.5 - 20.2)
Trinidad and Tobago	Streptococcus pneumoniae	109 (71 - 160)	6.9 (4.5 - 10.1)
Trinidad and Tobago	Vibrio cholerae	5 (3 - 10)	0.3 (0.2 - 0.6)
United States Virgin Islands	Acinetobacter baumannii	10 (5 - 16)	5.7 (3.2 - 9.3)
United States Virgin Islands	Aeromonas spp.	0 (0 - 0)	0.0 (0.0 - 0.0)
United States Virgin Islands	Campylobacter spp.	0 (0 - 0)	0.0 (0.0 - 0.1)
United States Virgin Islands	Chlamydia spp.	1 (1 - 1)	0.6 (0.4 - 0.9)
United States Virgin Islands	Citrobacter spp.	1 (1 - 2)	0.6 (0.4 - 1.0)
United States Virgin Islands	Clostridioides difficile	1 (0 - 1)	0.4 (0.3 - 0.7)
United States Virgin Islands	Enterobacter spp.	7 (4 - 10)	4.3 (2.7 - 6.3)
United States Virgin Islands	Enterococcus faecalis	5 (3 - 8)	3.2 (2.0 - 5.0)
United States Virgin Islands	Enterococcus faecium	6 (4 - 9)	3.4 (2.0 - 5.1)
United States Virgin Islands	Escherichia coli	21 (14 - 29)	12.5 (8.6 - 17.3)
United States Virgin Islands	Group A Streptococcus	7 (4 - 11)	4.4 (2.8 - 7.0)
United States Virgin Islands	Group B Streptococcus	5 (4 - 8)	3.5 (2.3 - 5.2)
United States Virgin Islands	Haemophilus influenzae	1 (1 - 2)	0.7 (0.5 - 1.1)
United States Virgin Islands	Klebsiella pneumoniae	16 (11 - 23)	9.5 (6.4 - 13.7)
United States Virgin Islands	Legionella spp.	1 (0 - 1)	0.4 (0.3 - 0.5)
United States Virgin Islands	Listeria monocytogenes	0 (0 - 0)	0.0 (0.0 - 0.1)
United States Virgin Islands	Morganella spp.	0 (0 - 0)	0.1 (0.1 - 0.2)

United States Virgin Islands	Mycoplasma spp.	1 (1 - 1)	0.6 (0.4 - 0.9)
United States Virgin Islands	Neisseria gonorrhoeae	0 (0 - 0)	0.0 (0.0 - 0.1)
United States Virgin Islands	Neisseria meningitidis	1 (0 - 1)	0.7 (0.4 - 1.2)
United States Virgin Islands	Non-typhoidal Salmonella	1 (0 - 1)	0.5 (0.3 - 0.8)
United States Virgin Islands	Other Klebsiella species	1 (1 - 2)	0.7 (0.4 - 1.3)
United States Virgin Islands	Other enterococci	3 (2 - 4)	1.8 (1.2 - 2.6)
United States Virgin Islands	Proteus spp.	3 (2 - 4)	1.9 (1.3 - 2.6)
United States Virgin Islands	Providencia spp.	0 (0 - 0)	0.1 (0.1 - 0.1)
United States Virgin Islands	Pseudomonas aeruginosa	14 (9 - 20)	8.3 (5.4 - 12.0)
United States Virgin Islands	Salmonella Paratyphi	0 (0 - 0)	0.0 (0.0 - 0.0)
United States Virgin Islands	Salmonella Typhi	0 (0 - 0)	0.2 (0.1 - 0.3)
United States Virgin Islands	Serratia spp.	2 (1 - 3)	1.2 (0.7 - 1.9)
United States Virgin Islands	Shigella spp.	0 (0 - 0)	0.0 (0.0 - 0.0)
United States Virgin Islands	Staphylococcus aureus	23 (16 - 33)	13.9 (9.5 - 19.7)
United States Virgin Islands	Streptococcus pneumoniae	11 (8 - 14)	6.8 (4.9 - 9.4)
United States Virgin Islands	Vibrio cholerae	0 (0 - 0)	0.1 (0.1 - 0.3)
Colombia	Acinetobacter baumannii	1,541 (831 - 2,544)	2.9 (1.6 - 4.8)
Colombia	Aeromonas spp.	3 (1 - 5)	0.0 (0.0 - 0.0)
Colombia	Campylobacter spp.	106 (30 - 236)	0.2 (0.1 - 0.4)
Colombia	Chlamydia spp.	205 (144 - 285)	0.4 (0.3 - 0.6)
Colombia	Citrobacter spp.	192 (121 - 290)	0.4 (0.2 - 0.6)
Colombia	Clostridioides difficile	128 (82 - 190)	0.3 (0.2 - 0.4)
Colombia	Enterobacter spp.	1,189 (748 - 1,809)	2.3 (1.4 - 3.5)
Colombia	Enterococcus faecalis	1,156 (687 - 1,872)	2.2 (1.3 - 3.6)
Colombia	Enterococcus faecium	1,241 (755 - 1,903)	2.4 (1.4 - 3.6)
Colombia	Escherichia coli	4,658 (3,099 - 6,687)	8.8 (5.8 - 12.6)
Colombia	Group A Streptococcus	1,227 (721 - 2,132)	2.4 (1.4 - 4.1)
Colombia	Group B Streptococcus	1,100 (690 - 1,658)	2.2 (1.4 - 3.3)
Colombia	Haemophilus influenzae	283 (194 - 396)	0.6 (0.4 - 0.8)
Colombia	Klebsiella pneumoniae	3,475 (2,246 - 5,146)	6.7 (4.3 - 9.8)
Colombia	Legionella spp.	256 (178 - 355)	0.5 (0.3 - 0.7)
Colombia	Listeria monocytogenes	32 (20 - 52)	0.1 (0.0 - 0.1)
Colombia	Morganella spp.	37 (25 - 54)	0.1 (0.0 - 0.1)
Colombia	Mycoplasma spp.	272 (192 - 370)	0.6 (0.4 - 0.8)
Colombia	Neisseria gonorrhoeae	12 (9 - 16)	0.0 (0.0 - 0.0)
Colombia	Neisseria meningitidis	291 (162 - 472)	0.6 (0.4 - 1.0)
Colombia	Non-typhoidal Salmonella	139 (76 - 236)	0.3 (0.2 - 0.5)
Colombia	Other Klebsiella species	261 (140 - 436)	0.5 (0.3 - 0.8)
Colombia	Other enterococci	551 (363 - 793)	1.0 (0.7 - 1.5)
Colombia	Proteus spp.	628 (424 - 883)	1.2 (0.8 - 1.6)

Colombia	Providencia spp.	24 (16 - 36)	0.0 (0.0 - 0.1)
Colombia	Pseudomonas aeruginosa	2,855 (1,814 - 4,272)	5.4 (3.4 - 8.2)
Colombia	Salmonella Paratyphi	1 (0 - 2)	0.0 (0.0 - 0.0)
Colombia	Salmonella Typhi	74 (41 - 119)	0.2 (0.1 - 0.3)
Colombia	Serratia spp.	375 (217 - 598)	0.7 (0.4 - 1.2)
Colombia	Shigella spp.	126 (61 - 233)	0.3 (0.1 - 0.5)
Colombia	Staphylococcus aureus	5,890 (3,917 - 8,565)	11.2 (7.4 - 16.2)
Colombia	Streptococcus pneumoniae	2,541 (1,764 - 3,543)	5.0 (3.5 - 7.0)
Colombia	Vibrio cholerae	5 (3 - 8)	0.0 (0.0 - 0.0)
Costa Rica	Acinetobacter baumannii	149 (80 - 245)	2.9 (1.6 - 4.8)
Costa Rica	Aeromonas spp.	0 (0 - 0)	0.0 (0.0 - 0.0)
Costa Rica	Campylobacter spp.	15 (3 - 37)	0.3 (0.1 - 0.7)
Costa Rica	Chlamydia spp.	17 (12 - 24)	0.3 (0.2 - 0.5)
Costa Rica	Citrobacter spp.	20 (12 - 31)	0.4 (0.2 - 0.6)
Costa Rica	Clostridioides difficile	18 (12 - 27)	0.4 (0.3 - 0.6)
Costa Rica	Enterobacter spp.	123 (76 - 187)	2.4 (1.5 - 3.7)
Costa Rica	Enterococcus faecalis	136 (77 - 225)	2.7 (1.5 - 4.4)
Costa Rica	Enterococcus faecium	156 (93 - 238)	3.0 (1.8 - 4.6)
Costa Rica	Escherichia coli	512 (329 - 750)	10.1 (6.5 - 14.8)
Costa Rica	Group A Streptococcus	100 (53 - 180)	2.0 (1.1 - 3.6)
Costa Rica	Group B Streptococcus	102 (62 - 157)	2.1 (1.2 - 3.1)
Costa Rica	Haemophilus influenzae	23 (16 - 32)	0.5 (0.3 - 0.7)
Costa Rica	Klebsiella pneumoniae	354 (224 - 525)	7.0 (4.4 - 10.4)
Costa Rica	Legionella spp.	27 (19 - 38)	0.6 (0.4 - 0.8)
Costa Rica	Listeria monocytogenes	3 (2 - 5)	0.1 (0.0 - 0.1)
Costa Rica	Morganella spp.	3 (2 - 4)	0.1 (0.0 - 0.1)
Costa Rica	Mycoplasma spp.	23 (16 - 31)	0.5 (0.3 - 0.6)
Costa Rica	Neisseria gonorrhoeae	1 (1 - 1)	0.0 (0.0 - 0.0)
Costa Rica	Neisseria meningitidis	22 (12 - 36)	0.5 (0.2 - 0.8)
Costa Rica	Non-typhoidal Salmonella	12 (5 - 25)	0.3 (0.1 - 0.5)
Costa Rica	Other Klebsiella species	35 (19 - 59)	0.7 (0.4 - 1.2)
Costa Rica	Other enterococci	48 (31 - 71)	1.0 (0.6 - 1.4)
Costa Rica	Proteus spp.	64 (41 - 91)	1.2 (0.8 - 1.8)
Costa Rica	Providencia spp.	2 (1 - 3)	0.0 (0.0 - 0.0)
Costa Rica	Pseudomonas aeruginosa	297 (188 - 448)	5.9 (3.7 - 8.8)
Costa Rica	Salmonella Paratyphi	0 (0 - 0)	0.0 (0.0 - 0.0)
Costa Rica	Salmonella Typhi	5 (3 - 8)	0.1 (0.1 - 0.2)
Costa Rica	Serratia spp.	38 (22 - 60)	0.7 (0.4 - 1.2)
Costa Rica	Shigella spp.	7 (3 - 16)	0.1 (0.1 - 0.3)
Costa Rica	Staphylococcus aureus	643 (419 - 949)	12.6 (8.2 - 18.7)

Costa Rica	Streptococcus pneumoniae	215 (147 - 305)	4.3 (2.9 - 6.1)
Costa Rica	Vibrio cholerae	0 (0 - 0)	0.0 (0.0 - 0.0)
El Salvador	Acinetobacter baumannii	286 (154 - 470)	4.6 (2.5 - 7.6)
El Salvador	Aeromonas spp.	1 (0 - 1)	0.0 (0.0 - 0.0)
El Salvador	Campylobacter spp.	36 (10 - 90)	0.6 (0.2 - 1.4)
El Salvador	Chlamydia spp.	51 (37 - 68)	0.8 (0.6 - 1.1)
El Salvador	Citrobacter spp.	33 (19 - 51)	0.5 (0.3 - 0.9)
El Salvador	Clostridioides difficile	10 (4 - 19)	0.2 (0.1 - 0.3)
El Salvador	Enterobacter spp.	207 (126 - 315)	3.4 (2.0 - 5.1)
El Salvador	Enterococcus faecalis	188 (106 - 308)	3.1 (1.7 - 5.1)
El Salvador	Enterococcus faecium	200 (112 - 316)	3.3 (1.9 - 5.2)
El Salvador	Escherichia coli	771 (490 - 1,125)	12.4 (7.9 - 18.1)
El Salvador	Group A Streptococcus	167 (89 - 300)	2.7 (1.4 - 4.9)
El Salvador	Group B Streptococcus	193 (125 - 282)	3.1 (2.0 - 4.6)
El Salvador	Haemophilus influenzae	65 (47 - 88)	1.0 (0.7 - 1.4)
El Salvador	Klebsiella pneumoniae	657 (420 - 959)	10.6 (6.8 - 15.5)
El Salvador	Legionella spp.	49 (35 - 66)	0.8 (0.6 - 1.1)
El Salvador	Listeria monocytogenes	4 (3 - 7)	0.1 (0.0 - 0.1)
El Salvador	Morganella spp.	6 (4 - 9)	0.1 (0.1 - 0.1)
El Salvador	Mycoplasma spp.	57 (41 - 75)	0.9 (0.7 - 1.2)
El Salvador	Neisseria gonorrhoeae	1 (0 - 1)	0.0 (0.0 - 0.0)
El Salvador	Neisseria meningitidis	57 (31 - 94)	1.0 (0.5 - 1.6)
El Salvador	Non-typhoidal Salmonella	39 (18 - 76)	0.6 (0.3 - 1.2)
El Salvador	Other Klebsiella species	48 (25 - 85)	0.8 (0.4 - 1.4)
El Salvador	Other enterococci	81 (50 - 122)	1.3 (0.8 - 1.9)
El Salvador	Proteus spp.	101 (63 - 150)	1.6 (1.0 - 2.4)
El Salvador	Providencia spp.	5 (3 - 7)	0.1 (0.0 - 0.1)
El Salvador	Pseudomonas aeruginosa	519 (332 - 766)	8.3 (5.3 - 12.3)
El Salvador	Salmonella Paratyphi	0 (0 - 0)	0.0 (0.0 - 0.0)
El Salvador	Salmonella Typhi	18 (9 - 29)	0.3 (0.2 - 0.5)
El Salvador	Serratia spp.	68 (38 - 112)	1.1 (0.6 - 1.8)
El Salvador	Shigella spp.	23 (9 - 50)	0.4 (0.1 - 0.8)
El Salvador	Staphylococcus aureus	1,111 (769 - 1,552)	17.6 (12.1 - 24.9)
El Salvador	Streptococcus pneumoniae	576 (414 - 777)	9.2 (6.6 - 12.5)
El Salvador	Vibrio cholerae	3 (1 - 7)	0.1 (0.0 - 0.1)
Guatemala	Acinetobacter baumannii	757 (433 - 1,202)	7.2 (4.1 - 11.4)
Guatemala	Aeromonas spp.	6 (3 - 11)	0.0 (0.0 - 0.1)
Guatemala	Campylobacter spp.	160 (46 - 364)	1.4 (0.4 - 3.2)
Guatemala	Chlamydia spp.	212 (156 - 280)	1.8 (1.4 - 2.4)
Guatemala	Citrobacter spp.	95 (54 - 150)	0.8 (0.5 - 1.3)

Guatemala	Clostridioides difficile	16 (7 - 31)	0.1 (0.0 - 0.2)
Guatemala	Enterobacter spp.	603 (385 - 900)	5.3 (3.4 - 7.9)
Guatemala	Enterococcus faecalis	518 (292 - 833)	4.4 (2.5 - 7.3)
Guatemala	Enterococcus faecium	500 (286 - 795)	4.5 (2.6 - 7.1)
Guatemala	Escherichia coli	2,047 (1,364 - 2,946)	18.6 (12.5 - 26.3)
Guatemala	Group A Streptococcus	422 (214 - 788)	3.7 (1.8 - 7.1)
Guatemala	Group B Streptococcus	682 (470 - 949)	5.7 (4.0 - 7.8)
Guatemala	Haemophilus influenzae	304 (224 - 401)	2.4 (1.8 - 3.1)
Guatemala	Klebsiella pneumoniae	2,133 (1,467 - 2,976)	19.0 (13.3 - 26.4)
Guatemala	Legionella spp.	117 (82 - 165)	1.0 (0.7 - 1.4)
Guatemala	Listeria monocytogenes	16 (11 - 25)	0.1 (0.1 - 0.2)
Guatemala	Morganella spp.	11 (6 - 18)	0.1 (0.1 - 0.2)
Guatemala	Mycoplasma spp.	238 (175 - 313)	1.7 (1.3 - 2.2)
Guatemala	Neisseria gonorrhoeae	6 (5 - 8)	0.0 (0.0 - 0.1)
Guatemala	Neisseria meningitidis	264 (149 - 421)	1.7 (0.9 - 2.6)
Guatemala	Non-typhoidal Salmonella	275 (96 - 651)	2.0 (0.7 - 4.6)
Guatemala	Other Klebsiella species	168 (82 - 294)	1.4 (0.7 - 2.5)
Guatemala	Other enterococci	172 (104 - 265)	1.6 (1.0 - 2.5)
Guatemala	Proteus spp.	251 (154 - 384)	2.4 (1.5 - 3.6)
Guatemala	Providencia spp.	12 (6 - 19)	0.1 (0.1 - 0.2)
Guatemala	Pseudomonas aeruginosa	1,451 (974 - 2,042)	13.1 (9.0 - 18.5)
Guatemala	Salmonella Paratyphi	0 (0 - 0)	0.0 (0.0 - 0.0)
Guatemala	Salmonella Typhi	144 (88 - 228)	1.0 (0.6 - 1.5)
Guatemala	Serratia spp.	214 (122 - 343)	1.8 (1.0 - 2.9)
Guatemala	Shigella spp.	406 (180 - 738)	2.7 (1.2 - 5.0)
Guatemala	Staphylococcus aureus	3,063 (2,202 - 4,161)	28.3 (21.0 - 37.9)
Guatemala	Streptococcus pneumoniae	2,536 (1,914 - 3,316)	20.3 (15.7 - 26.0)
Guatemala	Vibrio cholerae	11 (6 - 18)	0.1 (0.0 - 0.1)
Honduras	Acinetobacter baumannii	470 (260 - 754)	8.6 (4.8 - 13.9)
Honduras	Aeromonas spp.	2 (1 - 5)	0.0 (0.0 - 0.1)
Honduras	Campylobacter spp.	98 (30 - 230)	1.5 (0.4 - 3.9)
Honduras	Chlamydia spp.	52 (35 - 76)	0.8 (0.5 - 1.1)
Honduras	Citrobacter spp.	63 (39 - 95)	1.1 (0.7 - 1.6)
Honduras	Clostridioides difficile	13 (5 - 26)	0.1 (0.0 - 0.2)
Honduras	Enterobacter spp.	338 (217 - 494)	5.8 (3.7 - 8.3)
Honduras	Enterococcus faecalis	351 (206 - 567)	5.9 (3.4 - 9.6)
Honduras	Enterococcus faecium	367 (224 - 558)	6.3 (3.9 - 9.6)
Honduras	Escherichia coli	1,237 (817 - 1,778)	21.8 (14.8 - 31.0)
Honduras	Group A Streptococcus	214 (124 - 369)	3.7 (2.1 - 6.4)
Honduras	Group B Streptococcus	251 (167 - 358)	3.8 (2.6 - 5.5)
Honduras	Haemophilus influenzae	68 (46 - 95)	1.0 (0.7 - 1.4)

Honduras	Klebsiella pneumoniae	1,093 (723 - 1,552)	18.8 (12.4 - 26.5)
Honduras	Legionella spp.	36 (22 - 60)	0.5 (0.3 - 0.8)
Honduras	Listeria monocytogenes	9 (5 - 13)	0.1 (0.1 - 0.2)
Honduras	Morganella spp.	8 (4 - 13)	0.2 (0.1 - 0.3)
Honduras	Mycoplasma spp.	43 (29 - 61)	0.6 (0.4 - 0.8)
Honduras	Neisseria gonorrhoeae	3 (2 - 4)	0.0 (0.0 - 0.1)
Honduras	Neisseria meningitidis	117 (69 - 184)	1.4 (0.8 - 2.3)
Honduras	Non-typhoidal Salmonella	120 (58 - 241)	1.6 (0.8 - 3.4)
Honduras	Other Klebsiella species	120 (58 - 208)	2.0 (1.0 - 3.8)
Honduras	Other enterococci	108 (70 - 158)	2.0 (1.3 - 2.9)
Honduras	Proteus spp.	180 (116 - 264)	3.3 (2.2 - 4.9)
Honduras	Providencia spp.	9 (5 - 15)	0.2 (0.1 - 0.3)
Honduras	Pseudomonas aeruginosa	721 (470 - 1,043)	12.5 (8.2 - 17.8)
Honduras	Salmonella Paratyphi	0 (0 - 0)	0.0 (0.0 - 0.0)
Honduras	Salmonella Typhi	67 (40 - 103)	0.9 (0.5 - 1.3)
Honduras	Serratia spp.	139 (87 - 212)	2.3 (1.4 - 3.4)
Honduras	Shigella spp.	73 (27 - 160)	0.9 (0.3 - 1.9)
Honduras	Staphylococcus aureus	1,241 (855 - 1,746)	21.5 (15.1 - 29.8)
Honduras	Streptococcus pneumoniae	560 (391 - 782)	8.9 (6.3 - 12.2)
Honduras	Vibrio cholerae	15 (7 - 31)	0.2 (0.1 - 0.4)
Mexico	Acinetobacter baumannii	5,684 (3,186 - 8,908)	5.1 (2.9 - 8.0)
Mexico	Aeromonas spp.	10 (4 - 19)	0.0 (0.0 - 0.0)
Mexico	Campylobacter spp.	591 (171 - 1,256)	0.6 (0.2 - 1.2)
Mexico	Chlamydia spp.	692 (526 - 924)	0.6 (0.5 - 0.8)
Mexico	Citrobacter spp.	892 (562 - 1,318)	0.8 (0.5 - 1.2)
Mexico	Clostridioides difficile	375 (236 - 573)	0.3 (0.2 - 0.5)
Mexico	Enterobacter spp.	5,041 (3,233 - 7,304)	4.5 (2.9 - 6.5)
Mexico	Enterococcus faecalis	4,886 (2,965 - 7,444)	4.3 (2.6 - 6.5)
Mexico	Enterococcus faecium	5,195 (3,165 - 7,752)	4.6 (2.8 - 6.8)
Mexico	Escherichia coli	17,029 (11,596 - 24,102)	15.3 (10.5 - 21.5)
Mexico	Group A Streptococcus	3,695 (2,025 - 6,416)	3.3 (1.8 - 5.8)
Mexico	Group B Streptococcus	3,647 (2,457 - 5,307)	3.3 (2.2 - 4.8)
Mexico	Haemophilus influenzae	884 (659 - 1,167)	0.8 (0.6 - 1.1)
Mexico	Klebsiella pneumoniae	12,873 (8,516 - 18,541)	11.6 (7.8 - 16.7)
Mexico	Legionella spp.	530 (391 - 715)	0.5 (0.4 - 0.7)
Mexico	Listeria monocytogenes	51 (31 - 93)	0.0 (0.0 - 0.1)
Mexico	Morganella spp.	136 (89 - 201)	0.1 (0.1 - 0.2)
Mexico	Mycoplasma spp.	828 (648 - 1,046)	0.7 (0.6 - 0.9)
Mexico	Neisseria gonorrhoeae	60 (48 - 74)	0.0 (0.0 - 0.1)
Mexico	Neisseria meningitidis	1,208 (693 - 1,912)	1.0 (0.6 - 1.6)

Mexico	Non-typhoidal Salmonella	992 (444 - 1,889)	0.9 (0.4 - 1.7)
Mexico	Other Klebsiella species	1,409 (786 - 2,335)	1.2 (0.7 - 2.0)
Mexico	Other enterococci	1,814 (1,214 - 2,615)	1.7 (1.1 - 2.4)
Mexico	Proteus spp.	2,394 (1,563 - 3,405)	2.2 (1.4 - 3.1)
Mexico	Providencia spp.	108 (67 - 170)	0.1 (0.1 - 0.2)
Mexico	Pseudomonas aeruginosa	10,037 (6,531 - 14,474)	9.1 (5.9 - 13.0)
Mexico	Salmonella Paratyphi	1 (1 - 2)	0.0 (0.0 - 0.0)
Mexico	Salmonella Typhi	386 (225 - 697)	0.3 (0.2 - 0.6)
Mexico	Serratia spp.	1,656 (1,010 - 2,561)	1.5 (0.9 - 2.3)
Mexico	Shigella spp.	231 (96 - 458)	0.2 (0.1 - 0.4)
Mexico	Staphylococcus aureus	17,934 (12,258 - 25,546)	16.1 (11.1 - 22.9)
Mexico	Streptococcus pneumoniae	8,417 (6,326 - 11,182)	7.6 (5.7 - 10.1)
Mexico	Vibrio cholerae	20 (12 - 34)	0.0 (0.0 - 0.0)
Nicaragua	Acinetobacter baumannii	191 (110 - 306)	4.8 (2.8 - 7.6)
Nicaragua	Aeromonas spp.	0 (0 - 1)	0.0 (0.0 - 0.0)
Nicaragua	Campylobacter spp.	23 (8 - 51)	0.5 (0.2 - 1.2)
Nicaragua	Chlamydia spp.	25 (18 - 33)	0.6 (0.4 - 0.8)
Nicaragua	Citrobacter spp.	27 (17 - 41)	0.6 (0.4 - 0.9)
Nicaragua	Clostridioides difficile	4 (2 - 7)	0.1 (0.0 - 0.1)
Nicaragua	Enterobacter spp.	154 (100 - 224)	3.6 (2.4 - 5.2)
Nicaragua	Enterococcus faecalis	168 (100 - 258)	3.9 (2.3 - 6.1)
Nicaragua	Enterococcus faecium	189 (114 - 293)	4.5 (2.7 - 6.9)
Nicaragua	Escherichia coli	609 (397 - 885)	15.0 (9.7 - 21.7)
Nicaragua	Group A Streptococcus	142 (78 - 255)	3.4 (1.8 - 6.1)
Nicaragua	Group B Streptococcus	137 (90 - 201)	3.1 (2.0 - 4.6)
Nicaragua	Haemophilus influenzae	38 (30 - 49)	0.8 (0.6 - 1.1)
Nicaragua	Klebsiella pneumoniae	508 (337 - 737)	12.2 (8.0 - 17.8)
Nicaragua	Legionella spp.	30 (22 - 43)	0.7 (0.5 - 1.0)
Nicaragua	Listeria monocytogenes	4 (3 - 7)	0.1 (0.0 - 0.1)
Nicaragua	Morganella spp.	3 (2 - 5)	0.1 (0.0 - 0.1)
Nicaragua	Mycoplasma spp.	34 (27 - 43)	0.7 (0.5 - 0.9)
Nicaragua	Neisseria gonorrhoeae	1 (0 - 1)	0.0 (0.0 - 0.0)
Nicaragua	Neisseria meningitidis	53 (31 - 83)	0.9 (0.6 - 1.5)
Nicaragua	Non-typhoidal Salmonella	32 (18 - 55)	0.6 (0.3 - 1.1)
Nicaragua	Other Klebsiella species	45 (24 - 77)	1.0 (0.6 - 1.8)
Nicaragua	Other enterococci	58 (37 - 88)	1.5 (0.9 - 2.2)
Nicaragua	Proteus spp.	78 (49 - 116)	2.0 (1.3 - 2.9)
Nicaragua	Providencia spp.	2 (1 - 4)	0.1 (0.0 - 0.1)
Nicaragua	Pseudomonas aeruginosa	378 (252 - 542)	9.1 (6.1 - 13.2)
Nicaragua	Salmonella Paratyphi	0 (0 - 0)	0.0 (0.0 - 0.0)

Nicaragua	Salmonella Typhi	18 (11 - 29)	0.3 (0.2 - 0.5)
Nicaragua	Serratia spp.	61 (36 - 95)	1.4 (0.8 - 2.2)
Nicaragua	Shigella spp.	10 (3 - 21)	0.2 (0.1 - 0.4)
Nicaragua	Staphylococcus aureus	767 (538 - 1,070)	18.7 (13.1 - 26.2)
Nicaragua	Streptococcus pneumoniae	345 (264 - 457)	7.6 (5.8 - 10.1)
Nicaragua	Vibrio cholerae	22 (11 - 42)	0.4 (0.2 - 0.9)
Panama	Acinetobacter baumannii	136 (74 - 222)	3.2 (1.8 - 5.3)
Panama	Aeromonas spp.	0 (0 - 1)	0.0 (0.0 - 0.0)
Panama	Campylobacter spp.	18 (6 - 40)	0.4 (0.1 - 1.0)
Panama	Chlamydia spp.	20 (14 - 27)	0.5 (0.3 - 0.6)
Panama	Citrobacter spp.	16 (10 - 25)	0.4 (0.2 - 0.6)
Panama	Clostridioides difficile	18 (11 - 29)	0.5 (0.3 - 0.7)
Panama	Enterobacter spp.	104 (64 - 161)	2.5 (1.6 - 3.9)
Panama	Enterococcus faecalis	90 (51 - 147)	2.2 (1.2 - 3.5)
Panama	Enterococcus faecium	99 (59 - 154)	2.4 (1.4 - 3.7)
Panama	Escherichia coli	357 (230 - 521)	8.5 (5.5 - 12.4)
Panama	Group A Streptococcus	102 (56 - 181)	2.4 (1.3 - 4.3)
Panama	Group B Streptococcus	99 (63 - 147)	2.4 (1.5 - 3.5)
Panama	Haemophilus influenzae	28 (20 - 39)	0.7 (0.5 - 0.9)
Panama	Klebsiella pneumoniae	290 (186 - 428)	7.0 (4.5 - 10.2)
Panama	Legionella spp.	20 (14 - 28)	0.5 (0.3 - 0.7)
Panama	Listeria monocytogenes	3 (2 - 5)	0.1 (0.0 - 0.1)
Panama	Morganella spp.	2 (1 - 4)	0.1 (0.0 - 0.1)
Panama	Mycoplasma spp.	27 (20 - 36)	0.7 (0.5 - 0.9)
Panama	Neisseria gonorrhoeae	1 (1 - 1)	0.0 (0.0 - 0.0)
Panama	Neisseria meningitidis	29 (17 - 47)	0.7 (0.4 - 1.1)
Panama	Non-typhoidal Salmonella	20 (9 - 43)	0.5 (0.2 - 1.1)
Panama	Other Klebsiella species	21 (11 - 35)	0.5 (0.3 - 0.8)
Panama	Other enterococci	43 (27 - 64)	1.0 (0.6 - 1.5)
Panama	Proteus spp.	47 (30 - 69)	1.1 (0.7 - 1.6)
Panama	Providencia spp.	2 (1 - 3)	0.0 (0.0 - 0.1)
Panama	Pseudomonas aeruginosa	240 (151 - 360)	5.7 (3.6 - 8.6)
Panama	Salmonella Paratyphi	0 (0 - 0)	0.0 (0.0 - 0.0)
Panama	Salmonella Typhi	6 (4 - 10)	0.2 (0.1 - 0.3)
Panama	Serratia spp.	33 (19 - 53)	0.8 (0.5 - 1.3)
Panama	Shigella spp.	16 (7 - 32)	0.4 (0.2 - 0.8)
Panama	Staphylococcus aureus	487 (324 - 707)	11.6 (7.7 - 16.8)
Panama	Streptococcus pneumoniae	251 (180 - 345)	6.0 (4.3 - 8.3)
Panama	Vibrio cholerae	5 (3 - 8)	0.1 (0.1 - 0.2)
Venezuela (Bolivarian Republic of)	Acinetobacter baumannii	1,624 (853 - 2,676)	5.9 (3.2 - 9.8)

Venezuela (Bolivarian Republic of)	Aeromonas spp.	3 (1 - 5)	0.0 (0.0 - 0.0)
Venezuela (Bolivarian Republic of)	Campylobacter spp.	220 (69 - 486)	0.8 (0.3 - 1.8)
Venezuela (Bolivarian Republic of)	Chlamydia spp.	214 (140 - 308)	0.8 (0.5 - 1.2)
Venezuela (Bolivarian Republic of)	Citrobacter spp.	202 (114 - 325)	0.7 (0.4 - 1.2)
Venezuela (Bolivarian Republic of)	Clostridioides difficile	157 (93 - 243)	0.6 (0.4 - 0.9)
Venezuela (Bolivarian Republic of)	Enterobacter spp.	1,311 (764 - 2,027)	4.8 (2.8 - 7.4)
Venezuela (Bolivarian Republic of)	Enterococcus faecalis	712 (396 - 1,172)	2.5 (1.4 - 4.2)
Venezuela (Bolivarian Republic of)	Enterococcus faecium	773 (453 - 1,201)	2.7 (1.6 - 4.2)
Venezuela (Bolivarian Republic of)	Escherichia coli	2,436 (1,530 - 3,646)	8.9 (5.6 - 13.2)
Venezuela (Bolivarian Republic of)	Group A Streptococcus	538 (220 - 1,084)	2.0 (0.8 - 3.9)
Venezuela (Bolivarian Republic of)	Group B Streptococcus	783 (476 - 1,204)	2.9 (1.8 - 4.5)
Venezuela (Bolivarian Republic of)	Haemophilus influenzae	211 (142 - 300)	0.8 (0.5 - 1.1)
Venezuela (Bolivarian Republic of)	Klebsiella pneumoniae	2,042 (1,258 - 3,108)	7.5 (4.6 - 11.4)
Venezuela (Bolivarian Republic of)	Legionella spp.	97 (61 - 154)	0.4 (0.2 - 0.6)
Venezuela (Bolivarian Republic of)	Listeria monocytogenes	20 (12 - 33)	0.1 (0.0 - 0.1)
Venezuela (Bolivarian Republic of)	Morganella spp.	19 (11 - 32)	0.1 (0.0 - 0.1)
Venezuela (Bolivarian Republic of)	Mycoplasma spp.	257 (180 - 361)	1.0 (0.7 - 1.4)
Venezuela (Bolivarian Republic of)	Neisseria gonorrhoeae	6 (5 - 9)	0.0 (0.0 - 0.0)
Venezuela (Bolivarian Republic of)	Neisseria meningitidis	284 (156 - 466)	1.0 (0.6 - 1.7)
Venezuela (Bolivarian Republic of)	Non-typhoidal Salmonella	229 (117 - 407)	0.9 (0.4 - 1.5)
Venezuela (Bolivarian Republic of)	Other Klebsiella species	188 (97 - 324)	0.7 (0.3 - 1.1)
Venezuela (Bolivarian Republic of)	Other enterococci	340 (196 - 535)	1.3 (0.7 - 2.0)
Venezuela (Bolivarian Republic of)	Proteus spp.	349 (211 - 535)	1.3 (0.8 - 1.9)
Venezuela (Bolivarian Republic of)	Providencia spp.	15 (8 - 26)	0.1 (0.0 - 0.1)
Venezuela (Bolivarian Republic of)	Pseudomonas aeruginosa	1,728 (1,038 - 2,688)	6.3 (3.8 - 9.8)
Venezuela (Bolivarian Republic of)	Salmonella Paratyphi	0 (0 - 0)	0.0 (0.0 - 0.0)
Venezuela (Bolivarian Republic of)	Salmonella Typhi	42 (22 - 72)	0.2 (0.1 - 0.3)
Venezuela (Bolivarian Republic of)	Serratia spp.	341 (187 - 553)	1.2 (0.7 - 2.0)
Venezuela (Bolivarian Republic of)	Shigella spp.	102 (41 - 200)	0.4 (0.2 - 0.8)
Venezuela (Bolivarian Republic of)	Staphylococcus aureus	2,763 (1,729 - 4,182)	10.0 (6.3 - 15.1)
Venezuela (Bolivarian Republic of)	Streptococcus pneumoniae	2,023 (1,400 - 2,898)	7.5 (5.2 - 10.7)
Venezuela (Bolivarian Republic of)	Vibrio cholerae	7 (4 - 13)	0.0 (0.0 - 0.0)
Brazil	Acinetobacter baumannii	10,691 (6,297 - 16,675)	4.8 (2.9 - 7.5)
Brazil	Aeromonas spp.	84 (43 - 152)	0.0 (0.0 - 0.1)
Brazil	Campylobacter spp.	556 (178 - 1,181)	0.3 (0.1 - 0.6)
Brazil	Chlamydia spp.	1,993 (1,644 - 2,436)	0.9 (0.8 - 1.1)
Brazil	Citrobacter spp.	1,322 (924 - 1,859)	0.6 (0.4 - 0.8)
Brazil	Clostridioides difficile	499 (340 - 748)	0.2 (0.2 - 0.4)
Brazil	Enterobacter spp.	7,967 (5,679 - 10,926)	3.6 (2.6 - 5.0)
Brazil	Enterococcus faecalis	7,294 (4,729 - 10,548)	3.3 (2.1 - 4.7)
Brazil	Enterococcus faecium	7,253 (4,659 - 10,633)	3.2 (2.1 - 4.7)

Brazil	Escherichia coli	31,505 (23,717 - 40,846)	14.3 (10.8 - 18.5)
Brazil	Group A Streptococcus	7,020 (4,280 - 11,420)	3.3 (2.0 - 5.2)
Brazil	Group B Streptococcus	7,539 (5,572 - 10,255)	3.6 (2.6 - 4.8)
Brazil	Haemophilus influenzae	2,560 (2,139 - 3,100)	1.2 (1.0 - 1.5)
Brazil	Klebsiella pneumoniae	25,169 (18,489 - 33,857)	11.6 (8.6 - 15.6)
Brazil	Legionella spp.	1,882 (1,549 - 2,306)	0.9 (0.7 - 1.1)
Brazil	Listeria monocytogenes	153 (105 - 232)	0.1 (0.1 - 0.1)
Brazil	Morganella spp.	352 (248 - 460)	0.2 (0.1 - 0.2)
Brazil	Mycoplasma spp.	2,144 (1,833 - 2,550)	1.0 (0.9 - 1.2)
Brazil	Neisseria gonorrhoeae	92 (85 - 98)	0.0 (0.0 - 0.0)
Brazil	Neisseria meningitidis	2,145 (1,348 - 3,239)	1.1 (0.7 - 1.7)
Brazil	Non-typhoidal Salmonella	1,052 (667 - 1,631)	0.5 (0.3 - 0.8)
Brazil	Other Klebsiella species	1,718 (939 - 2,879)	0.7 (0.4 - 1.3)
Brazil	Other enterococci	3,831 (2,794 - 5,068)	1.8 (1.3 - 2.3)
Brazil	Proteus spp.	4,397 (3,264 - 5,771)	2.0 (1.5 - 2.6)
Brazil	Providencia spp.	267 (191 - 362)	0.1 (0.1 - 0.2)
Brazil	Pseudomonas aeruginosa	19,762 (14,346 - 26,848)	9.1 (6.6 - 12.3)
Brazil	Salmonella Paratyphi	1 (0 - 1)	0.0 (0.0 - 0.0)
Brazil	Salmonella Typhi	655 (400 - 1,005)	0.3 (0.2 - 0.5)
Brazil	Serratia spp.	2,500 (1,578 - 3,775)	1.1 (0.7 - 1.7)
Brazil	Shigella spp.	726 (314 - 1,345)	0.4 (0.2 - 0.7)
Brazil	Staphylococcus aureus	41,622 (32,690 - 52,710)	19.0 (15.0 - 24.0)
Brazil	Streptococcus pneumoniae	21,842 (18,315 - 26,459)	10.3 (8.6 - 12.5)
Brazil	Vibrio cholerae	113 (40 - 220)	0.1 (0.0 - 0.1)
Paraguay	Acinetobacter baumannii	261 (141 - 429)	4.8 (2.6 - 7.9)
Paraguay	Aeromonas spp.	1 (1 - 3)	0.0 (0.0 - 0.1)
Paraguay	Campylobacter spp.	12 (3 - 28)	0.2 (0.1 - 0.5)
Paraguay	Chlamydia spp.	36 (25 - 49)	0.7 (0.5 - 0.9)
Paraguay	Citrobacter spp.	30 (18 - 46)	0.5 (0.3 - 0.8)
Paraguay	Clostridioides difficile	35 (19 - 59)	0.6 (0.3 - 1.0)
Paraguay	Enterobacter spp.	173 (108 - 260)	3.2 (2.0 - 4.7)
Paraguay	Enterococcus faecalis	164 (97 - 266)	3.0 (1.8 - 4.8)
Paraguay	Enterococcus faecium	173 (101 - 264)	3.1 (1.9 - 4.8)
Paraguay	Escherichia coli	653 (428 - 948)	12.2 (8.1 - 17.6)
Paraguay	Group A Streptococcus	170 (92 - 298)	3.1 (1.6 - 5.4)
Paraguay	Group B Streptococcus	164 (105 - 245)	2.9 (1.9 - 4.4)
Paraguay	Haemophilus influenzae	50 (35 - 69)	0.9 (0.6 - 1.2)
Paraguay	Klebsiella pneumoniae	583 (377 - 856)	10.7 (7.0 - 15.6)
Paraguay	Legionella spp.	34 (24 - 46)	0.6 (0.4 - 0.8)
Paraguay	Listeria monocytogenes	5 (3 - 8)	0.1 (0.0 - 0.1)
Paraguay	Morganella spp.	5 (3 - 8)	0.1 (0.1 - 0.2)

Paraguay	Mycoplasma spp.	40 (29 - 54)	0.7 (0.5 - 0.9)
Paraguay	Neisseria gonorrhoeae	1 (1 - 1)	0.0 (0.0 - 0.0)
Paraguay	Neisseria meningitidis	73 (43 - 116)	1.2 (0.7 - 1.9)
Paraguay	Non-typhoidal Salmonella	33 (20 - 52)	0.6 (0.3 - 0.9)
Paraguay	Other Klebsiella species	39 (20 - 68)	0.7 (0.4 - 1.3)
Paraguay	Other enterococci	76 (48 - 113)	1.4 (0.9 - 2.1)
Paraguay	Proteus spp.	90 (59 - 132)	1.7 (1.1 - 2.5)
Paraguay	Providencia spp.	4 (3 - 7)	0.1 (0.0 - 0.1)
Paraguay	Pseudomonas aeruginosa	435 (278 - 648)	8.0 (5.2 - 11.9)
Paraguay	Salmonella Paratyphi	0 (0 - 0)	0.0 (0.0 - 0.0)
Paraguay	Salmonella Typhi	26 (15 - 41)	0.4 (0.2 - 0.7)
Paraguay	Serratia spp.	66 (39 - 106)	1.2 (0.7 - 1.9)
Paraguay	Shigella spp.	19 (7 - 41)	0.3 (0.1 - 0.7)
Paraguay	Staphylococcus aureus	880 (599 - 1,262)	16.2 (11.1 - 23.2)
Paraguay	Streptococcus pneumoniae	450 (317 - 618)	8.0 (5.7 - 11.0)
Paraguay	Vibrio cholerae	3 (1 - 7)	0.1 (0.0 - 0.1)
Afghanistan	Acinetobacter baumannii	2,206 (1,234 - 3,395)	18.6 (10.3 - 29.4)
Afghanistan	Aeromonas spp.	79 (25 - 195)	0.2 (0.1 - 0.3)
Afghanistan	Campylobacter spp.	109 (33 - 245)	0.3 (0.1 - 0.8)
Afghanistan	Chlamydia spp.	748 (504 - 1,072)	2.2 (1.6 - 3.1)
Afghanistan	Citrobacter spp.	238 (137 - 380)	1.3 (0.7 - 2.1)
Afghanistan	Clostridioides difficile	15 (5 - 39)	0.0 (0.0 - 0.1)
Afghanistan	Enterobacter spp.	1,390 (944 - 2,004)	7.8 (4.9 - 11.4)
Afghanistan	Enterococcus faecalis	964 (577 - 1,534)	6.1 (3.4 - 10.1)
Afghanistan	Enterococcus faecium	707 (406 - 1,169)	5.3 (3.0 - 9.0)
Afghanistan	Escherichia coli	5,058 (3,658 - 6,813)	25.7 (17.5 - 35.6)
Afghanistan	Group A Streptococcus	1,266 (690 - 2,182)	9.0 (4.2 - 17.3)
Afghanistan	Group B Streptococcus	3,856 (2,649 - 5,235)	11.4 (7.7 - 16.0)
Afghanistan	Haemophilus influenzae	1,294 (920 - 1,742)	3.3 (2.4 - 4.4)
Afghanistan	Klebsiella pneumoniae	6,904 (5,018 - 9,284)	32.0 (22.3 - 44.2)
Afghanistan	Legionella spp.	166 (84 - 310)	0.5 (0.3 - 0.7)
Afghanistan	Listeria monocytogenes	207 (96 - 406)	0.7 (0.3 - 1.3)
Afghanistan	Morganella spp.	17 (7 - 34)	0.2 (0.1 - 0.3)
Afghanistan	Mycoplasma spp.	570 (418 - 762)	1.3 (1.0 - 1.6)
Afghanistan	Neisseria gonorrhoeae	5 (3 - 7)	0.0 (0.0 - 0.1)
Afghanistan	Neisseria meningitidis	2,244 (1,326 - 3,450)	5.8 (3.5 - 8.8)
Afghanistan	Non-typhoidal Salmonella	2,054 (1,340 - 3,025)	6.5 (4.4 - 9.7)
Afghanistan	Other Klebsiella species	225 (92 - 457)	1.8 (0.7 - 3.6)
Afghanistan	Other enterococci	409 (233 - 664)	2.8 (1.5 - 4.7)
Afghanistan	Proteus spp.	477 (283 - 759)	4.2 (2.5 - 6.7)

Afghanistan	Providencia spp.	34 (12 - 67)	0.3 (0.1 - 0.6)
Afghanistan	Pseudomonas aeruginosa	3,337 (2,384 - 4,514)	17.4 (11.7 - 24.8)
Afghanistan	Salmonella Paratyphi	4 (1 - 8)	0.0 (0.0 - 0.0)
Afghanistan	Salmonella Typhi	2,033 (1,242 - 3,048)	5.5 (3.4 - 8.2)
Afghanistan	Serratia spp.	737 (439 - 1,179)	4.0 (2.3 - 6.4)
Afghanistan	Shigella spp.	432 (135 - 1,002)	0.9 (0.3 - 1.9)
Afghanistan	Staphylococcus aureus	5,557 (4,183 - 7,336)	28.5 (20.5 - 39.0)
Afghanistan	Streptococcus pneumoniae	8,591 (6,552 - 11,158)	25.6 (19.5 - 33.0)
Afghanistan	Vibrio cholerae	701 (303 - 1,323)	3.6 (1.4 - 7.0)
Algeria	Acinetobacter baumannii	1,121 (620 - 1,841)	4.1 (2.3 - 6.8)
Algeria	Aeromonas spp.	7 (3 - 14)	0.0 (0.0 - 0.0)
Algeria	Campylobacter spp.	16 (4 - 41)	0.1 (0.0 - 0.2)
Algeria	Chlamydia spp.	186 (131 - 263)	0.6 (0.5 - 0.9)
Algeria	Citrobacter spp.	117 (68 - 186)	0.4 (0.2 - 0.6)
Algeria	Clostridioides difficile	41 (21 - 76)	0.1 (0.1 - 0.2)
Algeria	Enterobacter spp.	762 (468 - 1,192)	2.6 (1.7 - 4.0)
Algeria	Enterococcus faecalis	631 (356 - 1,042)	2.2 (1.2 - 3.7)
Algeria	Enterococcus faecium	744 (432 - 1,228)	2.6 (1.5 - 4.2)
Algeria	Escherichia coli	2,627 (1,646 - 4,016)	9.6 (6.1 - 14.6)
Algeria	Group A Streptococcus	656 (311 - 1,243)	2.2 (1.0 - 4.4)
Algeria	Group B Streptococcus	830 (511 - 1,272)	2.7 (1.7 - 4.1)
Algeria	Haemophilus influenzae	250 (181 - 343)	0.8 (0.6 - 1.1)
Algeria	Klebsiella pneumoniae	2,449 (1,563 - 3,698)	8.7 (5.7 - 13.1)
Algeria	Legionella spp.	231 (151 - 364)	0.8 (0.5 - 1.1)
Algeria	Listeria monocytogenes	28 (18 - 47)	0.1 (0.1 - 0.1)
Algeria	Morganella spp.	13 (5 - 24)	0.1 (0.0 - 0.1)
Algeria	Mycoplasma spp.	241 (177 - 324)	0.7 (0.5 - 1.0)
Algeria	Neisseria gonorrhoeae	3 (2 - 4)	0.0 (0.0 - 0.0)
Algeria	Neisseria meningitidis	300 (170 - 496)	0.8 (0.4 - 1.3)
Algeria	Non-typhoidal Salmonella	167 (106 - 252)	0.5 (0.3 - 0.8)
Algeria	Other Klebsiella species	139 (71 - 247)	0.5 (0.2 - 0.9)
Algeria	Other enterococci	283 (160 - 463)	1.0 (0.6 - 1.7)
Algeria	Proteus spp.	316 (186 - 502)	1.2 (0.7 - 1.9)
Algeria	Providencia spp.	11 (4 - 20)	0.0 (0.0 - 0.1)
Algeria	Pseudomonas aeruginosa	1,960 (1,235 - 2,962)	7.1 (4.5 - 10.7)
Algeria	Salmonella Paratyphi	1 (0 - 3)	0.0 (0.0 - 0.0)
Algeria	Salmonella Typhi	177 (101 - 289)	0.5 (0.3 - 0.7)
Algeria	Serratia spp.	275 (158 - 454)	0.9 (0.5 - 1.5)
Algeria	Shigella spp.	42 (16 - 90)	0.1 (0.1 - 0.3)
Algeria	Staphylococcus aureus	4,030 (2,726 - 5,885)	14.9 (10.2 - 21.3)

Algeria	Streptococcus pneumoniae	2,067 (1,508 - 2,828)	7.0 (5.2 - 9.4)
Algeria	Vibrio cholerae	35 (15 - 77)	0.1 (0.0 - 0.3)
Bahrain	Acinetobacter baumannii	26 (14 - 45)	4.7 (2.5 - 7.9)
Bahrain	Aeromonas spp.	0 (0 - 0)	0.0 (0.0 - 0.1)
Bahrain	Campylobacter spp.	0 (0 - 1)	0.1 (0.0 - 0.3)
Bahrain	Chlamydia spp.	2 (2 - 3)	0.5 (0.4 - 0.6)
Bahrain	Citrobacter spp.	3 (2 - 5)	0.5 (0.3 - 0.8)
Bahrain	Clostridioides difficile	2 (1 - 3)	0.2 (0.1 - 0.3)
Bahrain	Enterobacter spp.	19 (12 - 29)	3.2 (2.1 - 4.8)
Bahrain	Enterococcus faecalis	19 (11 - 32)	3.2 (1.8 - 5.3)
Bahrain	Enterococcus faecium	23 (14 - 39)	3.7 (2.3 - 6.1)
Bahrain	Escherichia coli	68 (43 - 107)	13.6 (8.9 - 20.5)
Bahrain	Group A Streptococcus	31 (18 - 50)	6.2 (3.8 - 9.9)
Bahrain	Group B Streptococcus	20 (12 - 31)	3.5 (2.1 - 5.5)
Bahrain	Haemophilus influenzae	3 (2 - 4)	0.6 (0.5 - 0.8)
Bahrain	Klebsiella pneumoniae	51 (32 - 79)	9.6 (6.3 - 14.4)
Bahrain	Legionella spp.	4 (3 - 5)	0.8 (0.6 - 1.0)
Bahrain	Listeria monocytogenes	0 (0 - 1)	0.0 (0.0 - 0.1)
Bahrain	Morganella spp.	0 (0 - 1)	0.1 (0.0 - 0.2)
Bahrain	Mycoplasma spp.	3 (3 - 5)	0.6 (0.4 - 0.7)
Bahrain	Neisseria gonorrhoeae	0 (0 - 0)	0.0 (0.0 - 0.0)
Bahrain	Neisseria meningitidis	5 (3 - 9)	0.5 (0.3 - 0.8)
Bahrain	Non-typhoidal Salmonella	3 (2 - 5)	0.4 (0.2 - 0.7)
Bahrain	Other Klebsiella species	4 (2 - 8)	0.7 (0.3 - 1.2)
Bahrain	Other enterococci	8 (5 - 13)	1.8 (1.1 - 2.8)
Bahrain	Proteus spp.	9 (5 - 14)	1.9 (1.2 - 2.9)
Bahrain	Providencia spp.	0 (0 - 0)	0.1 (0.0 - 0.1)
Bahrain	Pseudomonas aeruginosa	46 (29 - 70)	8.7 (5.7 - 12.9)
Bahrain	Salmonella Paratyphi	0 (0 - 0)	0.0 (0.0 - 0.0)
Bahrain	Salmonella Typhi	3 (2 - 7)	0.3 (0.2 - 0.7)
Bahrain	Serratia spp.	6 (4 - 11)	0.9 (0.5 - 1.6)
Bahrain	Shigella spp.	1 (0 - 2)	0.2 (0.1 - 0.4)
Bahrain	Staphylococcus aureus	100 (64 - 152)	18.7 (13.0 - 26.9)
Bahrain	Streptococcus pneumoniae	33 (23 - 49)	5.9 (4.4 - 8.1)
Bahrain	Vibrio cholerae	0 (0 - 1)	0.1 (0.0 - 0.1)
Egypt	Acinetobacter baumannii	6,175 (3,490 - 9,760)	11.5 (6.4 - 17.8)
Egypt	Aeromonas spp.	147 (49 - 344)	0.2 (0.1 - 0.3)
Egypt	Campylobacter spp.	299 (96 - 721)	0.4 (0.1 - 0.8)
Egypt	Chlamydia spp.	1,026 (736 - 1,410)	1.6 (1.1 - 2.2)
Egypt	Citrobacter spp.	971 (534 - 1,590)	1.7 (1.0 - 2.8)

Egypt	Clostridioides difficile	22 (8 - 51)	0.0 (0.0 - 0.1)
Egypt	Enterobacter spp.	5,404 (3,069 - 8,349)	9.5 (5.5 - 14.5)
Egypt	Enterococcus faecalis	3,455 (1,742 - 5,844)	6.6 (3.2 - 11.9)
Egypt	Enterococcus faecium	3,842 (2,113 - 6,084)	7.3 (4.1 - 11.7)
Egypt	Escherichia coli	10,461 (6,480 - 15,926)	20.6 (12.9 - 30.8)
Egypt	Group A Streptococcus	1,479 (605 - 3,084)	2.6 (1.1 - 5.6)
Egypt	Group B Streptococcus	1,685 (1,083 - 2,486)	2.7 (1.7 - 4.0)
Egypt	Haemophilus influenzae	698 (499 - 960)	1.0 (0.7 - 1.3)
Egypt	Klebsiella pneumoniae	8,244 (5,078 - 12,324)	15.6 (9.6 - 23.1)
Egypt	Legionella spp.	404 (272 - 581)	0.6 (0.4 - 0.8)
Egypt	Listeria monocytogenes	65 (35 - 120)	0.1 (0.0 - 0.1)
Egypt	Morganella spp.	45 (17 - 90)	0.1 (0.0 - 0.2)
Egypt	Mycoplasma spp.	1,220 (894 - 1,643)	1.5 (1.1 - 2.0)
Egypt	Neisseria gonorrhoeae	4 (2 - 6)	0.0 (0.0 - 0.0)
Egypt	Neisseria meningitidis	1,074 (570 - 1,805)	1.3 (0.7 - 2.2)
Egypt	Non-typhoidal Salmonella	1,199 (704 - 1,881)	1.7 (1.0 - 2.7)
Egypt	Other Klebsiella species	1,337 (632 - 2,434)	2.6 (1.2 - 5.0)
Egypt	Other enterococci	906 (500 - 1,443)	1.7 (0.9 - 2.7)
Egypt	Proteus spp.	1,515 (864 - 2,366)	3.2 (1.9 - 4.9)
Egypt	Providencia spp.	46 (17 - 90)	0.1 (0.0 - 0.2)
Egypt	Pseudomonas aeruginosa	6,090 (3,739 - 9,135)	11.3 (7.0 - 16.7)
Egypt	Salmonella Paratyphi	4 (1 - 9)	0.0 (0.0 - 0.0)
Egypt	Salmonella Typhi	434 (221 - 727)	0.5 (0.2 - 0.8)
Egypt	Serratia spp.	1,441 (820 - 2,323)	2.5 (1.4 - 4.0)
Egypt	Shigella spp.	365 (115 - 893)	0.4 (0.1 - 0.9)
Egypt	Staphylococcus aureus	9,442 (5,884 - 14,109)	17.1 (10.8 - 25.2)
Egypt	Streptococcus pneumoniae	7,110 (5,146 - 9,551)	10.2 (7.3 - 13.8)
Egypt	Vibrio cholerae	998 (495 - 1,773)	1.5 (0.8 - 2.7)
Iran (Islamic Republic of)	Acinetobacter baumannii	4,507 (2,564 - 7,170)	6.9 (3.9 - 11.1)
Iran (Islamic Republic of)	Aeromonas spp.	13 (6 - 25)	0.0 (0.0 - 0.0)
Iran (Islamic Republic of)	Campylobacter spp.	16 (4 - 44)	0.0 (0.0 - 0.1)
Iran (Islamic Republic of)	Chlamydia spp.	578 (443 - 769)	0.9 (0.7 - 1.2)
Iran (Islamic Republic of)	Citrobacter spp.	406 (225 - 656)	0.6 (0.3 - 1.0)
Iran (Islamic Republic of)	Clostridioides difficile	99 (56 - 176)	0.1 (0.1 - 0.2)
Iran (Islamic Republic of)	Enterobacter spp.	2,871 (1,690 - 4,512)	4.3 (2.5 - 6.7)
Iran (Islamic Republic of)	Enterococcus faecalis	1,049 (600 - 1,678)	1.6 (0.9 - 2.6)
Iran (Islamic Republic of)	Enterococcus faecium	1,171 (701 - 1,839)	1.7 (1.1 - 2.7)
Iran (Islamic Republic of)	Escherichia coli	3,604 (2,384 - 5,268)	5.6 (3.7 - 8.1)
Iran (Islamic Republic of)	Group A Streptococcus	1,129 (497 - 2,203)	1.7 (0.7 - 3.4)
Iran (Islamic Republic of)	Group B Streptococcus	1,122 (703 - 1,715)	1.7 (1.1 - 2.6)
Iran (Islamic Republic of)	Haemophilus influenzae	336 (257 - 439)	0.5 (0.4 - 0.7)

Iran (Islamic Republic of)	Klebsiella pneumoniae	3,020 (1,992 - 4,476)	4.7 (3.1 - 6.9)
Iran (Islamic Republic of)	Legionella spp.	347 (257 - 477)	0.5 (0.4 - 0.7)
Iran (Islamic Republic of)	Listeria monocytogenes	52 (36 - 83)	0.1 (0.1 - 0.1)
Iran (Islamic Republic of)	Morganella spp.	26 (12 - 46)	0.0 (0.0 - 0.1)
Iran (Islamic Republic of)	Mycoplasma spp.	691 (550 - 881)	1.0 (0.8 - 1.3)
Iran (Islamic Republic of)	Neisseria gonorrhoeae	14 (3 - 17)	0.0 (0.0 - 0.0)
Iran (Islamic Republic of)	Neisseria meningitidis	509 (288 - 825)	0.7 (0.4 - 1.1)
Iran (Islamic Republic of)	Non-typhoidal Salmonella	440 (268 - 670)	0.6 (0.4 - 1.0)
Iran (Islamic Republic of)	Other Klebsiella species	252 (132 - 443)	0.4 (0.2 - 0.7)
Iran (Islamic Republic of)	Other enterococci	624 (366 - 959)	1.0 (0.6 - 1.5)
Iran (Islamic Republic of)	Proteus spp.	525 (325 - 797)	0.8 (0.5 - 1.3)
Iran (Islamic Republic of)	Providencia spp.	19 (9 - 34)	0.0 (0.0 - 0.1)
Iran (Islamic Republic of)	Pseudomonas aeruginosa	2,913 (1,855 - 4,313)	4.5 (2.9 - 6.6)
Iran (Islamic Republic of)	Salmonella Paratyphi	1 (0 - 2)	0.0 (0.0 - 0.0)
Iran (Islamic Republic of)	Salmonella Typhi	251 (121 - 444)	0.3 (0.2 - 0.6)
Iran (Islamic Republic of)	Serratia spp.	568 (330 - 906)	0.8 (0.5 - 1.3)
Iran (Islamic Republic of)	Shigella spp.	182 (81 - 344)	0.3 (0.1 - 0.6)
Iran (Islamic Republic of)	Staphylococcus aureus	4,893 (3,243 - 7,152)	7.5 (5.0 - 10.9)
Iran (Islamic Republic of)	Streptococcus pneumoniae	3,443 (2,675 - 4,467)	5.2 (4.0 - 6.7)
Iran (Islamic Republic of)	Vibrio cholerae	24 (9 - 51)	0.0 (0.0 - 0.1)
Iraq	Acinetobacter baumannii	1,397 (783 - 2,236)	6.6 (3.6 - 10.5)
Iraq	Aeromonas spp.	6 (3 - 11)	0.0 (0.0 - 0.0)
Iraq	Campylobacter spp.	17 (5 - 41)	0.1 (0.0 - 0.2)
Iraq	Chlamydia spp.	192 (126 - 286)	0.7 (0.5 - 0.9)
Iraq	Citrobacter spp.	161 (94 - 257)	0.7 (0.4 - 1.1)
Iraq	Clostridioides difficile	13 (5 - 28)	0.0 (0.0 - 0.1)
Iraq	Enterobacter spp.	970 (588 - 1,482)	4.0 (2.3 - 6.1)
Iraq	Enterococcus faecalis	525 (299 - 855)	2.3 (1.3 - 3.7)
Iraq	Enterococcus faecium	589 (329 - 952)	2.7 (1.5 - 4.2)
Iraq	Escherichia coli	1,899 (1,148 - 2,907)	8.8 (5.3 - 13.4)
Iraq	Group A Streptococcus	622 (298 - 1,211)	2.7 (1.1 - 5.5)
Iraq	Group B Streptococcus	805 (494 - 1,212)	2.9 (1.8 - 4.5)
Iraq	Haemophilus influenzae	213 (148 - 308)	0.7 (0.5 - 1.0)
Iraq	Klebsiella pneumoniae	1,929 (1,203 - 2,926)	8.3 (5.1 - 12.7)
Iraq	Legionella spp.	122 (71 - 223)	0.4 (0.3 - 0.6)
Iraq	Listeria monocytogenes	42 (25 - 71)	0.1 (0.1 - 0.2)
Iraq	Morganella spp.	17 (7 - 32)	0.1 (0.0 - 0.2)
Iraq	Mycoplasma spp.	247 (173 - 353)	0.7 (0.5 - 1.0)
Iraq	Neisseria gonorrhoeae	2 (1 - 3)	0.0 (0.0 - 0.0)
Iraq	Neisseria meningitidis	444 (259 - 709)	1.2 (0.7 - 2.0)

Iraq	Non-typhoidal Salmonella	326 (209 - 469)	1.1 (0.7 - 1.6)
Iraq	Other Klebsiella species	97 (45 - 181)	0.4 (0.2 - 0.8)
Iraq	Other enterococci	321 (183 - 519)	1.5 (0.9 - 2.4)
Iraq	Proteus spp.	272 (158 - 440)	1.4 (0.8 - 2.2)
Iraq	Providencia spp.	15 (6 - 30)	0.1 (0.0 - 0.2)
Iraq	Pseudomonas aeruginosa	1,530 (928 - 2,364)	6.8 (4.1 - 10.4)
Iraq	Salmonella Paratyphi	1 (0 - 3)	0.0 (0.0 - 0.0)
Iraq	Salmonella Typhi	177 (99 - 285)	0.4 (0.3 - 0.7)
Iraq	Serratia spp.	340 (198 - 547)	1.4 (0.8 - 2.3)
Iraq	Shigella spp.	56 (21 - 120)	0.2 (0.1 - 0.4)
Iraq	Staphylococcus aureus	2,447 (1,528 - 3,682)	10.7 (6.7 - 16.1)
Iraq	Streptococcus pneumoniae	1,863 (1,306 - 2,623)	6.6 (4.6 - 9.3)
Iraq	Vibrio cholerae	97 (45 - 202)	0.4 (0.2 - 0.9)
Jordan	Acinetobacter baumannii	411 (237 - 671)	7.2 (4.1 - 11.7)
Jordan	Aeromonas spp.	1 (0 - 2)	0.0 (0.0 - 0.0)
Jordan	Campylobacter spp.	2 (1 - 5)	0.0 (0.0 - 0.1)
Jordan	Chlamydia spp.	64 (45 - 92)	0.9 (0.7 - 1.3)
Jordan	Citrobacter spp.	47 (28 - 77)	0.7 (0.4 - 1.1)
Jordan	Clostridioides difficile	11 (6 - 19)	0.1 (0.1 - 0.2)
Jordan	Enterobacter spp.	332 (204 - 520)	5.0 (3.0 - 7.7)
Jordan	Enterococcus faecalis	108 (62 - 171)	1.9 (1.1 - 3.0)
Jordan	Enterococcus faecium	115 (69 - 182)	2.1 (1.3 - 3.2)
Jordan	Escherichia coli	353 (231 - 513)	6.6 (4.4 - 9.5)
Jordan	Group A Streptococcus	125 (64 - 229)	2.2 (1.1 - 4.2)
Jordan	Group B Streptococcus	136 (87 - 207)	2.0 (1.3 - 3.1)
Jordan	Haemophilus influenzae	46 (33 - 63)	0.6 (0.5 - 0.8)
Jordan	Klebsiella pneumoniae	316 (209 - 465)	5.4 (3.6 - 7.9)
Jordan	Legionella spp.	37 (24 - 62)	0.5 (0.4 - 0.7)
Jordan	Listeria monocytogenes	6 (4 - 10)	0.1 (0.1 - 0.1)
Jordan	Morganella spp.	2 (1 - 4)	0.1 (0.0 - 0.1)
Jordan	Mycoplasma spp.	96 (71 - 133)	1.1 (0.9 - 1.5)
Jordan	Neisseria gonorrhoeae	0 (0 - 0)	0.0 (0.0 - 0.0)
Jordan	Neisseria meningitidis	74 (44 - 121)	0.8 (0.4 - 1.2)
Jordan	Non-typhoidal Salmonella	52 (33 - 77)	0.7 (0.4 - 1.0)
Jordan	Other Klebsiella species	25 (13 - 44)	0.5 (0.2 - 0.8)
Jordan	Other enterococci	64 (39 - 101)	1.2 (0.7 - 1.8)
Jordan	Proteus spp.	50 (31 - 76)	1.0 (0.6 - 1.5)
Jordan	Providencia spp.	2 (1 - 3)	0.0 (0.0 - 0.1)
Jordan	Pseudomonas aeruginosa	290 (185 - 444)	5.0 (3.3 - 7.6)
Jordan	Salmonella Paratyphi	0 (0 - 1)	0.0 (0.0 - 0.0)

Jordan	Salmonella Typhi	36 (17 - 66)	0.3 (0.2 - 0.5)
Jordan	Serratia spp.	62 (37 - 101)	1.0 (0.6 - 1.6)
Jordan	Shigella spp.	2 (1 - 5)	0.0 (0.0 - 0.1)
Jordan	Staphylococcus aureus	471 (312 - 703)	8.1 (5.5 - 12.0)
Jordan	Streptococcus pneumoniae	424 (319 - 569)	5.9 (4.5 - 7.9)
Jordan	Vibrio cholerae	10 (4 - 20)	0.2 (0.1 - 0.3)
Kuwait	Acinetobacter baumannii	46 (26 - 76)	2.0 (1.2 - 3.3)
Kuwait	Aeromonas spp.	0 (0 - 0)	0.0 (0.0 - 0.0)
Kuwait	Campylobacter spp.	0 (0 - 1)	0.0 (0.0 - 0.0)
Kuwait	Chlamydia spp.	12 (9 - 14)	0.6 (0.5 - 0.7)
Kuwait	Citrobacter spp.	5 (3 - 8)	0.2 (0.1 - 0.3)
Kuwait	Clostridioides difficile	3 (2 - 5)	0.1 (0.1 - 0.2)
Kuwait	Enterobacter spp.	39 (26 - 59)	1.8 (1.2 - 2.5)
Kuwait	Enterococcus faecalis	33 (19 - 54)	1.4 (0.8 - 2.3)
Kuwait	Enterococcus faecium	41 (24 - 65)	1.7 (1.0 - 2.7)
Kuwait	Escherichia coli	140 (95 - 206)	6.7 (4.7 - 9.7)
Kuwait	Group A Streptococcus	32 (15 - 60)	1.3 (0.6 - 2.4)
Kuwait	Group B Streptococcus	44 (29 - 67)	1.9 (1.3 - 2.7)
Kuwait	Haemophilus influenzae	15 (13 - 19)	0.8 (0.6 - 0.9)
Kuwait	Klebsiella pneumoniae	112 (79 - 160)	5.3 (3.8 - 7.5)
Kuwait	Legionella spp.	27 (22 - 33)	1.3 (1.1 - 1.6)
Kuwait	Listeria monocytogenes	1 (1 - 2)	0.0 (0.0 - 0.1)
Kuwait	Morganella spp.	1 (0 - 1)	0.0 (0.0 - 0.1)
Kuwait	Mycoplasma spp.	21 (17 - 26)	0.9 (0.7 - 1.1)
Kuwait	Neisseria gonorrhoeae	0 (0 - 0)	0.0 (0.0 - 0.0)
Kuwait	Neisseria meningitidis	9 (5 - 16)	0.3 (0.1 - 0.4)
Kuwait	Non-typhoidal Salmonella	5 (3 - 7)	0.2 (0.1 - 0.2)
Kuwait	Other Klebsiella species	7 (4 - 12)	0.3 (0.2 - 0.5)
Kuwait	Other enterococci	14 (9 - 21)	0.7 (0.4 - 1.0)
Kuwait	Proteus spp.	14 (9 - 21)	0.7 (0.5 - 1.0)
Kuwait	Providencia spp.	0 (0 - 1)	0.0 (0.0 - 0.0)
Kuwait	Pseudomonas aeruginosa	109 (77 - 153)	5.2 (3.8 - 7.1)
Kuwait	Salmonella Paratyphi	2 (0 - 4)	0.0 (0.0 - 0.1)
Kuwait	Salmonella Typhi	6 (3 - 12)	0.2 (0.1 - 0.4)
Kuwait	Serratia spp.	10 (6 - 17)	0.4 (0.2 - 0.7)
Kuwait	Shigella spp.	0 (0 - 1)	0.0 (0.0 - 0.0)
Kuwait	Staphylococcus aureus	315 (236 - 427)	15.4 (11.9 - 20.3)
Kuwait	Streptococcus pneumoniae	134 (108 - 171)	6.3 (5.1 - 7.8)
Kuwait	Vibrio cholerae	2 (1 - 4)	0.1 (0.0 - 0.2)
Lebanon	Acinetobacter baumannii	187 (106 - 305)	3.7 (2.1 - 5.9)

Lebanon	Aeromonas spp.	1 (0 - 2)	0.0 (0.0 - 0.0)
Lebanon	Campylobacter spp.	2 (0 - 6)	0.0 (0.0 - 0.1)
Lebanon	Chlamydia spp.	22 (17 - 30)	0.4 (0.3 - 0.6)
Lebanon	Citrobacter spp.	18 (11 - 29)	0.4 (0.2 - 0.6)
Lebanon	Clostridioides difficile	5 (2 - 10)	0.1 (0.0 - 0.2)
Lebanon	Enterobacter spp.	133 (84 - 206)	2.6 (1.6 - 4.0)
Lebanon	Enterococcus faecalis	91 (50 - 153)	1.8 (1.0 - 3.0)
Lebanon	Enterococcus faecium	119 (69 - 196)	2.3 (1.4 - 3.8)
Lebanon	Escherichia coli	363 (231 - 558)	7.1 (4.5 - 10.9)
Lebanon	Group A Streptococcus	89 (39 - 174)	1.7 (0.8 - 3.4)
Lebanon	Group B Streptococcus	118 (76 - 178)	2.3 (1.5 - 3.5)
Lebanon	Haemophilus influenzae	28 (22 - 37)	0.6 (0.4 - 0.7)
Lebanon	Klebsiella pneumoniae	285 (186 - 428)	5.6 (3.6 - 8.4)
Lebanon	Legionella spp.	30 (23 - 40)	0.6 (0.5 - 0.8)
Lebanon	Listeria monocytogenes	3 (2 - 5)	0.1 (0.0 - 0.1)
Lebanon	Morganella spp.	1 (1 - 3)	0.0 (0.0 - 0.1)
Lebanon	Mycoplasma spp.	35 (27 - 46)	0.7 (0.5 - 0.9)
Lebanon	Neisseria gonorrhoeae	0 (0 - 1)	0.0 (0.0 - 0.0)
Lebanon	Neisseria meningitidis	24 (14 - 40)	0.5 (0.3 - 0.8)
Lebanon	Non-typhoidal Salmonella	17 (10 - 25)	0.3 (0.2 - 0.5)
Lebanon	Other Klebsiella species	19 (9 - 35)	0.4 (0.2 - 0.7)
Lebanon	Other enterococci	45 (26 - 70)	0.9 (0.5 - 1.4)
Lebanon	Proteus spp.	43 (26 - 67)	0.8 (0.5 - 1.3)
Lebanon	Providencia spp.	1 (0 - 2)	0.0 (0.0 - 0.0)
Lebanon	Pseudomonas aeruginosa	268 (174 - 398)	5.2 (3.4 - 7.8)
Lebanon	Salmonella Paratyphi	0 (0 - 0)	0.0 (0.0 - 0.0)
Lebanon	Salmonella Typhi	15 (7 - 26)	0.3 (0.1 - 0.5)
Lebanon	Serratia spp.	36 (21 - 59)	0.7 (0.4 - 1.2)
Lebanon	Shigella spp.	4 (1 - 10)	0.1 (0.0 - 0.2)
Lebanon	Staphylococcus aureus	628 (419 - 927)	12.3 (8.3 - 18.1)
Lebanon	Streptococcus pneumoniae	276 (208 - 372)	5.4 (4.1 - 7.3)
Lebanon	Vibrio cholerae	36 (17 - 69)	0.7 (0.3 - 1.4)
Libya	Acinetobacter baumannii	206 (113 - 347)	4.4 (2.5 - 7.3)
Libya	Aeromonas spp.	1 (1 - 2)	0.0 (0.0 - 0.1)
Libya	Campylobacter spp.	2 (0 - 6)	0.1 (0.0 - 0.2)
Libya	Chlamydia spp.	25 (18 - 36)	0.5 (0.4 - 0.8)
Libya	Citrobacter spp.	19 (10 - 31)	0.4 (0.2 - 0.6)
Libya	Clostridioides difficile	8 (4 - 14)	0.1 (0.1 - 0.3)
Libya	Enterobacter spp.	125 (74 - 195)	2.7 (1.6 - 4.1)
Libya	Enterococcus faecalis	105 (57 - 173)	2.2 (1.2 - 3.6)
Libya	Enterococcus faecium	122 (67 - 198)	2.6 (1.4 - 4.1)

Libya	Escherichia coli	406 (242 - 625)	8.9 (5.4 - 13.6)
Libya	Group A Streptococcus	107 (49 - 205)	2.2 (1.0 - 4.2)
Libya	Group B Streptococcus	118 (73 - 184)	2.5 (1.5 - 3.7)
Libya	Haemophilus influenzae	34 (25 - 48)	0.7 (0.5 - 1.0)
Libya	Klebsiella pneumoniae	378 (234 - 583)	8.3 (5.2 - 12.6)
Libya	Legionella spp.	24 (18 - 33)	0.5 (0.4 - 0.7)
Libya	Listeria monocytogenes	5 (3 - 10)	0.1 (0.1 - 0.2)
Libya	Morganella spp.	2 (1 - 4)	0.0 (0.0 - 0.1)
Libya	Mycoplasma spp.	32 (24 - 44)	0.6 (0.5 - 0.8)
Libya	Neisseria gonorrhoeae	0 (0 - 1)	0.0 (0.0 - 0.0)
Libya	Neisseria meningitidis	48 (25 - 83)	0.8 (0.4 - 1.4)
Libya	Non-typhoidal Salmonella	24 (14 - 37)	0.5 (0.3 - 0.7)
Libya	Other Klebsiella species	26 (13 - 48)	0.5 (0.3 - 1.0)
Libya	Other enterococci	43 (24 - 70)	0.9 (0.5 - 1.5)
Libya	Proteus spp.	52 (30 - 84)	1.2 (0.7 - 1.9)
Libya	Providencia spp.	2 (1 - 3)	0.0 (0.0 - 0.1)
Libya	Pseudomonas aeruginosa	307 (190 - 471)	6.7 (4.2 - 10.1)
Libya	Salmonella Paratyphi	0 (0 - 0)	0.0 (0.0 - 0.0)
Libya	Salmonella Typhi	27 (16 - 44)	0.5 (0.3 - 0.7)
Libya	Serratia spp.	45 (24 - 75)	0.9 (0.5 - 1.6)
Libya	Shigella spp.	10 (4 - 20)	0.2 (0.1 - 0.5)
Libya	Staphylococcus aureus	610 (403 - 899)	13.2 (8.9 - 19.2)
Libya	Streptococcus pneumoniae	316 (225 - 442)	6.5 (4.7 - 9.0)
Libya	Vibrio cholerae	26 (12 - 46)	0.6 (0.3 - 1.1)
Morocco	Acinetobacter baumannii	1,547 (845 - 2,549)	6.1 (3.3 - 9.9)
Morocco	Aeromonas spp.	21 (8 - 42)	0.1 (0.0 - 0.2)
Morocco	Campylobacter spp.	37 (10 - 86)	0.2 (0.0 - 0.4)
Morocco	Chlamydia spp.	200 (141 - 277)	0.8 (0.5 - 1.0)
Morocco	Citrobacter spp.	122 (71 - 195)	0.5 (0.3 - 0.7)
Morocco	Clostridioides difficile	7 (3 - 15)	0.0 (0.0 - 0.0)
Morocco	Enterobacter spp.	796 (499 - 1,207)	3.0 (1.9 - 4.5)
Morocco	Enterococcus faecalis	702 (396 - 1,134)	2.6 (1.4 - 4.3)
Morocco	Enterococcus faecium	794 (458 - 1,262)	2.9 (1.7 - 4.7)
Morocco	Escherichia coli	3,028 (1,910 - 4,531)	11.9 (7.5 - 17.8)
Morocco	Group A Streptococcus	675 (300 - 1,349)	2.6 (1.1 - 5.2)
Morocco	Group B Streptococcus	845 (547 - 1,250)	3.1 (2.0 - 4.6)
Morocco	Haemophilus influenzae	271 (194 - 365)	1.0 (0.7 - 1.4)
Morocco	Klebsiella pneumoniae	3,020 (1,903 - 4,492)	11.6 (7.4 - 17.1)
Morocco	Legionella spp.	191 (128 - 287)	0.7 (0.5 - 1.0)
Morocco	Listeria monocytogenes	44 (26 - 76)	0.1 (0.1 - 0.3)
Morocco	Morganella spp.	16 (7 - 31)	0.1 (0.0 - 0.1)

Morocco	Mycoplasma spp.	190 (138 - 257)	0.7 (0.5 - 0.9)
Morocco	Neisseria gonorrhoeae	4 (2 - 11)	0.0 (0.0 - 0.0)
Morocco	Neisseria meningitidis	308 (174 - 497)	1.0 (0.6 - 1.6)
Morocco	Non-typhoidal Salmonella	277 (164 - 453)	1.0 (0.6 - 1.6)
Morocco	Other Klebsiella species	168 (84 - 306)	0.6 (0.3 - 1.2)
Morocco	Other enterococci	300 (170 - 481)	1.2 (0.7 - 1.9)
Morocco	Proteus spp.	388 (226 - 616)	1.6 (0.9 - 2.5)
Morocco	Providencia spp.	16 (7 - 32)	0.1 (0.0 - 0.1)
Morocco	Pseudomonas aeruginosa	2,190 (1,384 - 3,260)	8.5 (5.4 - 12.5)
Morocco	Salmonella Paratyphi	1 (1 - 3)	0.0 (0.0 - 0.0)
Morocco	Salmonella Typhi	245 (142 - 378)	0.8 (0.4 - 1.2)
Morocco	Serratia spp.	338 (192 - 565)	1.2 (0.7 - 2.0)
Morocco	Shigella spp.	114 (41 - 261)	0.4 (0.1 - 1.0)
Morocco	Staphylococcus aureus	4,243 (2,911 - 6,019)	16.5 (11.5 - 23.2)
Morocco	Streptococcus pneumoniae	2,172 (1,580 - 2,993)	8.1 (5.9 - 11.0)
Morocco	Vibrio cholerae	273 (122 - 524)	1.1 (0.5 - 2.1)
Oman	Acinetobacter baumannii	60 (33 - 99)	4.6 (2.6 - 7.7)
Oman	Aeromonas spp.	0 (0 - 1)	0.0 (0.0 - 0.1)
Oman	Campylobacter spp.	1 (0 - 4)	0.2 (0.0 - 0.5)
Oman	Chlamydia spp.	10 (8 - 12)	0.8 (0.6 - 1.0)
Oman	Citrobacter spp.	6 (4 - 10)	0.4 (0.2 - 0.6)
Oman	Clostridioides difficile	5 (3 - 9)	0.2 (0.1 - 0.3)
Oman	Enterobacter spp.	46 (29 - 69)	3.3 (2.2 - 4.7)
Oman	Enterococcus faecalis	37 (21 - 59)	2.5 (1.4 - 4.1)
Oman	Enterococcus faecium	43 (24 - 70)	3.0 (1.8 - 4.8)
Oman	Escherichia coli	138 (87 - 203)	11.5 (7.7 - 16.6)
Oman	Group A Streptococcus	59 (36 - 96)	4.5 (2.7 - 7.6)
Oman	Group B Streptococcus	52 (34 - 77)	3.6 (2.5 - 5.3)
Oman	Haemophilus influenzae	13 (10 - 17)	1.0 (0.8 - 1.3)
Oman	Klebsiella pneumoniae	117 (78 - 172)	9.3 (6.5 - 13.4)
Oman	Legionella spp.	15 (12 - 20)	1.2 (1.0 - 1.5)
Oman	Listeria monocytogenes	2 (1 - 3)	0.1 (0.1 - 0.2)
Oman	Morganella spp.	1 (0 - 1)	0.1 (0.0 - 0.1)
Oman	Mycoplasma spp.	17 (14 - 22)	1.0 (0.9 - 1.3)
Oman	Neisseria gonorrhoeae	0 (0 - 0)	0.0 (0.0 - 0.0)
Oman	Neisseria meningitidis	15 (8 - 25)	0.5 (0.3 - 0.8)
Oman	Non-typhoidal Salmonella	9 (6 - 13)	0.4 (0.2 - 0.6)
Oman	Other Klebsiella species	7 (4 - 14)	0.5 (0.2 - 0.9)
Oman	Other enterococci	17 (10 - 26)	1.4 (0.9 - 2.2)
Oman	Proteus spp.	16 (10 - 24)	1.4 (0.9 - 2.1)

Oman	Providencia spp.	0 (0 - 1)	0.0 (0.0 - 0.1)
Oman	Pseudomonas aeruginosa	108 (73 - 155)	8.8 (6.2 - 12.2)
Oman	Salmonella Paratyphi	0 (0 - 0)	0.0 (0.0 - 0.0)
Oman	Salmonella Typhi	12 (5 - 25)	0.3 (0.1 - 0.6)
Oman	Serratia spp.	14 (8 - 22)	0.9 (0.5 - 1.4)
Oman	Shigella spp.	3 (1 - 6)	0.3 (0.1 - 0.6)
Oman	Staphylococcus aureus	254 (180 - 356)	21.2 (16.0 - 28.6)
Oman	Streptococcus pneumoniae	124 (98 - 159)	9.1 (7.3 - 11.2)
Oman	Vibrio cholerae	5 (2 - 9)	0.3 (0.1 - 0.7)
Palestine	Acinetobacter baumannii	116 (64 - 189)	5.6 (3.1 - 9.3)
Palestine	Aeromonas spp.	1 (0 - 1)	0.0 (0.0 - 0.1)
Palestine	Campylobacter spp.	2 (0 - 4)	0.1 (0.0 - 0.2)
Palestine	Chlamydia spp.	16 (12 - 23)	0.7 (0.5 - 0.9)
Palestine	Citrobacter spp.	12 (7 - 18)	0.5 (0.3 - 0.7)
Palestine	Clostridioides difficile	1 (0 - 2)	0.0 (0.0 - 0.0)
Palestine	Enterobacter spp.	69 (44 - 105)	3.1 (2.0 - 4.6)
Palestine	Enterococcus faecalis	60 (36 - 94)	2.7 (1.5 - 4.3)
Palestine	Enterococcus faecium	68 (41 - 108)	3.2 (1.9 - 5.0)
Palestine	Escherichia coli	243 (157 - 365)	12.1 (8.0 - 18.1)
Palestine	Group A Streptococcus	71 (37 - 130)	2.9 (1.3 - 5.7)
Palestine	Group B Streptococcus	76 (49 - 114)	2.9 (1.9 - 4.4)
Palestine	Haemophilus influenzae	22 (16 - 30)	0.9 (0.7 - 1.2)
Palestine	Klebsiella pneumoniae	243 (162 - 356)	10.8 (7.2 - 16.1)
Palestine	Legionella spp.	21 (14 - 32)	0.8 (0.6 - 1.1)
Palestine	Listeria monocytogenes	2 (2 - 4)	0.1 (0.1 - 0.1)
Palestine	Morganella spp.	1 (1 - 2)	0.1 (0.0 - 0.1)
Palestine	Mycoplasma spp.	21 (15 - 27)	0.7 (0.5 - 0.9)
Palestine	Neisseria gonorrhoeae	0 (0 - 0)	0.0 (0.0 - 0.0)
Palestine	Neisseria meningitidis	39 (24 - 60)	0.9 (0.5 - 1.4)
Palestine	Non-typhoidal Salmonella	30 (20 - 44)	0.8 (0.5 - 1.1)
Palestine	Other Klebsiella species	12 (6 - 22)	0.6 (0.3 - 1.1)
Palestine	Other enterococci	28 (17 - 43)	1.3 (0.8 - 2.1)
Palestine	Proteus spp.	30 (19 - 46)	1.6 (1.0 - 2.5)
Palestine	Providencia spp.	1 (1 - 2)	0.1 (0.0 - 0.1)
Palestine	Pseudomonas aeruginosa	186 (121 - 274)	8.7 (5.7 - 12.8)
Palestine	Salmonella Paratyphi	0 (0 - 1)	0.0 (0.0 - 0.0)
Palestine	Salmonella Typhi	32 (18 - 51)	0.6 (0.4 - 1.0)
Palestine	Serratia spp.	28 (17 - 44)	1.1 (0.6 - 1.8)
Palestine	Shigella spp.	3 (1 - 7)	0.1 (0.0 - 0.3)
Palestine	Staphylococcus aureus	378 (265 - 536)	18.2 (13.0 - 25.6)

Palestine	Streptococcus pneumoniae	192 (141 - 262)	7.6 (5.8 - 10.4)
Palestine	Vibrio cholerae	17 (10 - 28)	0.7 (0.3 - 1.3)
Qatar	Acinetobacter baumannii	23 (12 - 39)	4.7 (2.6 - 8.1)
Qatar	Aeromonas spp.	0 (0 - 0)	0.0 (0.0 - 0.0)
Qatar	Campylobacter spp.	0 (0 - 0)	0.1 (0.0 - 0.1)
Qatar	Chlamydia spp.	2 (1 - 3)	0.5 (0.4 - 0.7)
Qatar	Citrobacter spp.	3 (2 - 5)	0.5 (0.3 - 0.8)
Qatar	Clostridioides difficile	3 (2 - 4)	0.3 (0.1 - 0.5)
Qatar	Enterobacter spp.	18 (11 - 30)	3.5 (2.2 - 5.4)
Qatar	Enterococcus faecalis	19 (10 - 31)	3.4 (1.7 - 5.9)
Qatar	Enterococcus faecium	23 (13 - 39)	4.2 (2.5 - 6.9)
Qatar	Escherichia coli	59 (34 - 94)	14.0 (8.8 - 21.7)
Qatar	Group A Streptococcus	20 (10 - 36)	4.7 (2.4 - 8.2)
Qatar	Group B Streptococcus	20 (11 - 33)	3.5 (2.1 - 5.4)
Qatar	Haemophilus influenzae	3 (2 - 4)	0.7 (0.5 - 0.9)
Qatar	Klebsiella pneumoniae	43 (25 - 69)	9.6 (6.1 - 14.7)
Qatar	Legionella spp.	4 (3 - 6)	1.0 (0.7 - 1.2)
Qatar	Listeria monocytogenes	1 (0 - 1)	0.1 (0.0 - 0.1)
Qatar	Morganella spp.	0 (0 - 0)	0.1 (0.0 - 0.1)
Qatar	Mycoplasma spp.	5 (3 - 7)	0.7 (0.5 - 0.9)
Qatar	Neisseria gonorrhoeae	0 (0 - 0)	0.0 (0.0 - 0.0)
Qatar	Neisseria meningitidis	6 (3 - 10)	0.4 (0.2 - 0.7)
Qatar	Non-typhoidal Salmonella	3 (2 - 5)	0.3 (0.2 - 0.4)
Qatar	Other Klebsiella species	4 (2 - 8)	0.8 (0.4 - 1.5)
Qatar	Other enterococci	6 (3 - 10)	1.6 (0.9 - 2.5)
Qatar	Proteus spp.	6 (4 - 10)	1.7 (1.1 - 2.6)
Qatar	Providencia spp.	0 (0 - 0)	0.0 (0.0 - 0.1)
Qatar	Pseudomonas aeruginosa	39 (23 - 63)	8.9 (5.8 - 13.4)
Qatar	Salmonella Paratyphi	0 (0 - 0)	0.0 (0.0 - 0.0)
Qatar	Salmonella Typhi	5 (2 - 12)	0.3 (0.1 - 0.5)
Qatar	Serratia spp.	6 (3 - 10)	0.9 (0.6 - 1.5)
Qatar	Shigella spp.	0 (0 - 1)	0.1 (0.0 - 0.2)
Qatar	Staphylococcus aureus	95 (58 - 153)	20.8 (14.2 - 30.4)
Qatar	Streptococcus pneumoniae	33 (22 - 49)	6.4 (4.7 - 8.6)
Qatar	Vibrio cholerae	1 (0 - 1)	0.1 (0.0 - 0.3)
Saudi Arabia	Acinetobacter baumannii	643 (349 - 1,096)	4.2 (2.4 - 6.9)
Saudi Arabia	Aeromonas spp.	3 (2 - 7)	0.0 (0.0 - 0.1)
Saudi Arabia	Campylobacter spp.	5 (1 - 15)	0.1 (0.0 - 0.2)
Saudi Arabia	Chlamydia spp.	103 (78 - 137)	0.7 (0.5 - 0.8)
Saudi Arabia	Citrobacter spp.	69 (42 - 109)	0.5 (0.3 - 0.7)

Saudi Arabia	Clostridioides difficile	55 (28 - 102)	0.2 (0.1 - 0.4)
Saudi Arabia	Enterobacter spp.	479 (300 - 739)	3.2 (2.2 - 4.7)
Saudi Arabia	Enterococcus faecalis	446 (252 - 719)	3.1 (1.8 - 4.9)
Saudi Arabia	Enterococcus faecium	528 (306 - 867)	3.5 (2.2 - 5.5)
Saudi Arabia	Escherichia coli	1,640 (1,082 - 2,460)	13.1 (9.2 - 18.7)
Saudi Arabia	Group A Streptococcus	528 (297 - 907)	3.6 (2.1 - 5.9)
Saudi Arabia	Group B Streptococcus	531 (335 - 833)	3.2 (2.1 - 4.6)
Saudi Arabia	Haemophilus influenzae	139 (105 - 184)	0.9 (0.7 - 1.1)
Saudi Arabia	Klebsiella pneumoniae	1,342 (889 - 1,999)	9.6 (6.7 - 13.8)
Saudi Arabia	Legionella spp.	191 (146 - 245)	1.1 (0.9 - 1.4)
Saudi Arabia	Listeria monocytogenes	20 (11 - 37)	0.1 (0.1 - 0.2)
Saudi Arabia	Morganella spp.	8 (5 - 12)	0.1 (0.0 - 0.1)
Saudi Arabia	Mycoplasma spp.	215 (161 - 282)	0.9 (0.7 - 1.2)
Saudi Arabia	Neisseria gonorrhoeae	1 (1 - 1)	0.0 (0.0 - 0.0)
Saudi Arabia	Neisseria meningitidis	172 (92 - 302)	0.6 (0.3 - 1.0)
Saudi Arabia	Non-typhoidal Salmonella	71 (42 - 114)	0.4 (0.2 - 0.7)
Saudi Arabia	Other Klebsiella species	101 (52 - 180)	0.7 (0.4 - 1.2)
Saudi Arabia	Other enterococci	173 (108 - 264)	1.5 (1.0 - 2.1)
Saudi Arabia	Proteus spp.	190 (119 - 287)	1.7 (1.1 - 2.4)
Saudi Arabia	Providencia spp.	5 (3 - 9)	0.1 (0.0 - 0.1)
Saudi Arabia	Pseudomonas aeruginosa	1,206 (792 - 1,804)	8.5 (5.8 - 12.2)
Saudi Arabia	Salmonella Paratyphi	1 (0 - 2)	0.0 (0.0 - 0.0)
Saudi Arabia	Salmonella Typhi	79 (39 - 147)	0.3 (0.1 - 0.5)
Saudi Arabia	Serratia spp.	151 (87 - 249)	0.9 (0.5 - 1.5)
Saudi Arabia	Shigella spp.	25 (9 - 51)	0.2 (0.1 - 0.5)
Saudi Arabia	Staphylococcus aureus	2,899 (2,010 - 4,216)	19.8 (14.4 - 27.6)
Saudi Arabia	Streptococcus pneumoniae	1,391 (1,032 - 1,888)	7.8 (6.0 - 10.0)
Saudi Arabia	Vibrio cholerae	13 (6 - 28)	0.1 (0.0 - 0.2)
Sudan	Acinetobacter baumannii	1,183 (675 - 1,868)	6.1 (3.4 - 10.0)
Sudan	Aeromonas spp.	103 (31 - 254)	0.2 (0.1 - 0.5)
Sudan	Campylobacter spp.	110 (26 - 292)	0.4 (0.1 - 0.9)
Sudan	Chlamydia spp.	422 (268 - 622)	1.2 (0.8 - 1.7)
Sudan	Citrobacter spp.	163 (91 - 262)	0.6 (0.4 - 1.0)
Sudan	Clostridioides difficile	10 (3 - 27)	0.0 (0.0 - 0.1)
Sudan	Enterobacter spp.	957 (598 - 1,430)	3.9 (2.4 - 5.8)
Sudan	Enterococcus faecalis	699 (396 - 1,116)	3.2 (1.7 - 5.4)
Sudan	Enterococcus faecium	623 (349 - 1,020)	3.1 (1.7 - 5.1)
Sudan	Escherichia coli	3,325 (2,129 - 4,758)	13.9 (8.8 - 20.0)
Sudan	Group A Streptococcus	932 (476 - 1,661)	4.2 (1.8 - 8.1)
Sudan	Group B Streptococcus	1,811 (1,160 - 2,560)	5.3 (3.5 - 7.6)
Sudan	Haemophilus influenzae	545 (359 - 771)	1.5 (1.1 - 2.0)

Sudan	Klebsiella pneumoniae	3,820 (2,449 - 5,512)	14.8 (9.5 - 21.4)
Sudan	Legionella spp.	251 (126 - 486)	0.7 (0.4 - 1.1)
Sudan	Listeria monocytogenes	60 (27 - 122)	0.2 (0.1 - 0.3)
Sudan	Morganella spp.	4 (2 - 8)	0.0 (0.0 - 0.1)
Sudan	Mycoplasma spp.	403 (270 - 573)	1.0 (0.7 - 1.4)
Sudan	Neisseria gonorrhoeae	3 (1 - 7)	0.0 (0.0 - 0.0)
Sudan	Neisseria meningitidis	895 (494 - 1,487)	2.1 (1.2 - 3.4)
Sudan	Non-typhoidal Salmonella	831 (491 - 1,386)	2.2 (1.3 - 3.5)
Sudan	Other Klebsiella species	156 (70 - 305)	0.8 (0.4 - 1.6)
Sudan	Other enterococci	300 (169 - 485)	1.4 (0.8 - 2.2)
Sudan	Proteus spp.	323 (185 - 517)	1.8 (1.1 - 2.9)
Sudan	Providencia spp.	5 (2 - 10)	0.0 (0.0 - 0.1)
Sudan	Pseudomonas aeruginosa	2,352 (1,503 - 3,385)	9.6 (6.1 - 14.0)
Sudan	Salmonella Paratyphi	3 (1 - 7)	0.0 (0.0 - 0.0)
Sudan	Salmonella Typhi	626 (382 - 957)	1.5 (0.9 - 2.2)
Sudan	Serratia spp.	429 (251 - 682)	1.7 (1.0 - 2.8)
Sudan	Shigella spp.	465 (127 - 1,179)	1.1 (0.4 - 2.7)
Sudan	Staphylococcus aureus	4,027 (2,705 - 5,674)	16.9 (11.7 - 23.8)
Sudan	Streptococcus pneumoniae	3,777 (2,632 - 5,232)	11.9 (8.6 - 16.0)
Sudan	Vibrio cholerae	2,299 (1,131 - 4,532)	7.9 (4.0 - 14.2)
Syrian Arab Republic	Acinetobacter baumannii	1,036 (586 - 1,681)	10.7 (6.0 - 17.1)
Syrian Arab Republic	Aeromonas spp.	2 (1 - 5)	0.0 (0.0 - 0.1)
Syrian Arab Republic	Campylobacter spp.	5 (1 - 15)	0.1 (0.0 - 0.2)
Syrian Arab Republic	Chlamydia spp.	126 (88 - 178)	1.2 (0.9 - 1.8)
Syrian Arab Republic	Citrobacter spp.	103 (57 - 168)	1.0 (0.6 - 1.6)
Syrian Arab Republic	Clostridioides difficile	8 (3 - 18)	0.1 (0.0 - 0.1)
Syrian Arab Republic	Enterobacter spp.	723 (414 - 1,136)	7.0 (4.0 - 10.9)
Syrian Arab Republic	Enterococcus faecalis	307 (179 - 496)	3.0 (1.7 - 4.9)
Syrian Arab Republic	Enterococcus faecium	315 (182 - 497)	3.1 (1.8 - 4.8)
Syrian Arab Republic	Escherichia coli	1,078 (718 - 1,574)	11.3 (7.6 - 16.4)
Syrian Arab Republic	Group A Streptococcus	327 (148 - 628)	3.4 (1.5 - 6.6)
Syrian Arab Republic	Group B Streptococcus	346 (227 - 517)	3.4 (2.2 - 5.1)
Syrian Arab Republic	Haemophilus influenzae	108 (77 - 152)	1.0 (0.7 - 1.4)
Syrian Arab Republic	Klebsiella pneumoniae	877 (565 - 1,312)	9.0 (5.8 - 13.3)
Syrian Arab Republic	Legionella spp.	47 (33 - 68)	0.4 (0.3 - 0.6)
Syrian Arab Republic	Listeria monocytogenes	21 (11 - 42)	0.2 (0.1 - 0.4)
Syrian Arab Republic	Morganella spp.	11 (7 - 18)	0.1 (0.1 - 0.2)
Syrian Arab Republic	Mycoplasma spp.	157 (116 - 221)	1.4 (1.0 - 1.9)
Syrian Arab Republic	Neisseria gonorrhoeae	3 (2 - 4)	0.0 (0.0 - 0.0)
Syrian Arab Republic	Neisseria meningitidis	146 (82 - 238)	1.2 (0.7 - 1.9)

Syrian Arab Republic	Non-typhoidal Salmonella	120 (73 - 185)	1.1 (0.6 - 1.7)
Syrian Arab Republic	Other Klebsiella species	72 (34 - 135)	0.7 (0.3 - 1.3)
Syrian Arab Republic	Other enterococci	175 (108 - 263)	1.9 (1.2 - 2.9)
Syrian Arab Republic	Proteus spp.	163 (105 - 241)	1.8 (1.1 - 2.6)
Syrian Arab Republic	Providencia spp.	9 (5 - 14)	0.1 (0.1 - 0.2)
Syrian Arab Republic	Pseudomonas aeruginosa	783 (499 - 1,172)	8.1 (5.1 - 12.1)
Syrian Arab Republic	Salmonella Paratyphi	1 (0 - 2)	0.0 (0.0 - 0.0)
Syrian Arab Republic	Salmonella Typhi	71 (36 - 123)	0.5 (0.3 - 0.8)
Syrian Arab Republic	Serratia spp.	155 (87 - 257)	1.5 (0.8 - 2.4)
Syrian Arab Republic	Shigella spp.	12 (5 - 27)	0.1 (0.1 - 0.3)
Syrian Arab Republic	Staphylococcus aureus	1,211 (776 - 1,786)	12.3 (7.9 - 18.1)
Syrian Arab Republic	Streptococcus pneumoniae	1,016 (734 - 1,422)	9.6 (7.0 - 13.4)
Syrian Arab Republic	Vibrio cholerae	57 (25 - 110)	0.6 (0.3 - 1.2)
Tunisia	Acinetobacter baumannii	729 (381 - 1,205)	6.6 (3.4 - 10.9)
Tunisia	Aeromonas spp.	1 (0 - 2)	0.0 (0.0 - 0.0)
Tunisia	Campylobacter spp.	5 (1 - 14)	0.0 (0.0 - 0.1)
Tunisia	Chlamydia spp.	92 (63 - 134)	0.9 (0.6 - 1.3)
Tunisia	Citrobacter spp.	64 (33 - 109)	0.6 (0.3 - 1.0)
Tunisia	Clostridioides difficile	8 (3 - 18)	0.1 (0.0 - 0.2)
Tunisia	Enterobacter spp.	473 (258 - 767)	4.2 (2.3 - 6.9)
Tunisia	Enterococcus faecalis	177 (92 - 308)	1.6 (0.8 - 2.7)
Tunisia	Enterococcus faecium	201 (110 - 329)	1.7 (1.0 - 2.8)
Tunisia	Escherichia coli	596 (356 - 933)	5.4 (3.3 - 8.3)
Tunisia	Group A Streptococcus	185 (73 - 382)	1.7 (0.7 - 3.4)
Tunisia	Group B Streptococcus	180 (106 - 289)	1.7 (1.0 - 2.6)
Tunisia	Haemophilus influenzae	55 (37 - 77)	0.5 (0.4 - 0.7)
Tunisia	Klebsiella pneumoniae	495 (299 - 773)	4.5 (2.7 - 6.9)
Tunisia	Legionella spp.	58 (39 - 81)	0.5 (0.4 - 0.8)
Tunisia	Listeria monocytogenes	8 (5 - 14)	0.1 (0.0 - 0.1)
Tunisia	Morganella spp.	3 (1 - 7)	0.0 (0.0 - 0.1)
Tunisia	Mycoplasma spp.	110 (77 - 151)	1.0 (0.7 - 1.4)
Tunisia	Neisseria gonorrhoeae	1 (1 - 1)	0.0 (0.0 - 0.0)
Tunisia	Neisseria meningitidis	64 (35 - 106)	0.6 (0.3 - 1.0)
Tunisia	Non-typhoidal Salmonella	67 (38 - 107)	0.6 (0.3 - 1.0)
Tunisia	Other Klebsiella species	44 (22 - 79)	0.4 (0.2 - 0.7)
Tunisia	Other enterococci	98 (52 - 164)	0.9 (0.5 - 1.5)
Tunisia	Proteus spp.	85 (49 - 137)	0.8 (0.4 - 1.2)
Tunisia	Providencia spp.	2 (1 - 5)	0.0 (0.0 - 0.0)
Tunisia	Pseudomonas aeruginosa	487 (288 - 779)	4.4 (2.6 - 7.0)
Tunisia	Salmonella Paratyphi	0 (0 - 1)	0.0 (0.0 - 0.0)

Tunisia	Salmonella Typhi	30 (13 - 57)	0.3 (0.1 - 0.5)
Tunisia	Serratia spp.	88 (47 - 148)	0.8 (0.4 - 1.3)
Tunisia	Shigella spp.	5 (1 - 11)	0.0 (0.0 - 0.1)
Tunisia	Staphylococcus aureus	837 (514 - 1,316)	7.5 (4.7 - 11.7)
Tunisia	Streptococcus pneumoniae	562 (388 - 796)	5.2 (3.6 - 7.3)
Tunisia	Vibrio cholerae	7 (3 - 18)	0.1 (0.0 - 0.2)
Turkey	Acinetobacter baumannii	2,579 (1,430 - 4,313)	3.2 (1.8 - 5.3)
Turkey	Aeromonas spp.	10 (4 - 19)	0.0 (0.0 - 0.0)
Turkey	Campylobacter spp.	28 (5 - 75)	0.0 (0.0 - 0.1)
Turkey	Chlamydia spp.	350 (259 - 473)	0.5 (0.3 - 0.6)
Turkey	Citrobacter spp.	259 (161 - 408)	0.3 (0.2 - 0.5)
Turkey	Clostridioides difficile	59 (29 - 117)	0.1 (0.0 - 0.1)
Turkey	Enterobacter spp.	1,742 (1,107 - 2,650)	2.2 (1.4 - 3.3)
Turkey	Enterococcus faecalis	1,558 (888 - 2,546)	1.9 (1.1 - 3.2)
Turkey	Enterococcus faecium	1,966 (1,168 - 3,185)	2.4 (1.4 - 3.9)
Turkey	Escherichia coli	6,729 (4,372 - 10,148)	8.4 (5.5 - 12.7)
Turkey	Group A Streptococcus	1,468 (659 - 2,840)	1.9 (0.9 - 3.6)
Turkey	Group B Streptococcus	1,727 (1,080 - 2,646)	2.2 (1.4 - 3.4)
Turkey	Haemophilus influenzae	466 (340 - 631)	0.6 (0.5 - 0.8)
Turkey	Klebsiella pneumoniae	5,447 (3,552 - 8,265)	6.9 (4.5 - 10.5)
Turkey	Legionella spp.	587 (432 - 789)	0.8 (0.6 - 1.0)
Turkey	Listeria monocytogenes	35 (22 - 60)	0.0 (0.0 - 0.1)
Turkey	Morganella spp.	37 (20 - 60)	0.0 (0.0 - 0.1)
Turkey	Mycoplasma spp.	460 (345 - 606)	0.6 (0.5 - 0.8)
Turkey	Neisseria gonorrhoeae	6 (4 - 8)	0.0 (0.0 - 0.0)
Turkey	Neisseria meningitidis	386 (216 - 621)	0.6 (0.3 - 0.9)
Turkey	Non-typhoidal Salmonella	247 (153 - 379)	0.3 (0.2 - 0.5)
Turkey	Other Klebsiella species	319 (168 - 580)	0.4 (0.2 - 0.7)
Turkey	Other enterococci	736 (442 - 1,145)	0.9 (0.6 - 1.4)
Turkey	Proteus spp.	821 (512 - 1,286)	1.0 (0.6 - 1.6)
Turkey	Providencia spp.	24 (12 - 40)	0.0 (0.0 - 0.0)
Turkey	Pseudomonas aeruginosa	4,692 (3,061 - 6,953)	5.9 (3.9 - 8.8)
Turkey	Salmonella Paratyphi	2 (1 - 4)	0.0 (0.0 - 0.0)
Turkey	Salmonella Typhi	237 (130 - 398)	0.3 (0.2 - 0.6)
Turkey	Serratia spp.	583 (334 - 955)	0.7 (0.4 - 1.2)
Turkey	Shigella spp.	54 (19 - 118)	0.1 (0.0 - 0.2)
Turkey	Staphylococcus aureus	11,015 (7,602 - 15,994)	13.8 (9.6 - 19.9)
Turkey	Streptococcus pneumoniae	4,266 (3,116 - 5,852)	5.5 (4.1 - 7.6)
Turkey	Vibrio cholerae	356 (162 - 643)	0.5 (0.2 - 0.8)
United Arab Emirates	Acinetobacter baumannii	180 (89 - 325)	6.5 (3.7 - 10.5)

United Arab Emirates	Aeromonas spp.	0 (0 - 1)	0.0 (0.0 - 0.1)
United Arab Emirates	Campylobacter spp.	1 (0 - 4)	0.1 (0.0 - 0.4)
United Arab Emirates	Chlamydia spp.	19 (12 - 28)	1.0 (0.7 - 1.3)
United Arab Emirates	Citrobacter spp.	13 (7 - 23)	0.4 (0.2 - 0.7)
United Arab Emirates	Clostridioides difficile	15 (7 - 30)	0.2 (0.1 - 0.5)
United Arab Emirates	Enterobacter spp.	89 (50 - 151)	3.2 (2.1 - 4.9)
United Arab Emirates	Enterococcus faecalis	79 (43 - 139)	2.4 (1.4 - 4.0)
United Arab Emirates	Enterococcus faecium	97 (51 - 168)	3.0 (1.8 - 4.9)
United Arab Emirates	Escherichia coli	279 (163 - 458)	12.2 (8.1 - 18.0)
United Arab Emirates	Group A Streptococcus	88 (40 - 173)	2.4 (1.0 - 4.9)
United Arab Emirates	Group B Streptococcus	98 (55 - 166)	3.3 (2.3 - 4.8)
United Arab Emirates	Haemophilus influenzae	26 (17 - 38)	1.3 (0.9 - 1.7)
United Arab Emirates	Klebsiella pneumoniae	284 (168 - 460)	12.3 (8.6 - 17.5)
United Arab Emirates	Legionella spp.	22 (15 - 31)	1.1 (0.8 - 1.5)
United Arab Emirates	Listeria monocytogenes	5 (3 - 9)	0.1 (0.0 - 0.2)
United Arab Emirates	Morganella spp.	1 (1 - 3)	0.1 (0.0 - 0.1)
United Arab Emirates	Mycoplasma spp.	28 (18 - 43)	0.9 (0.7 - 1.2)
United Arab Emirates	Neisseria gonorrhoeae	0 (0 - 0)	0.0 (0.0 - 0.0)
United Arab Emirates	Neisseria meningitidis	58 (28 - 108)	0.7 (0.4 - 1.3)
United Arab Emirates	Non-typhoidal Salmonella	24 (14 - 39)	0.6 (0.3 - 1.0)
United Arab Emirates	Other Klebsiella species	18 (8 - 36)	0.5 (0.2 - 0.9)
United Arab Emirates	Other enterococci	29 (15 - 50)	1.2 (0.7 - 2.0)
United Arab Emirates	Proteus spp.	33 (18 - 58)	1.5 (0.9 - 2.3)
United Arab Emirates	Providencia spp.	1 (1 - 3)	0.1 (0.0 - 0.1)
United Arab Emirates	Pseudomonas aeruginosa	235 (132 - 391)	10.0 (6.9 - 14.6)
United Arab Emirates	Salmonella Paratyphi	0 (0 - 0)	0.0 (0.0 - 0.0)
United Arab Emirates	Salmonella Typhi	37 (18 - 68)	0.5 (0.3 - 0.9)
United Arab Emirates	Serratia spp.	39 (21 - 70)	1.1 (0.7 - 1.9)
United Arab Emirates	Shigella spp.	3 (1 - 7)	0.2 (0.0 - 0.6)
United Arab Emirates	Staphylococcus aureus	482 (302 - 771)	23.8 (17.4 - 32.0)
United Arab Emirates	Streptococcus pneumoniae	263 (168 - 405)	10.9 (8.1 - 14.2)
United Arab Emirates	Vibrio cholerae	5 (2 - 11)	0.2 (0.1 - 0.5)
Yemen	Acinetobacter baumannii	1,079 (609 - 1,755)	7.7 (4.4 - 12.9)
Yemen	Aeromonas spp.	100 (25 - 264)	0.3 (0.1 - 0.6)
Yemen	Campylobacter spp.	137 (32 - 370)	0.5 (0.1 - 1.2)
Yemen	Chlamydia spp.	360 (226 - 535)	1.4 (1.0 - 2.0)
Yemen	Citrobacter spp.	150 (83 - 246)	0.8 (0.4 - 1.3)
Yemen	Clostridioides difficile	8 (3 - 20)	0.0 (0.0 - 0.1)
Yemen	Enterobacter spp.	859 (531 - 1,300)	4.8 (2.9 - 7.2)
Yemen	Enterococcus faecalis	581 (334 - 944)	3.6 (1.9 - 6.1)
Yemen	Enterococcus faecium	495 (280 - 818)	3.4 (1.9 - 5.7)

Yemen	Escherichia coli	2,793 (1,826 - 3,996)	15.5 (10.1 - 22.5)
Yemen	Group A Streptococcus	737 (371 - 1,307)	4.2 (1.8 - 8.4)
Yemen	Group B Streptococcus	1,700 (1,058 - 2,487)	6.4 (4.3 - 9.3)
Yemen	Haemophilus influenzae	505 (340 - 717)	1.9 (1.3 - 2.5)
Yemen	Klebsiella pneumoniae	3,211 (2,026 - 4,697)	16.9 (11.0 - 25.1)
Yemen	Legionella spp.	150 (75 - 288)	0.6 (0.3 - 0.9)
Yemen	Listeria monocytogenes	74 (36 - 137)	0.3 (0.1 - 0.5)
Yemen	Morganella spp.	6 (2 - 12)	0.1 (0.0 - 0.1)
Yemen	Mycoplasma spp.	332 (224 - 474)	1.1 (0.8 - 1.5)
Yemen	Neisseria gonorrhoeae	2 (1 - 3)	0.0 (0.0 - 0.0)
Yemen	Neisseria meningitidis	1,014 (576 - 1,643)	3.1 (1.8 - 5.0)
Yemen	Non-typhoidal Salmonella	1,030 (566 - 1,895)	3.4 (2.0 - 5.6)
Yemen	Other Klebsiella species	133 (60 - 257)	1.0 (0.4 - 1.8)
Yemen	Other enterococci	250 (136 - 412)	1.6 (0.9 - 2.6)
Yemen	Proteus spp.	262 (146 - 424)	2.1 (1.2 - 3.4)
Yemen	Providencia spp.	8 (3 - 17)	0.1 (0.0 - 0.2)
Yemen	Pseudomonas aeruginosa	1,894 (1,206 - 2,764)	10.7 (6.9 - 15.8)
Yemen	Salmonella Paratyphi	2 (1 - 4)	0.0 (0.0 - 0.0)
Yemen	Salmonella Typhi	562 (334 - 857)	1.8 (1.1 - 2.7)
Yemen	Serratia spp.	388 (223 - 625)	2.1 (1.2 - 3.4)
Yemen	Shigella spp.	549 (127 - 1,472)	1.5 (0.4 - 3.8)
Yemen	Staphylococcus aureus	3,091 (2,059 - 4,332)	17.8 (12.2 - 25.3)
Yemen	Streptococcus pneumoniae	3,514 (2,385 - 4,890)	14.6 (10.4 - 20.2)
Yemen	Vibrio cholerae	1,282 (611 - 2,768)	5.6 (2.8 - 10.3)
Bangladesh	Acinetobacter baumannii	5,553 (3,141 - 9,095)	5.0 (2.8 - 8.1)
Bangladesh	Aeromonas spp.	831 (277 - 1,972)	0.9 (0.3 - 2.1)
Bangladesh	Campylobacter spp.	3,927 (734 - 11,727)	4.0 (0.7 - 12.2)
Bangladesh	Chlamydia spp.	1,076 (784 - 1,457)	0.9 (0.7 - 1.2)
Bangladesh	Citrobacter spp.	662 (397 - 1,030)	0.5 (0.3 - 0.8)
Bangladesh	Clostridioides difficile	43 (12 - 125)	0.0 (0.0 - 0.1)
Bangladesh	Enterobacter spp.	3,821 (2,511 - 5,713)	3.2 (2.1 - 4.8)
Bangladesh	Enterococcus faecalis	3,996 (2,388 - 6,396)	3.3 (1.9 - 5.2)
Bangladesh	Enterococcus faecium	3,638 (2,173 - 5,937)	3.0 (1.8 - 4.8)
Bangladesh	Escherichia coli	15,655 (10,768 - 22,674)	13.5 (9.2 - 19.5)
Bangladesh	Group A Streptococcus	4,464 (1,989 - 8,712)	4.1 (1.9 - 7.8)
Bangladesh	Group B Streptococcus	4,573 (3,105 - 6,678)	3.8 (2.6 - 5.8)
Bangladesh	Haemophilus influenzae	1,458 (1,119 - 1,895)	1.2 (0.9 - 1.6)
Bangladesh	Klebsiella pneumoniae	15,891 (10,892 - 22,707)	13.5 (9.2 - 19.4)
Bangladesh	Legionella spp.	741 (478 - 1,163)	0.6 (0.4 - 1.0)
Bangladesh	Listeria monocytogenes	160 (106 - 240)	0.1 (0.1 - 0.2)
Bangladesh	Morganella spp.	62 (36 - 94)	0.1 (0.0 - 0.1)

Bangladesh	Mycoplasma spp.	949 (723 - 1,224)	0.7 (0.6 - 1.0)
Bangladesh	Neisseria gonorrhoeae	57 (33 - 167)	0.1 (0.0 - 0.1)
Bangladesh	Neisseria meningitidis	2,076 (1,300 - 3,287)	1.5 (0.9 - 2.4)
Bangladesh	Non-typhoidal Salmonella	2,030 (1,287 - 3,361)	1.6 (1.0 - 2.8)
Bangladesh	Other Klebsiella species	1,038 (518 - 1,842)	0.8 (0.4 - 1.4)
Bangladesh	Other enterococci	1,576 (943 - 2,579)	1.4 (0.8 - 2.3)
Bangladesh	Proteus spp.	2,070 (1,266 - 3,234)	1.8 (1.1 - 2.9)
Bangladesh	Providencia spp.	71 (43 - 111)	0.1 (0.0 - 0.1)
Bangladesh	Pseudomonas aeruginosa	9,986 (6,667 - 14,573)	8.6 (5.7 - 12.5)
Bangladesh	Salmonella Paratyphi	299 (120 - 635)	0.2 (0.1 - 0.4)
Bangladesh	Salmonella Typhi	9,023 (5,060 - 14,557)	5.9 (3.4 - 9.4)
Bangladesh	Serratia spp.	1,901 (1,148 - 3,129)	1.6 (0.9 - 2.6)
Bangladesh	Shigella spp.	2,217 (645 - 6,209)	2.2 (0.6 - 6.4)
Bangladesh	Staphylococcus aureus	19,480 (13,955 - 27,174)	16.7 (12.2 - 23.2)
Bangladesh	Streptococcus pneumoniae	11,993 (9,156 - 15,718)	9.9 (7.6 - 13.0)
Bangladesh	Vibrio cholerae	1,507 (479 - 4,287)	1.2 (0.4 - 3.4)
Bhutan	Acinetobacter baumannii	65 (38 - 104)	13.2 (7.6 - 21.0)
Bhutan	Aeromonas spp.	1 (0 - 3)	0.3 (0.1 - 0.7)
Bhutan	Campylobacter spp.	16 (3 - 42)	3.6 (0.6 - 9.9)
Bhutan	Chlamydia spp.	12 (8 - 18)	2.3 (1.5 - 3.3)
Bhutan	Citrobacter spp.	7 (4 - 11)	1.3 (0.8 - 2.1)
Bhutan	Clostridioides difficile	2 (1 - 4)	0.2 (0.1 - 0.5)
Bhutan	Enterobacter spp.	38 (24 - 60)	7.0 (4.4 - 11.0)
Bhutan	Enterococcus faecalis	20 (11 - 35)	3.7 (2.1 - 6.3)
Bhutan	Enterococcus faecium	18 (11 - 32)	3.5 (2.0 - 5.9)
Bhutan	Escherichia coli	72 (49 - 105)	14.0 (9.7 - 20.1)
Bhutan	Group A Streptococcus	14 (7 - 26)	2.7 (1.4 - 5.1)
Bhutan	Group B Streptococcus	19 (12 - 28)	3.5 (2.3 - 5.1)
Bhutan	Haemophilus influenzae	8 (5 - 11)	1.3 (0.9 - 1.9)
Bhutan	Klebsiella pneumoniae	64 (43 - 95)	12.1 (8.2 - 18.0)
Bhutan	Legionella spp.	4 (2 - 7)	0.7 (0.4 - 1.2)
Bhutan	Listeria monocytogenes	1 (1 - 2)	0.2 (0.1 - 0.3)
Bhutan	Morganella spp.	1 (0 - 1)	0.2 (0.1 - 0.2)
Bhutan	Mycoplasma spp.	9 (6 - 14)	1.6 (1.1 - 2.3)
Bhutan	Neisseria gonorrhoeae	0 (0 - 1)	0.1 (0.0 - 0.1)
Bhutan	Neisseria meningitidis	14 (9 - 22)	2.2 (1.4 - 3.4)
Bhutan	Non-typhoidal Salmonella	12 (7 - 19)	2.2 (1.3 - 3.5)
Bhutan	Other Klebsiella species	6 (3 - 12)	1.1 (0.5 - 2.2)
Bhutan	Other enterococci	10 (6 - 15)	2.0 (1.3 - 3.1)
Bhutan	Proteus spp.	11 (7 - 16)	2.2 (1.4 - 3.3)

Bhutan	Providencia spp.	1 (0 - 1)	0.2 (0.1 - 0.3)
Bhutan	Pseudomonas aeruginosa	46 (30 - 70)	9.0 (5.8 - 13.4)
Bhutan	Salmonella Paratyphi	4 (2 - 8)	0.5 (0.2 - 1.1)
Bhutan	Salmonella Typhi	28 (14 - 48)	4.0 (2.1 - 6.9)
Bhutan	Serratia spp.	11 (7 - 19)	2.1 (1.3 - 3.5)
Bhutan	Shigella spp.	6 (2 - 15)	1.3 (0.4 - 3.3)
Bhutan	Staphylococcus aureus	67 (46 - 100)	12.8 (8.9 - 18.9)
Bhutan	Streptococcus pneumoniae	63 (44 - 89)	11.5 (8.2 - 16.0)
Bhutan	Vibrio cholerae	1 (0 - 3)	0.2 (0.1 - 0.6)
India	Acinetobacter baumannii	104,935 (62,619 - 165,543)	11.0 (6.5 - 17.2)
India	Aeromonas spp.	3,065 (1,423 - 5,909)	0.4 (0.2 - 0.7)
India	Campylobacter spp.	44,308 (10,859 - 116,532)	5.3 (1.2 - 14.2)
India	Chlamydia spp.	29,123 (21,237 - 38,907)	2.8 (2.0 - 3.7)
India	Citrobacter spp.	12,082 (7,179 - 19,036)	1.2 (0.7 - 1.8)
India	Clostridioides difficile	1,349 (555 - 2,820)	0.1 (0.0 - 0.2)
India	Enterobacter spp.	62,961 (39,481 - 92,466)	6.0 (3.8 - 8.8)
India	Enterococcus faecalis	35,188 (21,442 - 53,369)	3.3 (2.0 - 5.0)
India	Enterococcus faecium	30,147 (17,855 - 47,785)	2.8 (1.7 - 4.5)
India	Escherichia coli	157,082 (115,649 - 207,650)	16.1 (11.8 - 21.6)
India	Group A Streptococcus	32,658 (16,837 - 58,520)	3.3 (1.7 - 5.9)
India	Group B Streptococcus	51,997 (37,805 - 70,581)	4.9 (3.5 - 6.8)
India	Haemophilus influenzae	18,427 (14,389 - 23,335)	1.7 (1.3 - 2.2)
India	Klebsiella pneumoniae	134,418 (93,816 - 185,596)	13.2 (9.2 - 18.3)
India	Legionella spp.	7,240 (4,360 - 11,716)	0.7 (0.4 - 1.1)
India	Listeria monocytogenes	2,298 (1,582 - 3,410)	0.2 (0.1 - 0.3)
India	Morganella spp.	1,364 (857 - 1,995)	0.1 (0.1 - 0.2)
India	Mycoplasma spp.	18,942 (14,826 - 24,303)	1.7 (1.3 - 2.2)
India	Neisseria gonorrhoeae	1,170 (726 - 1,507)	0.1 (0.1 - 0.1)
India	Neisseria meningitidis	28,335 (18,134 - 43,067)	2.4 (1.5 - 3.6)
India	Non-typhoidal Salmonella	39,545 (21,403 - 69,775)	3.8 (1.9 - 7.1)
India	Other Klebsiella species	10,383 (4,943 - 18,744)	0.9 (0.5 - 1.7)
India	Other enterococci	18,930 (12,407 - 27,584)	2.0 (1.3 - 2.9)
India	Proteus spp.	20,835 (13,545 - 30,401)	2.1 (1.4 - 3.1)
India	Providencia spp.	1,713 (1,071 - 2,687)	0.2 (0.1 - 0.3)
India	Pseudomonas aeruginosa	88,624 (60,074 - 124,450)	8.8 (5.9 - 12.3)
India	Salmonella Paratyphi	15,681 (6,419 - 30,652)	1.1 (0.5 - 2.2)
India	Salmonella Typhi	59,488 (32,156 - 97,581)	4.4 (2.4 - 7.2)
India	Serratia spp.	23,495 (13,945 - 36,884)	2.2 (1.3 - 3.5)
India	Shigella spp.	24,539 (8,927 - 57,255)	2.8 (1.0 - 6.8)

India	Staphylococcus aureus	130,643 (92,938 - 179,952)	12.8 (9.1 - 17.6)
India	Streptococcus pneumoniae	151,768 (120,422 - 191,723)	14.4 (11.4 - 18.3)
India	Vibrio cholerae	4,536 (1,710 - 9,972)	0.4 (0.2 - 1.0)
Nepal	Acinetobacter baumannii	1,708 (958 - 2,713)	9.4 (5.3 - 14.8)
Nepal	Aeromonas spp.	28 (12 - 59)	0.2 (0.1 - 0.4)
Nepal	Campylobacter spp.	747 (211 - 1,937)	4.5 (1.1 - 12.0)
Nepal	Chlamydia spp.	319 (232 - 441)	1.5 (1.1 - 2.1)
Nepal	Citrobacter spp.	182 (110 - 282)	0.9 (0.5 - 1.3)
Nepal	Clostridioides difficile	9 (3 - 22)	0.0 (0.0 - 0.1)
Nepal	Enterobacter spp.	1,017 (662 - 1,510)	5.0 (3.2 - 7.4)
Nepal	Enterococcus faecalis	939 (557 - 1,496)	4.5 (2.6 - 7.1)
Nepal	Enterococcus faecium	862 (512 - 1,417)	4.2 (2.5 - 6.9)
Nepal	Escherichia coli	3,693 (2,599 - 5,235)	18.9 (13.3 - 27.0)
Nepal	Group A Streptococcus	594 (286 - 1,115)	2.9 (1.3 - 5.8)
Nepal	Group B Streptococcus	1,111 (801 - 1,536)	5.1 (3.6 - 7.3)
Nepal	Haemophilus influenzae	400 (304 - 521)	1.9 (1.4 - 2.5)
Nepal	Klebsiella pneumoniae	3,933 (2,745 - 5,558)	19.5 (13.5 - 28.0)
Nepal	Legionella spp.	156 (101 - 235)	0.7 (0.5 - 1.1)
Nepal	Listeria monocytogenes	38 (25 - 59)	0.2 (0.1 - 0.3)
Nepal	Morganella spp.	26 (15 - 40)	0.1 (0.1 - 0.2)
Nepal	Mycoplasma spp.	237 (181 - 307)	1.0 (0.8 - 1.3)
Nepal	Neisseria gonorrhoeae	18 (12 - 28)	0.1 (0.0 - 0.1)
Nepal	Neisseria meningitidis	501 (308 - 767)	2.0 (1.2 - 3.0)
Nepal	Non-typhoidal Salmonella	428 (280 - 647)	1.8 (1.1 - 2.8)
Nepal	Other Klebsiella species	277 (134 - 505)	1.3 (0.6 - 2.4)
Nepal	Other enterococci	358 (229 - 538)	1.9 (1.2 - 2.8)
Nepal	Proteus spp.	506 (318 - 766)	2.7 (1.7 - 4.1)
Nepal	Providencia spp.	32 (18 - 51)	0.2 (0.1 - 0.3)
Nepal	Pseudomonas aeruginosa	2,391 (1,626 - 3,440)	12.0 (8.2 - 17.5)
Nepal	Salmonella Paratyphi	239 (95 - 486)	0.7 (0.3 - 1.5)
Nepal	Salmonella Typhi	1,297 (763 - 2,034)	4.3 (2.6 - 6.7)
Nepal	Serratia spp.	478 (290 - 773)	2.2 (1.3 - 3.7)
Nepal	Shigella spp.	181 (63 - 406)	1.0 (0.3 - 2.6)
Nepal	Staphylococcus aureus	4,443 (3,163 - 6,222)	22.9 (15.9 - 32.1)
Nepal	Streptococcus pneumoniae	3,154 (2,409 - 4,099)	15.0 (11.2 - 19.7)
Nepal	Vibrio cholerae	38 (15 - 81)	0.2 (0.1 - 0.4)
Pakistan	Acinetobacter baumannii	10,109 (5,827 - 16,004)	9.5 (5.3 - 15.4)
Pakistan	Aeromonas spp.	3,907 (1,858 - 6,956)	2.5 (1.2 - 4.7)
Pakistan	Campylobacter spp.	14,987 (5,338 - 30,169)	13.0 (3.5 - 31.2)

Pakistan	Chlamydia spp.	3,279 (2,254 - 4,584)	1.6 (1.1 - 2.2)
Pakistan	Citrobacter spp.	1,458 (848 - 2,254)	1.0 (0.6 - 1.6)
Pakistan	Clostridioides difficile	111 (35 - 275)	0.0 (0.0 - 0.1)
Pakistan	Enterobacter spp.	7,610 (4,955 - 11,135)	5.3 (3.4 - 8.0)
Pakistan	Enterococcus faecalis	7,004 (4,301 - 10,689)	5.0 (2.9 - 7.8)
Pakistan	Enterococcus faecium	5,312 (3,112 - 8,686)	4.4 (2.6 - 7.1)
Pakistan	Escherichia coli	32,094 (23,336 - 42,776)	24.3 (17.3 - 33.1)
Pakistan	Group A Streptococcus	5,861 (3,174 - 10,433)	4.2 (1.9 - 8.4)
Pakistan	Group B Streptococcus	15,676 (10,961 - 21,433)	7.5 (5.1 - 10.3)
Pakistan	Haemophilus influenzae	4,843 (3,633 - 6,331)	2.2 (1.7 - 2.9)
Pakistan	Klebsiella pneumoniae	36,277 (25,336 - 49,879)	23.4 (15.8 - 32.5)
Pakistan	Legionella spp.	1,378 (700 - 2,551)	0.6 (0.4 - 1.0)
Pakistan	Listeria monocytogenes	1,009 (683 - 1,491)	0.5 (0.3 - 0.7)
Pakistan	Morganella spp.	188 (107 - 299)	0.2 (0.1 - 0.3)
Pakistan	Mycoplasma spp.	2,511 (1,814 - 3,354)	1.1 (0.8 - 1.4)
Pakistan	Neisseria gonorrhoeae	105 (40 - 161)	0.1 (0.0 - 0.1)
Pakistan	Neisseria meningitidis	9,729 (6,357 - 14,407)	4.1 (2.7 - 6.1)
Pakistan	Non-typhoidal Salmonella	6,877 (4,520 - 10,050)	3.5 (2.2 - 5.3)
Pakistan	Other Klebsiella species	1,486 (660 - 2,941)	1.3 (0.6 - 2.6)
Pakistan	Other enterococci	3,056 (1,928 - 4,628)	2.4 (1.5 - 3.7)
Pakistan	Proteus spp.	3,262 (2,011 - 4,983)	3.2 (2.0 - 4.8)
Pakistan	Providencia spp.	278 (154 - 458)	0.3 (0.2 - 0.5)
Pakistan	Pseudomonas aeruginosa	18,780 (12,676 - 26,235)	12.9 (8.5 - 18.6)
Pakistan	Salmonella Paratyphi	5,425 (2,204 - 10,687)	2.2 (0.9 - 4.3)
Pakistan	Salmonella Typhi	25,295 (15,564 - 37,546)	9.8 (6.0 - 14.5)
Pakistan	Serratia spp.	4,596 (2,725 - 7,312)	3.0 (1.7 - 4.8)
Pakistan	Shigella spp.	6,660 (2,709 - 13,071)	4.7 (1.7 - 10.7)
Pakistan	Staphylococcus aureus	30,801 (22,247 - 41,241)	21.5 (15.3 - 29.4)
Pakistan	Streptococcus pneumoniae	31,840 (24,268 - 41,516)	16.6 (12.6 - 21.7)
Pakistan	Vibrio cholerae	10,455 (4,220 - 21,592)	9.0 (3.1 - 20.4)
China	Acinetobacter baumannii	112,621 (62,397 - 180,317)	6.4 (3.6 - 10.4)
China	Aeromonas spp.	216 (96 - 441)	0.0 (0.0 - 0.0)
China	Campylobacter spp.	236 (35 - 727)	0.0 (0.0 - 0.0)
China	Chlamydia spp.	9,528 (6,761 - 13,533)	0.7 (0.5 - 0.9)
China	Citrobacter spp.	9,775 (5,474 - 16,194)	0.6 (0.3 - 0.9)
China	Clostridioides difficile	4,252 (2,103 - 8,339)	0.2 (0.1 - 0.5)
China	Enterobacter spp.	76,360 (44,537 - 125,668)	4.3 (2.5 - 7.0)
China	Enterococcus faecalis	26,999 (14,610 - 44,852)	1.5 (0.8 - 2.5)
China	Enterococcus faecium	33,319 (19,112 - 53,235)	1.8 (1.0 - 2.9)
China	Escherichia coli	89,670 (55,253 - 139,629)	5.2 (3.2 - 8.0)
China	Group A Streptococcus	27,907 (11,007 - 59,380)	1.7 (0.7 - 3.4)

China	Group B Streptococcus	31,723 (19,004 - 49,667)	1.9 (1.2 - 3.0)
China	Haemophilus influenzae	7,087 (5,029 - 9,967)	0.5 (0.4 - 0.7)
China	Klebsiella pneumoniae	71,785 (45,486 - 110,930)	4.2 (2.7 - 6.5)
China	Legionella spp.	6,569 (4,601 - 9,312)	0.4 (0.3 - 0.6)
China	Listeria monocytogenes	681 (409 - 1,303)	0.0 (0.0 - 0.1)
China	Morganella spp.	548 (230 - 1,025)	0.0 (0.0 - 0.1)
China	Mycoplasma spp.	12,555 (9,288 - 17,121)	0.9 (0.7 - 1.2)
China	Neisseria gonorrhoeae	194 (144 - 240)	0.0 (0.0 - 0.0)
China	Neisseria meningitidis	7,962 (4,430 - 13,183)	0.5 (0.3 - 0.8)
China	Non-typhoidal Salmonella	6,138 (3,688 - 9,594)	0.4 (0.2 - 0.6)
China	Other Klebsiella species	5,725 (2,918 - 10,547)	0.3 (0.2 - 0.6)
China	Other enterococci	16,804 (9,491 - 26,453)	1.0 (0.5 - 1.5)
China	Proteus spp.	12,948 (7,764 - 20,125)	0.7 (0.4 - 1.1)
China	Providencia spp.	331 (135 - 632)	0.0 (0.0 - 0.0)
China	Pseudomonas aeruginosa	74,101 (45,331 - 115,179)	4.4 (2.7 - 6.7)
China	Salmonella Paratyphi	142 (57 - 290)	0.0 (0.0 - 0.0)
China	Salmonella Typhi	1,102 (631 - 1,729)	0.1 (0.0 - 0.1)
China	Serratia spp.	13,953 (7,970 - 23,038)	0.8 (0.4 - 1.3)
China	Shigella spp.	301 (118 - 643)	0.0 (0.0 - 0.1)
China	Staphylococcus aureus	145,711 (90,858 - 225,824)	8.5 (5.4 - 13.1)
China	Streptococcus pneumoniae	78,296 (55,839 - 110,279)	5.2 (3.9 - 7.2)
China	Vibrio cholerae	92 (39 - 203)	0.0 (0.0 - 0.0)
Democratic People's Republic of Korea	Acinetobacter baumannii	3,044 (1,762 - 4,937)	10.5 (6.1 - 17.0)
Democratic People's Republic of Korea	Aeromonas spp.	2 (1 - 4)	0.0 (0.0 - 0.0)
Democratic People's Republic of Korea	Campylobacter spp.	4 (1 - 12)	0.0 (0.0 - 0.0)
Democratic People's Republic of Korea	Chlamydia spp.	434 (305 - 599)	1.7 (1.2 - 2.4)
Democratic People's Republic of Korea	Citrobacter spp.	295 (163 - 488)	1.0 (0.6 - 1.7)
Democratic People's Republic of Korea	Clostridioides difficile	7 (2 - 16)	0.0 (0.0 - 0.1)
Democratic People's Republic of Korea	Enterobacter spp.	1,670 (982 - 2,664)	5.7 (3.4 - 9.1)
Democratic People's Republic of Korea	Enterococcus faecalis	731 (406 - 1,207)	2.4 (1.4 - 4.0)
Democratic People's Republic of Korea	Enterococcus faecium	814 (466 - 1,313)	2.7 (1.6 - 4.3)
Democratic People's Republic of Korea	Escherichia coli	2,503 (1,599 - 3,712)	8.7 (5.6 - 12.8)
Democratic People's Republic of Korea	Group A Streptococcus	640 (271 - 1,325)	2.3 (1.0 - 4.6)
Democratic People's Republic of Korea	Group B Streptococcus	688 (447 - 1,052)	2.6 (1.7 - 3.9)

Democratic People's Republic of Korea	Haemophilus influenzae	234 (169 - 318)	0.9 (0.7 - 1.3)
Democratic People's Republic of Korea	Klebsiella pneumoniae	2,455 (1,577 - 3,643)	8.7 (5.6 - 12.8)
Democratic People's Republic of Korea	Legionella spp.	166 (111 - 238)	0.7 (0.4 - 1.0)
Democratic People's Republic of Korea	Listeria monocytogenes	20 (11 - 36)	0.1 (0.0 - 0.1)
Democratic People's Republic of Korea	Morganella spp.	32 (14 - 57)	0.1 (0.0 - 0.2)
Democratic People's Republic of Korea	Mycoplasma spp.	326 (241 - 446)	1.3 (1.0 - 1.8)
Democratic People's Republic of Korea	Neisseria gonorrhoeae	5 (2 - 8)	0.0 (0.0 - 0.0)
Democratic People's Republic of Korea	Neisseria meningitidis	380 (214 - 654)	1.4 (0.8 - 2.3)
Democratic People's Republic of Korea	Non-typhoidal Salmonella	426 (244 - 711)	1.5 (0.9 - 2.5)
Democratic People's Republic of Korea	Other Klebsiella species	190 (92 - 348)	0.6 (0.3 - 1.2)
Democratic People's Republic of Korea	Other enterococci	459 (267 - 734)	1.6 (0.9 - 2.6)
Democratic People's Republic of Korea	Proteus spp.	439 (256 - 690)	1.5 (0.9 - 2.3)
Democratic People's Republic of Korea	Providencia spp.	33 (14 - 61)	0.1 (0.0 - 0.2)
Democratic People's Republic of Korea	Pseudomonas aeruginosa	2,038 (1,291 - 3,099)	7.2 (4.6 - 10.9)
Democratic People's Republic of Korea	Salmonella Paratyphi	3 (1 - 7)	0.0 (0.0 - 0.0)
Democratic People's Republic of Korea	Salmonella Typhi	74 (40 - 127)	0.3 (0.1 - 0.5)
Democratic People's Republic of Korea	Serratia spp.	511 (290 - 855)	1.7 (1.0 - 2.9)
Democratic People's Republic of Korea	Shigella spp.	6 (2 - 14)	0.0 (0.0 - 0.1)
Democratic People's Republic of Korea	Staphylococcus aureus	3,027 (1,987 - 4,463)	10.7 (7.1 - 15.6)
Democratic People's Republic of Korea	Streptococcus pneumoniae	2,474 (1,790 - 3,449)	9.4 (7.0 - 12.9)
Democratic People's Republic of Korea	Vibrio cholerae	180 (95 - 312)	0.8 (0.4 - 1.4)
Taiwan (Province of China)	Acinetobacter baumannii	1,019 (582 - 1,617)	2.7 (1.5 - 4.2)
Taiwan (Province of China)	Aeromonas spp.	1 (0 - 1)	0.0 (0.0 - 0.0)
Taiwan (Province of China)	Campylobacter spp.	3 (0 - 7)	0.0 (0.0 - 0.0)
Taiwan (Province of China)	Chlamydia spp.	215 (169 - 270)	0.5 (0.4 - 0.7)
Taiwan (Province of China)	Citrobacter spp.	147 (95 - 220)	0.4 (0.2 - 0.6)
Taiwan (Province of China)	Clostridioides difficile	113 (70 - 190)	0.3 (0.2 - 0.5)
Taiwan (Province of China)	Enterobacter spp.	972 (660 - 1,413)	2.5 (1.7 - 3.7)
Taiwan (Province of China)	Enterococcus faecalis	1,064 (657 - 1,673)	2.8 (1.7 - 4.3)
Taiwan (Province of China)	Enterococcus faecium	1,140 (690 - 1,765)	2.9 (1.8 - 4.6)
Taiwan (Province of China)	Escherichia coli	4,400 (3,037 - 6,265)	11.2 (7.7 - 16.1)
Taiwan (Province of China)	Group A Streptococcus	891 (514 - 1,551)	2.4 (1.4 - 4.1)

Taiwan (Province of China)	Group B Streptococcus	938 (630 - 1,370)	2.5 (1.6 - 3.7)
Taiwan (Province of China)	Haemophilus influenzae	272 (214 - 342)	0.7 (0.5 - 0.9)
Taiwan (Province of China)	Klebsiella pneumoniae	2,649 (1,847 - 3,760)	6.8 (4.7 - 9.7)
Taiwan (Province of China)	Legionella spp.	547 (431 - 682)	1.4 (1.1 - 1.7)
Taiwan (Province of China)	Listeria monocytogenes	13 (8 - 24)	0.0 (0.0 - 0.1)
Taiwan (Province of China)	Morganella spp.	34 (24 - 46)	0.1 (0.1 - 0.1)
Taiwan (Province of China)	Mycoplasma spp.	340 (266 - 429)	0.9 (0.7 - 1.1)
Taiwan (Province of China)	Neisseria gonorrhoeae	5 (4 - 7)	0.0 (0.0 - 0.0)
Taiwan (Province of China)	Neisseria meningitidis	81 (42 - 140)	0.3 (0.1 - 0.5)
Taiwan (Province of China)	Non-typhoidal Salmonella	101 (58 - 161)	0.4 (0.2 - 0.6)
Taiwan (Province of China)	Other Klebsiella species	211 (115 - 355)	0.6 (0.3 - 0.9)
Taiwan (Province of China)	Other enterococci	465 (323 - 645)	1.2 (0.8 - 1.7)
Taiwan (Province of China)	Proteus spp.	505 (349 - 713)	1.3 (0.9 - 1.8)
Taiwan (Province of China)	Providencia spp.	15 (10 - 21)	0.0 (0.0 - 0.1)
Taiwan (Province of China)	Pseudomonas aeruginosa	2,600 (1,864 - 3,640)	6.6 (4.7 - 9.4)
Taiwan (Province of China)	Salmonella Paratyphi	2 (1 - 5)	0.0 (0.0 - 0.0)
Taiwan (Province of China)	Salmonella Typhi	15 (8 - 24)	0.1 (0.0 - 0.1)
Taiwan (Province of China)	Serratia spp.	219 (127 - 348)	0.6 (0.3 - 0.9)
Taiwan (Province of China)	Shigella spp.	1 (0 - 2)	0.0 (0.0 - 0.0)
Taiwan (Province of China)	Staphylococcus aureus	7,136 (5,196 - 9,940)	18.1 (13.1 - 25.4)
Taiwan (Province of China)	Streptococcus pneumoniae	2,381 (1,833 - 3,060)	6.1 (4.7 - 7.9)
Taiwan (Province of China)	Vibrio cholerae	2 (1 - 5)	0.0 (0.0 - 0.0)
American Samoa	Acinetobacter baumannii	4 (2 - 6)	9.6 (5.5 - 15.0)
American Samoa	Aeromonas spp.	0 (0 - 0)	0.0 (0.0 - 0.1)
American Samoa	Campylobacter spp.	0 (0 - 1)	0.7 (0.1 - 1.9)
American Samoa	Chlamydia spp.	0 (0 - 1)	1.0 (0.6 - 1.6)
American Samoa	Citrobacter spp.	1 (0 - 1)	1.2 (0.7 - 1.8)
American Samoa	Clostridioides difficile	0 (0 - 0)	0.3 (0.1 - 0.6)
American Samoa	Enterobacter spp.	4 (2 - 5)	8.0 (5.0 - 12.1)
American Samoa	Enterococcus faecalis	1 (1 - 2)	3.3 (2.0 - 5.1)
American Samoa	Enterococcus faecium	1 (1 - 2)	3.1 (1.9 - 4.8)
American Samoa	Escherichia coli	5 (4 - 7)	13.1 (9.2 - 17.9)
American Samoa	Group A Streptococcus	2 (2 - 4)	5.6 (3.5 - 8.8)
American Samoa	Group B Streptococcus	3 (2 - 5)	7.0 (4.3 - 10.8)
American Samoa	Haemophilus influenzae	1 (0 - 1)	1.2 (0.8 - 1.8)
American Samoa	Klebsiella pneumoniae	5 (3 - 7)	11.0 (7.3 - 15.8)
American Samoa	Legionella spp.	0 (0 - 0)	0.1 (0.1 - 0.3)
American Samoa	Listeria monocytogenes	0 (0 - 0)	0.1 (0.1 - 0.1)
American Samoa	Morganella spp.	0 (0 - 0)	0.2 (0.1 - 0.4)
American Samoa	Mycoplasma spp.	0 (0 - 1)	1.0 (0.7 - 1.3)
American Samoa	Neisseria gonorrhoeae	0 (0 - 0)	0.0 (0.0 - 0.0)

American Samoa	Neisseria meningitidis	1 (0 - 1)	1.6 (0.9 - 2.6)
American Samoa	Non-typhoidal Salmonella	1 (0 - 1)	1.8 (1.0 - 2.8)
American Samoa	Other Klebsiella species	0 (0 - 1)	0.7 (0.4 - 1.3)
American Samoa	Other enterococci	1 (1 - 2)	2.6 (1.8 - 3.9)
American Samoa	Proteus spp.	1 (1 - 1)	2.5 (1.7 - 3.5)
American Samoa	Providencia spp.	0 (0 - 0)	0.2 (0.1 - 0.3)
American Samoa	Pseudomonas aeruginosa	4 (2 - 6)	9.2 (5.9 - 13.5)
American Samoa	Salmonella Paratyphi	0 (0 - 0)	0.0 (0.0 - 0.0)
American Samoa	Salmonella Typhi	0 (0 - 1)	0.7 (0.4 - 1.2)
American Samoa	Serratia spp.	1 (1 - 1)	2.0 (1.2 - 3.2)
American Samoa	Shigella spp.	0 (0 - 1)	0.6 (0.2 - 1.4)
American Samoa	Staphylococcus aureus	5 (3 - 7)	11.5 (7.7 - 16.6)
American Samoa	Streptococcus pneumoniae	5 (4 - 7)	11.9 (8.4 - 16.5)
American Samoa	Vibrio cholerae	0 (0 - 0)	0.1 (0.0 - 0.2)
Cook Islands	Acinetobacter baumannii	2 (1 - 3)	9.1 (5.2 - 14.3)
Cook Islands	Aeromonas spp.	0 (0 - 0)	0.0 (0.0 - 0.0)
Cook Islands	Campylobacter spp.	0 (0 - 0)	0.3 (0.0 - 0.7)
Cook Islands	Chlamydia spp.	0 (0 - 0)	1.6 (1.2 - 2.1)
Cook Islands	Citrobacter spp.	0 (0 - 0)	0.7 (0.4 - 1.2)
Cook Islands	Clostridioides difficile	0 (0 - 0)	0.2 (0.1 - 0.4)
Cook Islands	Enterobacter spp.	1 (1 - 2)	5.7 (3.4 - 8.6)
Cook Islands	Enterococcus faecalis	0 (0 - 1)	2.0 (1.1 - 3.2)
Cook Islands	Enterococcus faecium	1 (0 - 1)	2.3 (1.4 - 3.5)
Cook Islands	Escherichia coli	2 (1 - 2)	7.2 (4.7 - 10.4)
Cook Islands	Group A Streptococcus	1 (0 - 1)	2.6 (1.3 - 5.0)
Cook Islands	Group B Streptococcus	1 (0 - 1)	2.6 (1.7 - 3.8)
Cook Islands	Haemophilus influenzae	0 (0 - 0)	1.0 (0.7 - 1.3)
Cook Islands	Klebsiella pneumoniae	2 (1 - 2)	6.6 (4.5 - 9.4)
Cook Islands	Legionella spp.	0 (0 - 0)	0.8 (0.6 - 1.1)
Cook Islands	Listeria monocytogenes	0 (0 - 0)	0.0 (0.0 - 0.1)
Cook Islands	Morganella spp.	0 (0 - 0)	0.1 (0.0 - 0.1)
Cook Islands	Mycoplasma spp.	0 (0 - 1)	2.0 (1.5 - 2.6)
Cook Islands	Neisseria gonorrhoeae	0 (0 - 0)	0.0 (0.0 - 0.0)
Cook Islands	Neisseria meningitidis	0 (0 - 0)	0.7 (0.4 - 1.1)
Cook Islands	Non-typhoidal Salmonella	0 (0 - 0)	0.6 (0.4 - 1.0)
Cook Islands	Other Klebsiella species	0 (0 - 0)	0.5 (0.2 - 0.8)
Cook Islands	Other enterococci	0 (0 - 0)	1.3 (0.7 - 2.0)
Cook Islands	Proteus spp.	0 (0 - 0)	1.1 (0.7 - 1.6)
Cook Islands	Providencia spp.	0 (0 - 0)	0.0 (0.0 - 0.1)
Cook Islands	Pseudomonas aeruginosa	2 (1 - 2)	6.8 (4.6 - 9.8)

Cook Islands	Salmonella Paratyphi	0 (0 - 0)	0.0 (0.0 - 0.0)
Cook Islands	Salmonella Typhi	0 (0 - 0)	0.3 (0.1 - 0.8)
Cook Islands	Serratia spp.	0 (0 - 0)	1.1 (0.6 - 1.7)
Cook Islands	Shigella spp.	0 (0 - 0)	0.2 (0.1 - 0.5)
Cook Islands	Staphylococcus aureus	3 (2 - 4)	11.4 (8.0 - 15.8)
Cook Islands	Streptococcus pneumoniae	2 (2 - 3)	10.4 (8.1 - 13.5)
Cook Islands	Vibrio cholerae	0 (0 - 0)	0.0 (0.0 - 0.0)
Fiji	Acinetobacter baumannii	76 (40 - 127)	12.8 (6.8 - 21.2)
Fiji	Aeromonas spp.	0 (0 - 1)	0.1 (0.0 - 0.2)
Fiji	Campylobacter spp.	9 (2 - 22)	1.7 (0.4 - 4.5)
Fiji	Chlamydia spp.	8 (5 - 11)	1.2 (0.8 - 1.8)
Fiji	Citrobacter spp.	5 (3 - 8)	0.8 (0.4 - 1.2)
Fiji	Clostridioides difficile	2 (1 - 3)	0.2 (0.1 - 0.4)
Fiji	Enterobacter spp.	30 (18 - 48)	4.8 (2.9 - 7.4)
Fiji	Enterococcus faecalis	24 (13 - 40)	3.7 (2.1 - 6.1)
Fiji	Enterococcus faecium	26 (14 - 42)	4.0 (2.3 - 6.4)
Fiji	Escherichia coli	103 (63 - 155)	17.4 (10.8 - 26.0)
Fiji	Group A Streptococcus	36 (18 - 69)	5.9 (2.9 - 11.1)
Fiji	Group B Streptococcus	35 (22 - 52)	5.3 (3.3 - 7.9)
Fiji	Haemophilus influenzae	11 (7 - 16)	1.6 (1.1 - 2.3)
Fiji	Klebsiella pneumoniae	115 (72 - 172)	18.8 (11.9 - 27.7)
Fiji	Legionella spp.	4 (3 - 7)	0.7 (0.4 - 1.0)
Fiji	Listeria monocytogenes	1 (1 - 1)	0.1 (0.1 - 0.2)
Fiji	Morganella spp.	1 (1 - 2)	0.2 (0.1 - 0.4)
Fiji	Mycoplasma spp.	7 (5 - 10)	1.0 (0.7 - 1.3)
Fiji	Neisseria gonorrhoeae	1 (0 - 1)	0.1 (0.0 - 0.1)
Fiji	Neisseria meningitidis	16 (9 - 27)	2.0 (1.1 - 3.3)
Fiji	Non-typhoidal Salmonella	11 (6 - 18)	1.6 (0.9 - 2.5)
Fiji	Other Klebsiella species	5 (2 - 9)	0.7 (0.3 - 1.4)
Fiji	Other enterococci	14 (8 - 23)	2.5 (1.5 - 4.1)
Fiji	Proteus spp.	16 (9 - 26)	2.8 (1.7 - 4.5)
Fiji	Providencia spp.	2 (1 - 3)	0.3 (0.1 - 0.5)
Fiji	Pseudomonas aeruginosa	81 (50 - 126)	13.5 (8.3 - 20.4)
Fiji	Salmonella Paratyphi	0 (0 - 0)	0.0 (0.0 - 0.0)
Fiji	Salmonella Typhi	15 (9 - 23)	1.8 (1.1 - 2.8)
Fiji	Serratia spp.	15 (8 - 25)	2.3 (1.3 - 3.8)
Fiji	Shigella spp.	12 (5 - 23)	1.9 (0.7 - 3.8)
Fiji	Staphylococcus aureus	136 (90 - 202)	22.4 (14.9 - 32.8)
Fiji	Streptococcus pneumoniae	92 (63 - 129)	13.6 (9.5 - 19.0)
Fiji	Vibrio cholerae	1 (1 - 3)	0.2 (0.1 - 0.5)

Guam	Acinetobacter baumannii	10 (6 - 16)	5.6 (3.2 - 8.8)
Guam	Aeromonas spp.	0 (0 - 0)	0.0 (0.0 - 0.0)
Guam	Campylobacter spp.	1 (0 - 1)	0.3 (0.1 - 0.7)
Guam	Chlamydia spp.	1 (1 - 2)	0.7 (0.4 - 1.0)
Guam	Citrobacter spp.	1 (1 - 2)	0.8 (0.4 - 1.2)
Guam	Clostridioides difficile	1 (0 - 1)	0.4 (0.2 - 0.8)
Guam	Enterobacter spp.	10 (6 - 15)	5.4 (3.4 - 8.1)
Guam	Enterococcus faecalis	4 (2 - 6)	2.1 (1.3 - 3.3)
Guam	Enterococcus faecium	4 (2 - 6)	2.1 (1.2 - 3.2)
Guam	Escherichia coli	14 (9 - 19)	7.5 (5.0 - 10.6)
Guam	Group A Streptococcus	5 (3 - 8)	2.8 (1.8 - 4.4)
Guam	Group B Streptococcus	8 (5 - 11)	4.2 (2.6 - 6.4)
Guam	Haemophilus influenzae	1 (1 - 2)	0.8 (0.6 - 1.1)
Guam	Klebsiella pneumoniae	11 (7 - 16)	6.1 (4.0 - 8.8)
Guam	Legionella spp.	0 (0 - 0)	0.1 (0.1 - 0.3)
Guam	Listeria monocytogenes	0 (0 - 0)	0.1 (0.0 - 0.1)
Guam	Morganella spp.	0 (0 - 0)	0.1 (0.1 - 0.1)
Guam	Mycoplasma spp.	1 (1 - 2)	0.9 (0.6 - 1.2)
Guam	Neisseria gonorrhoeae	0 (0 - 0)	0.0 (0.0 - 0.0)
Guam	Neisseria meningitidis	2 (1 - 3)	1.1 (0.7 - 1.7)
Guam	Non-typhoidal Salmonella	1 (1 - 2)	0.8 (0.5 - 1.3)
Guam	Other Klebsiella species	1 (0 - 2)	0.5 (0.3 - 0.9)
Guam	Other enterococci	2 (2 - 3)	1.3 (0.9 - 1.9)
Guam	Proteus spp.	2 (1 - 3)	1.2 (0.8 - 1.7)
Guam	Providencia spp.	0 (0 - 0)	0.1 (0.0 - 0.1)
Guam	Pseudomonas aeruginosa	10 (6 - 14)	5.3 (3.4 - 7.8)
Guam	Salmonella Paratyphi	0 (0 - 0)	0.0 (0.0 - 0.0)
Guam	Salmonella Typhi	1 (0 - 2)	0.4 (0.2 - 0.9)
Guam	Serratia spp.	2 (1 - 3)	1.1 (0.7 - 1.7)
Guam	Shigella spp.	0 (0 - 1)	0.3 (0.1 - 0.6)
Guam	Staphylococcus aureus	13 (9 - 19)	7.2 (4.7 - 10.5)
Guam	Streptococcus pneumoniae	14 (10 - 19)	7.8 (5.6 - 10.6)
Guam	Vibrio cholerae	0 (0 - 0)	0.1 (0.0 - 0.2)
Kiribati	Acinetobacter baumannii	12 (6 - 20)	22.7 (12.1 - 37.3)
Kiribati	Aeromonas spp.	0 (0 - 0)	0.4 (0.2 - 0.8)
Kiribati	Campylobacter spp.	3 (1 - 8)	7.7 (1.4 - 21.3)
Kiribati	Chlamydia spp.	2 (1 - 2)	2.1 (1.5 - 3.0)
Kiribati	Citrobacter spp.	1 (0 - 1)	1.4 (0.8 - 2.2)
Kiribati	Clostridioides difficile	0 (0 - 0)	0.0 (0.0 - 0.1)
Kiribati	Enterobacter spp.	5 (3 - 8)	7.8 (5.0 - 11.9)

Kiribati	Enterococcus faecalis	4 (2 - 6)	5.9 (3.4 - 9.7)
Kiribati	Enterococcus faecium	4 (2 - 6)	5.8 (3.3 - 9.6)
Kiribati	Escherichia coli	17 (11 - 24)	27.7 (19.3 - 39.3)
Kiribati	Group A Streptococcus	5 (3 - 8)	7.7 (3.9 - 14.0)
Kiribati	Group B Streptococcus	8 (6 - 11)	10.0 (6.7 - 14.1)
Kiribati	Haemophilus influenzae	2 (2 - 3)	2.8 (2.0 - 3.8)
Kiribati	Klebsiella pneumoniae	20 (14 - 29)	31.4 (21.2 - 45.2)
Kiribati	Legionella spp.	0 (0 - 1)	0.5 (0.3 - 0.8)
Kiribati	Listeria monocytogenes	1 (0 - 1)	0.6 (0.4 - 0.8)
Kiribati	Morganella spp.	0 (0 - 0)	0.4 (0.2 - 0.8)
Kiribati	Mycoplasma spp.	1 (1 - 1)	1.2 (0.9 - 1.5)
Kiribati	Neisseria gonorrhoeae	0 (0 - 0)	0.1 (0.1 - 0.2)
Kiribati	Neisseria meningitidis	6 (4 - 9)	5.5 (3.6 - 8.2)
Kiribati	Non-typhoidal Salmonella	4 (2 - 6)	4.7 (2.9 - 7.4)
Kiribati	Other Klebsiella species	1 (0 - 2)	1.8 (0.7 - 3.5)
Kiribati	Other enterococci	2 (1 - 3)	3.6 (2.2 - 5.7)
Kiribati	Proteus spp.	2 (1 - 4)	4.8 (3.0 - 7.5)
Kiribati	Providencia spp.	0 (0 - 1)	0.7 (0.3 - 1.4)
Kiribati	Pseudomonas aeruginosa	11 (7 - 16)	18.3 (11.8 - 27.4)
Kiribati	Salmonella Paratyphi	0 (0 - 0)	0.0 (0.0 - 0.1)
Kiribati	Salmonella Typhi	6 (4 - 8)	5.6 (3.5 - 8.3)
Kiribati	Serratia spp.	3 (2 - 4)	3.9 (2.3 - 6.4)
Kiribati	Shigella spp.	4 (2 - 8)	7.2 (2.4 - 16.3)
Kiribati	Staphylococcus aureus	18 (13 - 26)	29.2 (20.5 - 41.2)
Kiribati	Streptococcus pneumoniae	18 (14 - 24)	22.7 (17.0 - 30.3)
Kiribati	Vibrio cholerae	1 (0 - 2)	1.6 (0.7 - 3.7)
Marshall Islands	Acinetobacter baumannii	4 (2 - 7)	15.7 (9.0 - 25.9)
Marshall Islands	Aeromonas spp.	0 (0 - 0)	0.1 (0.0 - 0.2)
Marshall Islands	Campylobacter spp.	0 (0 - 1)	2.0 (0.4 - 5.8)
Marshall Islands	Chlamydia spp.	1 (0 - 1)	2.1 (1.5 - 3.0)
Marshall Islands	Citrobacter spp.	0 (0 - 1)	1.0 (0.6 - 1.6)
Marshall Islands	Clostridioides difficile	0 (0 - 0)	0.1 (0.0 - 0.2)
Marshall Islands	Enterobacter spp.	2 (1 - 3)	6.8 (4.4 - 10.4)
Marshall Islands	Enterococcus faecalis	1 (1 - 2)	4.2 (2.3 - 7.1)
Marshall Islands	Enterococcus faecium	1 (1 - 2)	4.2 (2.4 - 7.0)
Marshall Islands	Escherichia coli	6 (4 - 8)	20.0 (13.6 - 29.1)
Marshall Islands	Group A Streptococcus	1 (1 - 2)	4.7 (2.5 - 8.8)
Marshall Islands	Group B Streptococcus	3 (2 - 4)	8.8 (6.0 - 12.7)
Marshall Islands	Haemophilus influenzae	1 (1 - 1)	2.6 (1.9 - 3.6)
Marshall Islands	Klebsiella pneumoniae	7 (5 - 10)	23.0 (15.3 - 33.9)
Marshall Islands	Legionella spp.	0 (0 - 0)	0.5 (0.4 - 0.8)

Marshall Islands	Listeria monocytogenes	0 (0 - 0)	0.1 (0.1 - 0.2)
Marshall Islands	Morganella spp.	0 (0 - 0)	0.3 (0.1 - 0.5)
Marshall Islands	Mycoplasma spp.	1 (0 - 1)	1.4 (1.1 - 1.9)
Marshall Islands	Neisseria gonorrhoeae	0 (0 - 0)	0.0 (0.0 - 0.1)
Marshall Islands	Neisseria meningitidis	1 (1 - 2)	2.5 (1.5 - 4.1)
Marshall Islands	Non-typhoidal Salmonella	1 (1 - 1)	2.5 (1.5 - 4.0)
Marshall Islands	Other Klebsiella species	0 (0 - 1)	1.2 (0.5 - 2.3)
Marshall Islands	Other enterococci	1 (0 - 1)	2.5 (1.5 - 4.0)
Marshall Islands	Proteus spp.	1 (0 - 1)	3.1 (1.9 - 4.8)
Marshall Islands	Providencia spp.	0 (0 - 0)	0.4 (0.2 - 0.7)
Marshall Islands	Pseudomonas aeruginosa	4 (3 - 6)	15.3 (10.0 - 22.6)
Marshall Islands	Salmonella Paratyphi	0 (0 - 0)	0.0 (0.0 - 0.0)
Marshall Islands	Salmonella Typhi	1 (1 - 2)	2.3 (1.4 - 3.5)
Marshall Islands	Serratia spp.	1 (0 - 1)	2.7 (1.5 - 4.4)
Marshall Islands	Shigella spp.	0 (0 - 1)	1.8 (0.6 - 4.1)
Marshall Islands	Staphylococcus aureus	7 (5 - 10)	24.0 (16.7 - 33.9)
Marshall Islands	Streptococcus pneumoniae	8 (6 - 11)	21.9 (16.3 - 29.4)
Marshall Islands	Vibrio cholerae	0 (0 - 0)	0.6 (0.2 - 1.4)
Micronesia (Federated States of)	Acinetobacter baumannii	8 (4 - 13)	13.8 (7.8 - 22.8)
Micronesia (Federated States of)	Aeromonas spp.	0 (0 - 0)	0.1 (0.0 - 0.2)
Micronesia (Federated States of)	Campylobacter spp.	1 (0 - 2)	1.7 (0.3 - 4.9)
Micronesia (Federated States of)	Chlamydia spp.	1 (1 - 2)	1.9 (1.3 - 2.7)
Micronesia (Federated States of)	Citrobacter spp.	1 (0 - 1)	1.1 (0.7 - 1.8)
Micronesia (Federated States of)	Clostridioides difficile	0 (0 - 0)	0.1 (0.0 - 0.2)
Micronesia (Federated States of)	Enterobacter spp.	5 (3 - 7)	7.9 (5.0 - 12.3)
Micronesia (Federated States of)	Enterococcus faecalis	3 (2 - 5)	4.7 (2.7 - 7.9)
Micronesia (Federated States of)	Enterococcus faecium	3 (2 - 5)	4.9 (2.8 - 7.9)
Micronesia (Federated States of)	Escherichia coli	12 (8 - 17)	21.4 (14.3 - 30.9)
Micronesia (Federated States of)	Group A Streptococcus	3 (2 - 6)	5.7 (2.9 - 10.4)
Micronesia (Federated States of)	Group B Streptococcus	6 (4 - 8)	9.2 (6.2 - 13.4)
Micronesia (Federated States of)	Haemophilus influenzae	2 (1 - 2)	2.5 (1.8 - 3.4)
Micronesia (Federated States of)	Klebsiella pneumoniae	13 (9 - 20)	23.2 (15.5 - 33.6)
Micronesia (Federated States of)	Legionella spp.	0 (0 - 1)	0.6 (0.4 - 0.8)
Micronesia (Federated States of)	Listeria monocytogenes	0 (0 - 0)	0.1 (0.1 - 0.2)
Micronesia (Federated States of)	Morganella spp.	0 (0 - 0)	0.3 (0.1 - 0.5)
Micronesia (Federated States of)	Mycoplasma spp.	1 (1 - 2)	1.5 (1.0 - 2.0)
Micronesia (Federated States of)	Neisseria gonorrhoeae	0 (0 - 0)	0.0 (0.0 - 0.1)
Micronesia (Federated States of)	Neisseria meningitidis	2 (1 - 4)	2.6 (1.6 - 4.2)
Micronesia (Federated States of)	Non-typhoidal Salmonella	2 (1 - 3)	2.3 (1.4 - 3.7)
Micronesia (Federated States of)	Other Klebsiella species	1 (0 - 1)	1.2 (0.5 - 2.2)

Micronesia (Federated States of)	Other enterococci	2 (1 - 2)	2.9 (1.8 - 4.7)
Micronesia (Federated States of)	Proteus spp.	2 (1 - 3)	3.4 (2.1 - 5.2)
Micronesia (Federated States of)	Providencia spp.	0 (0 - 0)	0.4 (0.2 - 0.6)
Micronesia (Federated States of)	Pseudomonas aeruginosa	10 (6 - 14)	16.6 (10.6 - 24.8)
Micronesia (Federated States of)	Salmonella Paratyphi	0 (0 - 0)	0.0 (0.0 - 0.1)
Micronesia (Federated States of)	Salmonella Typhi	2 (1 - 3)	2.2 (1.4 - 3.3)
Micronesia (Federated States of)	Serratia spp.	2 (1 - 3)	2.9 (1.7 - 4.8)
Micronesia (Federated States of)	Shigella spp.	1 (0 - 2)	1.5 (0.5 - 3.4)
Micronesia (Federated States of)	Staphylococcus aureus	15 (10 - 21)	25.5 (17.7 - 36.3)
Micronesia (Federated States of)	Streptococcus pneumoniae	15 (11 - 20)	22.4 (16.5 - 30.4)
Micronesia (Federated States of)	Vibrio cholerae	0 (0 - 0)	0.3 (0.1 - 0.7)
Nauru	Acinetobacter baumannii	1 (0 - 1)	23.9 (13.8 - 38.6)
Nauru	Aeromonas spp.	0 (0 - 0)	0.1 (0.0 - 0.1)
Nauru	Campylobacter spp.	0 (0 - 0)	1.0 (0.2 - 2.8)
Nauru	Chlamydia spp.	0 (0 - 0)	3.8 (2.8 - 5.0)
Nauru	Citrobacter spp.	0 (0 - 0)	1.7 (1.0 - 2.8)
Nauru	Clostridioides difficile	0 (0 - 0)	0.2 (0.1 - 0.4)
Nauru	Enterobacter spp.	0 (0 - 1)	10.0 (6.2 - 15.6)
Nauru	Enterococcus faecalis	0 (0 - 0)	4.4 (2.5 - 7.3)
Nauru	Enterococcus faecium	0 (0 - 0)	4.5 (2.6 - 7.4)
Nauru	Escherichia coli	1 (0 - 1)	16.6 (11.1 - 24.2)
Nauru	Group A Streptococcus	0 (0 - 0)	5.4 (2.7 - 10.2)
Nauru	Group B Streptococcus	0 (0 - 0)	5.1 (3.5 - 7.7)
Nauru	Haemophilus influenzae	0 (0 - 0)	2.2 (1.7 - 2.9)
Nauru	Klebsiella pneumoniae	1 (1 - 1)	16.7 (11.5 - 24.4)
Nauru	Legionella spp.	0 (0 - 0)	1.2 (0.9 - 1.7)
Nauru	Listeria monocytogenes	0 (0 - 0)	0.2 (0.1 - 0.2)
Nauru	Morganella spp.	0 (0 - 0)	0.2 (0.1 - 0.4)
Nauru	Mycoplasma spp.	0 (0 - 0)	3.4 (2.6 - 4.5)
Nauru	Neisseria gonorrhoeae	0 (0 - 0)	0.0 (0.0 - 0.0)
Nauru	Neisseria meningitidis	0 (0 - 0)	2.8 (1.7 - 4.6)
Nauru	Non-typhoidal Salmonella	0 (0 - 0)	2.8 (1.6 - 4.5)
Nauru	Other Klebsiella species	0 (0 - 0)	1.1 (0.5 - 2.2)
Nauru	Other enterococci	0 (0 - 0)	3.0 (1.8 - 4.6)
Nauru	Proteus spp.	0 (0 - 0)	2.8 (1.8 - 4.4)
Nauru	Providencia spp.	0 (0 - 0)	0.2 (0.1 - 0.4)
Nauru	Pseudomonas aeruginosa	1 (0 - 1)	14.2 (9.3 - 21.1)
Nauru	Salmonella Paratyphi	0 (0 - 0)	0.0 (0.0 - 0.0)
Nauru	Salmonella Typhi	0 (0 - 0)	1.0 (0.6 - 1.6)
Nauru	Serratia spp.	0 (0 - 0)	3.0 (1.7 - 4.9)

Nauru	Shigella spp.	0 (0 - 0)	1.0 (0.4 - 2.2)
Nauru	Staphylococcus aureus	1 (1 - 1)	20.4 (14.6 - 28.9)
Nauru	Streptococcus pneumoniae	1 (1 - 2)	21.7 (16.9 - 28.7)
Nauru	Vibrio cholerae	0 (0 - 0)	0.2 (0.1 - 0.4)
Niue	Acinetobacter baumannii	0 (0 - 0)	7.2 (4.1 - 11.6)
Niue	Aeromonas spp.	0 (0 - 0)	0.0 (0.0 - 0.1)
Niue	Campylobacter spp.	0 (0 - 0)	0.6 (0.1 - 1.8)
Niue	Chlamydia spp.	0 (0 - 0)	1.1 (0.8 - 1.5)
Niue	Citrobacter spp.	0 (0 - 0)	0.7 (0.4 - 1.0)
Niue	Clostridioides difficile	0 (0 - 0)	0.5 (0.2 - 0.8)
Niue	Enterobacter spp.	0 (0 - 0)	4.6 (2.9 - 6.9)
Niue	Enterococcus faecalis	0 (0 - 0)	3.4 (2.0 - 5.4)
Niue	Enterococcus faecium	0 (0 - 0)	3.9 (2.3 - 6.1)
Niue	Escherichia coli	0 (0 - 0)	14.6 (9.6 - 21.3)
Niue	Group A Streptococcus	0 (0 - 0)	4.2 (2.3 - 7.5)
Niue	Group B Streptococcus	0 (0 - 0)	4.5 (3.0 - 6.6)
Niue	Haemophilus influenzae	0 (0 - 0)	1.5 (1.1 - 2.0)
Niue	Klebsiella pneumoniae	0 (0 - 0)	13.4 (8.9 - 19.3)
Niue	Legionella spp.	0 (0 - 0)	1.0 (0.7 - 1.4)
Niue	Listeria monocytogenes	0 (0 - 0)	0.1 (0.0 - 0.1)
Niue	Morganella spp.	0 (0 - 0)	0.1 (0.1 - 0.2)
Niue	Mycoplasma spp.	0 (0 - 0)	1.4 (1.0 - 1.9)
Niue	Neisseria gonorrhoeae	0 (0 - 0)	0.0 (0.0 - 0.0)
Niue	Neisseria meningitidis	0 (0 - 0)	1.2 (0.7 - 2.0)
Niue	Non-typhoidal Salmonella	0 (0 - 0)	0.6 (0.4 - 1.0)
Niue	Other Klebsiella species	0 (0 - 0)	0.7 (0.4 - 1.3)
Niue	Other enterococci	0 (0 - 0)	1.8 (1.1 - 2.8)
Niue	Proteus spp.	0 (0 - 0)	1.9 (1.2 - 3.0)
Niue	Providencia spp.	0 (0 - 0)	0.1 (0.0 - 0.2)
Niue	Pseudomonas aeruginosa	0 (0 - 0)	11.4 (7.5 - 16.8)
Niue	Salmonella Paratyphi	0 (0 - 0)	0.0 (0.0 - 0.0)
Niue	Salmonella Typhi	0 (0 - 0)	0.8 (0.4 - 1.3)
Niue	Serratia spp.	0 (0 - 0)	1.5 (0.9 - 2.4)
Niue	Shigella spp.	0 (0 - 0)	0.6 (0.2 - 1.3)
Niue	Staphylococcus aureus	0 (0 - 1)	22.7 (16.0 - 32.1)
Niue	Streptococcus pneumoniae	0 (0 - 0)	13.1 (9.8 - 17.4)
Niue	Vibrio cholerae	0 (0 - 0)	0.0 (0.0 - 0.1)
Northern Mariana Islands	Acinetobacter baumannii	4 (2 - 6)	8.8 (5.1 - 14.5)
Northern Mariana Islands	Aeromonas spp.	0 (0 - 0)	0.0 (0.0 - 0.1)
Northern Mariana Islands	Campylobacter spp.	0 (0 - 0)	0.6 (0.1 - 1.6)

Northern Mariana Islands	Chlamydia spp.	0 (0 - 1)	0.9 (0.6 - 1.4)
Northern Mariana Islands	Citrobacter spp.	0 (0 - 1)	1.1 (0.6 - 1.7)
Northern Mariana Islands	Clostridioides difficile	0 (0 - 0)	0.4 (0.2 - 0.8)
Northern Mariana Islands	Enterobacter spp.	4 (2 - 5)	8.2 (5.2 - 12.3)
Northern Mariana Islands	Enterococcus faecalis	2 (1 - 2)	3.5 (2.2 - 5.5)
Northern Mariana Islands	Enterococcus faecium	2 (1 - 2)	3.5 (2.2 - 5.3)
Northern Mariana Islands	Escherichia coli	5 (3 - 7)	12.7 (9.0 - 17.5)
Northern Mariana Islands	Group A Streptococcus	2 (1 - 3)	4.8 (3.1 - 7.5)
Northern Mariana Islands	Group B Streptococcus	2 (1 - 3)	5.6 (3.6 - 8.5)
Northern Mariana Islands	Haemophilus influenzae	0 (0 - 1)	1.1 (0.8 - 1.5)
Northern Mariana Islands	Klebsiella pneumoniae	4 (2 - 5)	9.0 (6.0 - 12.9)
Northern Mariana Islands	Legionella spp.	0 (0 - 0)	0.2 (0.1 - 0.4)
Northern Mariana Islands	Listeria monocytogenes	0 (0 - 0)	0.1 (0.0 - 0.1)
Northern Mariana Islands	Morganella spp.	0 (0 - 0)	0.1 (0.1 - 0.2)
Northern Mariana Islands	Mycoplasma spp.	0 (0 - 1)	1.2 (0.9 - 1.7)
Northern Mariana Islands	Neisseria gonorrhoeae	0 (0 - 0)	0.1 (0.0 - 0.1)
Northern Mariana Islands	Neisseria meningitidis	0 (0 - 1)	1.0 (0.6 - 1.6)
Northern Mariana Islands	Non-typhoidal Salmonella	0 (0 - 1)	0.8 (0.5 - 1.3)
Northern Mariana Islands	Other Klebsiella species	0 (0 - 1)	0.9 (0.5 - 1.4)
Northern Mariana Islands	Other enterococci	1 (0 - 1)	2.1 (1.4 - 3.0)
Northern Mariana Islands	Proteus spp.	1 (1 - 1)	2.0 (1.4 - 2.7)
Northern Mariana Islands	Providencia spp.	0 (0 - 0)	0.1 (0.1 - 0.1)
Northern Mariana Islands	Pseudomonas aeruginosa	3 (2 - 5)	8.3 (5.3 - 12.3)
Northern Mariana Islands	Salmonella Paratyphi	0 (0 - 0)	0.0 (0.0 - 0.0)
Northern Mariana Islands	Salmonella Typhi	0 (0 - 0)	0.4 (0.2 - 0.9)
Northern Mariana Islands	Serratia spp.	1 (0 - 1)	1.5 (0.9 - 2.3)
Northern Mariana Islands	Shigella spp.	0 (0 - 0)	0.5 (0.2 - 1.2)
Northern Mariana Islands	Staphylococcus aureus	5 (3 - 7)	12.0 (8.1 - 17.6)
Northern Mariana Islands	Streptococcus pneumoniae	4 (3 - 6)	10.5 (7.7 - 14.2)
Northern Mariana Islands	Vibrio cholerae	0 (0 - 0)	0.1 (0.0 - 0.2)
Palau	Acinetobacter baumannii	2 (1 - 3)	8.9 (5.1 - 14.6)
Palau	Aeromonas spp.	0 (0 - 0)	0.0 (0.0 - 0.1)
Palau	Campylobacter spp.	0 (0 - 0)	1.0 (0.2 - 2.9)
Palau	Chlamydia spp.	0 (0 - 0)	2.4 (1.9 - 3.1)
Palau	Citrobacter spp.	0 (0 - 0)	0.7 (0.4 - 1.2)
Palau	Clostridioides difficile	0 (0 - 0)	0.5 (0.3 - 0.8)
Palau	Enterobacter spp.	1 (1 - 2)	6.2 (4.2 - 9.3)
Palau	Enterococcus faecalis	1 (0 - 1)	3.6 (2.0 - 6.2)
Palau	Enterococcus faecium	1 (0 - 1)	4.3 (2.4 - 7.0)
Palau	Escherichia coli	3 (2 - 5)	18.5 (12.6 - 27.2)
Palau	Group A Streptococcus	1 (0 - 1)	3.7 (1.6 - 7.2)

Palau	Group B Streptococcus	1 (1 - 2)	7.2 (5.2 - 9.9)
Palau	Haemophilus influenzae	0 (0 - 1)	3.1 (2.5 - 3.9)
Palau	Klebsiella pneumoniae	3 (2 - 5)	19.2 (13.8 - 27.2)
Palau	Legionella spp.	0 (0 - 0)	2.2 (1.7 - 2.9)
Palau	Listeria monocytogenes	0 (0 - 0)	0.1 (0.1 - 0.2)
Palau	Morganella spp.	0 (0 - 0)	0.1 (0.1 - 0.2)
Palau	Mycoplasma spp.	1 (0 - 1)	3.3 (2.6 - 4.1)
Palau	Neisseria gonorrhoeae	0 (0 - 0)	0.0 (0.0 - 0.0)
Palau	Neisseria meningitidis	0 (0 - 0)	1.4 (0.8 - 2.4)
Palau	Non-typhoidal Salmonella	0 (0 - 0)	0.7 (0.4 - 1.2)
Palau	Other Klebsiella species	0 (0 - 0)	0.7 (0.3 - 1.4)
Palau	Other enterococci	0 (0 - 1)	1.9 (1.1 - 3.1)
Palau	Proteus spp.	0 (0 - 1)	2.0 (1.2 - 3.3)
Palau	Providencia spp.	0 (0 - 0)	0.1 (0.0 - 0.2)
Palau	Pseudomonas aeruginosa	3 (2 - 4)	16.7 (11.7 - 23.7)
Palau	Salmonella Paratyphi	0 (0 - 0)	0.1 (0.0 - 0.1)
Palau	Salmonella Typhi	0 (0 - 0)	1.8 (0.9 - 3.1)
Palau	Serratia spp.	0 (0 - 1)	1.7 (1.0 - 2.9)
Palau	Shigella spp.	0 (0 - 0)	0.9 (0.3 - 2.1)
Palau	Staphylococcus aureus	6 (5 - 8)	38.4 (28.9 - 51.6)
Palau	Streptococcus pneumoniae	4 (3 - 6)	27.4 (21.7 - 34.4)
Palau	Vibrio cholerae	0 (0 - 0)	0.0 (0.0 - 0.1)
Papua New Guinea	Acinetobacter baumannii	577 (326 - 926)	14.1 (7.7 - 23.0)
Papua New Guinea	Aeromonas spp.	16 (7 - 31)	0.3 (0.1 - 0.6)
Papua New Guinea	Campylobacter spp.	241 (70 - 540)	5.0 (1.1 - 12.6)
Papua New Guinea	Chlamydia spp.	223 (147 - 314)	2.5 (1.7 - 3.7)
Papua New Guinea	Citrobacter spp.	67 (38 - 108)	1.0 (0.6 - 1.6)
Papua New Guinea	Clostridioides difficile	3 (1 - 6)	0.0 (0.0 - 0.1)
Papua New Guinea	Enterobacter spp.	409 (266 - 615)	6.6 (4.1 - 10.1)
Papua New Guinea	Enterococcus faecalis	178 (105 - 282)	3.1 (1.7 - 5.2)
Papua New Guinea	Enterococcus faecium	130 (72 - 213)	2.6 (1.4 - 4.3)
Papua New Guinea	Escherichia coli	1,073 (758 - 1,503)	16.9 (11.5 - 23.7)
Papua New Guinea	Group A Streptococcus	260 (149 - 447)	5.1 (2.6 - 9.4)
Papua New Guinea	Group B Streptococcus	1,173 (788 - 1,628)	12.7 (8.3 - 18.2)
Papua New Guinea	Haemophilus influenzae	371 (268 - 500)	3.6 (2.6 - 4.9)
Papua New Guinea	Klebsiella pneumoniae	1,490 (1,050 - 2,056)	21.7 (14.6 - 30.9)
Papua New Guinea	Legionella spp.	34 (17 - 64)	0.4 (0.2 - 0.6)
Papua New Guinea	Listeria monocytogenes	30 (18 - 46)	0.3 (0.2 - 0.4)
Papua New Guinea	Morganella spp.	5 (2 - 9)	0.1 (0.1 - 0.3)
Papua New Guinea	Mycoplasma spp.	198 (139 - 270)	1.8 (1.2 - 2.4)
Papua New Guinea	Neisseria gonorrhoeae	2 (1 - 5)	0.0 (0.0 - 0.1)

Papua New Guinea	Neisseria meningitidis	483 (314 - 724)	4.4 (2.8 - 6.6)
Papua New Guinea	Non-typhoidal Salmonella	367 (233 - 545)	4.5 (2.7 - 7.0)
Papua New Guinea	Other Klebsiella species	33 (14 - 68)	0.6 (0.3 - 1.3)
Papua New Guinea	Other enterococci	103 (62 - 162)	2.1 (1.2 - 3.5)
Papua New Guinea	Proteus spp.	94 (56 - 149)	2.3 (1.4 - 3.7)
Papua New Guinea	Providencia spp.	8 (4 - 15)	0.2 (0.1 - 0.4)
Papua New Guinea	Pseudomonas aeruginosa	793 (531 - 1,122)	13.3 (8.4 - 19.5)
Papua New Guinea	Salmonella Paratyphi	12 (5 - 28)	0.1 (0.0 - 0.2)
Papua New Guinea	Salmonella Typhi	560 (336 - 860)	5.3 (3.2 - 8.0)
Papua New Guinea	Serratia spp.	168 (98 - 265)	2.7 (1.5 - 4.5)
Papua New Guinea	Shigella spp.	430 (171 - 817)	5.6 (2.2 - 11.2)
Papua New Guinea	Staphylococcus aureus	1,149 (812 - 1,594)	18.5 (12.6 - 26.8)
Papua New Guinea	Streptococcus pneumoniae	2,683 (1,964 - 3,580)	28.2 (20.3 - 37.9)
Papua New Guinea	Vibrio cholerae	67 (32 - 131)	1.4 (0.6 - 2.7)
Samoa	Acinetobacter baumannii	16 (9 - 25)	12.1 (6.9 - 18.8)
Samoa	Aeromonas spp.	0 (0 - 0)	0.0 (0.0 - 0.1)
Samoa	Campylobacter spp.	1 (0 - 3)	0.8 (0.1 - 2.2)
Samoa	Chlamydia spp.	3 (2 - 4)	1.9 (1.3 - 2.6)
Samoa	Citrobacter spp.	2 (1 - 3)	1.2 (0.7 - 2.0)
Samoa	Clostridioides difficile	0 (0 - 0)	0.1 (0.0 - 0.2)
Samoa	Enterobacter spp.	10 (6 - 16)	7.3 (4.3 - 11.1)
Samoa	Enterococcus faecalis	4 (3 - 7)	3.2 (1.8 - 5.2)
Samoa	Enterococcus faecium	5 (3 - 7)	3.3 (1.9 - 5.2)
Samoa	Escherichia coli	16 (11 - 24)	12.2 (8.1 - 17.5)
Samoa	Group A Streptococcus	5 (3 - 9)	3.8 (2.0 - 7.0)
Samoa	Group B Streptococcus	6 (4 - 9)	4.5 (3.0 - 6.6)
Samoa	Haemophilus influenzae	2 (1 - 3)	1.4 (1.0 - 1.9)
Samoa	Klebsiella pneumoniae	16 (10 - 23)	11.8 (7.7 - 17.0)
Samoa	Legionella spp.	1 (0 - 1)	0.4 (0.3 - 0.6)
Samoa	Listeria monocytogenes	0 (0 - 0)	0.1 (0.1 - 0.1)
Samoa	Morganella spp.	0 (0 - 0)	0.2 (0.1 - 0.3)
Samoa	Mycoplasma spp.	3 (2 - 4)	1.6 (1.1 - 2.1)
Samoa	Neisseria gonorrhoeae	0 (0 - 0)	0.0 (0.0 - 0.0)
Samoa	Neisseria meningitidis	3 (2 - 5)	1.8 (1.0 - 2.9)
Samoa	Non-typhoidal Salmonella	3 (2 - 5)	2.0 (1.2 - 3.3)
Samoa	Other Klebsiella species	1 (1 - 2)	0.8 (0.4 - 1.5)
Samoa	Other enterococci	3 (2 - 4)	2.2 (1.3 - 3.4)
Samoa	Proteus spp.	3 (2 - 4)	2.1 (1.4 - 3.3)
Samoa	Providencia spp.	0 (0 - 0)	0.2 (0.1 - 0.3)
Samoa	Pseudomonas aeruginosa	13 (8 - 19)	9.5 (6.1 - 14.3)

Samoa	Salmonella Paratyphi	0 (0 - 0)	0.0 (0.0 - 0.0)
Samoa	Salmonella Typhi	2 (1 - 3)	0.9 (0.5 - 1.5)
Samoa	Serratia spp.	3 (2 - 5)	2.1 (1.2 - 3.5)
Samoa	Shigella spp.	1 (0 - 2)	0.7 (0.2 - 1.6)
Samoa	Staphylococcus aureus	18 (12 - 26)	13.0 (8.8 - 18.7)
Samoa	Streptococcus pneumoniae	21 (15 - 28)	13.9 (10.2 - 18.7)
Samoa	Vibrio cholerae	0 (0 - 0)	0.2 (0.1 - 0.4)
Solomon Islands	Acinetobacter baumannii	55 (32 - 90)	20.9 (11.9 - 33.7)
Solomon Islands	Aeromonas spp.	1 (0 - 2)	0.4 (0.1 - 0.7)
Solomon Islands	Campylobacter spp.	17 (4 - 43)	7.8 (1.5 - 20.8)
Solomon Islands	Chlamydia spp.	12 (9 - 16)	3.4 (2.6 - 4.5)
Solomon Islands	Citrobacter spp.	4 (2 - 6)	1.0 (0.6 - 1.6)
Solomon Islands	Clostridioides difficile	0 (0 - 0)	0.0 (0.0 - 0.1)
Solomon Islands	Enterobacter spp.	24 (17 - 36)	7.4 (5.0 - 11.0)
Solomon Islands	Enterococcus faecalis	19 (12 - 29)	5.0 (3.0 - 8.1)
Solomon Islands	Enterococcus faecium	16 (10 - 27)	5.0 (2.9 - 8.2)
Solomon Islands	Escherichia coli	82 (59 - 115)	27.2 (19.4 - 37.8)
Solomon Islands	Group A Streptococcus	22 (13 - 37)	6.3 (3.3 - 11.4)
Solomon Islands	Group B Streptococcus	39 (29 - 50)	10.2 (7.6 - 13.6)
Solomon Islands	Haemophilus influenzae	16 (12 - 20)	4.2 (3.3 - 5.2)
Solomon Islands	Klebsiella pneumoniae	111 (83 - 151)	33.5 (24.7 - 45.9)
Solomon Islands	Legionella spp.	6 (4 - 9)	1.7 (1.3 - 2.3)
Solomon Islands	Listeria monocytogenes	1 (1 - 2)	0.2 (0.1 - 0.3)
Solomon Islands	Morganella spp.	1 (0 - 1)	0.3 (0.1 - 0.5)
Solomon Islands	Mycoplasma spp.	13 (10 - 16)	2.6 (2.1 - 3.3)
Solomon Islands	Neisseria gonorrhoeae	0 (0 - 1)	0.1 (0.0 - 0.2)
Solomon Islands	Neisseria meningitidis	23 (15 - 35)	3.6 (2.3 - 5.7)
Solomon Islands	Non-typhoidal Salmonella	14 (9 - 21)	2.9 (1.8 - 4.6)
Solomon Islands	Other Klebsiella species	4 (2 - 8)	1.2 (0.5 - 2.3)
Solomon Islands	Other enterococci	8 (5 - 13)	2.8 (1.7 - 4.4)
Solomon Islands	Proteus spp.	9 (6 - 15)	3.4 (2.1 - 5.4)
Solomon Islands	Providencia spp.	1 (0 - 2)	0.4 (0.2 - 0.6)
Solomon Islands	Pseudomonas aeruginosa	70 (50 - 97)	22.2 (15.8 - 31.0)
Solomon Islands	Salmonella Paratyphi	0 (0 - 0)	0.0 (0.0 - 0.1)
Solomon Islands	Salmonella Typhi	22 (15 - 32)	3.5 (2.2 - 5.1)
Solomon Islands	Serratia spp.	12 (7 - 19)	3.1 (1.8 - 5.1)
Solomon Islands	Shigella spp.	23 (9 - 44)	7.1 (2.5 - 15.5)
Solomon Islands	Staphylococcus aureus	144 (111 - 189)	47.9 (37.4 - 61.6)
Solomon Islands	Streptococcus pneumoniae	138 (111 - 170)	35.3 (29.0 - 43.2)
Solomon Islands	Vibrio cholerae	6 (3 - 12)	2.0 (0.9 - 4.2)

Tokelau	Acinetobacter baumannii	0 (0 - 0)	8.6 (4.7 - 13.8)
Tokelau	Aeromonas spp.	0 (0 - 0)	0.0 (0.0 - 0.1)
Tokelau	Campylobacter spp.	0 (0 - 0)	0.8 (0.1 - 2.3)
Tokelau	Chlamydia spp.	0 (0 - 0)	1.1 (0.7 - 1.6)
Tokelau	Citrobacter spp.	0 (0 - 0)	1.0 (0.6 - 1.7)
Tokelau	Clostridioides difficile	0 (0 - 0)	0.1 (0.0 - 0.2)
Tokelau	Enterobacter spp.	0 (0 - 0)	7.4 (4.4 - 11.5)
Tokelau	Enterococcus faecalis	0 (0 - 0)	2.8 (1.6 - 4.7)
Tokelau	Enterococcus faecium	0 (0 - 0)	2.9 (1.7 - 4.6)
Tokelau	Escherichia coli	0 (0 - 0)	10.9 (7.1 - 15.8)
Tokelau	Group A Streptococcus	0 (0 - 0)	3.5 (1.8 - 6.4)
Tokelau	Group B Streptococcus	0 (0 - 0)	6.3 (3.9 - 9.8)
Tokelau	Haemophilus influenzae	0 (0 - 0)	1.3 (0.9 - 1.8)
Tokelau	Klebsiella pneumoniae	0 (0 - 0)	9.9 (6.4 - 14.6)
Tokelau	Legionella spp.	0 (0 - 0)	0.2 (0.1 - 0.3)
Tokelau	Listeria monocytogenes	0 (0 - 0)	0.1 (0.0 - 0.1)
Tokelau	Morganella spp.	0 (0 - 0)	0.1 (0.1 - 0.2)
Tokelau	Mycoplasma spp.	0 (0 - 0)	1.1 (0.7 - 1.5)
Tokelau	Neisseria gonorrhoeae	0 (0 - 0)	0.0 (0.0 - 0.0)
Tokelau	Neisseria meningitidis	0 (0 - 0)	1.4 (0.8 - 2.3)
Tokelau	Non-typhoidal Salmonella	0 (0 - 0)	1.6 (0.9 - 2.5)
Tokelau	Other Klebsiella species	0 (0 - 0)	0.7 (0.4 - 1.3)
Tokelau	Other enterococci	0 (0 - 0)	1.9 (1.1 - 2.9)
Tokelau	Proteus spp.	0 (0 - 0)	1.8 (1.1 - 2.8)
Tokelau	Providencia spp.	0 (0 - 0)	0.1 (0.1 - 0.2)
Tokelau	Pseudomonas aeruginosa	0 (0 - 0)	8.2 (5.1 - 12.5)
Tokelau	Salmonella Paratyphi	0 (0 - 0)	0.0 (0.0 - 0.0)
Tokelau	Salmonella Typhi	0 (0 - 0)	0.7 (0.3 - 1.1)
Tokelau	Serratia spp.	0 (0 - 0)	1.8 (1.0 - 2.9)
Tokelau	Shigella spp.	0 (0 - 0)	0.8 (0.3 - 1.8)
Tokelau	Staphylococcus aureus	0 (0 - 0)	10.6 (6.8 - 15.7)
Tokelau	Streptococcus pneumoniae	0 (0 - 0)	12.6 (8.9 - 17.4)
Tokelau	Vibrio cholerae	0 (0 - 0)	0.2 (0.1 - 0.5)
Tonga	Acinetobacter baumannii	7 (4 - 11)	9.1 (5.3 - 14.5)
Tonga	Aeromonas spp.	0 (0 - 0)	0.0 (0.0 - 0.0)
Tonga	Campylobacter spp.	0 (0 - 1)	0.3 (0.1 - 0.9)
Tonga	Chlamydia spp.	1 (1 - 1)	1.2 (0.9 - 1.7)
Tonga	Citrobacter spp.	1 (0 - 1)	0.8 (0.5 - 1.3)
Tonga	Clostridioides difficile	0 (0 - 0)	0.1 (0.0 - 0.2)
Tonga	Enterobacter spp.	4 (3 - 6)	5.3 (3.4 - 7.7)

Tonga	Enterococcus faecalis	3 (2 - 5)	3.9 (2.3 - 6.2)
Tonga	Enterococcus faecium	3 (2 - 5)	4.1 (2.5 - 6.4)
Tonga	Escherichia coli	13 (9 - 19)	16.6 (11.3 - 23.4)
Tonga	Group A Streptococcus	3 (2 - 6)	3.9 (2.1 - 6.9)
Tonga	Group B Streptococcus	4 (3 - 6)	5.3 (3.7 - 7.5)
Tonga	Haemophilus influenzae	1 (1 - 2)	1.7 (1.2 - 2.2)
Tonga	Klebsiella pneumoniae	14 (9 - 19)	16.8 (11.4 - 23.4)
Tonga	Legionella spp.	0 (0 - 1)	0.6 (0.4 - 0.8)
Tonga	Listeria monocytogenes	0 (0 - 0)	0.3 (0.2 - 0.4)
Tonga	Morganella spp.	0 (0 - 0)	0.2 (0.1 - 0.3)
Tonga	Mycoplasma spp.	1 (1 - 1)	1.0 (0.8 - 1.4)
Tonga	Neisseria gonorrhoeae	0 (0 - 0)	0.0 (0.0 - 0.0)
Tonga	Neisseria meningitidis	2 (1 - 3)	2.1 (1.4 - 3.1)
Tonga	Non-typhoidal Salmonella	1 (1 - 2)	1.3 (0.8 - 2.0)
Tonga	Other Klebsiella species	1 (0 - 1)	1.0 (0.5 - 1.8)
Tonga	Other enterococci	2 (1 - 2)	2.1 (1.3 - 3.1)
Tonga	Proteus spp.	2 (1 - 3)	2.5 (1.6 - 3.7)
Tonga	Providencia spp.	0 (0 - 0)	0.2 (0.1 - 0.3)
Tonga	Pseudomonas aeruginosa	9 (6 - 14)	11.9 (7.9 - 17.1)
Tonga	Salmonella Paratyphi	0 (0 - 0)	0.0 (0.0 - 0.0)
Tonga	Salmonella Typhi	1 (1 - 2)	1.1 (0.7 - 1.7)
Tonga	Serratia spp.	2 (1 - 3)	2.1 (1.2 - 3.2)
Tonga	Shigella spp.	0 (0 - 1)	0.3 (0.1 - 0.7)
Tonga	Staphylococcus aureus	16 (12 - 22)	20.3 (14.7 - 27.9)
Tonga	Streptococcus pneumoniae	12 (9 - 16)	14.2 (10.7 - 18.6)
Tonga	Vibrio cholerae	0 (0 - 1)	0.5 (0.2 - 1.1)
Tuvalu	Acinetobacter baumannii	1 (1 - 2)	15.7 (8.9 - 25.7)
Tuvalu	Aeromonas spp.	0 (0 - 0)	0.1 (0.0 - 0.2)
Tuvalu	Campylobacter spp.	0 (0 - 0)	1.3 (0.2 - 3.7)
Tuvalu	Chlamydia spp.	0 (0 - 0)	2.4 (1.7 - 3.5)
Tuvalu	Citrobacter spp.	0 (0 - 0)	1.3 (0.8 - 2.2)
Tuvalu	Clostridioides difficile	0 (0 - 0)	0.1 (0.1 - 0.3)
Tuvalu	Enterobacter spp.	1 (0 - 1)	7.9 (4.7 - 12.6)
Tuvalu	Enterococcus faecalis	0 (0 - 1)	3.5 (2.0 - 6.0)
Tuvalu	Enterococcus faecium	0 (0 - 1)	3.6 (2.1 - 5.8)
Tuvalu	Escherichia coli	1 (1 - 2)	13.8 (9.1 - 20.0)
Tuvalu	Group A Streptococcus	0 (0 - 1)	4.3 (2.2 - 8.1)
Tuvalu	Group B Streptococcus	0 (0 - 1)	5.3 (3.5 - 8.1)
Tuvalu	Haemophilus influenzae	0 (0 - 0)	1.7 (1.2 - 2.4)
Tuvalu	Klebsiella pneumoniae	1 (1 - 2)	14.1 (9.1 - 21.2)
Tuvalu	Legionella spp.	0 (0 - 0)	0.5 (0.3 - 0.7)

Tuvalu	Listeria monocytogenes	0 (0 - 0)	0.1 (0.1 - 0.2)
Tuvalu	Morganella spp.	0 (0 - 0)	0.2 (0.1 - 0.4)
Tuvalu	Mycoplasma spp.	0 (0 - 0)	1.9 (1.4 - 2.6)
Tuvalu	Neisseria gonorrhoeae	0 (0 - 0)	0.0 (0.0 - 0.1)
Tuvalu	Neisseria meningitidis	0 (0 - 0)	2.2 (1.3 - 3.7)
Tuvalu	Non-typhoidal Salmonella	0 (0 - 0)	2.7 (1.5 - 4.4)
Tuvalu	Other Klebsiella species	0 (0 - 0)	1.0 (0.4 - 1.9)
Tuvalu	Other enterococci	0 (0 - 0)	2.4 (1.5 - 3.8)
Tuvalu	Proteus spp.	0 (0 - 0)	2.4 (1.5 - 3.8)
Tuvalu	Providencia spp.	0 (0 - 0)	0.2 (0.1 - 0.4)
Tuvalu	Pseudomonas aeruginosa	1 (1 - 2)	11.2 (7.0 - 16.9)
Tuvalu	Salmonella Paratyphi	0 (0 - 0)	0.0 (0.0 - 0.0)
Tuvalu	Salmonella Typhi	0 (0 - 0)	1.0 (0.6 - 1.7)
Tuvalu	Serratia spp.	0 (0 - 0)	2.5 (1.4 - 4.3)
Tuvalu	Shigella spp.	0 (0 - 0)	1.2 (0.4 - 2.8)
Tuvalu	Staphylococcus aureus	1 (1 - 2)	14.7 (9.8 - 21.6)
Tuvalu	Streptococcus pneumoniae	2 (1 - 2)	17.1 (12.4 - 23.7)
Tuvalu	Vibrio cholerae	0 (0 - 0)	0.3 (0.1 - 0.7)
Vanuatu	Acinetobacter baumannii	26 (15 - 42)	17.9 (10.4 - 29.0)
Vanuatu	Aeromonas spp.	0 (0 - 1)	0.2 (0.1 - 0.4)
Vanuatu	Campylobacter spp.	5 (1 - 14)	4.0 (0.7 - 11.2)
Vanuatu	Chlamydia spp.	6 (4 - 8)	2.8 (1.9 - 4.0)
Vanuatu	Citrobacter spp.	3 (2 - 4)	1.5 (0.8 - 2.5)
Vanuatu	Clostridioides difficile	0 (0 - 0)	0.0 (0.0 - 0.1)
Vanuatu	Enterobacter spp.	14 (9 - 22)	8.1 (4.9 - 12.4)
Vanuatu	Enterococcus faecalis	7 (4 - 11)	4.0 (2.2 - 6.6)
Vanuatu	Enterococcus faecium	6 (3 - 10)	3.6 (2.0 - 6.1)
Vanuatu	Escherichia coli	26 (18 - 38)	16.1 (10.9 - 22.9)
Vanuatu	Group A Streptococcus	8 (5 - 15)	5.2 (2.7 - 9.7)
Vanuatu	Group B Streptococcus	17 (12 - 25)	8.1 (5.5 - 11.8)
Vanuatu	Haemophilus influenzae	5 (4 - 7)	2.3 (1.7 - 3.1)
Vanuatu	Klebsiella pneumoniae	31 (21 - 45)	18.2 (11.9 - 26.4)
Vanuatu	Legionella spp.	1 (0 - 1)	0.4 (0.2 - 0.6)
Vanuatu	Listeria monocytogenes	0 (0 - 0)	0.1 (0.1 - 0.2)
Vanuatu	Morganella spp.	0 (0 - 0)	0.2 (0.1 - 0.3)
Vanuatu	Mycoplasma spp.	4 (3 - 6)	1.7 (1.3 - 2.4)
Vanuatu	Neisseria gonorrhoeae	0 (0 - 0)	0.0 (0.0 - 0.1)
Vanuatu	Neisseria meningitidis	9 (6 - 14)	3.4 (2.1 - 5.4)
Vanuatu	Non-typhoidal Salmonella	11 (7 - 16)	5.2 (3.1 - 8.2)
Vanuatu	Other Klebsiella species	2 (1 - 4)	1.2 (0.5 - 2.4)

Vanuatu	Other enterococci	4 (2 - 6)	2.5 (1.5 - 4.0)
Vanuatu	Proteus spp.	4 (2 - 6)	2.9 (1.7 - 4.5)
Vanuatu	Providencia spp.	0 (0 - 1)	0.3 (0.1 - 0.5)
Vanuatu	Pseudomonas aeruginosa	20 (13 - 29)	12.1 (7.8 - 17.9)
Vanuatu	Salmonella Paratyphi	0 (0 - 0)	0.0 (0.0 - 0.1)
Vanuatu	Salmonella Typhi	6 (4 - 9)	2.2 (1.3 - 3.3)
Vanuatu	Serratia spp.	6 (3 - 9)	3.2 (1.8 - 5.4)
Vanuatu	Shigella spp.	6 (2 - 13)	3.8 (1.4 - 8.4)
Vanuatu	Staphylococcus aureus	26 (18 - 37)	15.5 (10.5 - 22.1)
Vanuatu	Streptococcus pneumoniae	46 (34 - 62)	21.3 (15.7 - 28.8)
Vanuatu	Vibrio cholerae	4 (2 - 8)	2.4 (1.0 - 5.4)
Cambodia	Acinetobacter baumannii	834 (515 - 1,283)	8.5 (5.2 - 13.0)
Cambodia	Aeromonas spp.	7 (3 - 13)	0.1 (0.0 - 0.2)
Cambodia	Campylobacter spp.	141 (37 - 363)	1.5 (0.3 - 4.0)
Cambodia	Chlamydia spp.	275 (212 - 354)	2.4 (1.9 - 3.0)
Cambodia	Citrobacter spp.	112 (66 - 177)	1.0 (0.6 - 1.5)
Cambodia	Clostridioides difficile	7 (2 - 15)	0.0 (0.0 - 0.1)
Cambodia	Enterobacter spp.	691 (476 - 1,004)	6.2 (4.3 - 8.8)
Cambodia	Enterococcus faecalis	640 (367 - 1,017)	5.6 (3.2 - 8.9)
Cambodia	Enterococcus faecium	634 (366 - 1,018)	5.6 (3.3 - 8.9)
Cambodia	Escherichia coli	2,648 (1,879 - 3,639)	24.5 (17.5 - 33.4)
Cambodia	Group A Streptococcus	425 (229 - 754)	4.1 (2.1 - 7.4)
Cambodia	Group B Streptococcus	827 (620 - 1,075)	6.9 (5.2 - 9.0)
Cambodia	Haemophilus influenzae	371 (302 - 453)	3.1 (2.5 - 3.7)
Cambodia	Klebsiella pneumoniae	2,816 (2,094 - 3,752)	25.7 (19.2 - 34.1)
Cambodia	Legionella spp.	181 (125 - 269)	1.6 (1.1 - 2.1)
Cambodia	Listeria monocytogenes	12 (6 - 24)	0.1 (0.0 - 0.2)
Cambodia	Morganella spp.	13 (7 - 22)	0.1 (0.1 - 0.2)
Cambodia	Mycoplasma spp.	251 (201 - 311)	1.9 (1.5 - 2.3)
Cambodia	Neisseria gonorrhoeae	3 (1 - 5)	0.0 (0.0 - 0.0)
Cambodia	Neisseria meningitidis	248 (151 - 409)	1.7 (1.0 - 2.7)
Cambodia	Non-typhoidal Salmonella	202 (135 - 305)	1.6 (1.0 - 2.4)
Cambodia	Other Klebsiella species	226 (109 - 403)	2.0 (0.9 - 3.5)
Cambodia	Other enterococci	185 (118 - 283)	1.8 (1.2 - 2.8)
Cambodia	Proteus spp.	320 (197 - 489)	3.2 (2.0 - 4.8)
Cambodia	Providencia spp.	16 (9 - 27)	0.2 (0.1 - 0.3)
Cambodia	Pseudomonas aeruginosa	1,770 (1,296 - 2,374)	16.4 (12.1 - 21.7)
Cambodia	Salmonella Paratyphi	32 (13 - 70)	0.2 (0.1 - 0.4)
Cambodia	Salmonella Typhi	464 (280 - 739)	2.9 (1.8 - 4.5)
Cambodia	Serratia spp.	262 (163 - 413)	2.2 (1.4 - 3.5)

Cambodia	Shigella spp.	41 (14 - 96)	0.4 (0.1 - 0.9)
Cambodia	Staphylococcus aureus	3,976 (3,134 - 4,986)	38.2 (30.3 - 47.1)
Cambodia	Streptococcus pneumoniae	2,766 (2,298 - 3,354)	23.6 (19.6 - 27.9)
Cambodia	Vibrio cholerae	86 (29 - 206)	0.7 (0.2 - 1.7)
Indonesia	Acinetobacter baumannii	20,461 (11,783 - 32,201)	12.4 (7.1 - 19.5)
Indonesia	Aeromonas spp.	128 (61 - 233)	0.1 (0.0 - 0.2)
Indonesia	Campylobacter spp.	3,285 (806 - 8,618)	2.3 (0.5 - 6.3)
Indonesia	Chlamydia spp.	2,999 (2,130 - 4,152)	1.8 (1.3 - 2.5)
Indonesia	Citrobacter spp.	2,526 (1,491 - 3,896)	1.4 (0.8 - 2.1)
Indonesia	Clostridioides difficile	630 (304 - 1,173)	0.3 (0.1 - 0.5)
Indonesia	Enterobacter spp.	13,311 (8,414 - 19,818)	7.5 (4.7 - 10.9)
Indonesia	Enterococcus faecalis	8,076 (4,768 - 12,580)	4.5 (2.6 - 7.3)
Indonesia	Enterococcus faecium	7,984 (4,861 - 12,278)	4.5 (2.8 - 6.8)
Indonesia	Escherichia coli	28,457 (20,277 - 38,498)	17.0 (12.4 - 23.0)
Indonesia	Group A Streptococcus	6,158 (3,125 - 11,446)	3.6 (1.8 - 6.7)
Indonesia	Group B Streptococcus	6,953 (4,707 - 10,128)	4.0 (2.7 - 5.8)
Indonesia	Haemophilus influenzae	2,238 (1,647 - 2,933)	1.3 (0.9 - 1.7)
Indonesia	Klebsiella pneumoniae	23,793 (16,158 - 33,389)	14.0 (9.6 - 19.6)
Indonesia	Legionella spp.	705 (450 - 1,091)	0.4 (0.3 - 0.6)
Indonesia	Listeria monocytogenes	306 (206 - 464)	0.1 (0.1 - 0.2)
Indonesia	Morganella spp.	252 (136 - 415)	0.2 (0.1 - 0.3)
Indonesia	Mycoplasma spp.	2,155 (1,594 - 2,827)	1.2 (0.9 - 1.5)
Indonesia	Neisseria gonorrhoeae	51 (23 - 64)	0.0 (0.0 - 0.0)
Indonesia	Neisseria meningitidis	4,469 (2,809 - 6,774)	2.1 (1.3 - 3.1)
Indonesia	Non-typhoidal Salmonella	6,118 (3,556 - 10,176)	3.3 (1.8 - 5.8)
Indonesia	Other Klebsiella species	2,923 (1,485 - 5,019)	1.6 (0.8 - 2.9)
Indonesia	Other enterococci	3,313 (2,069 - 4,978)	2.0 (1.3 - 3.1)
Indonesia	Proteus spp.	4,497 (2,841 - 6,732)	2.8 (1.8 - 4.1)
Indonesia	Providencia spp.	306 (161 - 519)	0.2 (0.1 - 0.3)
Indonesia	Pseudomonas aeruginosa	16,493 (10,663 - 23,864)	9.8 (6.4 - 14.1)
Indonesia	Salmonella Paratyphi	451 (172 - 937)	0.2 (0.1 - 0.4)
Indonesia	Salmonella Typhi	6,911 (3,730 - 11,649)	2.8 (1.6 - 4.7)
Indonesia	Serratia spp.	4,409 (2,673 - 7,035)	2.4 (1.5 - 3.8)
Indonesia	Shigella spp.	2,430 (905 - 5,087)	1.6 (0.6 - 3.6)
Indonesia	Staphylococcus aureus	23,176 (15,436 - 32,655)	13.3 (9.1 - 18.5)
Indonesia	Streptococcus pneumoniae	19,336 (14,391 - 25,685)	11.0 (8.2 - 14.6)
Indonesia	Vibrio cholerae	740 (266 - 1,686)	0.4 (0.1 - 0.9)
Lao People's Democratic Republic	Acinetobacter baumannii	333 (194 - 527)	9.0 (5.2 - 14.3)
Lao People's Democratic Republic	Aeromonas spp.	3 (1 - 5)	0.1 (0.0 - 0.1)
Lao People's Democratic Republic	Campylobacter spp.	66 (19 - 158)	1.6 (0.4 - 4.0)

Lao People's Democratic Republic	Chlamydia spp.	111 (79 - 153)	2.1 (1.6 - 2.9)
Lao People's Democratic Republic	Citrobacter spp.	43 (26 - 67)	1.0 (0.6 - 1.5)
Lao People's Democratic Republic	Clostridioides difficile	2 (1 - 5)	0.0 (0.0 - 0.1)
Lao People's Democratic Republic	Enterobacter spp.	253 (169 - 383)	5.6 (3.8 - 8.5)
Lao People's Democratic Republic	Enterococcus faecalis	178 (105 - 282)	4.1 (2.3 - 6.6)
Lao People's Democratic Republic	Enterococcus faecium	159 (92 - 265)	3.8 (2.2 - 6.3)
Lao People's Democratic Republic	Escherichia coli	811 (572 - 1,131)	18.8 (13.4 - 26.1)
Lao People's Democratic Republic	Group A Streptococcus	181 (97 - 321)	4.5 (2.2 - 8.2)
Lao People's Democratic Republic	Group B Streptococcus	479 (342 - 655)	8.8 (6.3 - 12.0)
Lao People's Democratic Republic	Haemophilus influenzae	161 (120 - 213)	2.8 (2.2 - 3.6)
Lao People's Democratic Republic	Klebsiella pneumoniae	991 (699 - 1,375)	21.8 (15.6 - 30.3)
Lao People's Democratic Republic	Legionella spp.	32 (19 - 55)	0.6 (0.4 - 0.9)
Lao People's Democratic Republic	Listeria monocytogenes	10 (6 - 17)	0.1 (0.1 - 0.2)
Lao People's Democratic Republic	Morganella spp.	5 (3 - 9)	0.2 (0.1 - 0.3)
Lao People's Democratic Republic	Mycoplasma spp.	95 (70 - 125)	1.5 (1.1 - 2.0)
Lao People's Democratic Republic	Neisseria gonorrhoeae	1 (1 - 2)	0.0 (0.0 - 0.0)
Lao People's Democratic Republic	Neisseria meningitidis	192 (113 - 300)	2.9 (1.7 - 4.5)
Lao People's Democratic Republic	Non-typhoidal Salmonella	163 (103 - 254)	3.0 (1.8 - 4.7)
Lao People's Democratic Republic	Other Klebsiella species	48 (22 - 94)	1.1 (0.5 - 2.2)
Lao People's Democratic Republic	Other enterococci	81 (50 - 127)	2.1 (1.3 - 3.3)
Lao People's Democratic Republic	Proteus spp.	100 (60 - 154)	2.7 (1.7 - 4.2)
Lao People's Democratic Republic	Providencia spp.	8 (4 - 14)	0.2 (0.1 - 0.4)
Lao People's Democratic Republic	Pseudomonas aeruginosa	566 (396 - 805)	13.0 (8.9 - 18.5)
Lao People's Democratic Republic	Salmonella Paratyphi	15 (6 - 32)	0.2 (0.1 - 0.4)
Lao People's Democratic Republic	Salmonella Typhi	252 (155 - 393)	3.7 (2.2 - 5.6)
Lao People's Democratic Republic	Serratia spp.	117 (70 - 192)	2.6 (1.5 - 4.2)
Lao People's Democratic Republic	Shigella spp.	42 (14 - 97)	0.9 (0.3 - 1.9)
Lao People's Democratic Republic	Staphylococcus aureus	961 (705 - 1,310)	22.4 (16.7 - 30.6)
Lao People's Democratic Republic	Streptococcus pneumoniae	1,214 (917 - 1,595)	22.6 (17.5 - 29.0)
Lao People's Democratic Republic	Vibrio cholerae	235 (82 - 520)	4.9 (1.6 - 11.6)
Malaysia	Acinetobacter baumannii	2,292 (1,344 - 3,522)	9.8 (5.7 - 15.2)
Malaysia	Aeromonas spp.	3 (1 - 5)	0.0 (0.0 - 0.0)
Malaysia	Campylobacter spp.	89 (16 - 246)	0.4 (0.1 - 1.3)
Malaysia	Chlamydia spp.	737 (525 - 968)	3.3 (2.3 - 4.2)
Malaysia	Citrobacter spp.	208 (120 - 328)	0.9 (0.5 - 1.4)
Malaysia	Clostridioides difficile	204 (103 - 362)	0.7 (0.3 - 1.3)
Malaysia	Enterobacter spp.	1,529 (970 - 2,245)	6.3 (4.0 - 9.2)
Malaysia	Enterococcus faecalis	738 (423 - 1,203)	3.1 (1.7 - 5.2)
Malaysia	Enterococcus faecium	756 (462 - 1,138)	3.2 (2.0 - 4.7)
Malaysia	Escherichia coli	2,873 (1,968 - 4,044)	12.7 (8.8 - 17.8)
Malaysia	Group A Streptococcus	1,030 (652 - 1,555)	4.6 (2.9 - 6.9)

Malaysia	Group B Streptococcus	917 (639 - 1,253)	4.0 (2.7 - 5.4)
Malaysia	Haemophilus influenzae	420 (296 - 530)	1.8 (1.3 - 2.3)
Malaysia	Klebsiella pneumoniae	2,458 (1,734 - 3,363)	10.8 (7.7 - 14.5)
Malaysia	Legionella spp.	383 (258 - 495)	1.6 (1.1 - 2.1)
Malaysia	Listeria monocytogenes	22 (15 - 34)	0.1 (0.1 - 0.1)
Malaysia	Morganella spp.	26 (13 - 45)	0.1 (0.1 - 0.2)
Malaysia	Mycoplasma spp.	787 (558 - 995)	3.2 (2.2 - 4.0)
Malaysia	Neisseria gonorrhoeae	4 (3 - 6)	0.0 (0.0 - 0.0)
Malaysia	Neisseria meningitidis	211 (123 - 333)	0.7 (0.4 - 1.2)
Malaysia	Non-typhoidal Salmonella	188 (112 - 294)	0.7 (0.4 - 1.2)
Malaysia	Other Klebsiella species	202 (108 - 343)	0.8 (0.4 - 1.5)
Malaysia	Other enterococci	391 (242 - 577)	1.8 (1.1 - 2.6)
Malaysia	Proteus spp.	403 (261 - 583)	1.8 (1.2 - 2.6)
Malaysia	Providencia spp.	20 (10 - 33)	0.1 (0.0 - 0.1)
Malaysia	Pseudomonas aeruginosa	2,302 (1,633 - 3,142)	10.1 (7.2 - 13.7)
Malaysia	Salmonella Paratyphi	32 (12 - 69)	0.1 (0.0 - 0.2)
Malaysia	Salmonella Typhi	316 (136 - 598)	1.0 (0.5 - 1.9)
Malaysia	Serratia spp.	302 (178 - 477)	1.2 (0.7 - 1.9)
Malaysia	Shigella spp.	36 (12 - 82)	0.2 (0.1 - 0.4)
Malaysia	Staphylococcus aureus	4,063 (2,990 - 5,421)	17.8 (13.3 - 23.5)
Malaysia	Streptococcus pneumoniae	4,268 (3,048 - 5,398)	18.4 (13.1 - 22.9)
Malaysia	Vibrio cholerae	5 (2 - 12)	0.0 (0.0 - 0.1)
Maldives	Acinetobacter baumannii	19 (11 - 31)	6.9 (4.0 - 11.3)
Maldives	Aeromonas spp.	0 (0 - 0)	0.0 (0.0 - 0.0)
Maldives	Campylobacter spp.	1 (0 - 2)	0.3 (0.1 - 0.8)
Maldives	Chlamydia spp.	2 (2 - 3)	0.8 (0.6 - 1.2)
Maldives	Citrobacter spp.	2 (1 - 3)	0.6 (0.4 - 1.0)
Maldives	Clostridioides difficile	1 (0 - 1)	0.1 (0.1 - 0.3)
Maldives	Enterobacter spp.	13 (8 - 21)	4.4 (2.7 - 7.0)
Maldives	Enterococcus faecalis	5 (3 - 8)	1.7 (1.0 - 2.7)
Maldives	Enterococcus faecium	5 (3 - 8)	1.7 (1.0 - 2.6)
Maldives	Escherichia coli	16 (11 - 23)	5.9 (4.0 - 8.5)
Maldives	Group A Streptococcus	8 (5 - 12)	2.7 (1.7 - 4.5)
Maldives	Group B Streptococcus	6 (4 - 9)	1.9 (1.2 - 3.0)
Maldives	Haemophilus influenzae	2 (1 - 2)	0.5 (0.4 - 0.7)
Maldives	Klebsiella pneumoniae	13 (8 - 19)	4.7 (3.0 - 6.9)
Maldives	Legionella spp.	1 (1 - 2)	0.4 (0.3 - 0.6)
Maldives	Listeria monocytogenes	0 (0 - 0)	0.0 (0.0 - 0.1)
Maldives	Morganella spp.	0 (0 - 0)	0.0 (0.0 - 0.1)
Maldives	Mycoplasma spp.	3 (2 - 4)	0.9 (0.6 - 1.3)
Maldives	Neisseria gonorrhoeae	0 (0 - 0)	0.0 (0.0 - 0.0)

Maldives	Neisseria meningitidis	3 (1 - 4)	0.6 (0.4 - 1.1)
Maldives	Non-typhoidal Salmonella	3 (2 - 4)	0.8 (0.5 - 1.1)
Maldives	Other Klebsiella species	1 (1 - 2)	0.4 (0.2 - 0.6)
Maldives	Other enterococci	3 (2 - 5)	1.2 (0.8 - 1.7)
Maldives	Proteus spp.	2 (2 - 4)	0.9 (0.6 - 1.4)
Maldives	Providencia spp.	0 (0 - 0)	0.0 (0.0 - 0.0)
Maldives	Pseudomonas aeruginosa	13 (8 - 19)	4.7 (3.0 - 7.0)
Maldives	Salmonella Paratyphi	1 (0 - 2)	0.2 (0.1 - 0.3)
Maldives	Salmonella Typhi	6 (3 - 12)	1.3 (0.6 - 2.4)
Maldives	Serratia spp.	2 (1 - 4)	0.8 (0.5 - 1.3)
Maldives	Shigella spp.	0 (0 - 1)	0.1 (0.0 - 0.3)
Maldives	Staphylococcus aureus	21 (14 - 32)	7.5 (4.9 - 11.2)
Maldives	Streptococcus pneumoniae	15 (11 - 22)	5.0 (3.6 - 7.1)
Maldives	Vibrio cholerae	1 (0 - 2)	0.3 (0.1 - 0.8)
Mauritius	Acinetobacter baumannii	81 (43 - 133)	5.1 (2.8 - 8.4)
Mauritius	Aeromonas spp.	0 (0 - 0)	0.0 (0.0 - 0.0)
Mauritius	Campylobacter spp.	2 (1 - 5)	0.2 (0.0 - 0.4)
Mauritius	Chlamydia spp.	8 (6 - 11)	0.6 (0.4 - 0.8)
Mauritius	Citrobacter spp.	8 (4 - 12)	0.5 (0.3 - 0.8)
Mauritius	Clostridioides difficile	6 (4 - 9)	0.4 (0.3 - 0.6)
Mauritius	Enterobacter spp.	49 (29 - 76)	3.2 (1.9 - 4.9)
Mauritius	Enterococcus faecalis	40 (22 - 66)	2.5 (1.4 - 4.1)
Mauritius	Enterococcus faecium	49 (27 - 78)	3.0 (1.7 - 4.7)
Mauritius	Escherichia coli	160 (96 - 242)	10.2 (6.2 - 15.4)
Mauritius	Group A Streptococcus	50 (25 - 94)	3.3 (1.6 - 5.9)
Mauritius	Group B Streptococcus	45 (27 - 69)	3.0 (1.9 - 4.7)
Mauritius	Haemophilus influenzae	10 (7 - 15)	0.7 (0.5 - 1.0)
Mauritius	Klebsiella pneumoniae	136 (83 - 205)	8.9 (5.5 - 13.3)
Mauritius	Legionella spp.	8 (6 - 11)	0.6 (0.4 - 0.8)
Mauritius	Listeria monocytogenes	1 (0 - 2)	0.1 (0.0 - 0.1)
Mauritius	Morganella spp.	2 (1 - 3)	0.1 (0.1 - 0.2)
Mauritius	Mycoplasma spp.	9 (7 - 13)	0.7 (0.5 - 0.9)
Mauritius	Neisseria gonorrhoeae	0 (0 - 0)	0.0 (0.0 - 0.0)
Mauritius	Neisseria meningitidis	11 (6 - 19)	0.9 (0.5 - 1.4)
Mauritius	Non-typhoidal Salmonella	7 (4 - 12)	0.5 (0.3 - 0.8)
Mauritius	Other Klebsiella species	8 (4 - 14)	0.5 (0.2 - 0.9)
Mauritius	Other enterococci	22 (13 - 35)	1.4 (0.9 - 2.3)
Mauritius	Proteus spp.	23 (14 - 36)	1.4 (0.9 - 2.2)
Mauritius	Providencia spp.	1 (1 - 2)	0.1 (0.0 - 0.1)
Mauritius	Pseudomonas aeruginosa	117 (72 - 181)	7.6 (4.7 - 11.6)

Mauritius	Salmonella Paratyphi	1 (1 - 3)	0.1 (0.1 - 0.3)
Mauritius	Salmonella Typhi	3 (2 - 6)	0.3 (0.2 - 0.4)
Mauritius	Serratia spp.	18 (9 - 29)	1.1 (0.6 - 1.8)
Mauritius	Shigella spp.	1 (0 - 2)	0.1 (0.0 - 0.2)
Mauritius	Staphylococcus aureus	223 (145 - 328)	14.4 (9.5 - 21.1)
Mauritius	Streptococcus pneumoniae	99 (70 - 139)	6.8 (4.8 - 9.5)
Mauritius	Vibrio cholerae	1 (0 - 1)	0.1 (0.0 - 0.1)
Myanmar	Acinetobacter baumannii	3,530 (2,042 - 5,624)	9.1 (5.2 - 14.5)
Myanmar	Aeromonas spp.	15 (7 - 29)	0.0 (0.0 - 0.1)
Myanmar	Campylobacter spp.	485 (145 - 1,167)	1.2 (0.3 - 3.1)
Myanmar	Chlamydia spp.	729 (527 - 1,026)	1.7 (1.2 - 2.4)
Myanmar	Citrobacter spp.	376 (220 - 608)	0.8 (0.5 - 1.3)
Myanmar	Clostridioides difficile	26 (9 - 60)	0.0 (0.0 - 0.1)
Myanmar	Enterobacter spp.	2,232 (1,447 - 3,448)	5.1 (3.3 - 7.8)
Myanmar	Enterococcus faecalis	1,873 (1,095 - 3,016)	4.1 (2.4 - 6.6)
Myanmar	Enterococcus faecium	1,715 (976 - 2,870)	3.8 (2.2 - 6.3)
Myanmar	Escherichia coli	7,530 (5,068 - 10,960)	17.8 (12.1 - 25.9)
Myanmar	Group A Streptococcus	1,889 (955 - 3,453)	4.7 (2.3 - 8.6)
Myanmar	Group B Streptococcus	3,068 (2,104 - 4,344)	7.0 (4.8 - 10.0)
Myanmar	Haemophilus influenzae	1,002 (741 - 1,350)	2.3 (1.7 - 3.0)
Myanmar	Klebsiella pneumoniae	8,316 (5,704 - 11,935)	19.7 (13.5 - 28.4)
Myanmar	Legionella spp.	323 (201 - 506)	0.7 (0.5 - 1.1)
Myanmar	Listeria monocytogenes	87 (47 - 151)	0.2 (0.1 - 0.3)
Myanmar	Morganella spp.	52 (28 - 85)	0.1 (0.1 - 0.2)
Myanmar	Mycoplasma spp.	610 (440 - 815)	1.3 (1.0 - 1.7)
Myanmar	Neisseria gonorrhoeae	12 (8 - 17)	0.0 (0.0 - 0.0)
Myanmar	Neisseria meningitidis	1,251 (735 - 2,062)	2.5 (1.5 - 4.1)
Myanmar	Non-typhoidal Salmonella	1,057 (669 - 1,633)	2.3 (1.4 - 3.6)
Myanmar	Other Klebsiella species	508 (231 - 962)	1.0 (0.5 - 1.9)
Myanmar	Other enterococci	818 (511 - 1,258)	2.1 (1.3 - 3.2)
Myanmar	Proteus spp.	1,010 (613 - 1,588)	2.5 (1.5 - 3.9)
Myanmar	Providencia spp.	65 (35 - 109)	0.2 (0.1 - 0.3)
Myanmar	Pseudomonas aeruginosa	5,298 (3,526 - 7,765)	12.8 (8.5 - 18.6)
Myanmar	Salmonella Paratyphi	114 (43 - 257)	0.2 (0.1 - 0.5)
Myanmar	Salmonella Typhi	1,820 (1,080 - 2,970)	3.5 (2.1 - 5.6)
Myanmar	Serratia spp.	1,030 (605 - 1,720)	2.3 (1.4 - 3.9)
Myanmar	Shigella spp.	246 (90 - 545)	0.6 (0.2 - 1.3)
Myanmar	Staphylococcus aureus	9,373 (6,631 - 13,182)	22.6 (16.3 - 31.2)
Myanmar	Streptococcus pneumoniae	7,720 (5,806 - 10,347)	17.8 (13.5 - 23.6)
Myanmar	Vibrio cholerae	409 (139 - 998)	1.0 (0.3 - 2.3)

Philippines	Acinetobacter baumannii	6,159 (3,703 - 9,827)	9.0 (5.5 - 14.3)
Philippines	Aeromonas spp.	28 (13 - 51)	0.0 (0.0 - 0.1)
Philippines	Campylobacter spp.	626 (192 - 1,409)	0.8 (0.2 - 2.1)
Philippines	Chlamydia spp.	1,588 (1,231 - 1,994)	2.2 (1.7 - 2.8)
Philippines	Citrobacter spp.	580 (372 - 880)	0.7 (0.5 - 1.1)
Philippines	Clostridioides difficile	188 (87 - 333)	0.2 (0.1 - 0.3)
Philippines	Enterobacter spp.	3,556 (2,451 - 5,003)	4.7 (3.2 - 6.7)
Philippines	Enterococcus faecalis	2,414 (1,525 - 3,578)	3.1 (1.9 - 4.7)
Philippines	Enterococcus faecium	2,247 (1,338 - 3,577)	3.0 (1.8 - 4.8)
Philippines	Escherichia coli	10,720 (7,750 - 14,522)	15.3 (11.1 - 20.8)
Philippines	Group A Streptococcus	2,725 (1,685 - 4,391)	3.8 (2.3 - 6.4)
Philippines	Group B Streptococcus	4,479 (3,363 - 5,773)	5.8 (4.4 - 7.6)
Philippines	Haemophilus influenzae	1,837 (1,497 - 2,215)	2.4 (1.9 - 2.9)
Philippines	Klebsiella pneumoniae	12,326 (9,178 - 16,585)	17.0 (12.7 - 22.9)
Philippines	Legionella spp.	739 (530 - 1,020)	1.0 (0.7 - 1.3)
Philippines	Listeria monocytogenes	115 (77 - 165)	0.1 (0.1 - 0.2)
Philippines	Morganella spp.	99 (59 - 157)	0.2 (0.1 - 0.2)
Philippines	Mycoplasma spp.	1,470 (1,214 - 1,785)	1.7 (1.4 - 2.1)
Philippines	Neisseria gonorrhoeae	24 (10 - 32)	0.0 (0.0 - 0.0)
Philippines	Neisseria meningitidis	2,011 (1,328 - 2,905)	1.8 (1.2 - 2.7)
Philippines	Non-typhoidal Salmonella	1,909 (1,319 - 2,712)	2.0 (1.3 - 2.9)
Philippines	Other Klebsiella species	556 (270 - 1,026)	0.7 (0.4 - 1.4)
Philippines	Other enterococci	1,294 (876 - 1,889)	1.9 (1.2 - 2.8)
Philippines	Proteus spp.	1,378 (898 - 2,006)	2.1 (1.3 - 3.0)
Philippines	Providencia spp.	115 (68 - 185)	0.2 (0.1 - 0.3)
Philippines	Pseudomonas aeruginosa	8,460 (6,104 - 11,513)	12.0 (8.7 - 16.4)
Philippines	Salmonella Paratyphi	228 (86 - 481)	0.2 (0.1 - 0.4)
Philippines	Salmonella Typhi	2,726 (1,621 - 4,277)	2.4 (1.5 - 3.7)
Philippines	Serratia spp.	1,419 (897 - 2,201)	1.7 (1.1 - 2.8)
Philippines	Shigella spp.	392 (152 - 834)	0.5 (0.2 - 1.0)
Philippines	Staphylococcus aureus	16,325 (12,648 - 21,157)	24.3 (18.9 - 31.0)
Philippines	Streptococcus pneumoniae	15,436 (12,727 - 18,652)	20.3 (16.4 - 24.5)
Philippines	Vibrio cholerae	1,039 (497 - 1,827)	1.4 (0.6 - 2.6)
Seychelles	Acinetobacter baumannii	9 (6 - 14)	9.4 (5.7 - 14.1)
Seychelles	Aeromonas spp.	0 (0 - 0)	0.0 (0.0 - 0.0)
Seychelles	Campylobacter spp.	0 (0 - 1)	0.4 (0.1 - 1.1)
Seychelles	Chlamydia spp.	2 (2 - 3)	2.6 (2.2 - 3.2)
Seychelles	Citrobacter spp.	1 (1 - 1)	1.0 (0.6 - 1.4)
Seychelles	Clostridioides difficile	1 (0 - 1)	0.5 (0.3 - 0.9)
Seychelles	Enterobacter spp.	6 (4 - 9)	6.1 (4.0 - 8.7)

Seychelles	Enterococcus faecalis	4 (2 - 6)	3.7 (2.4 - 5.5)
Seychelles	Enterococcus faecium	4 (2 - 6)	3.5 (2.2 - 5.3)
Seychelles	Escherichia coli	15 (11 - 20)	15.7 (11.8 - 20.4)
Seychelles	Group A Streptococcus	2 (1 - 4)	2.5 (1.5 - 4.5)
Seychelles	Group B Streptococcus	4 (3 - 5)	4.1 (3.2 - 5.3)
Seychelles	Haemophilus influenzae	2 (1 - 2)	1.9 (1.6 - 2.2)
Seychelles	Klebsiella pneumoniae	12 (9 - 16)	12.8 (9.6 - 16.8)
Seychelles	Legionella spp.	1 (1 - 2)	1.4 (1.1 - 1.8)
Seychelles	Listeria monocytogenes	0 (0 - 0)	0.2 (0.1 - 0.2)
Seychelles	Morganella spp.	0 (0 - 0)	0.2 (0.1 - 0.3)
Seychelles	Mycoplasma spp.	3 (2 - 3)	2.6 (2.2 - 3.1)
Seychelles	Neisseria gonorrhoeae	0 (0 - 0)	0.0 (0.0 - 0.1)
Seychelles	Neisseria meningitidis	1 (1 - 2)	1.2 (0.8 - 1.8)
Seychelles	Non-typhoidal Salmonella	1 (1 - 1)	1.0 (0.6 - 1.5)
Seychelles	Other Klebsiella species	1 (1 - 2)	0.9 (0.5 - 1.6)
Seychelles	Other enterococci	2 (1 - 3)	2.1 (1.5 - 2.9)
Seychelles	Proteus spp.	2 (2 - 3)	2.2 (1.6 - 3.0)
Seychelles	Providencia spp.	0 (0 - 0)	0.2 (0.1 - 0.2)
Seychelles	Pseudomonas aeruginosa	10 (8 - 14)	10.7 (7.9 - 14.3)
Seychelles	Salmonella Paratyphi	0 (0 - 0)	0.1 (0.1 - 0.3)
Seychelles	Salmonella Typhi	1 (1 - 2)	1.4 (0.7 - 2.5)
Seychelles	Serratia spp.	2 (1 - 2)	1.5 (1.0 - 2.4)
Seychelles	Shigella spp.	0 (0 - 0)	0.2 (0.1 - 0.4)
Seychelles	Staphylococcus aureus	19 (15 - 24)	19.6 (15.5 - 25.0)
Seychelles	Streptococcus pneumoniae	17 (14 - 20)	17.8 (15.0 - 21.0)
Seychelles	Vibrio cholerae	0 (0 - 0)	0.1 (0.0 - 0.2)
Sri Lanka	Acinetobacter baumannii	1,664 (881 - 2,707)	7.6 (4.1 - 12.3)
Sri Lanka	Aeromonas spp.	2 (1 - 5)	0.0 (0.0 - 0.0)
Sri Lanka	Campylobacter spp.	71 (11 - 204)	0.4 (0.1 - 1.0)
Sri Lanka	Chlamydia spp.	232 (158 - 334)	1.1 (0.8 - 1.6)
Sri Lanka	Citrobacter spp.	144 (77 - 239)	0.6 (0.3 - 1.1)
Sri Lanka	Clostridioides difficile	44 (20 - 87)	0.2 (0.1 - 0.4)
Sri Lanka	Enterobacter spp.	1,020 (563 - 1,636)	4.6 (2.6 - 7.2)
Sri Lanka	Enterococcus faecalis	471 (264 - 768)	2.0 (1.1 - 3.3)
Sri Lanka	Enterococcus faecium	474 (264 - 751)	2.0 (1.1 - 3.2)
Sri Lanka	Escherichia coli	1,660 (1,057 - 2,452)	7.4 (4.8 - 10.9)
Sri Lanka	Group A Streptococcus	418 (170 - 845)	1.9 (0.8 - 3.8)
Sri Lanka	Group B Streptococcus	419 (256 - 656)	1.9 (1.2 - 3.0)
Sri Lanka	Haemophilus influenzae	137 (93 - 194)	0.7 (0.4 - 0.9)
Sri Lanka	Klebsiella pneumoniae	1,241 (772 - 1,888)	5.6 (3.5 - 8.6)
Sri Lanka	Legionella spp.	133 (92 - 185)	0.6 (0.4 - 0.9)

Sri Lanka	Listeria monocytogenes	25 (16 - 37)	0.1 (0.1 - 0.2)
Sri Lanka	Morganella spp.	18 (10 - 28)	0.1 (0.0 - 0.1)
Sri Lanka	Mycoplasma spp.	258 (184 - 353)	1.2 (0.9 - 1.7)
Sri Lanka	Neisseria gonorrhoeae	6 (4 - 9)	0.0 (0.0 - 0.0)
Sri Lanka	Neisseria meningitidis	143 (81 - 231)	0.7 (0.4 - 1.1)
Sri Lanka	Non-typhoidal Salmonella	140 (81 - 223)	0.6 (0.4 - 1.0)
Sri Lanka	Other Klebsiella species	110 (55 - 193)	0.5 (0.2 - 0.8)
Sri Lanka	Other enterococci	264 (157 - 410)	1.2 (0.7 - 1.9)
Sri Lanka	Proteus spp.	244 (151 - 367)	1.1 (0.7 - 1.6)
Sri Lanka	Providencia spp.	12 (7 - 19)	0.1 (0.0 - 0.1)
Sri Lanka	Pseudomonas aeruginosa	1,171 (706 - 1,804)	5.3 (3.2 - 8.2)
Sri Lanka	Salmonella Paratyphi	31 (12 - 68)	0.2 (0.1 - 0.3)
Sri Lanka	Salmonella Typhi	307 (142 - 552)	1.5 (0.7 - 2.7)
Sri Lanka	Serratia spp.	207 (113 - 340)	0.9 (0.5 - 1.5)
Sri Lanka	Shigella spp.	28 (9 - 69)	0.2 (0.0 - 0.4)
Sri Lanka	Staphylococcus aureus	1,941 (1,223 - 2,913)	8.8 (5.6 - 13.1)
Sri Lanka	Streptococcus pneumoniae	1,388 (964 - 1,957)	6.5 (4.6 - 9.1)
Sri Lanka	Vibrio cholerae	154 (51 - 371)	0.7 (0.3 - 1.7)
Thailand	Acinetobacter baumannii	6,480 (3,747 - 10,221)	6.8 (4.0 - 10.7)
Thailand	Aeromonas spp.	9 (3 - 19)	0.0 (0.0 - 0.0)
Thailand	Campylobacter spp.	893 (145 - 2,441)	0.9 (0.2 - 2.6)
Thailand	Chlamydia spp.	634 (395 - 930)	0.7 (0.4 - 1.0)
Thailand	Citrobacter spp.	784 (453 - 1,212)	0.8 (0.5 - 1.3)
Thailand	Clostridioides difficile	234 (101 - 467)	0.3 (0.2 - 0.6)
Thailand	Enterobacter spp.	6,034 (3,762 - 9,026)	6.4 (4.0 - 9.4)
Thailand	Enterococcus faecalis	2,647 (1,450 - 4,329)	2.7 (1.5 - 4.5)
Thailand	Enterococcus faecium	2,749 (1,636 - 4,231)	2.8 (1.7 - 4.3)
Thailand	Escherichia coli	8,943 (5,761 - 13,140)	9.3 (6.0 - 13.6)
Thailand	Group A Streptococcus	2,828 (1,660 - 4,728)	3.0 (1.8 - 5.0)
Thailand	Group B Streptococcus	3,852 (2,457 - 5,782)	4.2 (2.7 - 6.3)
Thailand	Haemophilus influenzae	747 (518 - 1,015)	0.8 (0.6 - 1.1)
Thailand	Klebsiella pneumoniae	6,557 (4,254 - 9,568)	6.9 (4.5 - 9.9)
Thailand	Legionella spp.	171 (84 - 298)	0.2 (0.1 - 0.3)
Thailand	Listeria monocytogenes	70 (44 - 110)	0.1 (0.1 - 0.1)
Thailand	Morganella spp.	68 (36 - 109)	0.1 (0.0 - 0.1)
Thailand	Mycoplasma spp.	826 (553 - 1,156)	1.0 (0.7 - 1.3)
Thailand	Neisseria gonorrhoeae	12 (8 - 16)	0.0 (0.0 - 0.0)
Thailand	Neisseria meningitidis	737 (443 - 1,139)	0.9 (0.6 - 1.4)
Thailand	Non-typhoidal Salmonella	770 (472 - 1,179)	0.9 (0.6 - 1.4)
Thailand	Other Klebsiella species	706 (371 - 1,242)	0.7 (0.4 - 1.3)

Thailand	Other enterococci	1,342 (834 - 1,990)	1.4 (0.9 - 2.1)
Thailand	Proteus spp.	1,306 (835 - 1,914)	1.3 (0.9 - 2.0)
Thailand	Providencia spp.	47 (25 - 76)	0.0 (0.0 - 0.1)
Thailand	Pseudomonas aeruginosa	5,855 (3,720 - 8,599)	6.1 (3.9 - 8.9)
Thailand	Salmonella Paratyphi	72 (27 - 151)	0.1 (0.1 - 0.3)
Thailand	Salmonella Typhi	740 (338 - 1,391)	1.4 (0.6 - 2.5)
Thailand	Serratia spp.	1,149 (683 - 1,768)	1.2 (0.7 - 1.8)
Thailand	Shigella spp.	251 (78 - 591)	0.3 (0.1 - 0.6)
Thailand	Staphylococcus aureus	9,290 (6,002 - 13,558)	9.6 (6.3 - 14.0)
Thailand	Streptococcus pneumoniae	7,882 (5,472 - 10,723)	8.8 (6.3 - 11.8)
Thailand	Vibrio cholerae	114 (36 - 283)	0.1 (0.0 - 0.3)
Timor-Leste	Acinetobacter baumannii	88 (54 - 134)	11.9 (7.1 - 18.3)
Timor-Leste	Aeromonas spp.	1 (0 - 1)	0.1 (0.0 - 0.1)
Timor-Leste	Campylobacter spp.	16 (4 - 40)	2.1 (0.4 - 5.6)
Timor-Leste	Chlamydia spp.	30 (22 - 41)	2.9 (2.1 - 3.8)
Timor-Leste	Citrobacter spp.	13 (8 - 20)	1.4 (0.8 - 2.2)
Timor-Leste	Clostridioides difficile	0 (0 - 1)	0.0 (0.0 - 0.1)
Timor-Leste	Enterobacter spp.	65 (44 - 94)	7.3 (4.7 - 10.7)
Timor-Leste	Enterococcus faecalis	29 (17 - 46)	3.6 (2.0 - 5.9)
Timor-Leste	Enterococcus faecium	27 (15 - 44)	3.5 (2.0 - 5.8)
Timor-Leste	Escherichia coli	120 (84 - 164)	14.6 (10.2 - 20.0)
Timor-Leste	Group A Streptococcus	28 (15 - 49)	3.7 (1.8 - 6.7)
Timor-Leste	Group B Streptococcus	71 (51 - 96)	6.4 (4.6 - 8.7)
Timor-Leste	Haemophilus influenzae	26 (19 - 33)	2.2 (1.7 - 2.7)
Timor-Leste	Klebsiella pneumoniae	136 (98 - 184)	15.5 (11.0 - 21.5)
Timor-Leste	Legionella spp.	6 (3 - 9)	0.5 (0.3 - 0.8)
Timor-Leste	Listeria monocytogenes	2 (1 - 2)	0.1 (0.1 - 0.2)
Timor-Leste	Morganella spp.	1 (1 - 2)	0.2 (0.1 - 0.3)
Timor-Leste	Mycoplasma spp.	25 (18 - 33)	1.9 (1.5 - 2.5)
Timor-Leste	Neisseria gonorrhoeae	0 (0 - 0)	0.0 (0.0 - 0.0)
Timor-Leste	Neisseria meningitidis	36 (24 - 53)	2.7 (1.7 - 4.1)
Timor-Leste	Non-typhoidal Salmonella	40 (26 - 59)	3.8 (2.4 - 5.9)
Timor-Leste	Other Klebsiella species	9 (4 - 17)	1.1 (0.5 - 2.2)
Timor-Leste	Other enterococci	16 (10 - 25)	2.1 (1.3 - 3.4)
Timor-Leste	Proteus spp.	17 (10 - 26)	2.4 (1.5 - 3.6)
Timor-Leste	Providencia spp.	1 (1 - 2)	0.2 (0.1 - 0.4)
Timor-Leste	Pseudomonas aeruginosa	88 (61 - 123)	10.4 (7.2 - 14.8)
Timor-Leste	Salmonella Paratyphi	3 (1 - 6)	0.2 (0.1 - 0.4)
Timor-Leste	Salmonella Typhi	35 (19 - 59)	2.4 (1.4 - 3.9)
Timor-Leste	Serratia spp.	23 (14 - 36)	2.6 (1.6 - 4.2)

Timor-Leste	Shigella spp.	9 (3 - 19)	0.9 (0.3 - 2.1)
Timor-Leste	Staphylococcus aureus	120 (87 - 164)	14.5 (10.6 - 19.7)
Timor-Leste	Streptococcus pneumoniae	215 (160 - 272)	19.9 (15.4 - 25.0)
Timor-Leste	Vibrio cholerae	16 (4 - 40)	1.7 (0.4 - 4.5)
Viet Nam	Acinetobacter baumannii	7,117 (4,070 - 11,578)	9.0 (5.2 - 14.5)
Viet Nam	Aeromonas spp.	4 (2 - 9)	0.0 (0.0 - 0.0)
Viet Nam	Campylobacter spp.	81 (16 - 222)	0.1 (0.0 - 0.3)
Viet Nam	Chlamydia spp.	1,017 (764 - 1,356)	1.4 (1.0 - 1.8)
Viet Nam	Citrobacter spp.	761 (429 - 1,260)	0.9 (0.5 - 1.5)
Viet Nam	Clostridioides difficile	143 (66 - 287)	0.2 (0.1 - 0.3)
Viet Nam	Enterobacter spp.	5,051 (3,096 - 7,956)	6.1 (3.8 - 9.4)
Viet Nam	Enterococcus faecalis	2,340 (1,330 - 3,828)	2.7 (1.6 - 4.6)
Viet Nam	Enterococcus faecium	2,407 (1,415 - 3,834)	2.8 (1.7 - 4.4)
Viet Nam	Escherichia coli	7,301 (4,696 - 10,672)	9.0 (5.9 - 13.0)
Viet Nam	Group A Streptococcus	2,502 (1,147 - 4,873)	3.1 (1.5 - 6.1)
Viet Nam	Group B Streptococcus	2,146 (1,397 - 3,282)	2.7 (1.8 - 4.1)
Viet Nam	Haemophilus influenzae	670 (507 - 890)	0.9 (0.7 - 1.2)
Viet Nam	Klebsiella pneumoniae	6,290 (4,157 - 9,346)	7.9 (5.3 - 11.5)
Viet Nam	Legionella spp.	441 (321 - 600)	0.6 (0.4 - 0.8)
Viet Nam	Listeria monocytogenes	71 (42 - 125)	0.1 (0.0 - 0.1)
Viet Nam	Morganella spp.	41 (21 - 69)	0.1 (0.0 - 0.1)
Viet Nam	Mycoplasma spp.	1,054 (813 - 1,350)	1.3 (1.0 - 1.7)
Viet Nam	Neisseria gonorrhoeae	3 (2 - 4)	0.0 (0.0 - 0.0)
Viet Nam	Neisseria meningitidis	873 (508 - 1,451)	1.0 (0.6 - 1.6)
Viet Nam	Non-typhoidal Salmonella	871 (540 - 1,367)	1.0 (0.7 - 1.6)
Viet Nam	Other Klebsiella species	667 (346 - 1,146)	0.8 (0.4 - 1.3)
Viet Nam	Other enterococci	1,126 (670 - 1,787)	1.4 (0.9 - 2.3)
Viet Nam	Proteus spp.	1,118 (685 - 1,687)	1.4 (0.9 - 2.1)
Viet Nam	Providencia spp.	34 (17 - 59)	0.0 (0.0 - 0.1)
Viet Nam	Pseudomonas aeruginosa	5,511 (3,528 - 8,310)	6.9 (4.5 - 10.3)
Viet Nam	Salmonella Paratyphi	142 (54 - 304)	0.2 (0.1 - 0.3)
Viet Nam	Salmonella Typhi	1,398 (708 - 2,475)	1.6 (0.8 - 2.8)
Viet Nam	Serratia spp.	1,185 (692 - 1,980)	1.4 (0.8 - 2.3)
Viet Nam	Shigella spp.	47 (16 - 108)	0.1 (0.0 - 0.2)
Viet Nam	Staphylococcus aureus	8,815 (5,878 - 12,972)	10.8 (7.4 - 15.7)
Viet Nam	Streptococcus pneumoniae	6,756 (5,162 - 8,908)	8.7 (6.7 - 11.3)
Viet Nam	Vibrio cholerae	61 (20 - 140)	0.1 (0.0 - 0.2)
Angola	Acinetobacter baumannii	1,137 (698 - 1,728)	10.0 (6.0 - 15.6)
Angola	Aeromonas spp.	251 (99 - 513)	1.1 (0.5 - 2.0)
Angola	Campylobacter spp.	682 (216 - 1,576)	4.9 (1.1 - 12.9)

Angola	Chlamydia spp.	546 (370 - 788)	2.4 (1.8 - 3.4)
Angola	Citrobacter spp.	213 (127 - 329)	1.2 (0.7 - 1.9)
Angola	Clostridioides difficile	19 (6 - 49)	0.1 (0.0 - 0.1)
Angola	Enterobacter spp.	1,152 (797 - 1,660)	7.1 (4.9 - 10.5)
Angola	Enterococcus faecalis	707 (437 - 1,080)	4.9 (2.7 - 7.7)
Angola	Enterococcus faecium	519 (298 - 857)	4.2 (2.4 - 7.0)
Angola	Escherichia coli	3,382 (2,490 - 4,537)	21.5 (15.8 - 28.8)
Angola	Group A Streptococcus	689 (383 - 1,174)	5.3 (2.4 - 10.3)
Angola	Group B Streptococcus	3,082 (2,178 - 4,178)	12.2 (8.8 - 16.5)
Angola	Haemophilus influenzae	829 (595 - 1,103)	3.2 (2.5 - 4.1)
Angola	Klebsiella pneumoniae	4,170 (3,030 - 5,554)	24.0 (17.6 - 32.2)
Angola	Legionella spp.	96 (51 - 181)	0.4 (0.3 - 0.6)
Angola	Listeria monocytogenes	121 (77 - 179)	0.5 (0.3 - 0.7)
Angola	Morganella spp.	7 (4 - 12)	0.1 (0.0 - 0.1)
Angola	Mycoplasma spp.	420 (293 - 589)	1.5 (1.1 - 1.9)
Angola	Neisseria gonorrhoeae	18 (8 - 35)	0.1 (0.1 - 0.3)
Angola	Neisseria meningitidis	1,549 (1,030 - 2,257)	4.7 (3.2 - 6.7)
Angola	Non-typhoidal Salmonella	2,094 (1,250 - 3,314)	7.7 (4.2 - 13.7)
Angola	Other Klebsiella species	207 (94 - 394)	1.8 (0.8 - 3.4)
Angola	Other enterococci	271 (171 - 425)	1.9 (1.1 - 3.1)
Angola	Proteus spp.	311 (187 - 486)	3.0 (1.8 - 4.8)
Angola	Providencia spp.	11 (6 - 19)	0.1 (0.1 - 0.2)
Angola	Pseudomonas aeruginosa	2,049 (1,435 - 2,813)	13.0 (9.0 - 18.4)
Angola	Salmonella Paratyphi	0 (0 - 1)	0.0 (0.0 - 0.0)
Angola	Salmonella Typhi	972 (621 - 1,410)	2.9 (1.8 - 4.2)
Angola	Serratia spp.	520 (325 - 797)	2.8 (1.7 - 4.4)
Angola	Shigella spp.	649 (227 - 1,371)	2.6 (1.0 - 5.8)
Angola	Staphylococcus aureus	3,245 (2,374 - 4,391)	21.7 (16.5 - 29.4)
Angola	Streptococcus pneumoniae	5,790 (4,253 - 7,862)	25.6 (20.1 - 32.6)
Angola	Vibrio cholerae	1,520 (680 - 2,877)	10.0 (4.2 - 21.8)
Central African Republic	Acinetobacter baumannii	403 (249 - 619)	19.4 (11.4 - 30.9)
Central African Republic	Aeromonas spp.	159 (59 - 354)	3.4 (1.4 - 6.6)
Central African Republic	Campylobacter spp.	106 (28 - 255)	3.5 (0.7 - 9.0)
Central African Republic	Chlamydia spp.	214 (140 - 313)	4.4 (3.0 - 6.1)
Central African Republic	Citrobacter spp.	45 (25 - 75)	1.4 (0.8 - 2.4)
Central African Republic	Clostridioides difficile	1 (0 - 3)	0.0 (0.0 - 0.1)
Central African Republic	Enterobacter spp.	277 (186 - 406)	8.7 (5.6 - 12.8)
Central African Republic	Enterococcus faecalis	163 (97 - 264)	5.8 (3.1 - 9.9)
Central African Republic	Enterococcus faecium	112 (61 - 203)	4.8 (2.5 - 8.5)
Central African Republic	Escherichia coli	1,265 (916 - 1,730)	33.7 (24.8 - 45.4)
Central African Republic	Group A Streptococcus	158 (88 - 279)	6.8 (3.0 - 13.6)

Central African Republic	Group B Streptococcus	1,306 (892 - 1,867)	23.9 (16.4 - 33.9)
Central African Republic	Haemophilus influenzae	327 (229 - 454)	6.0 (4.3 - 8.2)
Central African Republic	Klebsiella pneumoniae	1,566 (1,138 - 2,109)	41.0 (29.2 - 55.9)
Central African Republic	Legionella spp.	24 (12 - 45)	0.5 (0.3 - 0.8)
Central African Republic	Listeria monocytogenes	45 (27 - 71)	0.9 (0.6 - 1.5)
Central African Republic	Morganella spp.	2 (1 - 5)	0.2 (0.1 - 0.3)
Central African Republic	Mycoplasma spp.	116 (81 - 166)	2.0 (1.4 - 2.7)
Central African Republic	Neisseria gonorrhoeae	6 (3 - 12)	0.2 (0.1 - 0.5)
Central African Republic	Neisseria meningitidis	559 (384 - 804)	9.8 (6.7 - 13.9)
Central African Republic	Non-typhoidal Salmonella	769 (472 - 1,187)	15.3 (9.0 - 25.0)
Central African Republic	Other Klebsiella species	54 (21 - 116)	2.4 (0.9 - 5.1)
Central African Republic	Other enterococci	57 (34 - 95)	2.2 (1.2 - 4.1)
Central African Republic	Proteus spp.	83 (46 - 140)	4.2 (2.3 - 7.1)
Central African Republic	Providencia spp.	6 (3 - 11)	0.3 (0.2 - 0.6)
Central African Republic	Pseudomonas aeruginosa	603 (433 - 833)	17.5 (12.1 - 24.5)
Central African Republic	Salmonella Paratyphi	0 (0 - 0)	0.0 (0.0 - 0.0)
Central African Republic	Salmonella Typhi	631 (398 - 938)	10.7 (6.7 - 15.9)
Central African Republic	Serratia spp.	137 (82 - 215)	4.0 (2.3 - 6.5)
Central African Republic	Shigella spp.	1,919 (820 - 3,373)	39.8 (16.9 - 74.9)
Central African Republic	Staphylococcus aureus	1,056 (773 - 1,434)	32.2 (23.1 - 44.2)
Central African Republic	Streptococcus pneumoniae	2,196 (1,575 - 3,074)	45.1 (33.9 - 60.3)
Central African Republic	Vibrio cholerae	1,211 (528 - 2,415)	43.7 (16.5 - 97.8)
Congo	Acinetobacter baumannii	264 (152 - 420)	12.5 (7.2 - 19.9)
Congo	Aeromonas spp.	33 (10 - 71)	0.9 (0.4 - 1.7)
Congo	Campylobacter spp.	53 (13 - 129)	2.0 (0.4 - 5.4)
Congo	Chlamydia spp.	72 (52 - 102)	2.3 (1.7 - 3.2)
Congo	Citrobacter spp.	27 (16 - 43)	1.0 (0.6 - 1.5)
Congo	Clostridioides difficile	9 (3 - 21)	0.2 (0.1 - 0.4)
Congo	Enterobacter spp.	167 (112 - 247)	6.3 (4.3 - 9.3)
Congo	Enterococcus faecalis	128 (76 - 198)	4.7 (2.7 - 7.5)
Congo	Enterococcus faecium	104 (59 - 174)	4.2 (2.4 - 7.0)
Congo	Escherichia coli	570 (410 - 782)	21.9 (15.9 - 29.7)
Congo	Group A Streptococcus	130 (67 - 232)	5.5 (2.4 - 10.6)
Congo	Group B Streptococcus	323 (231 - 440)	9.5 (6.8 - 13.0)
Congo	Haemophilus influenzae	91 (69 - 123)	2.8 (2.2 - 3.6)
Congo	Klebsiella pneumoniae	677 (486 - 927)	25.0 (18.1 - 34.2)
Congo	Legionella spp.	22 (13 - 36)	0.7 (0.4 - 1.0)
Congo	Listeria monocytogenes	15 (10 - 22)	0.4 (0.3 - 0.6)
Congo	Morganella spp.	2 (1 - 3)	0.1 (0.0 - 0.2)
Congo	Mycoplasma spp.	50 (36 - 67)	1.3 (1.0 - 1.7)
Congo	Neisseria gonorrhoeae	4 (2 - 6)	0.1 (0.1 - 0.2)

Congo	Neisseria meningitidis	161 (109 - 229)	3.4 (2.4 - 4.9)
Congo	Non-typhoidal Salmonella	206 (124 - 334)	5.0 (2.9 - 8.6)
Congo	Other Klebsiella species	40 (18 - 76)	1.6 (0.7 - 3.0)
Congo	Other enterococci	46 (29 - 73)	1.9 (1.1 - 3.1)
Congo	Proteus spp.	65 (38 - 102)	3.0 (1.8 - 4.8)
Congo	Providencia spp.	3 (1 - 5)	0.1 (0.1 - 0.2)
Congo	Pseudomonas aeruginosa	368 (255 - 515)	14.3 (9.9 - 20.0)
Congo	Salmonella Paratyphi	0 (0 - 0)	0.0 (0.0 - 0.0)
Congo	Salmonella Typhi	136 (90 - 196)	2.8 (1.8 - 4.2)
Congo	Serratia spp.	77 (49 - 121)	2.6 (1.6 - 4.1)
Congo	Shigella spp.	192 (60 - 394)	4.8 (1.6 - 9.7)
Congo	Staphylococcus aureus	665 (489 - 906)	27.0 (20.3 - 36.0)
Congo	Streptococcus pneumoniae	689 (520 - 893)	22.6 (17.7 - 28.6)
Congo	Vibrio cholerae	235 (91 - 507)	8.2 (2.8 - 19.6)
Democratic Republic of the Congo	Acinetobacter baumannii	4,298 (2,631 - 6,523)	13.4 (7.9 - 21.0)
Democratic Republic of the Congo	Aeromonas spp.	601 (179 - 1,508)	0.8 (0.3 - 1.7)
Democratic Republic of the Congo	Campylobacter spp.	900 (204 - 2,277)	1.9 (0.4 - 5.0)
Democratic Republic of the Congo	Chlamydia spp.	1,534 (1,070 - 2,114)	2.7 (1.9 - 3.7)
Democratic Republic of the Congo	Citrobacter spp.	431 (251 - 680)	0.9 (0.5 - 1.4)
Democratic Republic of the Congo	Clostridioides difficile	30 (8 - 81)	0.0 (0.0 - 0.1)
Democratic Republic of the Congo	Enterobacter spp.	2,601 (1,815 - 3,740)	5.9 (4.0 - 8.6)
Democratic Republic of the Congo	Enterococcus faecalis	2,048 (1,263 - 3,125)	4.6 (2.6 - 7.2)
Democratic Republic of the Congo	Enterococcus faecium	1,510 (886 - 2,552)	3.8 (2.2 - 6.6)
Democratic Republic of the Congo	Escherichia coli	10,438 (7,741 - 14,009)	22.7 (16.6 - 30.5)
Democratic Republic of the Congo	Group A Streptococcus	2,184 (1,219 - 3,675)	5.7 (2.6 - 10.9)
Democratic Republic of the Congo	Group B Streptococcus	6,786 (4,857 - 9,123)	10.6 (7.6 - 14.4)
Democratic Republic of the Congo	Haemophilus influenzae	2,068 (1,538 - 2,754)	3.3 (2.5 - 4.3)
Democratic Republic of the Congo	Klebsiella pneumoniae	13,636 (10,211 - 18,036)	28.2 (20.6 - 38.1)
Democratic Republic of the Congo	Legionella spp.	437 (247 - 733)	0.7 (0.5 - 1.1)
Democratic Republic of the Congo	Listeria monocytogenes	337 (223 - 517)	0.5 (0.3 - 0.7)
Democratic Republic of the Congo	Morganella spp.	23 (12 - 38)	0.1 (0.0 - 0.1)
Democratic Republic of the Congo	Mycoplasma spp.	987 (719 - 1,326)	1.4 (1.0 - 1.9)
Democratic Republic of the Congo	Neisseria gonorrhoeae	55 (24 - 101)	0.1 (0.1 - 0.2)
Democratic Republic of the Congo	Neisseria meningitidis	3,830 (2,701 - 5,385)	4.4 (3.1 - 6.2)
Democratic Republic of the Congo	Non-typhoidal Salmonella	7,303 (4,531 - 10,807)	9.3 (5.8 - 13.9)
Democratic Republic of the Congo	Other Klebsiella species	601 (260 - 1,164)	1.6 (0.7 - 3.1)
Democratic Republic of the Congo	Other enterococci	714 (444 - 1,127)	1.7 (1.0 - 3.0)
Democratic Republic of the Congo	Proteus spp.	983 (590 - 1,548)	3.0 (1.8 - 4.8)
Democratic Republic of the Congo	Providencia spp.	40 (20 - 70)	0.1 (0.1 - 0.2)
Democratic Republic of the Congo	Pseudomonas aeruginosa	6,560 (4,803 - 8,773)	14.6 (10.4 - 20.4)

Democratic Republic of the Congo	Salmonella Paratyphi	1 (0 - 3)	0.0 (0.0 - 0.0)
Democratic Republic of the Congo	Salmonella Typhi	4,096 (2,670 - 5,878)	4.6 (2.9 - 6.6)
Democratic Republic of the Congo	Serratia spp.	1,352 (845 - 2,078)	2.6 (1.5 - 4.1)
Democratic Republic of the Congo	Shigella spp.	3,561 (997 - 8,680)	4.7 (1.6 - 10.6)
Democratic Republic of the Congo	Staphylococcus aureus	12,800 (9,625 - 16,729)	30.8 (22.9 - 40.7)
Democratic Republic of the Congo	Streptococcus pneumoniae	14,673 (11,136 - 19,136)	26.4 (20.2 - 34.2)
Democratic Republic of the Congo	Vibrio cholerae	4,149 (1,656 - 9,854)	8.6 (3.1 - 20.5)
Equatorial Guinea	Acinetobacter baumannii	64 (40 - 99)	11.4 (6.9 - 17.9)
Equatorial Guinea	Aeromonas spp.	2 (1 - 4)	0.3 (0.1 - 0.6)
Equatorial Guinea	Campylobacter spp.	3 (1 - 10)	0.7 (0.1 - 2.2)
Equatorial Guinea	Chlamydia spp.	19 (12 - 29)	2.5 (1.7 - 3.5)
Equatorial Guinea	Citrobacter spp.	12 (7 - 19)	1.5 (0.9 - 2.4)
Equatorial Guinea	Clostridioides difficile	7 (3 - 16)	0.5 (0.2 - 1.1)
Equatorial Guinea	Enterobacter spp.	63 (40 - 95)	8.4 (5.5 - 12.7)
Equatorial Guinea	Enterococcus faecalis	23 (14 - 37)	3.9 (2.2 - 6.3)
Equatorial Guinea	Enterococcus faecium	18 (10 - 29)	3.4 (2.0 - 5.6)
Equatorial Guinea	Escherichia coli	81 (53 - 120)	14.6 (9.9 - 20.7)
Equatorial Guinea	Group A Streptococcus	29 (15 - 52)	5.1 (2.4 - 9.9)
Equatorial Guinea	Group B Streptococcus	65 (42 - 98)	7.7 (5.3 - 11.4)
Equatorial Guinea	Haemophilus influenzae	18 (11 - 27)	2.1 (1.5 - 3.0)
Equatorial Guinea	Klebsiella pneumoniae	100 (67 - 144)	15.7 (10.8 - 22.4)
Equatorial Guinea	Legionella spp.	3 (2 - 7)	0.4 (0.3 - 0.7)
Equatorial Guinea	Listeria monocytogenes	3 (2 - 4)	0.3 (0.2 - 0.5)
Equatorial Guinea	Morganella spp.	0 (0 - 0)	0.1 (0.0 - 0.1)
Equatorial Guinea	Mycoplasma spp.	16 (10 - 24)	1.7 (1.1 - 2.4)
Equatorial Guinea	Neisseria gonorrhoeae	1 (0 - 1)	0.1 (0.0 - 0.2)
Equatorial Guinea	Neisseria meningitidis	49 (30 - 75)	4.0 (2.5 - 5.9)
Equatorial Guinea	Non-typhoidal Salmonella	56 (35 - 85)	5.4 (3.4 - 8.2)
Equatorial Guinea	Other Klebsiella species	6 (3 - 11)	1.2 (0.5 - 2.2)
Equatorial Guinea	Other enterococci	13 (8 - 21)	2.1 (1.3 - 3.4)
Equatorial Guinea	Proteus spp.	11 (6 - 16)	2.3 (1.4 - 3.6)
Equatorial Guinea	Providencia spp.	0 (0 - 1)	0.1 (0.0 - 0.1)
Equatorial Guinea	Pseudomonas aeruginosa	63 (42 - 93)	10.6 (7.1 - 15.6)
Equatorial Guinea	Salmonella Paratyphi	0 (0 - 0)	0.0 (0.0 - 0.0)
Equatorial Guinea	Salmonella Typhi	15 (9 - 23)	1.2 (0.7 - 1.8)
Equatorial Guinea	Serratia spp.	21 (12 - 32)	2.7 (1.7 - 4.3)
Equatorial Guinea	Shigella spp.	10 (3 - 23)	1.5 (0.5 - 3.5)
Equatorial Guinea	Staphylococcus aureus	85 (57 - 124)	15.0 (10.4 - 21.5)
Equatorial Guinea	Streptococcus pneumoniae	142 (95 - 210)	19.3 (14.1 - 26.7)
Equatorial Guinea	Vibrio cholerae	9 (3 - 21)	1.6 (0.5 - 4.3)

Gabon	Acinetobacter baumannii	140 (85 - 216)	15.2 (9.1 - 23.7)
Gabon	Aeromonas spp.	4 (1 - 8)	0.3 (0.1 - 0.7)
Gabon	Campylobacter spp.	8 (2 - 22)	0.8 (0.2 - 2.4)
Gabon	Chlamydia spp.	37 (26 - 51)	3.1 (2.3 - 4.2)
Gabon	Citrobacter spp.	15 (9 - 24)	1.4 (0.8 - 2.2)
Gabon	Clostridioides difficile	11 (4 - 25)	0.6 (0.2 - 1.4)
Gabon	Enterobacter spp.	85 (57 - 122)	7.7 (5.1 - 11.2)
Gabon	Enterococcus faecalis	43 (25 - 67)	4.2 (2.4 - 6.7)
Gabon	Enterococcus faecium	37 (21 - 61)	3.7 (2.2 - 6.0)
Gabon	Escherichia coli	156 (110 - 217)	15.7 (11.3 - 21.4)
Gabon	Group A Streptococcus	46 (23 - 85)	5.0 (2.3 - 9.5)
Gabon	Group B Streptococcus	71 (52 - 99)	5.9 (4.2 - 8.5)
Gabon	Haemophilus influenzae	25 (19 - 34)	2.1 (1.6 - 2.7)
Gabon	Klebsiella pneumoniae	170 (122 - 237)	16.4 (11.9 - 22.6)
Gabon	Legionella spp.	8 (5 - 13)	0.7 (0.5 - 1.0)
Gabon	Listeria monocytogenes	5 (3 - 7)	0.4 (0.3 - 0.5)
Gabon	Morganella spp.	1 (0 - 1)	0.1 (0.1 - 0.2)
Gabon	Mycoplasma spp.	26 (19 - 34)	1.9 (1.5 - 2.5)
Gabon	Neisseria gonorrhoeae	1 (0 - 2)	0.1 (0.0 - 0.1)
Gabon	Neisseria meningitidis	49 (33 - 71)	3.2 (2.2 - 4.6)
Gabon	Non-typhoidal Salmonella	50 (32 - 75)	3.9 (2.4 - 6.0)
Gabon	Other Klebsiella species	14 (7 - 25)	1.4 (0.6 - 2.5)
Gabon	Other enterococci	20 (12 - 31)	2.1 (1.3 - 3.3)
Gabon	Proteus spp.	23 (14 - 35)	2.6 (1.6 - 4.0)
Gabon	Providencia spp.	1 (1 - 2)	0.1 (0.1 - 0.2)
Gabon	Pseudomonas aeruginosa	111 (76 - 158)	11.1 (7.6 - 15.8)
Gabon	Salmonella Paratyphi	0 (0 - 0)	0.0 (0.0 - 0.0)
Gabon	Salmonella Typhi	16 (10 - 24)	1.0 (0.6 - 1.5)
Gabon	Serratia spp.	29 (18 - 45)	2.6 (1.6 - 4.2)
Gabon	Shigella spp.	23 (7 - 49)	1.8 (0.6 - 4.1)
Gabon	Staphylococcus aureus	167 (121 - 229)	16.7 (12.3 - 22.7)
Gabon	Streptococcus pneumoniae	213 (163 - 279)	18.7 (14.6 - 23.5)
Gabon	Vibrio cholerae	7 (2 - 18)	0.7 (0.2 - 1.9)
Burundi	Acinetobacter baumannii	650 (394 - 1,004)	15.1 (9.0 - 23.7)
Burundi	Aeromonas spp.	198 (62 - 487)	1.6 (0.7 - 3.2)
Burundi	Campylobacter spp.	938 (251 - 2,369)	11.5 (2.8 - 27.6)
Burundi	Chlamydia spp.	195 (132 - 279)	2.4 (1.7 - 3.3)
Burundi	Citrobacter spp.	58 (34 - 94)	0.9 (0.5 - 1.5)
Burundi	Clostridioides difficile	4 (1 - 10)	0.0 (0.0 - 0.1)
Burundi	Enterobacter spp.	330 (220 - 483)	5.6 (3.7 - 8.2)

Burundi	Enterococcus faecalis	319 (194 - 488)	5.1 (2.8 - 8.4)
Burundi	Enterococcus faecium	233 (133 - 385)	4.4 (2.5 - 7.4)
Burundi	Escherichia coli	1,866 (1,323 - 2,636)	27.1 (19.7 - 36.6)
Burundi	Group A Streptococcus	327 (186 - 556)	5.9 (2.7 - 11.4)
Burundi	Group B Streptococcus	781 (559 - 1,082)	8.6 (6.1 - 12.0)
Burundi	Haemophilus influenzae	291 (204 - 418)	3.2 (2.4 - 4.2)
Burundi	Klebsiella pneumoniae	2,158 (1,543 - 2,886)	30.9 (22.5 - 41.0)
Burundi	Legionella spp.	79 (46 - 135)	1.0 (0.6 - 1.4)
Burundi	Listeria monocytogenes	70 (45 - 109)	0.8 (0.5 - 1.2)
Burundi	Morganella spp.	3 (2 - 6)	0.1 (0.0 - 0.2)
Burundi	Mycoplasma spp.	130 (92 - 181)	1.3 (0.9 - 1.6)
Burundi	Neisseria gonorrhoeae	9 (4 - 13)	0.1 (0.1 - 0.2)
Burundi	Neisseria meningitidis	625 (406 - 925)	5.0 (3.4 - 7.1)
Burundi	Non-typhoidal Salmonella	608 (294 - 1,332)	5.2 (2.3 - 11.6)
Burundi	Other Klebsiella species	87 (37 - 168)	1.8 (0.8 - 3.6)
Burundi	Other enterococci	103 (63 - 168)	1.7 (1.0 - 3.0)
Burundi	Proteus spp.	145 (87 - 224)	3.3 (1.9 - 5.1)
Burundi	Providencia spp.	6 (3 - 10)	0.2 (0.1 - 0.3)
Burundi	Pseudomonas aeruginosa	983 (705 - 1,333)	15.3 (11.0 - 20.9)
Burundi	Salmonella Paratyphi	0 (0 - 1)	0.0 (0.0 - 0.0)
Burundi	Salmonella Typhi	966 (626 - 1,396)	6.9 (4.4 - 9.9)
Burundi	Serratia spp.	200 (121 - 309)	2.6 (1.5 - 4.2)
Burundi	Shigella spp.	767 (236 - 1,891)	7.1 (2.6 - 16.0)
Burundi	Staphylococcus aureus	2,159 (1,599 - 2,851)	37.3 (28.7 - 48.1)
Burundi	Streptococcus pneumoniae	2,047 (1,479 - 2,815)	25.0 (19.5 - 31.8)
Burundi	Vibrio cholerae	416 (166 - 987)	5.3 (2.1 - 11.8)
Comoros	Acinetobacter baumannii	74 (45 - 113)	16.3 (9.8 - 25.0)
Comoros	Aeromonas spp.	4 (2 - 8)	0.8 (0.3 - 1.5)
Comoros	Campylobacter spp.	29 (8 - 70)	5.9 (1.4 - 15.3)
Comoros	Chlamydia spp.	24 (17 - 33)	4.1 (3.0 - 5.5)
Comoros	Citrobacter spp.	8 (4 - 12)	1.4 (0.8 - 2.2)
Comoros	Clostridioides difficile	1 (0 - 2)	0.1 (0.0 - 0.2)
Comoros	Enterobacter spp.	37 (25 - 54)	7.1 (4.7 - 10.4)
Comoros	Enterococcus faecalis	21 (12 - 33)	4.2 (2.4 - 6.9)
Comoros	Enterococcus faecium	17 (10 - 29)	3.6 (2.1 - 6.1)
Comoros	Escherichia coli	91 (68 - 118)	18.1 (13.6 - 23.7)
Comoros	Group A Streptococcus	22 (11 - 40)	4.7 (2.1 - 9.0)
Comoros	Group B Streptococcus	43 (31 - 59)	7.2 (5.3 - 9.9)
Comoros	Haemophilus influenzae	17 (13 - 22)	2.9 (2.3 - 3.6)
Comoros	Klebsiella pneumoniae	100 (74 - 132)	19.3 (14.4 - 25.9)
Comoros	Legionella spp.	4 (2 - 7)	0.7 (0.4 - 1.1)

Comoros	Listeria monocytogenes	4 (2 - 5)	0.6 (0.4 - 0.9)
Comoros	Morganella spp.	0 (0 - 1)	0.1 (0.0 - 0.1)
Comoros	Mycoplasma spp.	15 (11 - 19)	2.3 (1.7 - 2.9)
Comoros	Neisseria gonorrhoeae	1 (0 - 1)	0.1 (0.1 - 0.1)
Comoros	Neisseria meningitidis	31 (22 - 44)	4.8 (3.3 - 6.7)
Comoros	Non-typhoidal Salmonella	32 (18 - 54)	5.4 (3.0 - 9.4)
Comoros	Other Klebsiella species	7 (3 - 13)	1.6 (0.7 - 2.9)
Comoros	Other enterococci	9 (5 - 14)	1.8 (1.1 - 3.0)
Comoros	Proteus spp.	12 (7 - 18)	2.6 (1.6 - 4.0)
Comoros	Providencia spp.	1 (0 - 1)	0.1 (0.1 - 0.2)
Comoros	Pseudomonas aeruginosa	56 (40 - 76)	11.0 (7.8 - 15.3)
Comoros	Salmonella Paratyphi	0 (0 - 0)	0.0 (0.0 - 0.0)
Comoros	Salmonella Typhi	20 (12 - 31)	2.8 (1.7 - 4.3)
Comoros	Serratia spp.	14 (9 - 23)	2.7 (1.7 - 4.4)
Comoros	Shigella spp.	19 (7 - 42)	3.5 (1.3 - 7.8)
Comoros	Staphylococcus aureus	86 (65 - 115)	17.4 (13.1 - 23.1)
Comoros	Streptococcus pneumoniae	137 (106 - 171)	23.7 (19.0 - 28.8)
Comoros	Vibrio cholerae	12 (5 - 25)	2.3 (1.0 - 4.8)
Djibouti	Acinetobacter baumannii	54 (33 - 84)	10.1 (6.1 - 15.9)
Djibouti	Aeromonas spp.	5 (2 - 12)	0.7 (0.3 - 1.3)
Djibouti	Campylobacter spp.	32 (8 - 76)	5.1 (1.0 - 13.4)
Djibouti	Chlamydia spp.	19 (13 - 26)	2.3 (1.7 - 3.2)
Djibouti	Citrobacter spp.	8 (5 - 12)	1.0 (0.6 - 1.6)
Djibouti	Clostridioides difficile	2 (1 - 4)	0.1 (0.0 - 0.3)
Djibouti	Enterobacter spp.	45 (30 - 66)	6.5 (4.4 - 9.5)
Djibouti	Enterococcus faecalis	31 (18 - 49)	4.6 (2.6 - 7.4)
Djibouti	Enterococcus faecium	25 (14 - 42)	4.1 (2.3 - 6.9)
Djibouti	Escherichia coli	140 (100 - 193)	21.5 (15.5 - 29.4)
Djibouti	Group A Streptococcus	33 (19 - 57)	5.5 (2.6 - 10.3)
Djibouti	Group B Streptococcus	88 (62 - 124)	10.2 (7.5 - 14.2)
Djibouti	Haemophilus influenzae	32 (23 - 43)	3.4 (2.6 - 4.4)
Djibouti	Klebsiella pneumoniae	173 (124 - 238)	24.2 (17.5 - 33.2)
Djibouti	Legionella spp.	5 (3 - 8)	0.6 (0.4 - 0.9)
Djibouti	Listeria monocytogenes	6 (4 - 9)	0.7 (0.5 - 1.0)
Djibouti	Morganella spp.	0 (0 - 1)	0.1 (0.0 - 0.1)
Djibouti	Mycoplasma spp.	18 (13 - 24)	1.7 (1.3 - 2.2)
Djibouti	Neisseria gonorrhoeae	1 (0 - 2)	0.1 (0.1 - 0.2)
Djibouti	Neisseria meningitidis	57 (38 - 86)	4.8 (3.3 - 6.9)
Djibouti	Non-typhoidal Salmonella	35 (21 - 61)	3.6 (2.0 - 6.6)
Djibouti	Other Klebsiella species	9 (4 - 17)	1.5 (0.7 - 2.8)

Djibouti	Other enterococci	12 (7 - 19)	1.9 (1.2 - 3.1)
Djibouti	Proteus spp.	14 (8 - 22)	2.8 (1.7 - 4.2)
Djibouti	Providencia spp.	1 (0 - 1)	0.1 (0.1 - 0.2)
Djibouti	Pseudomonas aeruginosa	92 (64 - 130)	13.9 (9.8 - 19.2)
Djibouti	Salmonella Paratyphi	0 (0 - 0)	0.0 (0.0 - 0.0)
Djibouti	Salmonella Typhi	47 (29 - 71)	3.7 (2.3 - 5.6)
Djibouti	Serratia spp.	20 (12 - 31)	2.6 (1.5 - 4.1)
Djibouti	Shigella spp.	23 (8 - 49)	2.9 (1.0 - 6.4)
Djibouti	Staphylococcus aureus	162 (118 - 224)	25.5 (19.2 - 33.9)
Djibouti	Streptococcus pneumoniae	237 (175 - 316)	27.0 (21.2 - 34.9)
Djibouti	Vibrio cholerae	20 (8 - 43)	2.7 (1.1 - 6.3)
Eritrea	Acinetobacter baumannii	423 (251 - 680)	18.8 (11.0 - 30.0)
Eritrea	Aeromonas spp.	76 (30 - 161)	1.6 (0.6 - 3.2)
Eritrea	Campylobacter spp.	388 (110 - 901)	11.6 (2.4 - 31.6)
Eritrea	Chlamydia spp.	111 (69 - 173)	3.0 (1.9 - 4.9)
Eritrea	Citrobacter spp.	31 (17 - 51)	1.0 (0.5 - 1.7)
Eritrea	Clostridioides difficile	3 (1 - 8)	0.0 (0.0 - 0.1)
Eritrea	Enterobacter spp.	191 (121 - 284)	6.6 (4.2 - 9.8)
Eritrea	Enterococcus faecalis	171 (98 - 277)	5.6 (3.0 - 9.4)
Eritrea	Enterococcus faecium	135 (75 - 231)	5.0 (2.7 - 8.3)
Eritrea	Escherichia coli	958 (682 - 1,360)	30.4 (22.2 - 41.8)
Eritrea	Group A Streptococcus	164 (91 - 298)	6.2 (2.8 - 12.5)
Eritrea	Group B Streptococcus	395 (261 - 579)	9.9 (6.6 - 14.5)
Eritrea	Haemophilus influenzae	167 (114 - 240)	4.0 (2.7 - 6.0)
Eritrea	Klebsiella pneumoniae	1,170 (811 - 1,658)	35.9 (25.3 - 51.0)
Eritrea	Legionella spp.	43 (23 - 76)	1.2 (0.7 - 2.0)
Eritrea	Listeria monocytogenes	38 (24 - 60)	0.9 (0.6 - 1.3)
Eritrea	Morganella spp.	2 (1 - 3)	0.1 (0.0 - 0.2)
Eritrea	Mycoplasma spp.	79 (50 - 122)	1.6 (1.0 - 2.6)
Eritrea	Neisseria gonorrhoeae	6 (3 - 9)	0.1 (0.1 - 0.2)
Eritrea	Neisseria meningitidis	296 (194 - 445)	4.9 (3.3 - 7.2)
Eritrea	Non-typhoidal Salmonella	239 (106 - 512)	4.7 (1.9 - 11.3)
Eritrea	Other Klebsiella species	58 (25 - 112)	2.2 (1.0 - 4.3)
Eritrea	Other enterococci	48 (28 - 81)	1.7 (1.0 - 3.2)
Eritrea	Proteus spp.	83 (47 - 134)	3.6 (2.1 - 5.9)
Eritrea	Providencia spp.	3 (2 - 6)	0.2 (0.1 - 0.3)
Eritrea	Pseudomonas aeruginosa	548 (372 - 786)	18.0 (12.4 - 25.5)
Eritrea	Salmonella Paratyphi	0 (0 - 0)	0.0 (0.0 - 0.0)
Eritrea	Salmonella Typhi	432 (265 - 668)	6.5 (3.9 - 10.1)
Eritrea	Serratia spp.	96 (56 - 152)	2.7 (1.5 - 4.4)

Eritrea	Shigella spp.	312 (107 - 712)	7.0 (2.3 - 17.4)
Eritrea	Staphylococcus aureus	1,320 (903 - 1,899)	46.2 (32.1 - 67.5)
Eritrea	Streptococcus pneumoniae	1,249 (859 - 1,815)	31.4 (21.9 - 46.4)
Eritrea	Vibrio cholerae	790 (364 - 1,700)	21.6 (8.8 - 52.2)
Ethiopia	Acinetobacter baumannii	4,168 (2,678 - 6,164)	9.1 (5.5 - 14.0)
Ethiopia	Aeromonas spp.	1,177 (469 - 2,322)	1.3 (0.5 - 2.4)
Ethiopia	Campylobacter spp.	7,962 (2,717 - 16,523)	13.0 (3.3 - 29.8)
Ethiopia	Chlamydia spp.	1,584 (1,134 - 2,176)	2.1 (1.6 - 2.7)
Ethiopia	Citrobacter spp.	745 (469 - 1,120)	1.2 (0.7 - 1.8)
Ethiopia	Clostridioides difficile	54 (19 - 126)	0.0 (0.0 - 0.1)
Ethiopia	Enterobacter spp.	3,942 (2,777 - 5,487)	6.8 (4.7 - 9.5)
Ethiopia	Enterococcus faecalis	3,019 (1,913 - 4,468)	5.4 (3.1 - 8.5)
Ethiopia	Enterococcus faecium	2,224 (1,355 - 3,491)	4.8 (2.8 - 7.6)
Ethiopia	Escherichia coli	13,937 (10,601 - 18,062)	24.4 (18.5 - 30.9)
Ethiopia	Group A Streptococcus	2,820 (1,692 - 4,633)	5.2 (2.6 - 9.7)
Ethiopia	Group B Streptococcus	8,366 (6,150 - 11,063)	10.0 (7.7 - 13.3)
Ethiopia	Haemophilus influenzae	2,370 (1,838 - 3,090)	2.8 (2.3 - 3.5)
Ethiopia	Klebsiella pneumoniae	16,224 (12,404 - 21,044)	25.6 (19.6 - 33.0)
Ethiopia	Legionella spp.	380 (219 - 658)	0.5 (0.3 - 0.7)
Ethiopia	Listeria monocytogenes	659 (357 - 1,096)	0.9 (0.5 - 1.5)
Ethiopia	Morganella spp.	29 (14 - 47)	0.1 (0.0 - 0.1)
Ethiopia	Mycoplasma spp.	1,212 (914 - 1,588)	1.3 (1.0 - 1.6)
Ethiopia	Neisseria gonorrhoeae	69 (45 - 111)	0.1 (0.1 - 0.2)
Ethiopia	Neisseria meningitidis	4,444 (3,127 - 6,333)	3.9 (2.8 - 5.4)
Ethiopia	Non-typhoidal Salmonella	4,079 (2,235 - 7,339)	4.4 (2.1 - 8.8)
Ethiopia	Other Klebsiella species	862 (408 - 1,593)	2.1 (0.9 - 3.8)
Ethiopia	Other enterococci	1,102 (694 - 1,674)	1.8 (1.1 - 2.9)
Ethiopia	Proteus spp.	1,341 (843 - 1,978)	3.2 (2.0 - 4.8)
Ethiopia	Providencia spp.	43 (21 - 69)	0.1 (0.1 - 0.2)
Ethiopia	Pseudomonas aeruginosa	7,715 (5,640 - 10,191)	13.1 (9.6 - 17.7)
Ethiopia	Salmonella Paratyphi	6 (2 - 15)	0.0 (0.0 - 0.0)
Ethiopia	Salmonella Typhi	5,484 (3,633 - 7,951)	4.3 (2.9 - 6.1)
Ethiopia	Serratia spp.	1,990 (1,277 - 2,972)	2.7 (1.7 - 4.2)
Ethiopia	Shigella spp.	4,337 (1,746 - 8,648)	5.5 (2.0 - 11.3)
Ethiopia	Staphylococcus aureus	13,416 (10,372 - 16,852)	24.9 (19.5 - 31.4)
Ethiopia	Streptococcus pneumoniae	18,120 (14,589 - 22,577)	23.9 (20.1 - 28.0)
Ethiopia	Vibrio cholerae	4,950 (2,300 - 9,917)	9.2 (3.8 - 20.3)
Kenya	Acinetobacter baumannii	2,105 (1,302 - 3,191)	10.9 (6.6 - 16.9)
Kenya	Aeromonas spp.	750 (334 - 1,370)	2.1 (1.0 - 3.7)
Kenya	Campylobacter spp.	2,145 (674 - 4,415)	8.5 (2.1 - 20.4)

Kenya	Chlamydia spp.	581 (438 - 761)	2.1 (1.6 - 2.7)
Kenya	Citrobacter spp.	264 (160 - 420)	1.1 (0.6 - 1.8)
Kenya	Clostridioides difficile	123 (59 - 240)	0.2 (0.1 - 0.5)
Kenya	Enterobacter spp.	1,497 (1,029 - 2,162)	6.5 (4.4 - 9.5)
Kenya	Enterococcus faecalis	1,409 (852 - 2,273)	5.9 (3.3 - 10.0)
Kenya	Enterococcus faecium	1,209 (705 - 2,040)	5.6 (3.2 - 9.6)
Kenya	Escherichia coli	6,211 (4,734 - 8,115)	26.5 (19.9 - 34.6)
Kenya	Group A Streptococcus	1,212 (699 - 2,056)	5.3 (2.6 - 10.0)
Kenya	Group B Streptococcus	2,430 (1,874 - 3,099)	8.0 (6.1 - 10.5)
Kenya	Haemophilus influenzae	856 (683 - 1,052)	2.8 (2.3 - 3.5)
Kenya	Klebsiella pneumoniae	6,925 (5,352 - 8,926)	28.0 (21.6 - 36.7)
Kenya	Legionella spp.	248 (162 - 384)	0.9 (0.6 - 1.2)
Kenya	Listeria monocytogenes	254 (186 - 346)	0.8 (0.6 - 1.1)
Kenya	Morganella spp.	14 (7 - 22)	0.1 (0.0 - 0.1)
Kenya	Mycoplasma spp.	446 (346 - 568)	1.3 (1.0 - 1.6)
Kenya	Neisseria gonorrhoeae	26 (15 - 35)	0.1 (0.0 - 0.1)
Kenya	Neisseria meningitidis	1,792 (1,328 - 2,373)	4.2 (3.2 - 5.5)
Kenya	Non-typhoidal Salmonella	3,564 (2,153 - 5,287)	8.2 (4.7 - 13.7)
Kenya	Other Klebsiella species	474 (214 - 906)	2.3 (1.0 - 4.4)
Kenya	Other enterococci	405 (262 - 599)	1.7 (1.1 - 2.8)
Kenya	Proteus spp.	673 (419 - 1,019)	3.5 (2.2 - 5.4)
Kenya	Providencia spp.	19 (10 - 32)	0.1 (0.1 - 0.2)
Kenya	Pseudomonas aeruginosa	3,553 (2,678 - 4,696)	15.1 (11.3 - 20.0)
Kenya	Salmonella Paratyphi	73 (30 - 150)	0.1 (0.1 - 0.3)
Kenya	Salmonella Typhi	2,835 (1,818 - 4,199)	5.5 (3.7 - 7.9)
Kenya	Serratia spp.	735 (470 - 1,097)	2.7 (1.6 - 4.0)
Kenya	Shigella spp.	2,332 (988 - 4,491)	7.4 (2.8 - 15.0)
Kenya	Staphylococcus aureus	7,359 (5,861 - 9,237)	33.1 (26.4 - 41.6)
Kenya	Streptococcus pneumoniae	6,532 (5,362 - 7,948)	22.8 (19.0 - 27.5)
Kenya	Vibrio cholerae	1,644 (791 - 3,301)	7.3 (3.0 - 15.9)
Madagascar	Acinetobacter baumannii	1,427 (835 - 2,254)	14.9 (8.3 - 23.9)
Madagascar	Aeromonas spp.	356 (141 - 695)	1.4 (0.6 - 2.6)
Madagascar	Campylobacter spp.	1,016 (318 - 2,351)	5.5 (1.4 - 14.3)
Madagascar	Chlamydia spp.	451 (316 - 613)	2.4 (1.8 - 3.3)
Madagascar	Citrobacter spp.	123 (73 - 196)	0.8 (0.5 - 1.4)
Madagascar	Clostridioides difficile	8 (3 - 21)	0.0 (0.0 - 0.1)
Madagascar	Enterobacter spp.	734 (489 - 1,053)	5.5 (3.6 - 8.1)
Madagascar	Enterococcus faecalis	628 (390 - 946)	4.6 (2.6 - 7.6)
Madagascar	Enterococcus faecium	464 (268 - 774)	4.0 (2.2 - 6.8)
Madagascar	Escherichia coli	3,740 (2,763 - 4,946)	24.8 (18.1 - 33.1)
Madagascar	Group A Streptococcus	676 (379 - 1,151)	5.7 (2.5 - 11.2)

Madagascar	Group B Streptococcus	1,832 (1,334 - 2,502)	9.1 (6.5 - 12.1)
Madagascar	Haemophilus influenzae	637 (479 - 846)	3.2 (2.4 - 4.2)
Madagascar	Klebsiella pneumoniae	4,353 (3,224 - 5,658)	28.6 (20.5 - 38.4)
Madagascar	Legionella spp.	162 (93 - 283)	0.9 (0.5 - 1.2)
Madagascar	Listeria monocytogenes	118 (78 - 170)	0.6 (0.4 - 0.9)
Madagascar	Morganella spp.	6 (3 - 10)	0.1 (0.0 - 0.1)
Madagascar	Mycoplasma spp.	307 (227 - 403)	1.3 (1.0 - 1.7)
Madagascar	Neisseria gonorrhoeae	21 (13 - 32)	0.1 (0.1 - 0.2)
Madagascar	Neisseria meningitidis	1,205 (826 - 1,706)	4.5 (3.1 - 6.3)
Madagascar	Non-typhoidal Salmonella	1,073 (478 - 2,295)	4.6 (2.0 - 10.3)
Madagascar	Other Klebsiella species	175 (76 - 344)	1.6 (0.7 - 3.2)
Madagascar	Other enterococci	211 (129 - 327)	1.7 (0.9 - 2.9)
Madagascar	Proteus spp.	291 (175 - 463)	3.0 (1.8 - 4.8)
Madagascar	Providencia spp.	11 (5 - 18)	0.1 (0.1 - 0.2)
Madagascar	Pseudomonas aeruginosa	2,070 (1,484 - 2,764)	14.7 (10.2 - 20.5)
Madagascar	Salmonella Paratyphi	0 (0 - 1)	0.0 (0.0 - 0.0)
Madagascar	Salmonella Typhi	1,534 (990 - 2,166)	5.4 (3.5 - 7.7)
Madagascar	Serratia spp.	404 (253 - 606)	2.5 (1.5 - 3.9)
Madagascar	Shigella spp.	1,077 (404 - 2,301)	4.6 (1.7 - 10.1)
Madagascar	Staphylococcus aureus	4,247 (3,197 - 5,531)	32.5 (24.5 - 43.2)
Madagascar	Streptococcus pneumoniae	4,451 (3,416 - 5,660)	24.5 (19.0 - 30.9)
Madagascar	Vibrio cholerae	1,858 (789 - 3,990)	12.3 (4.6 - 30.7)
Malawi	Acinetobacter baumannii	935 (564 - 1,448)	13.0 (7.7 - 20.3)
Malawi	Aeromonas spp.	133 (57 - 259)	1.0 (0.5 - 2.0)
Malawi	Campylobacter spp.	1,333 (433 - 2,817)	14.3 (3.6 - 34.6)
Malawi	Chlamydia spp.	258 (186 - 360)	2.1 (1.6 - 2.8)
Malawi	Citrobacter spp.	93 (56 - 147)	0.9 (0.5 - 1.5)
Malawi	Clostridioides difficile	7 (2 - 19)	0.0 (0.0 - 0.1)
Malawi	Enterobacter spp.	520 (367 - 747)	5.7 (3.9 - 8.1)
Malawi	Enterococcus faecalis	494 (311 - 746)	5.2 (3.0 - 8.2)
Malawi	Enterococcus faecium	376 (230 - 603)	4.6 (2.7 - 7.4)
Malawi	Escherichia coli	2,503 (1,912 - 3,268)	26.0 (19.8 - 33.5)
Malawi	Group A Streptococcus	490 (283 - 810)	5.4 (2.6 - 10.3)
Malawi	Group B Streptococcus	1,090 (798 - 1,472)	8.2 (6.3 - 11.0)
Malawi	Haemophilus influenzae	432 (333 - 571)	3.1 (2.5 - 3.8)
Malawi	Klebsiella pneumoniae	3,069 (2,316 - 3,984)	28.8 (21.9 - 37.3)
Malawi	Legionella spp.	105 (63 - 178)	0.9 (0.6 - 1.3)
Malawi	Listeria monocytogenes	131 (89 - 184)	1.0 (0.7 - 1.4)
Malawi	Morganella spp.	5 (3 - 9)	0.1 (0.0 - 0.1)
Malawi	Mycoplasma spp.	196 (148 - 257)	1.3 (1.0 - 1.6)
Malawi	Neisseria gonorrhoeae	10 (7 - 15)	0.1 (0.1 - 0.1)

Malawi	Neisseria meningitidis	933 (674 - 1,298)	5.2 (3.9 - 7.0)
Malawi	Non-typhoidal Salmonella	1,320 (791 - 2,001)	7.4 (4.2 - 12.2)
Malawi	Other Klebsiella species	141 (61 - 261)	1.9 (0.8 - 3.5)
Malawi	Other enterococci	161 (102 - 248)	1.7 (1.0 - 2.8)
Malawi	Proteus spp.	227 (141 - 349)	3.2 (1.9 - 4.9)
Malawi	Providencia spp.	8 (4 - 14)	0.1 (0.1 - 0.2)
Malawi	Pseudomonas aeruginosa	1,440 (1,059 - 1,904)	14.7 (10.7 - 19.6)
Malawi	Salmonella Paratyphi	0 (0 - 0)	0.0 (0.0 - 0.0)
Malawi	Salmonella Typhi	921 (604 - 1,346)	4.8 (3.1 - 6.9)
Malawi	Serratia spp.	291 (186 - 440)	2.6 (1.6 - 3.9)
Malawi	Shigella spp.	562 (214 - 1,134)	4.8 (1.7 - 10.1)
Malawi	Staphylococcus aureus	3,080 (2,408 - 3,922)	33.6 (26.7 - 42.2)
Malawi	Streptococcus pneumoniae	3,071 (2,436 - 3,888)	24.1 (19.9 - 28.8)
Malawi	Vibrio cholerae	363 (170 - 746)	3.5 (1.5 - 7.7)
Mozambique	Acinetobacter baumannii	1,938 (1,144 - 3,035)	17.5 (9.9 - 27.9)
Mozambique	Aeromonas spp.	141 (48 - 312)	0.5 (0.2 - 1.1)
Mozambique	Campylobacter spp.	413 (117 - 1,012)	2.4 (0.5 - 6.2)
Mozambique	Chlamydia spp.	484 (338 - 675)	2.3 (1.7 - 3.1)
Mozambique	Citrobacter spp.	170 (104 - 265)	0.9 (0.5 - 1.4)
Mozambique	Clostridioides difficile	15 (4 - 39)	0.0 (0.0 - 0.1)
Mozambique	Enterobacter spp.	885 (625 - 1,269)	5.7 (3.9 - 8.3)
Mozambique	Enterococcus faecalis	873 (573 - 1,269)	5.1 (3.0 - 8.0)
Mozambique	Enterococcus faecium	603 (378 - 978)	4.3 (2.5 - 7.1)
Mozambique	Escherichia coli	4,104 (3,075 - 5,421)	25.0 (18.7 - 33.4)
Mozambique	Group A Streptococcus	1,015 (610 - 1,659)	6.6 (3.1 - 12.6)
Mozambique	Group B Streptococcus	1,987 (1,454 - 2,797)	8.6 (6.3 - 11.9)
Mozambique	Haemophilus influenzae	778 (575 - 1,027)	3.2 (2.5 - 4.0)
Mozambique	Klebsiella pneumoniae	5,807 (4,438 - 7,594)	31.1 (23.1 - 41.1)
Mozambique	Legionella spp.	193 (109 - 336)	0.9 (0.6 - 1.4)
Mozambique	Listeria monocytogenes	159 (103 - 241)	0.8 (0.5 - 1.1)
Mozambique	Morganella spp.	10 (5 - 17)	0.1 (0.1 - 0.2)
Mozambique	Mycoplasma spp.	343 (252 - 457)	1.3 (1.0 - 1.6)
Mozambique	Neisseria gonorrhoeae	24 (14 - 38)	0.1 (0.1 - 0.2)
Mozambique	Neisseria meningitidis	1,905 (1,346 - 2,672)	5.7 (4.1 - 7.8)
Mozambique	Non-typhoidal Salmonella	1,822 (1,211 - 2,653)	5.6 (3.6 - 8.2)
Mozambique	Other Klebsiella species	196 (86 - 372)	1.6 (0.7 - 3.1)
Mozambique	Other enterococci	323 (208 - 503)	2.0 (1.2 - 3.4)
Mozambique	Proteus spp.	385 (245 - 587)	3.3 (2.0 - 5.2)
Mozambique	Providencia spp.	17 (9 - 30)	0.2 (0.1 - 0.3)
Mozambique	Pseudomonas aeruginosa	2,697 (1,998 - 3,600)	16.0 (11.4 - 21.9)

Mozambique	Salmonella Paratyphi	1 (0 - 3)	0.0 (0.0 - 0.0)
Mozambique	Salmonella Typhi	2,807 (1,900 - 4,042)	7.7 (5.2 - 10.9)
Mozambique	Serratia spp.	589 (383 - 889)	2.8 (1.7 - 4.4)
Mozambique	Shigella spp.	999 (331 - 2,108)	4.4 (1.4 - 9.5)
Mozambique	Staphylococcus aureus	5,512 (4,253 - 7,120)	36.3 (27.8 - 47.1)
Mozambique	Streptococcus pneumoniae	5,496 (4,227 - 7,033)	25.0 (19.8 - 31.0)
Mozambique	Vibrio cholerae	306 (124 - 693)	1.8 (0.7 - 4.4)
Rwanda	Acinetobacter baumannii	591 (359 - 919)	11.4 (6.7 - 17.8)
Rwanda	Aeromonas spp.	64 (25 - 134)	0.7 (0.3 - 1.3)
Rwanda	Campylobacter spp.	295 (75 - 702)	4.3 (0.9 - 11.4)
Rwanda	Chlamydia spp.	146 (105 - 202)	1.9 (1.4 - 2.5)
Rwanda	Citrobacter spp.	59 (36 - 93)	0.8 (0.5 - 1.3)
Rwanda	Clostridioides difficile	8 (2 - 18)	0.1 (0.0 - 0.1)
Rwanda	Enterobacter spp.	337 (234 - 486)	5.2 (3.6 - 7.5)
Rwanda	Enterococcus faecalis	333 (204 - 501)	4.9 (2.8 - 7.8)
Rwanda	Enterococcus faecium	275 (167 - 440)	4.4 (2.7 - 7.2)
Rwanda	Escherichia coli	1,510 (1,125 - 2,019)	23.1 (17.1 - 30.9)
Rwanda	Group A Streptococcus	345 (190 - 594)	5.6 (2.6 - 10.7)
Rwanda	Group B Streptococcus	566 (428 - 759)	6.7 (5.0 - 9.4)
Rwanda	Haemophilus influenzae	232 (177 - 302)	2.6 (2.1 - 3.3)
Rwanda	Klebsiella pneumoniae	1,817 (1,363 - 2,412)	25.6 (19.3 - 33.6)
Rwanda	Legionella spp.	76 (46 - 125)	1.0 (0.6 - 1.4)
Rwanda	Listeria monocytogenes	59 (40 - 87)	0.7 (0.5 - 0.9)
Rwanda	Morganella spp.	4 (2 - 6)	0.1 (0.0 - 0.1)
Rwanda	Mycoplasma spp.	118 (88 - 156)	1.2 (0.9 - 1.5)
Rwanda	Neisseria gonorrhoeae	8 (5 - 12)	0.1 (0.1 - 0.1)
Rwanda	Neisseria meningitidis	433 (300 - 626)	3.6 (2.6 - 5.1)
Rwanda	Non-typhoidal Salmonella	274 (145 - 509)	2.7 (1.4 - 5.4)
Rwanda	Other Klebsiella species	99 (46 - 178)	1.7 (0.8 - 3.1)
Rwanda	Other enterococci	106 (66 - 167)	1.7 (1.0 - 2.8)
Rwanda	Proteus spp.	159 (97 - 246)	3.0 (1.8 - 4.5)
Rwanda	Providencia spp.	5 (2 - 9)	0.1 (0.0 - 0.2)
Rwanda	Pseudomonas aeruginosa	934 (695 - 1,257)	14.1 (10.3 - 19.0)
Rwanda	Salmonella Paratyphi	0 (0 - 1)	0.0 (0.0 - 0.0)
Rwanda	Salmonella Typhi	562 (368 - 838)	4.3 (2.8 - 6.4)
Rwanda	Serratia spp.	176 (110 - 273)	2.3 (1.4 - 3.5)
Rwanda	Shigella spp.	172 (59 - 386)	2.0 (0.7 - 4.4)
Rwanda	Staphylococcus aureus	2,063 (1,610 - 2,635)	32.8 (25.8 - 41.6)
Rwanda	Streptococcus pneumoniae	1,706 (1,347 - 2,172)	20.5 (16.5 - 25.0)
Rwanda	Vibrio cholerae	348 (139 - 710)	4.9 (1.8 - 10.9)

Somalia	Acinetobacter baumannii	1,433 (903 - 2,171)	16.4 (9.7 - 26.1)
Somalia	Aeromonas spp.	331 (128 - 683)	1.9 (0.8 - 3.6)
Somalia	Campylobacter spp.	1,688 (524 - 3,715)	14.8 (3.4 - 38.7)
Somalia	Chlamydia spp.	891 (568 - 1,290)	4.3 (2.9 - 6.0)
Somalia	Citrobacter spp.	298 (167 - 478)	1.9 (1.1 - 3.2)
Somalia	Clostridioides difficile	6 (2 - 16)	0.0 (0.0 - 0.1)
Somalia	Enterobacter spp.	1,273 (881 - 1,860)	9.4 (6.1 - 14.0)
Somalia	Enterococcus faecalis	561 (334 - 883)	5.3 (2.9 - 9.2)
Somalia	Enterococcus faecium	345 (191 - 609)	4.3 (2.3 - 7.7)
Somalia	Escherichia coli	3,812 (2,771 - 5,243)	27.2 (19.6 - 37.4)
Somalia	Group A Streptococcus	597 (348 - 985)	6.8 (3.0 - 13.6)
Somalia	Group B Streptococcus	4,467 (2,940 - 6,444)	19.9 (13.3 - 28.8)
Somalia	Haemophilus influenzae	1,375 (967 - 1,864)	5.8 (4.2 - 7.8)
Somalia	Klebsiella pneumoniae	4,720 (3,411 - 6,404)	30.8 (22.0 - 42.8)
Somalia	Legionella spp.	54 (26 - 107)	0.3 (0.2 - 0.5)
Somalia	Listeria monocytogenes	189 (117 - 301)	1.2 (0.7 - 1.9)
Somalia	Morganella spp.	7 (3 - 13)	0.1 (0.1 - 0.2)
Somalia	Mycoplasma spp.	581 (393 - 828)	2.2 (1.6 - 3.0)
Somalia	Neisseria gonorrhoeae	24 (14 - 38)	0.2 (0.1 - 0.4)
Somalia	Neisseria meningitidis	2,955 (2,036 - 4,129)	13.0 (9.0 - 18.5)
Somalia	Non-typhoidal Salmonella	2,798 (1,730 - 4,339)	14.1 (8.1 - 23.2)
Somalia	Other Klebsiella species	158 (60 - 325)	2.2 (0.8 - 4.4)
Somalia	Other enterococci	266 (162 - 425)	2.4 (1.4 - 4.2)
Somalia	Proteus spp.	257 (147 - 431)	3.8 (2.1 - 6.5)
Somalia	Providencia spp.	15 (7 - 28)	0.3 (0.1 - 0.5)
Somalia	Pseudomonas aeruginosa	1,875 (1,319 - 2,565)	13.9 (9.4 - 20.0)
Somalia	Salmonella Paratyphi	1 (0 - 1)	0.0 (0.0 - 0.0)
Somalia	Salmonella Typhi	1,971 (1,221 - 2,952)	7.7 (4.8 - 11.5)
Somalia	Serratia spp.	627 (380 - 980)	4.4 (2.5 - 7.1)
Somalia	Shigella spp.	1,343 (518 - 2,993)	8.5 (3.0 - 19.7)
Somalia	Staphylococcus aureus	2,705 (1,965 - 3,693)	21.4 (15.4 - 30.4)
Somalia	Streptococcus pneumoniae	9,705 (6,968 - 13,237)	43.8 (32.9 - 58.1)
Somalia	Vibrio cholerae	198 (66 - 472)	1.6 (0.5 - 4.4)
South Sudan	Acinetobacter baumannii	659 (432 - 966)	13.7 (8.5 - 20.9)
South Sudan	Aeromonas spp.	130 (52 - 257)	1.7 (0.8 - 3.1)
South Sudan	Campylobacter spp.	703 (218 - 1,540)	12.9 (3.1 - 31.3)
South Sudan	Chlamydia spp.	421 (286 - 596)	4.0 (2.9 - 5.5)
South Sudan	Citrobacter spp.	136 (77 - 213)	1.7 (1.0 - 2.7)
South Sudan	Clostridioides difficile	3 (1 - 8)	0.0 (0.0 - 0.1)
South Sudan	Enterobacter spp.	541 (380 - 770)	7.7 (5.2 - 11.1)

South Sudan	Enterococcus faecalis	251 (154 - 385)	4.7 (2.5 - 7.6)
South Sudan	Enterococcus faecium	164 (92 - 275)	3.8 (2.1 - 6.6)
South Sudan	Escherichia coli	1,550 (1,140 - 2,086)	22.5 (16.5 - 29.6)
South Sudan	Group A Streptococcus	279 (160 - 472)	5.8 (2.7 - 11.1)
South Sudan	Group B Streptococcus	1,265 (909 - 1,755)	11.6 (8.4 - 16.0)
South Sudan	Haemophilus influenzae	514 (378 - 691)	4.4 (3.4 - 5.7)
South Sudan	Klebsiella pneumoniae	1,899 (1,420 - 2,507)	24.4 (18.2 - 32.7)
South Sudan	Legionella spp.	40 (20 - 76)	0.4 (0.2 - 0.6)
South Sudan	Listeria monocytogenes	88 (52 - 134)	0.9 (0.6 - 1.4)
South Sudan	Morganella spp.	3 (1 - 5)	0.1 (0.0 - 0.2)
South Sudan	Mycoplasma spp.	274 (198 - 378)	2.2 (1.6 - 3.0)
South Sudan	Neisseria gonorrhoeae	6 (4 - 13)	0.1 (0.1 - 0.2)
South Sudan	Neisseria meningitidis	1,083 (754 - 1,529)	9.3 (6.6 - 12.8)
South Sudan	Non-typhoidal Salmonella	1,027 (632 - 1,594)	10.8 (6.2 - 18.7)
South Sudan	Other Klebsiella species	71 (29 - 143)	1.8 (0.7 - 3.7)
South Sudan	Other enterococci	123 (76 - 193)	2.1 (1.2 - 3.5)
South Sudan	Proteus spp.	119 (68 - 186)	3.1 (1.8 - 5.0)
South Sudan	Providencia spp.	5 (2 - 9)	0.2 (0.1 - 0.3)
South Sudan	Pseudomonas aeruginosa	809 (592 - 1,089)	11.6 (8.1 - 15.9)
South Sudan	Salmonella Paratyphi	0 (0 - 1)	0.0 (0.0 - 0.0)
South Sudan	Salmonella Typhi	606 (381 - 893)	5.0 (3.2 - 7.3)
South Sudan	Serratia spp.	258 (162 - 392)	3.5 (2.1 - 5.6)
South Sudan	Shigella spp.	533 (187 - 1,078)	7.5 (2.7 - 15.9)
South Sudan	Staphylococcus aureus	1,196 (897 - 1,616)	18.1 (13.2 - 24.7)
South Sudan	Streptococcus pneumoniae	3,513 (2,625 - 4,688)	33.1 (26.1 - 42.4)
South Sudan	Vibrio cholerae	235 (96 - 491)	4.1 (1.6 - 9.3)
Uganda	Acinetobacter baumannii	1,823 (1,139 - 2,728)	11.5 (6.8 - 17.8)
Uganda	Aeromonas spp.	189 (77 - 380)	0.7 (0.3 - 1.3)
Uganda	Campylobacter spp.	1,029 (307 - 2,295)	5.4 (1.2 - 13.9)
Uganda	Chlamydia spp.	599 (412 - 845)	2.2 (1.6 - 2.8)
Uganda	Citrobacter spp.	254 (154 - 397)	0.9 (0.6 - 1.4)
Uganda	Clostridioides difficile	21 (7 - 52)	0.0 (0.0 - 0.1)
Uganda	Enterobacter spp.	1,264 (902 - 1,775)	5.7 (4.0 - 8.1)
Uganda	Enterococcus faecalis	1,085 (721 - 1,570)	4.7 (2.8 - 7.2)
Uganda	Enterococcus faecium	751 (472 - 1,190)	4.1 (2.5 - 6.6)
Uganda	Escherichia coli	4,671 (3,489 - 6,197)	21.8 (16.4 - 29.1)
Uganda	Group A Streptococcus	1,196 (741 - 1,868)	5.4 (2.6 - 10.1)
Uganda	Group B Streptococcus	2,705 (1,958 - 3,681)	8.3 (6.2 - 11.2)
Uganda	Haemophilus influenzae	940 (678 - 1,280)	2.9 (2.3 - 3.6)
Uganda	Klebsiella pneumoniae	6,464 (4,846 - 8,555)	25.6 (19.2 - 33.6)
Uganda	Legionella spp.	218 (122 - 390)	0.8 (0.5 - 1.1)

Uganda	Listeria monocytogenes	220 (144 - 332)	0.7 (0.5 - 0.9)
Uganda	Morganella spp.	9 (4 - 14)	0.1 (0.0 - 0.1)
Uganda	Mycoplasma spp.	474 (344 - 642)	1.3 (1.0 - 1.7)
Uganda	Neisseria gonorrhoeae	20 (13 - 31)	0.1 (0.1 - 0.1)
Uganda	Neisseria meningitidis	2,310 (1,577 - 3,367)	4.7 (3.4 - 6.6)
Uganda	Non-typhoidal Salmonella	1,379 (820 - 2,205)	3.5 (1.9 - 6.6)
Uganda	Other Klebsiella species	224 (101 - 411)	1.5 (0.7 - 2.8)
Uganda	Other enterococci	435 (276 - 656)	1.8 (1.1 - 2.9)
Uganda	Proteus spp.	435 (281 - 649)	2.8 (1.7 - 4.3)
Uganda	Providencia spp.	13 (6 - 21)	0.1 (0.1 - 0.2)
Uganda	Pseudomonas aeruginosa	3,163 (2,308 - 4,196)	14.0 (10.2 - 19.0)
Uganda	Salmonella Paratyphi	1 (0 - 3)	0.0 (0.0 - 0.0)
Uganda	Salmonella Typhi	2,361 (1,548 - 3,410)	4.5 (2.9 - 6.3)
Uganda	Serratia spp.	769 (504 - 1,131)	2.6 (1.6 - 4.0)
Uganda	Shigella spp.	793 (283 - 1,658)	3.1 (1.0 - 6.8)
Uganda	Staphylococcus aureus	5,723 (4,415 - 7,418)	28.7 (22.2 - 36.6)
Uganda	Streptococcus pneumoniae	6,665 (5,002 - 8,816)	23.0 (18.6 - 27.8)
Uganda	Vibrio cholerae	1,223 (560 - 2,457)	5.4 (2.1 - 12.1)
United Republic of Tanzania	Acinetobacter baumannii	2,777 (1,724 - 4,245)	10.7 (6.3 - 16.4)
United Republic of Tanzania	Aeromonas spp.	143 (64 - 271)	0.4 (0.2 - 0.9)
United Republic of Tanzania	Campylobacter spp.	3,013 (871 - 6,605)	10.8 (2.4 - 26.4)
United Republic of Tanzania	Chlamydia spp.	900 (638 - 1,248)	2.0 (1.5 - 2.7)
United Republic of Tanzania	Citrobacter spp.	317 (194 - 494)	0.8 (0.5 - 1.3)
United Republic of Tanzania	Clostridioides difficile	31 (10 - 72)	0.0 (0.0 - 0.1)
United Republic of Tanzania	Enterobacter spp.	1,749 (1,251 - 2,499)	5.1 (3.5 - 7.3)
United Republic of Tanzania	Enterococcus faecalis	1,592 (1,008 - 2,366)	4.6 (2.7 - 7.1)
United Republic of Tanzania	Enterococcus faecium	1,191 (729 - 1,908)	4.0 (2.4 - 6.4)
United Republic of Tanzania	Escherichia coli	7,446 (5,532 - 9,786)	22.5 (16.6 - 29.4)
United Republic of Tanzania	Group A Streptococcus	1,877 (1,109 - 3,071)	5.7 (2.8 - 10.7)
United Republic of Tanzania	Group B Streptococcus	3,501 (2,586 - 4,799)	7.4 (5.5 - 10.1)
United Republic of Tanzania	Haemophilus influenzae	1,514 (1,130 - 2,001)	2.9 (2.4 - 3.6)
United Republic of Tanzania	Klebsiella pneumoniae	10,007 (7,540 - 13,212)	25.6 (19.4 - 33.8)
United Republic of Tanzania	Legionella spp.	395 (237 - 686)	0.9 (0.6 - 1.3)
United Republic of Tanzania	Listeria monocytogenes	237 (157 - 357)	0.5 (0.4 - 0.8)
United Republic of Tanzania	Morganella spp.	14 (7 - 23)	0.1 (0.0 - 0.1)
United Republic of Tanzania	Mycoplasma spp.	780 (580 - 1,023)	1.4 (1.1 - 1.7)
United Republic of Tanzania	Neisseria gonorrhoeae	36 (23 - 52)	0.1 (0.1 - 0.1)
United Republic of Tanzania	Neisseria meningitidis	2,734 (1,831 - 4,067)	4.1 (2.8 - 6.0)
United Republic of Tanzania	Non-typhoidal Salmonella	1,868 (1,122 - 3,030)	3.5 (1.8 - 7.0)
United Republic of Tanzania	Other Klebsiella species	376 (173 - 688)	1.4 (0.6 - 2.6)

United Republic of Tanzania	Other enterococci	593 (371 - 925)	1.7 (1.0 - 2.9)
United Republic of Tanzania	Proteus spp.	706 (434 - 1,078)	2.7 (1.7 - 4.2)
United Republic of Tanzania	Providencia spp.	21 (10 - 36)	0.1 (0.0 - 0.2)
United Republic of Tanzania	Pseudomonas aeruginosa	5,067 (3,778 - 6,697)	14.0 (10.3 - 18.8)
United Republic of Tanzania	Salmonella Paratyphi	3 (1 - 7)	0.0 (0.0 - 0.0)
United Republic of Tanzania	Salmonella Typhi	3,999 (2,652 - 5,948)	5.7 (3.8 - 8.4)
United Republic of Tanzania	Serratia spp.	988 (631 - 1,530)	2.4 (1.4 - 3.7)
United Republic of Tanzania	Shigella spp.	1,366 (489 - 2,788)	4.3 (1.4 - 9.4)
United Republic of Tanzania	Staphylococcus aureus	10,491 (8,290 - 13,263)	31.5 (25.2 - 39.2)
United Republic of Tanzania	Streptococcus pneumoniae	10,817 (8,475 - 13,791)	22.8 (19.0 - 27.6)
United Republic of Tanzania	Vibrio cholerae	1,514 (709 - 3,090)	5.0 (2.1 - 10.9)
Zambia	Acinetobacter baumannii	837 (505 - 1,294)	12.9 (7.3 - 20.7)
Zambia	Aeromonas spp.	139 (59 - 273)	0.9 (0.4 - 1.7)
Zambia	Campylobacter spp.	320 (95 - 736)	3.4 (0.8 - 8.8)
Zambia	Chlamydia spp.	221 (163 - 300)	2.0 (1.5 - 2.7)
Zambia	Citrobacter spp.	99 (61 - 153)	1.0 (0.6 - 1.6)
Zambia	Clostridioides difficile	21 (8 - 49)	0.1 (0.0 - 0.2)
Zambia	Enterobacter spp.	530 (369 - 770)	6.3 (4.3 - 9.1)
Zambia	Enterococcus faecalis	537 (336 - 807)	6.0 (3.4 - 9.4)
Zambia	Enterococcus faecium	420 (253 - 673)	5.5 (3.2 - 9.0)
Zambia	Escherichia coli	2,361 (1,760 - 3,166)	26.7 (19.5 - 36.1)
Zambia	Group A Streptococcus	527 (306 - 888)	6.0 (2.9 - 11.5)
Zambia	Group B Streptococcus	936 (691 - 1,264)	7.8 (5.8 - 10.5)
Zambia	Haemophilus influenzae	374 (291 - 491)	2.9 (2.4 - 3.6)
Zambia	Klebsiella pneumoniae	2,852 (2,139 - 3,799)	29.3 (21.7 - 39.3)
Zambia	Legionella spp.	108 (67 - 180)	1.0 (0.7 - 1.4)
Zambia	Listeria monocytogenes	117 (82 - 166)	1.0 (0.7 - 1.4)
Zambia	Morganella spp.	5 (2 - 8)	0.1 (0.0 - 0.2)
Zambia	Mycoplasma spp.	196 (149 - 262)	1.3 (1.1 - 1.7)
Zambia	Neisseria gonorrhoeae	12 (7 - 18)	0.1 (0.1 - 0.2)
Zambia	Neisseria meningitidis	866 (618 - 1,222)	4.8 (3.6 - 6.6)
Zambia	Non-typhoidal Salmonella	584 (302 - 1,131)	3.7 (1.7 - 7.9)
Zambia	Other Klebsiella species	148 (69 - 279)	2.1 (0.9 - 3.8)
Zambia	Other enterococci	173 (110 - 268)	1.9 (1.2 - 3.2)
Zambia	Proteus spp.	229 (142 - 349)	3.5 (2.1 - 5.4)
Zambia	Providencia spp.	7 (4 - 12)	0.1 (0.1 - 0.2)
Zambia	Pseudomonas aeruginosa	1,427 (1,039 - 1,941)	16.0 (11.4 - 22.3)
Zambia	Salmonella Paratyphi	1 (0 - 1)	0.0 (0.0 - 0.0)
Zambia	Salmonella Typhi	968 (638 - 1,444)	4.7 (3.2 - 6.8)
Zambia	Serratia spp.	299 (194 - 452)	2.7 (1.7 - 4.3)

Zambia	Shigella spp.	337 (122 - 711)	2.6 (0.9 - 5.4)
Zambia	Staphylococcus aureus	2,991 (2,309 - 3,873)	36.0 (27.7 - 47.3)
Zambia	Streptococcus pneumoniae	2,822 (2,244 - 3,633)	23.7 (19.3 - 29.1)
Zambia	Vibrio cholerae	478 (236 - 901)	4.7 (2.1 - 9.8)
Botswana	Acinetobacter baumannii	121 (71 - 191)	9.8 (5.7 - 15.9)
Botswana	Aeromonas spp.	7 (2 - 16)	0.4 (0.1 - 1.0)
Botswana	Campylobacter spp.	49 (15 - 108)	3.3 (0.9 - 7.9)
Botswana	Chlamydia spp.	37 (26 - 50)	2.5 (1.8 - 3.3)
Botswana	Citrobacter spp.	13 (8 - 21)	0.9 (0.5 - 1.4)
Botswana	Clostridioides difficile	8 (3 - 19)	0.3 (0.1 - 0.8)
Botswana	Enterobacter spp.	88 (60 - 132)	6.3 (4.3 - 9.2)
Botswana	Enterococcus faecalis	70 (42 - 110)	4.9 (2.9 - 7.9)
Botswana	Enterococcus faecium	67 (40 - 108)	4.8 (2.9 - 7.9)
Botswana	Escherichia coli	284 (198 - 406)	21.7 (15.0 - 30.8)
Botswana	Group A Streptococcus	81 (41 - 147)	6.2 (2.9 - 11.8)
Botswana	Group B Streptococcus	124 (89 - 169)	8.1 (5.8 - 11.2)
Botswana	Haemophilus influenzae	51 (39 - 67)	3.3 (2.5 - 4.2)
Botswana	Klebsiella pneumoniae	341 (247 - 477)	24.5 (17.7 - 34.5)
Botswana	Legionella spp.	26 (17 - 36)	1.7 (1.2 - 2.3)
Botswana	Listeria monocytogenes	7 (5 - 11)	0.4 (0.3 - 0.6)
Botswana	Morganella spp.	1 (0 - 1)	0.1 (0.0 - 0.1)
Botswana	Mycoplasma spp.	46 (34 - 63)	2.5 (1.9 - 3.4)
Botswana	Neisseria gonorrhoeae	1 (1 - 2)	0.1 (0.1 - 0.1)
Botswana	Neisseria meningitidis	65 (43 - 96)	3.1 (2.1 - 4.5)
Botswana	Non-typhoidal Salmonella	65 (28 - 138)	3.6 (1.5 - 8.1)
Botswana	Other Klebsiella species	17 (8 - 32)	1.3 (0.6 - 2.2)
Botswana	Other enterococci	26 (16 - 43)	2.1 (1.2 - 3.3)
Botswana	Proteus spp.	31 (19 - 49)	2.6 (1.6 - 4.1)
Botswana	Providencia spp.	1 (0 - 1)	0.1 (0.0 - 0.1)
Botswana	Pseudomonas aeruginosa	230 (162 - 327)	17.0 (12.1 - 24.1)
Botswana	Salmonella Paratyphi	0 (0 - 0)	0.0 (0.0 - 0.0)
Botswana	Salmonella Typhi	26 (16 - 40)	1.3 (0.8 - 2.0)
Botswana	Serratia spp.	36 (23 - 57)	2.4 (1.5 - 3.8)
Botswana	Shigella spp.	81 (32 - 160)	4.6 (1.7 - 9.3)
Botswana	Staphylococcus aureus	496 (358 - 668)	37.4 (27.7 - 50.1)
Botswana	Streptococcus pneumoniae	440 (332 - 587)	27.6 (21.3 - 36.3)
Botswana	Vibrio cholerae	150 (55 - 310)	10.2 (3.8 - 22.1)
Eswatini	Acinetobacter baumannii	88 (51 - 141)	17.0 (9.8 - 27.6)
Eswatini	Aeromonas spp.	7 (3 - 17)	0.9 (0.4 - 1.9)
Eswatini	Campylobacter spp.	54 (17 - 120)	7.9 (2.3 - 19.0)

Eswatini	Chlamydia spp.	19 (13 - 26)	2.5 (1.8 - 3.5)
Eswatini	Citrobacter spp.	6 (4 - 10)	1.0 (0.6 - 1.6)
Eswatini	Clostridioides difficile	1 (0 - 2)	0.1 (0.0 - 0.2)
Eswatini	Enterobacter spp.	40 (27 - 59)	6.5 (4.2 - 9.7)
Eswatini	Enterococcus faecalis	33 (20 - 52)	5.4 (3.1 - 8.6)
Eswatini	Enterococcus faecium	29 (17 - 46)	4.9 (2.9 - 8.0)
Eswatini	Escherichia coli	149 (104 - 210)	24.7 (16.7 - 35.3)
Eswatini	Group A Streptococcus	42 (22 - 77)	7.6 (3.8 - 14.3)
Eswatini	Group B Streptococcus	67 (49 - 93)	9.0 (6.3 - 12.8)
Eswatini	Haemophilus influenzae	29 (22 - 38)	3.5 (2.6 - 4.6)
Eswatini	Klebsiella pneumoniae	201 (144 - 276)	30.8 (21.4 - 43.1)
Eswatini	Legionella spp.	8 (5 - 13)	1.1 (0.8 - 1.7)
Eswatini	Listeria monocytogenes	4 (3 - 6)	0.5 (0.3 - 0.7)
Eswatini	Morganella spp.	1 (0 - 1)	0.1 (0.1 - 0.2)
Eswatini	Mycoplasma spp.	18 (13 - 24)	1.9 (1.4 - 2.6)
Eswatini	Neisseria gonorrhoeae	1 (0 - 1)	0.1 (0.1 - 0.1)
Eswatini	Neisseria meningitidis	47 (32 - 67)	4.6 (3.1 - 6.5)
Eswatini	Non-typhoidal Salmonella	63 (26 - 140)	7.4 (2.7 - 18.5)
Eswatini	Other Klebsiella species	8 (4 - 16)	1.5 (0.7 - 2.8)
Eswatini	Other enterococci	13 (8 - 21)	2.4 (1.4 - 4.0)
Eswatini	Proteus spp.	17 (10 - 27)	3.4 (2.0 - 5.3)
Eswatini	Providencia spp.	1 (0 - 1)	0.2 (0.1 - 0.3)
Eswatini	Pseudomonas aeruginosa	115 (81 - 160)	18.5 (12.7 - 26.5)
Eswatini	Salmonella Paratyphi	0 (0 - 0)	0.0 (0.0 - 0.0)
Eswatini	Salmonella Typhi	34 (21 - 50)	3.6 (2.2 - 5.3)
Eswatini	Serratia spp.	21 (13 - 32)	3.1 (1.8 - 4.9)
Eswatini	Shigella spp.	88 (38 - 169)	10.5 (4.4 - 20.6)
Eswatini	Staphylococcus aureus	233 (170 - 317)	37.9 (27.8 - 51.5)
Eswatini	Streptococcus pneumoniae	232 (173 - 308)	28.9 (21.5 - 37.5)
Eswatini	Vibrio cholerae	57 (18 - 128)	8.9 (2.5 - 21.2)
Lesotho	Acinetobacter baumannii	240 (137 - 388)	22.3 (12.7 - 36.2)
Lesotho	Aeromonas spp.	22 (9 - 41)	1.6 (0.6 - 3.1)
Lesotho	Campylobacter spp.	181 (54 - 425)	14.8 (3.8 - 36.4)
Lesotho	Chlamydia spp.	49 (34 - 69)	3.3 (2.3 - 4.6)
Lesotho	Citrobacter spp.	13 (7 - 22)	1.0 (0.6 - 1.7)
Lesotho	Clostridioides difficile	1 (0 - 2)	0.0 (0.0 - 0.1)
Lesotho	Enterobacter spp.	88 (58 - 134)	7.0 (4.6 - 10.5)
Lesotho	Enterococcus faecalis	79 (46 - 127)	6.2 (3.6 - 10.2)
Lesotho	Enterococcus faecium	66 (39 - 113)	5.3 (3.1 - 8.9)
Lesotho	Escherichia coli	386 (273 - 534)	30.5 (21.4 - 42.4)
Lesotho	Group A Streptococcus	109 (51 - 201)	9.6 (4.3 - 18.1)

Lesotho	Group B Streptococcus	167 (114 - 233)	11.3 (7.7 - 15.9)
Lesotho	Haemophilus influenzae	69 (52 - 88)	4.4 (3.4 - 5.6)
Lesotho	Klebsiella pneumoniae	533 (384 - 730)	40.1 (28.6 - 55.3)
Lesotho	Legionella spp.	24 (15 - 38)	1.6 (1.0 - 2.4)
Lesotho	Listeria monocytogenes	9 (6 - 14)	0.6 (0.4 - 0.9)
Lesotho	Morganella spp.	1 (0 - 2)	0.1 (0.0 - 0.2)
Lesotho	Mycoplasma spp.	37 (28 - 49)	2.2 (1.6 - 2.8)
Lesotho	Neisseria gonorrhoeae	2 (1 - 3)	0.1 (0.1 - 0.2)
Lesotho	Neisseria meningitidis	103 (69 - 152)	5.6 (3.8 - 8.2)
Lesotho	Non-typhoidal Salmonella	175 (60 - 427)	11.7 (3.6 - 30.2)
Lesotho	Other Klebsiella species	21 (10 - 41)	1.7 (0.8 - 3.3)
Lesotho	Other enterococci	29 (17 - 50)	2.6 (1.4 - 4.5)
Lesotho	Proteus spp.	44 (26 - 73)	4.0 (2.4 - 6.6)
Lesotho	Providencia spp.	1 (1 - 3)	0.1 (0.1 - 0.2)
Lesotho	Pseudomonas aeruginosa	282 (197 - 393)	22.0 (15.0 - 31.3)
Lesotho	Salmonella Paratyphi	0 (0 - 0)	0.0 (0.0 - 0.0)
Lesotho	Salmonella Typhi	118 (69 - 183)	6.7 (4.0 - 10.3)
Lesotho	Serratia spp.	48 (29 - 78)	3.6 (2.1 - 5.8)
Lesotho	Shigella spp.	256 (115 - 493)	18.5 (7.9 - 36.5)
Lesotho	Staphylococcus aureus	640 (475 - 847)	50.3 (37.3 - 66.3)
Lesotho	Streptococcus pneumoniae	529 (414 - 664)	34.8 (27.5 - 44.0)
Lesotho	Vibrio cholerae	126 (38 - 305)	10.3 (3.0 - 26.3)
Namibia	Acinetobacter baumannii	137 (84 - 216)	10.3 (6.3 - 16.3)
Namibia	Aeromonas spp.	11 (4 - 23)	0.6 (0.2 - 1.4)
Namibia	Campylobacter spp.	84 (23 - 197)	5.5 (1.3 - 13.6)
Namibia	Chlamydia spp.	35 (25 - 50)	2.2 (1.6 - 3.0)
Namibia	Citrobacter spp.	12 (7 - 19)	0.8 (0.5 - 1.2)
Namibia	Clostridioides difficile	4 (1 - 10)	0.2 (0.1 - 0.4)
Namibia	Enterobacter spp.	80 (54 - 115)	5.4 (3.7 - 7.8)
Namibia	Enterococcus faecalis	66 (39 - 103)	4.4 (2.5 - 7.1)
Namibia	Enterococcus faecium	61 (37 - 96)	4.3 (2.6 - 6.7)
Namibia	Escherichia coli	289 (202 - 405)	20.2 (14.2 - 28.2)
Namibia	Group A Streptococcus	75 (41 - 133)	5.2 (2.6 - 9.5)
Namibia	Group B Streptococcus	113 (80 - 156)	6.8 (4.9 - 9.4)
Namibia	Haemophilus influenzae	48 (35 - 65)	2.8 (2.1 - 3.7)
Namibia	Klebsiella pneumoniae	356 (255 - 490)	23.5 (16.8 - 32.0)
Namibia	Legionella spp.	23 (15 - 33)	1.4 (1.0 - 2.0)
Namibia	Listeria monocytogenes	6 (4 - 9)	0.4 (0.2 - 0.5)
Namibia	Morganella spp.	1 (1 - 2)	0.1 (0.0 - 0.2)
Namibia	Mycoplasma spp.	35 (24 - 48)	1.8 (1.3 - 2.5)
Namibia	Neisseria gonorrhoeae	1 (1 - 2)	0.1 (0.0 - 0.1)

Namibia	Neisseria meningitidis	62 (41 - 91)	2.8 (1.8 - 4.0)
Namibia	Non-typhoidal Salmonella	91 (34 - 212)	4.8 (1.6 - 12.3)
Namibia	Other Klebsiella species	17 (8 - 31)	1.2 (0.6 - 2.2)
Namibia	Other enterococci	26 (16 - 42)	1.8 (1.1 - 2.9)
Namibia	Proteus spp.	33 (20 - 51)	2.5 (1.5 - 3.9)
Namibia	Providencia spp.	1 (1 - 2)	0.1 (0.0 - 0.2)
Namibia	Pseudomonas aeruginosa	224 (160 - 311)	15.3 (11.0 - 21.0)
Namibia	Salmonella Paratyphi	0 (0 - 0)	0.0 (0.0 - 0.0)
Namibia	Salmonella Typhi	35 (21 - 53)	1.6 (1.0 - 2.4)
Namibia	Serratia spp.	35 (21 - 55)	2.2 (1.3 - 3.5)
Namibia	Shigella spp.	125 (47 - 254)	7.0 (2.5 - 14.2)
Namibia	Staphylococcus aureus	491 (367 - 658)	34.5 (26.3 - 45.4)
Namibia	Streptococcus pneumoniae	390 (286 - 519)	23.3 (17.7 - 30.7)
Namibia	Vibrio cholerae	84 (25 - 199)	5.4 (1.5 - 13.0)
South Africa	Acinetobacter baumannii	3,269 (2,012 - 4,913)	8.1 (4.9 - 12.3)
South Africa	Aeromonas spp.	271 (124 - 528)	0.6 (0.3 - 1.3)
South Africa	Campylobacter spp.	1,803 (640 - 3,960)	4.4 (1.5 - 9.9)
South Africa	Chlamydia spp.	793 (632 - 997)	1.8 (1.5 - 2.3)
South Africa	Citrobacter spp.	346 (224 - 502)	0.8 (0.5 - 1.2)
South Africa	Clostridioides difficile	585 (293 - 1,152)	1.0 (0.5 - 2.0)
South Africa	Enterobacter spp.	2,271 (1,601 - 3,115)	5.3 (3.7 - 7.3)
South Africa	Enterococcus faecalis	1,486 (936 - 2,188)	3.5 (2.1 - 5.2)
South Africa	Enterococcus faecium	1,387 (876 - 2,077)	3.2 (2.0 - 4.9)
South Africa	Escherichia coli	6,173 (4,474 - 8,224)	15.0 (10.9 - 20.0)
South Africa	Group A Streptococcus	2,058 (1,103 - 3,537)	5.0 (2.7 - 8.8)
South Africa	Group B Streptococcus	3,227 (2,438 - 4,250)	7.4 (5.6 - 9.9)
South Africa	Haemophilus influenzae	1,030 (864 - 1,246)	2.3 (2.0 - 2.8)
South Africa	Klebsiella pneumoniae	7,482 (5,632 - 9,732)	17.8 (13.3 - 23.3)
South Africa	Legionella spp.	339 (247 - 468)	0.8 (0.6 - 1.0)
South Africa	Listeria monocytogenes	135 (103 - 175)	0.3 (0.2 - 0.4)
South Africa	Morganella spp.	23 (11 - 40)	0.1 (0.0 - 0.1)
South Africa	Mycoplasma spp.	816 (689 - 975)	1.7 (1.4 - 2.0)
South Africa	Neisseria gonorrhoeae	30 (25 - 40)	0.1 (0.1 - 0.1)
South Africa	Neisseria meningitidis	1,584 (1,105 - 2,213)	3.1 (2.2 - 4.3)
South Africa	Non-typhoidal Salmonella	1,851 (875 - 3,921)	4.1 (1.8 - 9.0)
South Africa	Other Klebsiella species	328 (164 - 578)	0.8 (0.4 - 1.4)
South Africa	Other enterococci	734 (466 - 1,102)	1.8 (1.1 - 2.7)
South Africa	Proteus spp.	785 (494 - 1,163)	2.0 (1.3 - 2.9)
South Africa	Providencia spp.	27 (13 - 47)	0.1 (0.0 - 0.1)
South Africa	Pseudomonas aeruginosa	5,005 (3,620 - 6,669)	12.0 (8.7 - 16.1)

South Africa	Salmonella Paratyphi	0 (0 - 1)	0.0 (0.0 - 0.0)
South Africa	Salmonella Typhi	647 (415 - 927)	1.3 (0.8 - 1.8)
South Africa	Serratia spp.	960 (624 - 1,419)	2.2 (1.4 - 3.3)
South Africa	Shigella spp.	2,453 (1,154 - 4,591)	5.7 (2.6 - 11.2)
South Africa	Staphylococcus aureus	8,964 (7,066 - 11,263)	21.7 (17.1 - 27.3)
South Africa	Streptococcus pneumoniae	9,115 (7,825 - 10,736)	20.3 (17.4 - 24.1)
South Africa	Vibrio cholerae	229 (65 - 576)	0.6 (0.2 - 1.5)
Zimbabwe	Acinetobacter baumannii	1,032 (636 - 1,569)	16.1 (9.6 - 25.2)
Zimbabwe	Aeromonas spp.	72 (29 - 144)	0.8 (0.3 - 1.5)
Zimbabwe	Campylobacter spp.	538 (156 - 1,202)	7.1 (1.7 - 17.1)
Zimbabwe	Chlamydia spp.	359 (264 - 480)	3.5 (2.6 - 4.6)
Zimbabwe	Citrobacter spp.	82 (48 - 128)	1.0 (0.6 - 1.5)
Zimbabwe	Clostridioides difficile	5 (2 - 13)	0.0 (0.0 - 0.1)
Zimbabwe	Enterobacter spp.	561 (396 - 794)	6.9 (4.7 - 9.9)
Zimbabwe	Enterococcus faecalis	412 (249 - 632)	5.2 (2.9 - 8.3)
Zimbabwe	Enterococcus faecium	327 (194 - 556)	4.5 (2.6 - 7.6)
Zimbabwe	Escherichia coli	2,226 (1,655 - 2,963)	26.9 (19.6 - 36.3)
Zimbabwe	Group A Streptococcus	470 (260 - 804)	6.6 (3.2 - 12.4)
Zimbabwe	Group B Streptococcus	1,355 (1,013 - 1,818)	12.4 (9.2 - 16.5)
Zimbabwe	Haemophilus influenzae	522 (404 - 676)	4.6 (3.7 - 5.8)
Zimbabwe	Klebsiella pneumoniae	3,132 (2,421 - 4,041)	35.2 (26.8 - 46.0)
Zimbabwe	Legionella spp.	134 (83 - 215)	1.3 (0.9 - 1.8)
Zimbabwe	Listeria monocytogenes	78 (51 - 112)	0.7 (0.5 - 1.1)
Zimbabwe	Morganella spp.	4 (2 - 7)	0.1 (0.0 - 0.1)
Zimbabwe	Mycoplasma spp.	277 (213 - 359)	2.2 (1.7 - 2.8)
Zimbabwe	Neisseria gonorrhoeae	10 (6 - 15)	0.1 (0.1 - 0.2)
Zimbabwe	Neisseria meningitidis	712 (509 - 988)	5.1 (3.7 - 7.2)
Zimbabwe	Non-typhoidal Salmonella	847 (399 - 1,637)	7.6 (3.0 - 17.0)
Zimbabwe	Other Klebsiella species	114 (48 - 220)	1.6 (0.7 - 3.3)
Zimbabwe	Other enterococci	148 (90 - 236)	2.0 (1.2 - 3.4)
Zimbabwe	Proteus spp.	204 (120 - 323)	3.3 (1.9 - 5.3)
Zimbabwe	Providencia spp.	7 (4 - 12)	0.1 (0.1 - 0.2)
Zimbabwe	Pseudomonas aeruginosa	1,608 (1,214 - 2,117)	19.1 (14.0 - 25.9)
Zimbabwe	Salmonella Paratyphi	0 (0 - 0)	0.0 (0.0 - 0.0)
Zimbabwe	Salmonella Typhi	577 (375 - 853)	4.1 (2.7 - 6.2)
Zimbabwe	Serratia spp.	261 (164 - 405)	2.9 (1.8 - 4.6)
Zimbabwe	Shigella spp.	830 (359 - 1,515)	8.8 (3.5 - 17.3)
Zimbabwe	Staphylococcus aureus	3,421 (2,716 - 4,346)	42.8 (33.8 - 54.7)
Zimbabwe	Streptococcus pneumoniae	3,785 (3,051 - 4,726)	36.1 (29.3 - 44.3)
Zimbabwe	Vibrio cholerae	693 (268 - 1,428)	8.7 (3.1 - 19.1)

Benin	Acinetobacter baumannii	657 (405 - 1,008)	11.8 (7.0 - 18.3)
Benin	Aeromonas spp.	72 (24 - 173)	0.7 (0.3 - 1.4)
Benin	Campylobacter spp.	261 (60 - 704)	3.5 (0.8 - 9.5)
Benin	Chlamydia spp.	304 (205 - 434)	2.7 (2.0 - 3.6)
Benin	Citrobacter spp.	78 (43 - 126)	0.9 (0.5 - 1.5)
Benin	Clostridioides difficile	5 (1 - 14)	0.0 (0.0 - 0.1)
Benin	Enterobacter spp.	469 (315 - 682)	5.8 (3.9 - 8.3)
Benin	Enterococcus faecalis	356 (217 - 545)	4.7 (2.6 - 7.4)
Benin	Enterococcus faecium	249 (147 - 413)	4.0 (2.3 - 6.6)
Benin	Escherichia coli	2,014 (1,438 - 2,755)	23.7 (17.1 - 32.1)
Benin	Group A Streptococcus	400 (233 - 657)	5.6 (2.8 - 10.4)
Benin	Group B Streptococcus	1,319 (923 - 1,794)	10.6 (7.8 - 14.5)
Benin	Haemophilus influenzae	501 (356 - 681)	3.9 (2.9 - 5.1)
Benin	Klebsiella pneumoniae	2,786 (2,000 - 3,772)	29.4 (21.6 - 39.2)
Benin	Legionella spp.	106 (56 - 195)	1.0 (0.6 - 1.4)
Benin	Listeria monocytogenes	114 (67 - 191)	0.9 (0.6 - 1.4)
Benin	Morganella spp.	3 (2 - 5)	0.1 (0.0 - 0.1)
Benin	Mycoplasma spp.	242 (170 - 337)	1.7 (1.3 - 2.3)
Benin	Neisseria gonorrhoeae	4 (2 - 6)	0.0 (0.0 - 0.1)
Benin	Neisseria meningitidis	751 (481 - 1,118)	4.8 (3.2 - 7.1)
Benin	Non-typhoidal Salmonella	1,832 (1,002 - 2,954)	12.8 (6.4 - 22.9)
Benin	Other Klebsiella species	83 (36 - 156)	1.5 (0.7 - 2.8)
Benin	Other enterococci	136 (83 - 218)	1.8 (1.0 - 2.9)
Benin	Proteus spp.	152 (92 - 239)	2.8 (1.7 - 4.4)
Benin	Providencia spp.	5 (3 - 8)	0.1 (0.1 - 0.2)
Benin	Pseudomonas aeruginosa	1,309 (946 - 1,778)	15.3 (11.0 - 20.8)
Benin	Salmonella Paratyphi	2 (1 - 4)	0.0 (0.0 - 0.0)
Benin	Salmonella Typhi	874 (542 - 1,338)	5.3 (3.3 - 7.9)
Benin	Serratia spp.	241 (146 - 386)	2.5 (1.5 - 4.1)
Benin	Shigella spp.	615 (187 - 1,489)	6.0 (2.1 - 12.8)
Benin	Staphylococcus aureus	2,612 (1,921 - 3,505)	33.3 (25.7 - 43.6)
Benin	Streptococcus pneumoniae	3,358 (2,425 - 4,549)	29.4 (22.8 - 38.0)
Benin	Vibrio cholerae	241 (75 - 612)	3.4 (1.0 - 9.0)
Burkina Faso	Acinetobacter baumannii	1,359 (848 - 2,088)	12.2 (7.4 - 19.1)
Burkina Faso	Aeromonas spp.	155 (60 - 322)	0.8 (0.3 - 1.6)
Burkina Faso	Campylobacter spp.	665 (191 - 1,544)	4.9 (1.1 - 13.0)
Burkina Faso	Chlamydia spp.	591 (412 - 832)	2.8 (2.1 - 3.7)
Burkina Faso	Citrobacter spp.	159 (92 - 255)	1.0 (0.6 - 1.5)
Burkina Faso	Clostridioides difficile	7 (2 - 17)	0.0 (0.0 - 0.1)
Burkina Faso	Enterobacter spp.	899 (629 - 1,252)	5.9 (4.1 - 8.2)

Burkina Faso	Enterococcus faecalis	748 (483 - 1,103)	5.1 (3.0 - 8.1)
Burkina Faso	Enterococcus faecium	531 (326 - 855)	4.4 (2.5 - 7.2)
Burkina Faso	Escherichia coli	4,569 (3,433 - 6,021)	28.0 (21.1 - 36.3)
Burkina Faso	Group A Streptococcus	929 (554 - 1,467)	6.5 (3.2 - 11.8)
Burkina Faso	Group B Streptococcus	2,475 (1,780 - 3,412)	10.7 (8.0 - 14.3)
Burkina Faso	Haemophilus influenzae	1,127 (820 - 1,512)	4.4 (3.4 - 5.5)
Burkina Faso	Klebsiella pneumoniae	6,596 (4,993 - 8,732)	35.0 (26.9 - 45.1)
Burkina Faso	Legionella spp.	234 (131 - 422)	1.2 (0.8 - 1.8)
Burkina Faso	Listeria monocytogenes	340 (166 - 626)	1.4 (0.7 - 2.5)
Burkina Faso	Morganella spp.	9 (5 - 15)	0.1 (0.1 - 0.2)
Burkina Faso	Mycoplasma spp.	522 (383 - 697)	1.9 (1.5 - 2.5)
Burkina Faso	Neisseria gonorrhoeae	8 (5 - 12)	0.0 (0.0 - 0.1)
Burkina Faso	Neisseria meningitidis	1,753 (1,090 - 2,776)	5.6 (3.6 - 8.6)
Burkina Faso	Non-typhoidal Salmonella	5,235 (3,025 - 8,196)	18.8 (10.1 - 31.4)
Burkina Faso	Other Klebsiella species	165 (74 - 308)	1.6 (0.7 - 3.1)
Burkina Faso	Other enterococci	291 (184 - 447)	2.0 (1.3 - 3.3)
Burkina Faso	Proteus spp.	332 (207 - 511)	3.2 (2.0 - 5.1)
Burkina Faso	Providencia spp.	15 (8 - 24)	0.2 (0.1 - 0.3)
Burkina Faso	Pseudomonas aeruginosa	2,872 (2,177 - 3,737)	17.1 (12.8 - 22.3)
Burkina Faso	Salmonella Paratyphi	94 (33 - 202)	0.3 (0.1 - 0.6)
Burkina Faso	Salmonella Typhi	3,138 (2,001 - 4,795)	9.8 (6.3 - 14.4)
Burkina Faso	Serratia spp.	498 (308 - 773)	2.7 (1.6 - 4.4)
Burkina Faso	Shigella spp.	1,036 (374 - 2,247)	5.5 (2.0 - 11.8)
Burkina Faso	Staphylococcus aureus	6,499 (5,065 - 8,242)	41.7 (33.8 - 51.8)
Burkina Faso	Streptococcus pneumoniae	7,987 (6,026 - 10,376)	34.5 (28.2 - 42.3)
Burkina Faso	Vibrio cholerae	503 (164 - 1,274)	4.0 (1.2 - 10.1)
Cabo Verde	Acinetobacter baumannii	49 (30 - 75)	11.6 (7.2 - 17.7)
Cabo Verde	Aeromonas spp.	0 (0 - 1)	0.1 (0.0 - 0.2)
Cabo Verde	Campylobacter spp.	3 (1 - 7)	0.6 (0.1 - 1.7)
Cabo Verde	Chlamydia spp.	12 (9 - 15)	2.7 (2.2 - 3.4)
Cabo Verde	Citrobacter spp.	5 (3 - 7)	1.0 (0.6 - 1.6)
Cabo Verde	Clostridioides difficile	3 (2 - 5)	0.5 (0.3 - 1.0)
Cabo Verde	Enterobacter spp.	28 (18 - 42)	6.4 (4.1 - 9.6)
Cabo Verde	Enterococcus faecalis	13 (7 - 21)	3.0 (1.7 - 4.8)
Cabo Verde	Enterococcus faecium	12 (7 - 19)	2.8 (1.7 - 4.4)
Cabo Verde	Escherichia coli	46 (32 - 63)	10.6 (7.5 - 14.6)
Cabo Verde	Group A Streptococcus	16 (8 - 31)	3.8 (1.7 - 7.3)
Cabo Verde	Group B Streptococcus	15 (11 - 22)	3.5 (2.5 - 5.0)
Cabo Verde	Haemophilus influenzae	7 (6 - 8)	1.5 (1.3 - 1.8)
Cabo Verde	Klebsiella pneumoniae	46 (34 - 63)	10.8 (7.8 - 14.6)
Cabo Verde	Legionella spp.	5 (4 - 6)	1.1 (0.8 - 1.4)

Cabo Verde	Listeria monocytogenes	1 (1 - 2)	0.3 (0.2 - 0.4)
Cabo Verde	Morganella spp.	0 (0 - 0)	0.0 (0.0 - 0.1)
Cabo Verde	Mycoplasma spp.	10 (8 - 12)	2.2 (1.8 - 2.6)
Cabo Verde	Neisseria gonorrhoeae	0 (0 - 0)	0.0 (0.0 - 0.0)
Cabo Verde	Neisseria meningitidis	9 (6 - 13)	1.7 (1.2 - 2.5)
Cabo Verde	Non-typhoidal Salmonella	12 (7 - 20)	2.6 (1.5 - 4.3)
Cabo Verde	Other Klebsiella species	4 (2 - 7)	0.9 (0.4 - 1.5)
Cabo Verde	Other enterococci	6 (4 - 10)	1.5 (0.9 - 2.3)
Cabo Verde	Proteus spp.	6 (4 - 10)	1.6 (1.0 - 2.3)
Cabo Verde	Providencia spp.	0 (0 - 0)	0.0 (0.0 - 0.1)
Cabo Verde	Pseudomonas aeruginosa	38 (27 - 51)	8.7 (6.2 - 12.0)
Cabo Verde	Salmonella Paratyphi	0 (0 - 0)	0.0 (0.0 - 0.0)
Cabo Verde	Salmonella Typhi	9 (4 - 15)	1.6 (0.8 - 2.6)
Cabo Verde	Serratia spp.	7 (4 - 11)	1.7 (1.0 - 2.6)
Cabo Verde	Shigella spp.	4 (2 - 9)	1.0 (0.4 - 2.1)
Cabo Verde	Staphylococcus aureus	64 (49 - 83)	14.8 (11.4 - 19.3)
Cabo Verde	Streptococcus pneumoniae	66 (55 - 79)	14.8 (12.5 - 17.6)
Cabo Verde	Vibrio cholerae	4 (1 - 10)	1.0 (0.3 - 2.4)
Cameroon	Acinetobacter baumannii	1,300 (794 - 1,996)	10.9 (6.5 - 17.1)
Cameroon	Aeromonas spp.	246 (82 - 559)	0.8 (0.3 - 1.7)
Cameroon	Campylobacter spp.	810 (227 - 2,014)	3.5 (0.9 - 8.7)
Cameroon	Chlamydia spp.	495 (354 - 666)	2.5 (1.8 - 3.4)
Cameroon	Citrobacter spp.	150 (88 - 238)	0.9 (0.5 - 1.4)
Cameroon	Clostridioides difficile	37 (13 - 83)	0.1 (0.0 - 0.2)
Cameroon	Enterobacter spp.	913 (639 - 1,292)	5.7 (3.9 - 8.1)
Cameroon	Enterococcus faecalis	702 (439 - 1,072)	4.4 (2.5 - 7.2)
Cameroon	Enterococcus faecium	531 (307 - 873)	3.8 (2.2 - 6.3)
Cameroon	Escherichia coli	3,730 (2,689 - 5,012)	22.5 (16.2 - 30.5)
Cameroon	Group A Streptococcus	843 (474 - 1,407)	5.9 (2.9 - 10.8)
Cameroon	Group B Streptococcus	2,077 (1,526 - 2,734)	9.6 (7.1 - 12.9)
Cameroon	Haemophilus influenzae	783 (562 - 1,053)	3.5 (2.6 - 4.5)
Cameroon	Klebsiella pneumoniae	4,655 (3,451 - 6,143)	26.7 (19.6 - 35.7)
Cameroon	Legionella spp.	188 (111 - 317)	1.0 (0.6 - 1.4)
Cameroon	Listeria monocytogenes	163 (100 - 255)	0.8 (0.5 - 1.1)
Cameroon	Morganella spp.	8 (4 - 13)	0.1 (0.0 - 0.1)
Cameroon	Mycoplasma spp.	434 (312 - 595)	1.7 (1.3 - 2.3)
Cameroon	Neisseria gonorrhoeae	10 (5 - 17)	0.0 (0.0 - 0.1)
Cameroon	Neisseria meningitidis	1,224 (812 - 1,810)	4.2 (2.8 - 6.0)
Cameroon	Non-typhoidal Salmonella	2,890 (1,437 - 5,331)	9.8 (4.7 - 19.1)
Cameroon	Other Klebsiella species	165 (68 - 315)	1.3 (0.6 - 2.5)

Cameroon	Other enterococci	281 (179 - 433)	1.9 (1.2 - 3.1)
Cameroon	Proteus spp.	317 (195 - 493)	2.7 (1.6 - 4.3)
Cameroon	Providencia spp.	11 (6 - 19)	0.1 (0.1 - 0.2)
Cameroon	Pseudomonas aeruginosa	2,407 (1,765 - 3,209)	15.0 (10.8 - 20.4)
Cameroon	Salmonella Paratyphi	3 (1 - 6)	0.0 (0.0 - 0.0)
Cameroon	Salmonella Typhi	1,416 (889 - 2,101)	4.4 (2.8 - 6.4)
Cameroon	Serratia spp.	455 (290 - 692)	2.4 (1.5 - 4.0)
Cameroon	Shigella spp.	2,090 (677 - 4,597)	7.1 (2.6 - 14.8)
Cameroon	Staphylococcus aureus	4,777 (3,593 - 6,306)	31.8 (24.2 - 41.9)
Cameroon	Streptococcus pneumoniae	5,717 (4,199 - 7,712)	27.5 (21.4 - 35.7)
Cameroon	Vibrio cholerae	1,715 (548 - 3,911)	10.1 (3.1 - 23.8)
Chad	Acinetobacter baumannii	1,117 (753 - 1,591)	13.0 (8.0 - 19.8)
Chad	Aeromonas spp.	449 (159 - 982)	2.0 (0.9 - 3.8)
Chad	Campylobacter spp.	1,343 (428 - 3,109)	8.5 (2.3 - 19.7)
Chad	Chlamydia spp.	854 (588 - 1,191)	4.3 (3.2 - 5.9)
Chad	Citrobacter spp.	257 (151 - 407)	1.8 (1.1 - 2.8)
Chad	Clostridioides difficile	5 (2 - 12)	0.0 (0.0 - 0.1)
Chad	Enterobacter spp.	1,178 (843 - 1,617)	9.0 (6.2 - 12.9)
Chad	Enterococcus faecalis	488 (308 - 742)	5.1 (2.9 - 8.3)
Chad	Enterococcus faecium	310 (184 - 528)	4.2 (2.3 - 7.4)
Chad	Escherichia coli	3,539 (2,642 - 4,672)	25.1 (19.1 - 32.4)
Chad	Group A Streptococcus	500 (297 - 805)	5.8 (2.7 - 11.3)
Chad	Group B Streptococcus	3,662 (2,534 - 4,999)	17.2 (12.5 - 23.0)
Chad	Haemophilus influenzae	1,148 (817 - 1,539)	5.0 (3.8 - 6.4)
Chad	Klebsiella pneumoniae	4,226 (3,164 - 5,529)	28.7 (21.9 - 38.0)
Chad	Legionella spp.	71 (35 - 142)	0.4 (0.2 - 0.6)
Chad	Listeria monocytogenes	223 (108 - 398)	1.3 (0.7 - 2.2)
Chad	Morganella spp.	4 (2 - 7)	0.1 (0.0 - 0.2)
Chad	Mycoplasma spp.	621 (448 - 836)	2.6 (2.0 - 3.4)
Chad	Neisseria gonorrhoeae	6 (3 - 9)	0.1 (0.0 - 0.1)
Chad	Neisseria meningitidis	1,750 (1,185 - 2,565)	8.0 (5.6 - 11.5)
Chad	Non-typhoidal Salmonella	4,641 (2,506 - 8,642)	22.7 (11.0 - 44.1)
Chad	Other Klebsiella species	136 (58 - 267)	2.0 (0.9 - 3.9)
Chad	Other enterococci	232 (144 - 360)	2.1 (1.3 - 3.6)
Chad	Proteus spp.	211 (127 - 338)	3.3 (1.9 - 5.3)
Chad	Providencia spp.	8 (4 - 14)	0.2 (0.1 - 0.3)
Chad	Pseudomonas aeruginosa	1,701 (1,245 - 2,231)	13.3 (9.4 - 18.4)
Chad	Salmonella Paratyphi	2 (1 - 4)	0.0 (0.0 - 0.0)
Chad	Salmonella Typhi	1,255 (817 - 1,809)	5.4 (3.6 - 7.8)
Chad	Serratia spp.	501 (320 - 749)	3.8 (2.4 - 6.0)

Chad	Shigella spp.	3,593 (1,357 - 7,202)	16.8 (6.6 - 32.5)
Chad	Staphylococcus aureus	2,524 (1,901 - 3,291)	21.0 (15.8 - 27.8)
Chad	Streptococcus pneumoniae	8,649 (6,403 - 11,152)	42.6 (34.2 - 52.6)
Chad	Vibrio cholerae	691 (241 - 1,609)	6.7 (2.1 - 16.1)
Côte d'Ivoire	Acinetobacter baumannii	1,228 (749 - 1,842)	11.7 (6.8 - 17.7)
Côte d'Ivoire	Aeromonas spp.	101 (32 - 231)	0.5 (0.2 - 1.0)
Côte d'Ivoire	Campylobacter spp.	358 (85 - 919)	2.5 (0.5 - 6.5)
Côte d'Ivoire	Chlamydia spp.	599 (410 - 847)	2.9 (2.1 - 3.8)
Côte d'Ivoire	Citrobacter spp.	143 (83 - 228)	0.9 (0.5 - 1.4)
Côte d'Ivoire	Clostridioides difficile	16 (5 - 39)	0.1 (0.0 - 0.1)
Côte d'Ivoire	Enterobacter spp.	911 (618 - 1,276)	5.8 (4.0 - 8.3)
Côte d'Ivoire	Enterococcus faecalis	666 (418 - 1,023)	4.4 (2.5 - 7.1)
Côte d'Ivoire	Enterococcus faecium	460 (270 - 777)	3.7 (2.1 - 6.2)
Côte d'Ivoire	Escherichia coli	3,578 (2,578 - 4,851)	22.6 (16.5 - 30.2)
Côte d'Ivoire	Group A Streptococcus	688 (395 - 1,161)	5.3 (2.5 - 9.9)
Côte d'Ivoire	Group B Streptococcus	2,631 (1,891 - 3,652)	11.4 (8.5 - 15.0)
Côte d'Ivoire	Haemophilus influenzae	845 (608 - 1,124)	3.7 (2.8 - 4.7)
Côte d'Ivoire	Klebsiella pneumoniae	4,876 (3,605 - 6,439)	28.3 (21.2 - 37.6)
Côte d'Ivoire	Legionella spp.	198 (108 - 359)	0.9 (0.6 - 1.4)
Côte d'Ivoire	Listeria monocytogenes	126 (77 - 191)	0.7 (0.4 - 1.0)
Côte d'Ivoire	Morganella spp.	7 (4 - 11)	0.1 (0.0 - 0.1)
Côte d'Ivoire	Mycoplasma spp.	436 (312 - 589)	1.7 (1.3 - 2.2)
Côte d'Ivoire	Neisseria gonorrhoeae	7 (4 - 12)	0.0 (0.0 - 0.1)
Côte d'Ivoire	Neisseria meningitidis	1,171 (795 - 1,676)	4.2 (2.9 - 6.1)
Côte d'Ivoire	Non-typhoidal Salmonella	2,914 (1,597 - 4,626)	10.7 (5.7 - 18.2)
Côte d'Ivoire	Other Klebsiella species	152 (64 - 299)	1.3 (0.6 - 2.6)
Côte d'Ivoire	Other enterococci	256 (163 - 407)	1.8 (1.1 - 3.0)
Côte d'Ivoire	Proteus spp.	280 (169 - 445)	2.7 (1.6 - 4.3)
Côte d'Ivoire	Providencia spp.	11 (6 - 18)	0.1 (0.1 - 0.2)
Côte d'Ivoire	Pseudomonas aeruginosa	2,403 (1,756 - 3,226)	15.0 (11.0 - 20.1)
Côte d'Ivoire	Salmonella Paratyphi	2 (1 - 6)	0.0 (0.0 - 0.0)
Côte d'Ivoire	Salmonella Typhi	1,587 (1,009 - 2,360)	5.2 (3.3 - 7.6)
Côte d'Ivoire	Serratia spp.	464 (289 - 719)	2.6 (1.6 - 4.0)
Côte d'Ivoire	Shigella spp.	871 (275 - 1,919)	4.4 (1.5 - 9.1)
Côte d'Ivoire	Staphylococcus aureus	4,496 (3,356 - 5,810)	31.1 (24.2 - 39.8)
Côte d'Ivoire	Streptococcus pneumoniae	5,652 (4,178 - 7,370)	28.3 (22.1 - 35.2)
Côte d'Ivoire	Vibrio cholerae	1,271 (408 - 2,781)	8.8 (2.9 - 19.9)
Gambia	Acinetobacter baumannii	134 (83 - 202)	14.2 (8.5 - 21.8)
Gambia	Aeromonas spp.	14 (6 - 27)	1.0 (0.4 - 1.9)
Gambia	Campylobacter spp.	18 (4 - 43)	1.6 (0.3 - 4.3)

Gambia	Chlamydia spp.	33 (23 - 45)	2.5 (1.8 - 3.3)
Gambia	Citrobacter spp.	11 (7 - 17)	0.9 (0.5 - 1.4)
Gambia	Clostridioides difficile	1 (0 - 3)	0.1 (0.0 - 0.1)
Gambia	Enterobacter spp.	67 (47 - 95)	5.8 (4.0 - 8.3)
Gambia	Enterococcus faecalis	63 (40 - 94)	5.2 (3.1 - 8.1)
Gambia	Enterococcus faecium	50 (30 - 79)	4.6 (2.8 - 7.4)
Gambia	Escherichia coli	290 (211 - 384)	25.2 (18.6 - 33.7)
Gambia	Group A Streptococcus	72 (40 - 126)	6.3 (3.0 - 11.9)
Gambia	Group B Streptococcus	130 (94 - 179)	8.9 (6.5 - 12.0)
Gambia	Haemophilus influenzae	45 (34 - 59)	3.2 (2.5 - 3.9)
Gambia	Klebsiella pneumoniae	391 (295 - 509)	31.4 (23.8 - 40.7)
Gambia	Legionella spp.	16 (10 - 26)	1.2 (0.8 - 1.7)
Gambia	Listeria monocytogenes	14 (7 - 24)	0.9 (0.5 - 1.6)
Gambia	Morganella spp.	1 (0 - 1)	0.1 (0.0 - 0.1)
Gambia	Mycoplasma spp.	24 (17 - 31)	1.5 (1.1 - 1.8)
Gambia	Neisseria gonorrhoeae	1 (0 - 1)	0.0 (0.0 - 0.1)
Gambia	Neisseria meningitidis	81 (55 - 116)	3.9 (2.6 - 5.5)
Gambia	Non-typhoidal Salmonella	173 (102 - 269)	8.0 (4.8 - 12.8)
Gambia	Other Klebsiella species	16 (7 - 30)	1.5 (0.7 - 2.9)
Gambia	Other enterococci	23 (15 - 36)	2.0 (1.2 - 3.2)
Gambia	Proteus spp.	31 (19 - 47)	3.2 (1.9 - 4.9)
Gambia	Providencia spp.	1 (1 - 2)	0.1 (0.1 - 0.2)
Gambia	Pseudomonas aeruginosa	198 (147 - 261)	17.0 (12.7 - 22.7)
Gambia	Salmonella Paratyphi	0 (0 - 0)	0.0 (0.0 - 0.0)
Gambia	Salmonella Typhi	130 (87 - 186)	5.5 (3.7 - 7.8)
Gambia	Serratia spp.	39 (25 - 59)	2.8 (1.8 - 4.4)
Gambia	Shigella spp.	48 (17 - 101)	3.3 (1.1 - 6.9)
Gambia	Staphylococcus aureus	433 (338 - 546)	40.3 (31.2 - 50.5)
Gambia	Streptococcus pneumoniae	359 (278 - 451)	26.8 (21.4 - 32.8)
Gambia	Vibrio cholerae	100 (33 - 226)	7.8 (2.4 - 19.0)
Ghana	Acinetobacter baumannii	1,624 (979 - 2,479)	11.0 (6.6 - 17.2)
Ghana	Aeromonas spp.	70 (28 - 140)	0.3 (0.1 - 0.6)
Ghana	Campylobacter spp.	627 (176 - 1,425)	3.5 (0.8 - 8.7)
Ghana	Chlamydia spp.	388 (285 - 523)	2.1 (1.6 - 2.7)
Ghana	Citrobacter spp.	160 (97 - 246)	0.8 (0.5 - 1.3)
Ghana	Clostridioides difficile	77 (33 - 154)	0.2 (0.1 - 0.5)
Ghana	Enterobacter spp.	946 (659 - 1,346)	5.3 (3.7 - 7.6)
Ghana	Enterococcus faecalis	909 (571 - 1,367)	4.7 (2.8 - 7.3)
Ghana	Enterococcus faecium	742 (455 - 1,193)	4.3 (2.6 - 6.9)
Ghana	Escherichia coli	3,699 (2,731 - 5,008)	21.6 (16.0 - 29.1)
Ghana	Group A Streptococcus	976 (550 - 1,670)	5.5 (2.7 - 10.2)

Ghana	Group B Streptococcus	1,549 (1,143 - 2,063)	7.4 (5.6 - 9.9)
Ghana	Haemophilus influenzae	590 (447 - 770)	2.9 (2.3 - 3.5)
Ghana	Klebsiella pneumoniae	4,865 (3,651 - 6,392)	26.0 (20.0 - 34.2)
Ghana	Legionella spp.	228 (154 - 330)	1.2 (0.9 - 1.6)
Ghana	Listeria monocytogenes	211 (143 - 292)	1.0 (0.7 - 1.3)
Ghana	Morganella spp.	7 (4 - 12)	0.1 (0.0 - 0.1)
Ghana	Mycoplasma spp.	343 (258 - 442)	1.5 (1.2 - 1.8)
Ghana	Neisseria gonorrhoeae	12 (7 - 20)	0.0 (0.0 - 0.1)
Ghana	Neisseria meningitidis	1,165 (823 - 1,644)	3.9 (2.8 - 5.4)
Ghana	Non-typhoidal Salmonella	1,492 (807 - 2,546)	5.4 (2.7 - 10.1)
Ghana	Other Klebsiella species	235 (110 - 427)	1.4 (0.7 - 2.6)
Ghana	Other enterococci	310 (193 - 484)	1.7 (1.0 - 2.7)
Ghana	Proteus spp.	398 (244 - 611)	2.6 (1.6 - 4.1)
Ghana	Providencia spp.	9 (5 - 17)	0.1 (0.0 - 0.1)
Ghana	Pseudomonas aeruginosa	2,625 (1,919 - 3,504)	15.0 (11.1 - 20.0)
Ghana	Salmonella Paratyphi	16 (6 - 36)	0.0 (0.0 - 0.1)
Ghana	Salmonella Typhi	1,948 (1,184 - 3,134)	5.6 (3.5 - 8.8)
Ghana	Serratia spp.	495 (314 - 747)	2.3 (1.4 - 3.5)
Ghana	Shigella spp.	747 (287 - 1,522)	3.3 (1.3 - 6.8)
Ghana	Staphylococcus aureus	5,849 (4,634 - 7,442)	35.5 (28.5 - 44.9)
Ghana	Streptococcus pneumoniae	4,779 (3,726 - 6,043)	23.6 (19.6 - 28.5)
Ghana	Vibrio cholerae	752 (225 - 1,763)	4.1 (1.2 - 9.8)
Guinea	Acinetobacter baumannii	1,085 (678 - 1,590)	15.9 (9.8 - 24.3)
Guinea	Aeromonas spp.	80 (34 - 155)	0.8 (0.4 - 1.6)
Guinea	Campylobacter spp.	313 (86 - 745)	4.4 (1.0 - 11.5)
Guinea	Chlamydia spp.	575 (392 - 844)	4.7 (3.3 - 6.6)
Guinea	Citrobacter spp.	195 (110 - 307)	1.9 (1.1 - 3.0)
Guinea	Clostridioides difficile	6 (2 - 14)	0.0 (0.0 - 0.1)
Guinea	Enterobacter spp.	882 (600 - 1,251)	9.4 (6.3 - 13.6)
Guinea	Enterococcus faecalis	377 (228 - 593)	4.9 (2.8 - 7.9)
Guinea	Enterococcus faecium	255 (147 - 433)	3.9 (2.2 - 6.6)
Guinea	Escherichia coli	2,075 (1,503 - 2,815)	22.5 (16.5 - 29.9)
Guinea	Group A Streptococcus	440 (238 - 760)	6.3 (2.8 - 12.2)
Guinea	Group B Streptococcus	2,118 (1,487 - 2,916)	16.0 (11.3 - 22.0)
Guinea	Haemophilus influenzae	783 (552 - 1,057)	5.4 (4.0 - 7.0)
Guinea	Klebsiella pneumoniae	2,860 (2,073 - 3,817)	28.3 (20.7 - 37.8)
Guinea	Legionella spp.	54 (28 - 98)	0.5 (0.3 - 0.7)
Guinea	Listeria monocytogenes	146 (87 - 224)	1.2 (0.7 - 1.7)
Guinea	Morganella spp.	5 (3 - 8)	0.1 (0.0 - 0.2)
Guinea	Mycoplasma spp.	422 (296 - 572)	2.8 (2.1 - 3.8)
Guinea	Neisseria gonorrhoeae	5 (3 - 8)	0.1 (0.0 - 0.1)

Guinea	Neisseria meningitidis	1,424 (959 - 2,030)	9.7 (6.6 - 13.6)
Guinea	Non-typhoidal Salmonella	3,118 (1,901 - 4,709)	22.8 (13.3 - 37.1)
Guinea	Other Klebsiella species	104 (44 - 212)	1.7 (0.7 - 3.3)
Guinea	Other enterococci	194 (119 - 305)	2.4 (1.4 - 4.0)
Guinea	Proteus spp.	187 (110 - 299)	3.2 (1.9 - 5.2)
Guinea	Providencia spp.	9 (4 - 15)	0.2 (0.1 - 0.3)
Guinea	Pseudomonas aeruginosa	1,279 (891 - 1,756)	14.2 (9.7 - 19.9)
Guinea	Salmonella Paratyphi	1 (0 - 4)	0.0 (0.0 - 0.0)
Guinea	Salmonella Typhi	831 (505 - 1,239)	5.3 (3.3 - 7.9)
Guinea	Serratia spp.	378 (239 - 594)	4.0 (2.5 - 6.5)
Guinea	Shigella spp.	695 (252 - 1,401)	7.2 (2.6 - 15.2)
Guinea	Staphylococcus aureus	1,894 (1,371 - 2,523)	21.6 (15.7 - 28.9)
Guinea	Streptococcus pneumoniae	5,621 (4,080 - 7,501)	43.2 (32.9 - 55.5)
Guinea	Vibrio cholerae	269 (91 - 623)	3.7 (1.2 - 9.4)
Guinea-Bissau	Acinetobacter baumannii	125 (74 - 195)	18.1 (10.6 - 28.6)
Guinea-Bissau	Aeromonas spp.	17 (6 - 37)	1.2 (0.5 - 2.3)
Guinea-Bissau	Campylobacter spp.	25 (6 - 64)	2.5 (0.5 - 6.9)
Guinea-Bissau	Chlamydia spp.	42 (29 - 57)	3.4 (2.4 - 4.6)
Guinea-Bissau	Citrobacter spp.	12 (7 - 19)	1.1 (0.6 - 1.8)
Guinea-Bissau	Clostridioides difficile	1 (0 - 2)	0.0 (0.0 - 0.1)
Guinea-Bissau	Enterobacter spp.	69 (47 - 100)	7.4 (4.9 - 10.7)
Guinea-Bissau	Enterococcus faecalis	55 (34 - 85)	5.6 (3.2 - 9.1)
Guinea-Bissau	Enterococcus faecium	39 (22 - 66)	4.8 (2.7 - 8.1)
Guinea-Bissau	Escherichia coli	278 (205 - 370)	28.1 (21.0 - 37.6)
Guinea-Bissau	Group A Streptococcus	54 (30 - 90)	6.2 (2.9 - 12.1)
Guinea-Bissau	Group B Streptococcus	192 (140 - 258)	13.8 (10.1 - 18.4)
Guinea-Bissau	Haemophilus influenzae	58 (43 - 76)	4.4 (3.4 - 5.6)
Guinea-Bissau	Klebsiella pneumoniae	385 (286 - 510)	36.3 (27.3 - 48.2)
Guinea-Bissau	Legionella spp.	12 (7 - 20)	0.9 (0.6 - 1.3)
Guinea-Bissau	Listeria monocytogenes	14 (9 - 20)	1.1 (0.7 - 1.5)
Guinea-Bissau	Morganella spp.	1 (0 - 1)	0.1 (0.0 - 0.2)
Guinea-Bissau	Mycoplasma spp.	26 (19 - 34)	1.7 (1.3 - 2.2)
Guinea-Bissau	Neisseria gonorrhoeae	1 (1 - 2)	0.1 (0.0 - 0.1)
Guinea-Bissau	Neisseria meningitidis	120 (84 - 170)	6.6 (4.7 - 9.1)
Guinea-Bissau	Non-typhoidal Salmonella	160 (90 - 280)	9.4 (4.8 - 19.0)
Guinea-Bissau	Other Klebsiella species	16 (6 - 30)	2.1 (0.8 - 4.0)
Guinea-Bissau	Other enterococci	19 (12 - 30)	2.0 (1.1 - 3.4)
Guinea-Bissau	Proteus spp.	25 (15 - 41)	3.6 (2.1 - 5.8)
Guinea-Bissau	Providencia spp.	1 (1 - 2)	0.2 (0.1 - 0.3)
Guinea-Bissau	Pseudomonas aeruginosa	175 (124 - 237)	17.9 (12.6 - 24.6)

Guinea-Bissau	Salmonella Paratyphi	0 (0 - 0)	0.0 (0.0 - 0.0)
Guinea-Bissau	Salmonella Typhi	125 (80 - 186)	6.1 (3.9 - 9.1)
Guinea-Bissau	Serratia spp.	38 (23 - 58)	3.2 (1.9 - 5.1)
Guinea-Bissau	Shigella spp.	145 (49 - 323)	10.3 (3.7 - 22.1)
Guinea-Bissau	Staphylococcus aureus	344 (261 - 447)	38.6 (29.8 - 49.8)
Guinea-Bissau	Streptococcus pneumoniae	404 (310 - 517)	34.1 (26.9 - 42.5)
Guinea-Bissau	Vibrio cholerae	30 (9 - 67)	2.9 (0.9 - 6.8)
Liberia	Acinetobacter baumannii	212 (128 - 322)	10.8 (6.3 - 16.8)
Liberia	Aeromonas spp.	31 (12 - 67)	0.8 (0.3 - 1.8)
Liberia	Campylobacter spp.	115 (32 - 283)	4.0 (0.9 - 10.6)
Liberia	Chlamydia spp.	59 (41 - 84)	2.0 (1.5 - 2.7)
Liberia	Citrobacter spp.	25 (15 - 39)	0.9 (0.5 - 1.4)
Liberia	Clostridioides difficile	2 (1 - 6)	0.0 (0.0 - 0.1)
Liberia	Enterobacter spp.	132 (91 - 190)	5.3 (3.6 - 7.8)
Liberia	Enterococcus faecalis	131 (83 - 197)	4.9 (2.9 - 7.4)
Liberia	Enterococcus faecium	99 (58 - 160)	4.2 (2.5 - 7.0)
Liberia	Escherichia coli	566 (409 - 770)	22.2 (16.2 - 30.4)
Liberia	Group A Streptococcus	141 (80 - 237)	5.7 (2.8 - 10.6)
Liberia	Group B Streptococcus	253 (186 - 347)	7.7 (5.7 - 10.5)
Liberia	Haemophilus influenzae	86 (62 - 119)	2.7 (2.1 - 3.4)
Liberia	Klebsiella pneumoniae	712 (526 - 950)	25.9 (19.1 - 35.0)
Liberia	Legionella spp.	26 (16 - 45)	0.9 (0.6 - 1.3)
Liberia	Listeria monocytogenes	24 (16 - 36)	0.7 (0.5 - 1.1)
Liberia	Morganella spp.	1 (1 - 2)	0.1 (0.0 - 0.1)
Liberia	Mycoplasma spp.	44 (32 - 61)	1.2 (0.9 - 1.5)
Liberia	Neisseria gonorrhoeae	2 (1 - 4)	0.0 (0.0 - 0.1)
Liberia	Neisseria meningitidis	225 (154 - 329)	4.8 (3.5 - 6.8)
Liberia	Non-typhoidal Salmonella	492 (250 - 874)	11.3 (5.3 - 22.3)
Liberia	Other Klebsiella species	35 (15 - 69)	1.6 (0.7 - 3.1)
Liberia	Other enterococci	45 (28 - 68)	1.7 (1.1 - 2.8)
Liberia	Proteus spp.	59 (35 - 90)	2.9 (1.7 - 4.5)
Liberia	Providencia spp.	2 (1 - 3)	0.1 (0.1 - 0.2)
Liberia	Pseudomonas aeruginosa	353 (254 - 483)	13.8 (9.8 - 19.0)
Liberia	Salmonella Paratyphi	0 (0 - 1)	0.0 (0.0 - 0.0)
Liberia	Salmonella Typhi	280 (183 - 411)	5.4 (3.6 - 7.8)
Liberia	Serratia spp.	78 (51 - 118)	2.5 (1.5 - 3.9)
Liberia	Shigella spp.	277 (96 - 611)	7.3 (2.5 - 15.9)
Liberia	Staphylococcus aureus	721 (541 - 947)	30.6 (23.5 - 39.6)
Liberia	Streptococcus pneumoniae	654 (492 - 869)	21.4 (16.9 - 26.8)
Liberia	Vibrio cholerae	31 (9 - 88)	1.2 (0.3 - 3.6)

Mali	Acinetobacter baumannii	1,329 (829 - 1,992)	10.8 (6.4 - 16.9)
Mali	Aeromonas spp.	96 (41 - 185)	0.6 (0.2 - 1.2)
Mali	Campylobacter spp.	412 (114 - 1,066)	3.4 (0.6 - 9.9)
Mali	Chlamydia spp.	595 (392 - 868)	2.2 (1.5 - 3.1)
Mali	Citrobacter spp.	245 (139 - 388)	1.2 (0.7 - 1.9)
Mali	Clostridioides difficile	8 (2 - 23)	0.0 (0.0 - 0.1)
Mali	Enterobacter spp.	1,114 (773 - 1,581)	6.3 (4.2 - 9.1)
Mali	Enterococcus faecalis	895 (578 - 1,324)	5.6 (3.2 - 8.7)
Mali	Enterococcus faecium	580 (353 - 929)	4.5 (2.6 - 7.5)
Mali	Escherichia coli	4,524 (3,256 - 6,191)	25.3 (18.2 - 34.2)
Mali	Group A Streptococcus	1,139 (700 - 1,766)	7.6 (3.9 - 13.3)
Mali	Group B Streptococcus	3,208 (2,270 - 4,489)	11.7 (8.4 - 16.4)
Mali	Haemophilus influenzae	1,110 (791 - 1,526)	3.7 (2.7 - 5.0)
Mali	Klebsiella pneumoniae	6,800 (4,960 - 9,201)	32.0 (23.0 - 43.6)
Mali	Legionella spp.	167 (80 - 326)	0.6 (0.4 - 1.0)
Mali	Listeria monocytogenes	391 (194 - 693)	1.7 (0.8 - 2.9)
Mali	Morganella spp.	7 (4 - 11)	0.1 (0.0 - 0.1)
Mali	Mycoplasma spp.	488 (348 - 678)	1.5 (1.1 - 2.1)
Mali	Neisseria gonorrhoeae	8 (5 - 14)	0.1 (0.0 - 0.1)
Mali	Neisseria meningitidis	2,563 (1,643 - 3,853)	8.1 (5.2 - 12.0)
Mali	Non-typhoidal Salmonella	6,956 (4,072 - 10,569)	25.0 (14.3 - 39.2)
Mali	Other Klebsiella species	160 (67 - 309)	1.6 (0.7 - 3.1)
Mali	Other enterococci	391 (245 - 613)	2.4 (1.4 - 3.8)
Mali	Proteus spp.	356 (221 - 541)	3.3 (2.0 - 5.1)
Mali	Providencia spp.	11 (6 - 18)	0.1 (0.1 - 0.2)
Mali	Pseudomonas aeruginosa	2,838 (2,063 - 3,874)	15.1 (10.4 - 21.0)
Mali	Salmonella Paratyphi	2 (1 - 6)	0.0 (0.0 - 0.0)
Mali	Salmonella Typhi	2,541 (1,623 - 3,731)	7.9 (5.2 - 11.5)
Mali	Serratia spp.	724 (453 - 1,121)	3.5 (2.2 - 5.6)
Mali	Shigella spp.	719 (248 - 1,567)	4.2 (1.4 - 9.5)
Mali	Staphylococcus aureus	5,020 (3,720 - 6,742)	27.9 (20.6 - 37.3)
Mali	Streptococcus pneumoniae	7,861 (5,809 - 10,476)	29.7 (22.6 - 38.7)
Mali	Vibrio cholerae	758 (231 - 2,051)	5.8 (1.5 - 18.1)
Mauritania	Acinetobacter baumannii	163 (98 - 251)	7.9 (4.7 - 12.4)
Mauritania	Aeromonas spp.	19 (7 - 44)	0.6 (0.2 - 1.2)
Mauritania	Campylobacter spp.	78 (21 - 200)	2.9 (0.7 - 8.0)
Mauritania	Chlamydia spp.	56 (39 - 77)	2.0 (1.5 - 2.7)
Mauritania	Citrobacter spp.	20 (12 - 31)	0.8 (0.5 - 1.2)
Mauritania	Clostridioides difficile	6 (2 - 14)	0.1 (0.0 - 0.3)
Mauritania	Enterobacter spp.	129 (87 - 187)	5.2 (3.5 - 7.4)

Mauritania	Enterococcus faecalis	96 (57 - 148)	3.9 (2.2 - 6.2)
Mauritania	Enterococcus faecium	77 (46 - 124)	3.5 (2.0 - 5.5)
Mauritania	Escherichia coli	437 (305 - 611)	18.3 (12.9 - 24.9)
Mauritania	Group A Streptococcus	102 (56 - 177)	4.5 (2.3 - 8.3)
Mauritania	Group B Streptococcus	239 (170 - 323)	8.0 (6.0 - 10.7)
Mauritania	Haemophilus influenzae	71 (50 - 101)	2.5 (1.9 - 3.3)
Mauritania	Klebsiella pneumoniae	506 (354 - 692)	20.3 (14.5 - 27.5)
Mauritania	Legionella spp.	23 (14 - 38)	0.8 (0.5 - 1.2)
Mauritania	Listeria monocytogenes	14 (8 - 23)	0.5 (0.3 - 0.8)
Mauritania	Morganella spp.	1 (1 - 2)	0.1 (0.0 - 0.1)
Mauritania	Mycoplasma spp.	44 (30 - 62)	1.4 (1.0 - 1.8)
Mauritania	Neisseria gonorrhoeae	1 (1 - 1)	0.0 (0.0 - 0.1)
Mauritania	Neisseria meningitidis	97 (60 - 152)	2.6 (1.6 - 3.9)
Mauritania	Non-typhoidal Salmonella	201 (88 - 434)	5.8 (2.3 - 13.6)
Mauritania	Other Klebsiella species	25 (12 - 46)	1.2 (0.5 - 2.2)
Mauritania	Other enterococci	38 (23 - 59)	1.6 (1.0 - 2.5)
Mauritania	Proteus spp.	45 (27 - 69)	2.2 (1.3 - 3.4)
Mauritania	Providencia spp.	1 (1 - 2)	0.1 (0.0 - 0.1)
Mauritania	Pseudomonas aeruginosa	292 (203 - 405)	12.2 (8.7 - 16.7)
Mauritania	Salmonella Paratyphi	0 (0 - 1)	0.0 (0.0 - 0.0)
Mauritania	Salmonella Typhi	125 (70 - 208)	2.8 (1.6 - 4.4)
Mauritania	Serratia spp.	56 (35 - 87)	2.0 (1.2 - 3.2)
Mauritania	Shigella spp.	169 (58 - 360)	5.2 (1.8 - 10.7)
Mauritania	Staphylococcus aureus	544 (397 - 727)	24.3 (18.4 - 31.5)
Mauritania	Streptococcus pneumoniae	531 (378 - 748)	20.1 (15.3 - 26.2)
Mauritania	Vibrio cholerae	131 (37 - 316)	5.1 (1.3 - 12.7)
Niger	Acinetobacter baumannii	1,067 (692 - 1,549)	10.4 (6.3 - 16.0)
Niger	Aeromonas spp.	428 (153 - 945)	1.5 (0.6 - 3.0)
Niger	Campylobacter spp.	2,250 (682 - 5,146)	12.1 (3.0 - 30.7)
Niger	Chlamydia spp.	718 (493 - 990)	3.0 (2.2 - 3.9)
Niger	Citrobacter spp.	190 (109 - 311)	1.1 (0.6 - 1.8)
Niger	Clostridioides difficile	6 (2 - 17)	0.0 (0.0 - 0.1)
Niger	Enterobacter spp.	1,069 (755 - 1,486)	6.7 (4.5 - 9.3)
Niger	Enterococcus faecalis	671 (409 - 1,011)	4.9 (2.7 - 8.0)
Niger	Enterococcus faecium	448 (257 - 739)	4.2 (2.3 - 7.0)
Niger	Escherichia coli	4,710 (3,511 - 6,373)	25.8 (19.1 - 34.0)
Niger	Group A Streptococcus	713 (425 - 1,115)	5.6 (2.7 - 10.2)
Niger	Group B Streptococcus	3,910 (2,862 - 5,292)	14.1 (10.5 - 19.0)
Niger	Haemophilus influenzae	1,398 (1,008 - 1,913)	4.6 (3.5 - 6.0)
Niger	Klebsiella pneumoniae	6,471 (4,734 - 8,563)	31.9 (23.6 - 42.0)
Niger	Legionella spp.	139 (73 - 261)	0.6 (0.4 - 0.9)

Niger	Listeria monocytogenes	414 (196 - 767)	1.4 (0.7 - 2.5)
Niger	Morganella spp.	5 (3 - 9)	0.1 (0.0 - 0.1)
Niger	Mycoplasma spp.	593 (418 - 811)	1.9 (1.4 - 2.5)
Niger	Neisseria gonorrhoeae	6 (2 - 11)	0.0 (0.0 - 0.1)
Niger	Neisseria meningitidis	1,999 (1,298 - 2,941)	6.1 (4.0 - 8.8)
Niger	Non-typhoidal Salmonella	7,601 (3,597 - 14,044)	26.2 (11.2 - 52.5)
Niger	Other Klebsiella species	166 (72 - 321)	1.8 (0.8 - 3.4)
Niger	Other enterococci	260 (160 - 410)	1.8 (1.1 - 3.0)
Niger	Proteus spp.	274 (164 - 425)	3.1 (1.8 - 4.7)
Niger	Providencia spp.	9 (5 - 16)	0.1 (0.1 - 0.2)
Niger	Pseudomonas aeruginosa	2,538 (1,859 - 3,357)	14.7 (10.6 - 19.6)
Niger	Salmonella Paratyphi	2 (1 - 6)	0.0 (0.0 - 0.0)
Niger	Salmonella Typhi	1,984 (1,276 - 2,959)	6.0 (3.9 - 8.9)
Niger	Serratia spp.	530 (328 - 810)	2.9 (1.7 - 4.6)
Niger	Shigella spp.	2,791 (976 - 6,096)	10.1 (3.9 - 21.3)
Niger	Staphylococcus aureus	4,839 (3,571 - 6,328)	29.7 (22.6 - 38.7)
Niger	Streptococcus pneumoniae	9,701 (7,093 - 12,969)	36.7 (28.5 - 47.2)
Niger	Vibrio cholerae	376 (107 - 994)	2.7 (0.7 - 7.3)
Nigeria	Acinetobacter baumannii	8,049 (5,218 - 11,510)	7.2 (4.4 - 11.1)
Nigeria	Aeromonas spp.	5,017 (1,970 - 9,533)	2.1 (1.0 - 4.0)
Nigeria	Campylobacter spp.	13,550 (4,916 - 27,755)	8.2 (2.5 - 18.8)
Nigeria	Chlamydia spp.	5,858 (4,050 - 8,006)	2.6 (1.9 - 3.4)
Nigeria	Citrobacter spp.	1,747 (1,058 - 2,646)	1.2 (0.7 - 1.8)
Nigeria	Clostridioides difficile	447 (218 - 834)	0.2 (0.1 - 0.4)
Nigeria	Enterobacter spp.	10,302 (7,261 - 14,131)	6.8 (4.6 - 9.6)
Nigeria	Enterococcus faecalis	6,158 (3,821 - 9,285)	5.0 (2.7 - 8.2)
Nigeria	Enterococcus faecium	4,514 (2,606 - 7,228)	4.3 (2.4 - 7.1)
Nigeria	Escherichia coli	39,183 (29,535 - 52,036)	24.5 (18.1 - 32.9)
Nigeria	Group A Streptococcus	6,322 (3,786 - 10,425)	5.3 (2.5 - 9.8)
Nigeria	Group B Streptococcus	31,375 (22,726 - 41,427)	13.2 (10.0 - 17.1)
Nigeria	Haemophilus influenzae	10,418 (7,914 - 13,509)	4.2 (3.3 - 5.2)
Nigeria	Klebsiella pneumoniae	45,782 (34,092 - 59,000)	26.5 (19.7 - 35.3)
Nigeria	Legionella spp.	1,413 (721 - 2,711)	0.6 (0.4 - 1.0)
Nigeria	Listeria monocytogenes	2,471 (1,461 - 3,845)	1.1 (0.7 - 1.7)
Nigeria	Morganella spp.	48 (27 - 78)	0.1 (0.0 - 0.1)
Nigeria	Mycoplasma spp.	5,303 (3,999 - 7,035)	2.0 (1.6 - 2.5)
Nigeria	Neisseria gonorrhoeae	49 (31 - 78)	0.0 (0.0 - 0.1)
Nigeria	Neisseria meningitidis	13,200 (9,330 - 18,411)	5.1 (3.7 - 7.1)
Nigeria	Non-typhoidal Salmonella	53,575 (30,936 - 82,017)	21.5 (11.5 - 35.9)
Nigeria	Other Klebsiella species	1,590 (657 - 3,104)	1.7 (0.7 - 3.2)

Nigeria	Other enterococci	2,465 (1,537 - 3,745)	1.9 (1.1 - 2.9)
Nigeria	Proteus spp.	2,587 (1,534 - 3,974)	2.9 (1.7 - 4.5)
Nigeria	Providencia spp.	69 (37 - 111)	0.1 (0.0 - 0.2)
Nigeria	Pseudomonas aeruginosa	21,157 (15,773 - 27,204)	13.5 (9.8 - 18.1)
Nigeria	Salmonella Paratyphi	144 (50 - 312)	0.0 (0.0 - 0.1)
Nigeria	Salmonella Typhi	10,154 (6,637 - 15,111)	3.9 (2.6 - 5.6)
Nigeria	Serratia spp.	4,371 (2,781 - 6,455)	2.8 (1.7 - 4.2)
Nigeria	Shigella spp.	28,702 (13,636 - 46,954)	13.3 (6.2 - 24.0)
Nigeria	Staphylococcus aureus	36,693 (28,345 - 47,048)	24.3 (18.6 - 32.1)
Nigeria	Streptococcus pneumoniae	68,449 (54,133 - 86,458)	30.8 (25.0 - 37.6)
Nigeria	Vibrio cholerae	38,514 (17,704 - 63,208)	26.4 (12.2 - 47.1)
Sao Tome and Principe	Acinetobacter baumannii	11 (6 - 17)	12.0 (7.0 - 18.5)
Sao Tome and Principe	Aeromonas spp.	0 (0 - 0)	0.1 (0.0 - 0.2)
Sao Tome and Principe	Campylobacter spp.	1 (0 - 2)	0.6 (0.1 - 1.7)
Sao Tome and Principe	Chlamydia spp.	2 (2 - 3)	2.2 (1.7 - 2.9)
Sao Tome and Principe	Citrobacter spp.	1 (1 - 1)	0.8 (0.5 - 1.2)
Sao Tome and Principe	Clostridioides difficile	0 (0 - 1)	0.2 (0.1 - 0.5)
Sao Tome and Principe	Enterobacter spp.	6 (4 - 8)	5.5 (3.8 - 7.7)
Sao Tome and Principe	Enterococcus faecalis	5 (3 - 8)	4.6 (2.6 - 7.4)
Sao Tome and Principe	Enterococcus faecium	5 (3 - 7)	4.4 (2.5 - 7.0)
Sao Tome and Principe	Escherichia coli	22 (15 - 30)	21.9 (15.7 - 29.7)
Sao Tome and Principe	Group A Streptococcus	5 (3 - 8)	4.5 (2.2 - 8.4)
Sao Tome and Principe	Group B Streptococcus	8 (5 - 10)	6.6 (4.8 - 8.6)
Sao Tome and Principe	Haemophilus influenzae	3 (2 - 4)	2.9 (2.2 - 3.5)
Sao Tome and Principe	Klebsiella pneumoniae	26 (19 - 35)	25.2 (18.3 - 33.1)
Sao Tome and Principe	Legionella spp.	2 (1 - 2)	1.5 (1.0 - 1.9)
Sao Tome and Principe	Listeria monocytogenes	0 (0 - 0)	0.3 (0.2 - 0.4)
Sao Tome and Principe	Morganella spp.	0 (0 - 0)	0.1 (0.1 - 0.2)
Sao Tome and Principe	Mycoplasma spp.	2 (2 - 3)	1.6 (1.2 - 2.1)
Sao Tome and Principe	Neisseria gonorrhoeae	0 (0 - 0)	0.0 (0.0 - 0.0)
Sao Tome and Principe	Neisseria meningitidis	4 (2 - 6)	2.2 (1.3 - 3.3)
Sao Tome and Principe	Non-typhoidal Salmonella	6 (3 - 9)	3.4 (2.0 - 5.6)
Sao Tome and Principe	Other Klebsiella species	1 (1 - 3)	1.4 (0.6 - 2.5)
Sao Tome and Principe	Other enterococci	2 (1 - 3)	1.8 (1.1 - 2.8)
Sao Tome and Principe	Proteus spp.	3 (2 - 4)	2.8 (1.7 - 4.2)
Sao Tome and Principe	Providencia spp.	0 (0 - 0)	0.1 (0.1 - 0.2)
Sao Tome and Principe	Pseudomonas aeruginosa	16 (12 - 22)	16.1 (11.7 - 21.7)
Sao Tome and Principe	Salmonella Paratyphi	0 (0 - 0)	0.0 (0.0 - 0.0)
Sao Tome and Principe	Salmonella Typhi	6 (4 - 10)	3.0 (1.8 - 4.6)
Sao Tome and Principe	Serratia spp.	2 (1 - 4)	2.1 (1.2 - 3.4)

Sao Tome and Principe	Shigella spp.	1 (0 - 3)	1.0 (0.4 - 2.2)
Sao Tome and Principe	Staphylococcus aureus	38 (29 - 48)	38.9 (30.4 - 49.2)
Sao Tome and Principe	Streptococcus pneumoniae	26 (20 - 33)	23.0 (18.1 - 28.4)
Sao Tome and Principe	Vibrio cholerae	2 (1 - 4)	1.6 (0.4 - 4.1)
Senegal	Acinetobacter baumannii	832 (487 - 1,309)	11.7 (6.7 - 19.0)
Senegal	Aeromonas spp.	34 (14 - 68)	0.3 (0.1 - 0.6)
Senegal	Campylobacter spp.	137 (32 - 352)	1.6 (0.3 - 4.3)
Senegal	Chlamydia spp.	218 (151 - 306)	1.9 (1.4 - 2.5)
Senegal	Citrobacter spp.	72 (42 - 112)	0.8 (0.4 - 1.2)
Senegal	Clostridioides difficile	13 (4 - 31)	0.1 (0.0 - 0.2)
Senegal	Enterobacter spp.	432 (291 - 627)	4.8 (3.2 - 7.0)
Senegal	Enterococcus faecalis	404 (245 - 624)	4.3 (2.4 - 6.9)
Senegal	Enterococcus faecium	313 (182 - 515)	3.8 (2.1 - 6.2)
Senegal	Escherichia coli	1,963 (1,423 - 2,663)	21.2 (15.5 - 28.6)
Senegal	Group A Streptococcus	415 (238 - 709)	4.8 (2.5 - 9.2)
Senegal	Group B Streptococcus	902 (662 - 1,225)	7.4 (5.5 - 10.0)
Senegal	Haemophilus influenzae	311 (229 - 402)	2.6 (2.0 - 3.2)
Senegal	Klebsiella pneumoniae	2,457 (1,799 - 3,257)	24.8 (18.2 - 33.6)
Senegal	Legionella spp.	106 (63 - 182)	0.9 (0.6 - 1.3)
Senegal	Listeria monocytogenes	94 (58 - 141)	0.9 (0.6 - 1.3)
Senegal	Morganella spp.	6 (3 - 10)	0.1 (0.0 - 0.2)
Senegal	Mycoplasma spp.	153 (111 - 205)	1.1 (0.9 - 1.5)
Senegal	Neisseria gonorrhoeae	4 (2 - 6)	0.0 (0.0 - 0.1)
Senegal	Neisseria meningitidis	541 (367 - 777)	3.6 (2.5 - 5.1)
Senegal	Non-typhoidal Salmonella	1,158 (515 - 2,293)	8.9 (3.3 - 21.0)
Senegal	Other Klebsiella species	107 (47 - 202)	1.4 (0.6 - 2.6)
Senegal	Other enterococci	144 (89 - 226)	1.7 (1.0 - 2.7)
Senegal	Proteus spp.	193 (118 - 299)	2.7 (1.6 - 4.1)
Senegal	Providencia spp.	9 (4 - 15)	0.1 (0.1 - 0.2)
Senegal	Pseudomonas aeruginosa	1,234 (895 - 1,677)	13.2 (9.5 - 18.2)
Senegal	Salmonella Paratyphi	2 (1 - 5)	0.0 (0.0 - 0.0)
Senegal	Salmonella Typhi	817 (519 - 1,211)	4.8 (3.0 - 7.1)
Senegal	Serratia spp.	233 (145 - 363)	2.2 (1.3 - 3.5)
Senegal	Shigella spp.	1,446 (637 - 2,644)	13.2 (5.6 - 24.2)
Senegal	Staphylococcus aureus	2,592 (1,961 - 3,360)	29.7 (22.7 - 38.2)
Senegal	Streptococcus pneumoniae	2,217 (1,669 - 2,844)	20.2 (16.0 - 24.9)
Senegal	Vibrio cholerae	186 (59 - 449)	2.0 (0.6 - 5.0)
Sierra Leone	Acinetobacter baumannii	474 (286 - 735)	11.7 (6.9 - 18.6)
Sierra Leone	Aeromonas spp.	44 (13 - 100)	0.8 (0.3 - 1.7)
Sierra Leone	Campylobacter spp.	193 (38 - 508)	4.7 (0.9 - 12.8)

Sierra Leone	Chlamydia spp.	194 (135 - 274)	2.9 (2.1 - 4.0)
Sierra Leone	Citrobacter spp.	55 (30 - 89)	0.9 (0.5 - 1.5)
Sierra Leone	Clostridioides difficile	4 (1 - 10)	0.0 (0.0 - 0.1)
Sierra Leone	Enterobacter spp.	308 (209 - 447)	5.7 (3.9 - 8.4)
Sierra Leone	Enterococcus faecalis	246 (151 - 380)	4.6 (2.6 - 7.5)
Sierra Leone	Enterococcus faecium	177 (104 - 304)	3.9 (2.2 - 6.7)
Sierra Leone	Escherichia coli	1,381 (997 - 1,889)	25.0 (18.4 - 33.7)
Sierra Leone	Group A Streptococcus	304 (175 - 504)	5.9 (2.9 - 11.0)
Sierra Leone	Group B Streptococcus	802 (573 - 1,093)	10.9 (7.9 - 14.8)
Sierra Leone	Haemophilus influenzae	374 (269 - 506)	4.6 (3.5 - 6.0)
Sierra Leone	Klebsiella pneumoniae	2,013 (1,467 - 2,733)	32.3 (23.7 - 44.1)
Sierra Leone	Legionella spp.	67 (40 - 114)	1.0 (0.7 - 1.6)
Sierra Leone	Listeria monocytogenes	89 (53 - 145)	1.2 (0.8 - 1.8)
Sierra Leone	Morganella spp.	2 (1 - 4)	0.1 (0.0 - 0.1)
Sierra Leone	Mycoplasma spp.	170 (120 - 230)	2.0 (1.5 - 2.6)
Sierra Leone	Neisseria gonorrhoeae	3 (2 - 5)	0.0 (0.0 - 0.1)
Sierra Leone	Neisseria meningitidis	697 (451 - 1,046)	7.2 (4.9 - 10.8)
Sierra Leone	Non-typhoidal Salmonella	1,501 (812 - 2,413)	17.3 (8.6 - 30.4)
Sierra Leone	Other Klebsiella species	58 (25 - 114)	1.4 (0.6 - 2.7)
Sierra Leone	Other enterococci	95 (58 - 155)	1.8 (1.1 - 3.0)
Sierra Leone	Proteus spp.	110 (64 - 177)	2.8 (1.6 - 4.5)
Sierra Leone	Providencia spp.	4 (2 - 7)	0.1 (0.1 - 0.2)
Sierra Leone	Pseudomonas aeruginosa	922 (663 - 1,251)	16.2 (11.8 - 22.4)
Sierra Leone	Salmonella Paratyphi	1 (0 - 2)	0.0 (0.0 - 0.0)
Sierra Leone	Salmonella Typhi	664 (414 - 985)	6.6 (4.2 - 10.0)
Sierra Leone	Serratia spp.	167 (103 - 269)	2.6 (1.6 - 4.3)
Sierra Leone	Shigella spp.	406 (110 - 966)	7.5 (2.3 - 16.5)
Sierra Leone	Staphylococcus aureus	2,003 (1,477 - 2,654)	37.8 (29.2 - 49.2)
Sierra Leone	Streptococcus pneumoniae	2,599 (1,900 - 3,472)	34.9 (26.7 - 45.0)
Sierra Leone	Vibrio cholerae	265 (71 - 690)	5.2 (1.4 - 13.3)
Togo	Acinetobacter baumannii	375 (224 - 570)	11.4 (6.8 - 17.8)
Togo	Aeromonas spp.	77 (25 - 178)	1.0 (0.4 - 2.1)
Togo	Campylobacter spp.	252 (71 - 614)	4.4 (1.1 - 11.0)
Togo	Chlamydia spp.	129 (93 - 180)	2.5 (1.9 - 3.3)
Togo	Citrobacter spp.	35 (20 - 57)	0.8 (0.5 - 1.3)
Togo	Clostridioides difficile	6 (2 - 15)	0.1 (0.0 - 0.2)
Togo	Enterobacter spp.	228 (158 - 333)	5.4 (3.7 - 7.8)
Togo	Enterococcus faecalis	189 (113 - 291)	4.3 (2.5 - 6.9)
Togo	Enterococcus faecium	149 (87 - 251)	3.8 (2.2 - 6.5)
Togo	Escherichia coli	1,016 (751 - 1,364)	22.7 (16.8 - 30.7)
Togo	Group A Streptococcus	197 (107 - 338)	5.1 (2.4 - 9.8)

Togo	Group B Streptococcus	498 (364 - 686)	8.8 (6.6 - 11.9)
Togo	Haemophilus influenzae	179 (135 - 236)	3.2 (2.6 - 4.1)
Togo	Klebsiella pneumoniae	1,217 (892 - 1,624)	26.8 (20.1 - 35.8)
Togo	Legionella spp.	59 (35 - 98)	1.1 (0.7 - 1.6)
Togo	Listeria monocytogenes	37 (23 - 54)	0.7 (0.4 - 1.0)
Togo	Morganella spp.	2 (1 - 3)	0.1 (0.0 - 0.1)
Togo	Mycoplasma spp.	99 (74 - 132)	1.6 (1.2 - 2.0)
Togo	Neisseria gonorrhoeae	2 (1 - 4)	0.0 (0.0 - 0.1)
Togo	Neisseria meningitidis	259 (172 - 384)	3.4 (2.3 - 5.0)
Togo	Non-typhoidal Salmonella	1,134 (592 - 2,004)	14.3 (7.1 - 26.1)
Togo	Other Klebsiella species	51 (23 - 98)	1.4 (0.6 - 2.6)
Togo	Other enterococci	65 (39 - 103)	1.6 (1.0 - 2.7)
Togo	Proteus spp.	86 (52 - 137)	2.6 (1.6 - 4.1)
Togo	Providencia spp.	3 (2 - 5)	0.1 (0.1 - 0.2)
Togo	Pseudomonas aeruginosa	636 (461 - 862)	14.8 (10.9 - 20.2)
Togo	Salmonella Paratyphi	1 (0 - 2)	0.0 (0.0 - 0.0)
Togo	Salmonella Typhi	363 (229 - 537)	4.3 (2.7 - 6.2)
Togo	Serratia spp.	112 (69 - 176)	2.2 (1.4 - 3.6)
Togo	Shigella spp.	643 (216 - 1,316)	8.6 (3.2 - 17.6)
Togo	Staphylococcus aureus	1,353 (1,029 - 1,768)	34.3 (26.9 - 44.0)
Togo	Streptococcus pneumoniae	1,293 (982 - 1,697)	25.4 (20.5 - 31.2)
Togo	Vibrio cholerae	350 (112 - 821)	7.6 (2.2 - 20.2)
